# Site-Divergent Oxidations within Venerable Macrolide Antibiotic Scaffolds Unveil Compounds with Broad Spectrum and Anti-MRSA Activities

**DOI:** 10.1021/acscentsci.5c02343

**Published:** 2026-03-17

**Authors:** Olivia C. Langner, Brandon Q. Mercado, Sebastian M. Krajewski, Song Lin, Scott J. Miller

**Affiliations:** † Department of Chemistry, Yale University, 225 Prospect Street, New Haven, Connecticut 06511, United States; ‡ Department of Chemistry and Chemical Biology, 5922 Cornell University, Ithaca, New York 14853, United States

## Abstract

The synthesis of bioactive compounds with differential, and ideally enhanced, activities presents persistent and growing challenges for the field of organic synthesis. By leveraging Nature’s ability to build complex, stereochemically rich, and biologically active molecular scaffolds, site-selective modification of natural products can deliver analogs without the need for lengthy *de novo* syntheses. Yet, achieving selective reactivity at a single desired position is complicated by the presence of multiple iterations of similar reactive functional groups, thus precluding widespread adoption of catalyst-controlled site-selective modification. Herein we describe the development of complementary systems for the oxidation of secondary alcohols on erythromycin A, clarithromycin, and azithromycin using a newly designed azaadamantyl oxoammonium catalyst, wherein different hydroxyl groups show disparate reactivities under the same conditions. The application of this methodology has enabled the generation of a suite of oxidized macrolide antibiotics and derivatives that take advantage of the newly installed carbonyls. Antimicrobial activity testing revealed that multiple compounds retain activity against a broad range of pathogens consistent with erythromycin A coverage. Additionally, three of the compounds reported herein display antibiotic activity against CA-MRSA and MRSA­(*mph*(C)), for which the clinical analogs erythromycin A, clarithromycin, and azithromycin exhibit no activity at tested concentrations.

## Graphical abstract

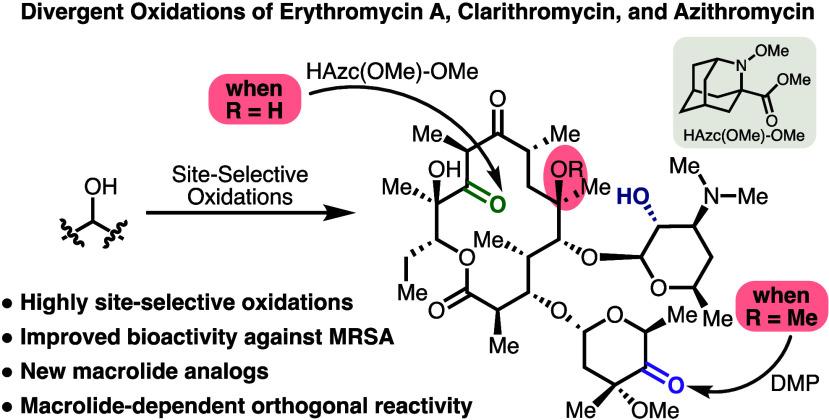

## Introduction

Erythromycin A and related macrolide derivatives make up a class of widely administered antibiotics with millions of prescriptions annually.^1,2^ This class of molecules promotes bacterial cell death via binding to the 50S subunit of the ribosome inhibiting translation.^3,4^ While naturally occurring erythromycin A has high potency, it undergoes rapid, acid-catalyzed dehydration to form an inactive spiroketal and loss of the cladinose sugar, making oral administration a challenge.^5^ To overcome this, several analogs have been designed that prevent degradation via spiroketalization; most notable are clarithromycin and azithromycin.^6,7^ Despite these advances, erythromycin-resistant pathogens retain resistance to many analogs, and, as such, these therapeutics do not address the ever-growing rates of antibiotic resistance.^8–10^ Pioneering work from the Myers group in 2016 reported a total synthesis-based platform to access a vast new array of macrolide antibiotics.^11^ In a complementary approach, semisynthetic, site-selective modifications to native erythromycin itself represent a useful strategy to rapidly access a diverse span of chemical space. However, to further expand chemical space beyond the large set of classical efforts in the literature to derivatize macrolides, new and efficient site-selective reactions are needed.^12–17^ Efforts to achieve selective transformations, however, are complicated by the presence of multiple functional groups of comparable reactivity, particularly iterations of the same functional group.^18–24^ Once one selective reaction is discovered, the challenge of achieving alternative selectivity on the same or related scaffolds can be daunting. However, complex catalyst-substrate interactions can be leveraged by identifying appropriate pairings to modify site-selectivity.^25^

Previously, our group demonstrated two different group transfer strategies on erythromycin A to access acylated and deoxygenated derivatives. With acyl transfer catalysis, it was found that, in the absence of a chiral catalyst, the C2′ hydroxyl group (blue, desosamine) was the most reactive followed by the C4′′ hydroxyl group (purple, cladinose) and the C11 hydroxyl group (green) on the macrolide, which were selectively modified using peptide based catalysts.^26,27^ A similar order of reactivity was seen with phosphoramidite transfer; however, in a notable limitation, no catalyst was identified that could functionalize the C11 hydroxyl group with this strategy.^28^ Taken together, these studies reveal the potential, and underscore the challenges, associated with site-selective modification and installation of functional groups that can be leveraged for new reactivity and diversification of bioactive scaffolds.

Site-selective oxidation of secondary alcohols resident in erythromycin A could introduce particularly auspicious, positionally non-native carbonyl groups capable of undergoing a vast lexicon of further derivatizations to alter and enhance bioactive properties. In collaboration with the Lin and Sigman groups, we recently reported the use of a peptide-embedded aminoxyl for the desymmetrative oxidative lactonization of *meso*-diols (**P1**, Figure 1A).^29^ In this work, a new amino acid-derived aminoxyl, **Azc-OMe** (Figure 1B), was designed, synthesized, and incorporated into a diverse array of peptidic scaffolds. This work culminated in the broadly selective desymmetrization of 25 *meso*-diols, likely facilitated by a combination of factors including noncovalent interactions leading to enantioselectivity. Inspired by these findings, we sought to explore this system for the site-selective oxidation of complex, functional group-rich natural products. To this end, the design of a more sterically accessible aminoxyl (**HAzc­(OMe)-OMe**), inspired by foundational work from Iwabuchi and co-workers, was undertaken (Figure 1B).^30^ Erythromycin A was selected as a target of our campaign given its accessibility and the importance of macrolide antibiotics in modern medicine (Figure 1C).

**Figure 1:**
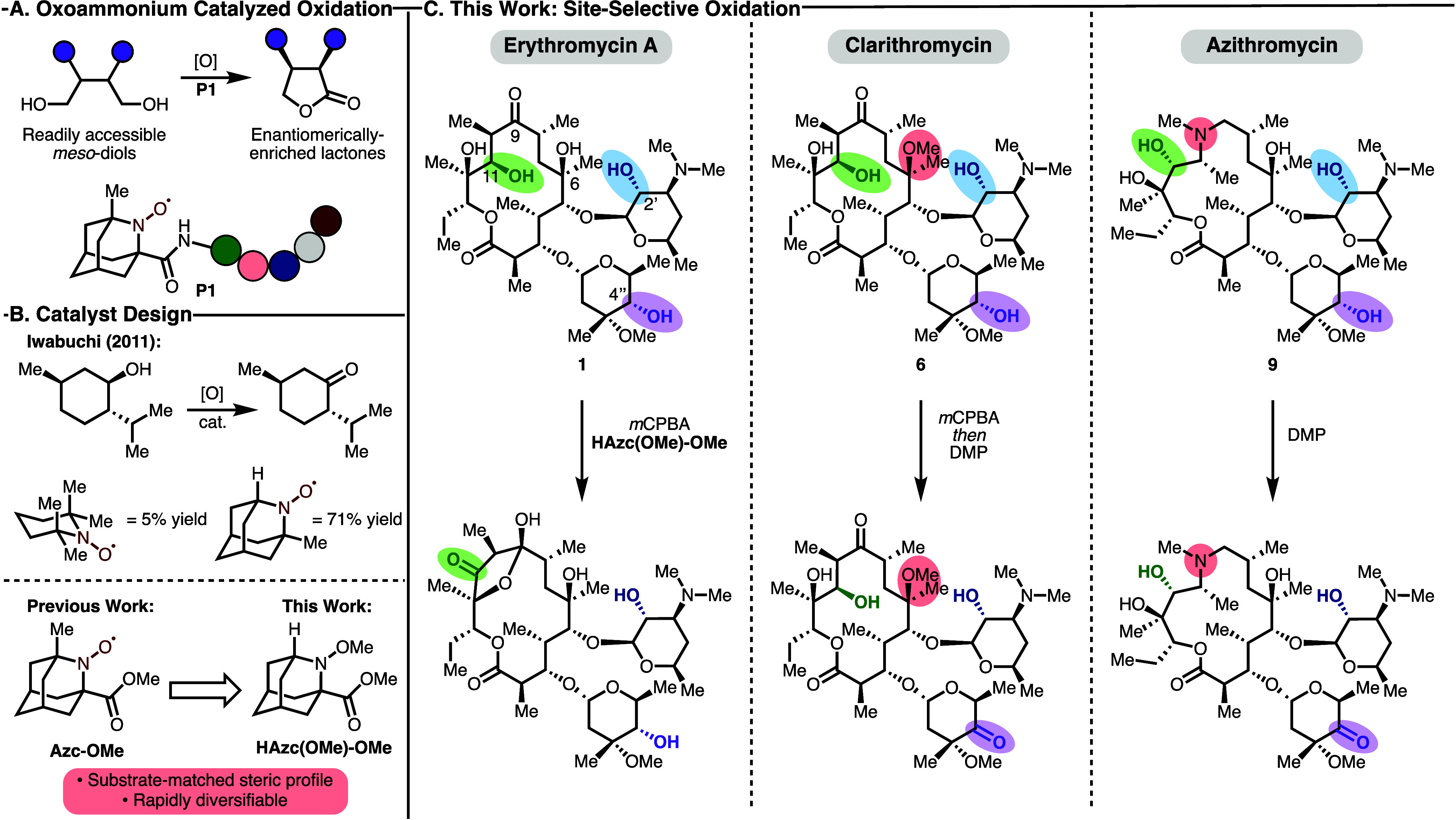
An overview of previous work and how it is applied to the work presented herein. (A) Oxoammonium catalyzed oxidation of *meso*-diols to form enantiomerically enriched lactones using a bespoke amino acid-derived aminoxyl, **Azc-OMe**.^29^ (B) Prior work showing the effect of catalyst alterations on secondary alcohol oxidation, and the catalyst design undertaken herein.^30^ (C) Outline of the site-selective oxidations of macrolide antibiotics to form novel analogs described in this work.

## Results and Discussion

### Preliminary Oxidations

Our study of this transformation began with a screen of common oxidants for their ability to efficiently and cleanly oxidize erythromycin A (**1**, Table 1) to any corresponding monooxidation products with 4-acetamido-TEMPO (ACT). Many common reagents led to rapid decomposition of **1** (Table 1, Entries 1–7). Oxidation with Dess-Martin periodinane (DMP) led to multiple oxidation products, including dioxidation, in a nonselective manner (Entry 8). In contrast, peroxide-based oxidants provided cleaner reaction profiles, with *meta*-chloroperoxybenzoic acid (*m*CPBA) inducing quantitative *in situ* conversion of the tertiary amine to the *N*-oxide **2** (Entries 9–12). With efficient access to **2**, the same oxidations were examined with **2** as the starting material. However, in no case were we able to observe efficient or selective alcohol oxidation, observing instead degradation or indiscriminate alcohol oxidations.

**Table 1:**
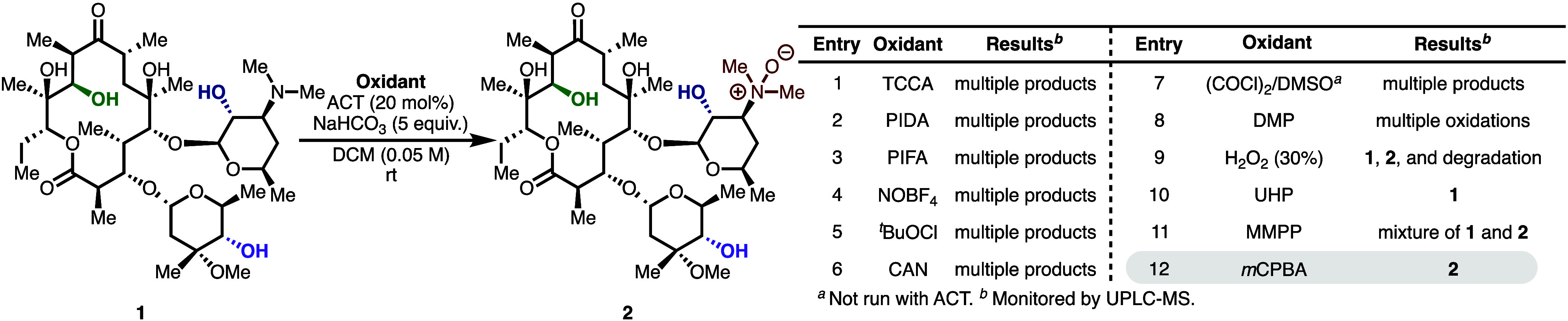
Oxidant Screening

With *m*CPBA as our terminal oxidant, we then turned our attention to oxidations with other aminoxyl-based catalysts, in analogy to our prior findings for *meso*-diol desymmetrization with an aminoxyl platform.^29^ Unfortunately, neither simple aminoxyls such as ACT or **Azc-OMe**, nor peptide-embedded variants resulted in appreciable levels of alcohol oxidation. We hypothesized that this could be due to the increased steric demand associated with oxidizing secondary hydroxyl groups, as opposed to primary alcohols, especially when embedded within the complex molecular framework presented by erythromycin A.^29,31^ Notably, seminal work from Iwabuchi and co-workers showed that decreasing steric bulk near the reactive oxoammonium site improves the yield of secondary alcohol oxidations (Figure 1B).^30^

To address this issue, a new, less sterically hindered demethylated oxoammonium ion precursor was envisioned (**HAzc-OMe**) and synthesized in 9 steps with 14% overall yield (see for full synthesis). While **HAzc-OMe** was found to be competent for oxidation reactions, the decreased steric bulk of **HAzc-OMe**, though rendering it more reactive, made it more prone to degradation during purification, saponification, and peptide couplings. Accordingly, we found that protection of the aminoxyl as its *O*-methyl hydroxylamine (**HAzc­(OMe)-OMe**) derivative could be easily achieved with FeSO_4_ and H_2_O_2_ in DMSO (50% yield, Scheme 1). The *O*-methyl hydroxylamine could then be deprotected *in situ* using *m*CPBA to access the reactive species.^32^ This protecting group allowed for facile isolation of the methyl ester and was stable to both hydrolysis and subsequent couplings to access a variety of new catalysts.

**Scheme 1:**
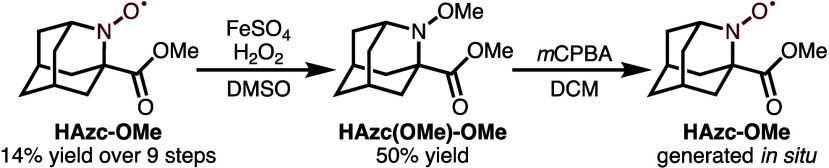
*In Situ* Generation of HAzc-OMe

### Site-Selective Oxidations of Erythromycin

With **HAzc­(OMe)-OMe** in hand, we explored the oxidation of **1**. Gratifyingly, both good reactivity and excellent site specificity were observed, with only one oxidation product detected by both crude ^1^H NMR and UPLC-MS. Conditions were then screened to improve the observed ratio of *N*-oxide **2** (formed quantitatively *in situ*) to product. The identity of the base was found to have a marked effect with dibasic sodium phosphate providing the best ratio of *N*-oxide to product (50:50, Figure 2A, Entries 1–6). The equivalents of peroxide and base were also found to have a drastic impact; three equivalents of each showed no detectable conversion, while reactivity was recovered but to a diminished extent with five equivalents (Entries 7 and 8). Conversion was found to reach a maximum by 24 hr, and the conditions from Entry 10 were used moving forward (Entries 9 and 10; see for full screening data).

**Figure 2:**
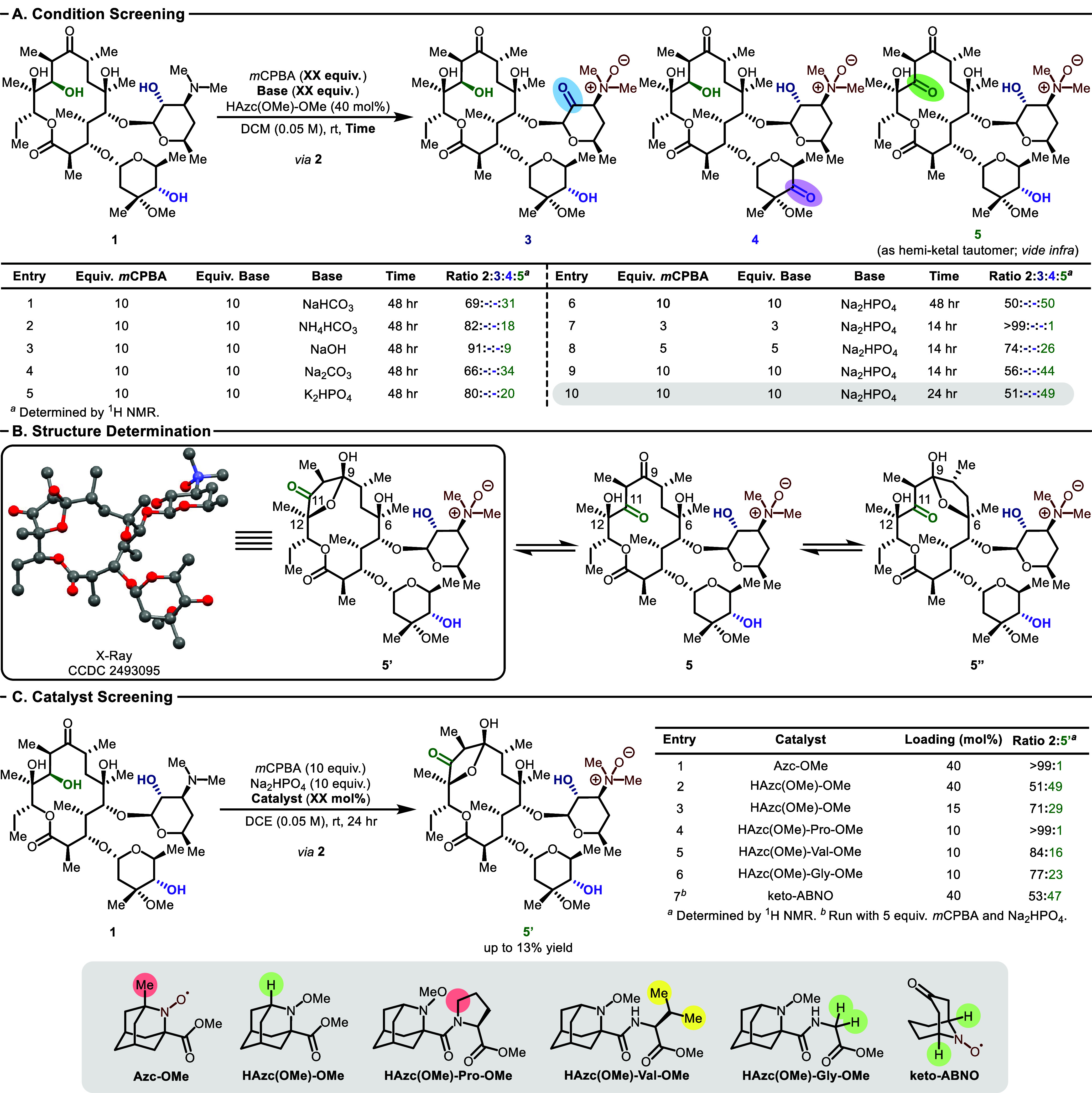
Condition screening, structural determination, and catalyst screening. (A) Condition screening to identify ideal conditions for the formation of oxidized erythromycin *N*-oxide, **5**. “-” used to indicate not observed by UPLC-MS or ^1^H NMR. (B) Structural determination of the predominant isomer of **5**. (C) Steric effects of selected catalysts.

Efforts were then undertaken to determine the site of oxidation. The hydroxyl group on the cladinose sugar (purple) was excluded on the basis of mass spectrometry fragmentation, and two-dimensional NMR studies indicated that the desosamine hydroxyl group (blue) also remained unaltered. However, based on ^13^C NMR analysis, two carbonyls were not present on the macrolide core.^33^ It has been well documented that disruption of the hydrogen bond between the C11 hydroxyl group and the native C9 ketone shifts the equilibrium toward the hemiketal isomers, which indicated a possible explanation of what was observed via NMR.^27,34^ ^13^C NMR shifts were calculated and gave strong support to the dominant species in solution being either of the two possible hemiketal isomers (C9,C12-hemiketal **5′** versus C6,C9-hemiketal **5′′**; see for details).^35^ Crystallization efforts proved fruitful, and the solid state structure was confirmed to be **5′** via single crystal X-ray crystallography. While definitive corroboration that the same hemiketal is dominant in solution has been elusive, it is certainly plausible since the equilibration between **5′** and **5′′** only shows one isomer by ^1^H and ^13^C NMR (Figure 2B).

It is interesting to note that modifications to **HAzc­(OMe)-OMe** revealed a very strong dependence on steric effects (Figure 2C, Entries 1–7). Most notably, with **1**, all modifications tested led to the formation of the same major product, **5′**. The reactions with the aminoxyl catalysts are also pleasingly clean. Isolations of pure products were achieved using preparative reversed phase column chromatography, which resulted in loss of material. Even so, excellent reproducibility was achieved such that reactions that reached a ratio of approximately 50:50 **2** to **5′** could deliver product in 13% isolated yield as analytically pure material in one step from commercially available erythromycin A, with both the *N*-oxidation and C11-OH oxidation occurring in a single operational step.

### Site-Selective Oxidation of Clarithromycin and Azithromycin

A striking divergence was observed when we applied the findings above to different macrolides. For example, when we applied our optimized conditions to clarithromycin, **6** — differing only by methylation of the C6 hydroxyl group — we observed a total divergence in reactivity (Figure 3A). Upon exposure to catalytic **HAzc­(OMe)-OMe** and *m*CPBA, only amine *N*-oxidation to generate product **7** was observed with no detectable alcohol oxidation at any site. Yet, with only *m*CPBA to generate **7** followed by DMP, which rapidly consumes erythromycin A to yield a complex mixture of overoxidation products (*vide supra*), we now observed a highly site-selective monooxidation. To our surprise, this site of oxidation was no longer at C11 on the macrolide (green) but instead at the C4′′ hydroxyl group on the cladinose sugar (purple) to give C4′′-keto-clarithromycin *N*-oxide, **8**.

**Figure 3:**
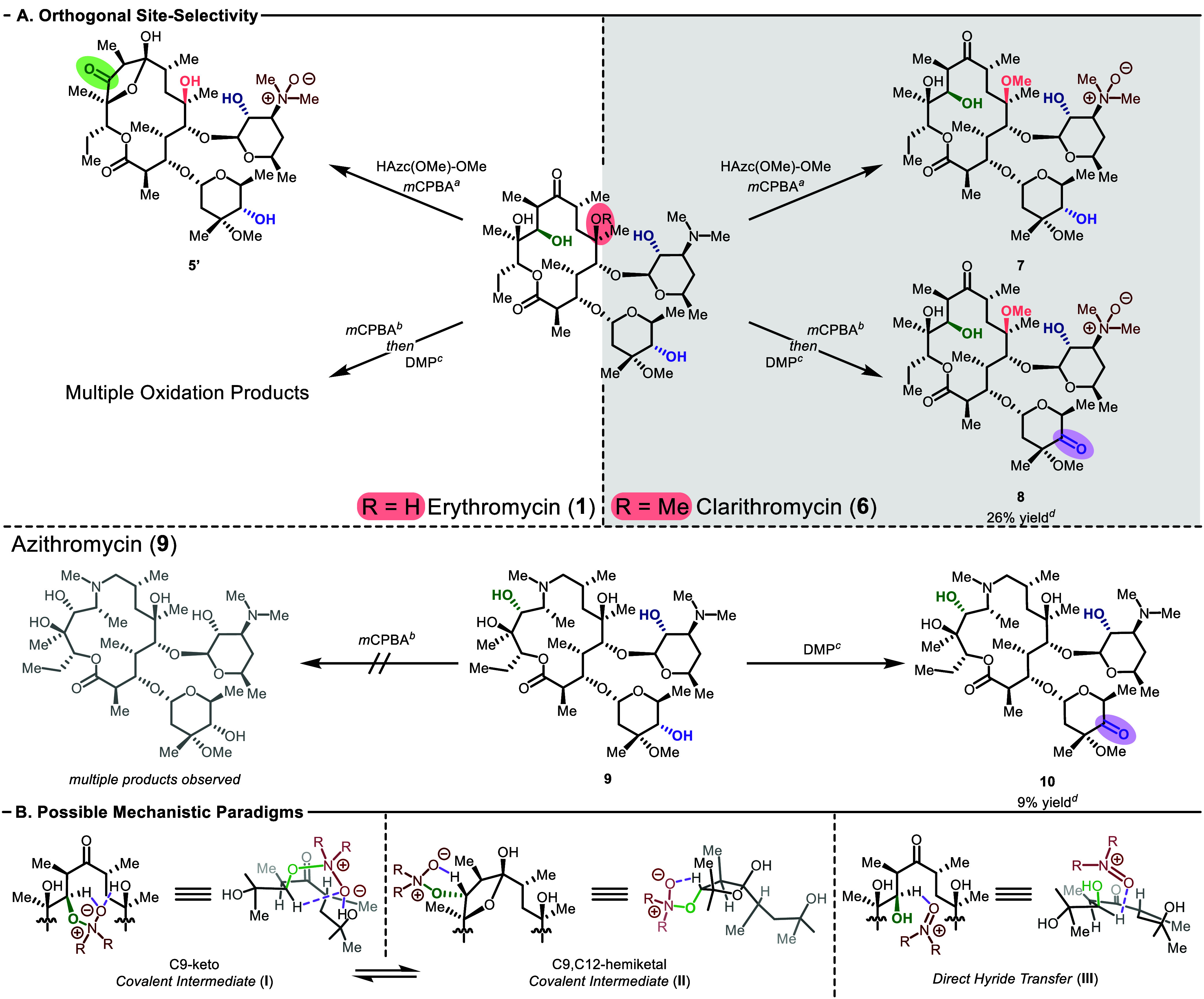
Orthogonal reactivity of macrolides and possible mechanistic paradigms. (A) Orthogonal selectivity between erythromycin A (**1**), clarithromycin (**6**), and azithromycin (**9**). ^*a*^HAzc­(OMe)-OMe (40 mol%), *m*CPBA (10 equiv.), Na_2_HPO_4_ (10 equiv.), DCE (0.05 M), rt, 24 hr. ^*b*^ *m*CPBA (2.0 equiv.), DCM (0.05 M), rt, 30 min. ^*c*^DMP (4.0 equiv.), DCM (0.05 M), rt, 24 hr. ^*d*^Analytical purity was prioritized during demanding purifications leading to lower isolated yields. (B) Proposed possible mechanistic paradigms for the oxidation of erythromycin A.

With azithromycin, **9**, yet another divergent outcome was observed. In this case, the **HAzc­(OMe)-OMe** oxidation protocol led only to degradation, as did exposure to *m*CPBA alone (Figure 3A). However, DMP-mediated oxidation led to clean conversion to the C4′′-keto-azithromycin, **10**, complementary to the formation of **8**.^15^ It is therefore notable that site-selective oxidations of three superficially related macrolides required the development of three distinct protocols yielding three distinct outcomes, highlighting the complexity of site-selective oxidations.

### Discussion of Selectivity

It is interesting to note that the erythromycin hydroxyl group reactivity hierarchy previously observed for group transfer (acylation, P­(III)-transfer) chemistry proved unrelated to the corresponding hydroxyl group oxidation rates.^26–28^ That is, whereas site-selective acylation and P­(III) transfer with erythromycin reveals an intrinsic preference for the C2′ hydroxyl group (desosamine, blue), followed by the C4′′ hydroxyl group (cladinose, purple), before acylation of the C11 macrolide hydroxyl group (green), the oxidation results presented above reveal an entirely distinct set of reactivity preferences for oxidation under various conditions (*vide supra*). In the case of **HAzc­(OMe)-OMe**-catalyzed oxidations, we envision that multiple factors could contribute to the observed preference for C11 oxidation. Two alternative mechanistic paradigms are often considered for alcohol oxidation with oxoammonium catalysts: one invokes a covalent intermediate formed by attack of the hydroxyl group at the oxoammonium nitrogen followed by “Cope-type” elimination to release a carbonyl, which is generally accepted as the operable mechanism under basic conditions (**I** or **II**, Figure 3B);^36,37^ alternatively, direct hydride transfer to the oxoammonium under acidic conditions has been proposed (**III**, Figure 3B).^38^ The first mechanism might show a stronger dependence on steric accessibility as the tetrahedral intermediate preceding Cope elimination is sterically demanding. We hypothesize that the latter mechanism might be more sensitive to hydricity. The former mechanism might be better supported by the differential reactivity of erythromycin A versus clarithromycin and the steric effects observed in catalyst design. Perhaps the clarithromycin C6-OMe creates too much steric congestion for the tetrahedral intermediate; the erythromycin C6-OH could also stabilize the tetrahedral intermediate (or related transition states) through a key H-bond (**I**). Related, it is also possible that with erythromycin, the **HAzc­(OMe)-OMe** derived oxoammonium reacts uniquely with the C9,C12-hemiketal tautomer, which is essentially inaccessible with clarithromycin (**II**).^34,39,40^ Further experimentation showed evidence that pathway **II** is a feasible contributor (see for details). While full elucidation of the mechanistic factors governing site-selectivity is beyond the scope of this report, the interplay of factors that allows for a full reversal of selectivity with only methylation of a single hydroxyl group (erythromycin A vs clarithromycin) is complex and representative of the challenges in developing site-selective transformations.

### Analogs of Oxidized Macrolides

Taking advantage of the chemical versatility of carbonyl groups, analog **5′** was found to be a platform for the synthesis of several new analogs of erythromycin A. Reduction of the amine *N*-oxide could be achieved using Raney Ni to afford carbonyl derivative **11** (Figure 4A).^41^ With regards to controlled further oxidation, we found that, while DMP-mediated oxidation of erythromycin *N*-oxide, **2**, led to a mixture of products (Table 1), further oxidation of isolated **5′** with DMP provided clean access to the double oxidation product **12**, which could also be readily deprotected using the Raney Ni procedure to give **13**.

**Figure 4:**
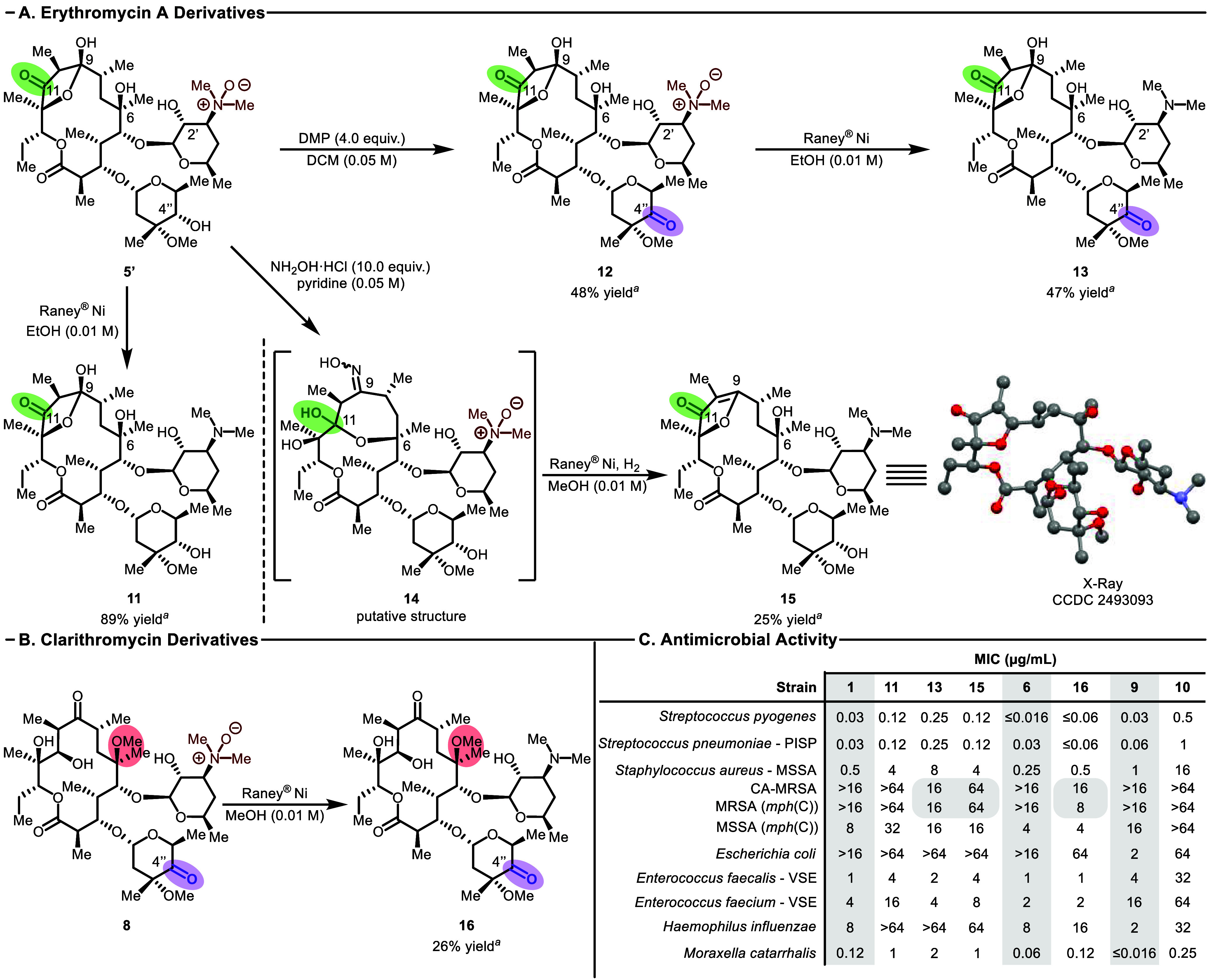
Macrolide analogs synthesized leveraging this methodology and antimicrobial activity. (A) Derivatization of **5′** to generate erythromycin A analogs. (B) Derivatization of **8** to generate a clarithromycin analog. ^*a*^Analytical purity was prioritized during demanding purifications leading to lower isolated yields. (C) Selected MIC (μg/mL) values for analogs.

The putative C9 oxime **14** is prepared following condensation of **5′** with hydroxylamine before being moved into the subsequent reduction with Raney Ni under an atmosphere of H_2_ to cleanly provide **15** via elimination to access the dialkyl furanone erythromycin derivative, as confirmed by X-ray crystallography. We found that this product could also be accessed directly from **11** via acid-catalyzed rearrangement, which led to a mixture of products including **15**.

Additionally, C4′′-keto-clarithromycin **16** was also accessible from **8** using the Raney Ni deprotection procedure (Figure 4B).

### Antimicrobial Activity

Analogs **2**, **5′**, **7**, **8**, **10**, **11**, **12**, **13**, **14**, **15**, and **16** were also tested for antimicrobial activity against a panel of bacterial strains including *Streptococcus pyogenes*, *Streptococcus pneumoniae*, *Staphylococcus aureus*, *Escherichia coli*, *Enterococcus faecalis*, *Enterococcus faecium*, *Haemophilus influenzae*, and *Moraxella* *catarrhalis* (Figure 4C). As expected, it was found that analogs containing the *N*-oxide (**2**, **5′**, **7**, **8**, **12**, and **14**) showed little to no bioactivity, consistent with the known ribosomal binding model that is anchored by the C3′ amine (see for full bioactivity results).^42–44^ Monooxidation erythromycin A analog **11**, however, maintained bioactivity across almost all strains tested that were susceptible to **1**, **6**, and **9**, albeit with lower potency. The retention of bioactivity is notable given that there is evidence that the C9-keto isomer (versus the hemiketal structure) binds preferentially to the ribosome.^45^ The activity of **11** thus may imply either a binding-induced isomerization of **11** to the ketone, or that the hemiketal form can still bind to the ribosome, albeit perhaps to a lesser extent. Interestingly, the derivative **15** also maintained good activity and bacterial strain coverage despite its confinement to the 5-membered ring. This is especially exciting given that this has been shown to be one of the acid-catalyzed degradation products of **11**, meaning that the acid-catalyzed loss of activity that plagues erythromycin A is perhaps no longer a liability with our derivative. Also of note is the observation that C4′′-keto analogs **13** and **16** showed growth inhibition for *S. aureus* strains with *mph*(C) resistance, as well in community-associated methicillin-resistant *S. aureus* (CA-MRSA); in contrast, the parent compounds **1**, **6**, and **9** showed no such activity at the concentrations tested. The *mph*(C) resistance mechanism is reported to proceed through C2′ hydroxyl group phosphorylation, which disrupts ribosomal binding.^46–48^ It is unclear why oxidation at C4′′ (purple) for erythromycin and clarithromycin would impact this resistance mechanism, nor why C4′′ oxidation of azithromycin, **10**, fails to provide the analogous activity with the resistant strains. Finally, the activity profile for C4′′-keto-clarithromycin, **16**, shows the broadest spectrum of activities, with several enhanced activities relative to its parent, **6**.

## Conclusion

We have shown site-selective oxidations of erythromycin A, clarithromycin, and azithromycin displaying apparent differential catalyst-substrate and reagent-substrate interactions. Selectivity and reactivity were facilitated by the design and synthesis of the aminoxyl core **HAzc­(OMe)-OMe**, which is related to prior disclosures but implemented here in a precatalyst form.^49,50^ Orthogonal conditions were then required to access each macrolide analog, and each newly installed carbonyl presents access to a wide variety of chemical space to be explored.

Furthermore, we have demonstrated the feasibility and utility of site-selective oxidations in the synthesis of molecules with enhanced antimicrobial activity. Installation of nominally minor structural changes accessed a suite of analogs with broad spectrum antibiotic activity, including three that showed inhibition of strains that are resistant to erythromycin A, clarithromycin, and azithromycin, highlighting the utility of site-selective functionalization campaigns.

## Supplementary Material













## Data Availability

The document for this work is available free of charge online. Copies of experimental data, including NMR FID signals and coordinates for structures obtained in the computational work, can be found in the FAIR data file.

## Notes

Detailed experimental procedures and analytical data; X-ray crystallographic data for **5′**, **7**, and **15**; and NMR spectra (PDF)
FAIR data files including NMR.fid, HRMS, IR, .cif, and .xyz files (ZIP-1, ZIP-2, ZIP-3, ZIP-4, ZIP-5)

## Notes

Accession Codes Deposition Numbers 2493095, 2493094, and 2493093 contain the supplementary crystallographic data for this paper. These data can be obtained free of charge via the joint Cambridge Crystallographic Data Centre (CCDC) and Fachinformationszentrum Karlsruhe Access Structures service.
