# Supplementary material for: Site-Divergent Oxidations within Venerable Macrolide Antibiotic Scaffolds Unveil Compounds with Broad Spectrum and Anti-MRSA Activities
Source: ACS Cent Sci. 2026 Mar 17;12(3):375–82. doi: 10.1021/acscentsci.5c02343 (PMC13022725; doi:10.1021/acscentsci.5c02343)
Supplement: Supplementary file 1 [file oc5c02343_si_001.pdf]

Supporting Information

Site-Divergent Oxidations within Venerable Macrolide Antibiotic Scaffolds Unveil Compounds with Broad Spectrum and *anti*-MRSA Activities

Olivia C. Langner, Brandon Q. Mercado, Sebastian M. Krajewski, Song Lin, and Scott J. Miller*

Department of Chemistry, Yale University, New Haven, Connecticut 06520 – 8170, United States

Department of Chemistry and Chemical Biology, Cornell University, Ithaca, New York 14853, United States.

Correspondence: Scott J. Miller scott.miller@yale.edu

Table of Contents

1. General Information 3

2. Catalyst Synthesis 6

2.1. HAzc(OMe)-OMe Synthesis *6*
2.2. Other Aminoxyl/Oxoammonium Catalysts *12*
2.3. Peptide Catalysts *13*
2.3.1. Representative Synthetic Procedure for HAzc(OMe) Peptides 13
2.3.2. Characterization of HAzc(OMe) Catalysts 15

3. Synthesis of Erythromycin Derivatives 19

4. Synthesis of Clarithromycin Derivatives 38

5. Synthesis of Azithromycin Analog 47

6. Reaction Ratio Determination 51

7. Reaction Optimization 52

8. Mechanism Probes 59

8.1 Acid-Catalyzed Degradation of 11 .... *59*
8.2 Oxidation of Erythromycin A Spiroketal *65*

9. ^{13}C NMR Calculations 72

9.1 Computational Methodologies *72*
9.2 Computational Results *73*

10. Biological Assays 78

10.1 Materials and Methods for Round 1 of Testing *78*
10.2 Activity Results for Round 1 of Testing *81*
10.3 Materials and Methods for Round 2 of Testing *82*
10.4 Activity Results for Round 2 of Testing *85*

11. NMR Spectra 87

12. X-Ray Structures 132

13. References 141

1. General Information

Room temperature is defined as 19–22 °C. All other commercially available reagents were purchased from common suppliers and used without further purification unless otherwise noted. *m*CPBA was assumed to be 72% active peroxide. ^{t}BuOCl was synthesized according to a literature procedure.[1] Deionized water was used for reactions, extractions, and quenching solutions. All other solvents were purchased from commercial suppliers and used without further purification unless otherwise noted. No unexpected or unusually high safety hazards were encountered.

Analytical Methods: *Thin-layer chromatography:* Analytical thin-layer chromatography (TLC) was performed using EMD Millipore silica gel 60 F254 precoated plates (0.25 mm thickness) and developed plates were visualized under a UV lamp and stained with $KMnO_4$. R_f values are reported.

Column chromatography: Normal phase flash column chromatography was conducted on an automated Biotage® IsoleraTM One purification system equipped with a 10, 25, 50, or 100 g SNAP Ultra (HP Sphere, 25 µm silica) cartridge or using 60 Å Silica Gel (32–62 micron) with an appropriate mobile phase composition and gradient. Reversed phase flash column chromatography was performed using an automated Biotage® IsoleraTM One purification system equipped with a 12, 30, 60, or 120 g SNAP Ultra C18 cartridge. The desired fractions were analyzed by TLC or UPLC/MS. Preparatory high-performance liquid chromatography (HPLC) was performed on Shimadzu Prominence HPLC system equipped with a photodiode array detector (210 nm) and a Luna® 5 µm C18(2) 100 Å column with a flow rate of 15 mL/min. Column temperature was unregulated.

NMR: ^{1}H NMR spectra were recorded on Agilent 400, 500, 600, or 800 MHz spectrometers at ambient temperature unless otherwise stated. All NMR solvents were purchased from Cambridge Isotope Laboratories and used without further purification. Deuterated solvents were stored at ambient temperature and were used immediately after opening. Spectra were processed using MestReNova 14.2.0 using the automatic phasing and polynomial baseline correction capabilities. Additional manual phasing was done as necessary. Splitting was determined using the automatic multiplet analysis function with manual intervention as necessary. Spectral data are reported as follows: chemical shift (multiplicity [singlet (s), broad singlet (brs), doublet (d), triplet (t), quartet (q), pentet (p), multiplet (m), doublet of doublets (dd), doublet of doublet of doublets (ddd), doublet of triplet of doublets (dtd), doublet of doublet of doublet of doublets (dddd), doublet of triplets (dt), triplet of doublets (td), etc.], coupling constant, integration). Chemical shifts are reported in ppm (δ), and coupling constants are reported in Hz. ^{1}H Resonances are referenced to solvent residual peaks for $CDCl_3$ (7.26 ppm), MeOD (3.31 ppm), or DMSO-d_6 (2.50 ppm).[2] ^{13}C NMR spectra were recorded on Agilent 400, 500, 600, or 800 MHz spectrometers with protons fully decoupled unless otherwise stated. ^{13}C resonances are reported in ppm relative to solvent residual peaks for $CDCl_3$ (77.2 ppm) or DMSO-d_6 (39.5).[2] Note: Small deviations in chemical shifts may be observed depending on the concentration of NMR samples, and exchangeable protons are often not observed.

Fourier-Transform (FT) infrared (IR) spectroscopy: Measurements were recorded on a Shimadzu IRTracer-100 spectrometer equipped with a diamond ATR unit, and select νmax are reported in cm^{-1}. Data was recorded in the range from 400 to 4000 cm^{-1} with a resolution of 2 cm^{-1}. At least 16 spectra were averaged, and air was subtracted as background. Data acquisition and post-

processing were performed with LabSolutions IR Version 2.23. Only relevant peaks are reported here. Please see the fair data for full spectra.
Optical rotation: Optical rotations were recorded on an Autopol VI Automatic Polarimeter at the sodium D-line (589 nm), using a Type 40T TempTrolTM cell of 0.50 dm path length at 20 °C and reported as follows: $[\alpha]^{temp}_{\lambda}$, concentration (c, in g/100 mL), and solvent.
Mass spectrometry: Ultra high-performance liquid chromatography/mass spectrometry (UPLC/MS) and low-resolution mass spectrometry (LRMS) were performed on a Waters Acquity SQD2 instrument equipped with an Ultra BEH C18 column (1.7 µm, 2.1 x 50 mm), a dual atmospheric pressure chemical ionization (API)/electrospray ionization (ESI) mass spectrometry detector and a photodiode array detector. High-resolution mass spectrometry (HRMS) was conducted by the Chemical and Biophysical Instrumentation Center (CBIC) at Yale University and was performed on a Waters Xevo Q-TOF high-resolution mass spectrometer using ESI.
X-Ray Crystallography: X-ray diffraction was conducted by the CBIC at Yale University.

Abbreviations:

ACT	4-Acetamido-2,2,6,6-tetramethylpiperidine 1-Oxyl
API	Atmospheric Pressure Chemical Ionization
aq	Aqueous
Boc	*tert*-Butoxycarbonyl
CAN	Ceric Ammonium Nitrate
CBIC	Chemical and Biophysical Instrumentation Center
Cbz	Benzyloxycarbonyl
DCM	Dichloromethane
DIPEA	N,N-Diisopropylethylamine
DMF	Dimethylformamide
DMP	Dess–Martin Periodinane (1,1,1-Tris(acetyloxy)-1,1-dihydro-1,2-benziodoxol-3-(1*H*)-one)
DMS	Dimethyl Sulfide
DMSO	Dimethyl Sulfoxide
EtOAc	Ethyl Acetate
EtOH	Ethanol
ESI	Electrospray Ionization
FTIR	Fourier-Transform Infrared Spectroscopy
HATU	O-(7-Azabenzotriazol-1-yl)-N,N,N',N'-Tetramethyluronium Hexafluorophosphate
Hex	Hexanes
HPLC	High-Performance Liquid Chromatography
IPA	Isopropyl Alcohol
LRMS	Low-Resolution Mass Spectrometry
M	Molar (i.e. mol/L)
*m*CBA	*meta*-Chlorobenzoic Acid
*m*CPBA	*meta*-Chloroperoxybenzoic Acid
MeCN	Acetonitrile
MeOH	Methanol
MMPP	Magnesium Monoperoxyphthalate
MTBE	Methyl *tert*-Butyl Ether
NMR	Nuclear Magnetic Resonance

PIDA	(Diacetoxyiodo)benzene
PIFA	(Bis(trifluoroacetoxy)iodo)benzene
Piv	2,2-Dimethylpropanoyl
RMS	Root Mean Square
TBAOH	Tetrabutylammonium Hydroxide
TCCA	Trichloroisocyanuric Acid
THF	Tetrahydrofuran
TLC	Thin-Layer Chromatography
Ts	Tosyl/*p*-Toluenesulfonyl
UHP	Hydrogen Peroxide–Urea
UPLC/MS	Ultra High-Performance Liquid Chromatography/Mass Spectrometry
UV	Ultra-violet

2. Catalyst Synthesis

2.1. HAzc(OMe)-OMe Synthesis

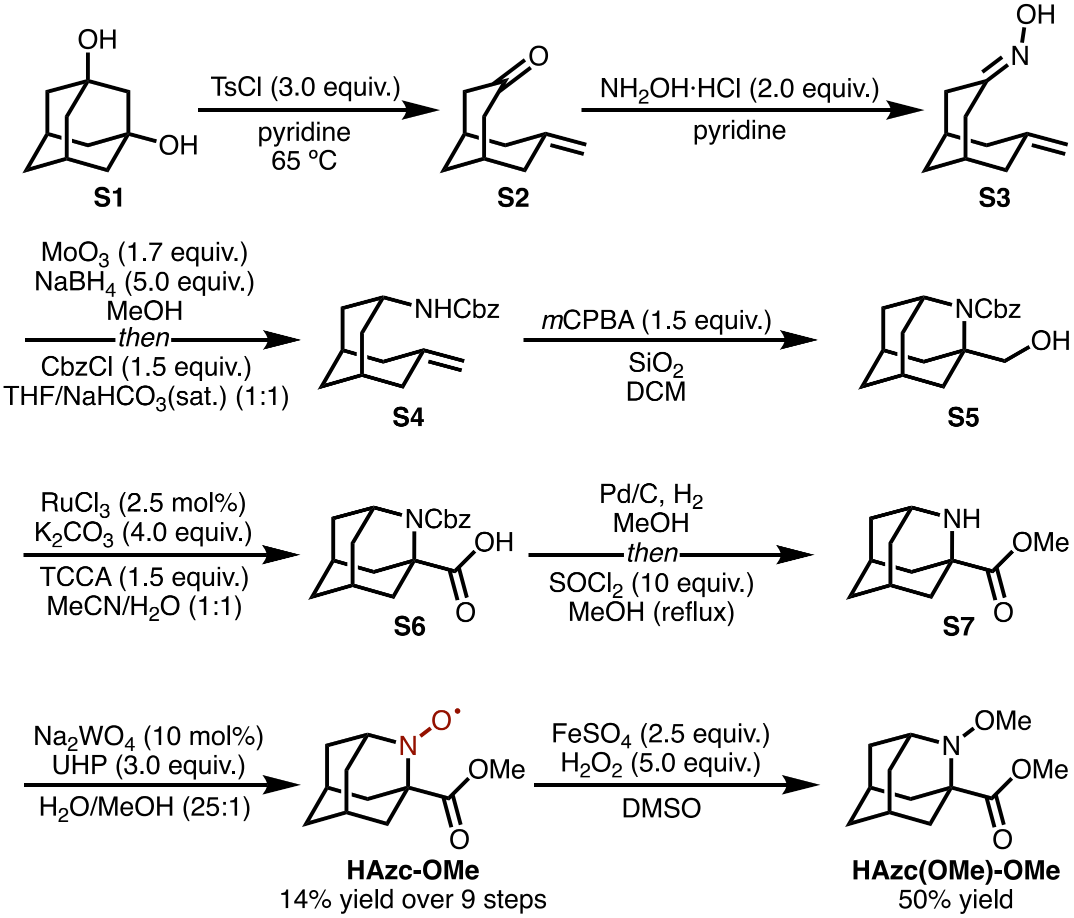

Scheme S1. Synthesis of **HAzc(OMe)-OMe.**

The synthesis of **HAzc(OMe)-OMe** is performed without column chromatography purification until the generation of **HAzc-OMe**. Crude ^{1}H have been included in Section 11 to aid in replication of these procedures.

7-Methylenebicyclo[3.3.1]nonan-3-one (**S2**)

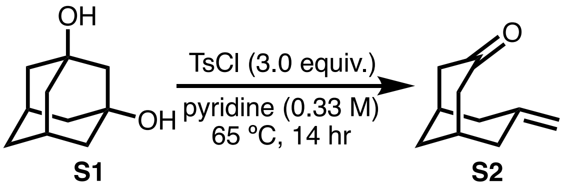

A 1 L round bottom flask equipped with a stir bar was charged with adamantane diol **S1** (16.8 g, 100 mmol, 1.00 equiv) and tosyl chloride (57.2 g, 300 mmol, 3.00 equiv.). Pyridine (300 mL) was then added. The resulting solution was immersed in an oil bath preheated to 65 ºC and stirred overnight. During this time, the pale-yellow solution darkened to a clear orange. After completion

of the reaction as determined by TLC, the hot solution was poured over ~300 g of ice. After the ice was fully melted, the mixture was extracted with Hex:EtOAc (1:1, 150 mL) and filtered through a plug of Celite®. This solution was then concentrated, and ketone was **S2** moved forward crude without further purification.

7-Methylenebicyclo[3.3.1]nonan-3-one Oxime (**S3**)

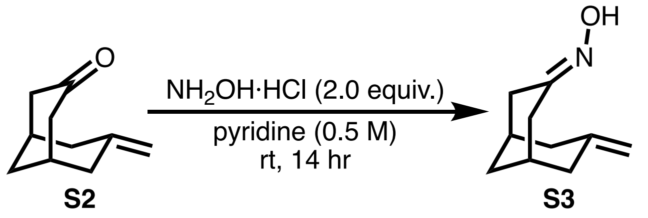

A 500 mL round bottom flask equipped with a stir bar was charged with ketone **S2** (assumed 15.0 g, 100 mmol, 1.00 equiv) and hydroxylamine hydrochloride (13.9 g, 200 mmol, 2.00 equiv.). Pyridine (200 mL) was then added. The resulting solution was stirred at room temperature overnight before being concentrated to ~15% of the initial volume then was brought up in H_2O and extracted with EtOAc. The combined organic layers were dried over Na_2SO_4 and concentrated before repeated evaporations with toluene *in vacuo* until no pyridine was detectable by ^{1}H NMR. The resulting pale-yellow solid **S3** was moved forward crude without further purification. (~91 % yield over 2 steps by ^{1}H NMR; see Section 11 for representative crude ^{1}H NMR).[3]

Benzyl (7-Methylenebicyclo[3.3.1]nonan-3-yl)carbamate (**S4**)

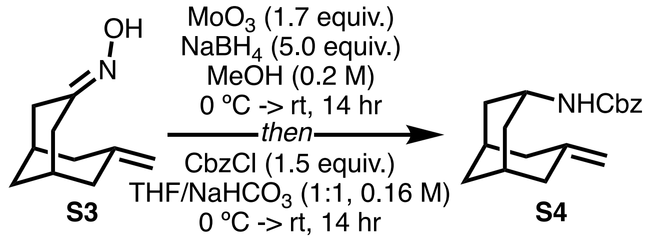

A 1 L round bottom flask equipped with a stir bar was charged with crude oxime **S3** (15.5 g, 93.8 mmol, 1.00 equiv) and molybdenum trioxide (23.0 g, 159.5 mmol, 1.70 equiv.). Methanol (470 mL) was then added. The resulting mixture was immersed in an ice bath followed by the portion-wise addition of $NaBH_4$ (17.7 g, 469 mmol, 5.00 equiv.). (N.B. Slow addition is crucial as the molybdenum leads to increased foaming over the course of the reaction.) After addition was completed, the reaction was allowed to slowly warm to room temperature and stir overnight. The mixture was then filtered over Celite® and concentrated before being moved forward without further purification.

A 1 L round bottom flask equipped with a stir bar was charged with the resulting solid from the previous step (assumed 93.8 mmol, 1.00 equiv). THF/$NaHCO_{3(aq)}$ (saturated) (1:1, 570 mL) was then added to the flask. The resulting mixture was immersed in an ice bath followed by dropwise addition of CbzCl (20 mL, 140.7 mmol, 1.50 equiv.). Following complete addition, the reaction was allowed to slowly warm to room temperature and was stirred overnight. The mixture was then

filtered over Celite®, and the filtrate extracted with EtOAc before being dried over Na_2SO_4 and concentrated. The pale-yellow solid was brought up in EtOH (100 mL) before dropwise addition to a 4 L Erlenmeyer containing 1.5 L of H_2O with vigorous stirring. Following complete addition, the resulting suspension was filtered. The addition into water and filtration was repeated until the amount of benzyl alcohol was <5 % by ^{1}H NMR. The product **S4** was collected as a pale yellow solid and moved forward crude without further purification (~68 % yield by ^{1}H NMR; see Section 11 for representative crude ^{1}H NMR).[4]

Benzyl 1-(Hydroxymethyl)-2-azaadamantane-2-carboxylate (**S5**)

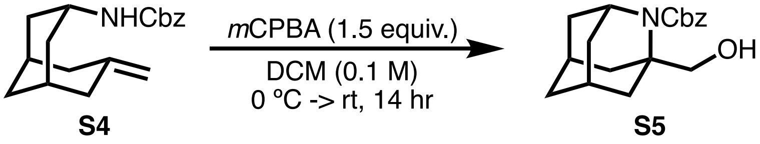

A 1 L round bottom flask equipped with a stir bar was charged with crude olefin **S4** (18.3 g, 64.1 mmol, 1.00 equiv). DCM (640 mL) was then added. The resulting mixture was immersed in an ice bath followed by portion-wise addition of *m*CPBA (23.0 g, 96.2 mmol, 1.50 equiv.). The reaction mixture was vigorously stirred and was allowed to gradually warm to room temperature. The solution was stirred at this temperature for 4 hours before the addition of SiO_2 (~100 g) and additional stirring for 1.5 days. (N.B. The SiO_2 catalyzes the ring opening via nucleophilic attack by the N rather than acidic protonation or hydride shift.) The slurry was filtered and extracted with EtOAc and DCM. The organic solution was then washed $NaHSO_{3(aq)}$ (saturated, 1x) and NaOH (2 M, 3x) before being dried over Na_2SO_4 and concentrated to afford the pale-yellow solid **S5**, which was carried forward crude without further purification. (~75 % yield by NMR; see Section 11 for representative crude ^{1}H NMR).

2-((Benzyloxy)carbonyl)-2-azaadamantane-1-carboxylic Acid (**S6**)

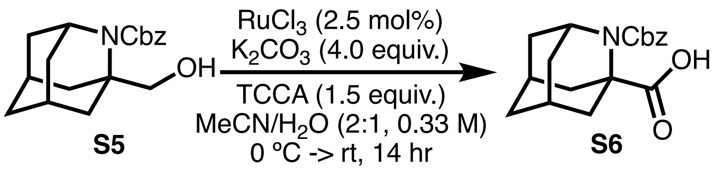

A 500 mL round bottom flask equipped with a stir bar was charged with crude alcohol **S5** (17.6 g, 58.4 mmol, 1.00 equiv.), $RuCl_3$ (329.1 mg, 1.46 mmol, 2.5 mol%), and K_2CO_3 (32.3 g, 233.6 mmol, 4.00 equiv.). MeCN/H_2O (2:1, 174 mL) was added. The resulting suspension was immersed in an ice bath and subsequently a solution of TCCA (0.5 M in MeCN, 20.3 g, 87.6 mmol, 1.50 equiv.) was added dropwise. The reaction mixture was vigorously stirred and allowed to gradually warm to room temperature overnight. Then, ~10 mL of IPA was added before the reaction was stirred for an additional hour. The solution mixture was then filtered through a pad of Celite® and flushed through with $NaHCO_{3(aq)}$ (saturated). The combined solution was acidified with $HCl_{(aq)}$ (1 M) to a pH of ~1 and concentrated. Carboxylic acid **S6** was moved forward without further purification (~60 % yield by ^{1}H NMR; see Section 11 for representative crude ^{1}H NMR).[5]

Methyl 2-Azaadamantane-1-carboxylate (**S7**)

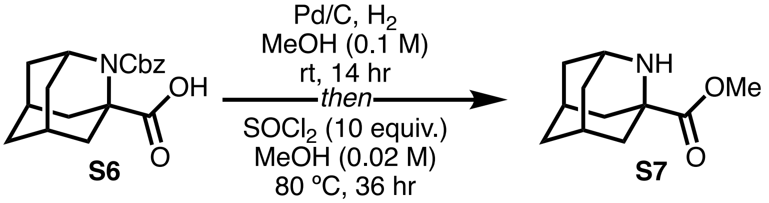

A 500 mL round bottom flask equipped with a stir bar was charged with acid **S6** (3.50 g, 11.0 mmol, 1.00 equiv.). The flask was evacuated and backfilled with N_2 and MeOH (110 mL) was added. The resulting solution was sparged with N_2 prior to the addition of Pd/C (10% on activated carbon, 50% wet with water, 350 mg, 10% by weight) followed by additional sparging with N_2. The suspension was then sparged with H_2 and stirred under a balloon of H_2 overnight. Following this, the balloon was removed, and the reaction was stirred open to air before being filtered over Celite® and washed with MeOH (375 mL total, 2x) into a 1 L round bottom flask equipped with a stir bar. $SOCl_2$ (8.00 mL, 110 mmol, 10 equiv.) was then added slowly to the vigorously stirring solution. After complete addition, the reaction was heated to reflux and stirred at this temperature for 1.5 days before it was cooled to room temperature and evaporated by a stream of N_2 to approximately 20% of the original volume. The solution was then slowly added to $NaHCO_{3(aq)}$ (saturated, 400 mL), filtered over Celite®, and extracted with EtOAc. The combined organic layers were dried over Na_2SO_4 before being concentrated and moved forward without further purification as an off-white solid (**S7**) (~90 % yield by ^1H NMR; see Section 11 for representative crude ^1H NMR).

Methyl 2-Azaadamantane-*N*-oxyl-1-carboxylate (**HAzc-OMe**)

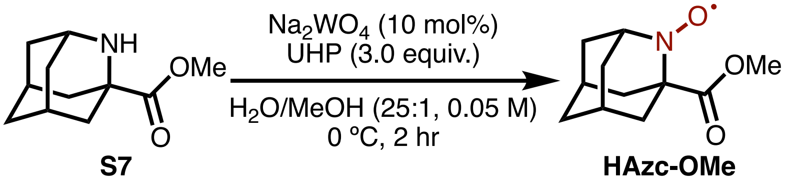

Adapted from literature precedent,[6] a 25 mL round bottom flask equipped with a stir bar was charged with **S7** (250 mg, 1.30 mmol, 1.00 equiv) and $Na_2WO_4 \cdot 2H_2O$ (42.9 mg, 0.13 mmol, 10.0 mol%). MeOH (0.09 mL) and H_2O (2.34 mL) were added. The resulting suspension was immersed in an ice bath followed by the dropwise addition of UHP (362 mg, 3.85 mmol, 3.00 equiv.) in water (2.12 M in H_2O, 1.82 mL) over 1.5 hours. The reaction mixture was vigorously stirred at 0 ºC for 5 hours before being transferred to a -20 ºC freezer for 4 days. The frozen red reaction was thawed and extracted with DCM until the DCM was no longer pink (~30 mL, 4x). The combined organic layers were dried over Na_2SO_4 and concentrated via rotary evaporation with the bath kept cold by addition of ice. (N.B. Heating or excess time spent in organic solution will lead to degradation of the aminoxyl.) The crude residue was then purified via SiO_2 column chromatography (40% EtOAc/Hex). **HAzc-OMe** was collected as a red crystalline solid (156 mg, 0.74 mmol, 57% yield).[4]

As part of a separate project, an X-ray structure for **HAzc-OMe** was obtained in house (CCDC 2366598).[7]

R_f: 0.37 (20% EtOAc/Hex, visualized with $KMnO_4$ stain)

NMR: Paramagnetism precludes NMR characterization.

FTIR (solid) cm^{-1}: 2944, 2927, 2899, 1735, 1450, 1437, 1377.

HRMS (ESI+) calculated for $C_{11}H_{17}NO_3^{\bullet+}$ $[M+H]^{\bullet+}$ 211.1203, found 211.1198.

Methyl 2-Azaadamantane-*N*-methoxy-1-carboxylate (**HAzc(OMe)-OMe**)

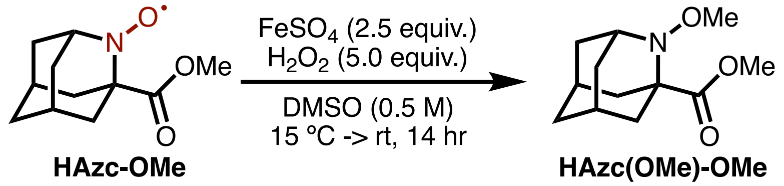

Based on literature precedent,[8] a 10 mL round bottom flask equipped with a stir bar was charged with **HAzc-OMe** (105 mg, 0.50 mmol, 1.00 equiv) and $FeSO_4 \cdot 7H_2O$ (348 mg, 1.25 mmol, 2.50 equiv.). DMSO (2.5 mL) was added. The resulting suspension was immersed in a chilled water bath followed by the dropwise addition of H_2O_2 (30% w/w in H_2O, 0.255 mL, 2.50 mmol, 5.00 equiv.) over 30 minutes via syringe pump. The reaction mixture was vigorously stirred at room temperature overnight before being diluted with water. The dark orange solution was then extracted with Et_2O (3x). The combined organic layers were dried over Na_2SO_4 and concentrated via rotary evaporation. The crude residue was then purified via SiO_2 column chromatography (20% EtOAc/Hex). **HAzc(OMe)-OMe** was collected as a pale-yellow oil (55.3 mg, 0.25 mmol, 50% yield). (N.B. Slow inversion of the N forms a chiral center stable enough to present diastereotopic signals in ^{1}H and ^{13}C NMR.)

R_f: 0.62 (20% EtOAc/Hex, visualized with $KMnO_4$ stain)

^{1}H NMR (600 MHz, $CDCl_3$) δ 3.76 (s, 3H), 3.52 (s, 3H), 3.47 (s, 1H), 2.42 (dd, *J* = 13.1, 2.7 Hz, 1H), 2.27 (dt, *J* = 12.8, 2.9 Hz, 1H), 2.15 – 2.02 (m, 3H), 1.99 (hept, *J* = 3.4 Hz, 1H), 1.85 (dq, *J* = 12.8, 3.1 Hz, 1H), 1.82 – 1.68 (m, 4H), 1.37 – 1.23 (m, 1H).

^{13}C NMR (151 MHz, $CDCl_3$) δ 174.8, 64.3, 59.5, 53.1, 52.1, 40.0, 35.9, 35.8, 31.0, 29.1, 26.6, 26.5.

FTIR (solid) cm^{-1}: 2910, 2853, 2807, 1734, 1445, 1343, 1331.

HRMS (ESI+) calculated for $C_{12}H_{20}NO_3$ $[M+H]^+$ 226.1438, found 226.1432.

2.2. Other Aminoxyl/Oxoammonium Catalysts

keto-ABNO

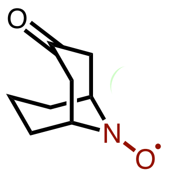

keto-ABNO was prepared according to literature precedent.[6]

NMR: Paramagnetism precludes NMR characterization.

LRMS: (ESI+) calculated for $C_8H_{14}NO_2$ $[M+H]^+$ 156.1019, found 156.24. (N.B. Formic acid buffered solution leads to quenching to the hydroxylamine.)

Azc-OMe

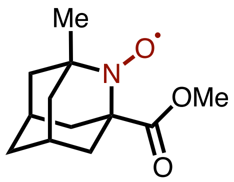

Azc-OMe was prepared according to literature precedent.[3]

NMR: Paramagnetism precludes NMR characterization.

LRMS: (ESI+) calculated for $C_{12}H_{19}NO_3$ $[M+H]^{\bullet+}$ 225.1359, found 225.14.

2.3. Peptide Catalysts

2.3.1. Representative Synthetic Procedure for HAzc(OMe) Peptides

Peptide Coupling Procedure

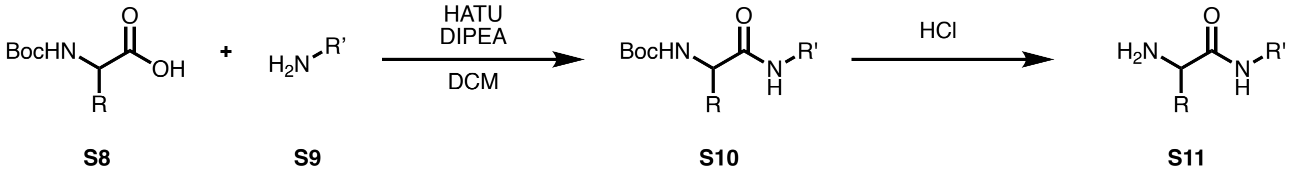

A round bottom flask equipped with a stir bar was charged with carboxylic acid **S8** (1.00 equiv.), amine **S9** (1.10 equiv.), and HATU (1.20 equiv.). DCM (0.33 M) was added followed quickly by DIPEA (2.00 equiv.). The mixture was vigorously stirred until full consumption of **S8** was observed by UPLC/MS. (Typical reactions times range from 2–4 hours with longer required for some sterically hindered coupling partners.) The reaction was then diluted with DCM and washed with $NaHCO_{3(aq)}$ (saturated, 1x), citric acid (10% aq w/v, 2x), and $NaHCO_{3(aq)}$ (saturated, 1x). The organic layer was then dried over Na_2SO_4 and concentrated before being moved into the next step without further purification unless otherwise noted.

Boc Deprotection Procedure

A round bottom flask equipped with a stir bar was charged with the Boc-protected amine **S10** (1.00 equiv.) and HCl (4 M in dioxanes, 6 equiv.). The solution was stirred vigorously until full consumption of the Boc-protected amine was observed by UPLC/MS. (Typical reactions times range from 2–4 hours with longer required for some sterically hindered amines.) The solution was then concentrated to dryness via rotary evaporator and azeotroped twice with DCM. The crude foam **S11** was then carried into the next without further purification.

HAzc(OMe)-OMe Hydrolysis Procedure

A round bottom flask equipped with a stir bar was charged with **HAzc(OMe)-OMe** (1.00 equiv). MeOH (0.20 M) and KOH (4 M in H_2O, 10 equiv.) were added. The reaction was stirred vigorously for 7 days before being acidified with $HCl_{(aq)}$ (1 M) until pH ~2 and extracted with $CHCl_3$:IPA (3:1, 3x). The combined organic layers were dried over Na_2SO_4 and concentrated to provide **HAzc(OMe)-OH** which was moved forward crude without further purification.

HAzc(OMe)-OH Coupling Procedure

A round bottom flask equipped with a stir bar was charged with **HAzc(OMe)-OH** (1.00 equiv.) and HATU (1.20 equiv.). The deprotected amine coupling partner (equivalents vary by reaction) (0.33 M in DMF) and DIPEA (2.00 equiv.) were added. The solution was stirred vigorously until full consumption of **HAzc(OMe)-OH** was observed by UPLC/MS. (Typical reactions times range from 2–4 hours with longer required for some sterically hindered amines.). The reaction was then diluted with DCM and washed with $NaHCO_{3(aq)}$ (saturated, 1x), citric acid (10% aq w/v, 2x), and

$NaHCO_{3(aq)}$ (saturated, 1x). The organic layer was then concentrated then purified via reverse phase column chromatography.

2.3.2. Characterization of HAzc(OMe) Catalysts

HAzc(OMe)-Gly-OMe (**C1**)

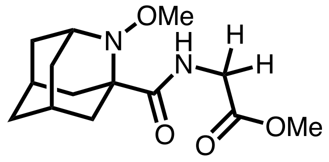

HAzc(OMe)-OMe Hydrolysis Procedure was performed (45.0 mg, 0.20 mmol, 1.00 equiv. **HAzc(OMe-OMe)**) followed by **HAzc(OMe)-OH Coupling Procedure** using H-Gly-OMe•HCl (27.6 mg, 0.22 mmol, 1.10 equiv.). The crude compound was purified by reverse phase column chromatography (Biotage, SNAP Ultra C18 12 g, 0–100% MeCN/H_2O) to provide HAzc(OMe)-Gly-OMe **C1** as a white foam (46.7 mg, 0.165 mmol, 83% yield).

R_f: 0.28 (20% EtOAc/Hex, visualized with $KMnO_4$ stain)

^{1}H NMR (600 MHz, $CDCl_3$) δ 7.14 (t, *J* = 5.3 Hz, 1H), 4.16 – 4.03 (m, 2H), 3.76 (s, 3H), 3.56 – 3.44 (m, 4H), 2.31 – 2.24 (m, 2H), 2.05 (p, *J* = 3.3 Hz, 1H), 1.98 – 1.91 (m, 2H), 1.91 – 1.82 (m, 4H), 1.78 – 1.69 (m, 2H), 1.29 (ddt, *J* = 12.7, 3.0, 1.8 Hz, 1H).

^{13}C NMR (151 MHz, $CDCl_3$) δ 175.5, 170.9, 64.2, 58.9, 52.4, 52.2, 41.9, 41.0, 36.0, 35.9, 29.4, 29.1, 26.8, 26.5.

FTIR (solid) cm^{-1}: 3401, 2931, 2853,1754, 1669, 1511, 1445, 1370.

HRMS (ESI+) calculated for $C_{14}H_{23}N_2O_4$ $[M+H]^+$ 283.1652, found 283.1648.

HAzc(OMe)-Val-OMe (**C2**)

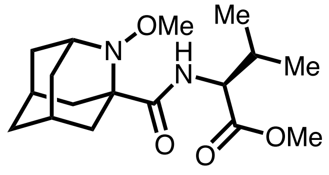

HAzc(OMe)-OMe Hydrolysis Procedure was performed (45.0 mg, 0.20 mmol. 1.00 equiv. **HAzc(OMe-OMe)**) followed by **HAzc(OMe)-OH Coupling Procedure** using H-Val-OMe•HCl (36.9 mg, 0.22 mmol, 1.10 equiv.). The crude compound was purified by reverse phase column chromatography (Biotage, SNAP Ultra C18 12 g, 0–100% MeCN/H_2O) to provide HAzc(OMe)-Val-OMe **C2** as a white foam (63.3 mg, 0.195 mmol, 98% yield). (N.B. Peak doubling due to purported *N*-invertomers at the hydroxylamine nitrogen.)

R_f: 0.46 (20% EtOAc/Hex, visualized with $KMnO_4$ stain)

^{1}H NMR (400 MHz, $CDCl_3$) δ 4.65 – 4.52 (m, 1H), 3.73 (s, 3H), 3.62 – 3.45 (m, 4H), 2.32 – 2.24 (m, 2H), 2.23 – 2.12 (m, 1H), 2.07 – 2.02 (m, 1H), 2.01 – 1.81 (m, 6H), 1.74 (dt, *J* = 3.7, 1.8 Hz, 2H), 1.33 – 1.24 (m, 1H), 1.00 – 0.91 (m, 6H).

^{13}C NMR (101 MHz, $CDCl_3$) δ 173.0, 172.9, 58.8, 58.7, 57.2, 56.5, 52.1, 52.1, 42.4, 41.6, 35.8, 32.1, 31.5, 29.6, 29.5, 29.1, 26.8, 26.7, 26.5, 19.3, 19.1, 18.3, 18.0.

FTIR (solid) cm^{-1}: 3407, 2934, 2853, 1742, 1681, 1500, 1466, 1445.

Optical Rotation: $[\alpha]^{20}_D$ -25.8 (*c* = 0.307, DCM)

HRMS (ESI+) calculated for $C_{17}H_{29}N_2O_4$ $[M+H]^+$ 325.2122, found 325.2114.

HAzc(OMe)-Pro-OMe (**C3**)

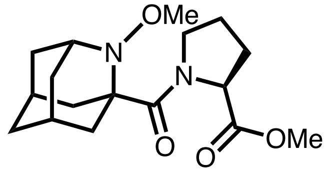

HAzc(OMe)-OMe Hydrolysis Procedure was performed (45.0 mg, 0.20 mmol, 1.00 equiv. **HAzc(OMe-OMe)**) followed by **HAzc(OMe)-OH Coupling Procedure** using H-Pro-OMe•HCl (36.4 mg, 0.22 mmol, 1.10 equiv.). The crude compound was purified by reverse phase column chromatography (Biotage, SNAP Ultra C18 12 g, 0–100% MeCN/H_2O) to provide HAzc(OMe)-Pro-OMe **C3** as a white foam (16 mg, 0.05 mmol, 25% yield). (N.B. Peak doubling due to purported *N*-invertomers at the hydroxylamine nitrogen.)

R_f: 0.22 (20% EtOAc/Hex, visualized with $KMnO_4$ stain)

^{1}H NMR (400 MHz, $CDCl_3$) δ 5.13 – 4.79 (m, 1H), 4.56 – 4.40 (m, 1H), 3.76 – 3.68 (m, 3H), 3.58 (d, *J* = 16.7 Hz, 2H), 3.51 – 3.37 (m, 3H), 2.58 – 2.45 (m, 1H), 2.34 – 2.21 (m, 2H), 2.10 – 1.83 (m, 7H), 1.82 – 1.65 (m, 5H), 1.31 – 1.22 (m, 1H).

^{13}C NMR (101 MHz, $CDCl_3$) δ 173.8, 173.4, 172.3, 64.8, 64.7, 60.5, 60.4, 58.9, 58.9, 52.9, 52.9, 52.1, 52.1, 48.4, 47.9, 37.4, 37.2, 36.1, 36.1, 31.9, 31.6, 29.1, 29.1, 28.4, 28.1, 27.2, 27.2, 26.8, 26.7, 26.3, 25.9.

FTIR (solid) cm^{-1}: 2927, 2854, 1748, 1633, 1445, 1407.

Optical Rotation: $[\alpha]^{20}_D$ -29.5 (*c* = 0.327, DCM)

HRMS (ESI+) calculated for $C_{17}H_{27}N_2O_4$ $[M+H]^+$ 323.1965, found 323.1957.

HAzc(OMe)-Pro-Aib-Phe-OMe (**C4**)

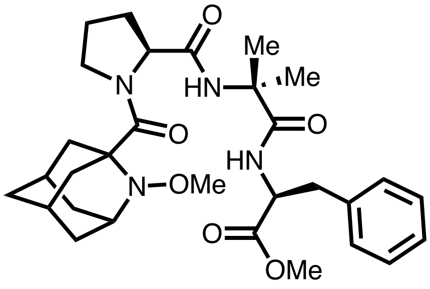

The **Peptide Coupling Procedure** was performed with H-Phe-OMe•HCl (1.08 g, 5.00 mmol, 1.00 equiv.) and Boc-Aib-OH (1.12g, 5.50 mmol, 1.10 equiv.). The resulting Boc-protected amine was then subjected to the **Boc Deprotection Procedure**. This afforded H-Aib-Phe-OMe•HCl which was coupled with Boc-Pro-OH (1.18 g, 5.50 mmol, 1.10 equiv.) using the **Peptide Coupling Procedure**. The crude trimeric peptide was then purified by reverse phase column chromatography (Biotage, SNAP Ultra C18 120 g, 0–100% MeCN/H_2O) to afford Boc-Pro-Aib-Phe-OMe as a fluffy white solid (1.02 g, 2.21 mmol, 44% yield over 3 steps).

Boc Deprotection Procedure was carried out with Boc-Pro-Aib-Phe-OMe (115 mg, 0.25 mmol, 1.00 equiv.). The resulting free amine was then carried into the **HAzc(OMe)-OH Coupling Procedure** with the product of the **HAzc(OMe)-OMe Hydrolysis Procedure** (0.25 mmol scale). The crude compound was purified by reverse phase column chromatography (Biotage, SNAP Ultra C18 30 g, 0–100% MeCN/H_2O) to provide **HAzc(OMe)-Pro-Aib-Phe-OMe** as a white foam (67.3 mg, 0.12 mmol, 49% yield over 2 steps). (N.B. Peak doubling due to purported *N*-invertomers at the hydroxylamine nitrogen.)

R_f: 0.73 (2.5% MeOH/DCM, visualized with $KMnO_4$ stain)

^{1}H NMR (600 MHz, $CDCl_3$) δ 7.56 (d, *J* = 8.2 Hz, 0.56H), 7.47 (d, *J* = 7.6 Hz, 0.44H), 7.26 – 7.15 (m, 5H), 6.50 (s, 0.56H), 6.45 (s, 0.44H), 5.19 (ddd, *J* = 10.6, 7.4, 2.4 Hz, 0.44H), 4.80 (td, *J* = 8.0, 6.4 Hz, 0.56H), 4.73 (q, *J* = 7.0 Hz, 0.44H), 4.63 (dt, *J* = 11.5, 5.5 Hz, 0.56H), 4.44 (dd, *J* = 8.5, 4.5 Hz, 0.56H), 4.32 (t, *J* = 7.3 Hz, 0.44H), 3.78 (dt, *J* = 11.9, 7.1 Hz, 0.56H), 3.66 (s, 1.68H), 3.64 (s, 1.32H), 3.52 – 3.50 (m, 0.56 H), 3.47 (s, 2.12H), 3.44 – 3.42 (m, 0.44H), 3.41 (s, 1.32H), 3.23 – 3.01 (m, 2H), 2.51 – 2.41 (m, 1H), 2.29 – 2.16 (m, 2H), 2.09 – 2.00 (m, 4H), 1.93 – 1.53 (m, 8H), 1.51 (s, 1.32H), 1.49 (s, 1.68H), 1.44 (s, 1.32H), 1.32 (s, 1.68H), 1.30 – 1.23 (m, 1H).

^{13}C NMR (151 MHz, $CDCl_3$) δ 174.4, 174.3, 174.1, 173.5, 172.3, 172.3, 171.6, 137.3, 137.2, 129.5, 129.4, 128.5, 128.3, 126.8, 126.7, 65.0, 64.8, 62.6, 62.5, 58.9, 58.4, 57.4, 57.4, 54.2, 53.7, 52.6, 52.2, 52.1, 51.8, 49.2, 48.1, 38.1, 37.9, 37.2, 37.1, 36.0, 36.0, 35.9, 35.9, 32.3, 31.8, 28.9, 28.8, 27.8, 27.2, 27.1, 27.1, 26.9, 26.6, 26.6, 26.3, 26.1, 25.3, 24.6.

FTIR (solid) cm^{-1}: 3326, 2925, 2854, 1749, 1668, 1615, 1528, 1445.

Optical Rotation: $[\alpha]^{20}_D$ -8.04 (*c* = 0.520, DCM)

HRMS (ESI+) calculated for $C_{30}H_{43}N_4O_6$ $[M+H]^+$ 555.3177, found 555.3161.

3. Synthesis of Erythromycin Derivatives

Erythromycin A *N*-Oxide (**2**)

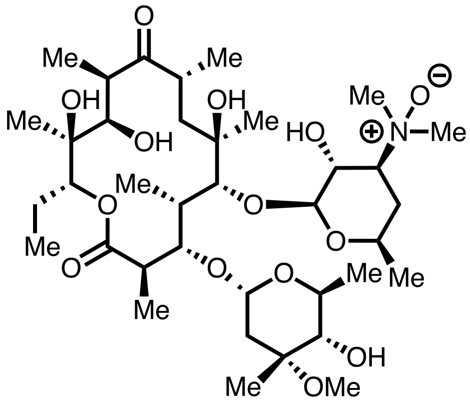

A 25 mL flask equipped with a stir bar was charged with erythromycin A **1** (367 mg, 0.50 mmol, 1.00 equiv.). DCM (10.0 mL) was added followed by *m*CPBA (240 mg, 1.00 mmol, 2.00 equiv.). The resulting suspension was vigorously stirred at room temperature for 30 minutes. The reaction was quenched by addition of excess DMS upon complete consumption of **1** as determined by UPLC/MS then stirred for an additional 1 hour. The reaction was then diluted with $CHCl_3$:IPA (3:1) and washed with $NaHCO_{3(aq)}$ (saturated, 3x). The organic layer was dried over Na_2SO_4 and concentrated via rotary evaporation. The crude white solid was then purified via SiO_2 column chromatography (25–30% MeOH/DCM) to afford erythromycin A *N*-oxide **2** as a white powder (317 mg, 0.422 mmol, 84% yield).

Erythromycin A *N*-oxide has previously been reported in the literature.[9] In-house data is reported herein.

R_f: 0.45 (25% MeOH/DCM, visualized with $KMnO_4$ stain)

^{1}H NMR (600 MHz, $CDCl_3$) δ 5.02 (dt, *J* = 10.9, 1.9 Hz, 1H), 4.88 (d, *J* = 4.8 Hz, 1H), 4.52 (d, *J* = 7.0 Hz, 1H), 4.02 – 3.95 (m, 2H), 3.81 (d, *J* = 2.4 Hz, 1H), 3.76 (td, *J* = 7.3, 3.5 Hz, 1H), 3.61 (ddd, *J* = 10.6, 6.0, 1.9 Hz, 1H), 3.57 (dd, *J* = 7.8, 2.6 Hz, 1H), 3.35 (s, 3H), 3.21 – 3.17 (m, 6H), 3.09 (d, *J* = 6.6 Hz, 1H), 3.02 (d, *J* = 9.3 Hz, 1H), 2.93 – 2.86 (m, 1H), 2.67 (ddt, *J* = 14.6, 7.7, 3.8 Hz, 1H), 2.37 (d, *J* = 15.1 Hz, 1H), 2.00 – 1.94 (m, 3H), 1.90 (ddd, *J* = 14.4, 7.6, 2.2 Hz, 1H), 1.71 (d, *J* = 15.1 Hz, 1H), 1.57 (ddd, *J* = 15.1, 5.0, 1.5 Hz, 1H), 1.51 – 1.47 (m, 1H), 1.46 (s, 3H), 1.37 – 1.32 (m, 1H), 1.29 – 1.26 (m, 6H), 1.24 (d, *J* = 1.1 Hz, 3H), 1.19 – 1.13 (m, 13H), 1.11 (s, 3H), 0.84 (td, *J* = 7.4, 1.3 Hz, 3H). (N.B. The exchangeable protons not observed.)

^{1}H NMR (500 MHz, DMSO-d_6) δ 5.10 (dd, *J* = 10.9, 2.4 Hz, 1H), 4.74 (d, *J* = 4.8 Hz, 1H), 4.55 (s, 1H), 4.44 (d, *J* = 7.0 Hz, 1H), 4.28 (s, 1H), 4.22 (d, *J* = 8.7 Hz, 1H), 4.06 – 3.97 (m, 2H), 3.86 (d, *J* = 5.8 Hz, 1H), 3.76 (dd, *J* = 5.8, 2.1 Hz, 1H), 3.72 – 3.64 (m, 1H), 3.50 (dd, *J* = 10.1, 7.0 Hz, 1H), 3.46 (d, *J* = 7.6 Hz, 1H), 3.24 (s, 4H), 3.03 (s, 3H), 3.00 (s, 3H), 2.92 – 2.83 (m, 3H), 2.77 (dq, *J* = 9.6, 7.0 Hz, 1H), 2.30 (d, *J* = 15.0 Hz, 1H), 1.97 – 1.92 (m, 1H), 1.89 (t, *J* = 7.6 Hz, 1H), 1.84 – 1.75 (m, 1H), 1.68 (dd, *J* = 14.8, 5.9 Hz, 1H), 1.52 (dd, *J* = 15.1, 5.1 Hz, 1H), 1.42 –

1.35 (m, 1H), 1.31 – 1.23 (m, 5H), 1.17 (d, J = 6.2 Hz, 3H), 1.14 (s, 3H), 1.12 – 1.08 (m, 6H), 1.06 – 1.01 (m, 12H), 0.76 (t, J = 7.4 Hz, 3H).

^{13}C{^{1}H} NMR (151 MHz, $CDCl_3$) δ 222.5, 176.0, 102.9, 96.4, 83.8, 80.1, 78.1, 76.9, 76.4, 75.1, 74.7, 72.8, 72.7, 69.1, 67.3, 65.7, 59.2, 52.1, 49.8, 45.5, 44.9, 39.6, 38.6, 37.8, 35.1, 35.1, 27.2, 21.7, 21.4, 21.2, 18.9, 18.4, 16.3, 16.2, 12.2, 10.8, 9.2.

^{13}C{^{1}H} NMR (151 MHz, DMSO-d_6) δ 217.9, 174.7, 102.0, 95.9, 83.1, 79.2, 77.5, 75.8, 74.8, 74.7, 73.3, 72.6, 72.2, 68.8, 66.2, 64.9, 59.0, 51.6, 48.9, 44.4, 42.4, 39.5, 39.1, 38.3, 34.9, 34.0, 27.0, 21.2, 21.0, 20.9, 18.6, 18.2, 17.4, 15.9, 11.3, 10.6, 9.0.

FTIR (solid) cm^{-1}: 3474, 2971, 2937, 1726, 1700, 1457, 1376.

Optical Rotation: $[\alpha]^{20}_{D}$ -52.3 (c = 0.280, MeOH)

HRMS (ESI+) calculated for $C_{37}H_{68}NO_{14}$ $[M+H]^+$ 750.4634, found 750.4617.

Full assignment of the ^{1}H and ^{13}C NMR shifts in DMSO-d_6 was undertaken to support the structural assignment of **5’** using a combination of ^{1}H, ^{13}C, COSY, HSQC, and HMBC NMR techniques. Each H and C were assigned to the structure, and carbon numbering (red) is consistent in all following compounds (**Figure S1**). Full spectra are included in Section 11 (*vide infra*).

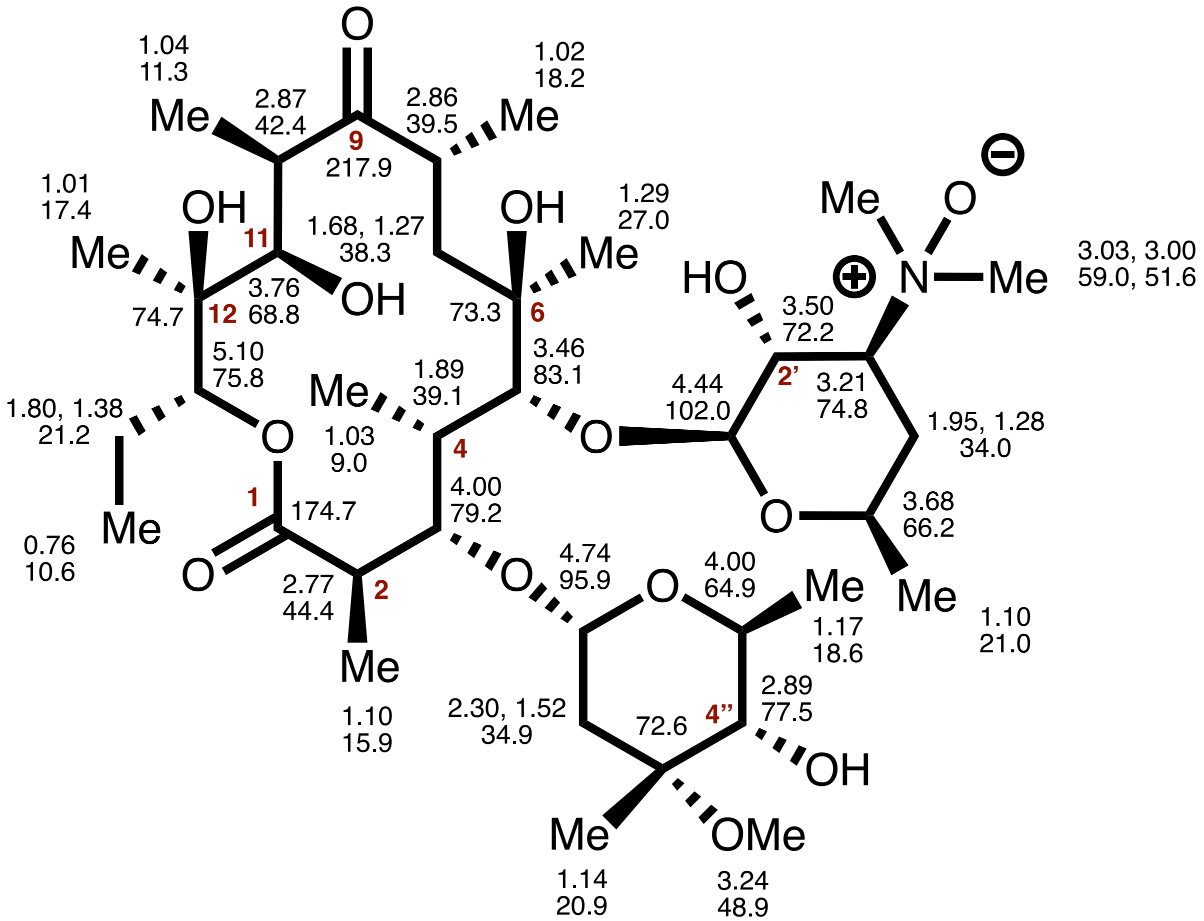

Figure S1. ^{1}H (top) and ^{13}C (bottom) assignments for erythromycin A *N*-oxide **2** in DMSO-d_6. Red numbers indicate the carbon numbering used.

C11-Keto-Erythyromycin *N*-Oxide (**5’**)

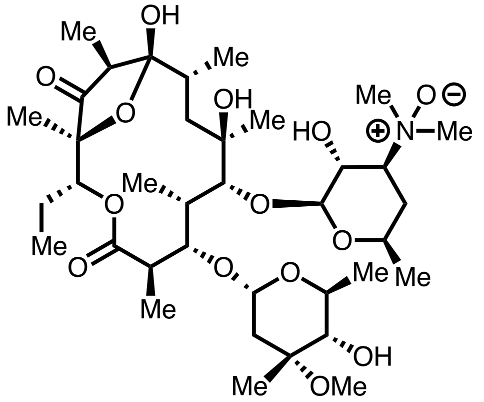

A 250 mL flask equipped with a stir bar was charged with erythromycin A **1** (3.67 g, 5.00 mmol, 1.00 equiv.), Na_2HPO_4 (1.41 g, 10.0 mmol, 2.00 equiv.), and keto-ABNO (308 mg, 2.00 mmol, 0.40 equiv.). DCM (100.0 mL) was added followed by *m*CPBA (5.99 g, 25.0 mmol, 5.00 equiv.). The resulting suspension was vigorously stirred at room temperature for 24 hr. The reaction was quenched by addition of excess DMS then stirred for 1 hour. The reaction was then diluted with $CHCl_3$:IPA (3:1) and washed with $NaHCO_{3(aq)}$ (saturated, 3x). The organic layer was dried over Na_2SO_4 and concentrated via rotary evaporation. The crude yellow solid was then purified via reverse phase preparatory HPLC (5–20% H_2O/MeCN over 0–5 min, 20–50% H_2O/MeCN over 5–50 min, 50–100% H_2O/MeCN over 50–54 min, hold 100% MeCN for 54–55 min, 100–5% H_2O/MeCN over 55–58 min, and hold 5% H_2O/MeCN for 58–60 min. Typical elution times ~ 39–42 min). C11-Keto-erythyromycin *N*-oxide **5’** was collected as a white powder (492 mg, 0.658 mmol, 13% yield).

Alternatively, C11-Keto-erythyromycin *N*-oxide **5’** can be synthesized using the following procedure: A one dram vial equipped with a stir bar was charged with erythromycin A **1** (36.7 mg, 0.05 mmol, 1.00 equiv.), Na_2HPO_4 (71.0 mg, 0.50 mmol, 10.0 equiv.), and **HAzc(OMe)-OMe** (4.5 mg, 0.02 mmol, 0.40 equiv.). DCE (1.00 mL) was added followed by *m*CPBA (120 mg, 0.50 mmol, 10.00 equiv.). The resulting suspension was vigorously stirred at room temperature for 24 hr. The reaction was quenched by addition of excess DMS then stirred for 1 hour. The reaction was then diluted with $CHCl_3$:IPA (3:1) and washed with $NaHCO_{3(aq)}$ (saturated, 3x). The organic layer was dried over Na_2SO_4 and concentrated via rotary evaporation. The crude yellow solid was then analyzed via ^1H NMR. Crude reaction mixtures were found to be analogous using either procedure.

Structural confirmation was obtained via X-ray crystallography (*vida infra*, Section 12).

R_f: 0.37 (10% MeOH/DCM, visualized with $KMnO_4$ stain)

^{1}H NMR (600 MHz, $CDCl_3$) δ 4.90 (dd, *J* = 9.9, 3.4 Hz, 1H), 4.78 (d, *J* = 4.7 Hz, 1H), 4.59 – 4.52 (m, 2H), 4.06 (dq, *J* = 9.1, 6.3 Hz, 1H), 3.79 (dd, *J* = 10.1, 7.1 Hz, 1H), 3.73 (d, *J* = 9.0 Hz, 1H), 3.68 (ddd, *J* = 10.8, 6.1, 1.8 Hz, 1H), 3.56 (d, *J* = 5.9 Hz, 1H), 3.44 – 3.36 (m, 2H), 3.32 (s, 3H), 3.27 (s, 3H), 3.21 – 3.17 (m, 4H), 3.02 (d, *J* = 9.3 Hz, 1H), 2.53 (qd, *J* = 7.2, 5.3 Hz, 1H), 2.47 (q,

J = 7.0 Hz, 1H), 2.42 – 2.37 (m, 2H), 2.16 – 2.10 (m, 1H), 1.99 (d, J = 13.4 Hz, 1H), 1.95 (td, J = 6.5, 3.5 Hz, 1H), 1.82 (dqt, J = 15.3, 7.7, 4.0 Hz, 1H), 1.62 – 1.58 (m, 1H), 1.55 (dd, J = 15.1, 4.9 Hz, 1H), 1.47 (s, 3H), 1.39 – 1.36 (m, 1H), 1.29 (dd, J = 6.3, 4.8 Hz, 7H), 1.25 (d, J = 7.1 Hz, 4H), 1.22 (d, J = 7.7 Hz, 6H), 1.19 (d, J = 7.4 Hz, 3H), 1.17 (d, J = 7.0 Hz, 3H), 1.15 (d, J = 7.1 Hz, 3H), 0.85 (t, J = 7.4 Hz, 3H).

^{1}H NMR (800 MHz, DMSO-d_6) δ 4.84 (tt, J = 7.7, 3.1 Hz, 1H), 4.65 (t, J = 4.1 Hz, 1H), 4.38 (d, J = 7.3 Hz, 1H), 4.31 (dt, J = 8.7, 4.3 Hz, 1H), 4.21 (d, J = 8.6 Hz, 1H), 4.05 (dq, J = 13.7, 7.2 Hz, 1H), 3.68 (s, 1H), 3.64 – 3.59 (m, 1H), 3.60 – 3.55 (m, 1H), 3.39 (t, J = 7.5 Hz, 2H), 3.19 (d, J = 7.1 Hz, 3H), 3.06 (d, J = 7.2 Hz, 3H), 3.03 (d, J = 7.1 Hz, 3H), 2.86 (t, J = 8.7 Hz, 1H), 2.62 (t, J = 7.2 Hz, 1H), 2.58 (q, J = 7.2 Hz, 1H), 2.33 – 2.29 (m, 1H), 2.26 (dd, J = 15.2, 7.0 Hz, 1H), 2.01 – 1.98 (m, 1H), 1.92 (q, J = 7.5 Hz, 1H), 1.82 – 1.76 (m, 2H), 1.63 – 1.59 (m, 1H), 1.49 – 1.45 (m, 2H), 1.37 (d, J = 7.1 Hz, 3H), 1.34 (d, J = 11.7 Hz, 1H), 1.23 (s, 1H), 1.17 – 1.13 (m, 9H), 1.13 – 1.09 (m, 7H), 1.06 (d, J = 7.2 Hz, 3H), 1.04 – 1.00 (m, 6H), 0.75 (t, J = 7.5 Hz, 3H).

^{13}C{^{1}H} NMR (151 MHz, $CDCl_3$) δ 216.6, 177.4, 106.9, 103.9, 97.7, 82.9, 79.2, 78.6, 78.1, 77.8, 76.6, 74.3, 72.7, 72.0, 68.1, 66.1, 65.3, 49.7, 47.8, 46.8, 44.1, 40.2, 38.3, 35.1, 34.6, 29.8, 25.9, 25.0, 23.1, 21.7, 21.1, 18.1, 18.0, 14.8, 11.0, 10.5, 10.2.

^{13}C{^{1}H} NMR (201 MHz, DMSO-d_6) δ 217.0, 177.1, 106.8, 103.0, 96.7, 83.7, 81.4, 78.1, 77.6, 77.6, 74.6, 73.7, 72.7, 71.5, 67.3, 64.9, 58.8, 52.1, 48.9, 47.4, 46.8, 42.7, 38.9, 35.9, 34.8, 33.8, 26.0, 24.0, 22.0, 21.0, 20.7, 18.5, 17.9, 15.7, 10.7, 10.5, 10.1.

FTIR (solid) cm^{-1}: 2971, 2934, 2850, 1730, 1457, 1373.

Optical Rotation: $[\alpha]^{20}_D$ -29.2 (c = 0.660, MeOH)

HRMS (ESI+) calculated for $C_{37}H_{66}NO_{14}$ $[M+H]^+$ 748.4478, found 748.4458.

Full assignment of the ^{1}H and ^{13}C NMR were undertaken to support the structural assignment of **5'** using a combination of ^{1}H, ^{13}C, COSY, HSQC, and HMBC NMR techniques. Each H and C were assigned to the structure (**Figure S2**). Full spectra are included in Section 11 (*vide infra*). Assignment was further corroborated by ^{13}C calculations detailed in Section 9 and X-ray crystallography detailed in Section 12.

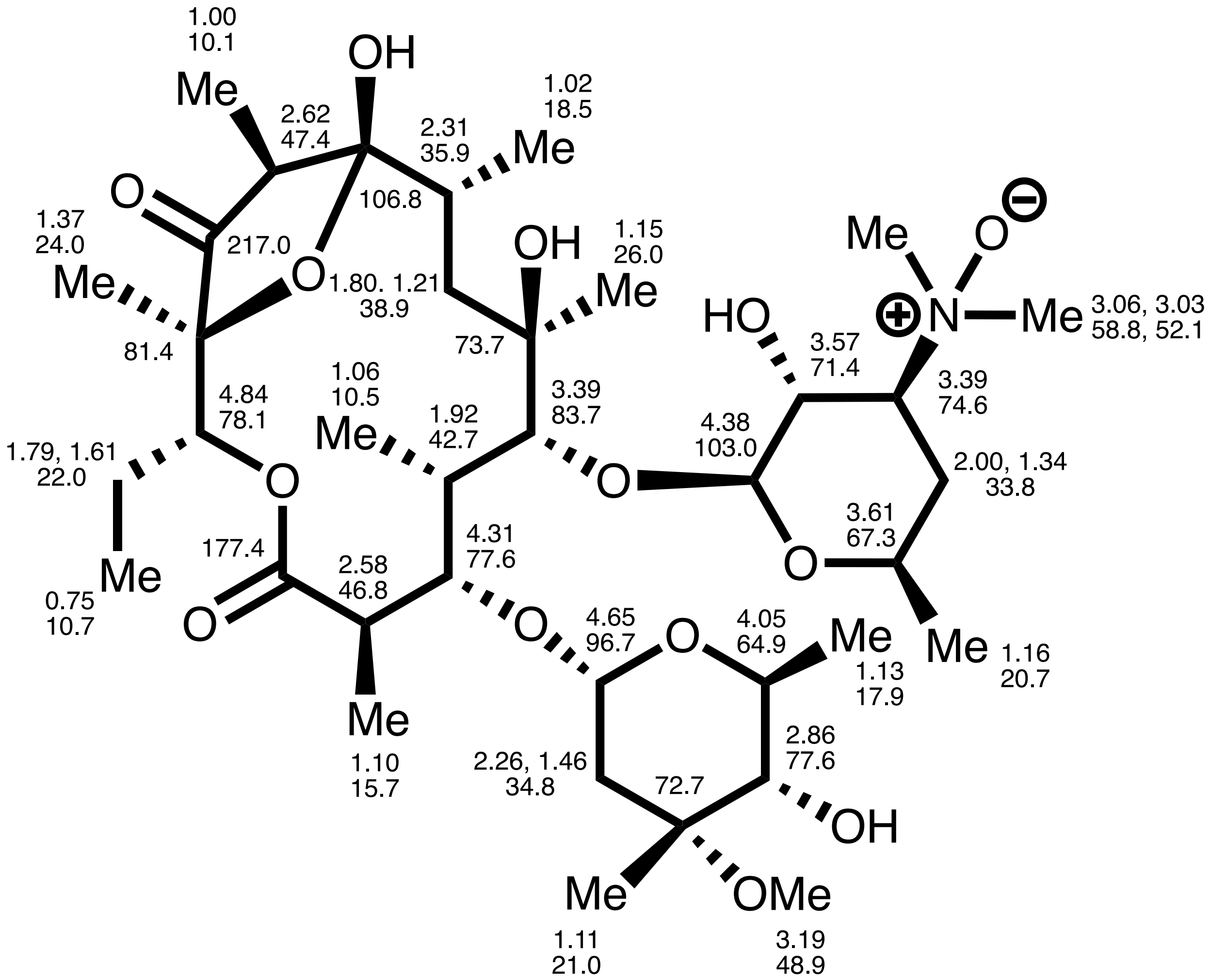

Figure S2. ^{1}H (top) and ^{13}C (bottom) assignments for C11-keto-erythromycin *N*-oxide **5'** in DMSO-d_6.

Overlay of the HSQC spectra for **2** (green) and **5'** (red) demonstrates the preservation of the sp^3 C2' (**2**: 3.50, 72.2; **5'**: 3.57, 71.4) and C4'' (**2**: 2.89, 77.5; **5'**: 2.86, 77.5) carbons based on the observed connectivity of the ^{1}H signal (**Figure S3**). The C11 proton (**2**: 3.76, 68.8) is no longer observed in **5'**, supporting the assertion that oxidation has taken place at that site. Note that only one carbonyl is observed due to the formation of the C9,C12-hemiketal indicated by the appearance of the new ^{13}C shift at 106.8 ppm.

Additionally, UPLC/MS shows that the fragmentation mass resulting from the loss of the cladinose sugar (-158) still retains evidence of the oxidation (-2) when compared with **2**. This indicates that oxidation does not take place on cladinose. The relevant masses observed in the ESI$^+$ for **2** are 750 and 592 vs for **5'** at 748 and 590 (**Figure S4**).

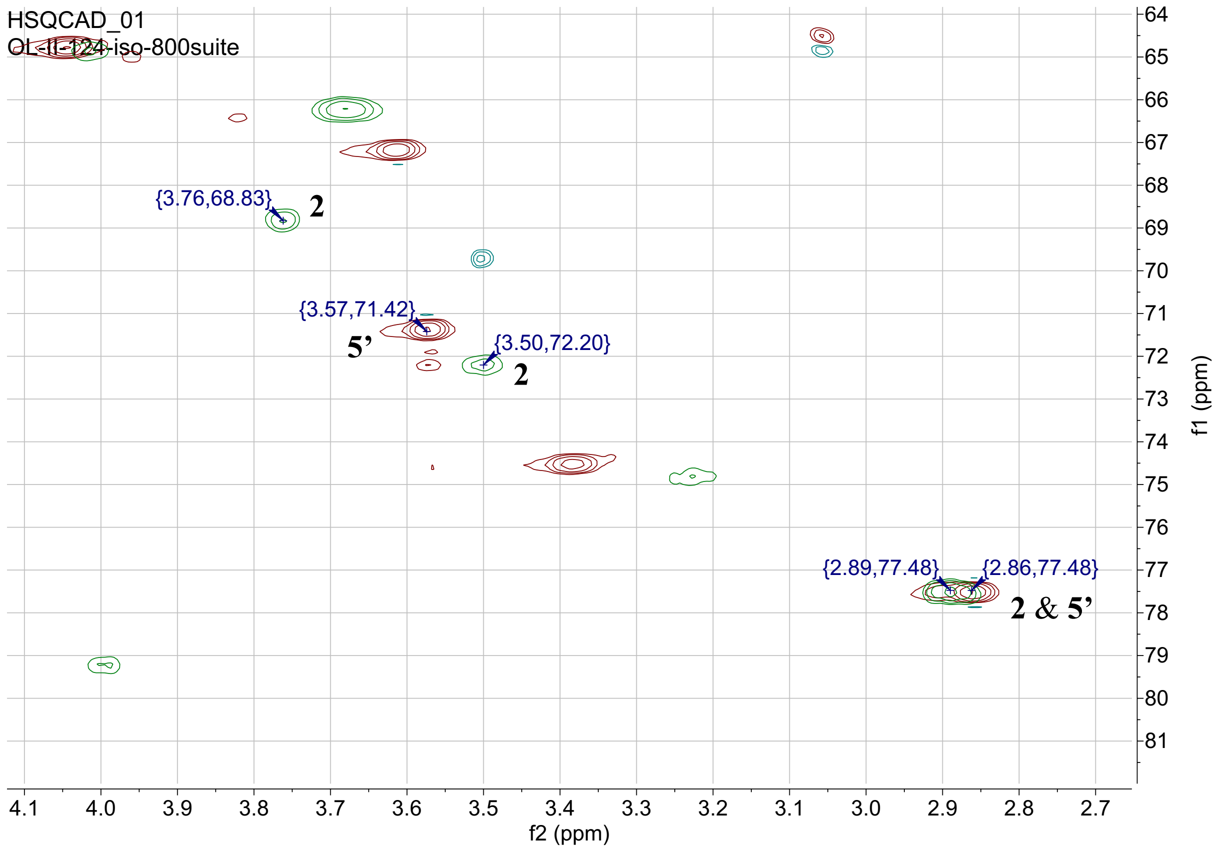

Figure S3. Overlay of HSQC in DMSO-d_6 for **2** (green) and **5’** (red) highlighting loss of the C11 proton of **5’**.

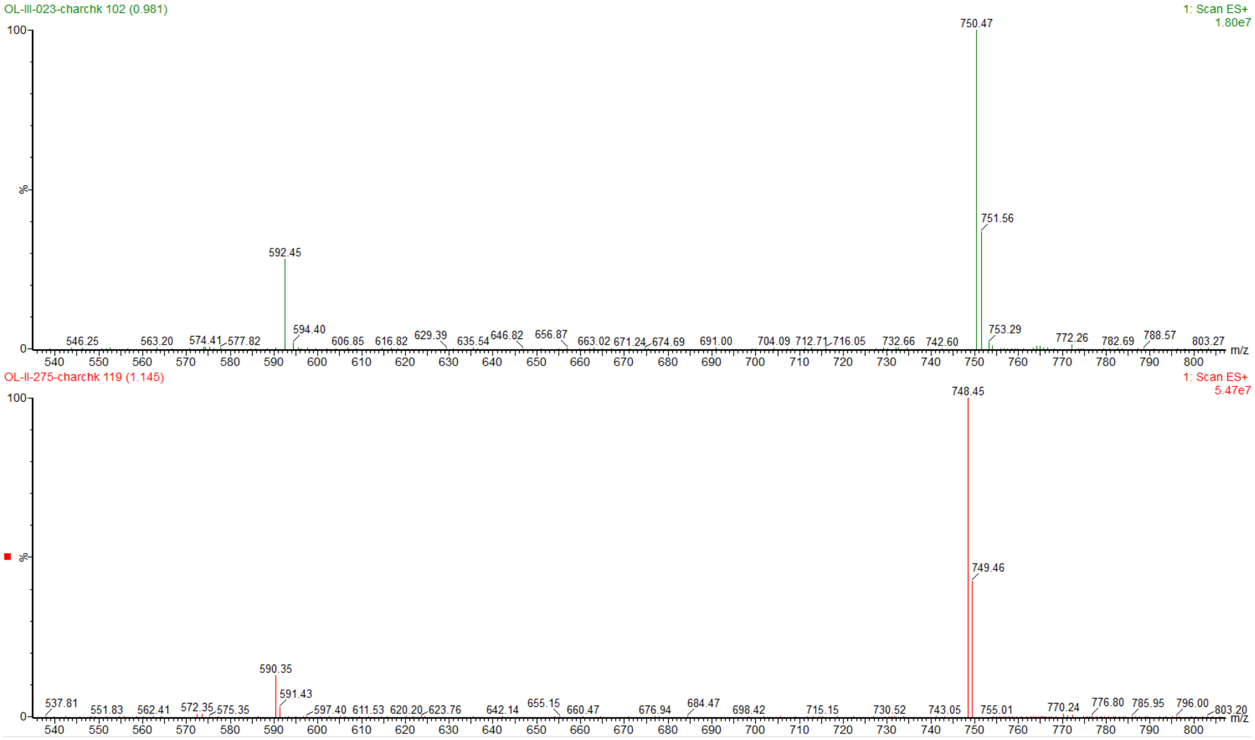

Figure S4. UPLC/MS trace of **2** (top) and **5’** (bottom) showing key fragmentation.

C11-Keto-Erythyromycin (**11**)

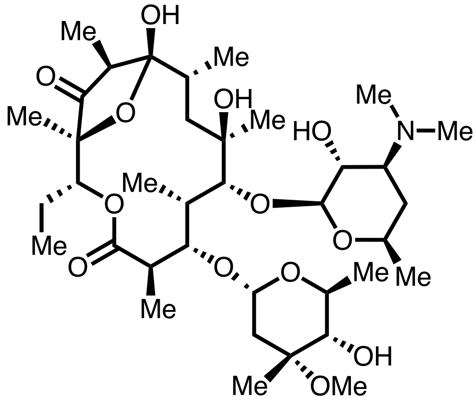

A 50 mL flask equipped with a stir bar was charged with C11-keto-erythyromycin *N*-oxide **5'** (175 mg, 0.23 mmol, 1.00 equiv.). EtOH (15.8 mL) was added followed by sparging with N_2. Raney Ni® 2800 (2.7 mL) was then added, and the resulting suspension was vigorously stirred at room temperature for 30 min while sparging with $N_{2(g)}$. The reaction was then stirred open to air before it was filtered over Celite® rinsing with H_2O and $CHCl_3$:IPA (3:1). Brine was added, and the biphasic mixture was extracted with $CHCl_3$:IPA (3:1, 3x). The combined organic layers were dried over Na_2SO_4 and concentrated via rotary evaporation to afford C11-Keto-erythyromycin **11** as a white powder (152 mg, 0.21 mmol, 89% yield) without further purification.[9]

R_f: 0.56 (10% MeOH/DCM, visualized with $KMnO_4$ stain)

^{1}H NMR (600 MHz, $CDCl_3$) δ 5.08 (dd, *J* = 10.1, 3.3 Hz, 1H), 4.74 (d, *J* = 5.0 Hz, 1H), 4.47 (s, 1H), 4.36 (d, *J* = 7.3 Hz, 1H), 4.15 – 4.08 (m, 2H), 3.64 – 3.55 (m, 1H), 3.48 (d, *J* = 2.6 Hz, 1H), 3.40 (dd, *J* = 10.2, 7.2 Hz, 1H), 3.25 (d, *J* = 0.9 Hz, 3H), 3.00 (d, *J* = 9.5 Hz, 1H), 2.63 (td, *J* = 11.4, 3.8 Hz, 1H), 2.49 (t, *J* = 7.4 Hz, 1H), 2.44 (tq, *J* = 7.0, 3.4 Hz, 2H), 2.32 (d, *J* = 8.6 Hz, 9H), 1.95 – 1.88 (m, 2H), 1.81 – 1.75 (m, 1H), 1.71 (ddd, *J* = 14.6, 7.3, 2.7 Hz, 2H), 1.52 (dd, *J* = 15.3, 5.2 Hz, 1H), 1.44 (s, 3H), 1.36 (dd, *J* = 9.3, 7.2 Hz, 1H), 1.28 (d, *J* = 6.3 Hz, 3H), 1.26 (s, 3H), 1.24 – 1.21 (m, 7H), 1.20 (s, 3H), 1.19 (d, *J* = 7.1 Hz, 3H), 1.16 (d, *J* = 7.0 Hz, 3H), 1.06 (d, *J* = 7.2 Hz, 3H).

^{13}C{^{1}H} NMR (151 MHz, $CDCl_3$) δ 216.6, 176.9, 106.9, 106.8, 99.1, 83.2, 81.5, 79.5, 77.6, 72.7, 72.6, 70.6, 69.6, 66.5, 64.3, 49.5, 49.2, 47.5, 42.9, 42.2, 40.7, 40.4, 38.0, 35.1, 29.8, 25.3, 24.4, 22.9, 21.5, 21.2, 17.7, 17.6, 16.6, 10.9, 10.8, 10.2. (N.B. The methyl groups are equivalent.)

FTIR (solid) cm^{-1}: 3431, 2971, 1755, 1730, 1456, 1374, 1263.

Optical Rotation: $[\alpha]^{20}_D$ -2.23 (*c* = 2.49, MeOH)

HRMS (ESI+) calculated for $C_{37}H_{66}NO_{13}$ $[M+H]^+$ 732.4529, found 732.4525.

C11,C4''-Diketo-Erythromycin *N*-Oxide (**12**)

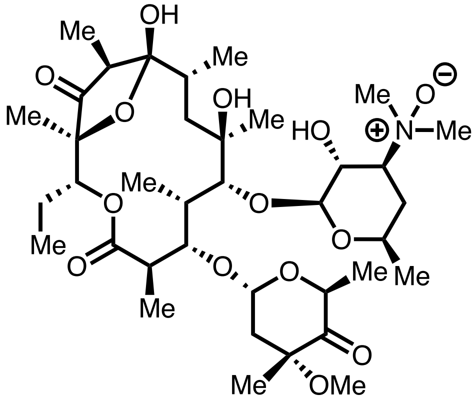

A 2-dram vial equipped with a stir bar was charged with C11-keto-erythromycin *N*-oxide **5'** (90.3 mg, 0.12 mmol, 1.00 equiv.). DCM (2.40 mL) was added followed by DMP (102.4 mg, 0.24 mmol, 2.00 equiv.). The resulting suspension was vigorously stirred at room temperature for 24 hr. The reaction was quenched by addition of excess DMS then stirred for an additional 1 hour. The reaction was then diluted with $CHCl_3$:IPA (3:1) and washed with $NaHCO_{3(aq)}$ (saturated, 3x). The organic layer was dried over Na_2SO_4 and concentrated via rotary evaporation. The crude yellow solid was then purified via reverse phase preparatory HPLC (5–20% H_2O/MeCN over 0–5 min, 20–50% H_2O/MeCN over 5–50 min, 50–100% H_2O/MeCN over 50–54 min, hold 100% MeCN for 54–55 min, 100–5% H_2O/MeCN over 55–58 min, and hold 5% H_2O/MeCN for 58–60 min. Typical elution times ~ 45–48 min). C11,C4''-Diketo-erythromycin *N*-oxide **12** was collected as a white powder (43.5 mg, 0.058 mmol, 48% yield).

R_f: 0.45 (10% MeOH/DCM, visualized with $KMnO_4$ stain)

^{1}H NMR (500 MHz, $CDCl_3$) δ 5.08 (t, *J* = 7.0 Hz, 1H), 4.95 (dd, *J* = 9.6, 3.7 Hz, 1H), 4.50 (d, *J* = 7.2 Hz, 1H), 4.48 – 4.44 (m, 1H), 4.25 (t, *J* = 8.1 Hz, 1H), 3.82 (dd, *J* = 9.9, 7.2 Hz, 1H), 3.73 (dtd, *J* = 11.1, 6.7, 5.0 Hz, 1H), 3.54 (d, *J* = 1.9 Hz, 1H), 3.53 – 3.48 (m, 1H), 3.35 (s, 3H), 3.27 (s, 3H), 3.23 (s, 3H), 2.54 – 2.47 (m, 2H), 2.45 (q, *J* = 6.9 Hz, 1H), 2.29 (d, *J* = 7.0 Hz, 2H), 2.22 – 2.17 (m, 1H), 1.91 (d, *J* = 12.9 Hz, 1H), 1.85 (td, *J* = 7.4, 1.8 Hz, 1H), 1.81 (td, *J* = 7.4, 3.7 Hz, 1H), 1.77 – 1.71 (m, 1H), 1.51 (d, *J* = 12.3 Hz, 1H), 1.41 (s, 3H), 1.38 (s, 3H), 1.35 – 1.32 (m, 6H), 1.27 (d, *J* = 7.3 Hz, 3H), 1.21 (s, 3H), 1.19 – 1.16 (m, 7H), 1.10 (d, *J* = 7.0 Hz, 3H), 0.86 (d, *J* = 7.3 Hz, 3H).

^{13}C{^{1}H} NMR (151 MHz, $CDCl_3$) δ 216.2, 211.3, 176.8, 107.0, 104.2, 98.8, 87.2, 82.9, 80.0, 79.6, 77.3, 76.6, 72.7, 72.5, 70.7, 68.5, 57.0, 55.3, 51.7, 49.0, 47.8, 42.6, 41.9, 37.8, 36.5, 34.1, 24.2, 23.6, 22.8, 21.7, 21.1, 18.1, 17.0, 15.3, 11.8, 10.9, 10.1.

FTIR (solid) cm^{-1}: 2981, 2927, 2853, 1694, 1460, 1370.

Optical Rotation: $[\alpha]^{20}_D$ -15.73 (*c* = 0.150, MeOH)

HRMS (ESI+) calculated for $C_{37}H_{64}NO_{14}$ $[M+H]^+$ 746.4321, found 746.4301.

Full assignment of the ^{1}H and ^{13}C NMR were undertaken to support the structural assignment of **12** using a combination of ^{1}H, ^{13}C, COSY, HSQC, and HMBC NMR techniques. Each H and C were assigned to the structure (**Figure S5**). Full spectra are included in Section 11 (*vide infra*).

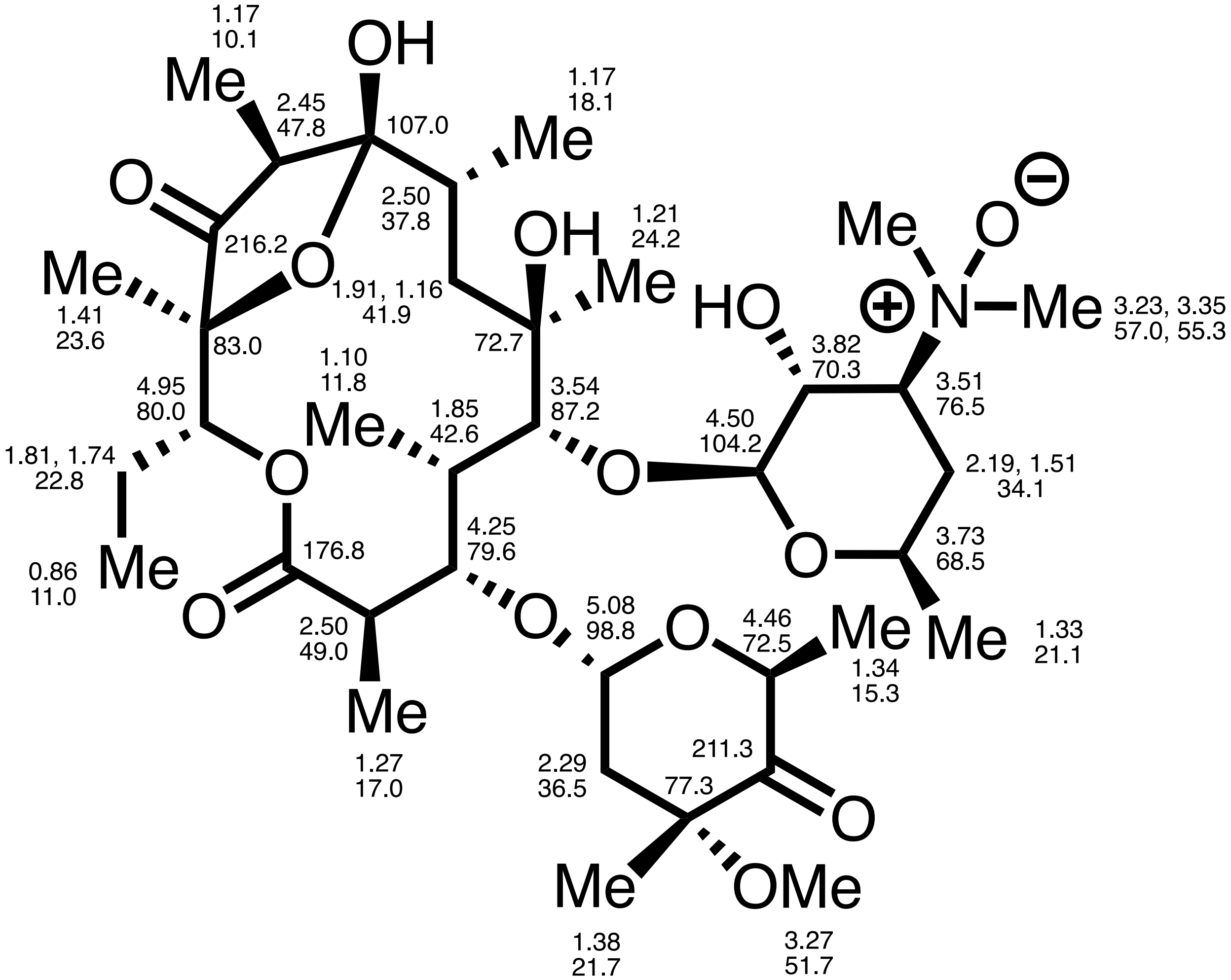

Figure S5. ^{1}H (top) and ^{13}C (bottom) assignments for C11,C4''-diketo-erythromycin *N*-oxide **12** in $CDCl_3$.

HMBC correlations can be seen between the newly installed C4'' ketone (211.3) and many ^{1}H shifts of the cladinose sugar (4.46, 2.29, 1.38, and 1.34) (**Figure S6**). Additionally, an overlay of the HSQC spectra for **12** (red) and **8** (green) demonstrates that many ^{1}H shifts of the cladinose sugar are consistent with those observed in the monooxidation of clarithromycin to form C4''-keto-clarithromycin (**8**, *vide infra*) (e.g. **12** vs **8**: 5.08 vs 5.17; 4.46 vs 4.39; 2.29 vs 2.3 and 2.23) (**Figure S7**). The carbonyl carbons of **12** and **8** also have nearly identical shifts (211.3 vs 211.2).

Additionally, UPLC/MS shows that the fragmentation mass resulting from the loss of the cladinose sugar (-158) still retains evidence of a single oxidation (-2) when compared with **2** rather than a double oxidation (-4). This indicates that one oxidation takes place on cladinose. The relevant masses observed in the ESI^{+} for **2** are 750 and 592 vs for **12** at 746 and 590 (**Figure S8**).

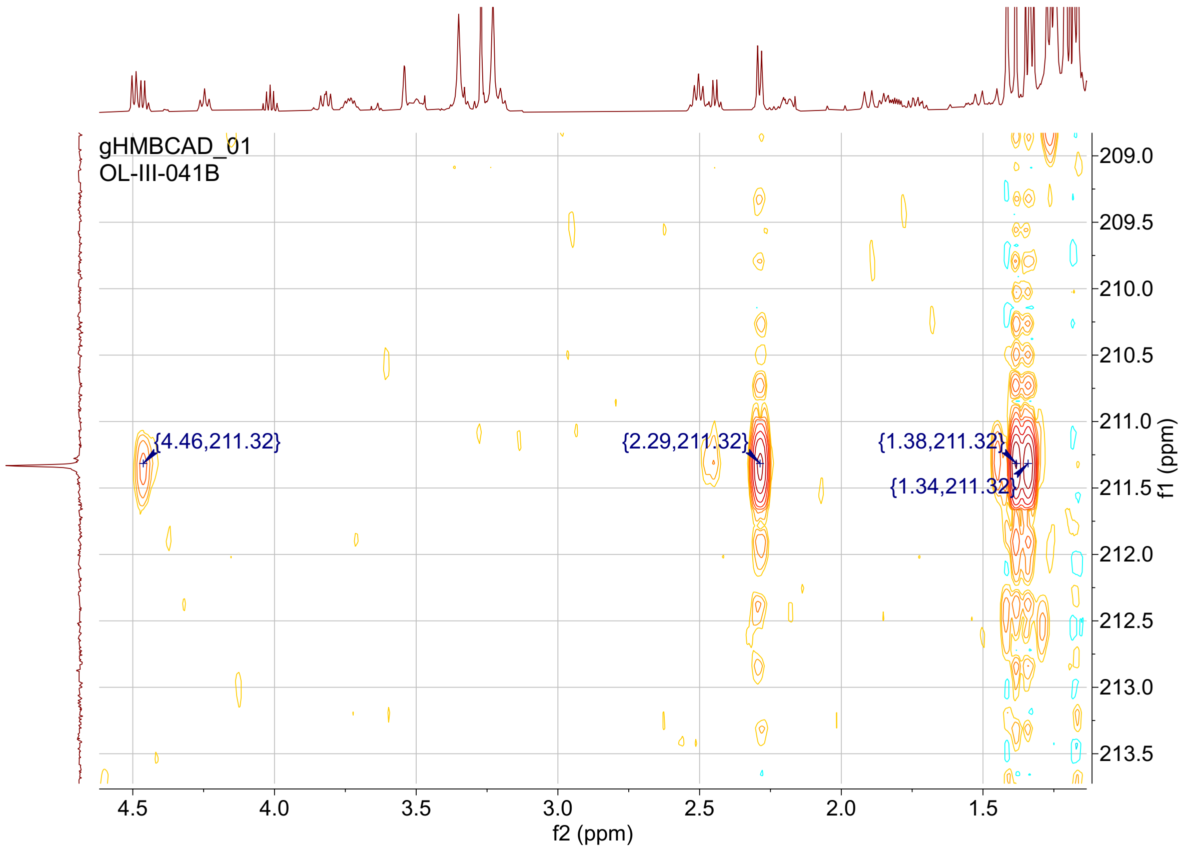

Figure S6. HMBC of **12** in $CDCl_3$ highlighting correlations between the new carbonyl C (211.3) and key shifts on the cladinose sugar (4.46, 2.29, 1.38, and 1.34).

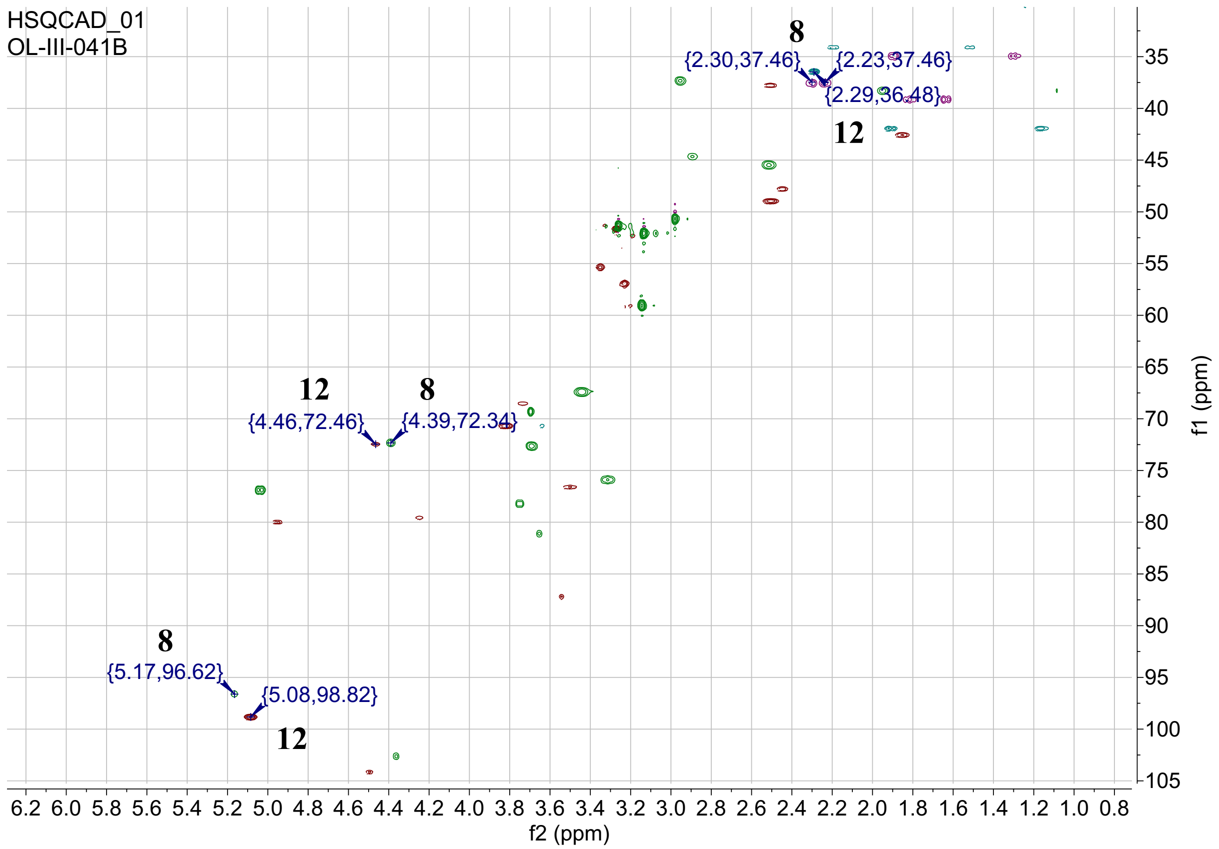

Figure S7. Overlay of HSQC in $CDCl_3$ for **8** (green) and **12** (red) highlighting the consistency of key shifts on the cladinose sugar.

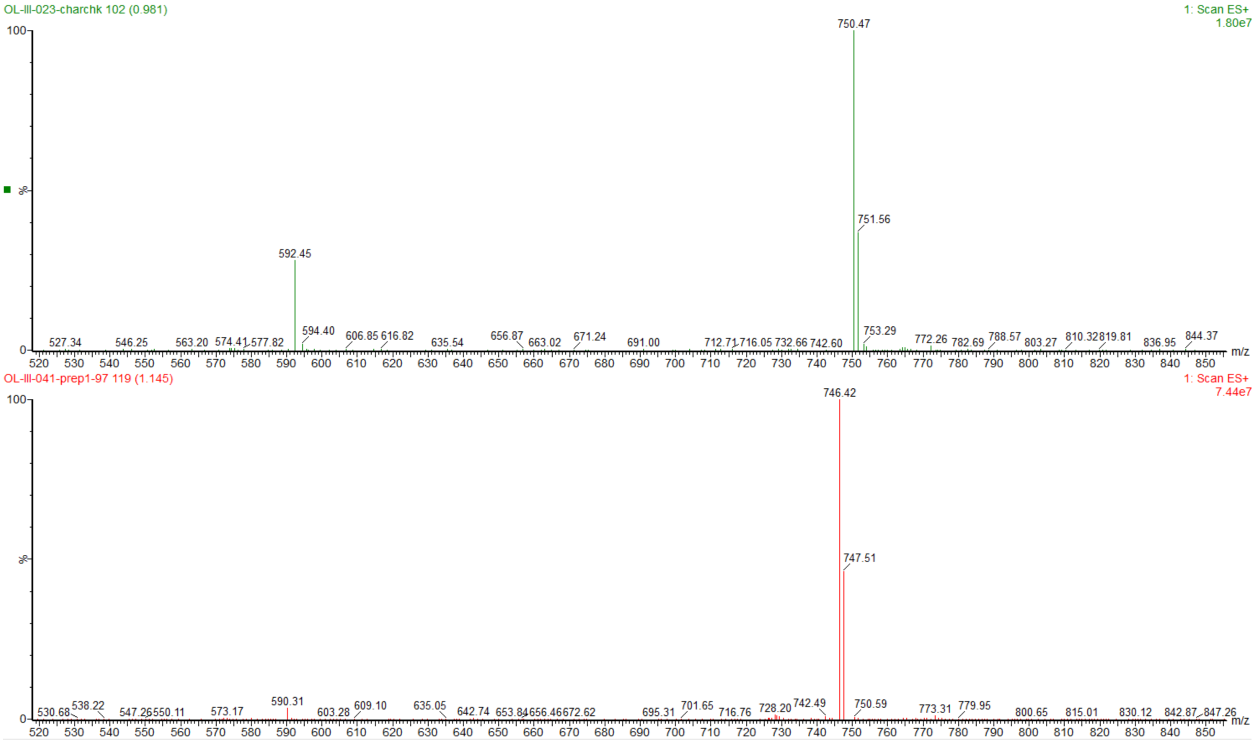

Figure S8. UPLC/MS trace of **2** (top) and **12** (bottom) showing key fragmentation.

C11,C4''-Diketo-Erythyromycin (**13**)

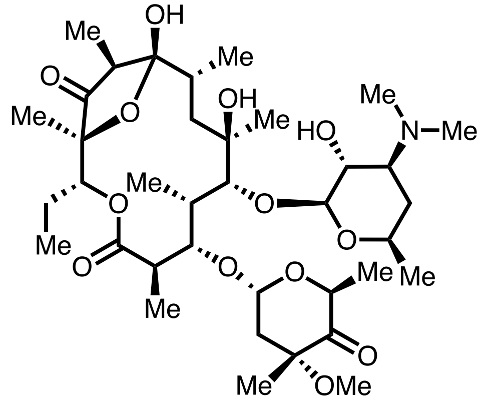

A scintillation vial equipped with a stir bar was charged with C11,C4''-diketo-erythyromycin *N*-oxide **12** (43.5 mg, 0.058 mmol, 1.00 equiv). EtOH (4.40 mL) was added followed by sparging with N_2. Raney Ni® 2800 (0.60 mL) was then added, and the resulting suspension was vigorously stirred at room temperature for 30 min while sparging with N_2. The reaction was then stirred open to air before it was filtered over Celite® rinsing with H_2O and $CHCl_3$:IPA (3:1). Brine was then added and the biphasic mixture was extracted with $CHCl_3$:IPA (3:1, 3x). The combined organic layers were dried over Na_2SO_4 and concentrated via rotary evaporation. The crude white solid was then purified via neutralized SiO_2 column chromatography (2.5–20% MeOH/DCM). C11,C4''-Diketo-erythyromycin **13** was collected as a white powder (19.9 mg, 0.027 mmol, 47% yield).

R_f: 0.70 (20% MeOH/DCM, visualized with $KMnO_4$ stain)

^{1}H NMR (600 MHz, $CDCl_3$) δ 5.13 – 5.06 (m, 2H), 4.50 (d, *J* = 7.7 Hz, 2H), 4.35 (d, *J* = 7.3 Hz, 1H), 4.12 (t, *J* = 8.0 Hz, 1H), 3.67 – 3.61 (m, 1H), 3.51 (s, 1H), 3.41 (dd, *J* = 10.2, 7.4 Hz, 1H), 3.29 – 3.26 (m, 3H), 2.63 (ddd, *J* = 12.1, 10.2, 4.0 Hz, 1H), 2.52 – 2.42 (m, 3H), 2.32 (s, 6H), 2.30 – 2.28 (m, 2H), 1.90 – 1.85 (m, 2H), 1.80 – 1.75 (m, 2H), 1.70 (ddd, *J* = 13.0, 4.2, 2.0 Hz, 1H), 1.41 (d, *J* = 8.0 Hz, 6H), 1.38 (d, *J* = 6.7 Hz, 3H), 1.27 – 1.24 (m, 9H), 1.19 – 1.17 (m, 6H), 1.11 (dd, *J* = 13.0, 8.7 Hz, 1H), 1.06 (d, *J* = 7.0 Hz, 3H), 0.87 – 0.84 (m, 3H). (N.B. The exchangeable protons are not observed.)

^{13}C{^{1}H} NMR (151 MHz, $CDCl_3$) δ 216.2, 211.0, 176.6, 106.8, 106.7, 99.2, 91.4, 83.2, 80.1, 79.8, 77.3, 72.6, 72.2, 70.8, 69.9, 64.2, 51.6, 49.6, 47.8, 42.6, 42.4, 40.8, 38.0, 36.3, 30.5, 24.7, 23.6, 22.8, 21.9, 21.3, 17.6, 17.0, 15.1, 11.3, 10.8, 10.1. (N.B. The methyl groups on the amine are equivalent.)

FTIR (solid) cm^{-1}: 2979, 2940, 2879, 1732, 1459, 1372.

Optical Rotation: $[\alpha]^{20}_D$ -17.0 (*c* = 1.33, DCM)

HRMS (ESI+) calculated for $C_{37}H_{64}NO_{13}$ $[M+H]^+$ 730.4372, found 730.4360.

C9-Erythromycinoximine Acetal *N*-Oxide (**14**)

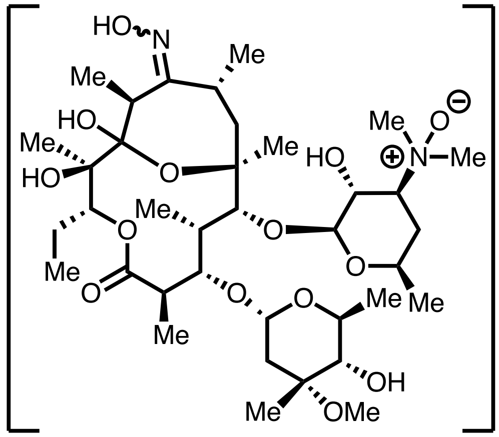

A 25 mL round bottom flask equipped with a stir bar was charged with hydroxylamine hydrochloride (357 mg, 4.77 mmol, 10.0 equiv.) and $MgSO_4$ (357 mg, 100 w/w%). Pyridine (3.00 mL) was added. The resulting suspension was vigorously stirred at room temperature for 10 minutes. C11-Keto-erythromycin *N*-oxide **5'** (357.0 mg, 0.48 mmol, 1.00 equiv) was then added as a solution in pyridine (6.50 mL), and the reaction was heated to 50 ºC and stirred overnight. The reaction was then cooled to room temperature, diluted with $CHCl_3$:IPA (3:1), and washed with H_2O (3x). The organic layer was dried over Na_2SO_4 and concentrated under a stream of air as concentration via rotary evaporation led to degradation. The crude yellow solid was purified via reverse phase preparatory HPLC (5–20% H_2O/MeCN over 0–5 min, 20–50% H_2O/MeCN over 5–50 min, 40–100% H_2O/MeCN over 50–54 min, hold 100% MeCN for 54–60 min, 100–5% H_2O/MeCN over 60–62 min, and hold 5% H_2O/MeCN for 62–65 min. Typical elution times ~ 30–33 min). C9-Erythromycinoximine acetal *N*-oxide **14** was collected as a white powder (48.3 mg, 0.063 mmol, 13% yield).

From **Figure 4**, compound **14** was isolated, and the data collected is consistent with an oxime intermediate that contains a hemi-ketal. Full structural elucidation was not undertaken.

R_f: 0.35 (25% MeOH/DCM, visualized with $KMnO_4$ stain)

^{1}H NMR (500 MHz, $CDCl_3$) δ 5.02 (dd, *J* = 10.5, 2.9 Hz, 1H), 4.88 – 4.76 (m, 2H), 4.48 (d, *J* = 7.0 Hz, 1H), 4.13 – 4.03 (m, 1H), 4.00 (dq, *J* = 9.3, 6.2 Hz, 1H), 3.78 – 3.72 (m, 1H), 3.66 – 3.60 (m, 1H), 3.57 (d, *J* = 9.2 Hz, 1H), 3.47 – 3.41 (m, 1H), 3.36 (s, 3H), 3.23 – 3.18 (m, 6H), 3.14 – 3.08 (m, 1H), 2.98 (d, *J* = 9.2 Hz, 1H), 2.57 – 2.52 (m, 1H), 2.43 (d, *J* = 15.4 Hz, 1H), 2.14 (m, 2H), 2.00 – 1.93 (m, 1H), 1.75 (d, *J* = 6.4 Hz, 1H), 1.71 – 1.65 (m, 1H), 1.57 (d, *J* = 14.7 Hz, 1H), 1.52 (dd, *J* = 15.3, 4.8 Hz, 1H), 1.39 – 1.36 (m, 7H), 1.29 (s, 3H), 1.27 – 1.24 (m, 15H), 1.23 – 1.22 (m, 3H), 0.87 (t, *J* = 7.2 Hz, 3H). (N.B. The exchangeable protons are not observed.)

^{13}C{^{1}H} NMR (151 MHz, $CDCl_3$) δ 176.5, 168.9, 109.5, 103.5, 97.1, 83.3, 80.0, 78.0, 77.5, 76.8, 76.4, 76.1, 72.8, 72.5, 67.8, 65.7, 59.1, 52.2, 49.8, 45.8, 45.1, 44.3, 37.2, 35.1, 35.1, 28.9, 28.4, 24.1, 22.1, 21.7, 21.2, 20.8, 18.5, 13.0, 11.7, 11.2, 10.5.

FTIR (solid) cm^{-1}: 2926, 1729, 1455, 1375, 1260.

Optical Rotation: $[\alpha]^{20}_{D}$ -31.18 (*c* = 0.340, MeOH)

HRMS (ESI+) calculated for $C_{37}H_{67}N_2O_{14}$ $[M+H]^+$ 763.4581, found 763.4587.

C9,12-Erythromycin furanone (**15**)

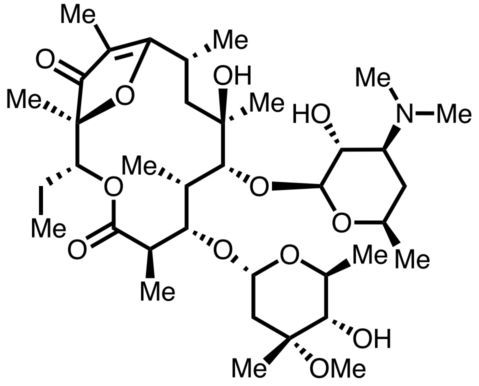

A scintillation vial equipped with a stir bar was charged C9-erythromycinoximine acetal *N*-oxide **14** (35.7 mg, 0.047 mmol, 1.00 equiv). MeOH (4.40 mL) was added followed by sparging with N_2. Raney Ni® 2800 (0.50 mL) was then added, and the resulting suspension was vigorously stirred at room temperature for 30 min while sparging with N_2. The reaction was then sparged with H_2 and stirred under a balloon of H_2 overnight before it was stirred open to air and filtered over Celite® rinsing with H_2O and $CHCl_3$:IPA (3:1). Brine was then added and the biphasic mixture was extracted with $CHCl_3$:IPA (3:1, 3x). The combined organic layers were dried over Na_2SO_4 and concentrated via rotary evaporation. The crude white solid was then purified via neutralized SiO_2 column chromatography (5–30% MeOH/DCM). C9,12-Erythromycin furanone **15** was collected as a white powder (8.3 mg, 0.012 mmol, 25% yield).

Structural assignment is confirmed by X-ray crystallography (Section 12).

R_f: 0.33 (30% MeOH/DCM, visualized with $KMnO_4$ stain)

^{1}H NMR (800 MHz, $CDCl_3$) δ 5.02 (dd, *J* = 10.7, 3.2 Hz, 1H), 4.80 (dd, *J* = 4.9, 1.9 Hz, 1H), 4.53 (d, *J* = 7.3 Hz, 1H), 4.11 (dq, *J* = 8.8, 6.4 Hz, 1H), 4.00 (dd, *J* = 6.2, 2.8 Hz, 1H), 3.72 (d, *J* = 4.5 Hz, 1H), 3.70 (ddd, *J* = 11.1, 6.1, 1.9 Hz, 1H), 3.47 (dd, *J* = 10.3, 7.3 Hz, 1H), 3.27 (s, 3H), 3.06 (d, *J* = 8.8 Hz, 1H), 2.99 – 2.94 (m, 2H), 2.56 (s, 6H), 2.48 – 2.45 (m, 1H), 2.33 (dd, *J* = 15.0, 1.9 Hz, 1H), 2.14 (dd, *J* = 15.0, 3.4 Hz, 1H), 2.02 (ddq, *J* = 14.7, 10.7, 7.4 Hz, 1H), 1.95 (ddd, *J* = 12.8, 4.2, 2.0 Hz, 1H), 1.84 (dd, *J* = 15.0, 6.4 Hz, 1H), 1.79 (hd, *J* = 7.6, 2.9 Hz, 1H), 1.74 (s, 3H), 1.67 – 1.63 (m, 1H), 1.57 (dd, *J* = 15.0, 4.7 Hz, 1H), 1.40 (td, *J* = 12.7, 11.0 Hz, 1H), 1.37 – 1.35 (m, 6H), 1.30 – 1.27 (m, 6H), 1.24 (d, *J* = 3.6 Hz, 6H), 1.18 (d, *J* = 7.0 Hz, 3H), 1.03 (d, *J* = 7.3 Hz, 3H), 0.89 (t, *J* = 7.4 Hz, 3H). (N.B. The exchangeable protons are not observed.)

^{13}C{^{1}H} NMR (201 MHz, $CDCl_3$) δ 205.1, 193.0, 176.0, 108.7, 104.9, 96.8, 87.8, 87.4, 79.0, 78.1, 77.4, 74.8, 73.0, 70.6, 69.4, 66.8, 64.5, 49.4, 46.6, 43.1, 41.9, 40.3, 35.3, 31.8, 31.1, 26.5, 21.6, 21.5, 21.4, 21.0, 20.7, 17.8, 14.4, 10.9, 10.9, 6.1. (N.B. The methyl groups on the amine are equivalent.)

FTIR (solid) cm^{-1}: 3420, 2973, 2934, 1738, 1697, 1619, 1458, 1378.

Optical Rotation: $[\alpha]^{20}_D$ -38.6 (*c* = 0.553, MeOH)

HRMS (ESI+) calculated for $C_{37}H_{64}NO_{12}$ $[M+H]^+$ 714.4423, found 714.4395.

4. Synthesis of Clarithromycin Derivatives

Clarithromycin *N*-Oxide (**7**)

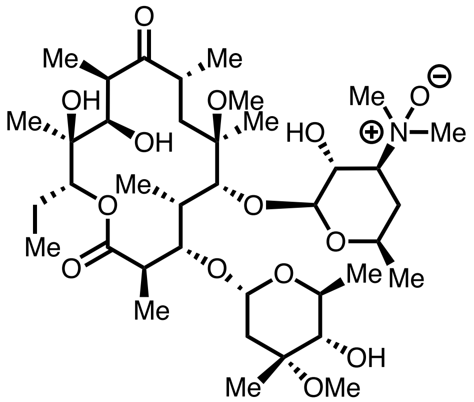

A 25 mL flask equipped with a stir bar was charged with clarithromycin **6** (374 mg, 0.50 mmol, 1.00 equiv.). DCM (10.0 mL) was added followed by *m*CPBA (240. mg, 1.00 mmol, 2.00 equiv.). The resulting suspension was vigorously stirred at room temperature for 30 minutes. The reaction was quenched by addition of $Na_2SO_{3(aq)}$ (saturated) upon complete consumption of **6** as determined by UPLC/MS then stirred for 1 hour. The reaction was then diluted with $CHCl_3$:IPA (3:1) and washed with $NaHCO_{3(aq)}$ (saturated, 3x). The organic layer was dried over Na_2SO_4 and concentrated via rotary evaporation. The crude white solid was then purified via SiO_2 column chromatography (20–25% MeOH/DCM). Clarithromycin *N*-oxide **7** was collected as a white powder (347 mg, 0.454 mmol, 91% yield).

R_f: 0.34 (10% MeOH/DCM, visualized with $KMnO_4$ stain)

^{1}H NMR (400 MHz, $CDCl_3$) δ 5.04 (dd, *J* = 11.1, 2.3 Hz, 1H), 4.92 (d, *J* = 4.7 Hz, 1H), 4.54 (d, *J* = 7.0 Hz, 1H), 4.12 – 3.96 (m, 1H), 3.79 – 3.70 (m, 3H), 3.67 (d, *J* = 7.2 Hz, 1H), 3.62 (ddd, *J* = 10.7, 6.0, 1.8 Hz, 1H), 3.36 (s, 3H), 3.35 – 3.29 (m, 1H), 3.20 (s, 6H), 3.16 (s, 1H), 3.07 – 2.96 (m, 5H), 2.95 – 2.81 (m, 1H), 2.66 – 2.50 (m, 1H), 2.40 – 2.35 (m, 1H), 2.02 – 1.83 (m, 4H), 1.71 (dd, *J* = 14.8, 2.0 Hz, 1H), 1.58 (dd, *J* = 15.1, 4.9 Hz, 1H), 1.47 (dtd, *J* = 14.2, 7.2, 3.9 Hz, 1H), 1.39 (s, 3H), 1.35 – 1.23 (m, 10H), 1.20 (d, *J* = 7.2 Hz, 3H), 1.16 – 1.09 (m, 11H), 0.84 (t, *J* = 7.4 Hz, 3H).

^{13}C{^{1}H} NMR (151 MHz, $CDCl_3$) δ 221.2, 176.0, 102.7, 96.2, 81.4, 78.7, 78.5, 78.0, 76.8, 76.5, 74.4, 72.9, 72.8, 69.2, 67.1, 65.9, 59.2, 52.2, 50.8, 49.8, 45.5, 45.2, 39.5, 39.4, 37.4, 35.1, 35.1, 21.7, 21.4, 21.2, 20.0, 18.9, 18.2, 16.2, 16.1, 12.5, 10.8, 9.1.

FTIR (solid) cm^{-1}: 3440, 2972, 2940, 1731, 1690, 1460, 1376.

Optical Rotation: $[\alpha]^{20}_D$ -87.8 (*c* = 0.687, MeOH)

HRMS (ESI+) calculated for $C_{38}H_{70}NO_{14}$ $[M+H]^+$ 764.4791, found 764.4785.

Full assignment of the ^{1}H and ^{13}C NMR of **7** were undertaken to support the structural assignment of **8** using a combination of ^{1}H, ^{13}C, COSY, HSQC, and HMBC NMR techniques. Each H and C were assigned to the structure (**Figure S9**). Full spectra are included in Section 11 and X-ray crystallography data is included in Section 12 (*vide infra*).

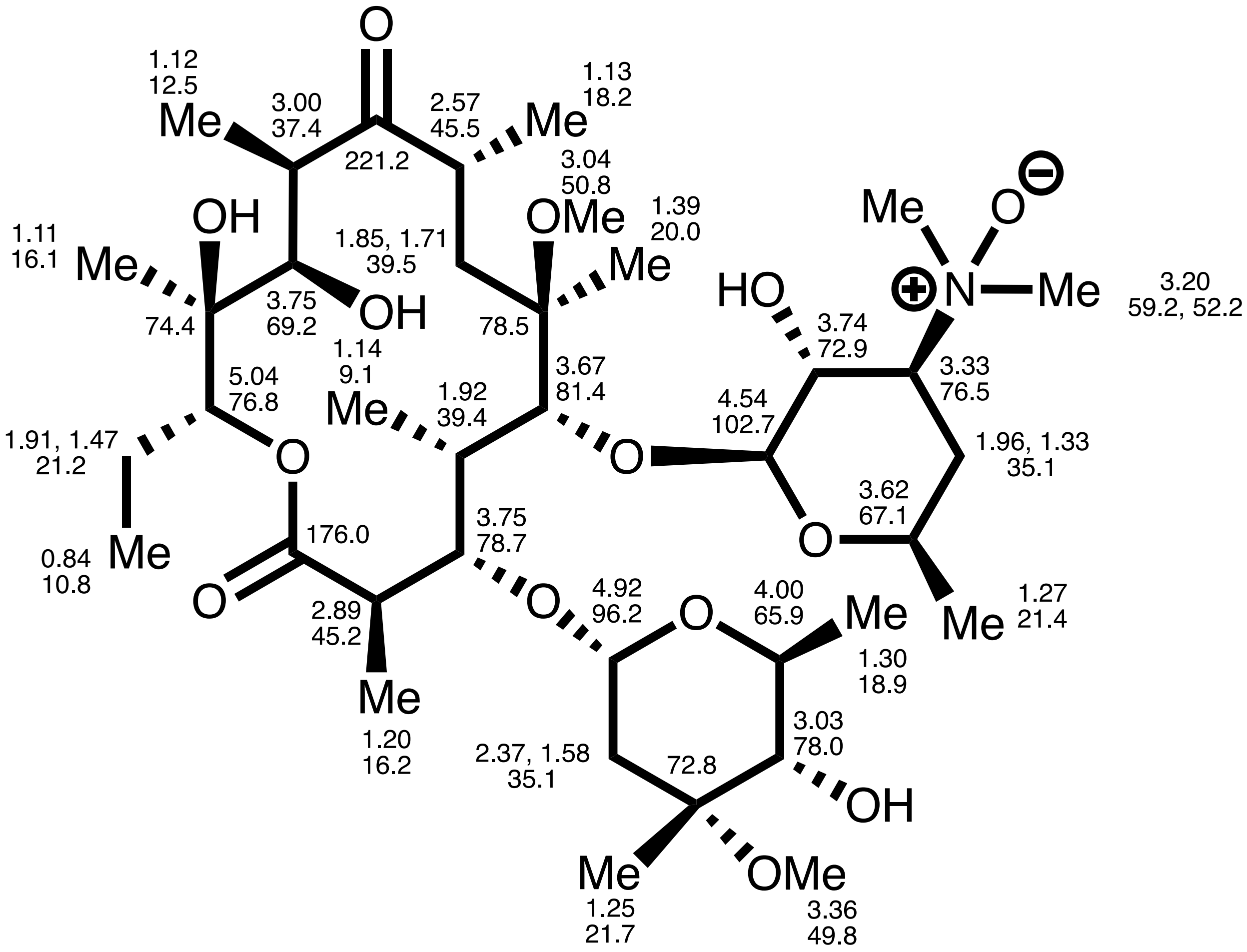

Figure S9. ^{1}H (top) and ^{13}C (top) assignments for clarithromycin *N*-oxide **7** in $CDCl_3$.

C4''-Keto-Clarithromycin *N*-Oxide (**8**)

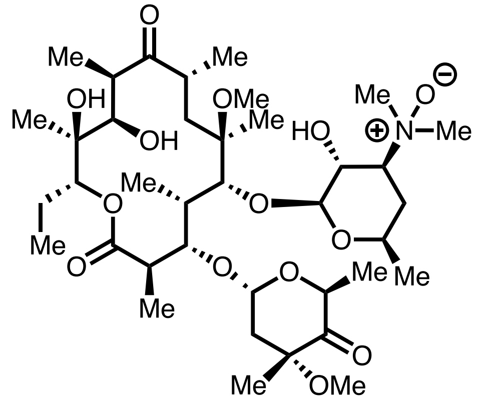

A 25 mL flask equipped with a stir bar was charged with clarithromycin **6** (374 mg, 0.50 mmol, 1.00 equiv.). DCM (10.0 mL) was added followed by *m*CPBA (240. mg, 1.00 mmol, 2.00 equiv.). The resulting suspension was vigorously stirred at room temperature for 30 minutes. The reaction was quenched by addition of excess DMS upon complete consumption of **6** as determined by UPLC/MS then stirred for 1 hour. The reaction was then diluted with $CHCl_3$:IPA (3:1) and washed with $NaHCO_{3(aq)}$ (saturated, 3x). The organic layer was dried over Na_2SO_4 and concentrated via rotary evaporation, and crude clarithromycin *N*-oxide **7** was carried forward without further purification.

A 25 mL flask equipped with a stir bar was charged with clarithromycin *N*-oxide **7** (assumed 0.50 mmol, 1.00 equiv.). DCM (10.0 mL) was added followed by DMP (424. mg, 1.00 mmol, 2.00 equiv.). The resulting suspension was vigorously stirred at room temperature for 24 hr. The reaction was quenched by addition of excess DMS then stirred for 1 hour. The reaction was then diluted with $CHCl_3$:IPA (3:1) and washed with $NaHCO_{3(aq)}$ (saturated, 3x). The organic layer was dried over Na_2SO_4 and concentrated via rotary evaporation. The crude yellow solid was then purified via reverse phase preparatory HPLC (5–20% H_2O/MeCN over 0–5 min, 20–50% H_2O/MeCN over 5–50 min, 50–100% H_2O/MeCN over 50–54 min, hold 100% MeCN for 54–55 min, 100–5% H_2O/MeCN over 55–58 min, and hold 5% H_2O/MeCN for 58–60 min. Typical elution times ~ 45.5–47.5 min). C4''-Keto-clarithromycin *N*-oxide **8** was collected as a white powder (99.2 mg, 0.13 mmol, 26% yield over 2 steps).

R_f: 0.34 (10% MeOH/DCM, visualized with $KMnO_4$ stain)

^{1}H NMR (600 MHz, $CDCl_3$) δ 5.17 (dd, J = 7.9, 6.0 Hz, 1H), 5.04 (dd, J = 11.2, 2.3 Hz, 1H), 4.42 – 4.37 (m, 1H), 4.36 (d, J = 7.1 Hz, 1H), 3.75 (dd, J = 10.0, 1.5 Hz, 1H), 3.72 – 3.67 (m, 2H), 3.65 (d, J = 6.1 Hz, 1H), 3.44 (dqd, J = 12.2, 6.1, 1.8 Hz, 1H), 3.34 – 3.29 (m, 1H), 3.26 (s, 3H), 3.14 (d, J = 6.0 Hz, 6H), 2.98 (s, 3H), 2.97 – 2.93 (m, 1H), 2.89 (dq, J = 9.9, 7.2 Hz, 1H), 2.51 (dtd, J = 14.1, 7.1, 1.8 Hz, 1H), 2.30 (dd, J = 14.4, 6.0 Hz, 1H), 2.23 (dd, J = 14.4, 7.9 Hz, 1H), 1.96 (s, 1H), 1.92 – 1.84 (m, 2H), 1.81 (dd, J = 14.9, 11.8 Hz, 1H), 1.63 (dd, J = 15.0, 1.9 Hz, 1H), 1.44 (ddd, J = 14.3, 11.1, 7.2 Hz, 1H), 1.37 (s, 3H), 1.35 (d, J = 6.8 Hz, 3H), 1.31 (s, 3H), 1.29 – 1.26 (m, 1H), 1.21 (d, J = 6.0 Hz, 3H), 1.18 (d, J = 7.2 Hz, 3H), 1.09 – 1.05 (m, 12H), 0.79 (t, J = 7.4 Hz, 3H). (N.B. The exchangeable protons are not observed.)

$^{13}C\{^{1}H\}$ NMR (151 MHz, $CDCl_3$) δ 221.0, 211.2, 175.5, 102.7, 96.6, 81.1, 78.3, 78.2, 77.1, 76.8, 75.9, 74.3, 72.6, 72.3, 69.2, 67.3, 59.0, 52.0, 51.3, 50.6, 45.4, 44.6, 39.2, 38.3, 37.5, 37.2, 34.8, 21.3, 21.1, 20.6, 19.6, 18.1, 16.5, 16.0, 15.7, 12.4, 10.6, 9.0.

FTIR (solid) cm^{-1}: 2977, 2938, 1734, 1461, 1376, 1346.

Optical Rotation: $[\alpha]^{20}_{D}$ -70.8 (c = 0.373, MeOH)

HRMS (ESI+) calculated for $C_{38}H_{68}NO_{14}$ $[M+H]^+$ 762.4634, found 762.4617.

Full assignment of the ^{1}H and ^{13}C NMR were undertaken to support the structural assignment of **8** using a combination of ^{1}H, ^{13}C, COSY, HSQC, and HMBC NMR techniques. Each H and C were assigned to the structure (**Figure S10**). Full spectra are included in Section 11 (*vide infra*).

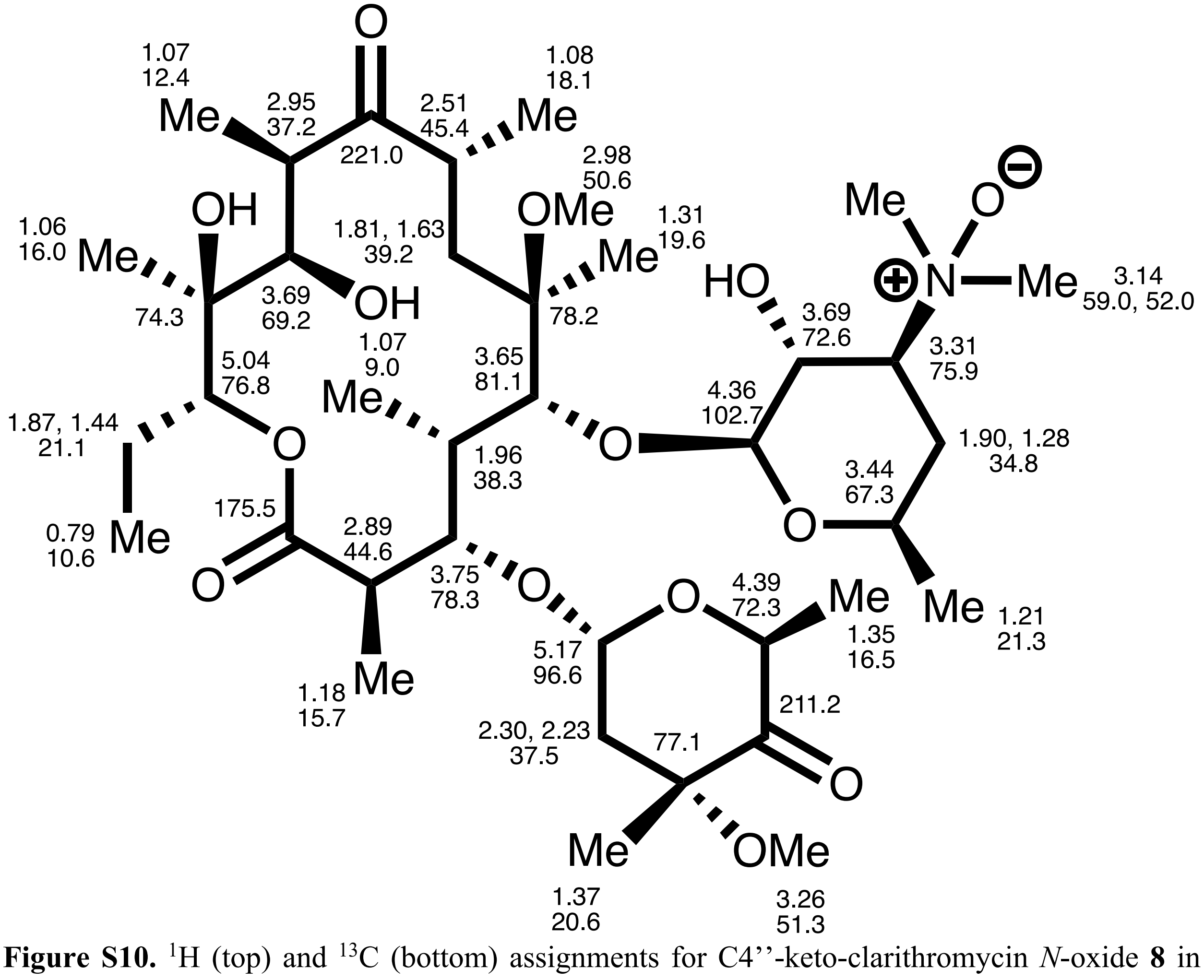

Figure S10. ^{1}H (top) and ^{13}C (bottom) assignments for C4''-keto-clarithromycin *N*-oxide **8** in $CDCl_3$.

HMBC correlations can be seen between the newly installed C4'' ketone (211.2) and five ^{1}H shifts of the cladinose sugar (4.39, 2.30, 2.23, 1.37, and 1.35) (**Figure S11**). Additionally, overlay of the HSQC spectra for **7** (green) and **8** (red) demonstrates the preservation of the sp^3 C11 (**7**: 3.75, 69.1;

8: 3.69, 69.2) and C2' (**7**: 3.74, 72.9; **8**: 3.69, 72.6) carbons based on the observed connectivity of the ^{1}H signal (**Figure S12**). The C4'' proton (3.03) is no longer observed in **8**, supporting the assertion that oxidation has taken place at that site. Many ^{1}H shifts of the rest of the cladinose sugar can also be seen to shift significantly between **7** and **8** (e.g. **7** vs **8**: 4.92 vs 5.17; 4.00 vs 4.39; 1.58 vs 2.23) (**Figure S13**).

Additionally, UPLC/MS shows that the fragmentation mass resulting from the loss of the cladinose sugar (-158) loses evidence of oxidation (-2) when compared with **7**. This indicates that oxidation takes place on cladinose. The relevant masses observed in the ESI^{+} for **7** are 764 and 606 vs for **8** at 762 and 606 (**Figure S14)**.

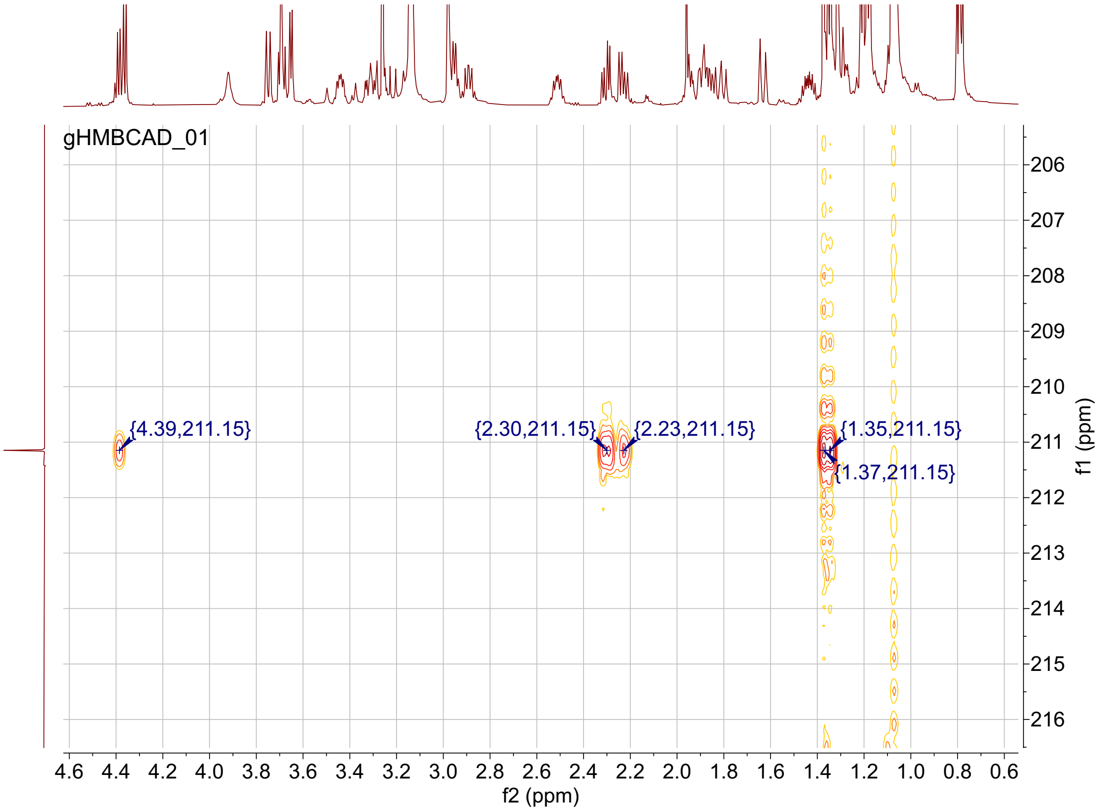

Figure S11. HMBC of **8** in $CDCl_3$ highlighting correlations between the new carbonyl C (211.2) and key shifts on the cladinose sugar (4.39, 2.30, 2.23, 1.37, and 1.35).

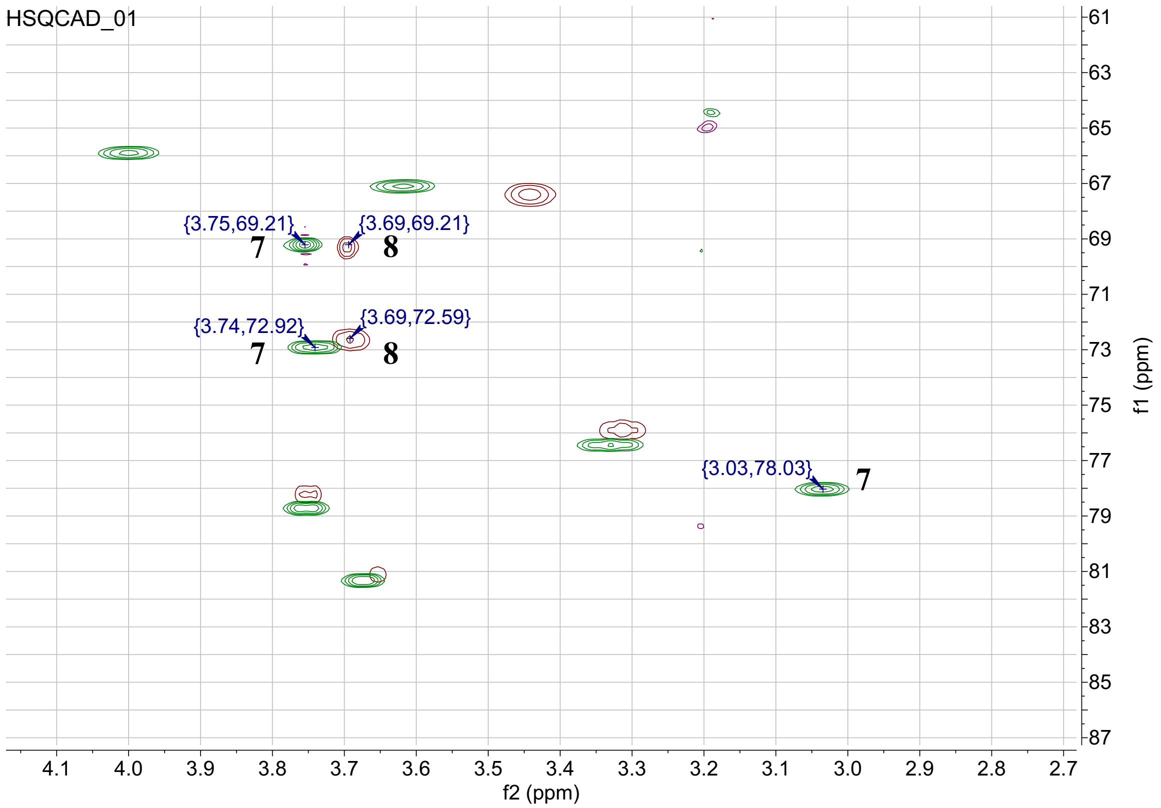

Figure S12. Overlay of HSQC in $CDCl_3$ for **7** (green) and **8** (red) highlighting loss of the C4'' proton of **8**.

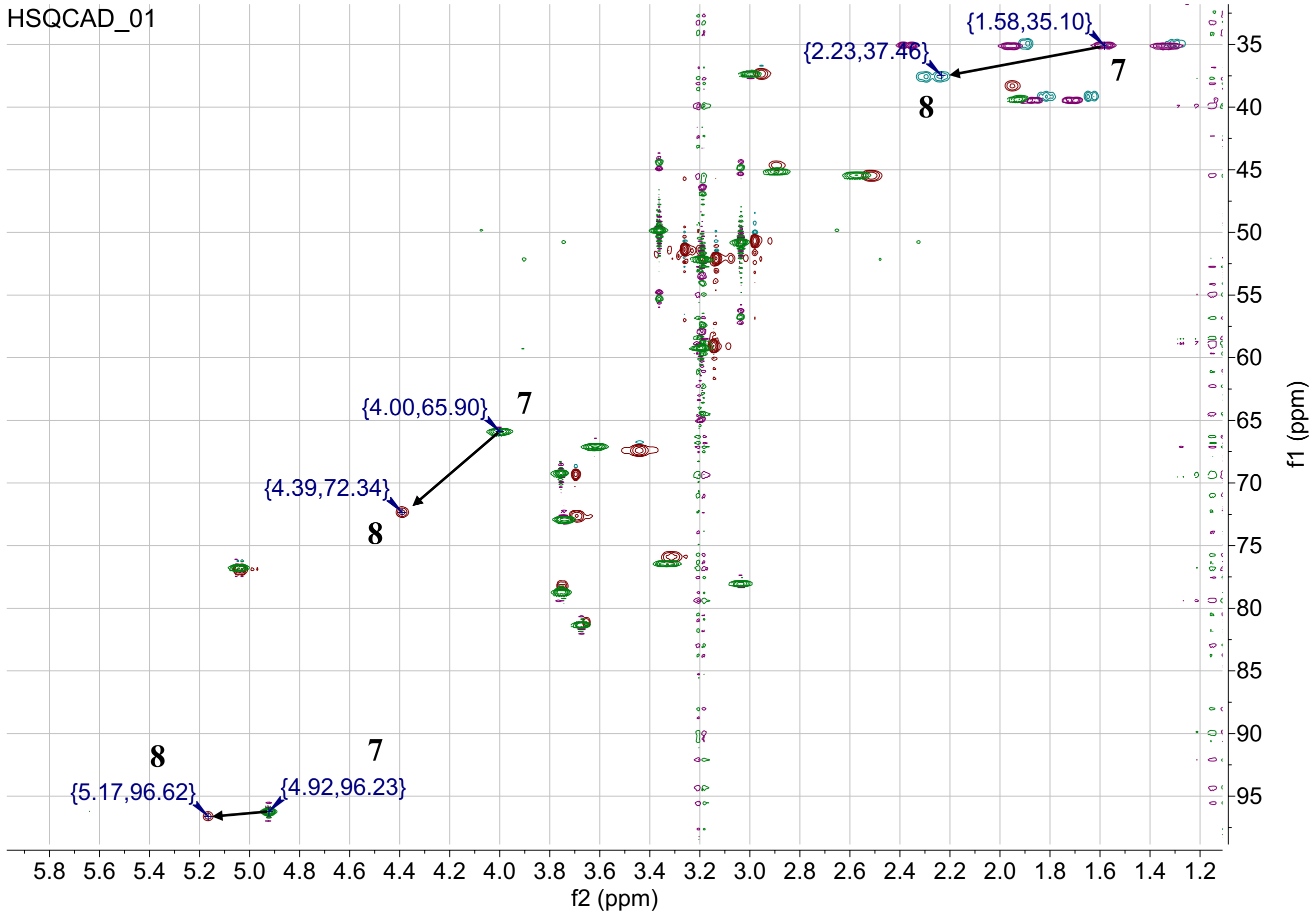

Figure S13. Overlay of HSQC in $CDCl_3$ for **7** (green) and **8** (red) highlighting major shifts in the peaks associated with the cladinose sugar after oxidation.

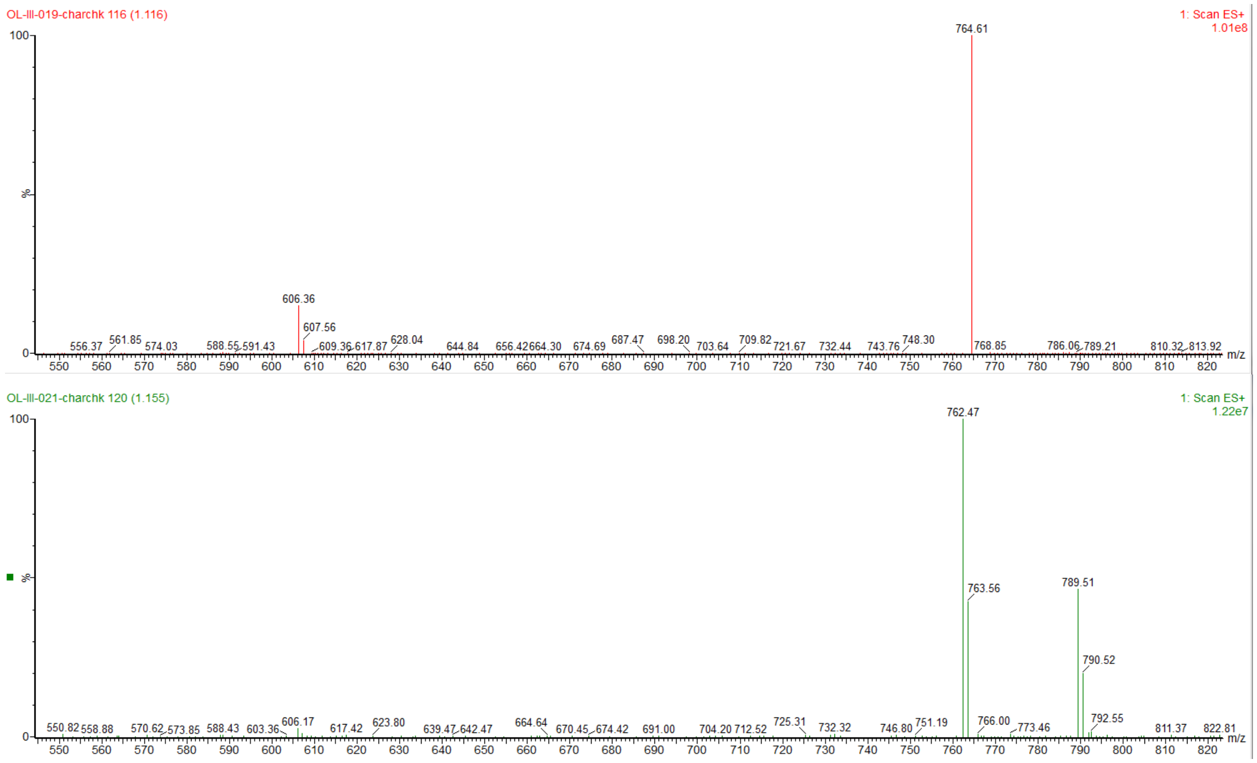

Figure S14. UPLC/MS trace of **7** (top) and **8** (bottom) showing key fragmentation.

C4''-Keto-Clarithromycin (**16**)

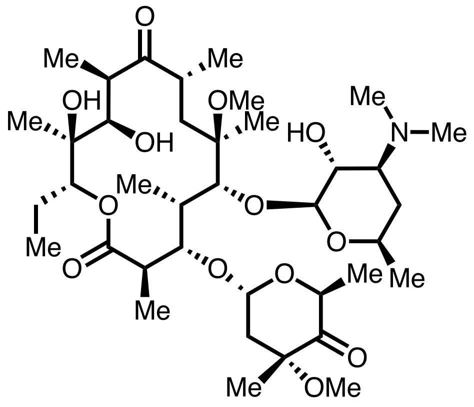

A scintillation vial equipped with a stir bar was charged with C4''-keto-clarithromycin *N*-oxide **8** (26.3 mg, 0.031 mmol, 1.00 equiv). MeOH (2.60 mL) was added followed by sparging with N_2. Raney Ni® 2800 (0.30 mL) was then added, and the resulting suspension was vigorously stirred at room temperature for 30 min while sparging with N_2. The reaction was stirred open to air before it was filtered over Celite®, rinsing with H_2O and $CHCl_3$:IPA (3:1). Brine was then added and the biphasic mixture was extracted with $CHCl_3$:IPA (3:1, 3x). The combined organic layers were dried over Na_2SO_4 and concentrated via rotary evaporation. The crude white solid was then purified via neutralized SiO_2 column chromatography (2.5–15% MeOH/DCM). C4''-Keto-clarithromycin **16** was collected as a white powder (6.0 mg, 0.0080 mmol, 26% yield).

R_f: 0.81 (20% MeOH/DCM, visualized with $KMnO_4$ stain)

^{1}H NMR (600 MHz, $CDCl_3$) δ 5.25 – 5.21 (m, 1H), 5.12 – 5.08 (m, 1H), 4.47 (q, *J* = 6.8 Hz, 1H), 4.28 (dd, *J* = 7.2, 1.2 Hz, 1H), 3.83 (d, *J* = 10.2 Hz, 1H), 3.75 (d, *J* = 1.7 Hz, 1H), 3.69 (d, *J* = 6.2 Hz, 1H), 3.40 – 3.34 (m, 1H), 3.31 (d, *J* = 1.0 Hz, 3H), 3.21 – 3.16 (m, 2H), 3.03 (d, *J* = 1.1 Hz, 3H), 2.96 – 2.89 (m, 1H), 2.61 – 2.53 (m, 1H), 2.46 – 2.40 (m, 1H), 2.35 (dd, *J* = 14.3, 6.1 Hz, 1H), 2.27 – 2.26 (m, 6H), 2.02 – 1.99 (m, 1H), 1.96 – 1.90 (m, 1H), 1.84 (dd, *J* = 14.8, 11.8 Hz, 1H), 1.68 (dd, *J* = 14.9, 1.7 Hz, 1H), 1.67 – 1.63 (m, 1H), 1.51 – 1.47 (m, 1H), 1.43 (s, 3H), 1.40 (dd, *J* = 6.8, 1.0 Hz, 3H), 1.39 (s, 3H), 1.25 – 1.20 (m, 9H), 1.14 – 1.12 (m, 8H), 1.10 – 1.09 (m, 3H), 0.85 (t, *J* = 7.4 Hz, 3H). (N.B. The exchangeable protons are not observed.)

^{13}C{^{1}H} NMR (151 MHz, $CDCl_3$) δ 221.2, 211.4, 175.6, 103.6, 96.9, 81.3, 78.5, 78.3, 77.2, 77.0, 74.4, 72.5, 71.0, 69.4, 69.3, 65.4, 51.4, 50.7, 45.5, 44.8, 40.4, 39.4, 38.4, 37.3, 37.2, 28.7, 21.6, 21.2, 20.8, 19.7, 18.2, 16.7, 16.1, 15.8, 12.5, 10.7, 9.2. (N.B. The methyl groups on the amine are equivalent.)

FTIR (solid) cm^{-1}: 2976, 2938, 1732, 1690, 1458, 1378.

Optical Rotation: $[\alpha]^{20}_D$ -109.7 (*c* = 0.46, DCM)

HRMS (ESI+) calculated for $C_{38}H_{68}NO_{13}$ $[M+H]^+$ 746.4685, found 746.4668.

5. Synthesis of Azithromycin Analog

C4''-Keto-Azithromycin (**10**)

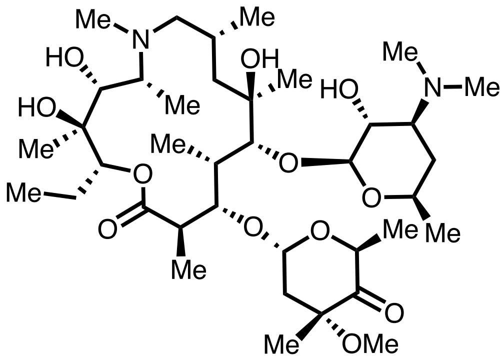

A 25 mL flask equipped with a stir bar was charged with azithromycin **9** (375 mg, 0.50 mmol, 1.00 equiv). DCM (10.0 mL) was added followed by DMP (848 mg, 2.00 mmol, 4.00 equiv). The resulting suspension was vigorously stirred at room temperature for 24 hr. The reaction was then diluted with DCM and washed with $Na_2CO_{3(aq)}$ (saturated, 1x). The organic layer was dried concentrated via rotary evaporation then brought up in citric acid (10% aq w/v) before being washed with Et_2O (3x). The aqueous layer was then basified using 4M NaOH and extracted with DCM (3x). The combined organic layers were dried over Na_2SO_4 and concentrated via rotary evaporation. The crude yellow solid was then purified via SiO_2 column chromatography (1% + 7% + 92% Et_3N/MeOH/DCM). C4''-Keto-azithromycin **10** was collected as a white powder (34.0 mg, 0.045 mmol, 9.1% yield).[10] (N.B. Compound streaks heavily. Only analytically pure material was isolated contributing to the low yield.)

R_f: 0.07 (20% MeOH/DCM, visualized with $KMnO_4$ stain)

^{1}H NMR (600 MHz, $CDCl_3$) δ 5.33 (t, *J* = 7.1 Hz, 1H), 4.67 (d, *J* = 10.5 Hz, 1H), 4.58 (q, *J* = 6.8 Hz, 1H), 4.32 (dd, *J* = 7.2, 1.4 Hz, 1H), 4.28 (dd, *J* = 7.5, 1.9 Hz, 1H), 3.66 (d, *J* = 5.9 Hz, 1H), 3.64 (s, 1H), 3.41 – 3.34 (m, 1H), 3.31 (d, *J* = 1.1 Hz, 3H), 3.26 – 3.22 (m, 1H), 2.93 – 2.86 (m, 1H), 2.74 – 2.70 (m, 1H), 2.50 (dd, *J* = 11.8, 2.5 Hz, 1H), 2.47 – 2.42 (m, 1H), 2.35 (d, *J* = 1.5 Hz, 3H), 2.33 (d, *J* = 6.1 Hz, 1H), 2.29 (d, *J* = 7.9 Hz, 1H), 2.27 (s, 6H), 2.12 – 2.04 (m, 2H), 1.96 (d, *J* = 8.4 Hz, 1H), 1.92 – 1.87 (m, 1H), 1.79 (d, *J* = 14.4 Hz, 1H), 1.66 – 1.63 (m, 1H), 1.54 – 1.50 (m, 1H), 1.44 (s, 3H), 1.43 (dd, *J* = 6.7, 1.1 Hz, 3H), 1.32 – 1.30 (m, 4H), 1.25 – 1.24 (m, 4H), 1.22 (d, *J* = 4.9 Hz, 3H), 1.11 – 1.09 (m, 3H), 1.07 (d, *J* = 1.2 Hz, 3H), 1.06 – 1.04 (m, 3H), 0.92 – 0.89 (m, 6H). (N.B. The exchangeable protons are not observed.)

^{13}C{^{1}H} NMR (151 MHz, $CDCl_3$) δ 212.0, 178.5, 103.9, 96.5, 84.9, 78.9, 78.0, 77.4, 75.2, 74.5, 73.7, 72.5, 71.1, 70.4, 69.4, 65.5, 62.2, 51.4, 44.7, 42.3, 40.5, 39.9, 36.8, 36.8, 29.2, 27.1, 26.8, 22.0, 21.5, 21.2, 21.1, 16.5, 16.3, 15.5, 11.3, 9.4, 7.7.

FTIR (solid) cm^{-1}: 2970, 2932, 1732, 1458, 1378, 1257.

Optical Rotation: $[\alpha]^{20}_D$ -6.59 (*c* = 0.273, MeOH)

HRMS (ESI+) calculated for $C_{38}H_{68}NO_{14}$ $[M+H]^+$ 747.5002, found 747.4988.

Full assignment of the ^1H and ^{13}C NMR were undertaken to support the structural assignment of **10** using a combination of ^1H, ^{13}C, COSY, HSQC, and HMBC NMR techniques. Each H and C were assigned to the structure (**Figure S15**). Full spectra are included in Section 11 (*vide infra*).

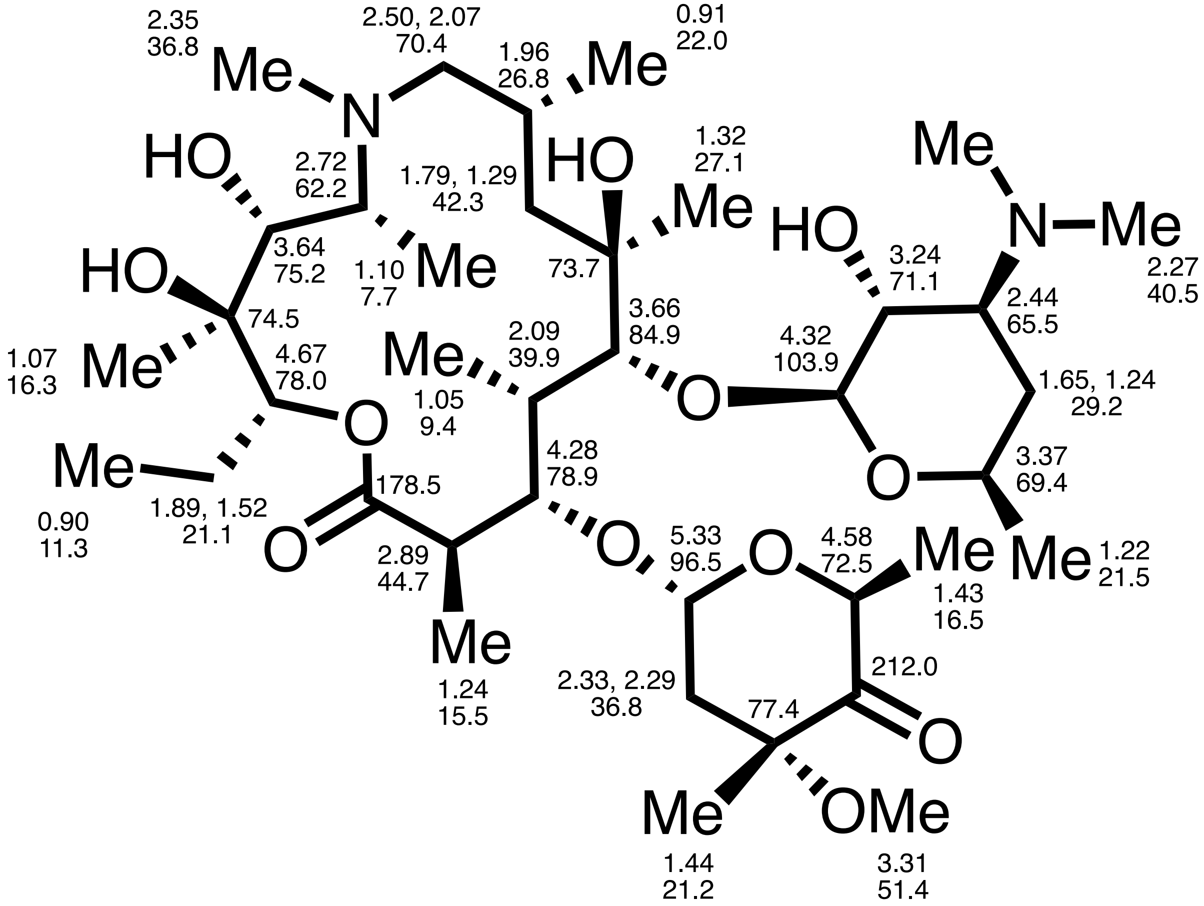

Figure S15. ^1H (top) and ^{13}C (bottom) assignments for C4''-keto-azithromycin **10** in $CDCl_3$.

HMBC correlations can be seen between the newly installed C4'' ketone (211.3) and five ^1H shifts of the cladinose sugar (4.58, 2.33, 2.29, 1.44 and 1.43) (**Figure S16**). Additionally, an overlay of the HSQC spectra for **10** (red) and **8** (green) demonstrates that many of ^1H shifts of the cladinose sugar are consistent with those observed in the monooxidation of clarithromycin to form C4''-keto-clarithromycin (**8**, *vide supra*) (e.g. **10** vs **8**: 5.33 vs 5.17; 4.58 vs 4.39; 2.33 and 2.29 vs 2.3 and 2.23) (**Figure 17**). The carbonyl carbon also has a very similar shift (**10**: 212.0 vs **8**: 211.2).

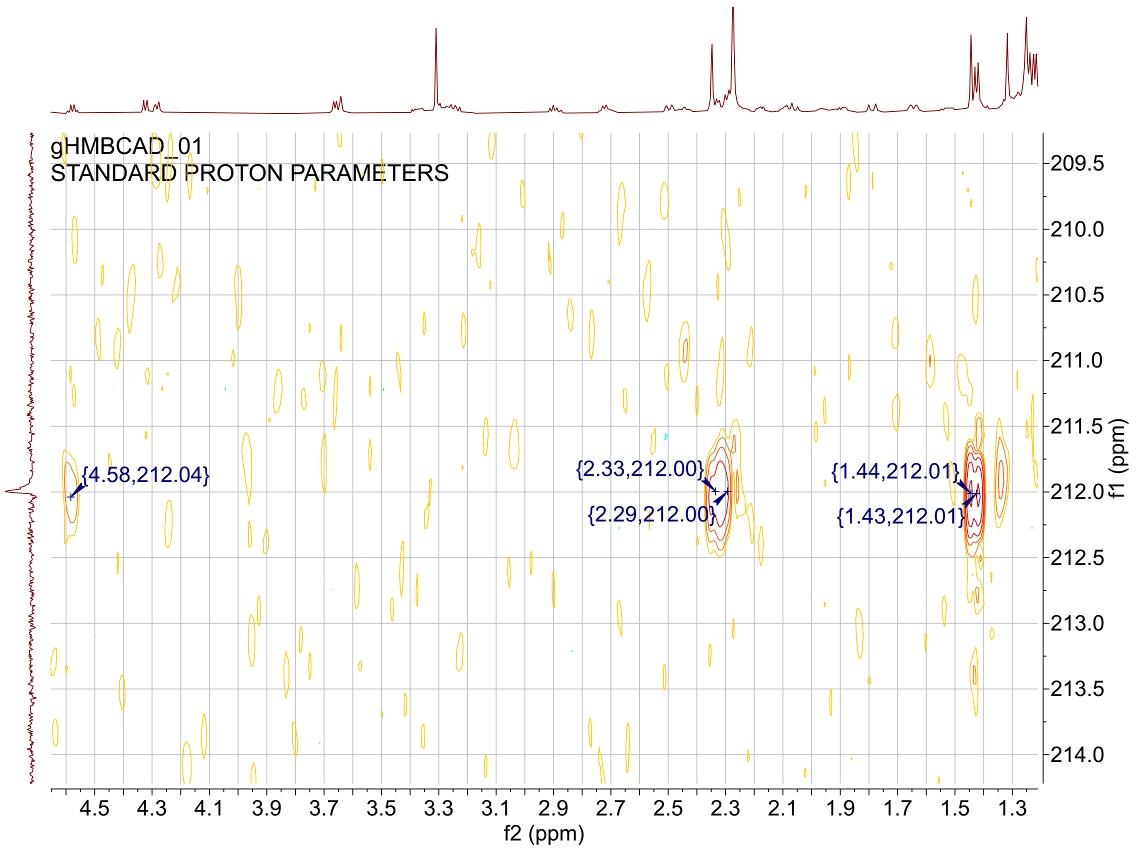

Figure S16. HMBC of **10** in $CDCl_3$ highlighting correlations between the new carbonyl C (212.0) and key shifts on the cladinose sugar (4.58, 2.33, 2.29, 1.44, and 1.43).

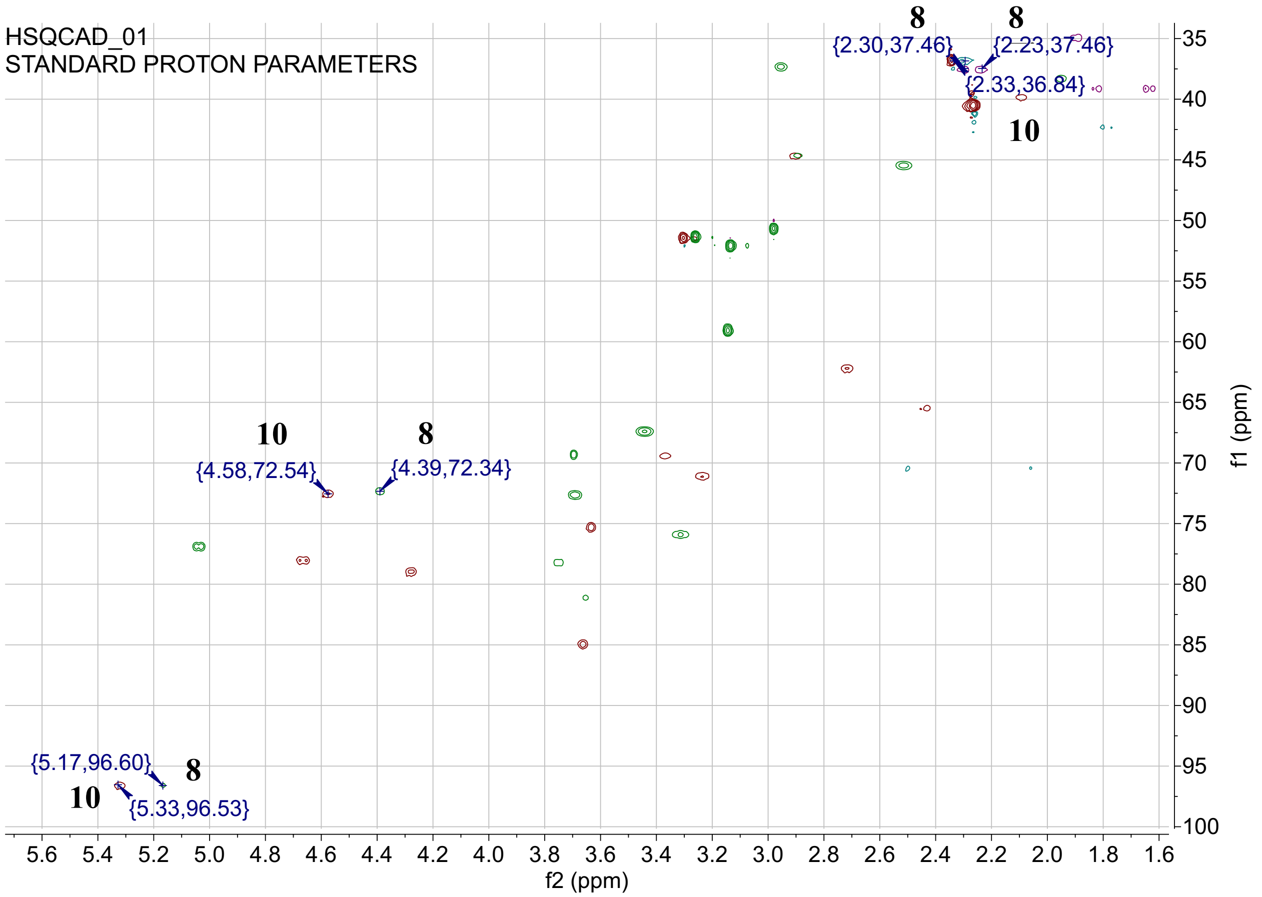

Figure S17. Overlay of HSQC in $CDCl_3$ for **8** (green) and **10** (red) highlighting the consistency of key shifts on the cladinose sugar.

6. Reaction Ratio Determination

Multiple efforts were made during reaction optimization to identify a suitable internal standard. However, it was found that, in our hands, every standard screened led to irreproducible results even when weighed out with a known amount of pure erythromycin A. Given this observation, we report results as a ratio of **2** to **5'**. This does not account for minor degradation products present but does represent the major species observed in each reaction mixture. Integrations were taken using the peaks present at 5.10 ppm and 4.84 ppm respectively in DMSO-d_6 and were found to be consistent and reproducible. It was found that some peaks were shifted in $CDCl_3$ dependent on the amount of residual *m*CBA in each sample. This effect was not observed in more polar solvents leading to their selection for ratio determination.

7. Reaction Optimization

Oxidant Screen

General Procedure used for oxidant screening:

For entries 1–15, the following procedure was used unless otherwise noted: A 1-dram vial equipped with a stir bar was charged with $NaHCO_3$ (21.0 mg, 0.25 mmol, 5.00 equiv.), ACT (2.1 mg, 0.010 mmol, 0.20 equiv.), and **Oxidant**. DCM (1.00 mL) was added followed by erythromycin **1** (36.7 mg, 0.050 mmol, 1.00 equiv.). The resulting suspension was stirred vigorously and monitored with UPLC/MS for **Time**.

N.B. For entries 16 and 17, the following procedure was used: A 1-dram vial equipped with a stir bar was charged with erythromycin **1** (36.7 mg, 0.050 mmol, 1.00 equiv.). DCM (0.50 mL) and DMSO (7.8 µL, 0.11 mmol, 2.20 equiv. or 23.7 µL, 0.33 mmol, 6.60 equiv.) were added, and the solution was cooled to -78 ºC. Oxalyl chloride (4.7 µL, 0.055 mmol, 1.10 equiv. or 14.2 µL, 0.165 mmol, 3.30 equiv.) was then added. The solution was stirred at -78 ºC, and aliquots were taken and quenched with Et_3N to monitor by UPLC/MS.

Table S1. Oxidant Screen.

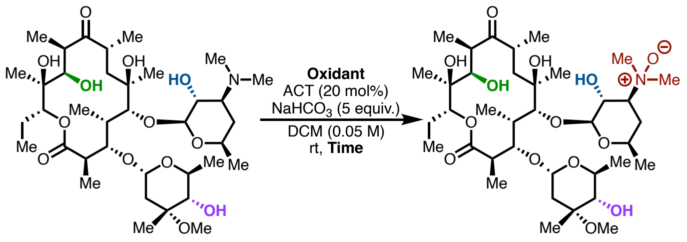

1 2

Entry	Oxidant	Equiv. Oxidant	Time	Results[d]
1[a]	TCCA	2[c]	3 hr	multiple chlorination products
2[a]	PIDA	2	3 hr	multiple degradation products
3[a]	PIFA	2	3 hr	multiple degradation products
4[a]	$NOBF_4$	2	3 hr	multiple degradation products
5[a]	^{t}BuOCl	2	3 hr	multiple degradation products
6[a]	CAN	2	3 hr	clean **1**
7	CAN	2	24 hr	multiple degradation products
8	*m*CPBA	3	2 hr	**2**
9	*m*CPBA	2	24 hr	**2** and minor Baeyer-Villiger oxidation
10	MMPP	3	2 hr	**1** and **2**
11	MMPP	3	24 hr	**1** and **2**
12	H_2O_2 (30%)	3	2 hr	**1** and **2**
13	H_2O_2 (30%)	3	24 hr	**1**, **2**, and multiple degradation products
14	UHP	3	2 hr	clean **1**
15	DMP[b]	1	2 hr	multiple oxidations
16	$(COCl)_2$/DMSO[b]	1.1	24 hr	no reaction
17	$(COCl)_2$/DMSO[b]	3.3	24 hr	multiple degradation products

[a] Also run with preformed **2**, and only degradation was observed. [b] Run without ACT. [c] Each equivalent of TCCA can turn over ACT a possible 3 times. [d] Monitored by UPLC-MS.

Base Screen

General Procedure used for base screening:

A 1-dram vial equipped with a stir bar was charged with erythromycin **1** (36.7 g, 0.050 mmol, 1.00 equiv.) and **Base** (0.50 mmol, 10.0 equiv.). DCM (1.00 mL) was added followed by *m*CPBA (119.9 mg, 0.50 mmol, 10.0 equiv.). The resulting suspension was vigorously stirred at room temperature for 30 minutes before the addition of HAzc(OMe)-OMe (4.5 mg, 0.020 mmol, 0.40 equiv.). The mixture was then stirred for two days before being quenched with $Na_2SO_{3(aq)}$ (saturated). The reaction was then diluted with DCM and washed with $NaHCO_{3(aq)}$ (saturated, 1x). The aqueous layer was then extracted with DCM (2x). The combined organic layers were dried over Na_2SO_4 and concentrated via rotary evaporation. Crude ^{1}H NMR ratios were obtained at this point.

Table S2. Base Screen.

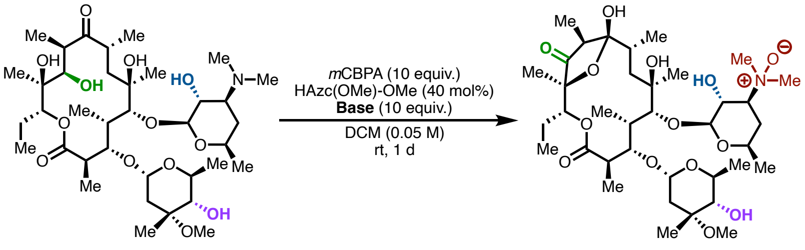

1

5'

Entry	Base	Ratio 2:5'[a]
1	$NaHCO_3$	69:31
2	Na_2CO_3	66:34
3	K_2CO_3	>99:1
4	Cs_2CO_3	>99:1
5	Na_2HPO_4	51:49
6	Li_2CO_3	83:17
7	NaOAc	nd[b]
8	NH_4HCO_3	82:18
9	TBAOH	nd[b]
10	NaPiv	89:11
11	NaOH	91:9
12	K_3PO_4	80:20
13	K_2HPO_4	80:20
14	KH_2PO_4	nd[b]
15	NaH_2PO_4	nd[b]

[a] Determined by NMR. [b] NMR showed too many biproducts to determine.

Equivalents Screen

General Procedure used for equivalents screening:

A 1-dram vial equipped with a stir bar was charged with erythromycin **1** (36.7 g, 0.050 mmol, 1.00 equiv.) and Na_2HPO_4 (**XX equiv.**). DCM (1.00 mL) was added followed by *m*CPBA (**XX equiv.**). The resulting suspension was vigorously stirred at room temperature for 30 minutes before the addition of HAzc(OMe)-OMe (4.5 mg, 0.020 mmol, 0.40 equiv.). The mixture was then stirred for 14 hours before being quenched with excess DMS. The reaction was then diluted with DCM and

washed with $NaHCO_{3(aq)}$ (saturated, 5x). The combined organic layer was dried over Na_2SO_4 and concentrated via rotary evaporation. Crude ^1H NMR ratios were obtained at this point.

Table S3. Equivalents Screen.

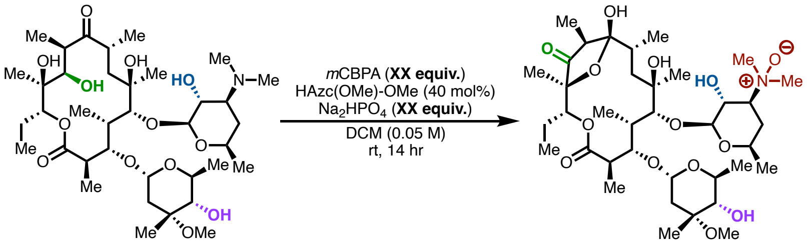

1 5'

Entry	Equiv. *m*CPBA	Equiv. Base	Ratio 2:5'[a]
1	3	3	>99:1
2	5	5	74:26
3	10	10	51:49
4	5	7.5	74:26
5	7.5	5	63:37

[a] Determined by NMR.

<u>Time Screen</u>

General Procedure used for time screening:

A 1-dram vial equipped with a stir bar was charged with erythromycin **1** (36.7 g, 0.050 mmol, 1.00 equiv.) and Na_2HPO_4 (71.0 mg, 0.50 mmol, 10.0 equiv.). DCM (1.00 mL) was added followed by *m*CPBA (119.9 mg, 0.50 mmol, 10.0 equiv.). The resulting suspension was vigorously stirred at room temperature for 30 minutes before the addition of HAzc(OMe)-OMe (4.5 mg, 0.020 mmol, 0.40 equiv.). The mixture was then stirred for **Time** before being quenched with excess DMS. The reaction was then diluted with DCM and washed with $NaHCO_{3(aq)}$ (saturated, 5x). The combined organic layer was dried over Na_2SO_4 and concentrated via rotary evaporation. Crude ^1H NMR ratios were obtained at this point.

Table S4. Time Screen.

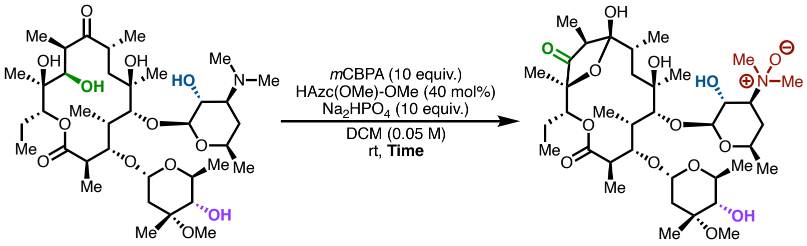

1 5'

Entry	Time (hr)	Ratio 2:5'[a]
1	14	56:44
2	24	51:49
3	64	52:48

[a] Determined by NMR.

Concentration Screen

A 1-dram vial equipped with a stir bar was charged with erythromycin **1** (36.7 g, 0.050 mmol, 1.00 equiv.) and Na_2HPO_4 (71.0 mg, 0.50 mmol, 10.0 equiv.). DCM was added followed by *m*CPBA (119.9 mg, 0.50 mmol, 10.0 equiv.). The resulting suspension was vigorously stirred at room temperature for 30 minutes before the addition of HAzc(OMe)-OMe (4.5 mg, 0.020 mmol, 0.40 equiv.). The mixture was then stirred for 24 hr before being quenched with excess DMS. The reaction was then diluted with DCM and washed with $NaHCO_{3(aq)}$ (saturated, 5x). The combined organic layer was dried over Na_2SO_4 and concentrated via rotary evaporation. Crude ^{1}H NMR ratios were obtained at this point.

N.B. While 0.1 M provided similar ratios compared to 0.05 M, the reaction mixture rapidly became too thick to stir effectively, so 0.05 M was selected to ensure proper mixing and reproducibility.

Table S5. Concentration Screen.

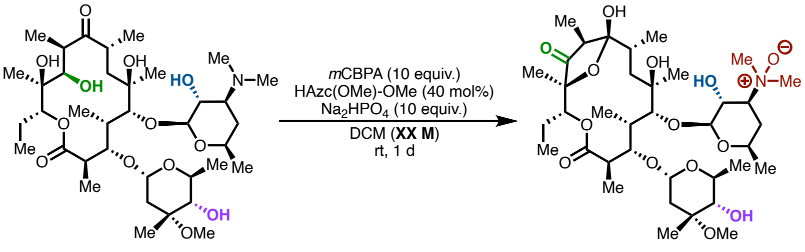

1

5'

Entry	Concentration (M)	Ratio 2:5'[a]
1	0.01	66:34
2	0.025	65:35
3	0.05	51:49
4	0.1	50:50

[a] Determined by NMR.

Solvent Screen

A 1-dram vial equipped with a stir bar was charged with erythromycin **1** (36.7 g, 0.050 mmol, 1.00 equiv.) and Na_2HPO_4 (71.0 mg, 0.50 mmol, 10.0 equiv.). **Solvent** (1.00 mL) was added followed by *m*CPBA (119.9 mg, 0.50 mmol, 10.0 equiv.). The resulting suspension was vigorously stirred at room temperature for 30 minutes before the addition of HAzc(OMe)-OMe (4.5 mg, 0.020 mmol, 0.40 equiv.). The mixture was then stirred for 24 hr before being quenched with DMS. The reaction was then diluted with DCM and washed with $NaHCO_{3(aq)}$ (saturated, 5x). The combined organic layer was dried over Na_2SO_4 and concentrated via rotary evaporation. Crude ^{1}H NMR ratios were obtained at this point.

N.B. DCM and DCE were both used moving forward depending on scale.

Table S6. Solvent Screen

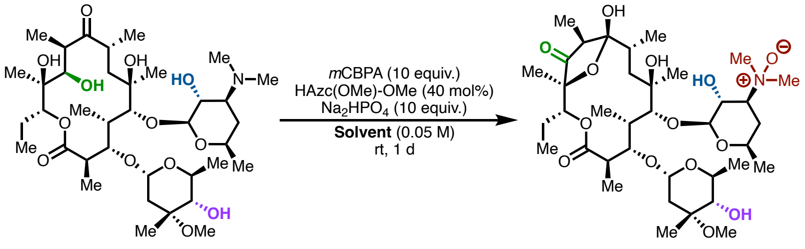

Entry	Solvent	Ratio 2:5'[a]
1	$CHCl_3$	75:25
2	DCE	53:47
3	DCM	51:49
4	Toluene	53:47
5	MTBE	64:36

[a] Determined by NMR.

Catalyst Screen

A 1-dram vial equipped with a stir bar was charged with erythromycin **1** (36.7 g, 0.050 mmol, 1.00 equiv.) and Na_2HPO_4 (71.0 mg, 0.50 mmol, 10.0 equiv.). DCE (1.00 mL) was added followed by *m*CPBA (119.9 mg, 0.50 mmol, 10.0 equiv.). The resulting suspension was vigorously stirred at room temperature for 30 minutes before the addition of **Catalyst** (**Loading**). The mixture was then stirred for 24 hr before being quenched with excess DMS. The reaction was then diluted with DCM and washed with $NaHCO_{3(aq)}$ (saturated, 5x). The combined organic layer was dried over Na_2SO_4 and concentrated via rotary evaporation. Crude ^{1}H NMR ratios were obtained at this point.

Table S7. Catalyst Screen.

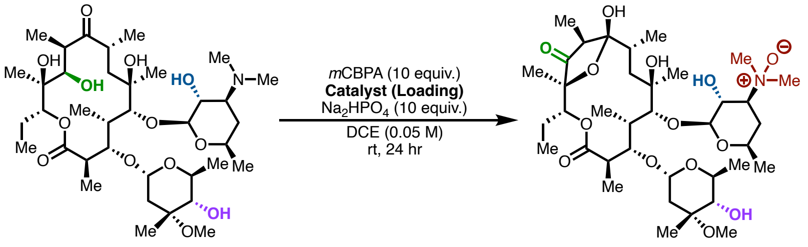

Entry	Catalyst	Loading (mol%)	Ratio 2:5'[a]
1	HAzc-OMe	40	51:49
2	HAzc(OMe)-OMe	40	51:49
2	HAzc(OMe)-OMe	15	71:29
3	Azc-Val-^{D}Pip-Aib-Phe-NMe_2	20	>99:1
4	Azc-Val-^{D}Pro-Aib-Phe-NMe_2	20	>99:1
5	Azc-Val-^{D}Pro-Aib-^{D}Phe-NMe_2	20	>99:1
6	Azc-Val-^{D}Pro-Aib-Dpa-NMe_2	20	>99:1
7	Azc-^{D}Pro-Aib-Phe-NMe_2	20	>99:1
8	HAzc(OMe)-Val-Pro-Aib-Phe-OMe	10	>99:1
9	HAzc(OMe)-Val-^{D}Pro-Deg-Phe-OMe	10	>99:1
10	HAzc(OMe)-Pro-Aib-Phe-OMe	10	>99:1
11	HAzc(OMe)-Pro-OMe	10	>99:1
12	HAzc(OMe)-Val-OMe	10	84:16
13	HAzc(OMe)-Gly-OMe	10	77:23
14[b]	keto-ABNO	40	53:47

[a] Determined by NMR. [b] Run with 5 equiv. Na_2HPO_4 and *m*CPBA.

Additive Screen

A 1-dram vial equipped with a stir bar was charged with erythromycin **1** (36.7 g, 0.050 mmol, 1.00 equiv.), Na_2HPO_4 (71.0 mg, 0.50 mmol, 10.0 equiv.), and **Additive** (40 mg). DCE (1.00 mL) was added followed by *m*CPBA (119.9 mg, 0.50 mmol, 10.0 equiv.). The resulting suspension was vigorously stirred at room temperature for 30 minutes before the addition of HAzc(OMe)-OMe (4.5 mg, 0.020 mmol, 0.40 equiv.). The mixture was then stirred for 24 hr before being quenched with excess DMS. The reaction was then diluted with DCM and washed with $NaHCO_{3(aq)}$ (saturated, 5x). The combined organic layer was dried over Na_2SO_4 and concentrated via rotary evaporation. Crude ^{1}H NMR ratios were obtained at this point.

N.B. In the case of dry *m*CPBA, 360 mg of *m*CPBA was dissolved in 3.00 mL of DCE and washed with sodium phosphate buffer (pH 7.4). 1.00 mL of the organic layer was used in place of the DCE and *m*CPBA (no solid *m*CPBA was added in addition to this).

Table S8. Additive Screen.

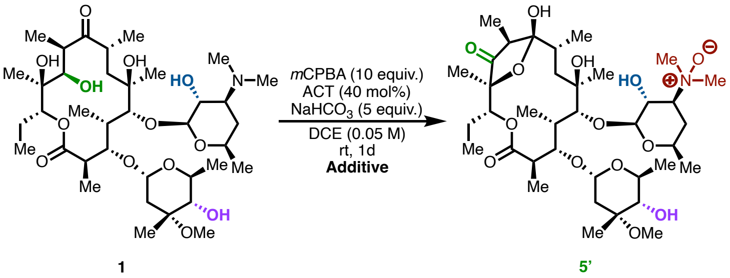

Entry	Additive	Ratio 2:5'[a]
1	10 Å MS	68:32
2	5 Å MS	67:33
3	3 Å MS	76:24
4	*dry m*CPBA	67:33
5	Na_2SO_4	70:30

[a] Determined by NMR.

8. Mechanism Probes

8.1 Acid-Catalyzed Degradation of 11

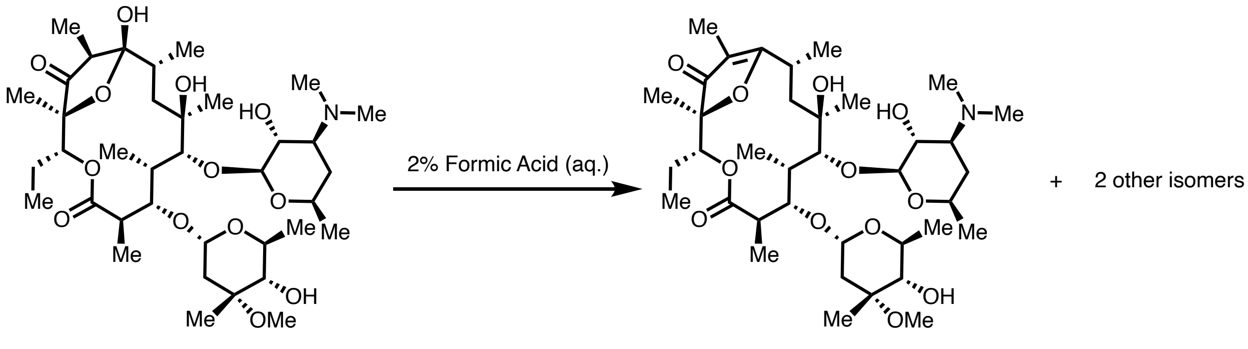

A scintillation vial equipped with a stir bar was charged with C11-keto-erythromycin A, **11**, (6.8 mg, 0.0090 mmol, 1.00 equiv.). Formic acid (2% aqueous, 1 mL) was added, and the resulting solution was vigorously stirred at room temperature for 16 hours. The reaction was then extracted with DCM (3x). The combined organic layers were dried over Na_2SO_4 and concentrated via rotary evaporation. The crude white solid was then analyzed by ^{1}H and ^{13}C NMR and compared with purified C9,12-erythromycin furanone **15** (**Figure S18**, **S19**, and **S20**).

Especially informative in these spectra are the ^{13}C carbon shifts between 174–218 ppm that indicate the presence of 3 distinct erythromycin-derived products with the diagnostic furanone peaks at 193 ppm (**Figure S20**).

Additionally, UPLC/MS data indicates that three observed products result from a dehydration given by the observed $[M+H]^+ = 714$ (**Figure S21** and **S22**).

Based on these spectra, we concluded that **15** appears to be one of three major acid-catalyzed degradation products. This coupled with the bioactivity data (*vide infra*) indicates that the acid-catalyzed degradation of **11** might not lead to the ablation of bioactivity that is reported for erythromycin A.

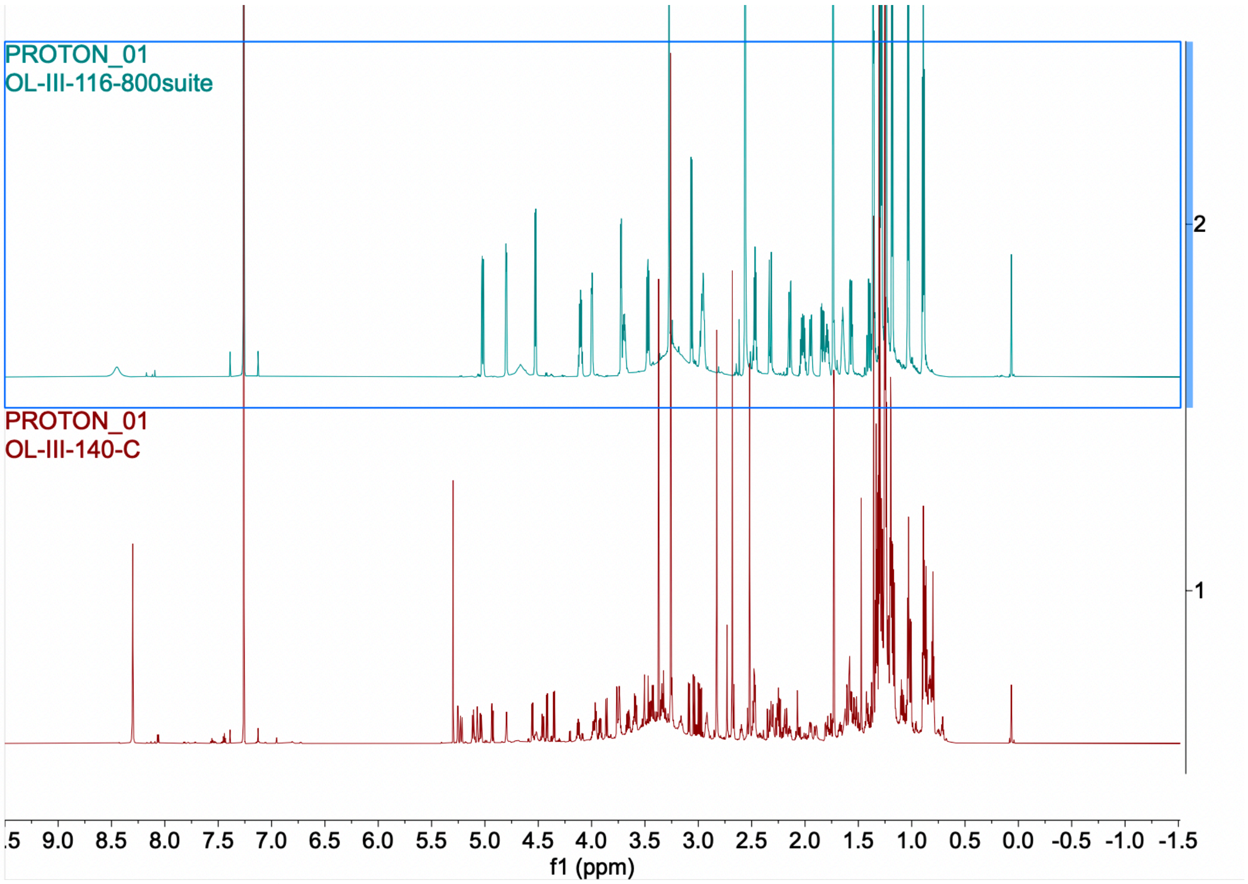

Figure S18. Stacked ^{1}H spectra of **15** (top) and the acid-catalyzed degradation of **11** (bottom) in $CDCl_3$.

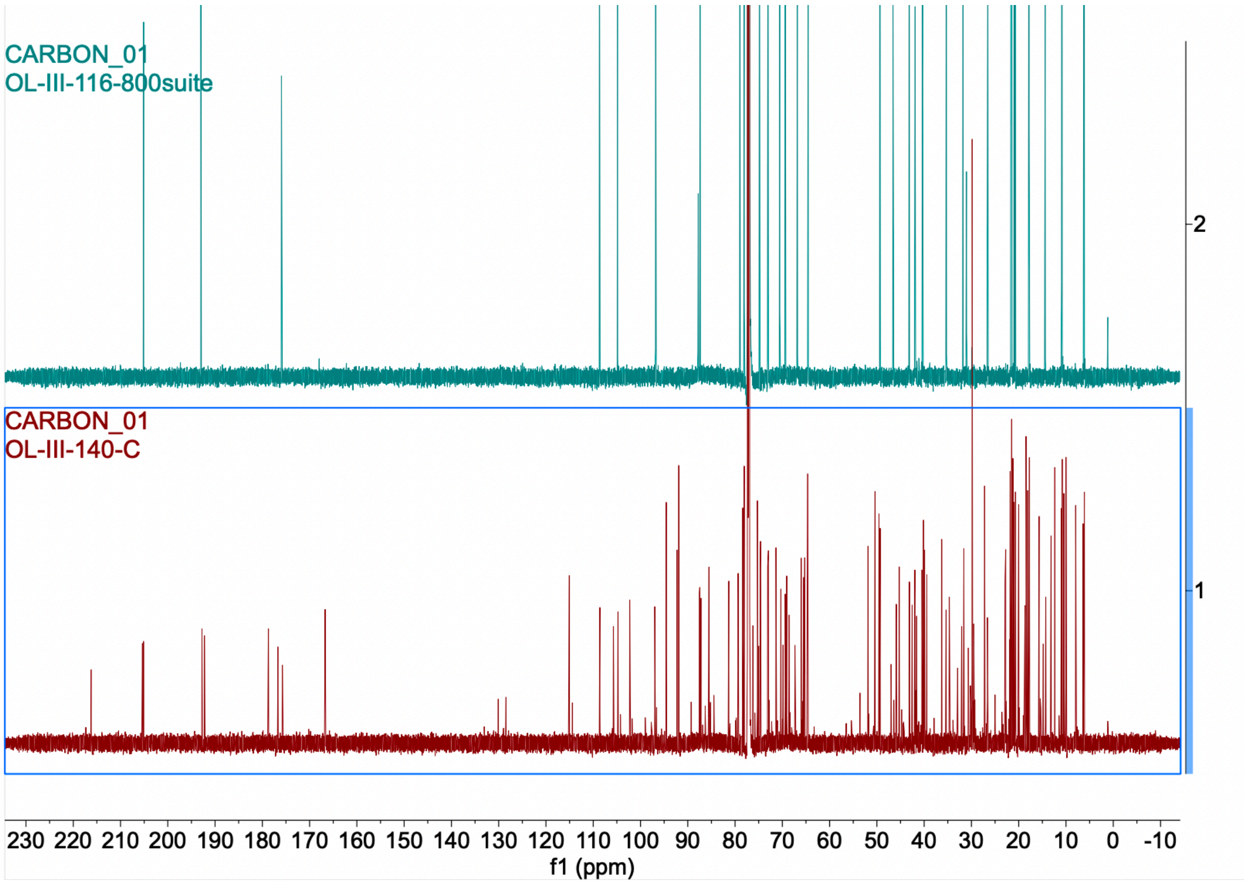

Figure S19. Stacked ^{13}C spectra of **15** (top) and the acid-catalyzed degradation of **11** (bottom) in $CDCl_3$.

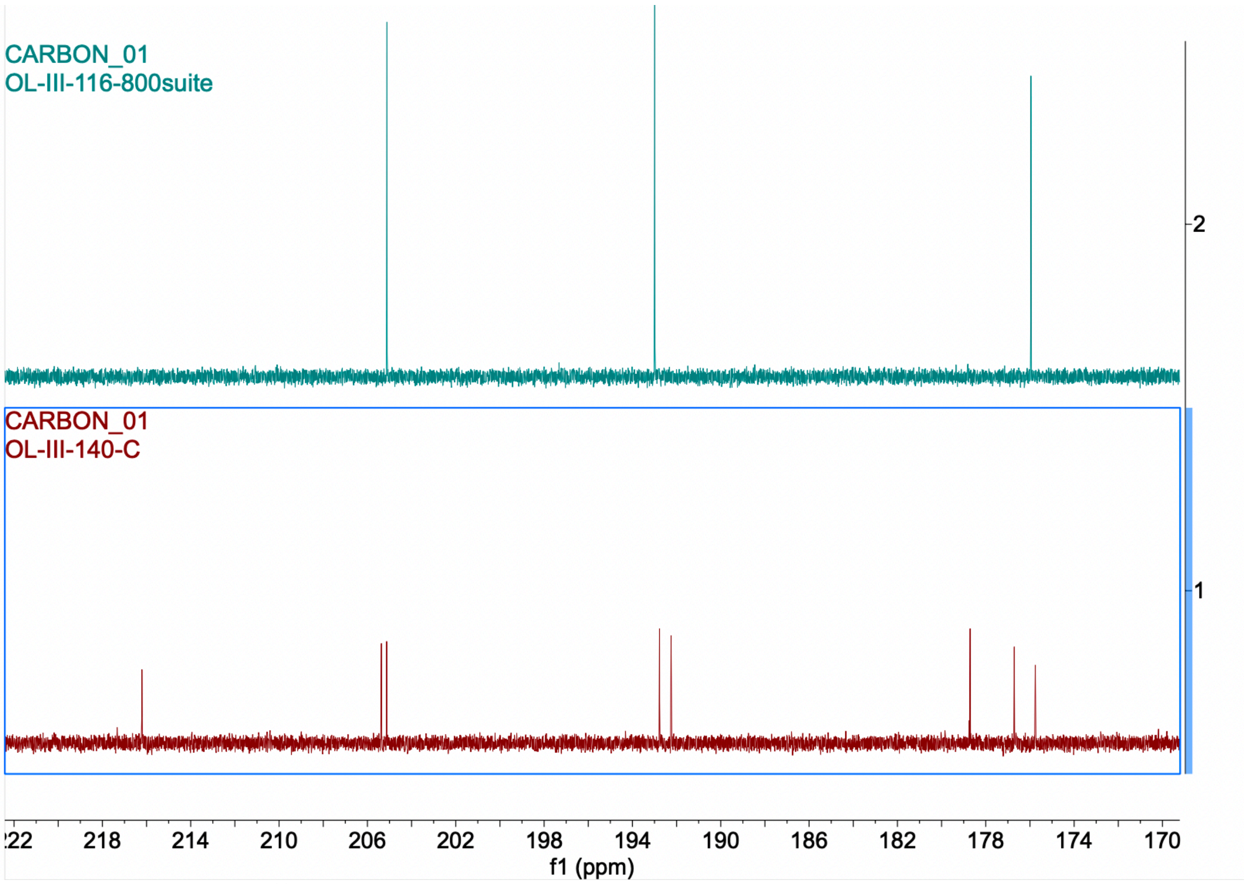

Figure S20. Stacked ^{13}C spectra of **15** (top) and the acid-catalyzed degradation of **11** (bottom) in $CDCl_3$ inset from 170–220 ppm to highlight key furanone peaks.

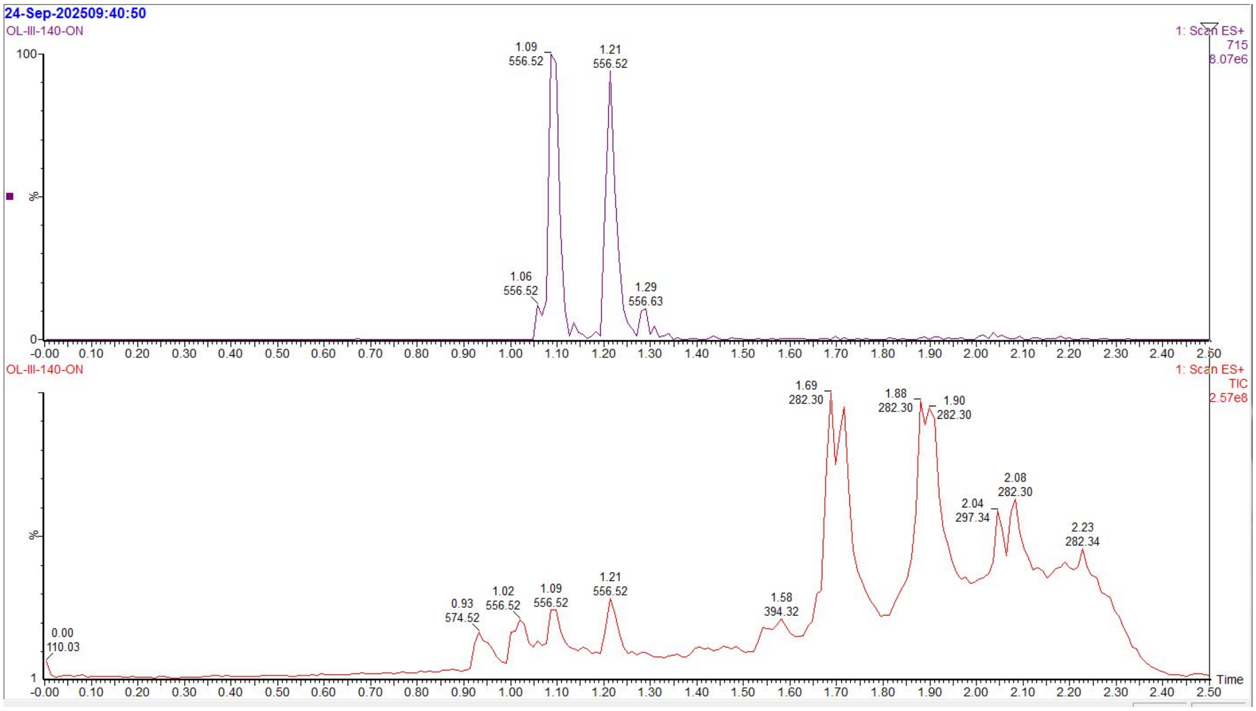

Figure S21. UPLC/MS spectra for the acid-catalyzed degradation of **11** showing the time-resolved trace of the ESI+ data along with the extracted ESI+ for the expected mass.

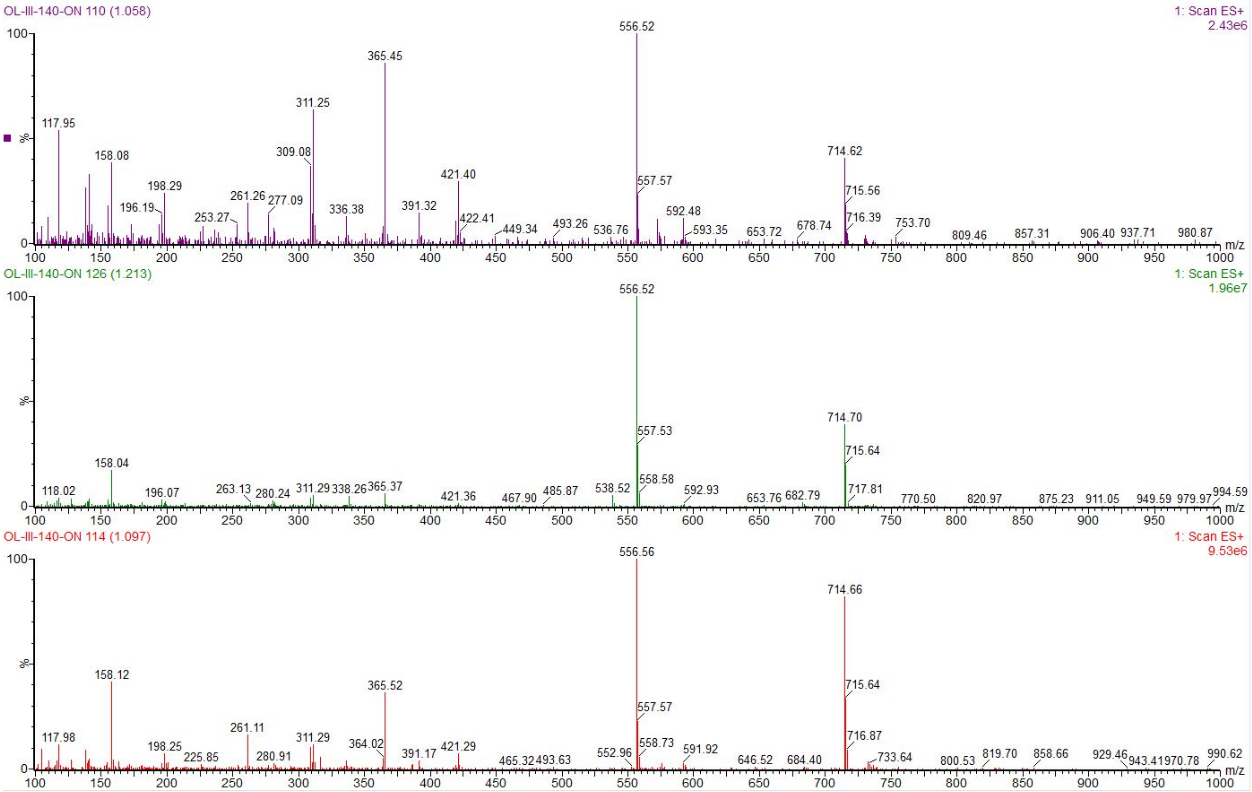

Figure S22. UPLC/MS spectra for the acid-catalyzed degradation of **11** showing the fragmentation of each of the $[M+H]^+ = 714$ for the crude mixture.

8.2 Oxidation of Erythromycin A Spiroketal

Anhydroerythromycin (**S12**)

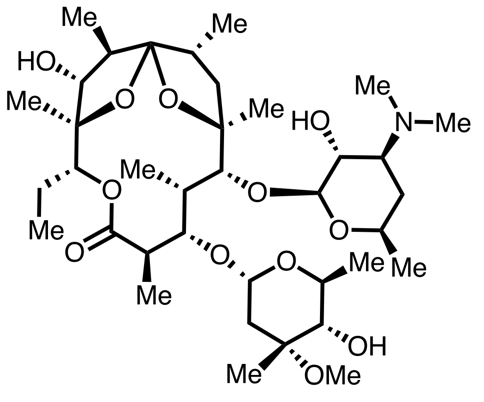

S12

A scintillation vial equipped with a stir bar was charged with erythromycin A **1** (73.3 mg, 0.10 mmol, 1.00 equiv.). Formic acid (2% aqueous, 1 mL) was added, and the resulting solution was vigorously stirred at room temperature for 1 hour. The reaction was then concentrated via rotary evaporation and azeotroped with DCM. The crude white solid anhydroerythromycin **S12** was moved forward without further purification.

R_f: 0.43 (10% MeOH/DCM, visualized with $KMnO_4$ stain)

^{1}H NMR (800 MHz, $CDCl_3$) δ 5.29 (d, *J* = 4.8 Hz, 1H), 5.10 (dd, *J* = 11.5, 3.1 Hz, 1H), 4.48 – 4.44 (m, 1H), 4.41 (d, *J* = 7.2 Hz, 1H), 3.99 – 3.93 (m, 1H), 3.60 – 3.56 (m, 1H), 3.52 (dd, *J* = 10.2, 7.3 Hz, 1H), 3.49 (d, *J* = 2.5 Hz, 2H), 3.43 (d, *J* = 10.8 Hz, 1H), 3.24 (s, 3H), 3.16 (tdd, *J* = 14.8, 11.1, 6.1 Hz, 2H), 2.97 (d, *J* = 9.5 Hz, 1H), 2.76 (s, 6H), 2.34 (dd, *J* = 14.3, 11.8 Hz, 1H), 2.30 – 2.25 (m, 1H), 2.23 (d, *J* = 15.4 Hz, 1H), 2.14 – 2.07 (m, 2H), 1.98 – 1.93 (m, 1H), 1.64 (dqd, *J* = 14.6, 7.3, 2.9 Hz, 1H), 1.51 (dd, *J* = 15.3, 5.0 Hz, 1H), 1.48 – 1.41 (m, 2H), 1.38 (s, 3H), 1.31 (d, *J* = 6.2 Hz, 3H), 1.28 – 1.26 (m, 6H), 1.18 (s, 3H), 1.16 (d, *J* = 6.4 Hz, 3H), 1.13 (d, *J* = 7.6 Hz, 3H), 1.05 – 1.02 (m, 6H), 0.82 (t, *J* = 7.4 Hz, 3H). (N.B. The exchangeable protons are not observed.)

^{13}C{^{1}H} NMR (201 MHz, $cdcl_3$) δ 179.6, 168.7, 116.5, 100.6, 94.3, 87.7, 85.6, 83.2, 81.3, 80.5, 78.4, 75.5, 73.0, 67.9, 67.9, 67.6, 65.1, 51.0, 49.5, 44.9, 41.7, 41.1, 40.9, 40.8, 34.7, 31.3, 27.1, 25.1, 24.4, 21.8, 21.2, 18.8, 18.1, 15.8, 13.2, 12.4, 11.1.

FTIR (solid) cm^{-1}: 2972, 2937, 2363, 2333, 1718, 1595, 1458, 1378.

Optical Rotation: $[\alpha]^{20}_D$ -41.17(*c* = 2.020, MeOH)

HRMS (ESI+) calculated for $C_{37}H_{66}NO_{12}$ $[M+H]^+$ 716.4580, found 716.4567.

Anhydroerythromycin *N*-Oxide (**S13**)

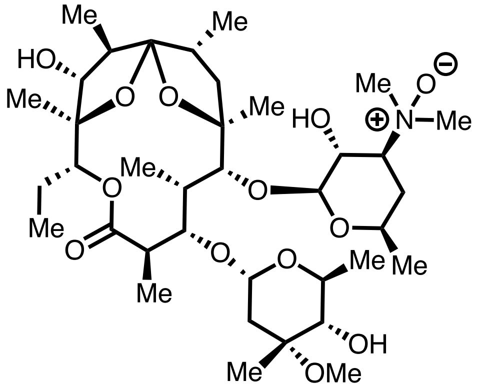

S13

A scintillation vial equipped with a stir bar was charged with erythromycin A **1** (367 mg, 0.50 mmol, 1.00 equiv.). Formic acid (2% aqueous, 5 mL) was added, and the resulting solution was vigorously stirred at room temperature for 1 hour. The reaction was then concentrated via rotary evaporation and azeotroped with DCM. The crude white solid anhydroerythromycin **S12** was moved forward without further purification.

A scintillation vial equipped with a stir bar was charged with anhydroerythromycin **S12** (358 mg, 0.5 mmol, 1.0 equiv.). DCM (10.0 mL) was added followed by *m*CPBA (240. mg, 1.00 mmol, 2.00 equiv.). The resulting suspension was vigorously stirred at room temperature for 4 hours. The reaction was then diluted with $CHCl_3$:IPA (3:1) and washed with $NaHSO_{3(aq)}$ (saturated 1x) followed by $NaHCO_{3(aq)}$ (saturated, 3x). The organic layer was dried over Na_2SO_4 and concentrated via rotary evaporation. The crude white solid anhydroerythromycin *N*-oxide **S13** was moved forward crude without further purification.

R_f: 0.33 (20% MeOH/DCM, visualized with $KMnO_4$ stain)

^{1}H NMR (800 MHz, DMSO-d_6) δ 5.25 (d, J = 5.2 Hz, 1H), 4.99 (d, J = 4.7 Hz, 1H), 4.80 (dd, J = 11.8, 2.8 Hz, 1H), 4.24 (d, J = 7.1 Hz, 1H), 4.22 – 4.20 (m, 1H), 4.05 (dt, J = 16.4, 5.9 Hz, 2H), 3.59 (dtd, J = 11.5, 7.2, 5.4 Hz, 1H), 3.55 (dd, J = 9.9, 7.1 Hz, 1H), 3.35 (dd, J = 11.5, 5.3 Hz, 2H), 3.30 (d, J = 5.9 Hz, 1H), 3.18 (s, 3H), 3.08 – 3.03 (m, 5H), 3.02 (s, 3H), 2.86 (t, J = 9.4 Hz, 1H), 2.34 (t, J = 13.4 Hz, 1H), 2.20 (d, J = 15.0 Hz, 1H), 2.13 – 2.07 (m, 2H), 2.03 (q, J = 6.7 Hz, 1H), 2.01 – 1.98 (m, 1H), 1.50 (ddd, J = 14.2, 7.5, 2.9 Hz, 1H), 1.47 (dd, J = 15.0, 4.8 Hz, 1H), 1.40 (dd, J = 12.4, 6.5 Hz, 1H), 1.31 – 1.27 (m, 4H), 1.16 (s, 3H), 1.15 – 1.13 (m, 6H), 1.11 (s, 3H), 1.08 (d, J = 6.3 Hz, 3H), 1.07 (d, J = 7.5 Hz, 3H), 0.99 (d, J = 7.3 Hz, 3H), 0.96 (d, J = 7.4 Hz, 3H), 0.70 (t, J = 7.4 Hz, 3H). (N.B. One exchangeable proton is not observed.)

^{13}C{^{1}H} NMR (201 MHz, DMSO-d_6) δ 178.5, 115.3, 103.3, 94.7, 86.3, 84.6, 81.2, 80.7, 80.6, 77.8, 75.6, 74.7, 72.8, 70.8, 67.3, 64.4, 59.0, 51.8, 48.8, 48.4, 45.8, 43.3, 41.2, 41.0, 34.5, 34.0, 28.3, 24.8, 23.3, 21.0, 20.8, 17.8, 15.1, 14.2, 13.4, 12.1, 11.0.

FTIR (solid) cm^{-1}: 2971, 2932, 2363, 2337, 1726, 1672, 1457, 1379.

Optical Rotation: $[\alpha]^{20}_{D}$ -48.00 (c = 0.075, MeOH)

HRMS (ESI+) calculated for $C_{37}H_{66}NO_{13}$ $[M+H]^+$ 732.4529, found 732.4527.

Full assignment of the ^{1}H and ^{13}C NMR were undertaken for **S13** to support the structural assignment of **S14** using a combination of ^{1}H, ^{13}C, COSY, HSQC, and HMBC NMR techniques. Each H and C were assigned to the structure (**Figure S23**). Full spectra are included in Section 11 (*vide infra*).

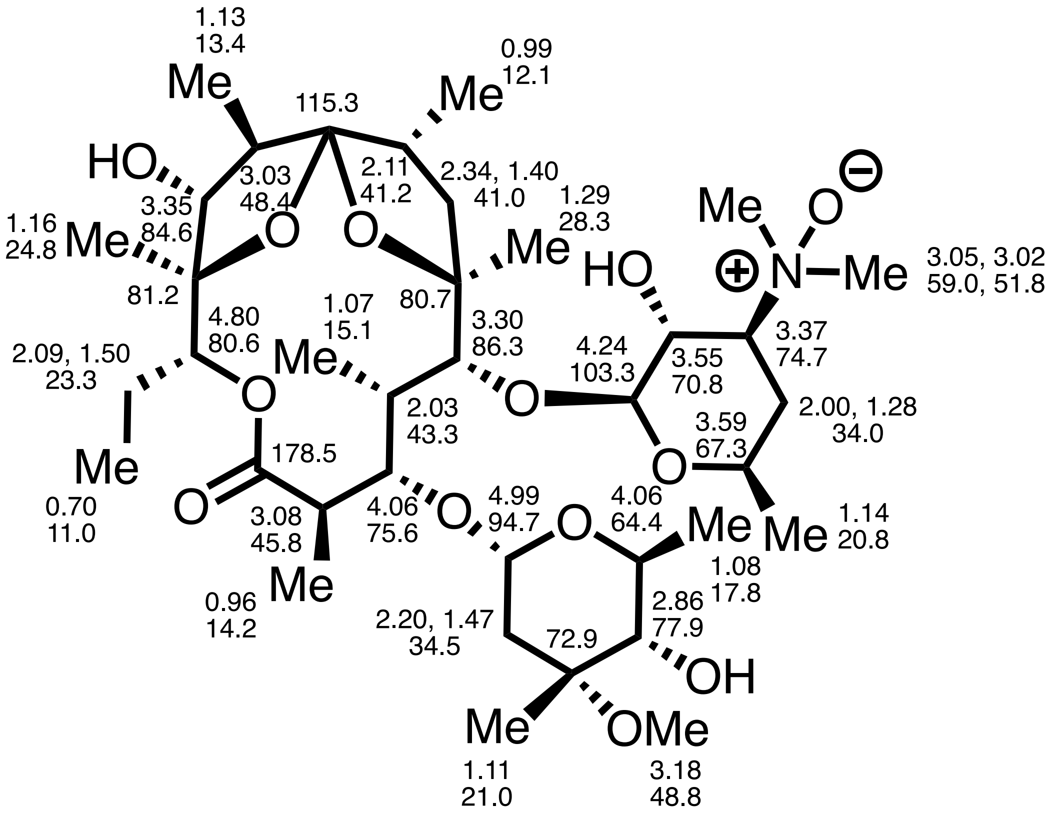

Figure S23. ^{1}H (top) and ^{13}C (top) assignments for anhydroerythromycin *N*-oxide **S13** in DMSO-d_6.

C11-Keto-Anhydroerythromycin *N*-Oxide (**S14**)

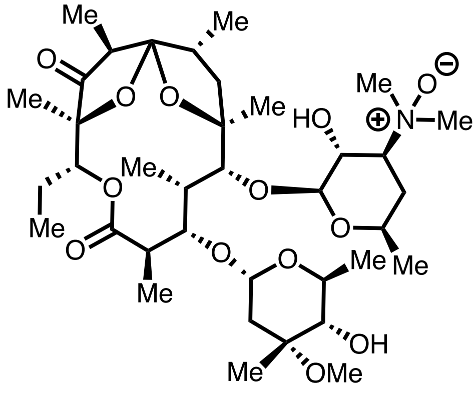

S14

A 1-dram vial equipped with a stir bar was charged with anhydroerythromycin **S12** (31.3 mg, 0.043 mmol, 1.00 equiv.), Na_2HPO_4 (61.0 mg, 0.43 mmol, 10.0 equiv.), and **HAzc(OMe)-OMe** (3.9 mg, 0.017 mmol, 0.40 equiv.). DCM (1.00 mL) was added followed by *m*CPBA (103 mg, 0.43 mmol, 10.0 equiv.). The resulting suspension was vigorously stirred at room temperature for 24 hr. The reaction was quenched by addition of excess DMS then stirred for 1 hour. The reaction was then diluted with $CHCl_3$:IPA (3:1) and washed with $NaHCO_{3(aq)}$ (saturated, 5x). The organic layer was dried over Na_2SO_4 and concentrated via rotary evaporation. The crude yellow solid **S14** was then analyzed without further purification.

^{1}H NMR (800 MHz, DMSO-*d*$_6$) δ 5.16 (d, *J* = 4.9 Hz, 1H), 4.89 (dd, *J* = 11.7, 3.1 Hz, 1H), 4.36 – 4.33 (m, 2H), 4.04 (dq, *J* = 9.5, 6.4 Hz, 1H), 3.68 – 3.65 (m, 1H), 3.46 – 3.45 (m, 2H), 3.44 – 3.42 (m, 2H), 3.35 – 3.34 (m, 1H), 3.16 (s, 3H), 3.11 (s, 3H), 3.07 (s, 3H), 2.87 – 2.85 (m, 1H), 2.44 (dd, *J* = 14.2, 11.9 Hz, 1H), 2.32 – 2.28 (m, 1H), 2.15 (m, 2H), 2.06 – 2.04 (m, 1H), 1.61 – 1.59 (m, 1H), 1.55 (dd, *J* = 12.1, 6.6 Hz, 1H), 1.47 (d, *J* = 5.3 Hz, 1H), 1.41 – 1.38 (m, 1H), 1.34 (s, 3H), 1.31 (d, *J* = 4.1 Hz, 1H), 1.22 (d, *J* = 7.7 Hz, 3H), 1.17 – 1.16 (m, 6H), 1.13 (d, *J* = 7.62, 3H), 1.09 (s, 3H), 1.06 (d, *J* = 6.3 Hz, 3H), 0.96 (d, *J* = 7.2 Hz, 3H), 0.90 (d, *J* = 7.5 Hz, 3H), 0.70 (t, *J* = 7.3 Hz, 3H). (N.B. The exchangeable protons are not notated)

^{13}C{^{1}H} NMR (201 MHz, DMSO-*d*$_6$) δ 216.0, 178.3, 114.6, 101.5, 93.7, 85.8, 84.7, 80.5, 78.8, 77.8, 74.7, 74.3, 72.9, 70.6, 67.5, 64.5, 58.4, 52.5, 51.2, 48.9, 44.5, 41.1, 40.8, 40.7, 34.5, 33.9, 26.0, 22.2, 21.0, 20.9, 19.4, 19.2, 18.2, 13.0, 12.0, 10.9, 9.7.

HRMS (ESI+) calculated for $C_{37}H_{64}NO_{13}$ $[M+H]^+$ 730.4372, found 730.4364.

Full assignment of the ^{1}H and ^{13}C NMR were undertaken to support the structural assignment of **S14** using a combination of ^{1}H, ^{13}C, COSY, HSQC, and HMBC NMR techniques. Each H and C were assigned to the structure (**Figure S24**). Full spectra are included in Section 11 (*vide infra*).

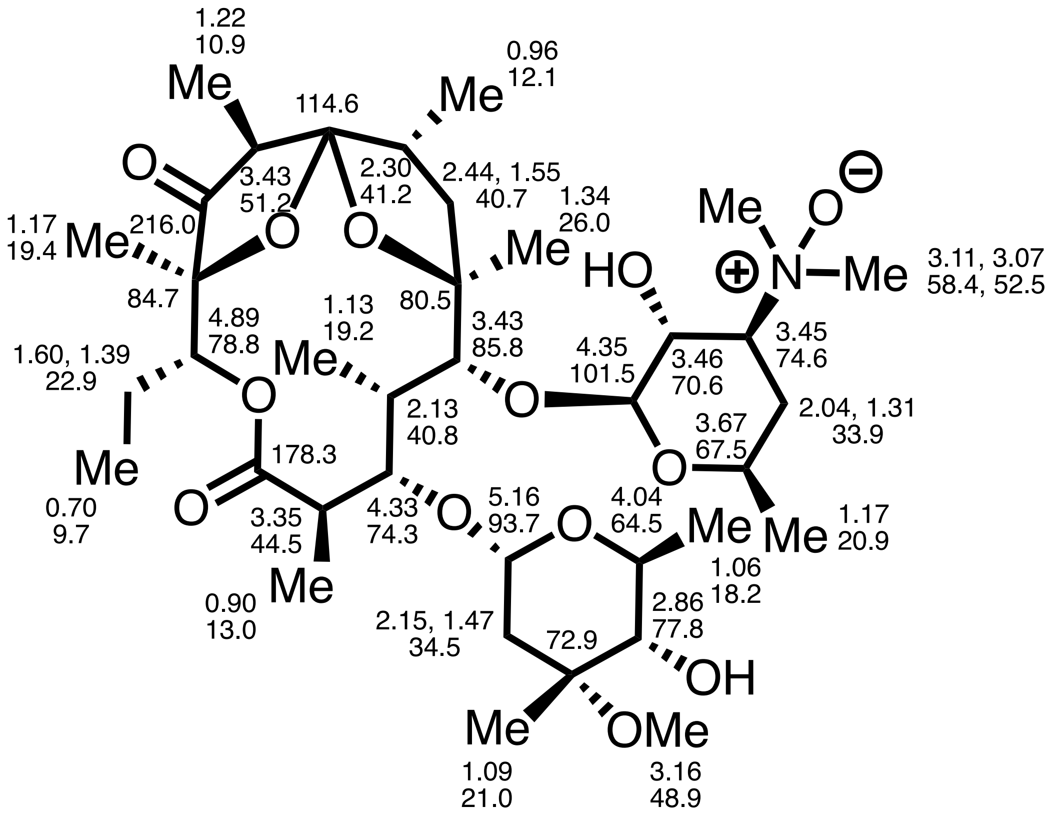

Figure S24. ^{1}H (top) and ^{13}C (bottom) assignments for C11-Keto-Anhydroerythromycin *N*-Oxide **S14** in DMSO-d_6.

Overlay of the HSQC spectra for **S13** (green) and **S14** (red) demonstrates the preservation of the sp^3 C2' (**S13**: 3.55, 70.8; **S14**: 3.46, 70.6) and C4'' (**S13**: 2.86, 77.9; **S14**: 2.86, 77.8) carbons based on the observed connectivity of the ^{1}H signal (**Figure S25**). The C11 proton (**S13**: 3.35, 84.6) is no longer observed in **S14**, supporting the assertion that oxidation has taken place at that site.

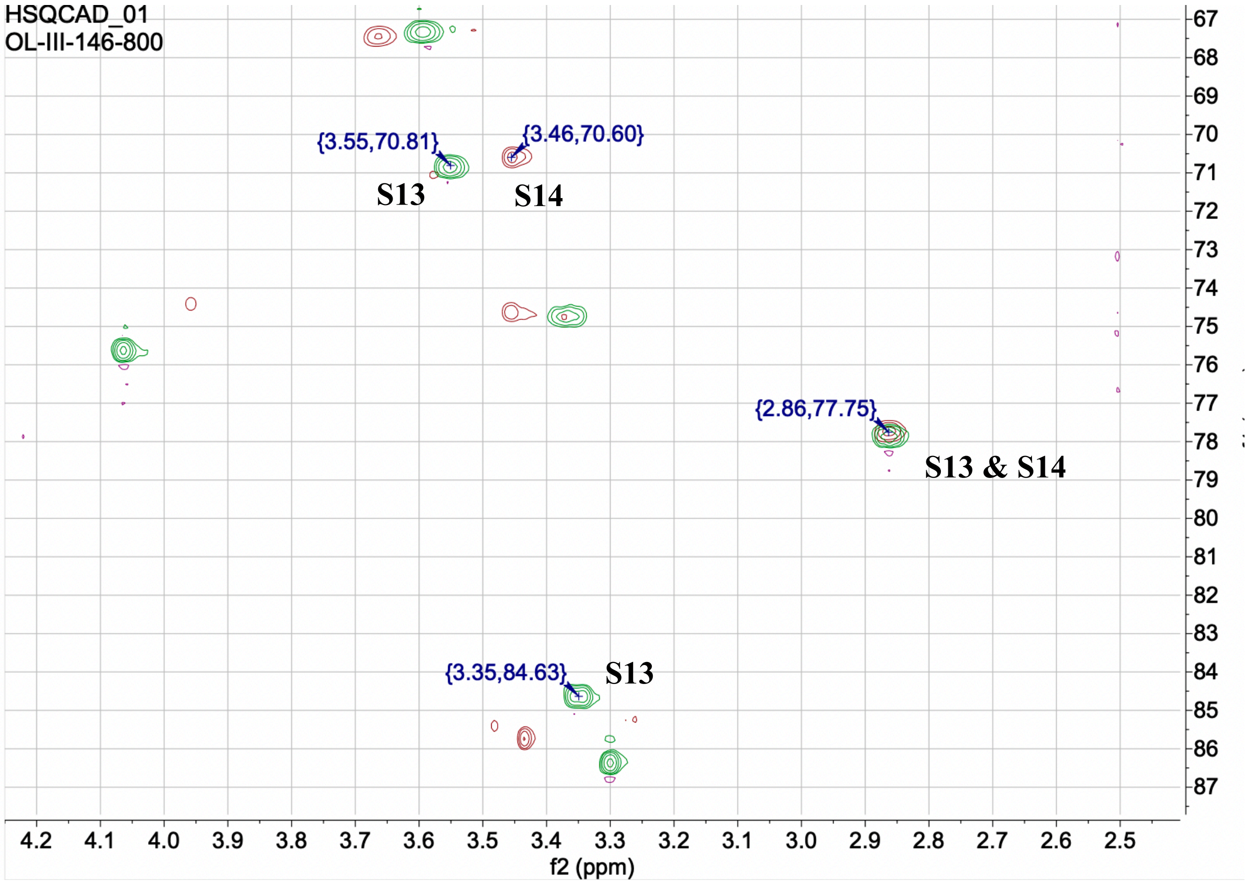

Figure S25. Overlay of HSQC in DMSO-d_6 for **S13** (green) and **S14** (red) highlighting loss of the C11 proton of **S7**.

Characterization and assignment of **S14** is consistent C11 oxidation, as seen on erythromycin A. The clean conversion observed by ^{1}H NMR (**Figure S26**, <1:99, **S13**:**S14**) demonstrates the 5-membered ring within the macrocycle is a competent substrate in the oxidation. While the spiroketal is not a direct analog of the C9,C12-hemiketal erythromycin A, we believe that the reactivity of the spiroketal supports the hypothesis that reactivity of C9,C12-hemiketal erythromycin A could be a contributing pathway in the formation of **5'**.

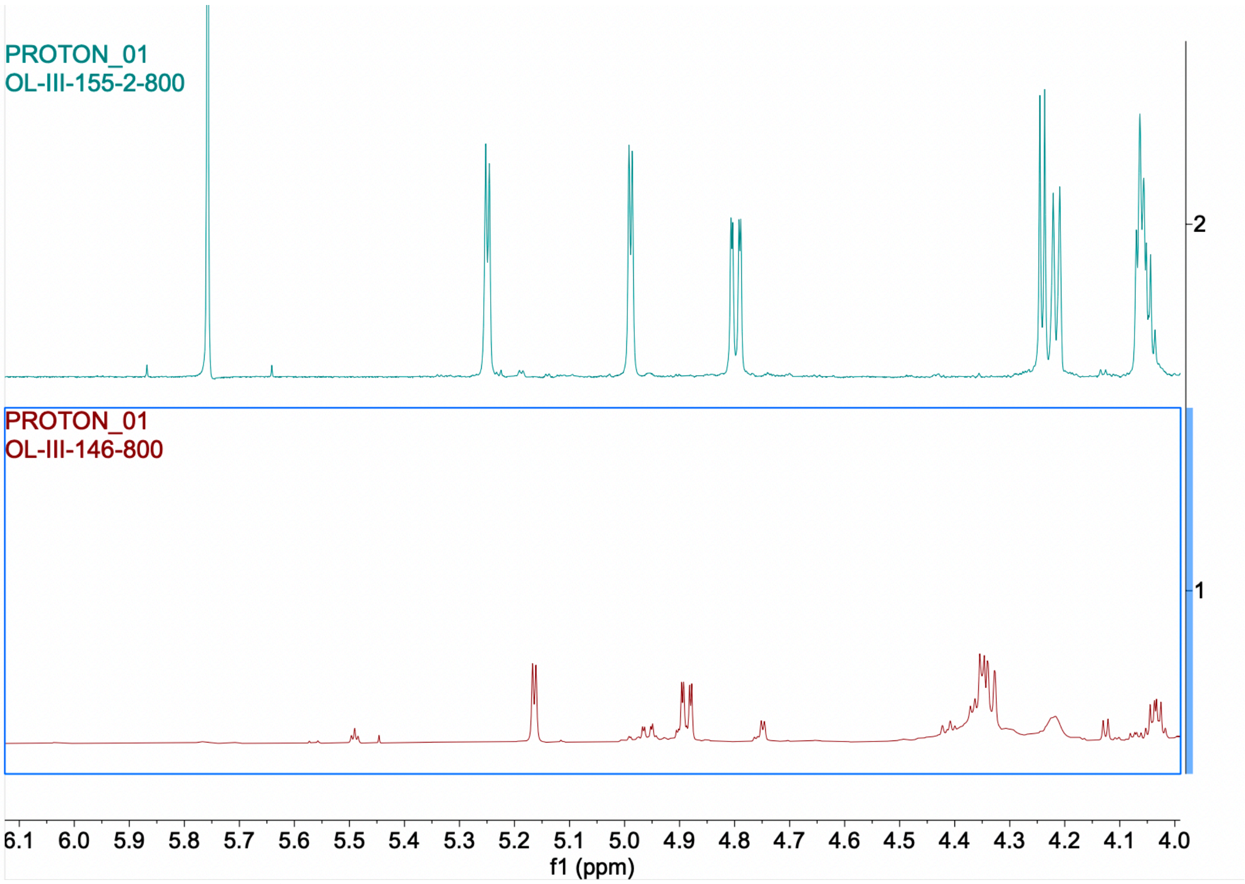

Figure S26. Stacked ^{1}H NMR in DMSO-d_6 for **S13** (green, top) and **S14** (red, bottom) showing the complete consumption of **S13**.

9. ^{13}C NMR Calculations

9.1 Computational Methodologies

Level 0 (Conformational Search): GFN2[11]/ALPB[12](CH_2Cl_2)//GFN-FF[13]/ALPB[12](CH_2Cl_2) via CREST[14] (XTB)

Level 1 (Initial Refinement): GFN2[11]/ALPB[12](CH_2Cl_2)[14] from XTB via TSCoDe.[15]

Level 2 (DFT Geometries and Thermochemical Corrections): R2SCAN- 3c[16]/CPCM[17](DMSO) via ORCA 5.0.4.[18-19] Keywords used were Defgrid3, TightOpt, LARGEPRINT, and Freq, with a MaxStep value of 0.05 Bohrs. Solvent (DMSO) was specified through ε value (47.2) in the %cpcm block of the input file.

Level 3 (DFT Single Point Energies on **Level 2** Structures): ωB97M-V[20]/def2-TZVPP[21]/CPCM[17](DMSO) via ORCA 5.0.4.[18-19] The Defgrid3 keyword was used for added accuracy. Solvent (DMSO) was specified through ε value (47.2) in the %cpcm block of the input file.

Level 4 (Chemical Shieldings Single Point Calculations on **Level 2** structures): PBE0[22]/6-311+G(2d,p)/CPCM[17](DMSO) via ORCA 5.0.4.[18-19] Keywords used were Defgrid3 and NMR. In some cases, SCF convergence required the use of the Slowconv keyword. Solvent (DMSO) was specified through ε value (47.2) in the %cpcm block of the input file. This energy level was chosen because of its good cost-to-accuracy performance in NMR shieldings calculations. The correlation coefficients to convert isotropic shielding values to chemical shifts were adopted from a literature benchmark.[23]

Three-dimensional geometries were obtained from SMILES strings using the Avogadro GUI (Openbabel) and relative stereochemical consistency of the obtained structures was confirmed by visual inspection.[24] The conformational space was explored using CREST[14] (**Level 0**) and followed by similarity and energetic pruning at **Level 1** — both steps were performed through an automated protocol via TSCoDe.[15] The resulting ensembles were then optimized at **Level 2**, and vibrational analysis was carried out at this level. The presence of only positive vibrational frequencies ascertained the local minimum nature of each stationary point. High level single point energy calculations (**Level 3**) and chemical shielding values calculations (**Level 4**) were carried out on **Level 2**-optimized geometries.

For each conformer, the free energy correction (G_{corr}) was extracted from **Level 2**. The single point electronic energies (EE) were taken from **Level 3**, and the isotropic chemical shielding values (σ) were taken from **Level 4**.

The composite free energies (G) for each conformer were calculated using the following equation:

$$G = EE + G_{corr}$$

These energies were then used to calculate the Boltzmann distribution of conformers using the following equations where G_{low} is the energy of the lowest energy conformer for a molecule in kcal/mol, G_{rel} is the relative energy of a conformer compared to the lowest energy conformer, B_f is the Boltzman factor, and P is the fractional population of each conformer based on the Boltzmann distribution:

$$G_{rel} = (G - G_{low})$$
$$B_f = e^{-G_{rel}/(RT)}$$
$$P = \frac{B_f}{\sum B_f}$$

The ^{13}C NMR chemical shifts (C) were then calculated for each conformer using the extracted chemical shielding values (σ) in the following equation based on a literature benchmark:[23]

$$C_i = (\sigma_i - 186.8438)/(-1.0447)$$

These shifts were then weighted using the fractional population (P) to provide the averaged calculated ^{13}C NMR chemical shifts for each ensemble of conformers (C_{avg}).

$$C_{avg} = \sum (C * P)$$

This workflow was performed on parent Erythromycin A and compared to ^{13}C NMR values obtained in house and from external publications for validation and showed an RMS (root mean square) difference of 2.69 ppm (Table S12) indicating good accuracy and giving us confidence using this method moving forward.

9.2 Computational Results

N.B. Only the 25 conformers with lowest energies are shown in the tables for clarity, but all conformers calculated are available in the additional supporting data. Each experimental column contains the values obtained for **5’**, but they have been reorganized to reflect the ordering of the calculated values. For the full structural assignment of **5’**, see **Figure S2**.

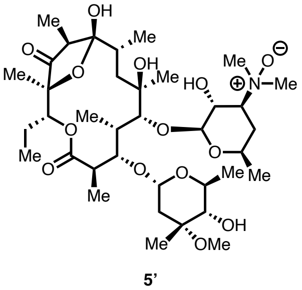

5’

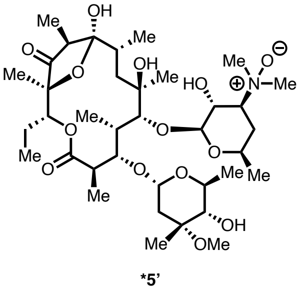

*5’

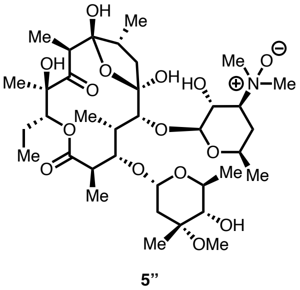

5”

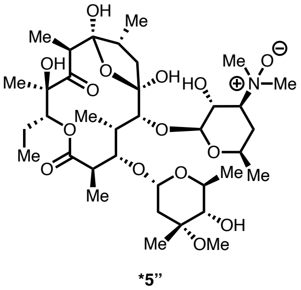

*5”

Table S9. Erythromycin A (**1**) Benchmarking Values.

G_{rel} (kcal/mol)	*P*	C_{avg} (ppm)	*Published* (ppm)[25]
0.00	0.920	0.00	0.00
1.57	0.064	1.57	1.57
2.69	0.010	2.69	2.69
3.71	0.002	3.71	3.71
3.85	0.001	3.85	3.85
4.22	0.001	4.22	4.22
4.27	0.001	4.27	4.27
4.66	0.000	4.66	4.66
4.89	0.000	4.89	4.89
4.92	0.000	4.92	4.92
5.27	0.000	5.27	5.27
5.57	0.000	5.57	5.57
6.00	0.000	6.00	6.00
6.09	0.000	6.09	6.09
6.17	0.000	6.17	6.17
6.26	0.000	6.26	6.26
6.30	0.000	6.30	6.30
6.75	0.000	6.75	6.75
7.49	0.000	7.49	7.49
7.76	0.000	7.76	7.76
8.05	0.000	8.05	8.05
8.14	0.000	8.14	8.14
8.44	0.000	8.44	8.44
9.51	0.000	9.51	9.51
10.05	0.000	10.05	10.05
		0.00	0.00
		1.57	1.57
		2.69	2.69
		3.71	3.71
		3.85	3.85
		4.22	4.22
		4.27	4.27
		4.66	4.66
		4.89	4.89
		4.92	4.92
		5.27	5.27

5.57	5.57

Table S10. Relative Energies and Boltzmann Factors for Each Possible Hemiketal.

5'		*5'		5''		*5''	
G_{rel}	P	G_{rel}	P	G_{rel}	P	G_{rel}	P
0.00	0.246	0.00	0.905	0.00	0.396	0.00	0.246
0.31	0.146	2.17	0.023	0.17	0.295	0.31	0.146
0.31	0.145	2.53	0.013	0.22	0.274	0.31	0.145
0.34	0.139	2.60	0.011	1.72	0.022	0.34	0.139
0.35	0.135	2.61	0.011	2.87	0.003	0.35	0.135
0.36	0.134	2.81	0.008	2.90	0.003	0.36	0.134
1.64	0.015	2.84	0.007	3.20	0.002	1.64	0.015
2.54	0.003	3.08	0.005	3.46	0.001	2.54	0.003
2.55	0.003	3.13	0.005	3.49	0.001	2.55	0.003
2.58	0.003	3.26	0.004	3.68	0.001	2.58	0.003
2.63	0.003	3.35	0.003	3.68	0.001	2.63	0.003
2.71	0.003	3.48	0.003	4.20	0.000	2.71	0.003
3.04	0.001	3.55	0.002	4.33	0.000	3.04	0.001
3.06	0.001	4.46	0.001	4.42	0.000	3.06	0.001
3.07	0.001	5.59	0.000	4.77	0.000	3.07	0.001
3.07	0.001	5.95	0.000	4.91	0.000	3.07	0.001
3.14	0.001	6.18	0.000	5.26	0.000	3.14	0.001
3.23	0.001	6.20	0.000	5.29	0.000	3.23	0.001
3.26	0.001	6.42	0.000	5.33	0.000	3.26	0.001
3.27	0.001	6.43	0.000	5.34	0.000	3.27	0.001
3.31	0.001	6.76	0.000	5.37	0.000	3.31	0.001
3.44	0.001	7.30	0.000	5.60	0.000	3.44	0.001
3.45	0.001	7.42	0.000	5.65	0.000	3.45	0.001
3.45	0.001	7.55	0.000	6.07	0.000	3.45	0.001
3.46	0.001	7.61	0.000	6.33	0.000	3.46	0.001

Table S11. Calculated and Experimental ^{13}C NMR Shifts for Each Possible Hemiketal

5'		*5'		5''		*5''	
C_{avg}	*Exper*	C_{avg}	*Exper*	C_{avg}	*Exper*	C_{avg}	*Exper*
105.3	106.8	106.7	106.8	110.6	106.8	107.3	106.8

50.2	47.4	52.9	47.4	47.4	47.4	60.1	47.4
6.5	10.1	12.8	10.1	16.5	10.1	12.7	10.1
36.9	35.9	34.0	35.9	225.5	217.0	220.9	217.0
20.0	18.5	17.9	18.5	81.4	81.4	82.7	81.4
36.7	38.9	36.9	38.9	82.4	78.1	84.5	78.1
77.2	73.7	75.7	73.7	178.4	177.1	179.2	177.1
24.6	26.0	27.8	26.0	43.5	46.8	44.2	46.8
86.2	83.7	87.7	83.7	73.2	77.6	74.4	77.6
40.2	42.7	39.1	42.7	41.0	42.7	43.1	42.7
78.9	77.6	76.9	77.6	13.6	10.5	8.5	10.5
47.3	46.8	44.2	46.8	81.5	83.7	79.3	83.7
179.4	177.1	177.6	177.1	87.4	73.7	89.3	73.7
78.6	78.1	76.1	78.1	41.2	38.9	37.4	38.9
81.2	81.4	82.0	81.4	42.8	35.9	38.6	35.9
220.6	217.0	222.5	217.0	15.9	18.5	11.4	18.5
18.1	24.0	18.5	24.0	29.0	26.0	24.8	26.0
24.1	22.0	22.8	22.0	100.0	103.0	100.9	103.0
10.0	10.7	10.1	10.7	68.6	67.3	68.0	67.3
15.5	15.7	18.2	15.7	34.7	33.8	34.5	33.8
9.7	10.5	10.3	10.5	76.0	74.6	76.0	74.6
103.9	103.0	103.0	103.0	49.3	52.1	49.4	52.1
68.6	67.3	69.3	67.3	56.8	58.8	57.0	58.8
34.8	33.8	34.4	33.8	19.4	20.7	19.8	20.7
75.5	74.6	75.7	74.6	71.8	71.5	72.5	71.5
49.3	52.1	49.7	52.1	92.9	96.7	93.7	96.7
56.6	58.8	56.8	58.8	32.8	34.8	32.6	34.8
72.9	71.5	72.3	71.5	73.4	72.7	73.4	72.7
78.1	77.6	77.7	77.6	77.6	77.6	77.8	77.6
73.0	72.7	73.2	72.7	65.8	64.9	65.5	64.9
33.1	34.8	33.6	34.8	16.8	17.9	17.2	17.9
95.9	96.7	92.1	96.7	47.5	48.9	47.6	48.9
65.2	64.9	65.8	64.9	20.4	21.0	20.5	21.0
17.4	17.9	16.7	17.9	9.2	15.7	11.2	15.7
47.6	48.9	48.0	48.9	27.0	22.0	25.8	22.0
20.2	21.0	20.1	21.0	12.2	10.7	11.4	10.7
19.7	20.7	19.0	20.7	23.1	24.0	26.2	24.0

Table S12. Average Difference and RMS for Each Possible Hemiketal.

Compound	Average Difference	RMS
**1**	0.341	2.69
**5'**	0.050	1.98
***5'**	0.074	2.36
**5''**	-0.696	3.94
***5''**	-0.501	4.20

10. Biological Assays

Information and Data Provided by Microbiologics. Two rounds of testing were run with the second including known resistance mechanisms for macrolide antibiotics

10.1 Materials and Methods for Round 1 of Testing

Test agents and comparators:

The test agents, thiostrepton, and teicoplanin were provided by Yale and were stored at room temperature prior to testing. The comparator compounds vancomycin, linezolid and ceftazidime were provided by Microbiologics. Stock solutions of each compound were prepared on the day of testing using solvents recommended by CLSI and the client. Stock solutions of each compound were made at 101X the final testing concentration. DMSO (Sigma; St. Louis, MO; Lot No. MKCP0105) was used as the solvent and diluent for Yale compounds. Information regarding compound source, lot number, testing concentrations, and solvent/diluent information for the test agents and comparators are detailed below:

Test Agent	Supplier	Cat No./Lot No.	Test Concentration Range (µg/mL)	Solvent/Diluent
**6**	Yale	-	0.03-32/0.001-1[a]	DMSO/DMSO
**1**	Yale	-	0.03-32/0.001-1	DMSO/DMSO
**7**	Yale	-	0.03-32/0.001-1	DMSO/DMSO
**11**	Yale	-	0.03-32/0.001-1	DMSO/DMSO
**8**	Yale	-	0.03-32/0.001-1	DMSO/DMSO
**2**	Yale	-	0.03-32/0.001-1	DMSO/DMSO
**5'**	Yale	-	0.03-32/0.001-1	DMSO/DMSO
Thiostrepton	Yale	-	0.03-32/0.001-1	DMSO/DMSO
Teicoplanin	Yale	-	0.03-32/0.001-1	DMSO/DMSO
Vancomycin	Sigma	V2002/080M1341V	0.016-16	diH_2O/diH_2O
Linezolid	Selleckchem	S1408/S140802	0.016-16	diH_2O/diH_2O
Ceftazidime	Sigma	C3809/117M4826V	0.016-16	diH_2O/diH_2O

[a]Yale compounds test ranges against streptococci were 0.001-1 µg/mL

Test organisms:

The test organisms evaluated in this study consisted of reference strains from the American Type Culture Collection (ATCC; Manassas, VA) and clinical isolates from the Microbiologics repository (MMX; Kalamazoo, MI).

Upon receipt at Microbiologics, the isolates were streaked under suitable conditions onto agar media appropriate to each organism and were incubated for 18 to 24 hr at 35 ºC. Colonies harvested from these growth plates were resuspended in the appropriate medium containing a cryoprotectant. Aliquots of each suspension were then frozen at -80 ºC. Prior to testing, the isolates were streaked from frozen vials onto Trypticase soy agar with 5% sheep blood (Remel; Lenexa, KS; Lot No. 2146991) and incubated under optimal conditions for growth.

Test medium:

The test medium used for the broth microdilution was cation adjusted Mueller Hinton broth (CAMHB; Becton Dickinson [BD]; Franklin Lakes, NJ; Lot No. 4102223). The medium was prepared according to guidelines from CLSI.[26-27] For *S. pneumoniae,* this medium was supplemented with 3% laked horse blood (LHB; Hemostat, Dixon, CA; Lot No. 788088-1-1).

Broth Microdilution MIC Assay:

The MIC assay method followed the broth microdilution procedure described by CLSI and employed automated liquid handlers to conduct serial dilutions and liquid transfers.[26-27] Automated liquid handlers included the Multidrop 384 (Labsystems, Helsinki, Finland), Biomek 3000, and Biomek FX (Beckman Coulter, Fullerton CA).

The wells in columns 2 through 12 in a standard 96-well microdilution plate (Costar 3795) were filled with 150 µl of the appropriate diluent. These plates would become the ‘mother plates’ from which ‘daughter’ or test plates would be prepared. The drugs (300 µL at 101X the desired top concentration in the test plates) were dispensed into the appropriate well in column 1 of the mother plates. The Biomek 3000 was used to make serial two-fold dilutions through column 11 in the “mother plates.” The wells of column 12 contained no drug and served as the organism growth control wells.

Rows A through H of the daughter plates were loaded with 190 µL per well of the appropriate test medium using Multidrop 384. The daughter plates were prepared using the Biomek FX which transferred 2 µL of drug solution from each well of the mother plates to the corresponding well of the daughter plates in a single step.

A standardized inoculum of each organism was prepared by CLSI methods.[26-27] Colonies were picked from the primary plate and a suspension was prepared to equal a 0.5 McFarland turbidity standard. Suspensions were diluted 1:20 in the appropriate medium and transferred to compartments of sterile reservoirs divided by length (Beckman Coulter). The Biomek 3000 was used to inoculate the plates. Daughter plates were placed on the Biomek 3000 in reverse orientation so that plates were inoculated from low to high drug concentration. The Biomek 3000 delivered 10 µL of standardized inoculum into each well of the appropriate daughter plate for an additional 1:20 dilution targeting a final inoculum concentration of approximately 5 x 10^5 CFU/mL.[26-27]

The plates were stacked 3 to 4 high, covered with a sterile lid on the top plate, and incubated aerobically at 35 ºC for 16 to 24 hr according to CLSI standards. Following incubation, the microplates were removed from the incubator and viewed from the bottom using a plate viewer. For each of the test media and drugs, an un-inoculated solubility control plate was observed for evidence of drug precipitation. The MIC was recorded as the lowest concentration of drug that completely inhibited visible growth of the organism.

Results of susceptibility testing of the test compounds are shown in **Table S13**. No precipitation was observed with test articles in this study. The comparators were within the established CLSI quality control ranges for *Staphylococcus aureus* ATCC 29213, *Enterococcus faecalis* ATCC 29212, *Streptococcus pneumoniae* ATCC 49619, and *Escherichia coli* ATCC 25922 (**Table S13**).

10.2 Activity Results for Round 1 of Testing

Table S13. Activity of Yale test agents against Gram-positive pathogens, including resistant isolates

Species	Isolate info	MIC (µg/mL)											
		**6**	**1**	**7**	**11**	**8**	**2**	**5'**	**THI**	**TCO**	**VAN**	**LZD**	**CAZ**
S. aureus	ATCC 29213 (MSSA; QC)	0.25	0.5	>32	2	>32	>32	>32	0.06	0.5 (0.25-1)	1 (0.5-2)	4 (1-4)	16
	NRS384 (USA300; MRSA)	32	>32	>32	>32	>32	>32	>32	≤0.03	0.5	1	4	>16
E. faecalis	ATCC 29212 (VSE; QC)	1	2	>32	4	>32	>32	>32	≤0.03	0.25 (0.25-1)	2 (1-4)	2 (1-4)	>16
	MMX 486 (VanA; VRE)	>32	>32	>32	>32	>32	>32	>32	0.06	>32	>16	1	>16
E. faecium	ATCC 19434 (VSE)	2	2	>32	2	>32	>32	>32	0.06	0.5	0.5	4	>16
	MMX 485 (VanA; VRE)	>32	>32	>32	>32	>32	>32	>32	0.06	>32	>16	1	>16
	MMX 487 (VanB; VRE)	>32	>32	>32	>32	>32	>32	>32	≤0.03	0.5	>16	2	>16
S. pneumoniae	ATCC 49619 (PISP; QC)	0.03	0.03	1	0.12	1	1	>1	≤0.001	0.12	0.25 (0.12-05)	2 (0.25-2)	1
S. pyogenes	ATCC 19615	0.016	0.03	0.25	0.12	0.25	0.5	>1	0.002	0.12	0.25	1	0.12
E. coli	ATCC 25922 (QC)	32	>32	>32	>32	>32	>32	>32	>32	>32	>16	>16	0.25 (0.06-0.5)

THI, thiostrepton; TCO, teicoplanin; VAN, vancomycin; LNZ, linezolid; CAZ, Ceftazidime. MSSA, methicillin-susceptible Staphylococcus aureus; MRSA, methicillin-resistant *S. aureus*; VSE, vancomycin-susceptible Enterococci; VRE, vancomycin-resistant Enterococci; PISP, penicillin-intermediate resistant S. pneumoniae.

CLSI QC ranges shown in parenthesis.

10.3 Materials and Methods for Round 2 of Testing

Test Articles and Comparators:

The test articles were provided in powder form by Yale University. The compounds were stored at room temperature prior to testing. Comparator compounds were provided by Microbiologics. Stock solutions of all test compounds were prepared on the day of testing using DMSO for the Yale test agents and solvents recommended by the CLSI for comparators.[26-27] Stock solutions of test agents were made at 101X the final testing concentration. Information regarding test agents and comparators is detailed below:

Test Agent	Supplier	Cat No./Lot No.	Test Concentration Range (µg/mL)	Solvent/Diluent
**2**	Yale	-	0.06-64	DMSO/DMSO
**12'**	Yale	-	0.06-64	DMSO/DMSO
**14**	Yale	-	0.06-64	DMSO/DMSO
**15**	Yale	-	0.06-64	DMSO/DMSO
**17**	Yale	-	0.06-64	DMSO/DMSO
**18**	Yale	-	0.06-64	DMSO/DMSO
**19**	Yale	-	0.06-64	DMSO/DMSO
**20**	Yale	-	0.06-64	DMSO/DMSO
**21**	Yale	-	0.06-64	DMSO/DMSO
**22**	Yale	-	0.06-64	DMSO/DMSO
**23**	Yale	-	0.06-64	DMSO/DMSO
**6**	USP	R196J0	0.016-16	Water (glacial acetic acid dropwise)/water
**9**	USP	R103C0	0.016-16	Water (glacial acetic acid dropwise)/water
**1**	Sigma	011M150V	0.016-16	Water (glacial acetic acid dropwise)/water
Levofloxacin	Sigma	038M4848V	0.016-16	Water/water

Test organisms:

Test organisms consisted of isolates from the American Type Culture Collection (ATCC; Manassas, VA), Network of Antimicrobial Resistance in *S. aureus* (NRS; BEI Resources, Manassas, VA), and the Microbiologics Repository (MMX; Kalamazoo, MI). Upon initial receipt at Microbiologics, the organisms were sub-cultured onto an appropriate agar medium and incubated under atmospheric conditions required for growth. Following incubation for 18 to 24 hr at 35 °C in the appropriate atmosphere, colonies were harvested from these plates and cell suspensions were prepared and frozen at -80 ºC with a cryoprotectant.

Prior to testing, isolates were streaked from frozen vials onto the appropriate agar. Unless otherwise noted, Trypticase Soy Agar with 5% sheep blood (TSA; Remel; Lenexa, KS; Lot No. 227964) was used and inoculated plates were incubated at 35 °C overnight in ambient atmosphere. *Haemophilus influenzae* isolates were streaked onto Chocolate agar (Remel; Lot No. 117772).

Test medium:

Organisms were tested in the appropriate media according to CLSI guidelines.[26-27] Cation-adjusted Mueller Hinton broth (CAMHB; BD/BBL Lot No. 4102223) was used for broth microdilution testing with the following exceptions: CAMHB was supplemented with 5% lysed horse blood (LHB; Hemostat; Dixon, CA; Lot No. 788088) for testing of *Streptococcus* spp. and for *H. influenzae* testing, *Haemophilus* Test medium (HTM) was made by supplementing MHB (Difco, Lot No. 3150802) with 15 µg/mL nicotinamide adenine dinucleotide (NAD; Sigma; Lot No. SLBX4629), 15 µg/mL hematin porcine (Sigma; Lot No. SLCL9885), and 5 g/L of yeast extract (BD; Lot No. 7179576).

Broth Microdilution MIC Assay:

The MIC assay method followed the procedures described by CLSI and employed automated liquid handlers to conduct serial dilutions and liquid transfers. Automated liquid handlers included the Multidrop Combi (ThermoScientific), Biomek 3000, and Biomek FX (Beckman Coulter; Fullerton, CA).

The wells in columns 2 through 12 in standard 96-well microdilution plates (Costar 3795) were filled with 150 µl of the appropriate diluent. These would become the "mother plates" from which "daughter," or test plates, would be prepared. The drugs (300 µL at 101X the desired top concentration in the test plates) were dispensed into the appropriate well in column 1 of the mother plates. The Biomek 3000 was used to make serial two-fold dilutions through column 11 in the "mother plate." The wells of column 12 contained no drug and served as the organism growth control wells.

Rows A through H of the daughter plates were loaded with 190 µL per well of the appropriate test medium using the Multidrop Combi. The daughter plates were prepared using the Biomek FX which transferred 2 µL of drug solution from each well of a mother plate to the corresponding well of the daughter plate in a single step. A standardized inoculum of each organism was prepared per CLSI.[26-27] Colonies were picked from the streak plate and a suspension was prepared to equal a 0.5 McFarland turbidity standard in saline. Suspensions were then diluted 1:10 and transferred to compartments of sterile reservoirs. Daughter plates were placed on the Biomek 3000 in reverse orientation so that plates were inoculated from low to high drug concentration. The Biomek 3000 delivered 10 µL of standardized inoculum into each well of the appropriate daughter plate for an additional 1:20 dilution, targeting a final concentration of 5 x 105 CFU/mL.

Plates were stacked 3 to 4 high, covered with a sterile lid on the top plate, placed in plastic

bags, and incubated at 35ºC and read at 20 hr. An un-inoculated control plate was observed for evidence of drug precipitation or contamination. MIC values were read where visible growth of the organism was inhibited according to CLSI guidelines.[26-27]

Results of susceptibility testing of the test compounds are shown in **Table S14**. MIC values were within CLSI-established test ranges for the comparator drugs against the QC organisms, except for levofloxacin which was one-dilution above the QC range for *Haemophilus influenzae* ATCC 49247 although this agent was in QC with the three other evaluated QC organisms. (**Table S14**).

10.4 Activity Results for Round 2 of Testing

Table S14. Activity of Yale test agents against Gram-positive pathogens, including resistant isolates

Species	Isolate info	MIC (µg/mL)							
		2	5'	7	8	10	11	12	13
S. aureus	ATCC 29213 (MSSA; QC)	>64	>64	64	64	16	4	>64	8
	NRS 384 (USA300; CA-MRSA)	>64	>64	>64	>64	>64	>64	>64	16
	MMX 2170 (mph(C); MRSA)	>64	>64	>64	>64	>64	>64	>64	16
	MMX 3247 (mph(C); MSSA)	>64	>64	>64	>64	>64	32	>64	16
	MMX 3037 (erm(C))	>64	>64	>64	>64	>64	>64	>64	>64
	MMX 3067 (efr)	>64	>64	>64	>64	>64	>64	>64	>64
E. faecalis	ATCC 29212 (VSE; QC)	>64	>64	64	>64	32	4	>64	2
	MMX 486 (VanA; VRE)	>64	>64	>64	>64	>64	>64	>64	>64
E. faecium	ATCC 19434 (VSE)	>64	>64	>64	>64	64	16	>64	4
	MMX 0485 (VanA; VRE)	>64	>64	>64	>64	>64	>64	>64	>64
	MMX 0487 (VanB; VRE)	>64	>64	>64	>64	>64	>64	>64	>64
	MMX 0854 (erm(A); erm(B))	>64	>64	>64	>64	>64	>64	>64	>64
S. pneumoniae	ATCC 49619 (PISP; QC)	1	4	0.5	1	1	0.12	4	0.25
	MMX 3033 (erm(B))	>64	>64	>64	>64	>64	>64	>64	>64
S. pyogenes	ATCC 19615	0.25	2	0.12	0.5	0.5	0.12	4	0.25
H. influenzae	ATCC 49427 (QC)	>64	>64	>64	>64	32	>64	>64	>64
	ATCC 49766 (QC)	>64	>64	>64	>64	32	>64	>64	>64
M. catarrhalis	ATCC 25238	32	>64	16	32	0.25	1	>64	2
	ATCC 8176	32	>64	16	32	0.25	1	>64	2
E. coli	ATCC 25922 (QC)	>64	>64	>64	>64	64	>64	>64	>64

Table S14. Activity of Yale test agents against Gram-positive pathogens, including resistant isolates (*continued*)

Species	Isolate info	MIC (µg/mL)						
		14	15	16	6	9	1	LVX
S. aureus	ATCC 29213 (MSSA; QC)	>64	4	0.5	0.25 (0.12-0.5)	1 (0.5-2)	0.5 (0.25-1)	0.5 (0.06-0.5)
	NRS 384 (USA300; CA-MRSA)	>64	64	16	>16	>16	>16	1
	MMX 2170 (mph(C); MRSA)	>64	64	8	>16	>16	>16	8
	MMX 3247 (mph(C); MSSA)	>64	16	4	4	16	8	0.25
	MMX 3037 (erm(C))	>64	>64	>64	>16	>16	>16	0.12
	MMX 3067 (efr)	>64	>64	>64	>16	>16	>16	8
E. faecalis	ATCC 29212 (VSE; QC)	>64	4	1	1	4	1 (1-4)	0.5 (0.25-2)
	MMX 486 (VanA; VRE)	>64	>64	>64	>16	>16	>16	>8
E. faecium	ATCC 19434 (VSE)	>64	8	2	2	16	4	>8
	MMX 0485 (VanA; VRE)	>64	>64	>64	>16	>16	>16	2
	MMX 0487 (VanB; VRE)	>64	>64	>64	>16	>16	>16	2
	MMX 0854 (erm(A); erm(B))	>64	>64	>64	>16	>16	>16	>8
S. pneumoniae	ATCC 49619 (PISP; QC)	4	0.12	≤0.06	0.03 (0.03-0.12)	0.06 (0.06-0.25	0.03 (0.03-0.12)	1 (0.5-2)
	MMX 3033 (erm(B))	>64	>64	>64	>16	>16	>16	1
S. pyogenes	ATCC 19615	2	0.12	≤0.06	≤0.0016	0.03	0.03	0.5
H. influenzae	ATCC 49427 (QC)	64	64	16	8 (4-16)	2 (1-4)	8	0.06 (0.008-0.03)
	ATCC 49766 (QC)	64	>64	32	16	1	8	0.03
M. catarrhalis	ATCC 25238	32	2	0.12	0.06	≤0.0016	0.12	0.03
	ATCC 8176	32	1	0.12	0.06	≤0.0016	0.12	0.03
E. coli	ATCC 25922 (QC)	>64	>64	64	>16	2	>16	0.016

LVX, levofloxacin. MSSA, methicillin-susceptible *Staphylococcus aureus*; MRSA, methicillin-resistant *S. aureus*; CA, community-acquired; VSE, vancomycin-susceptible *Enterococci*; VRE, vancomycin-resistant *Enterococci*; PISP, penicillin-intermediate resistant *S. pneumoniae*.
CLSI QC ranges shown in parenthesis.

11. NMR Spectra

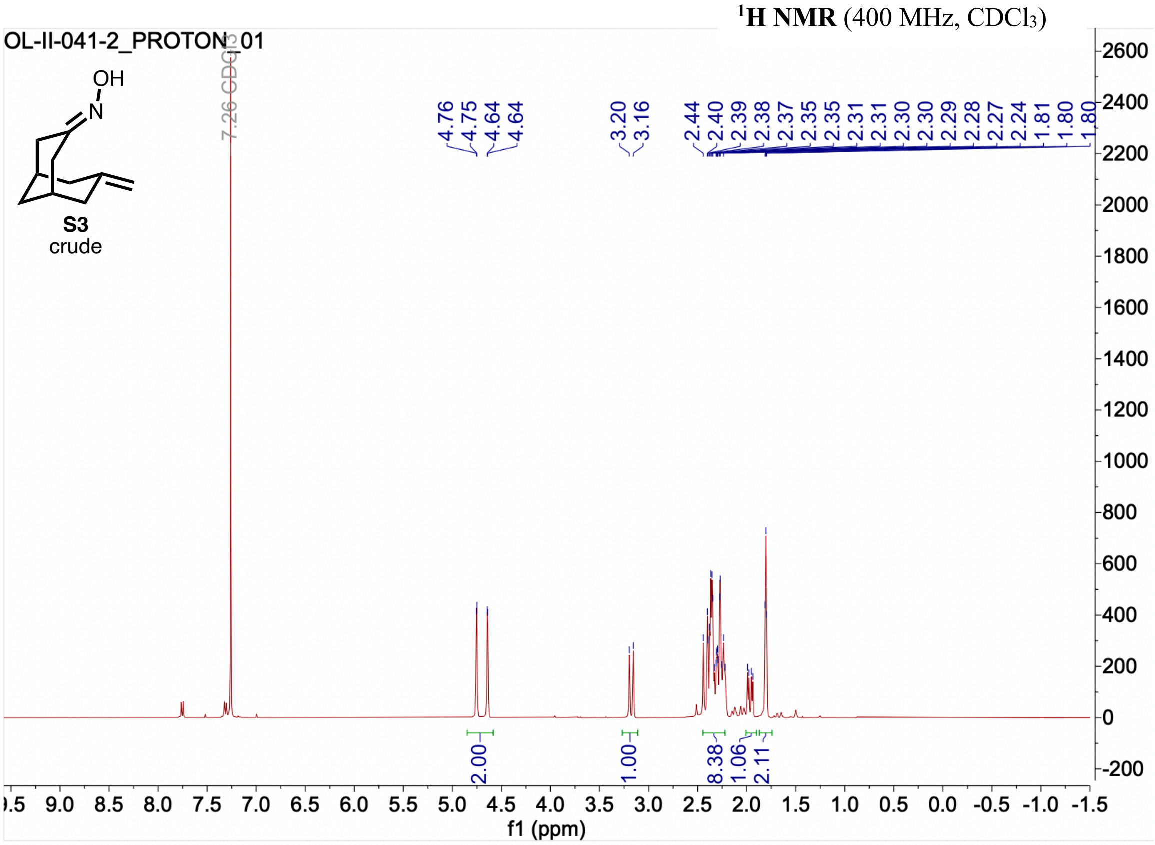

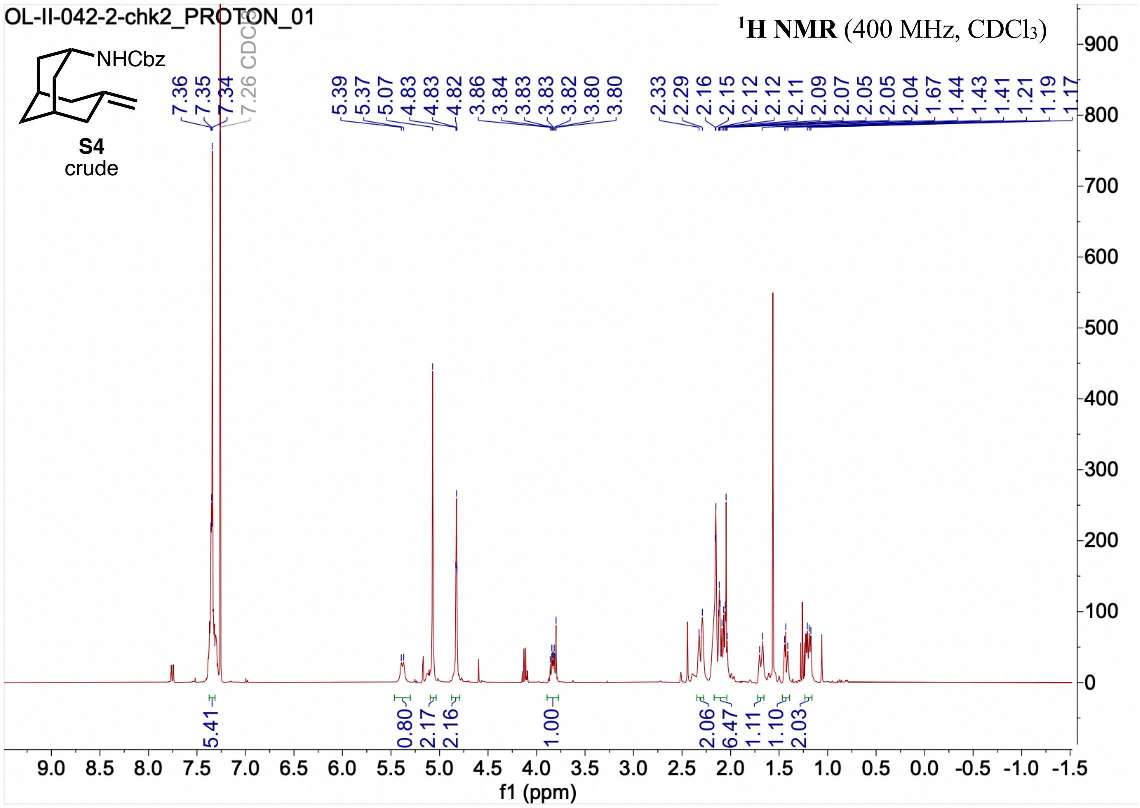

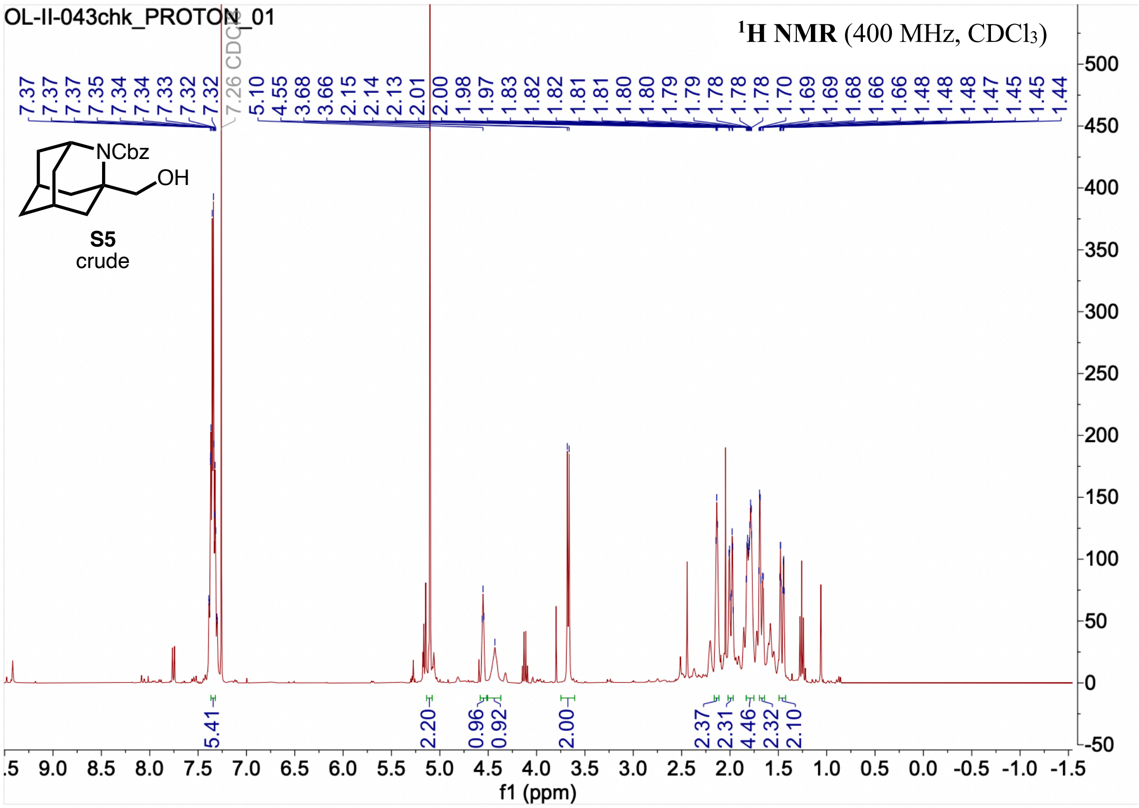
OL-II-043chk_PROTON_01
¹H NMR (400 MHz, CDCl₃)
NCbz
OH
S5
crude
f1 (ppm)

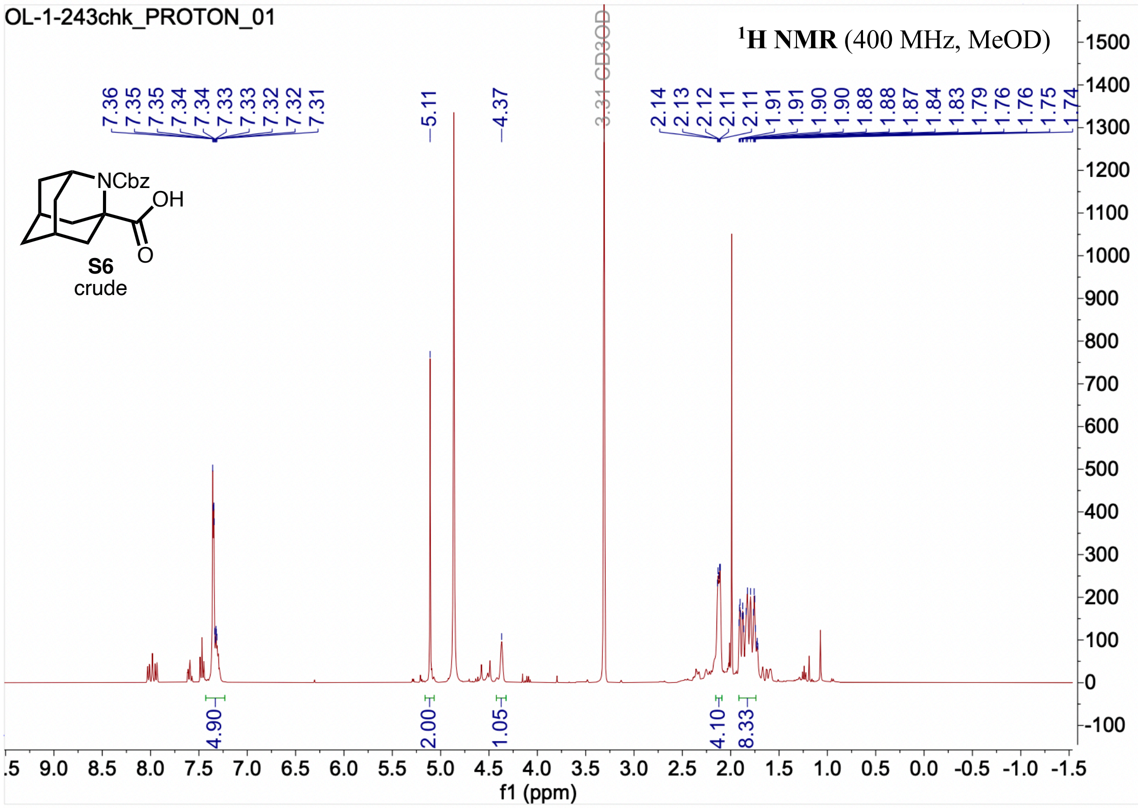
OL-1-243chk_PROTON_01
¹H NMR (400 MHz, MeOD)
NCbz
OH
O
S6
crude
f1 (ppm)

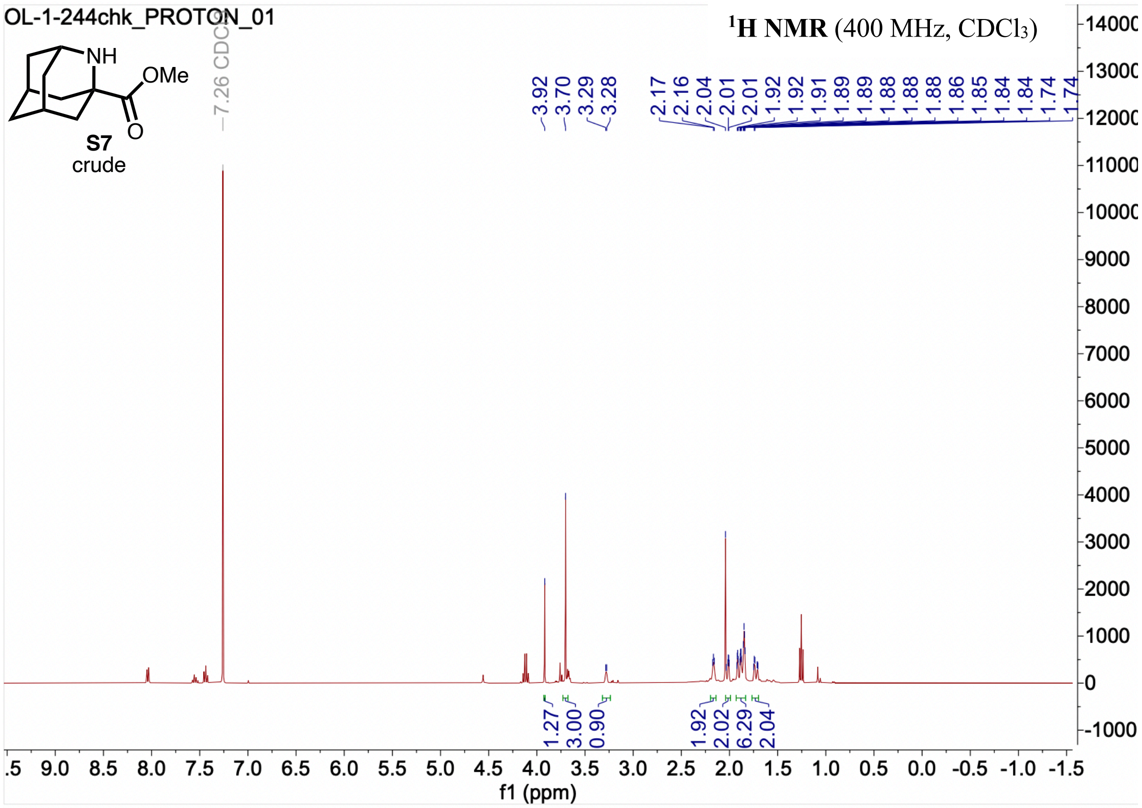
OL-1-244chk_PROTON_01
NH
OMe
O
S7
crude
7.26 CDCl3
^{1}H NMR (400 MHz, CDCl$_3$)
3.92
3.70
3.29
3.28
2.17
2.16
2.04
2.01
2.01
1.92
1.92
1.91
1.89
1.89
1.88
1.88
1.88
1.86
1.85
1.84
1.84
1.74
1.74
1.27
3.00
0.90
1.92
2.02
6.29
2.04
f1 (ppm)

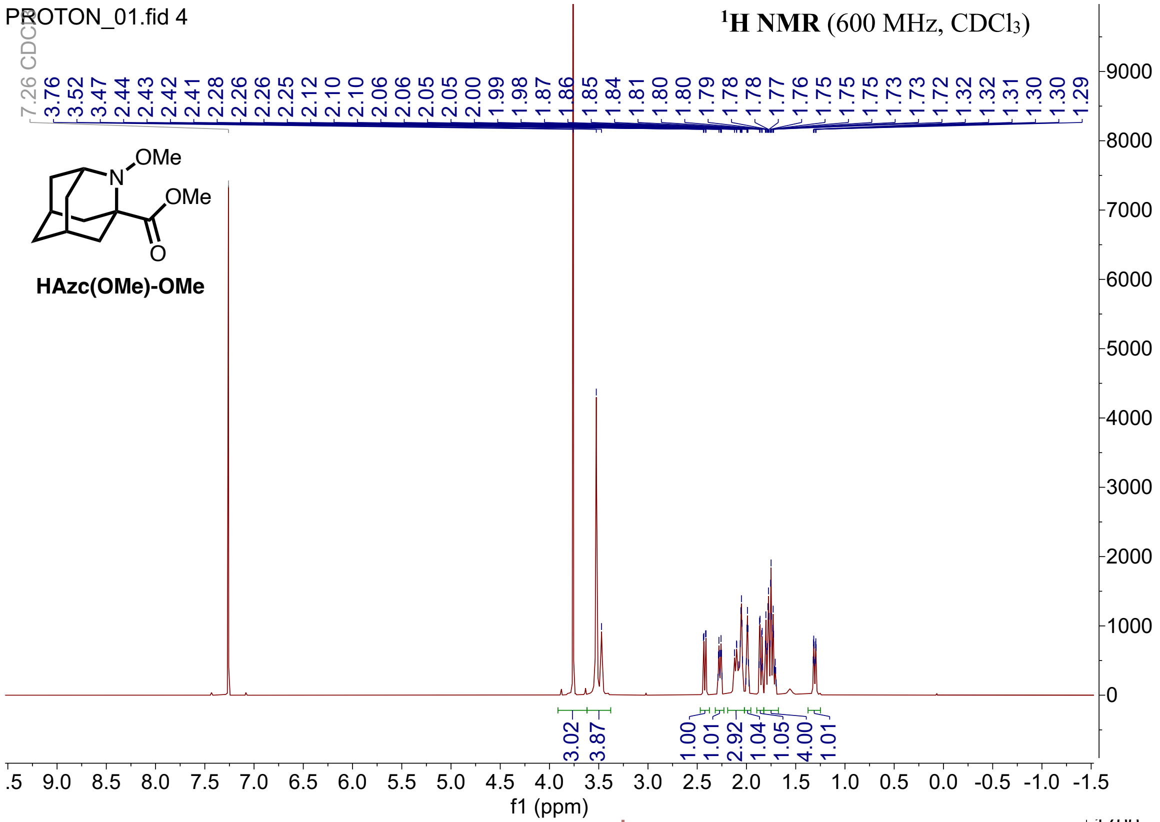
PROTON_01.fid 4
¹H NMR (600 MHz, CDCl₃)
HAzc(OMe)-OMe
7.26 CDCl3
3.76
3.52
3.47
2.44
2.43
2.42
2.41
2.28
2.26
2.26
2.25
2.12
2.10
2.10
2.06
2.06
2.05
2.05
2.00
1.99
1.98
1.87
1.86
1.85
1.84
1.81
1.80
1.80
1.79
1.78
1.78
1.77
1.76
1.75
1.75
1.75
1.73
1.73
1.72
1.32
1.32
1.31
1.30
1.30
1.29
3.02
3.87
1.00
1.01
2.92
1.04
1.05
4.00
1.01
f1 (ppm)

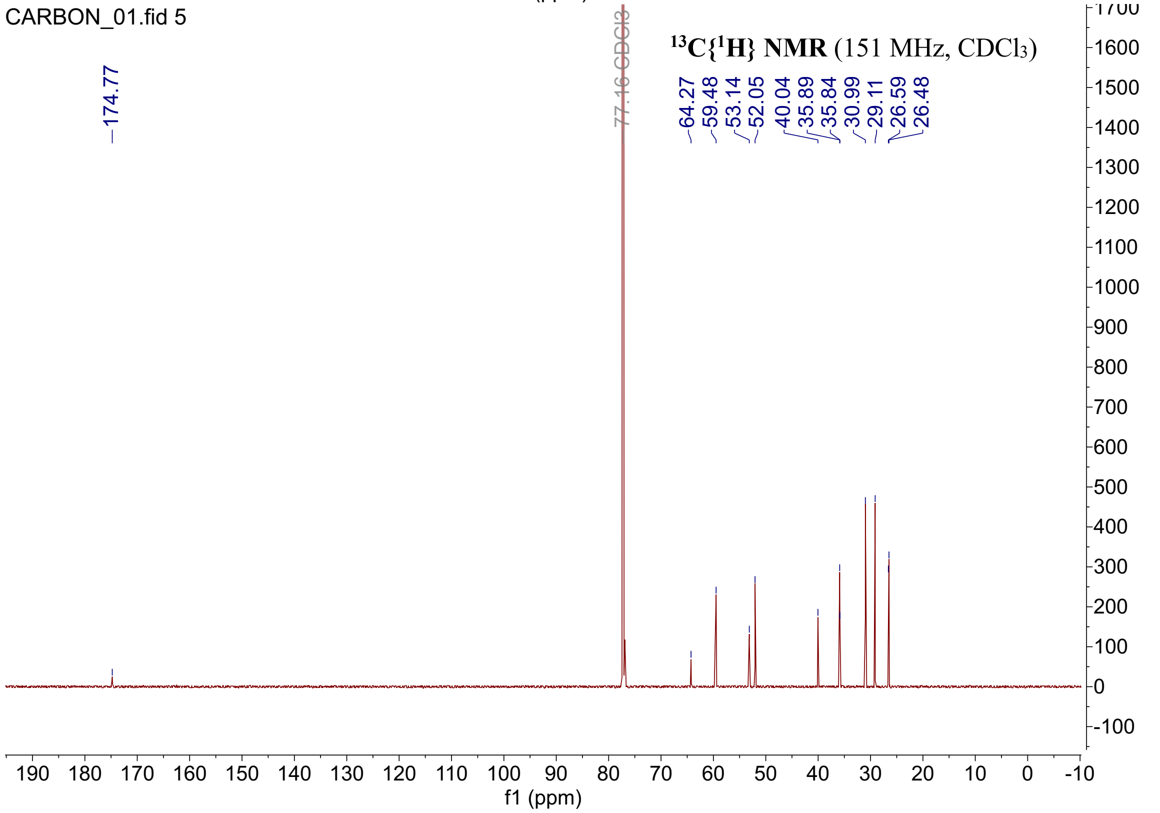
CARBON_01.fid 5
¹³C{¹H} NMR (151 MHz, CDCl₃)
174.77
77.16 CDCl3
64.27
59.48
53.14
52.05
40.04
35.89
35.84
30.99
29.11
26.59
26.48
f1 (ppm)

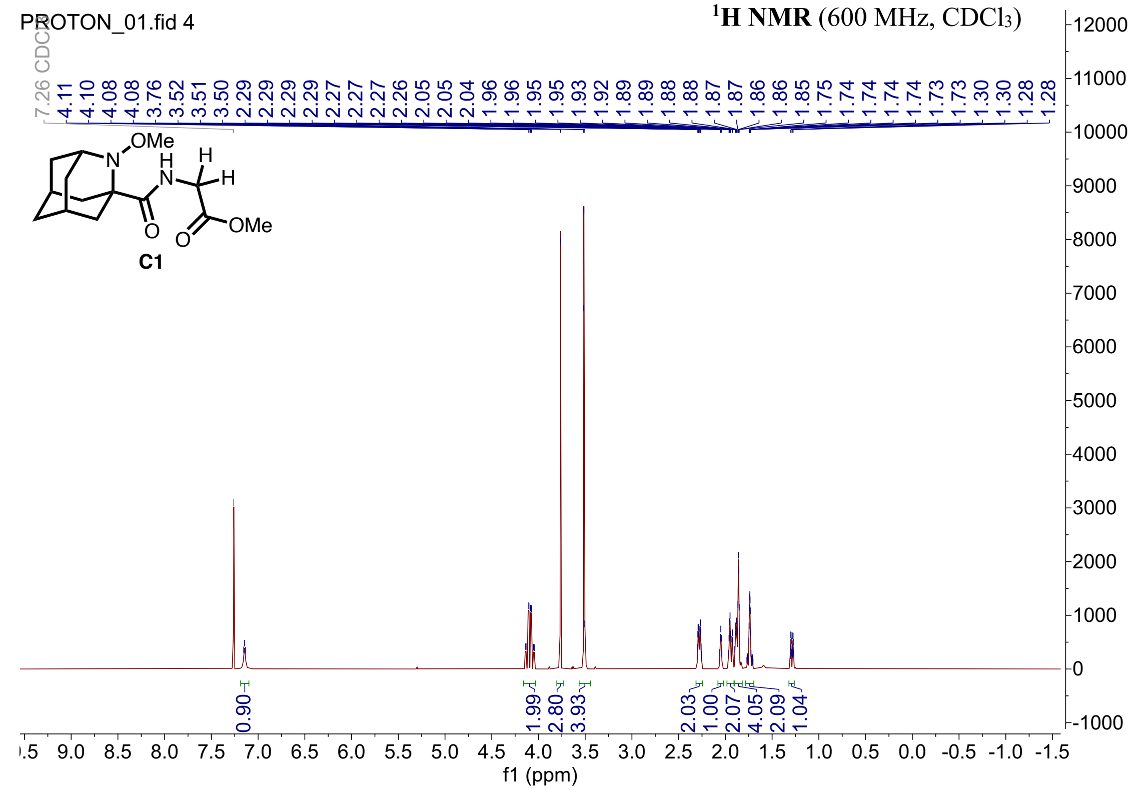
PROTON_01.fid 4
^{1}H NMR (600 MHz, CDCl$_3$)
7.26 CDCl3
4.11 4.10 4.08 4.08 3.76 3.52 3.51 3.50 2.29 2.29 2.29 2.29 2.27 2.27 2.27 2.26 2.05 2.05 2.04 1.96 1.96 1.95 1.95 1.93 1.92 1.89 1.89 1.88 1.88 1.87 1.87 1.86 1.86 1.85 1.75 1.74 1.74 1.74 1.74 1.73 1.73 1.30 1.30 1.28 1.28
OMe
N
H
N
H
H
O
O
OMe
C1
0.90
1.99
2.80
3.93
2.03
1.00
2.07
4.05
2.09
1.04
f1 (ppm)

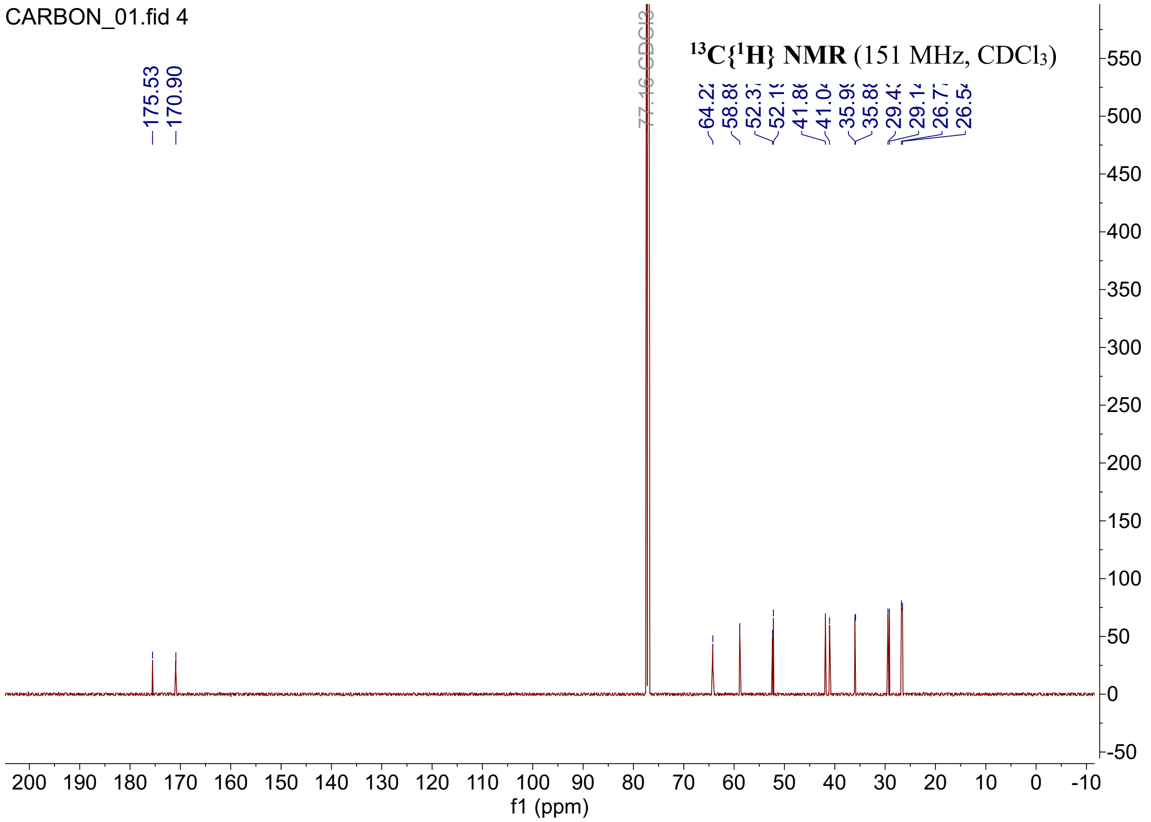
CARBON_01.fid 4
^{13}C{^{1}H} NMR (151 MHz, CDCl$_3$)
175.53
170.90
f1 (ppm)

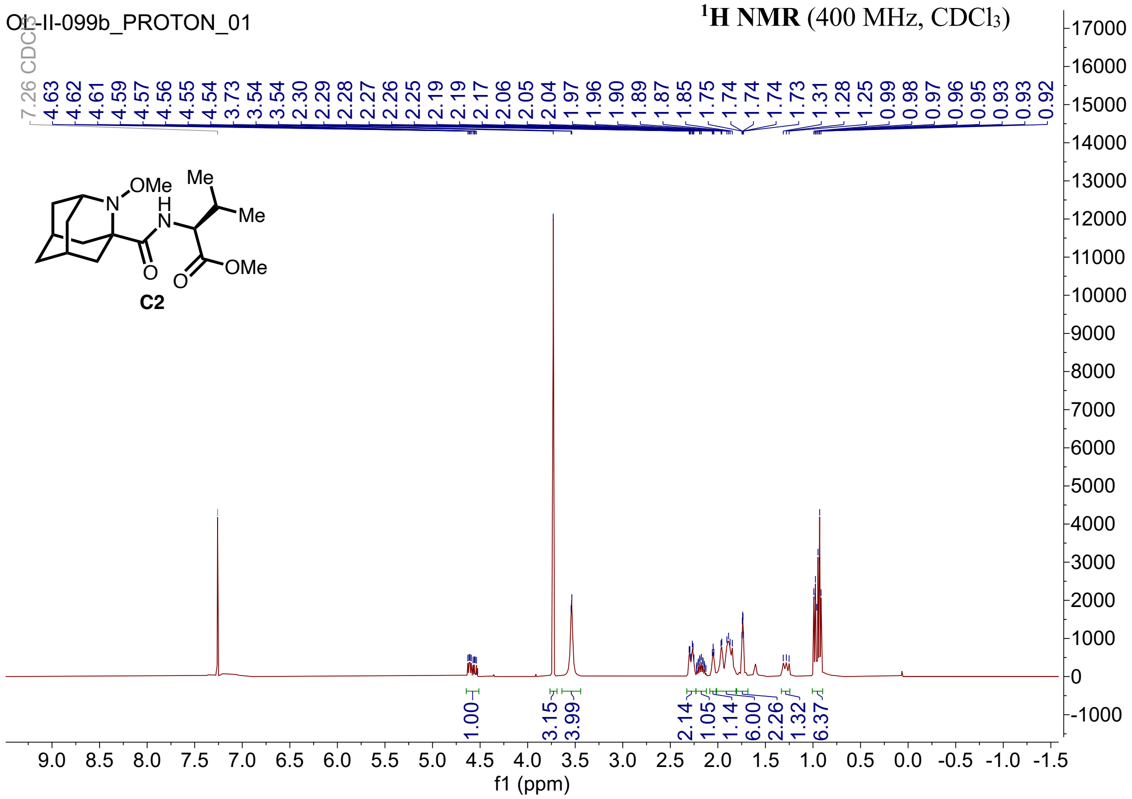
OL-II-099b_PROTON_01
1H NMR (400 MHz, CDCl3)
7.26 CDCl3
4.63 4.62 4.61 4.59 4.57 4.56 4.55 4.54 3.73 3.54 3.54 2.30 2.29 2.28 2.27 2.26 2.25 2.19 2.19 2.17 2.06 2.05 2.04 1.97 1.96 1.90 1.89 1.87 1.85 1.75 1.74 1.74 1.74 1.73 1.31 1.28 1.25 0.99 0.98 0.97 0.96 0.95 0.93 0.93 0.92
OMe
N
H
N
Me
Me
O
O
OMe
C2
1.00
3.15
3.99
2.14
1.05
1.14
6.00
2.26
1.32
6.37
f1 (ppm)

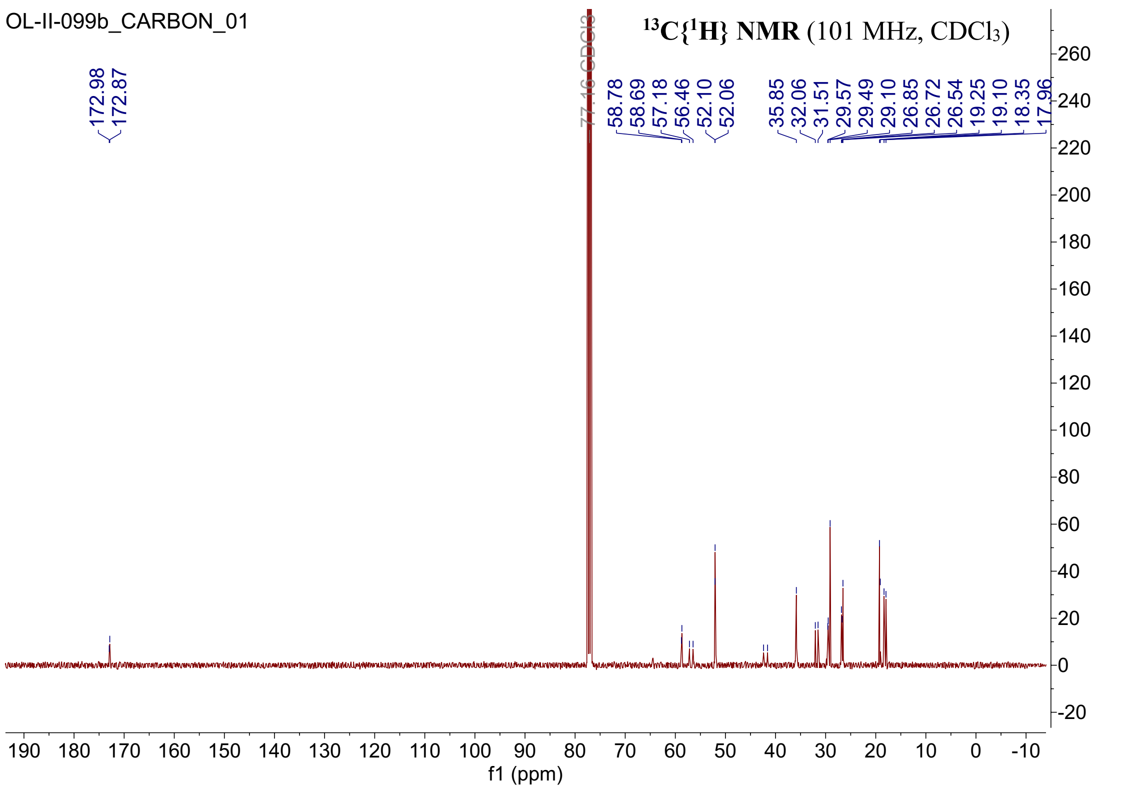
OL-II-099b_CARBON_01
13C{1H} NMR (101 MHz, CDCl3)
172.98 172.87
77.16 CDCl3
58.78 58.69 57.18 56.46 52.10 52.06
35.85 32.06 31.51 29.57 29.49 29.10 26.85 26.72 26.54 19.25 19.10 18.35 17.96
f1 (ppm)

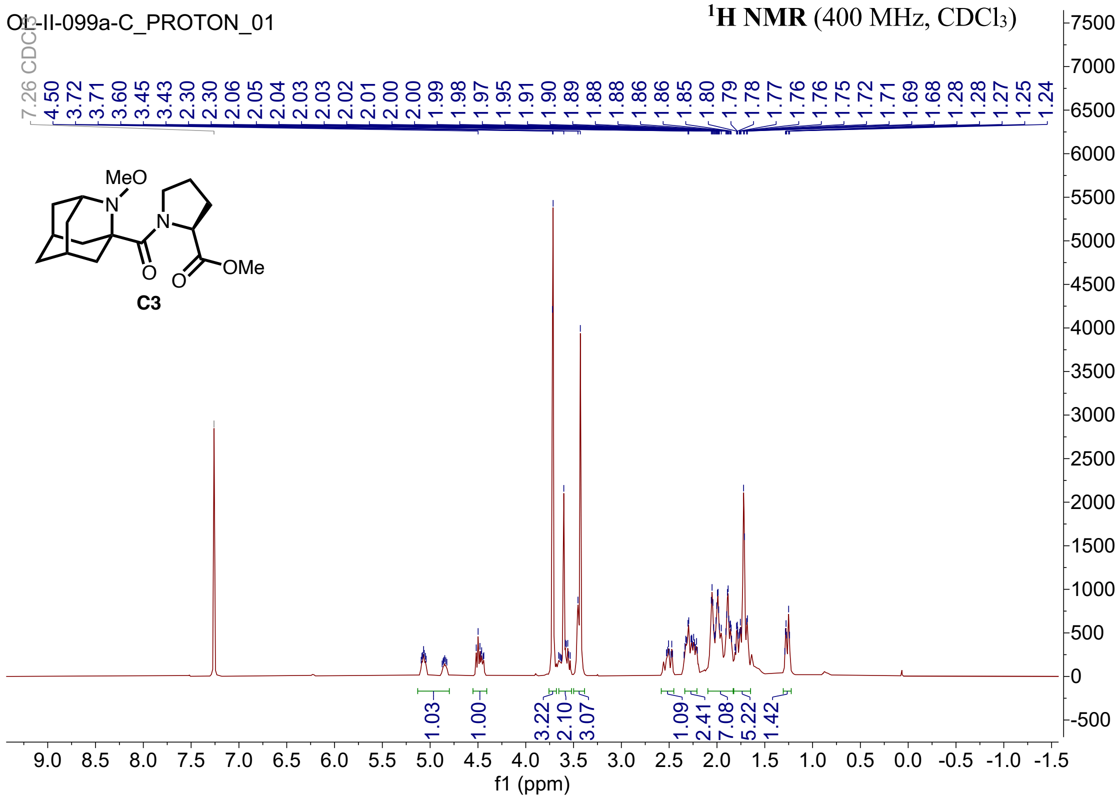
OL-II-099a-C_PROTON_01
1H NMR (400 MHz, CDCl3)
7.26 CDCl3
4.50 3.72 3.71 3.60 3.45 3.43 2.30 2.30 2.06 2.05 2.04 2.03 2.03 2.02 2.01 2.00 2.00 1.99 1.98 1.97 1.95 1.91 1.90 1.89 1.88 1.88 1.86 1.86 1.85 1.80 1.79 1.78 1.77 1.76 1.76 1.75 1.72 1.71 1.69 1.68 1.28 1.28 1.27 1.25 1.24
MeO
N
N
O
O
OMe
C3
1.03 1.00 3.22 2.10 3.07 1.09 2.41 7.08 5.22 1.42
f1 (ppm)

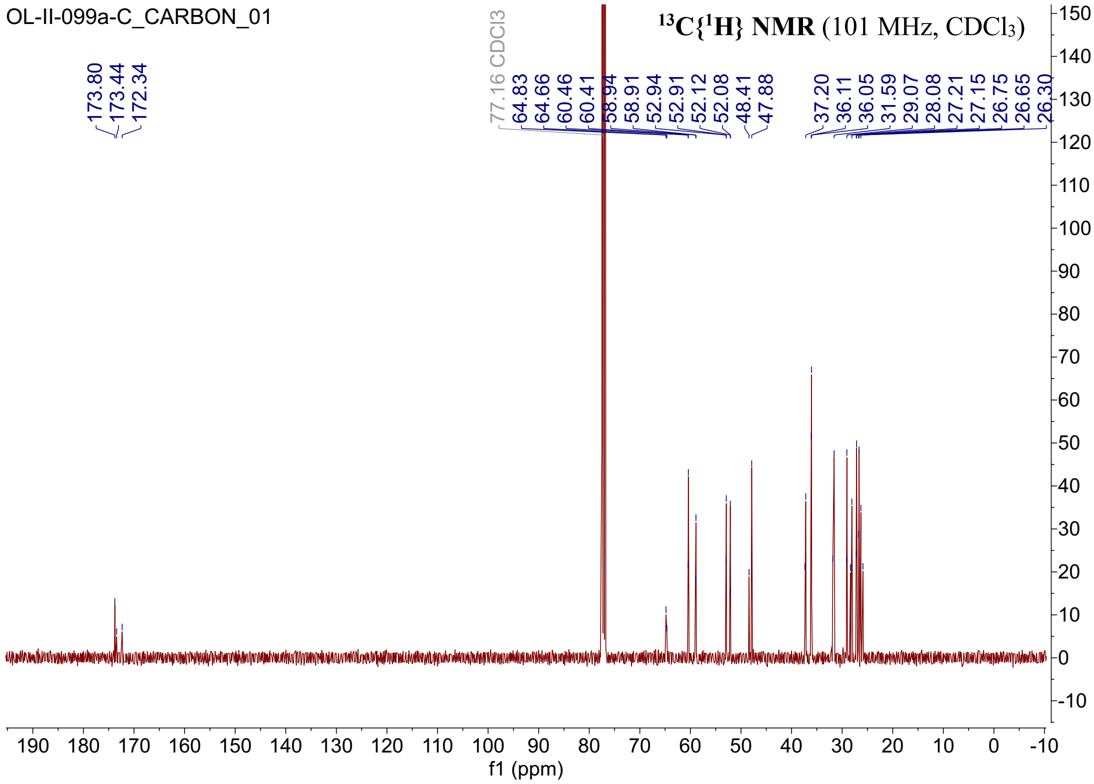
OL-II-099a-C_CARBON_01
13C{1H} NMR (101 MHz, CDCl3)
173.80 173.44 172.34
77.16 CDCl3
64.83 64.66 60.46 60.41 58.94 58.91 52.94 52.91 52.12 52.08 48.41 47.88
37.20 36.11 36.05 31.59 29.07 28.08 27.21 27.15 26.75 26.65 26.30
f1 (ppm)

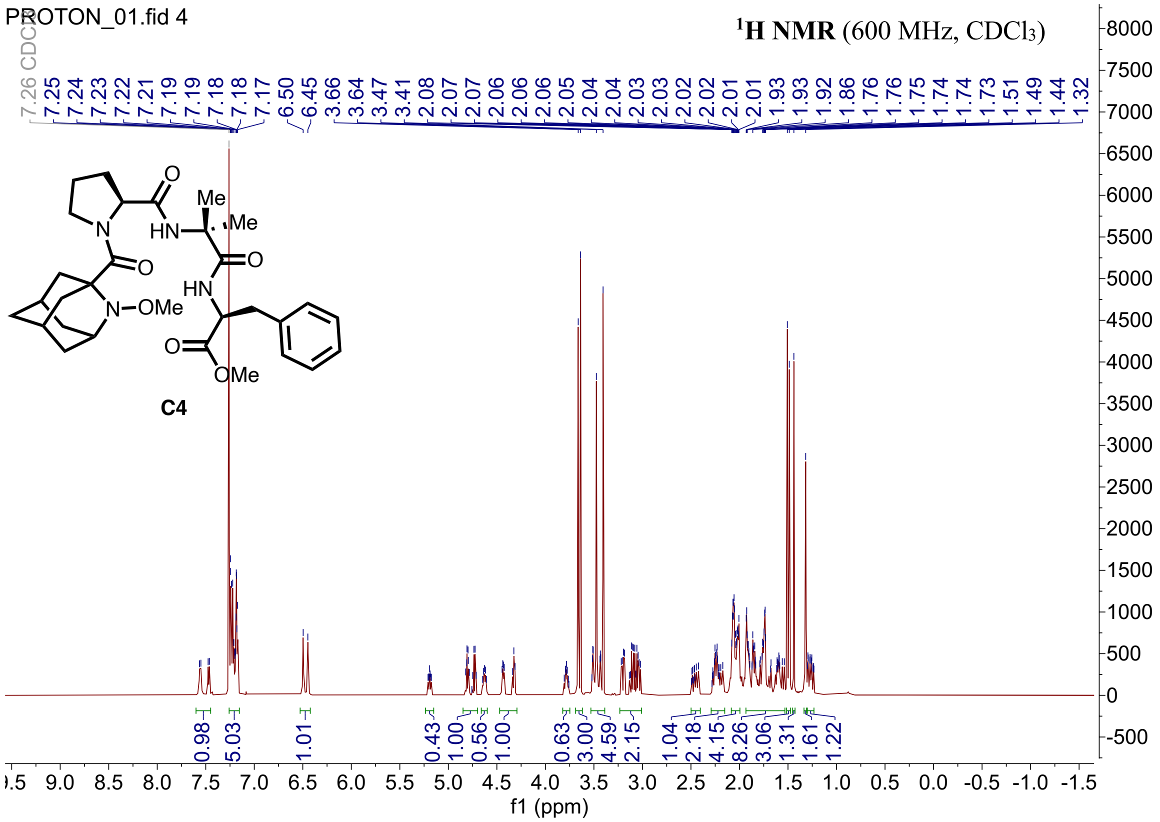
PROTON_01.fid 4
¹H NMR (600 MHz, CDCl₃)
7.26 CDCl3
7.25 7.24 7.23 7.22 7.21 7.19 7.19 7.18 7.18 7.17 6.50 6.45 3.66 3.64 3.47 3.41 2.08 2.07 2.07 2.06 2.06 2.06 2.05 2.04 2.04 2.03 2.03 2.02 2.02 2.01 2.01 1.93 1.93 1.92 1.86 1.76 1.76 1.75 1.74 1.74 1.73 1.51 1.49 1.44 1.32
0.98 5.03 1.01 0.43 1.00 0.56 1.00 0.63 3.00 4.59 2.15 1.04 2.18 4.15 8.26 3.06 1.31 1.61 1.22
Me
Me
HN
O
N
O
HN
O
N–OMe
O
OMe
C4
f1 (ppm)

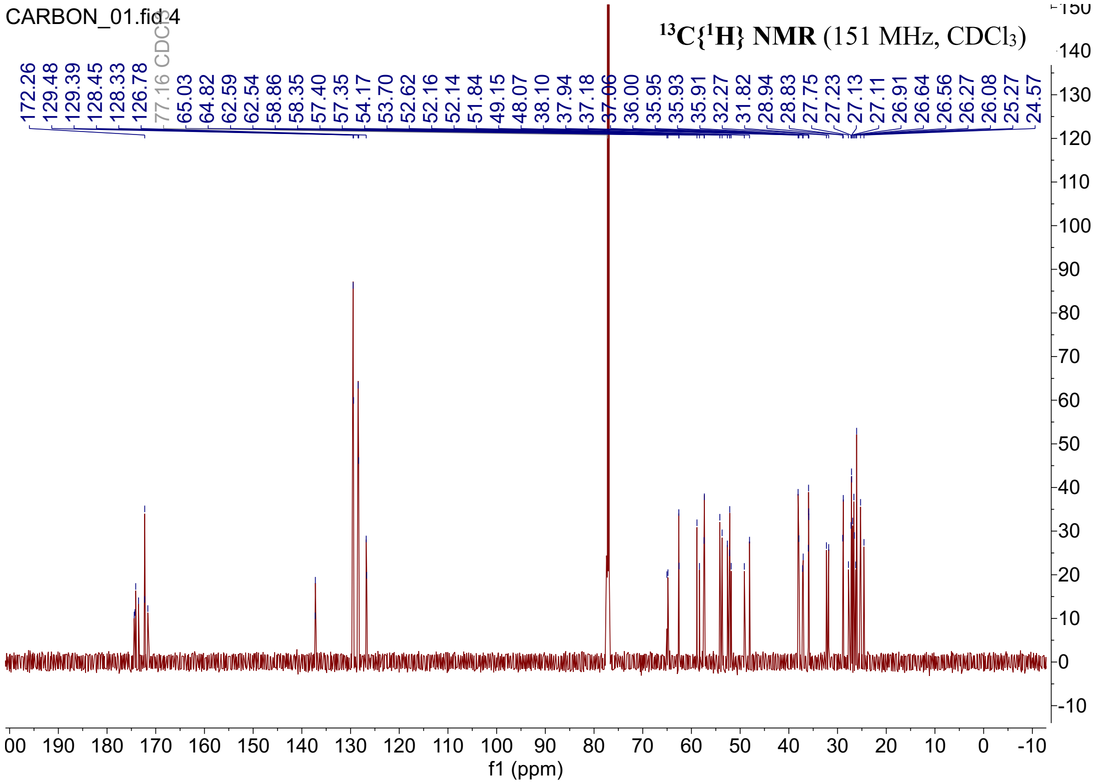
CARBON_01.fid 4
¹³C{¹H} NMR (151 MHz, CDCl₃)
77.16 CDCl3
172.26 129.48 129.39 128.45 128.33 126.78 65.03 64.82 62.59 62.54 58.86 58.35 57.40 57.35 54.17 53.70 52.62 52.16 52.14 51.84 49.15 48.07 38.10 37.94 37.18 37.06 36.00 35.95 35.93 35.91 32.27 31.82 28.94 28.83 27.75 27.23 27.13 27.11 26.91 26.64 26.56 26.27 26.08 25.27 24.57
f1 (ppm)

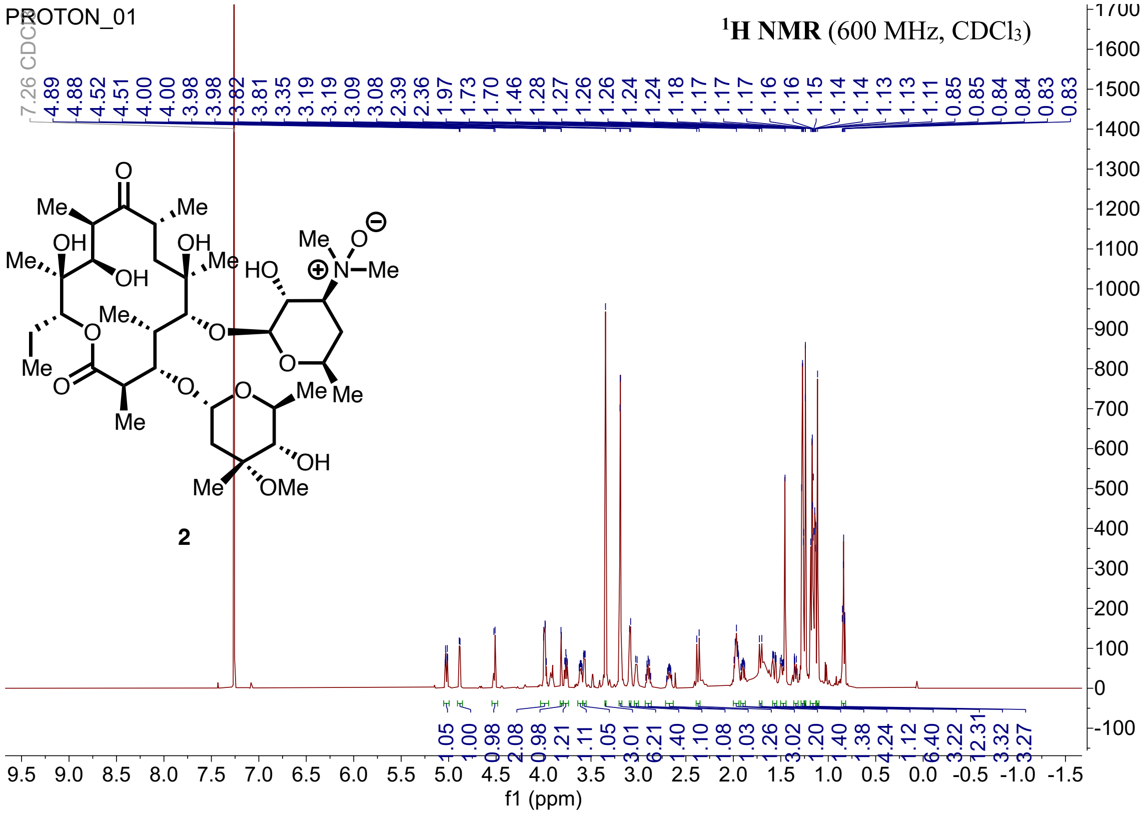
PROTON_01
¹H NMR (600 MHz, CDCl₃)
7.26 CDCl3
4.89 4.88 4.52 4.51 4.00 4.00 3.98 3.98 3.82 3.81 3.35 3.19 3.19 3.09 3.08 2.39 2.36 1.97 1.73 1.70 1.46 1.28 1.27 1.26 1.26 1.24 1.24 1.18 1.17 1.17 1.17 1.16 1.16 1.15 1.14 1.14 1.13 1.13 1.11 0.85 0.85 0.84 0.84 0.83 0.83
1.05 1.00 0.98 2.08 0.98 1.21 1.11 1.05 3.01 6.21 1.40 1.10 1.08 1.03 1.26 3.02 1.20 1.40 1.38 4.24 1.12 6.40 3.22 12.31 3.32 3.27
Me
OH
Me
OH
Me
OH
HO
O
Me
N
Me
O
O
Me
2
f1 (ppm)

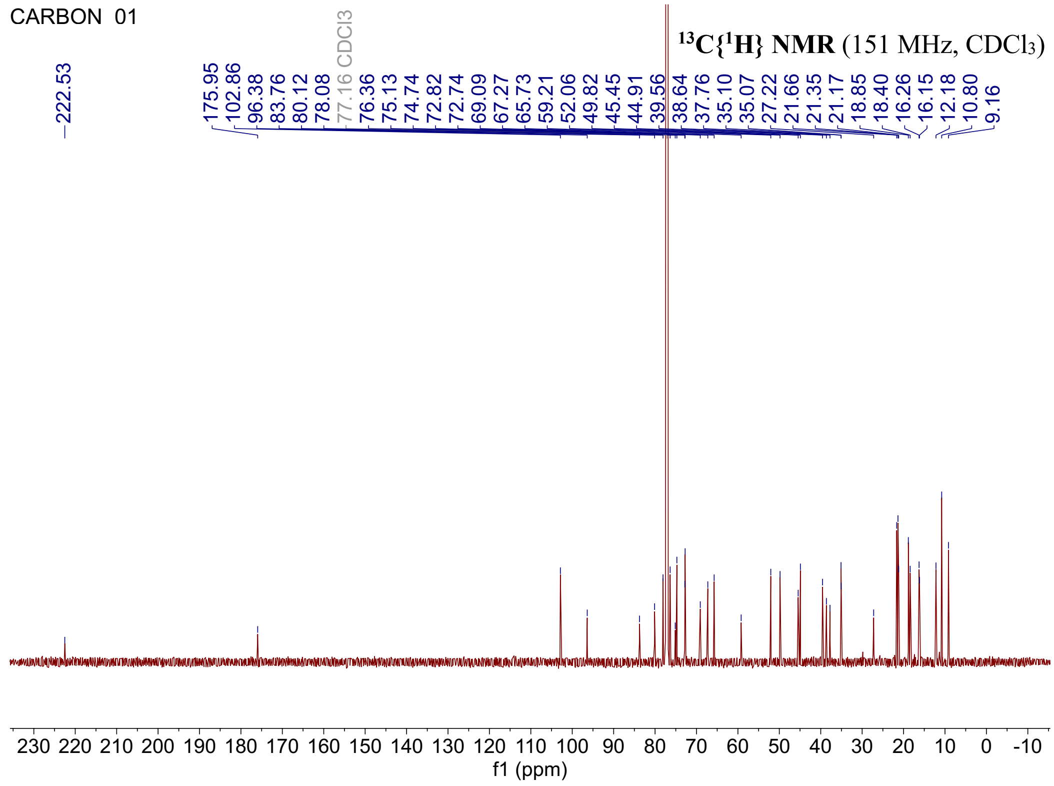
CARBON_01
¹³C{¹H} NMR (151 MHz, CDCl₃)
222.53
175.95 102.86 96.38 83.76 80.12 78.08 77.16 CDCl3 76.36 75.13 74.74 72.82 72.74 69.09 67.27 65.73 59.21 52.06 49.82 45.45 44.91 39.56 38.64 37.76 35.10 35.07 27.22 21.66 21.35 21.17 18.85 18.40 16.26 16.15 12.18 10.80 9.16
f1 (ppm)

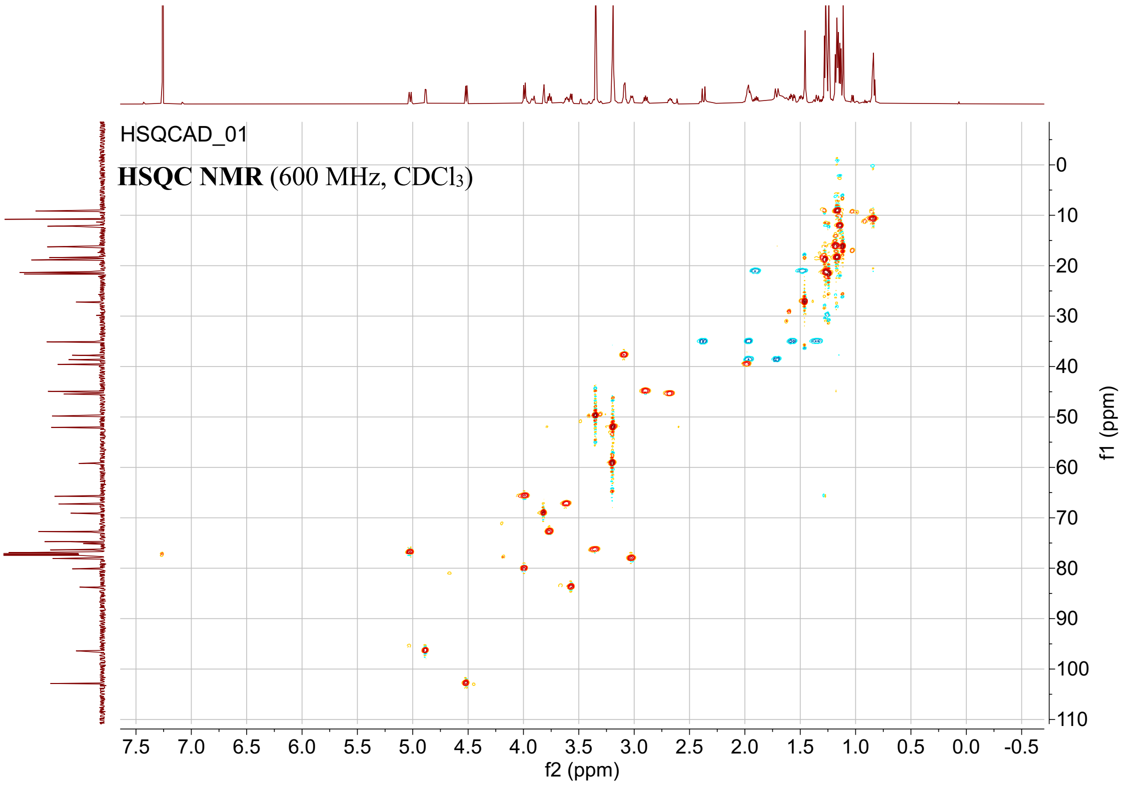

HSQCAD_01
HSQC NMR (600 MHz, CDCl3)
f1 (ppm)
f2 (ppm)

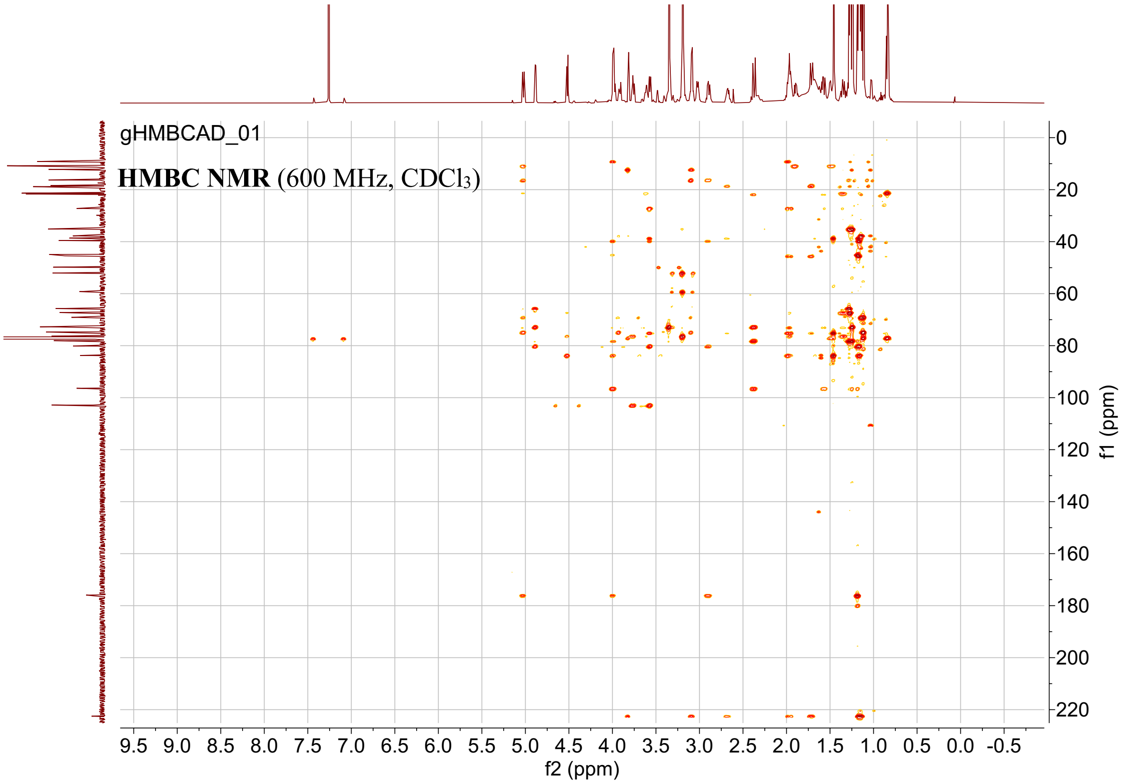

gHMBCAD_01
HMBC NMR (600 MHz, CDCl3)
f1 (ppm)
f2 (ppm)

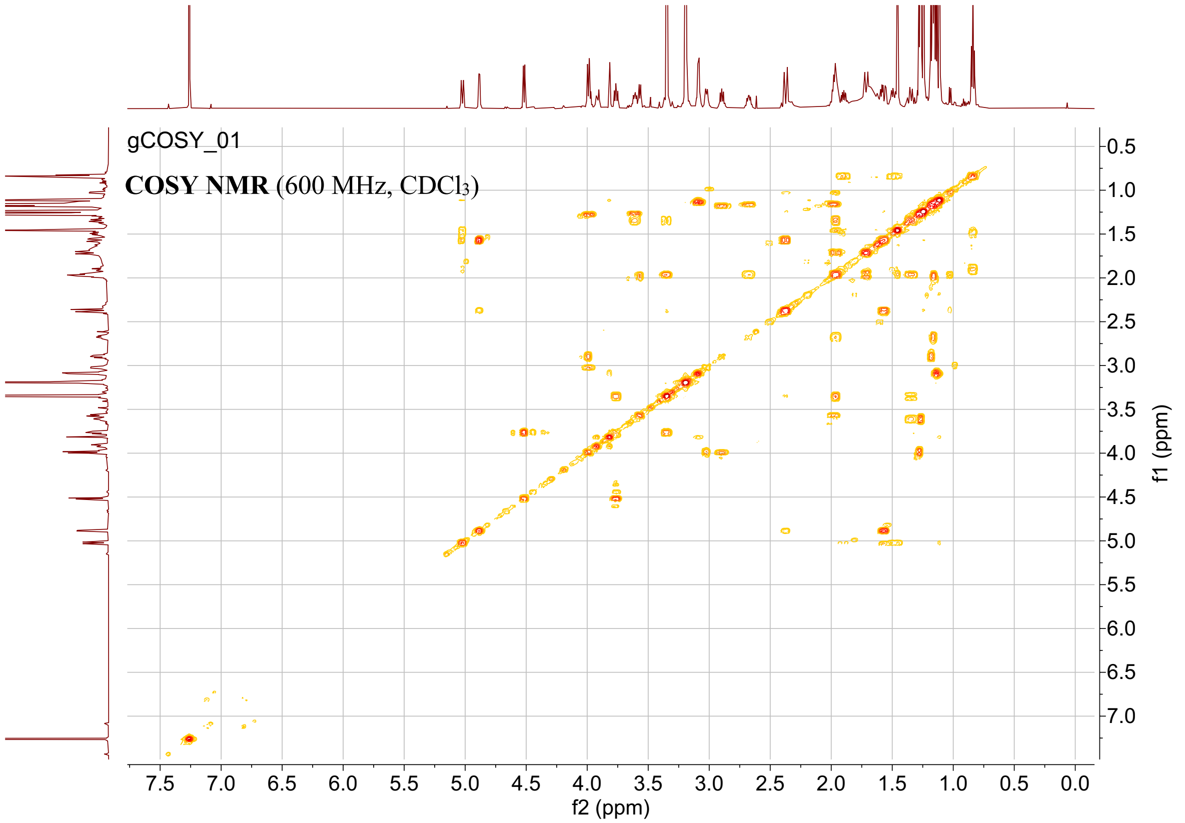
gCOSY_01
COSY NMR (600 MHz, CDCl3)
f2 (ppm)
f1 (ppm)

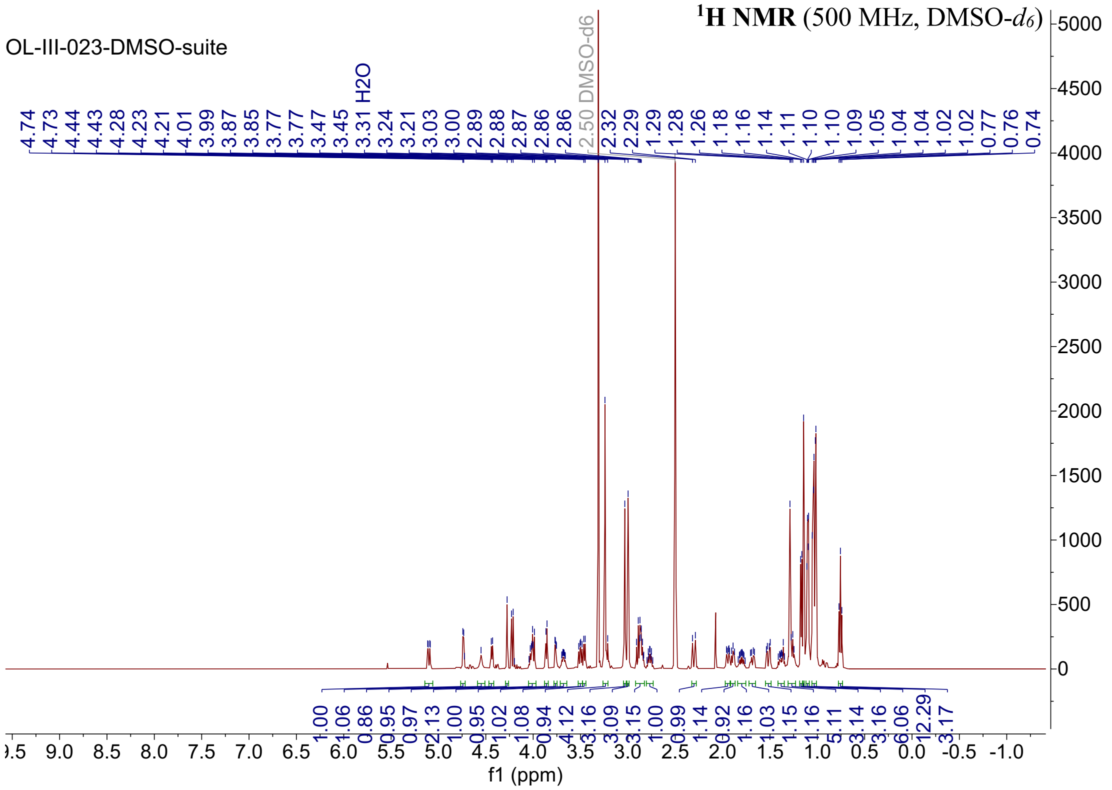
OL-III-023-DMSO-suite
1H NMR (500 MHz, DMSO-d6)
H2O
DMSO-d6
f1 (ppm)

$^{13}C\{^1H\}$ NMR (151 MHz, DMSO-d_6)

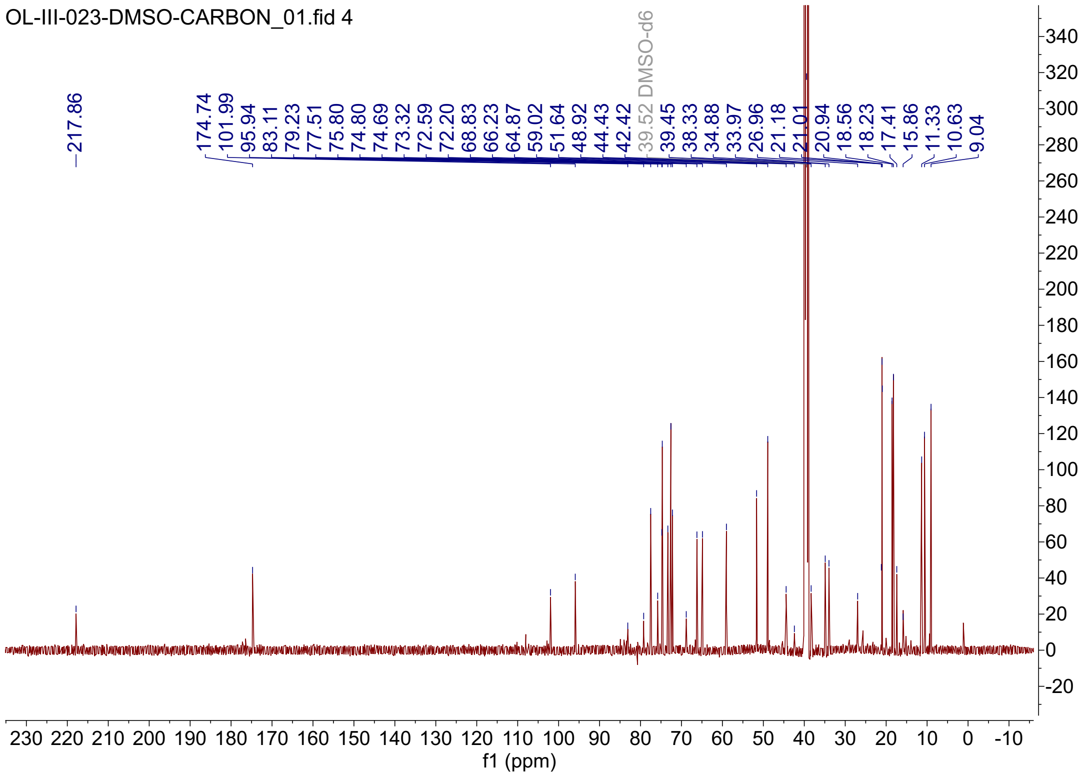

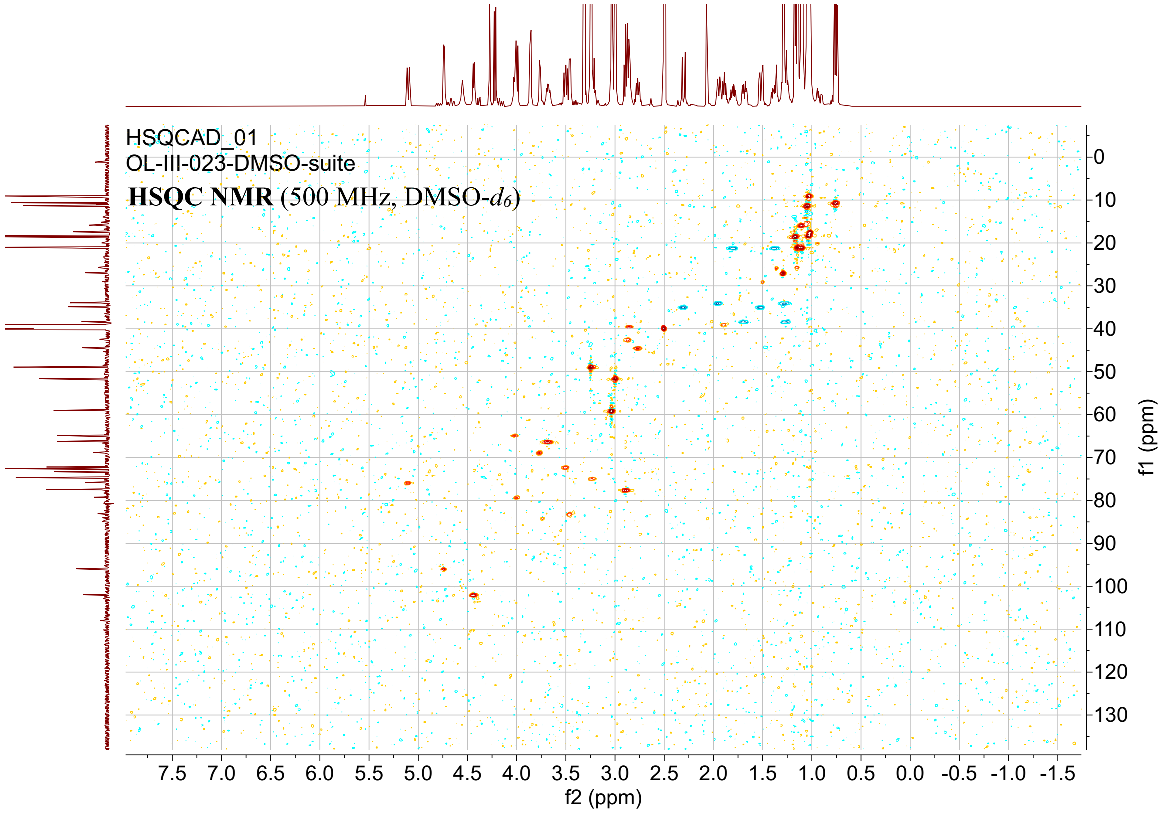

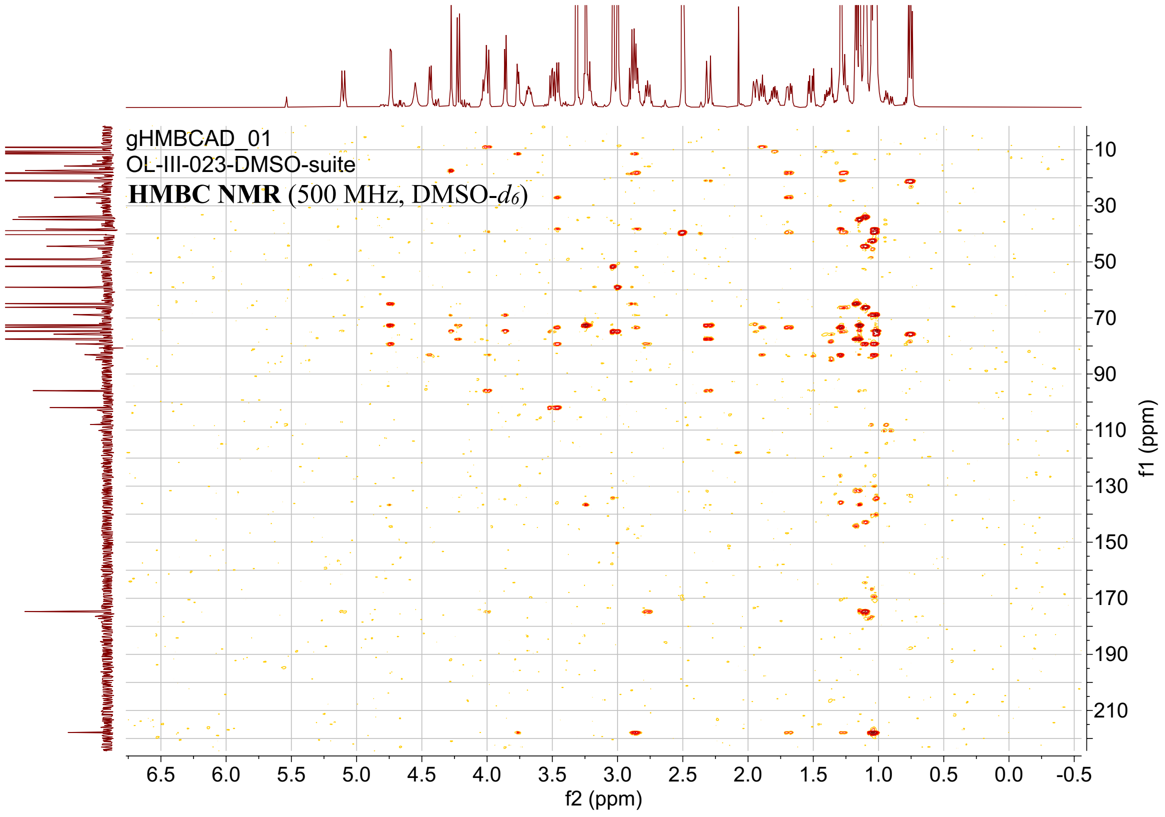
gHMBCAD_01
OL-III-023-DMSO-suite
HMBC NMR (500 MHz, DMSO-d_6)
f2 (ppm)
f1 (ppm)

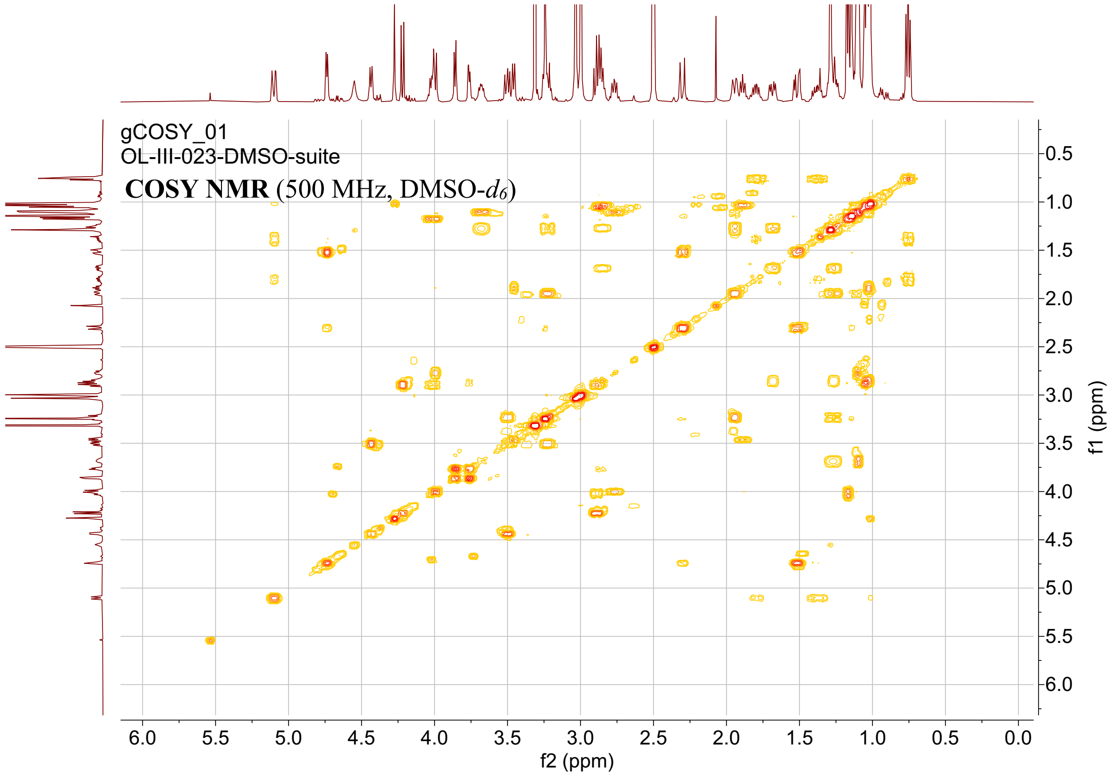
gCOSY_01
OL-III-023-DMSO-suite
COSY NMR (500 MHz, DMSO-d_6)
f2 (ppm)
f1 (ppm)

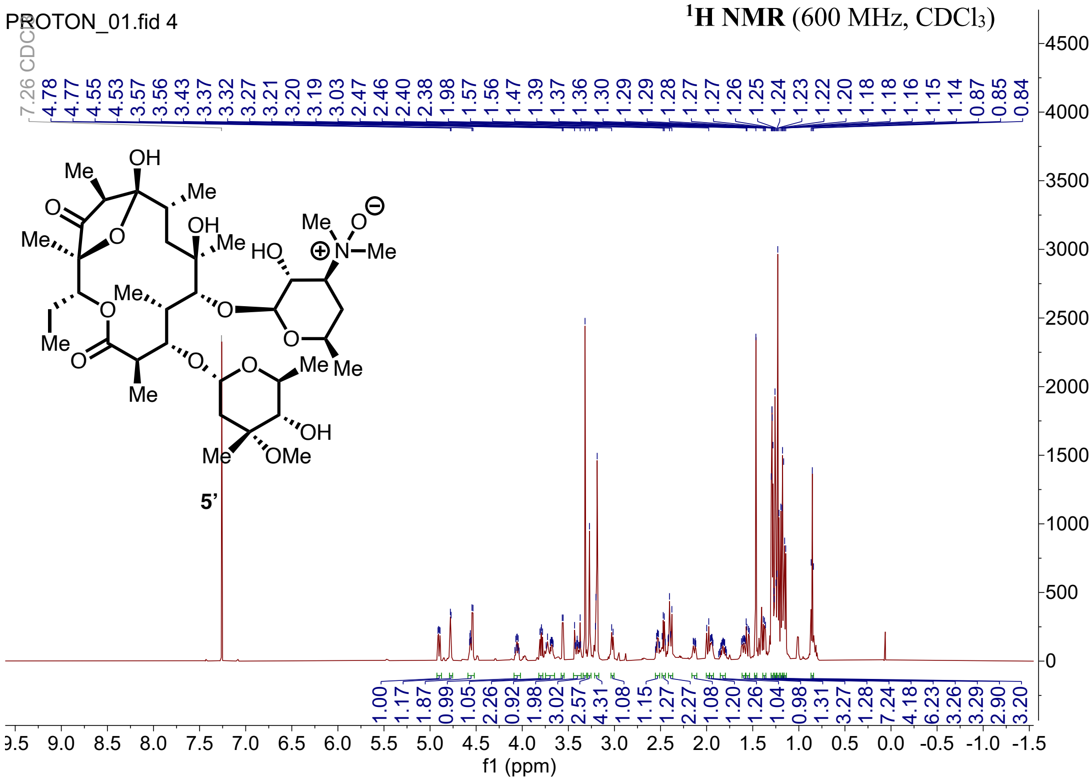
PROTON_01.fid 4
1H NMR (600 MHz, CDCl3)
7.26 CDCl3
4.78 4.77 4.55 4.53 3.57 3.56 3.43 3.37 3.32 3.27 3.21 3.20 3.19 3.03 2.47 2.46 2.40 2.38 1.98 1.57 1.56 1.47 1.39 1.37 1.36 1.30 1.29 1.29 1.28 1.27 1.27 1.26 1.25 1.24 1.23 1.22 1.20 1.18 1.18 1.16 1.15 1.14 0.87 0.85 0.84
1.00 1.17 1.87 0.99 1.05 2.26 0.92 1.98 3.02 2.57 4.31 1.08 1.15 1.27 2.27 1.08 1.20 1.26 1.04 0.98 1.31 3.27 1.28 7.24 4.18 6.23 3.26 3.29 2.90 3.20
5'
f1 (ppm)

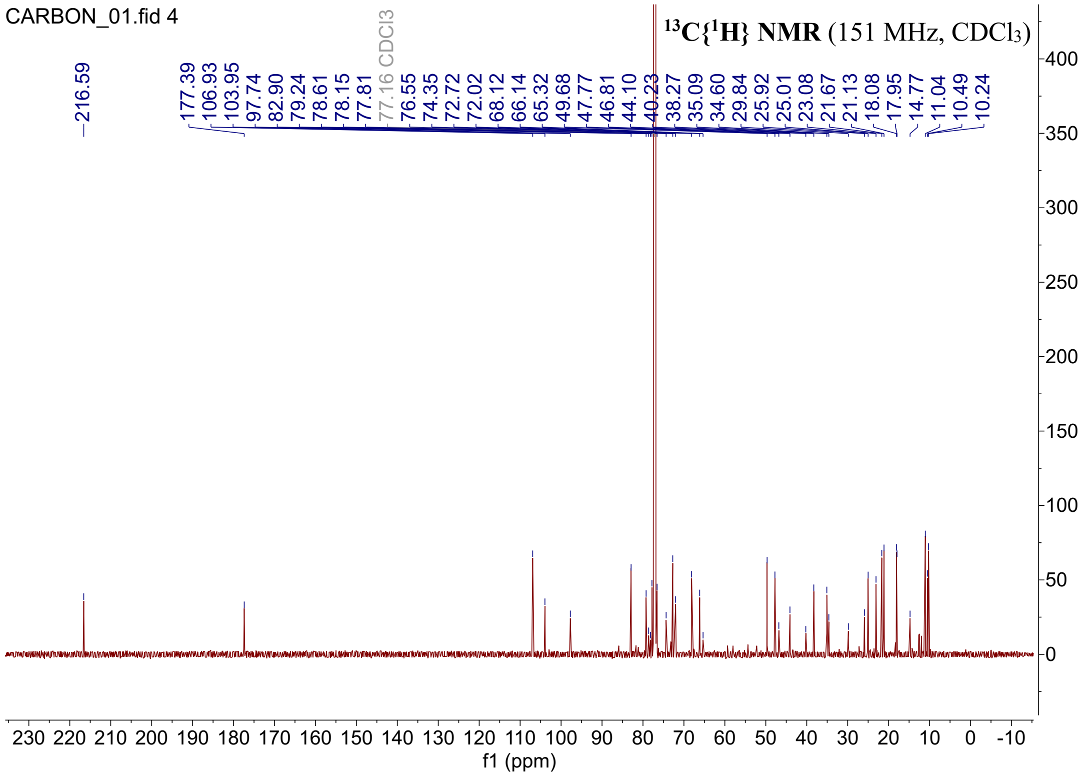
CARBON_01.fid 4
13C{1H} NMR (151 MHz, CDCl3)
216.59
177.39 106.93 103.95 97.74 82.90 79.24 78.61 78.15 77.81 77.16 CDCl3 76.55 74.35 72.72 72.02 68.12 66.14 65.32 49.68 47.77 46.81 44.10 40.23 38.27 35.09 34.60 29.84 25.92 25.01 23.08 21.67 21.13 18.08 17.95 14.77 11.04 10.49 10.24
f1 (ppm)

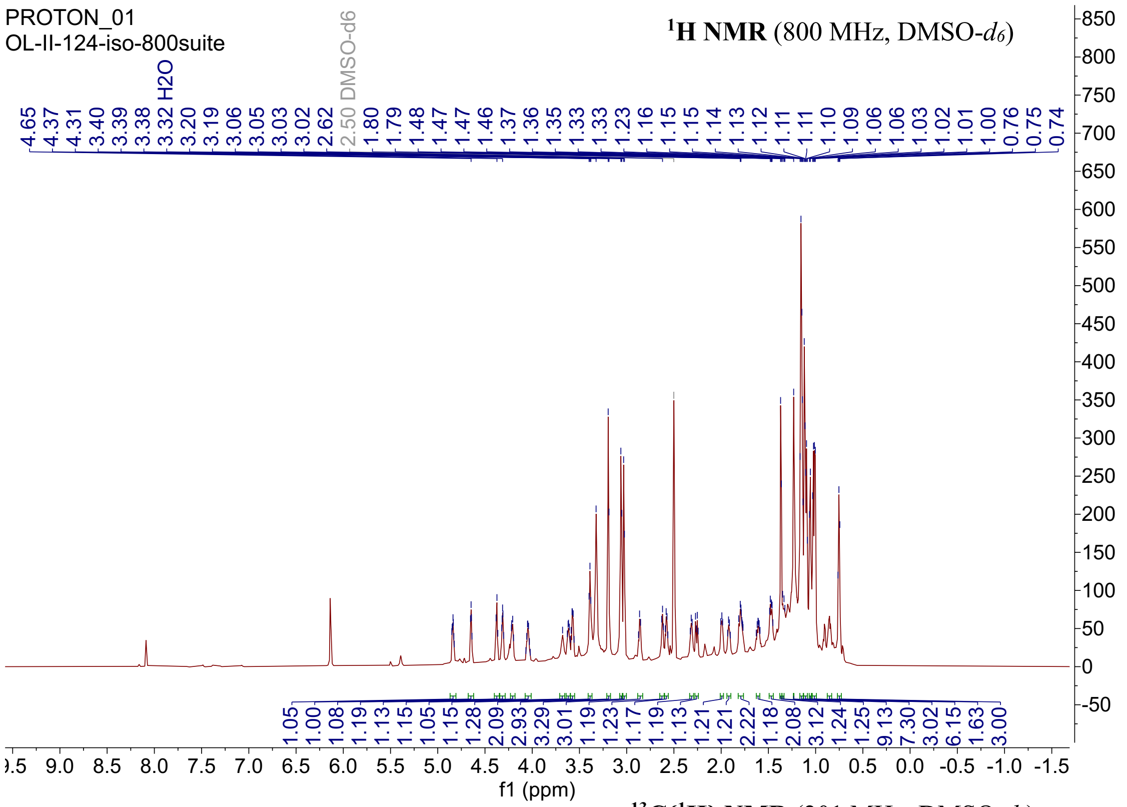
PROTON_01
OL-II-124-iso-800suite
^{1}H NMR (800 MHz, DMSO-d_6)
f1 (ppm)

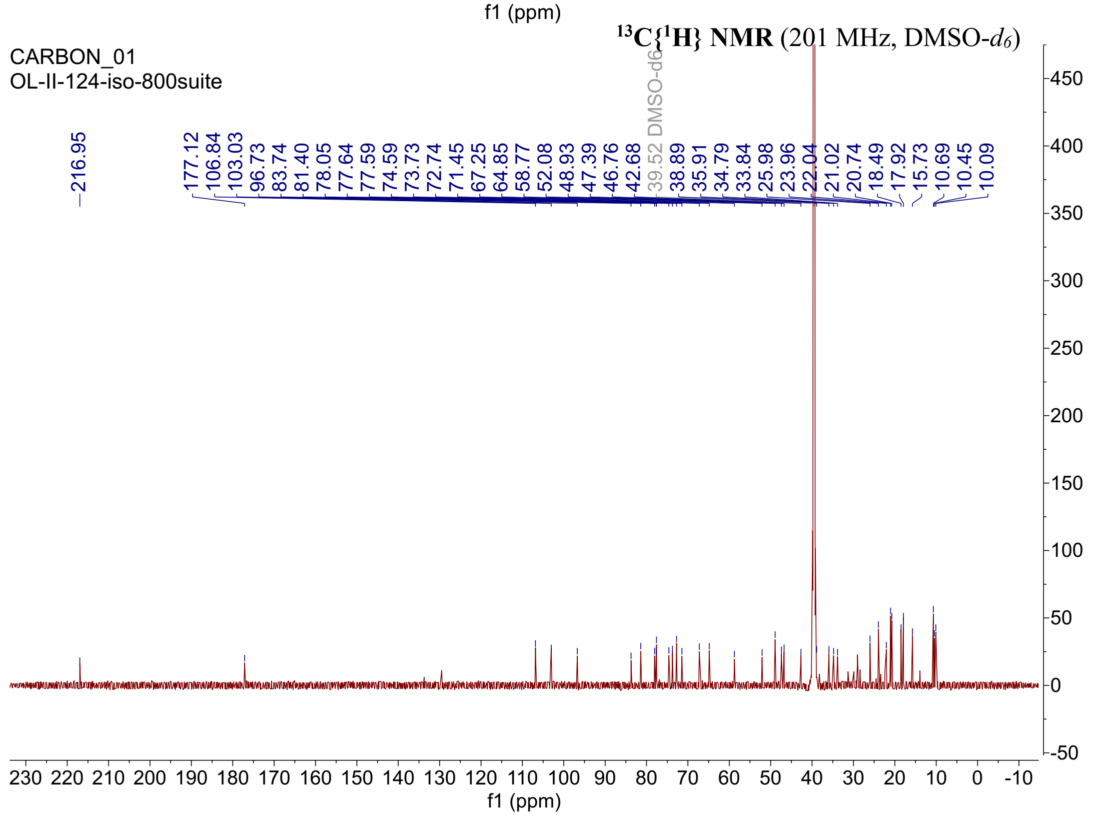
^{13}C{^{1}H} NMR (201 MHz, DMSO-d_6)
CARBON_01
OL-II-124-iso-800suite
f1 (ppm)

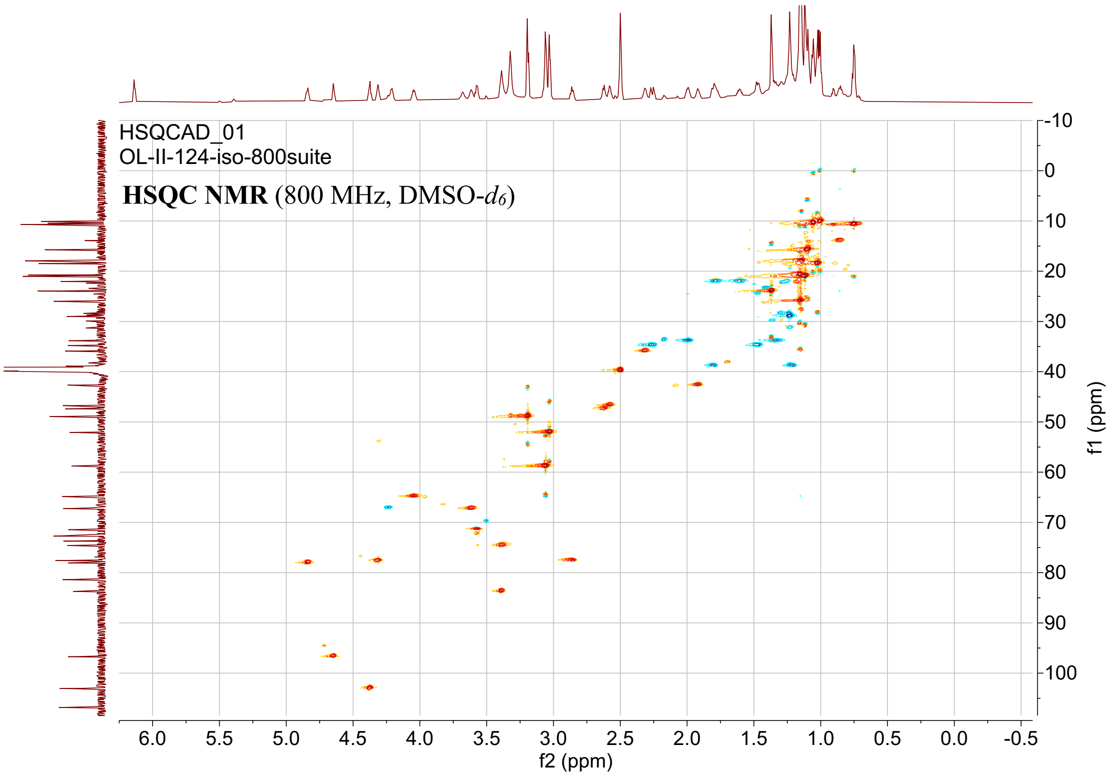
HSQCAD_01
OL-II-124-iso-800suite
HSQC NMR (800 MHz, DMSO-d_6)
f2 (ppm)
f1 (ppm)

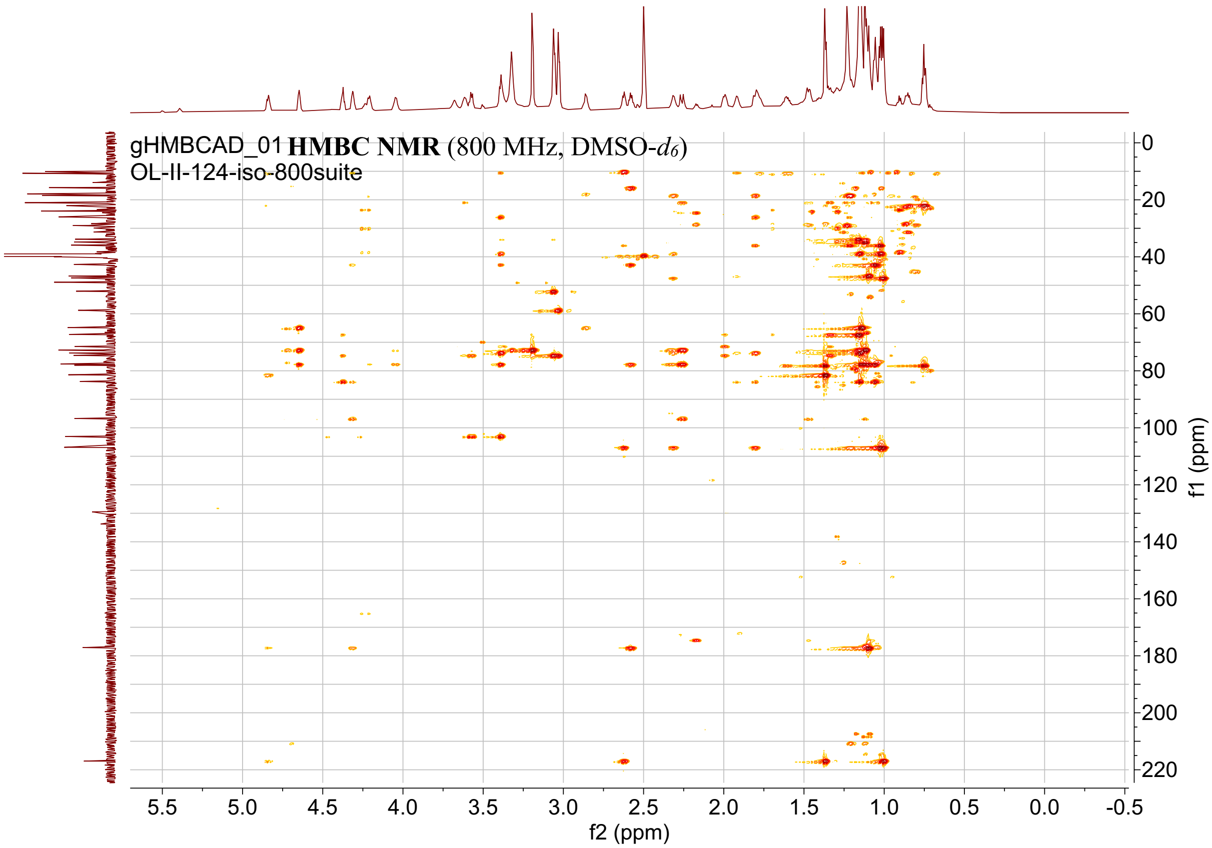
gHMBCAD_01 HMBC NMR (800 MHz, DMSO-d_6)
OL-II-124-iso-800suite
f2 (ppm)
f1 (ppm)

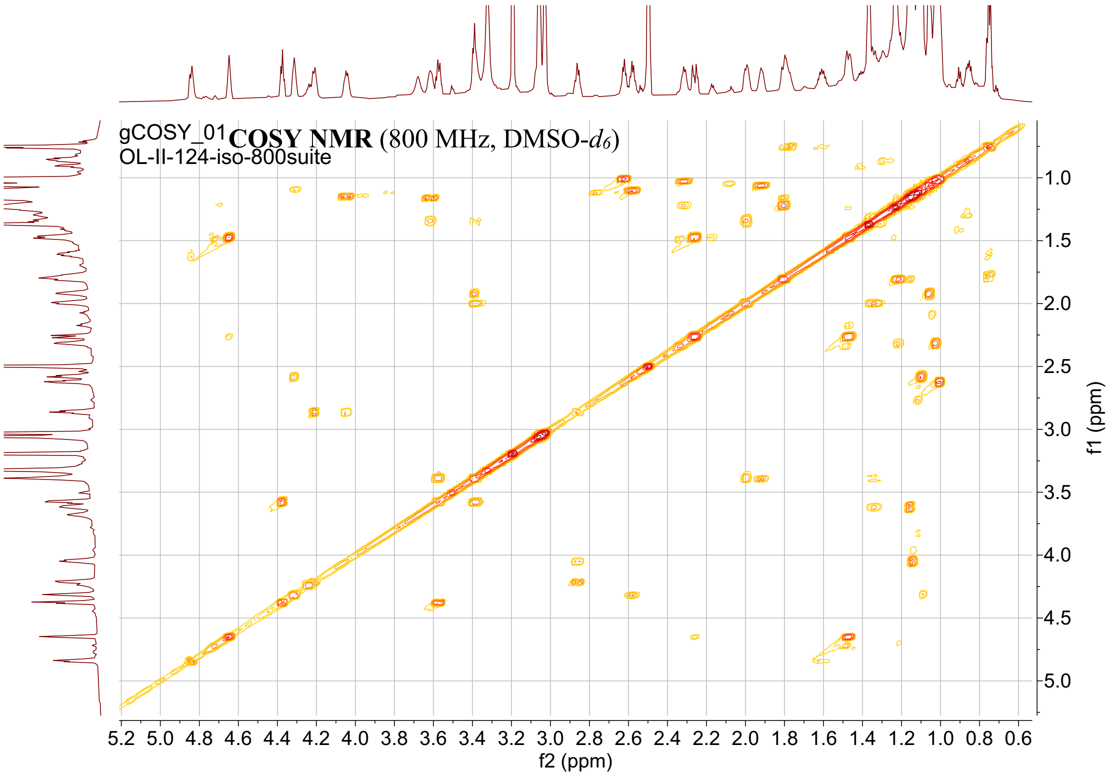
gCOSY_01
COSY NMR (800 MHz, DMSO-d_6)
OL-II-124-iso-800suite
f2 (ppm)
f1 (ppm)

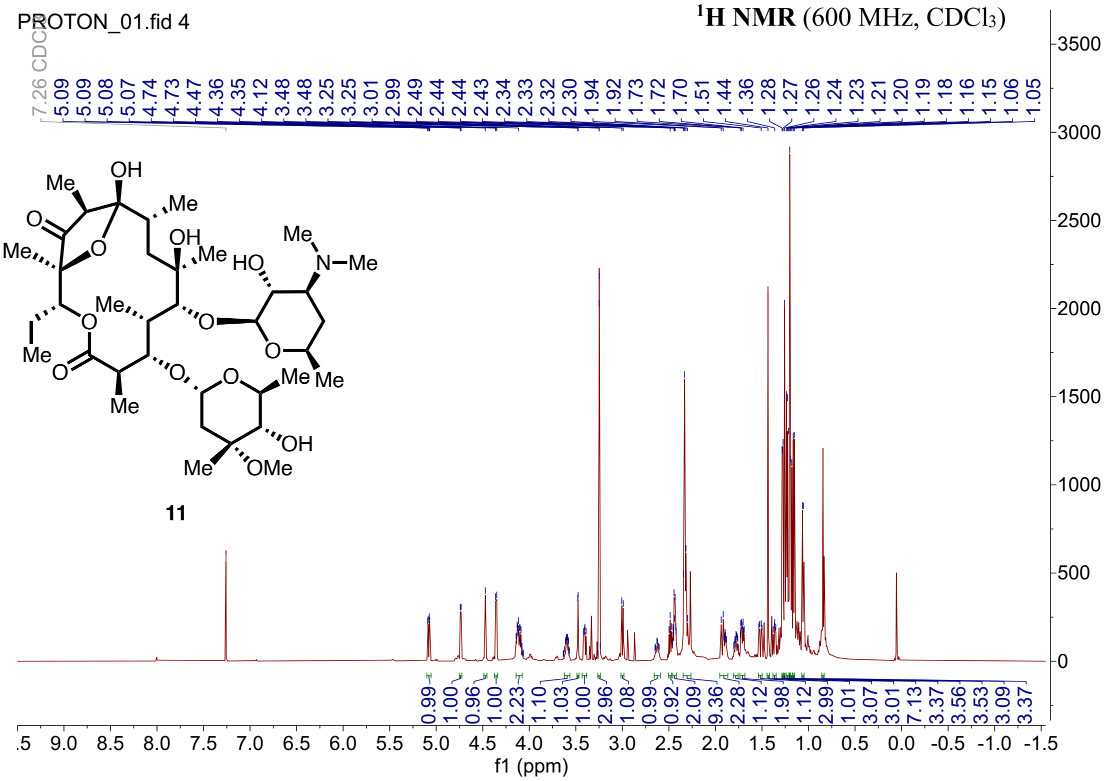
PROTON_01.fid 4
1H NMR (600 MHz, CDCl3)
7.26 CDCl3
5.09 5.09 5.08 5.07 4.74 4.73 4.47 4.36 4.35 4.12 3.48 3.48 3.25 3.25 3.01 2.99 2.49 2.44 2.44 2.43 2.34 2.33 2.32 2.30 1.94 1.92 1.73 1.72 1.70 1.51 1.44 1.36 1.28 1.27 1.26 1.24 1.23 1.21 1.20 1.19 1.18 1.16 1.15 1.06 1.05
0.99 1.00 0.96 1.00 2.23 1.10 1.03 1.00 2.96 1.08 0.99 0.92 2.09 9.36 2.28 1.12 1.98 1.12 2.99 1.01 3.07 3.01 7.13 3.37 3.56 3.53 3.09 3.37
11
f1 (ppm)

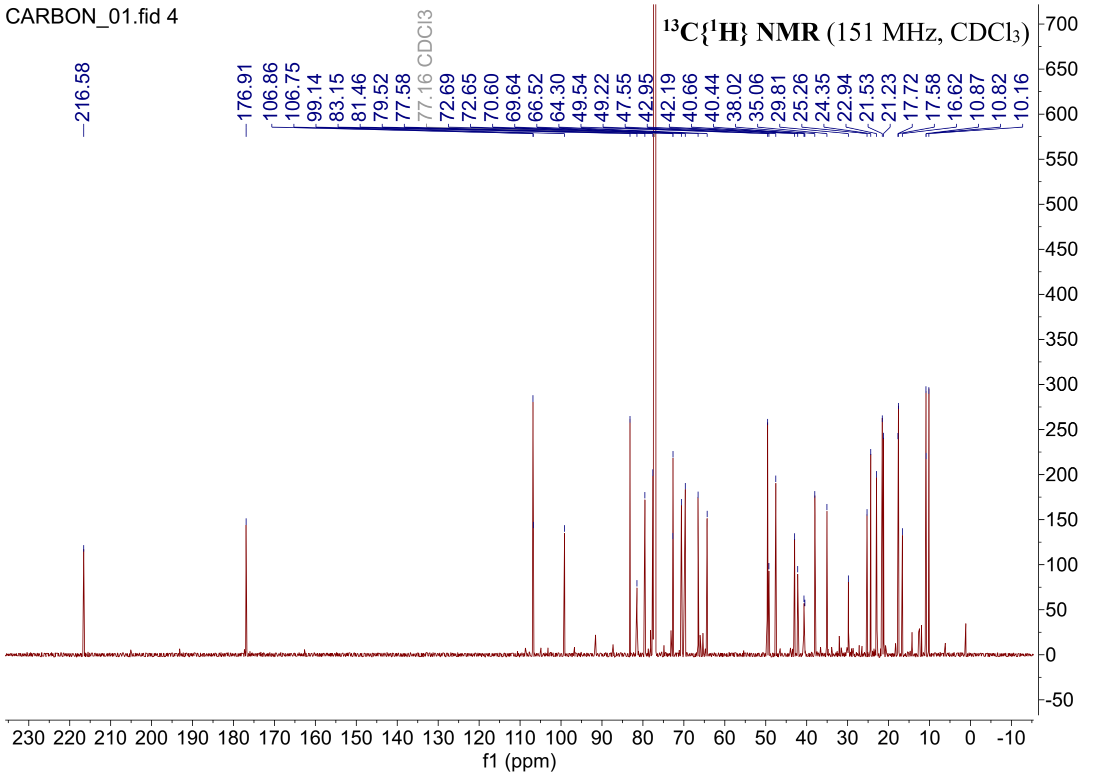
CARBON_01.fid 4
13C{1H} NMR (151 MHz, CDCl3)
216.58
176.91 106.86 106.75 99.14 83.15 81.46 79.52 77.58 77.16 CDCl3 72.69 72.65 70.60 69.64 66.52 64.30 49.54 49.22 47.55 42.95 42.19 40.66 40.44 38.02 35.06 29.81 25.26 24.35 22.94 21.53 21.23 17.72 17.58 16.62 10.87 10.82 10.16
f1 (ppm)

^{1}H NMR (500 MHz, $CDCl_3$)

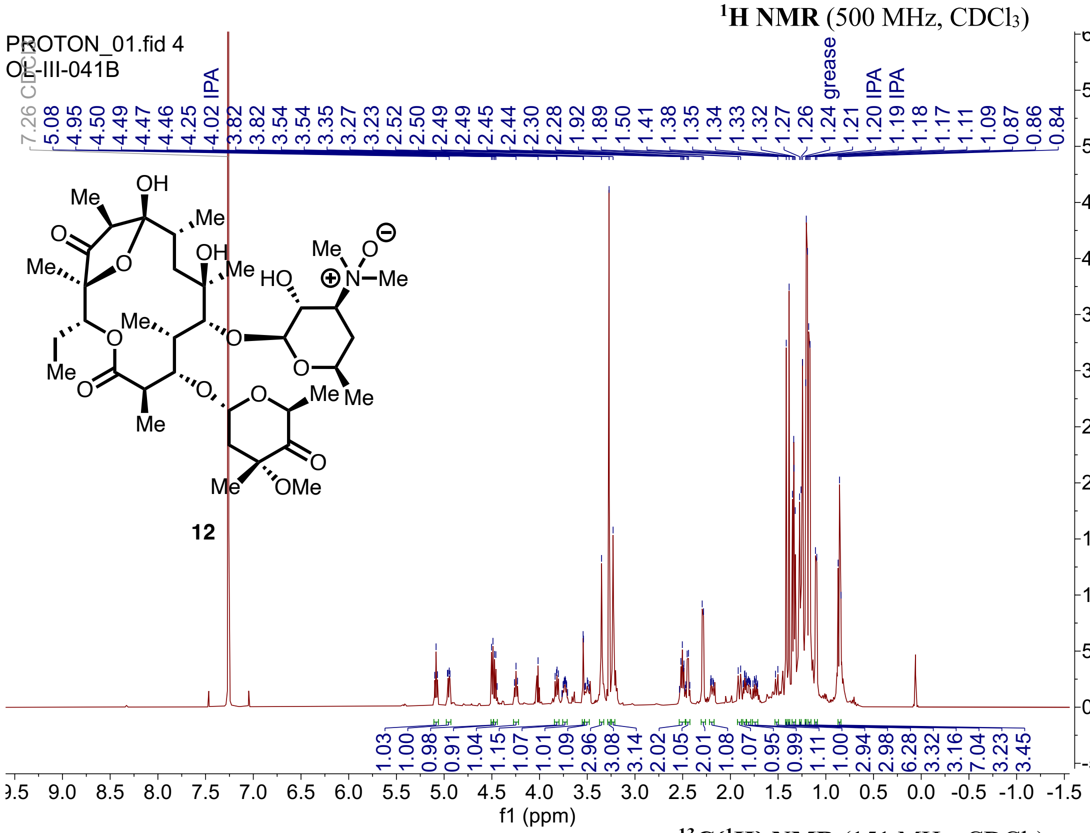

^{13}C{^{1}H} NMR (151 MHz, $CDCl_3$)

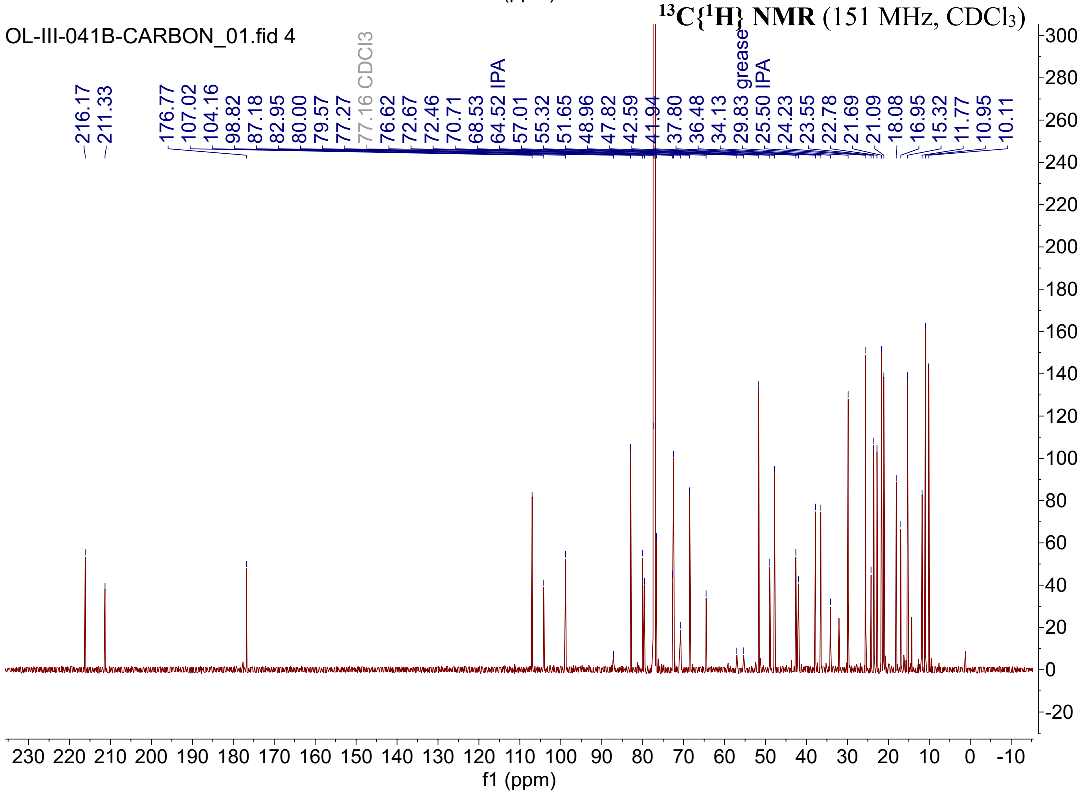

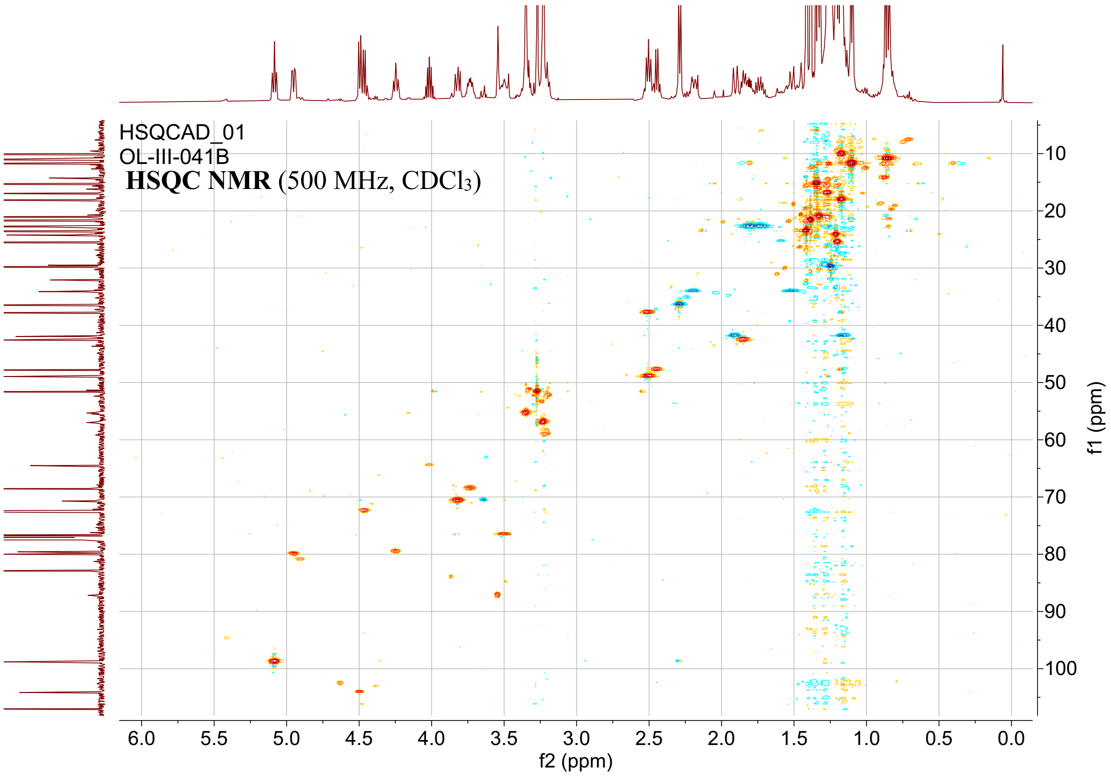
HSQCAD_01
OL-III-041B
HSQC NMR (500 MHz, CDCl3)
f2 (ppm)
f1 (ppm)

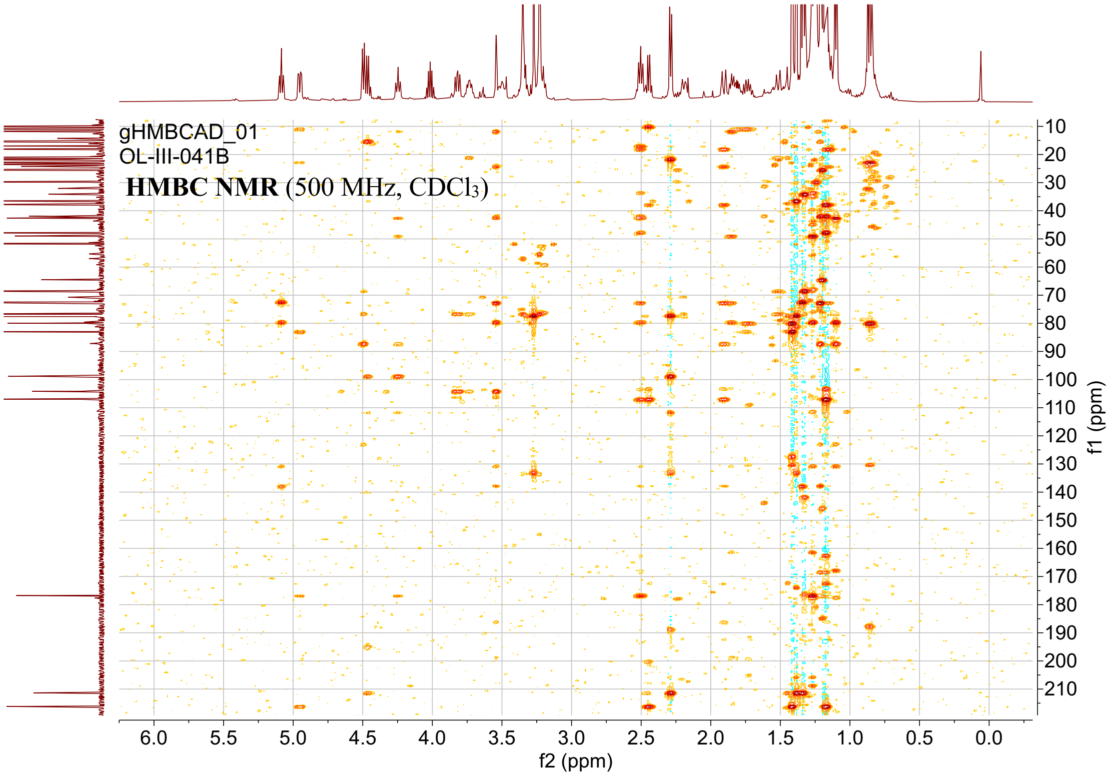
gHMBCAD_01
OL-III-041B
HMBC NMR (500 MHz, CDCl3)
f2 (ppm)
f1 (ppm)

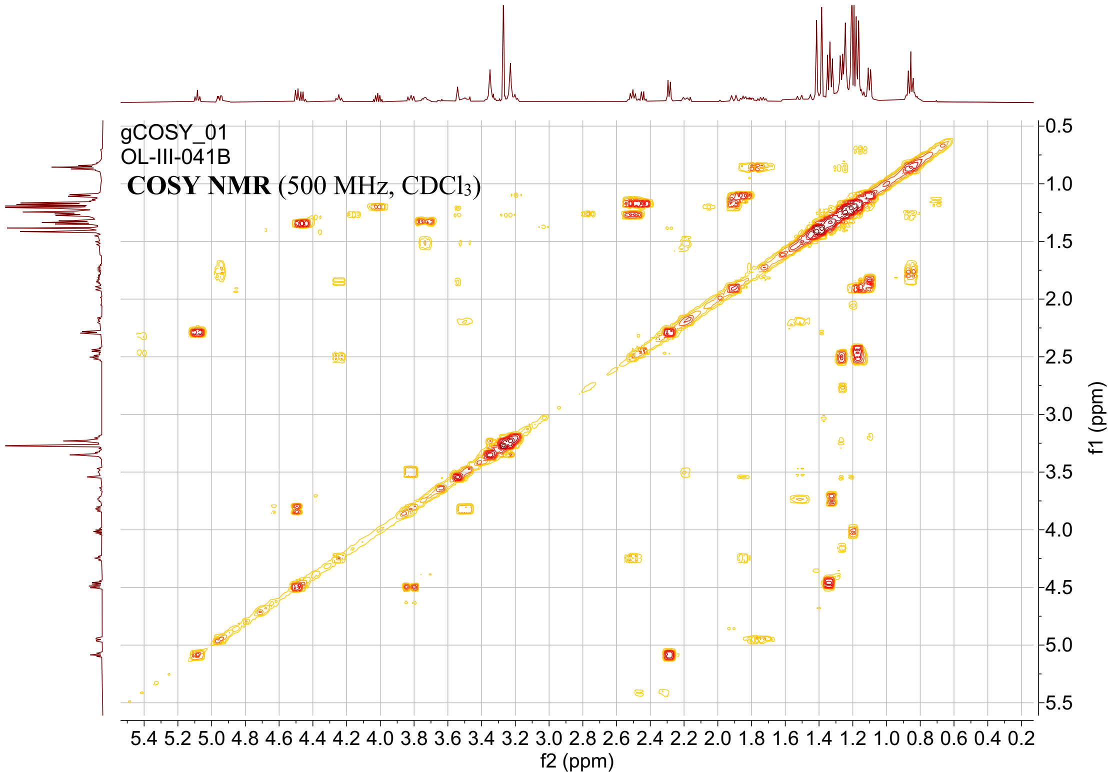
gCOSY_01
OL-III-041B
COSY NMR (500 MHz, $CDCl_3$)
f1 (ppm)
f2 (ppm)
0.5
1.0
1.5
2.0
2.5
3.0
3.5
4.0
4.5
5.0
5.5
5.4 5.2 5.0 4.8 4.6 4.4 4.2 4.0 3.8 3.6 3.4 3.2 3.0 2.8 2.6 2.4 2.2 2.0 1.8 1.6 1.4 1.2 1.0 0.8 0.6 0.4 0.2

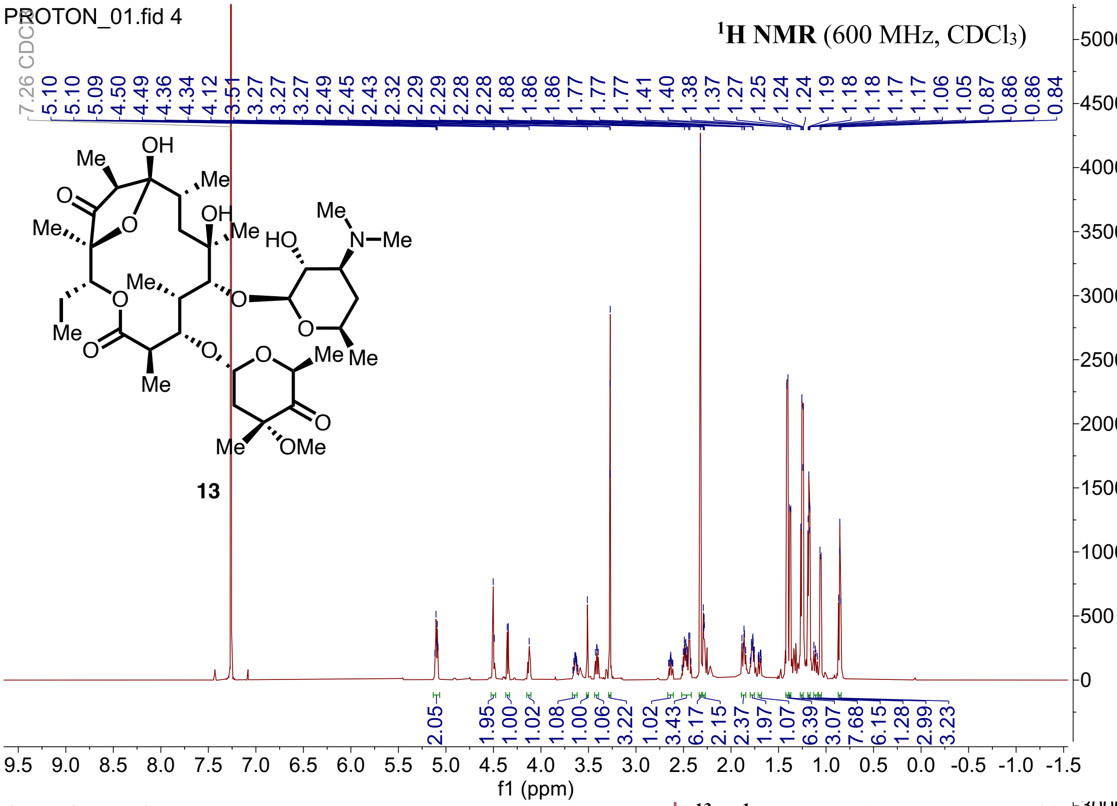
PROTON_01.fid 4
¹H NMR (600 MHz, CDCl3)
13
f1 (ppm)

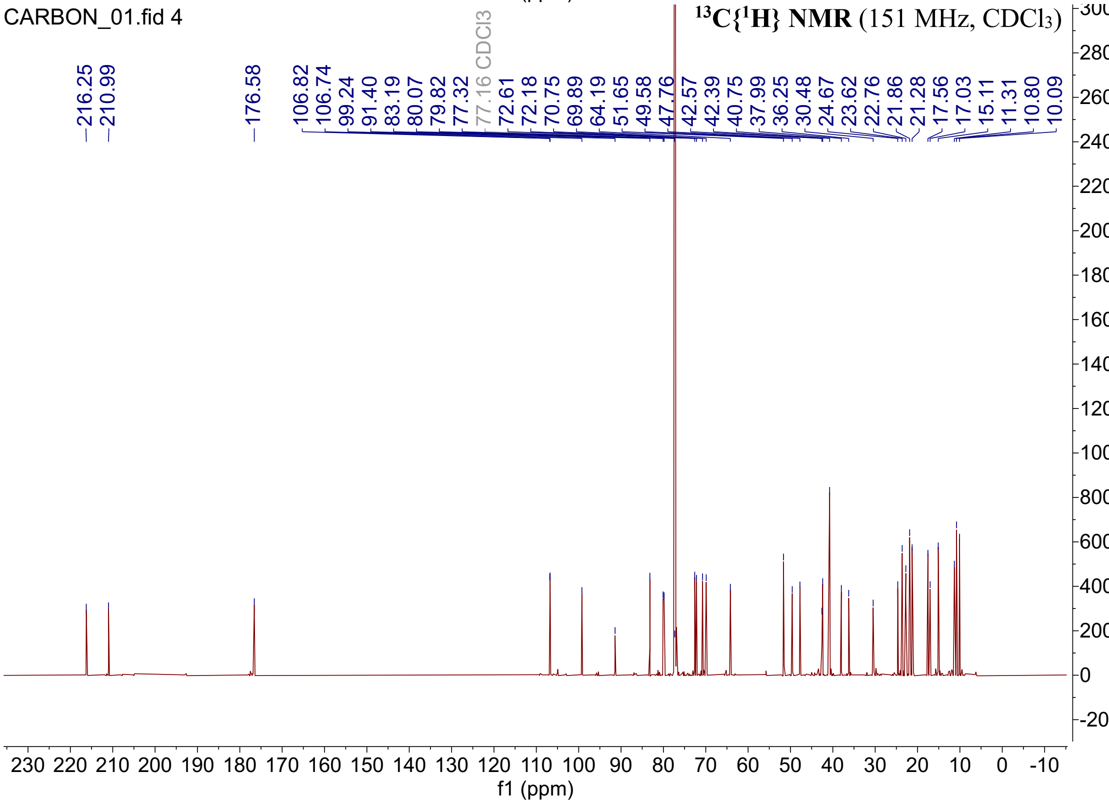
CARBON_01.fid 4
¹³C{¹H} NMR (151 MHz, CDCl3)
216.25
210.99
176.58
106.82
106.74
99.24
91.40
83.19
80.07
79.82
77.32
77.16 CDCl3
72.61
72.18
70.75
69.89
64.19
51.65
49.58
47.76
42.57
42.39
40.75
37.99
36.25
30.48
24.67
23.62
22.76
21.86
21.28
17.56
17.03
15.11
11.31
10.80
10.09
f1 (ppm)

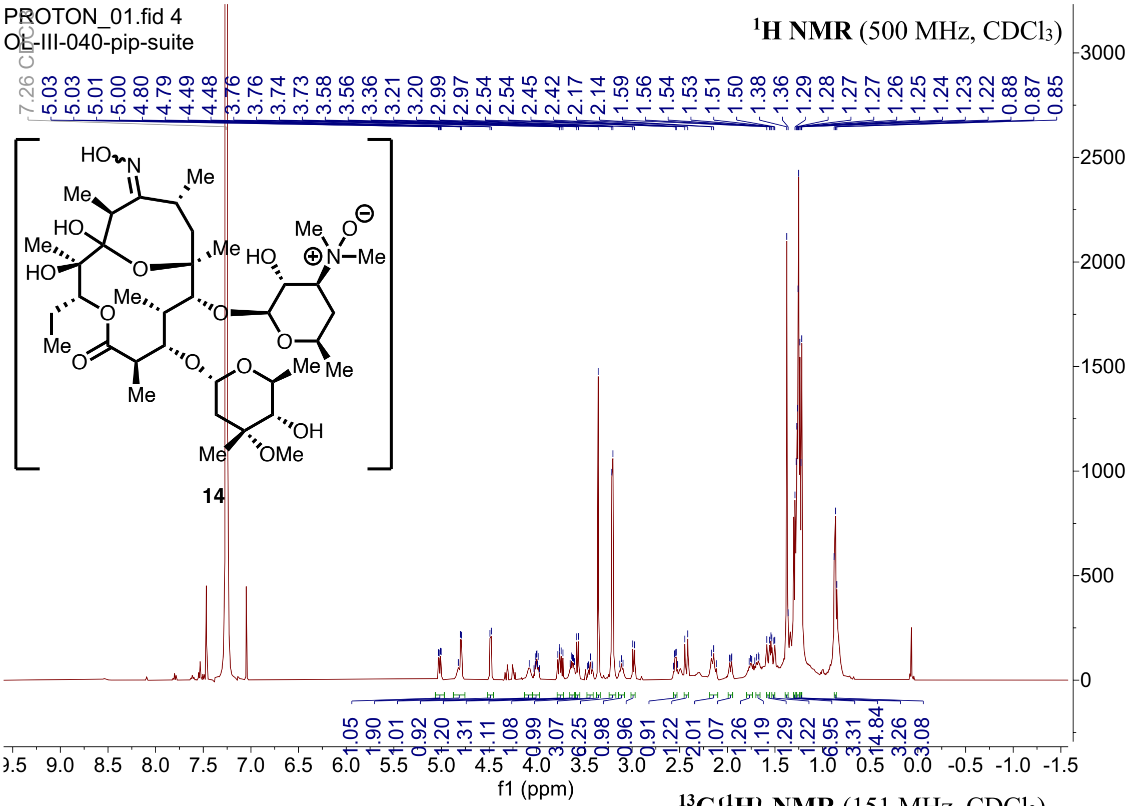
PROTON_01.fid 4
OL-III-040-pip-suite
1H NMR (500 MHz, CDCl3)
14
f1 (ppm)

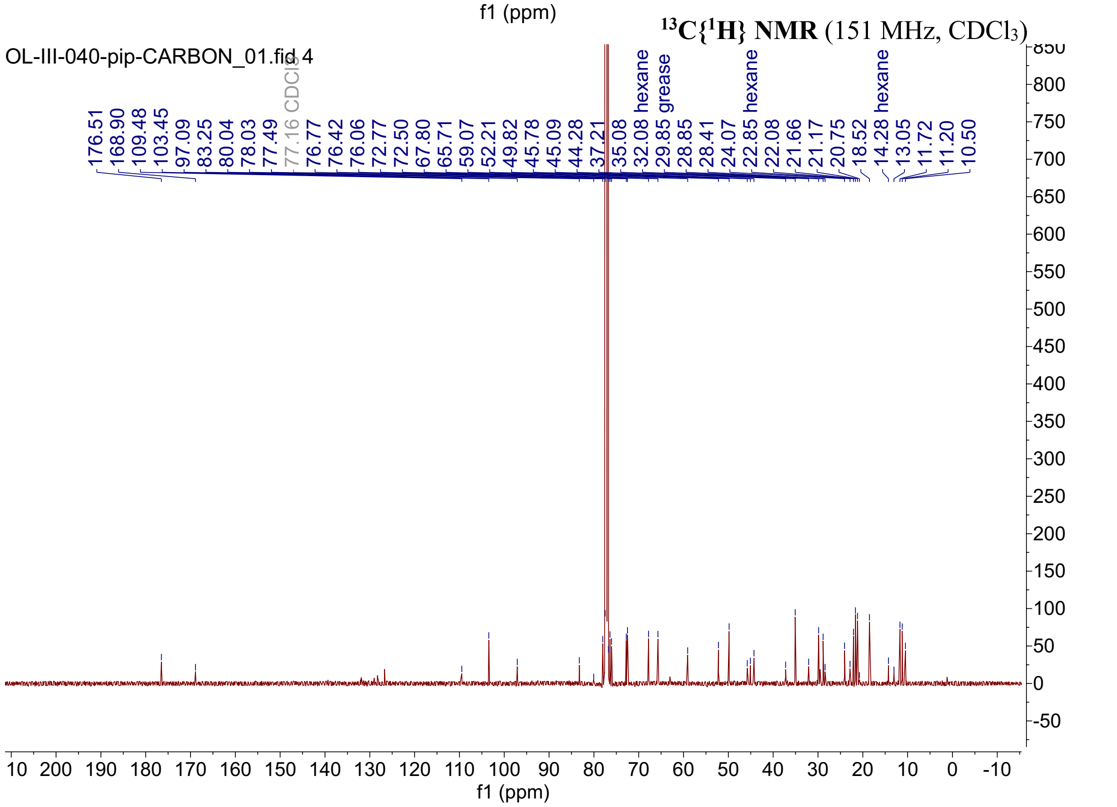
13C{1H} NMR (151 MHz, CDCl3)
OL-III-040-pip-CARBON_01.fid 4
f1 (ppm)

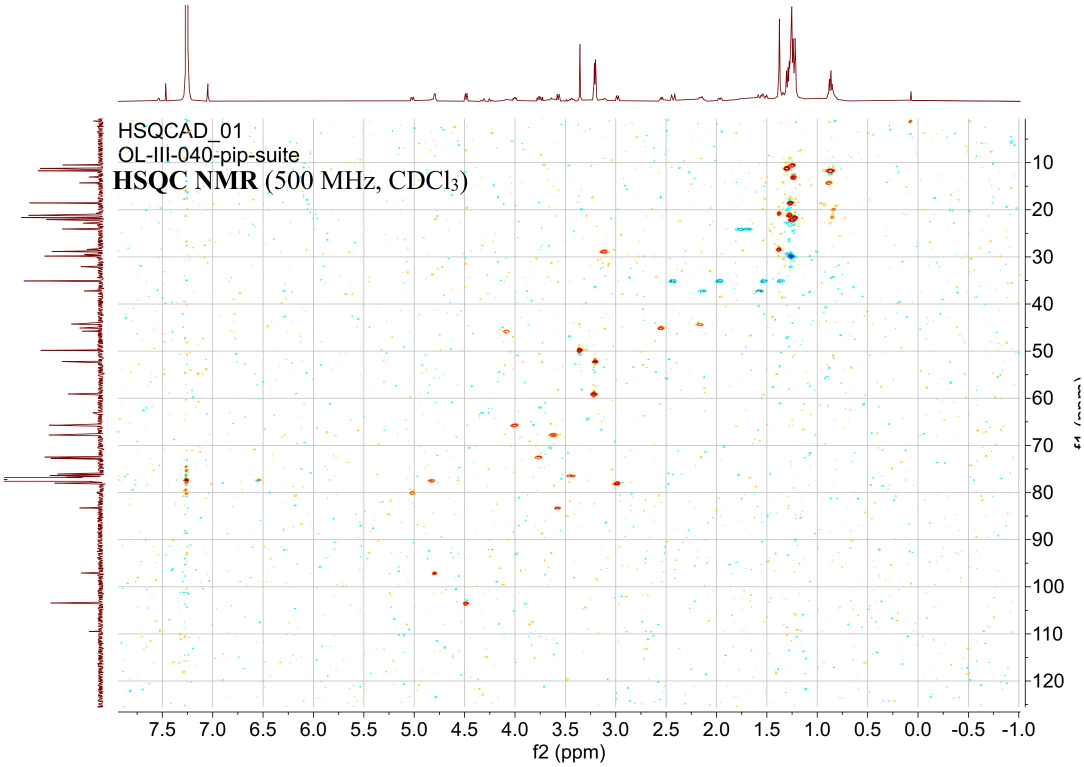
HSQCAD_01
OL-III-040-pip-suite
HSQC NMR (500 MHz, CDCl3)
f2 (ppm)

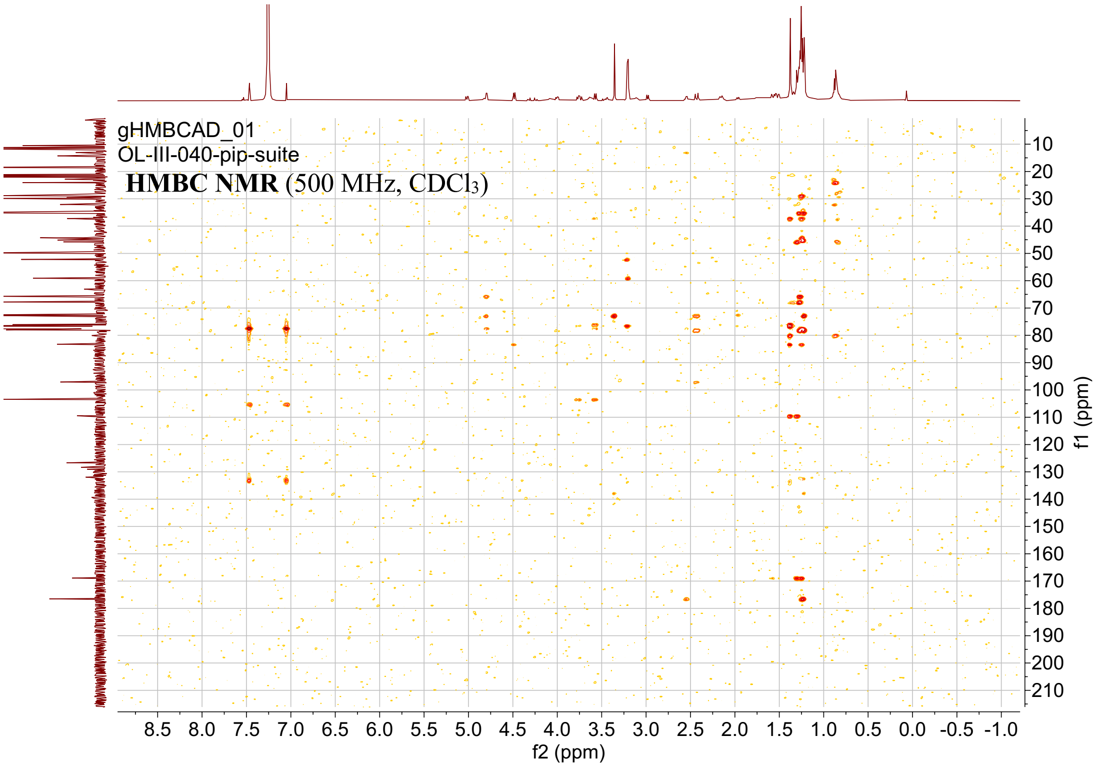
gHMBCAD_01
OL-III-040-pip-suite
HMBC NMR (500 MHz, CDCl3)
f2 (ppm)
f1 (ppm)

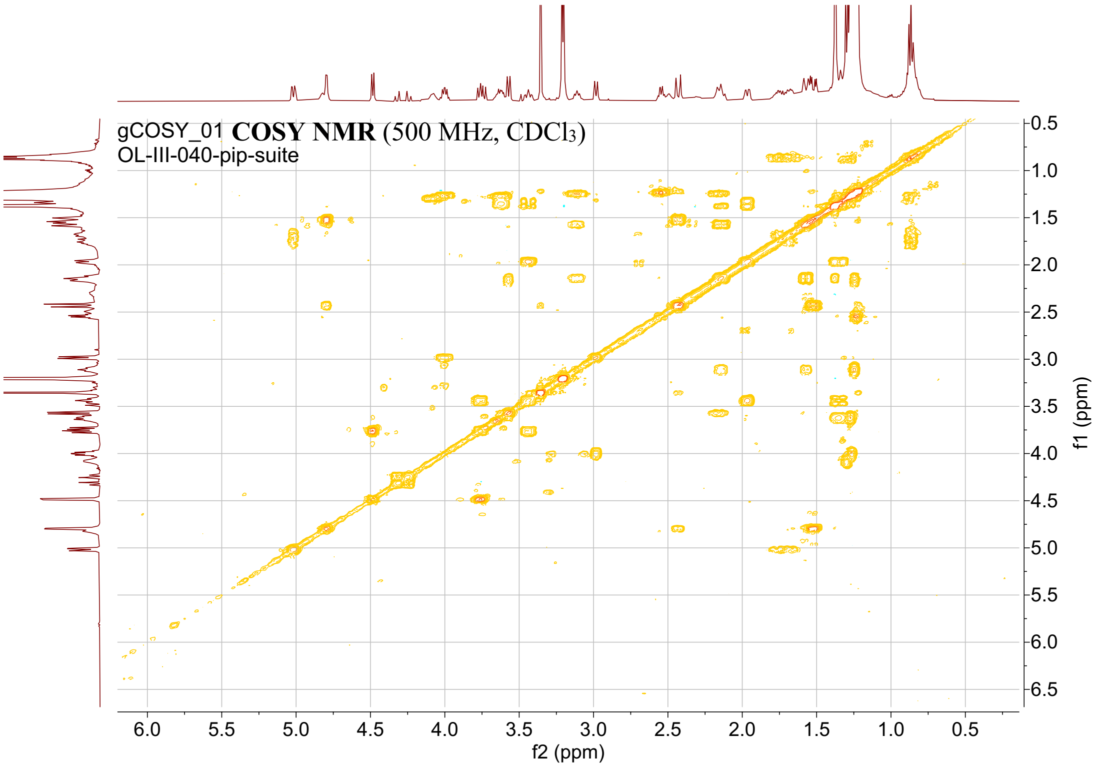
gCOSY_01 COSY NMR (500 MHz, CDCl3)
OL-III-040-pip-suite
f2 (ppm)
f1 (ppm)
6.0
5.5
5.0
4.5
4.0
3.5
3.0
2.5
2.0
1.5
1.0
0.5

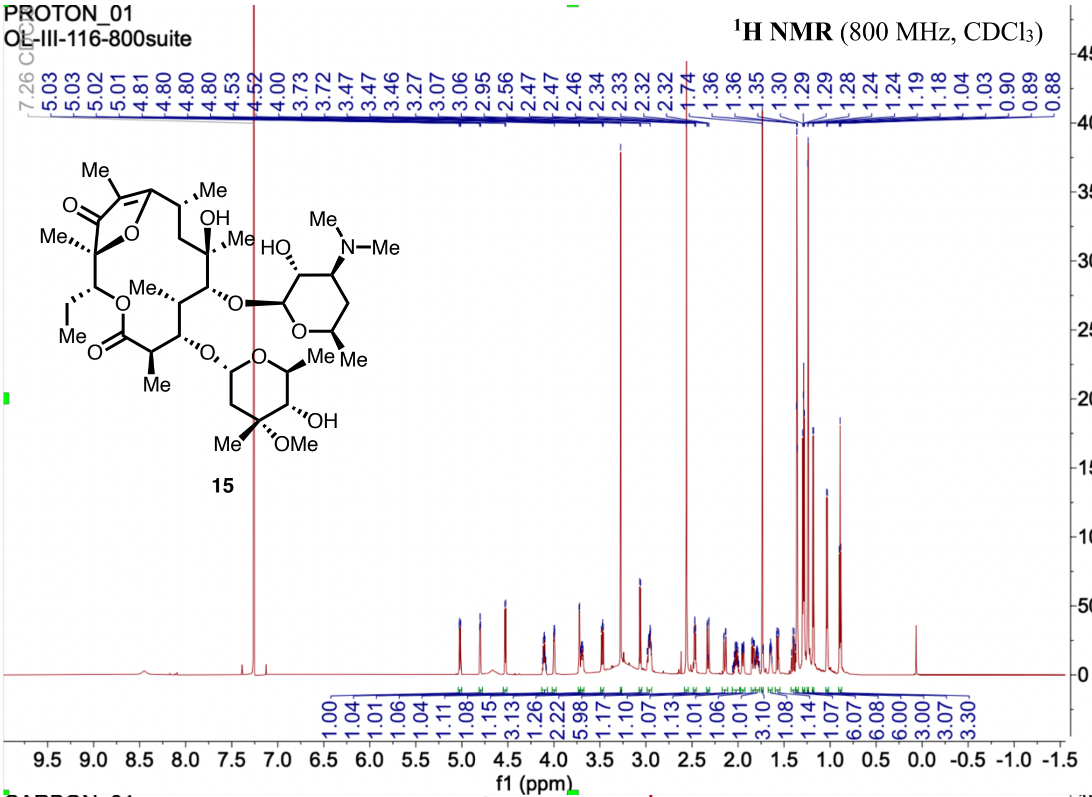
PROTON_01
OL-III-116-800suite
^{1}H NMR (800 MHz, CDCl$_3$)
7.26 CDCl3
5.03 5.03 5.02 5.01 4.81 4.80 4.80 4.80 4.53 4.52 4.00 3.73 3.72 3.47 3.47 3.46 3.27 3.07 3.06 2.95 2.56 2.47 2.47 2.46 2.34 2.33 2.32 2.32 1.74 1.36 1.36 1.35 1.30 1.29 1.29 1.28 1.24 1.24 1.19 1.18 1.04 1.03 0.90 0.89 0.88
1.00 1.04 1.01 1.06 1.04 1.11 1.08 1.15 3.13 1.26 2.22 5.98 1.17 1.10 1.07 1.13 1.01 1.06 1.01 3.10 1.08 1.14 1.07 6.07 6.08 6.00 3.00 3.07 3.30
Me
Me
O
OH
Me
O
Me
HO
N
Me
Me
O
Me
O
O
O
Me
O
O
O
Me
Me
Me
OH
Me
OMe
15
f1 (ppm)

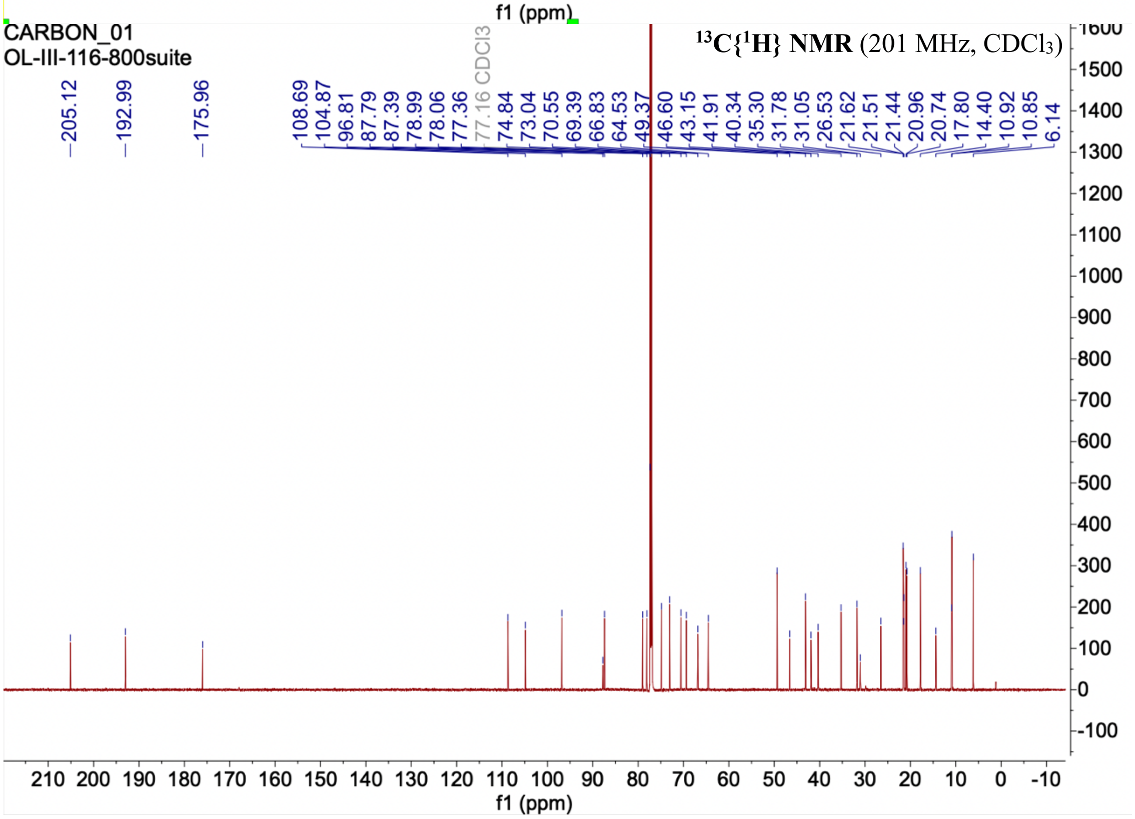
CARBON_01
OL-III-116-800suite
^{13}C{^{1}H} NMR (201 MHz, CDCl$_3$)
205.12
192.99
175.96
108.69 104.87 96.81 87.79 87.39 78.99 78.06 77.36 77.16 CDCl3 74.84 73.04 70.55 69.39 66.83 64.53 49.37 46.60 43.15 41.91 40.34 35.30 31.78 31.05 26.53 21.62 21.51 21.44 20.96 20.74 17.80 14.40 10.92 10.85 6.14
f1 (ppm)

HSCQ NMR (800 MHz, $CDCl_3$)

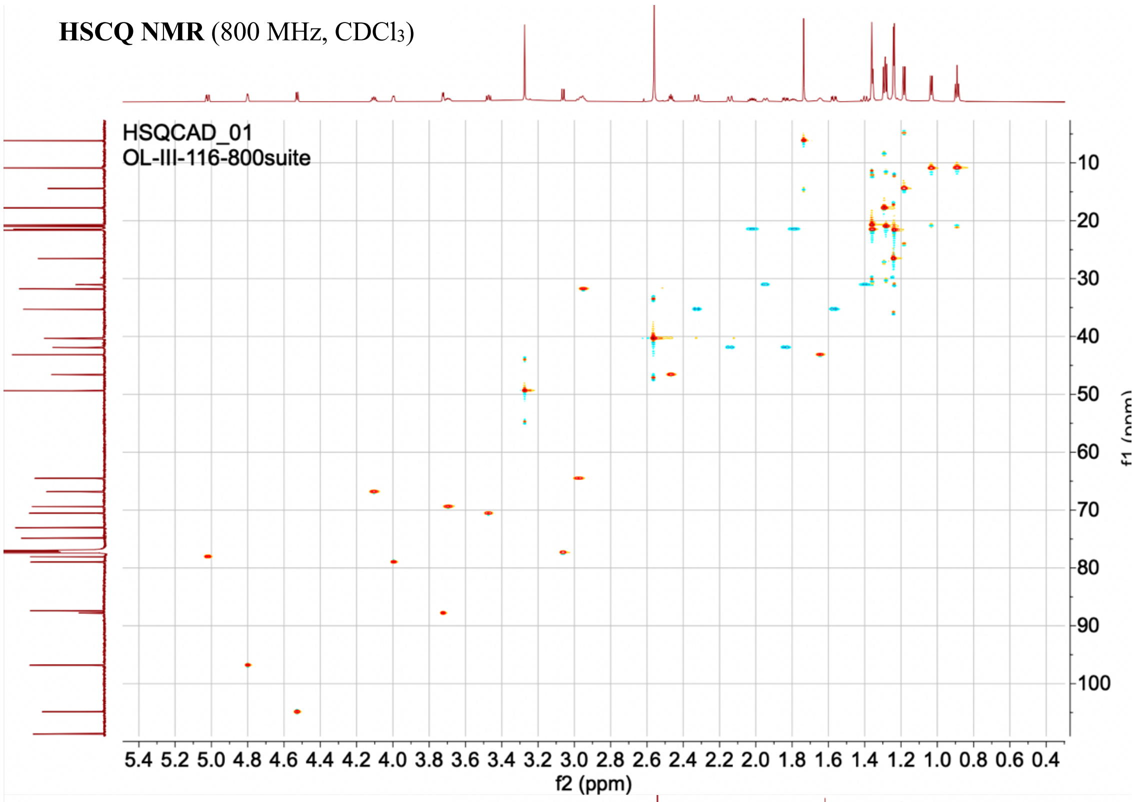

HMBC NMR (800 MHz, $CDCl_3$)

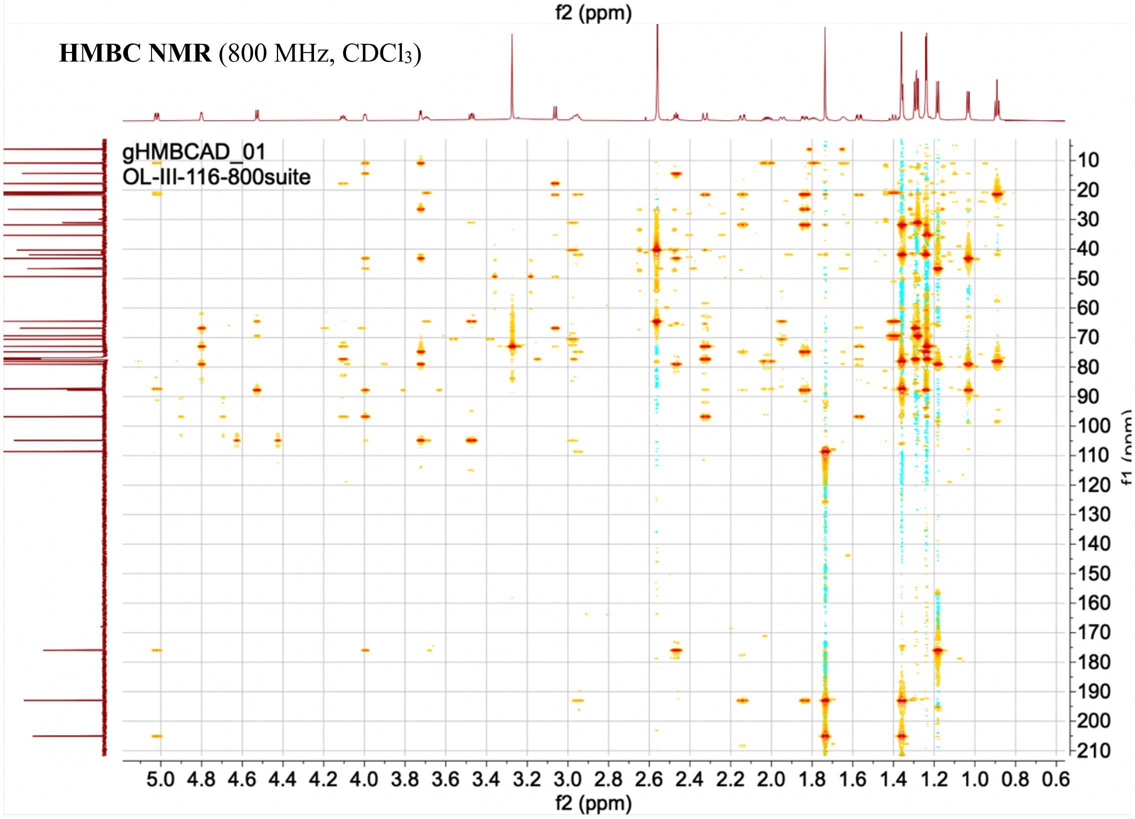

COSY NMR (800 MHz, $CDCl_3$)

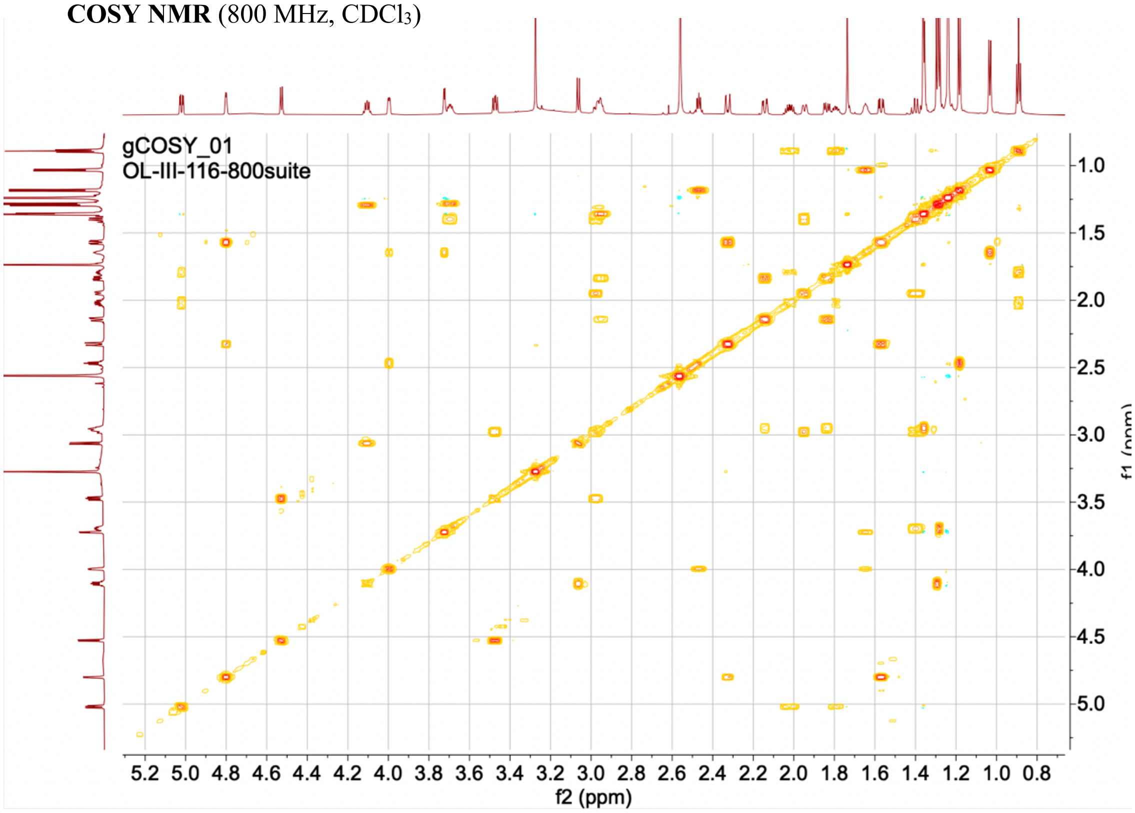

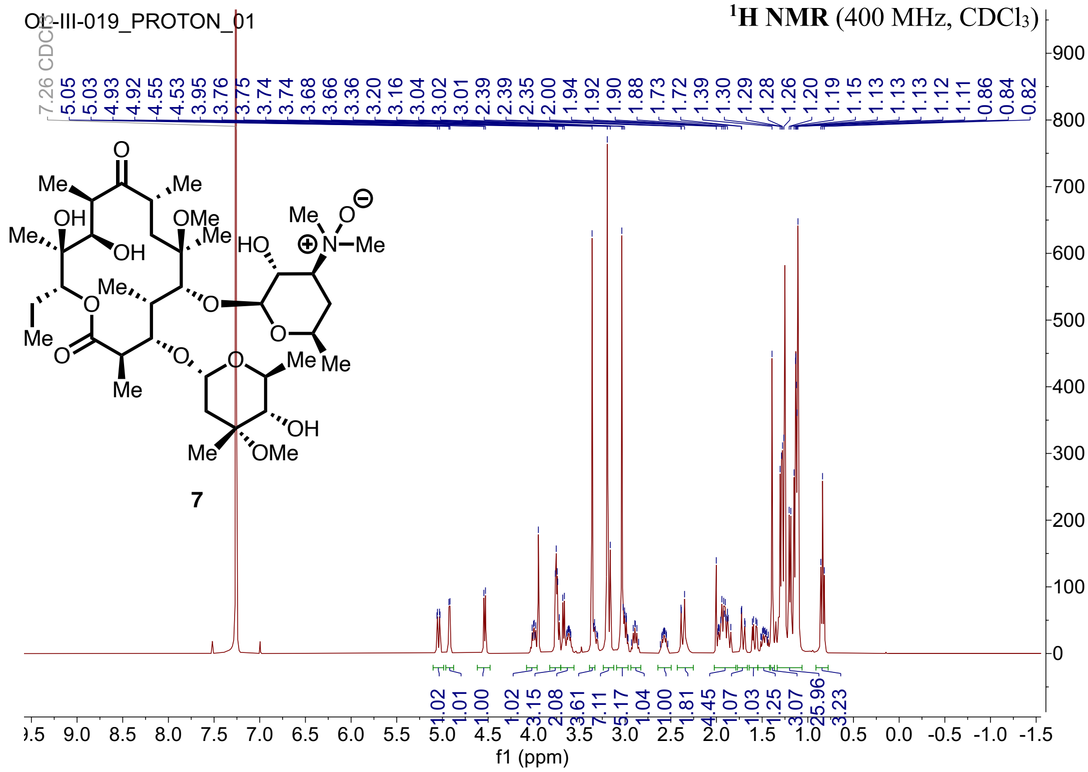
OL-III-019_PROTON_01
^{1}H NMR (400 MHz, CDCl$_3$)
7.26 CDCl3
5.05 5.03 4.93 4.92 4.55 4.53 3.95 3.76 3.75 3.74 3.74 3.68 3.66 3.36 3.20 3.16 3.04 3.02 3.01 2.39 2.39 2.35 2.00 1.94 1.92 1.90 1.88 1.73 1.72 1.39 1.30 1.29 1.28 1.26 1.20 1.19 1.15 1.13 1.13 1.13 1.12 1.11 0.86 0.84 0.82
1.02 1.01 1.00 1.02 3.15 2.08 3.61 7.11 5.17 1.04 1.00 1.81 4.45 1.07 1.03 1.25 3.07 25.96 3.23
7
f1 (ppm)

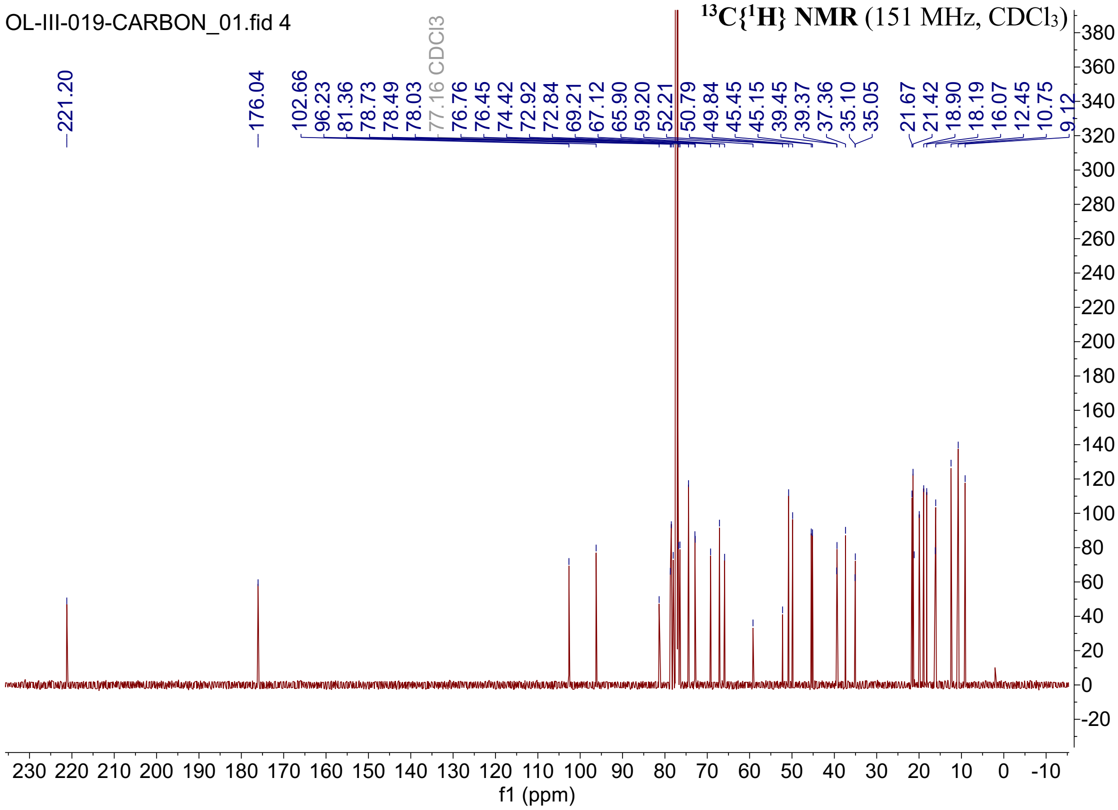
OL-III-019-CARBON_01.fid 4
^{13}C{^{1}H} NMR (151 MHz, CDCl$_3$)
221.20
176.04
102.66 96.23 81.36 78.73 78.49 78.03 77.16 CDCl3 76.76 76.45 74.42 72.92 72.84 69.21 67.12 65.90 59.20 52.21 50.79 49.84 45.45 45.15 39.45 39.37 37.36 35.10 35.05 21.67 21.42 18.90 18.19 16.07 12.45 10.75 9.12
f1 (ppm)

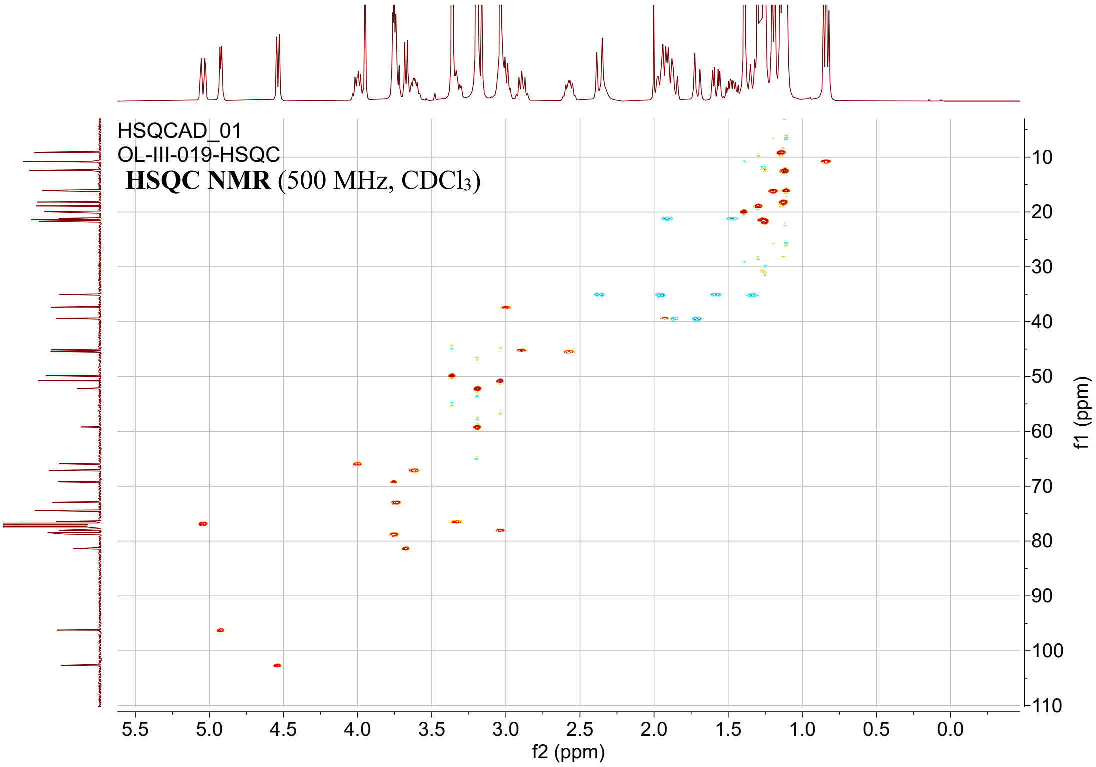
HSQCAD_01
OL-III-019-HSQC
HSQC NMR (500 MHz, CDCl3)
f1 (ppm)
f2 (ppm)

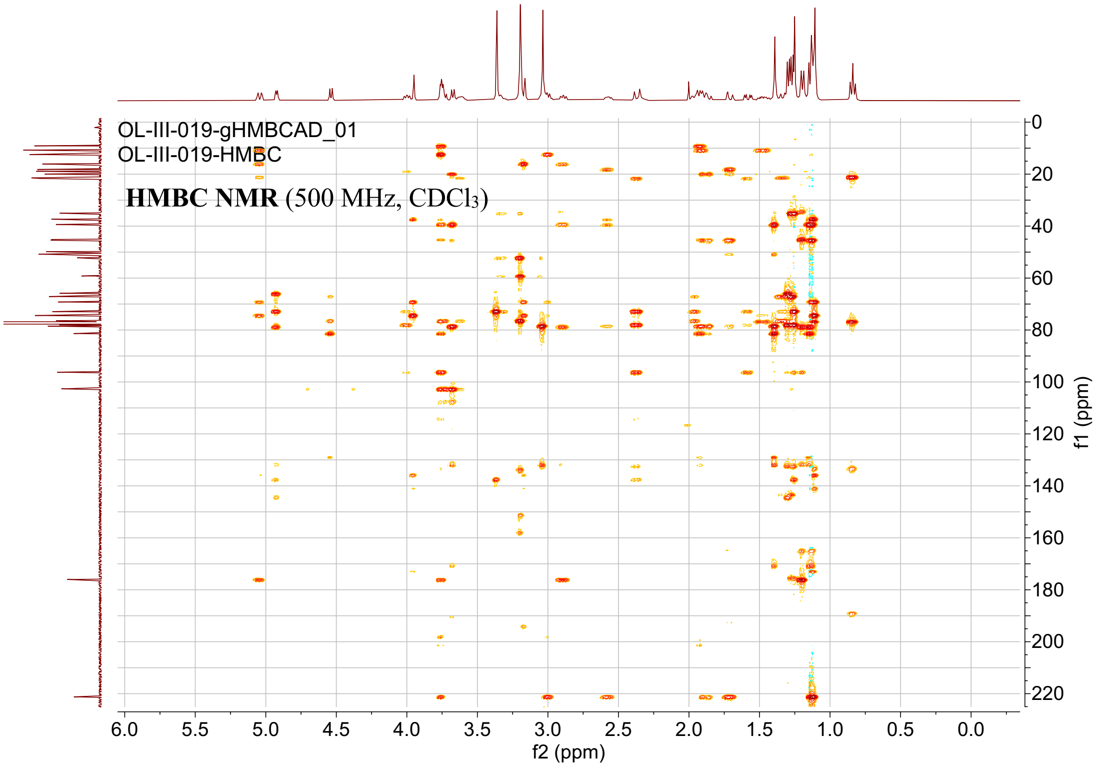
OL-III-019-gHMBCAD_01
OL-III-019-HMBC
HMBC NMR (500 MHz, CDCl3)
f1 (ppm)
f2 (ppm)

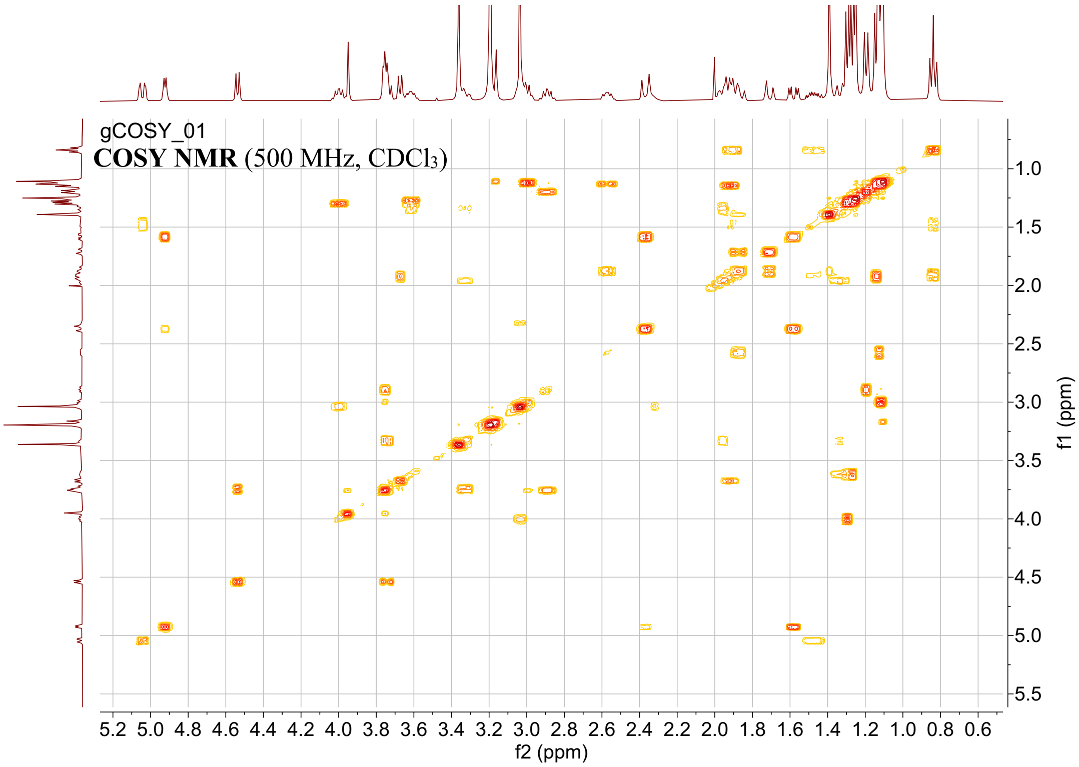
gCOSY_01
COSY NMR (500 MHz, CDCl3)
f2 (ppm)
f1 (ppm)

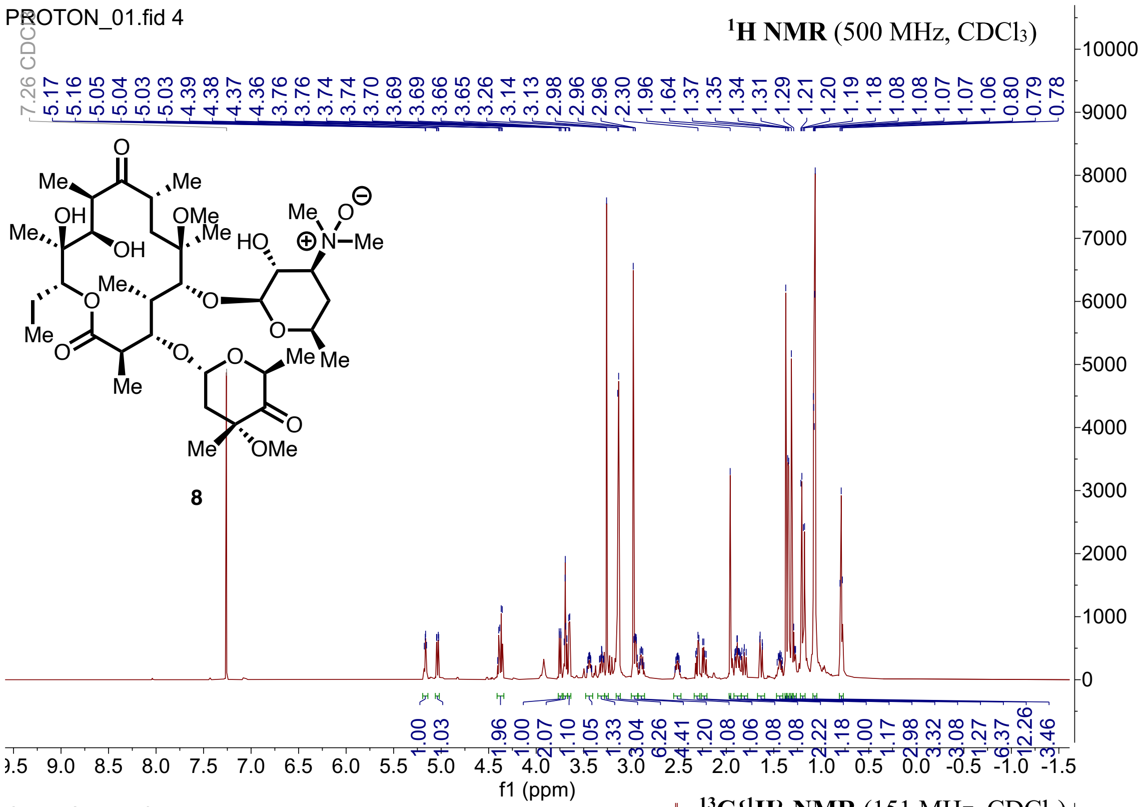
PROTON_01.fid 4
^{1}H NMR (500 MHz, CDCl$_3$)
7.26 CDCl3
5.17 5.16 5.05 5.04 5.03 5.03 4.39 4.38 4.37 4.36 3.76 3.76 3.74 3.74 3.70 3.69 3.69 3.66 3.65 3.26 3.14 3.13 2.98 2.96 2.96 2.30 1.96 1.64 1.37 1.35 1.34 1.31 1.29 1.21 1.20 1.19 1.18 1.08 1.08 1.07 1.07 1.06 0.80 0.79 0.78
1.00 1.03 1.96 1.00 2.07 1.10 1.05 1.33 3.04 6.26 4.41 1.20 1.08 1.06 1.08 1.08 2.22 1.18 1.00 1.17 2.98 3.32 3.08 1.27 6.37 12.26 3.46
8
f1 (ppm)

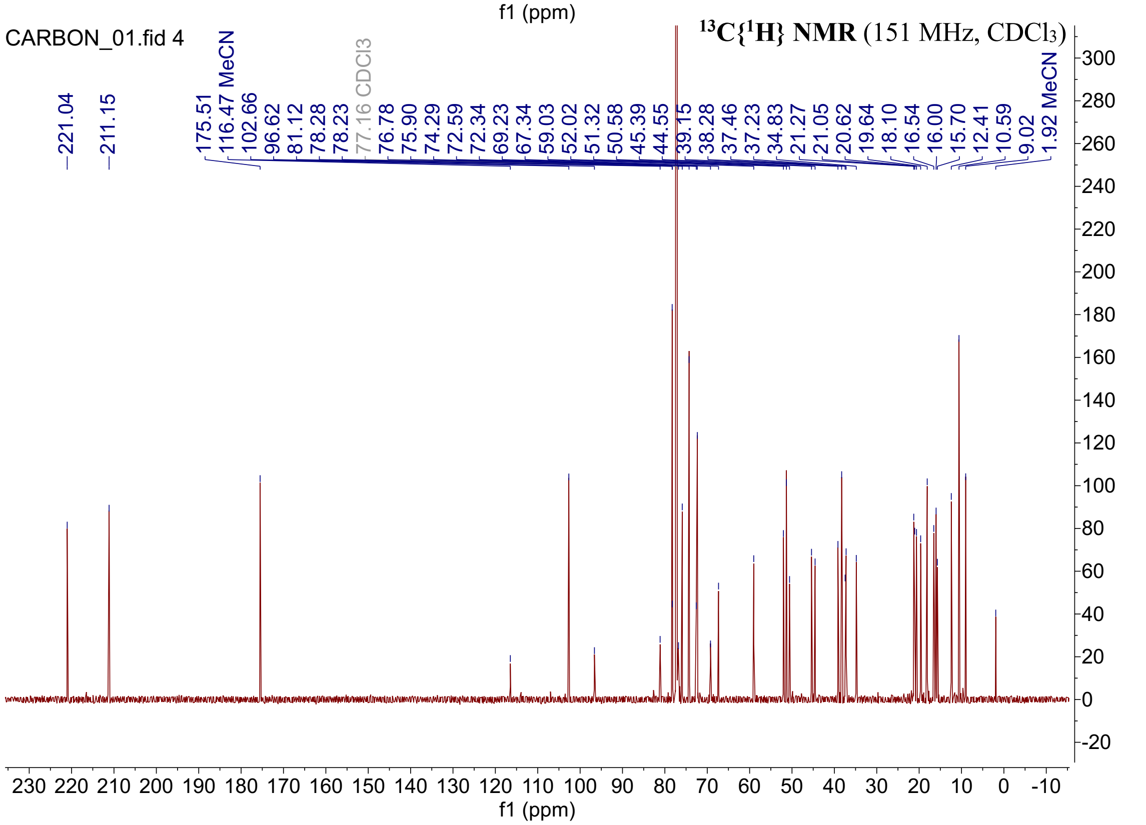
CARBON_01.fid 4
^{13}C{^{1}H} NMR (151 MHz, CDCl$_3$)
221.04 211.15
175.51 116.47 MeCN 102.66 96.62 81.12 78.28 78.23 77.16 CDCl3 76.78 75.90 74.29 72.59 72.34 69.23 67.34 59.03 52.02 51.32 50.58 45.39 44.55 39.15 38.28 37.46 37.23 34.83 21.27 21.05 20.62 19.64 18.10 16.54 16.00 15.70 12.41 10.59 9.02 1.92 MeCN
f1 (ppm)

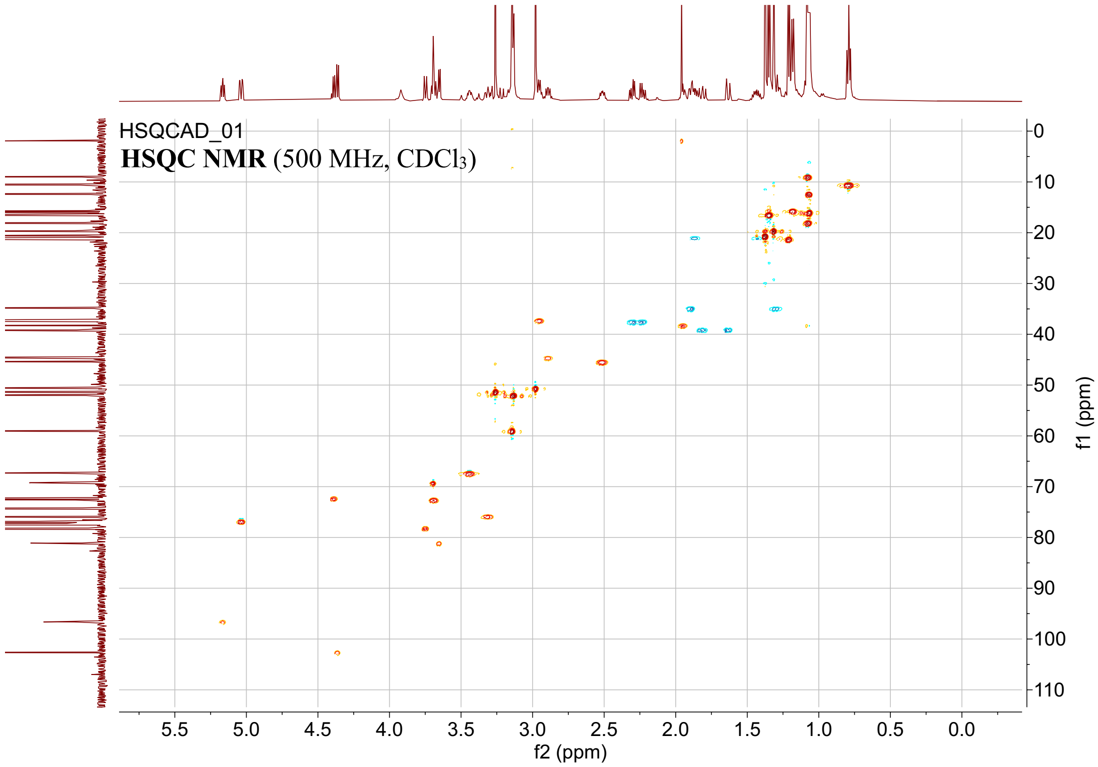
HSQCAD_01
HSQC NMR (500 MHz, CDCl3)
f2 (ppm)
f1 (ppm)

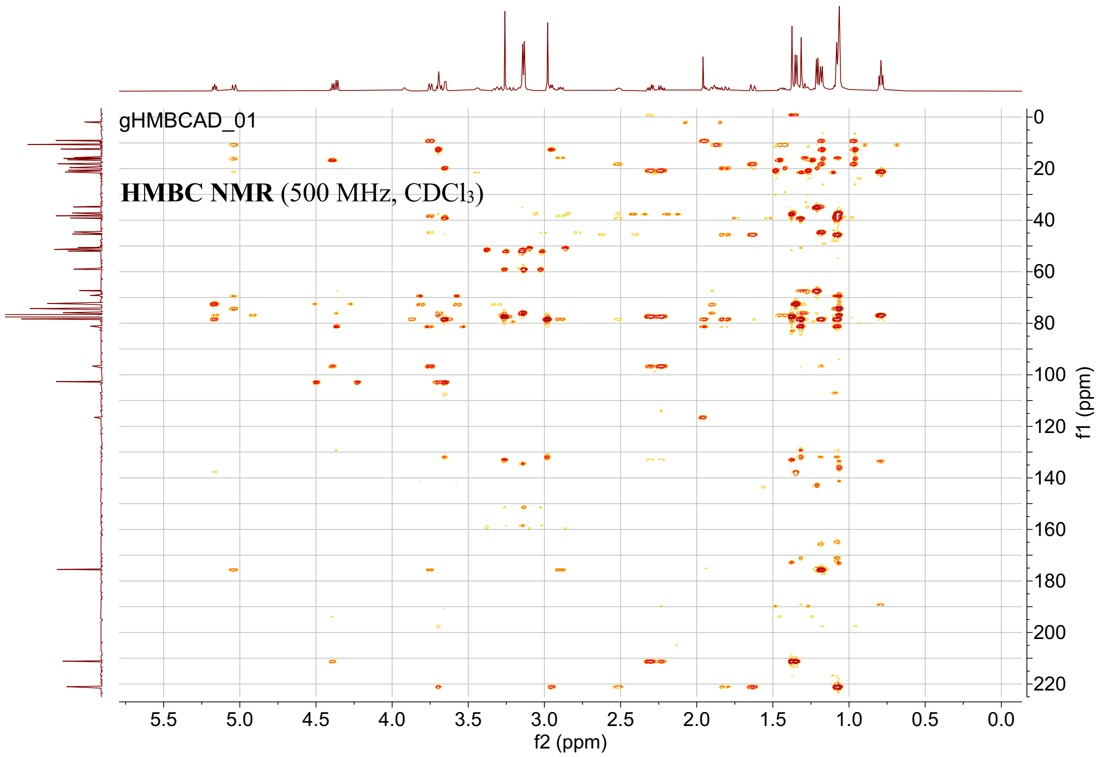
gHMBCAD_01
HMBC NMR (500 MHz, CDCl3)
f2 (ppm)
f1 (ppm)

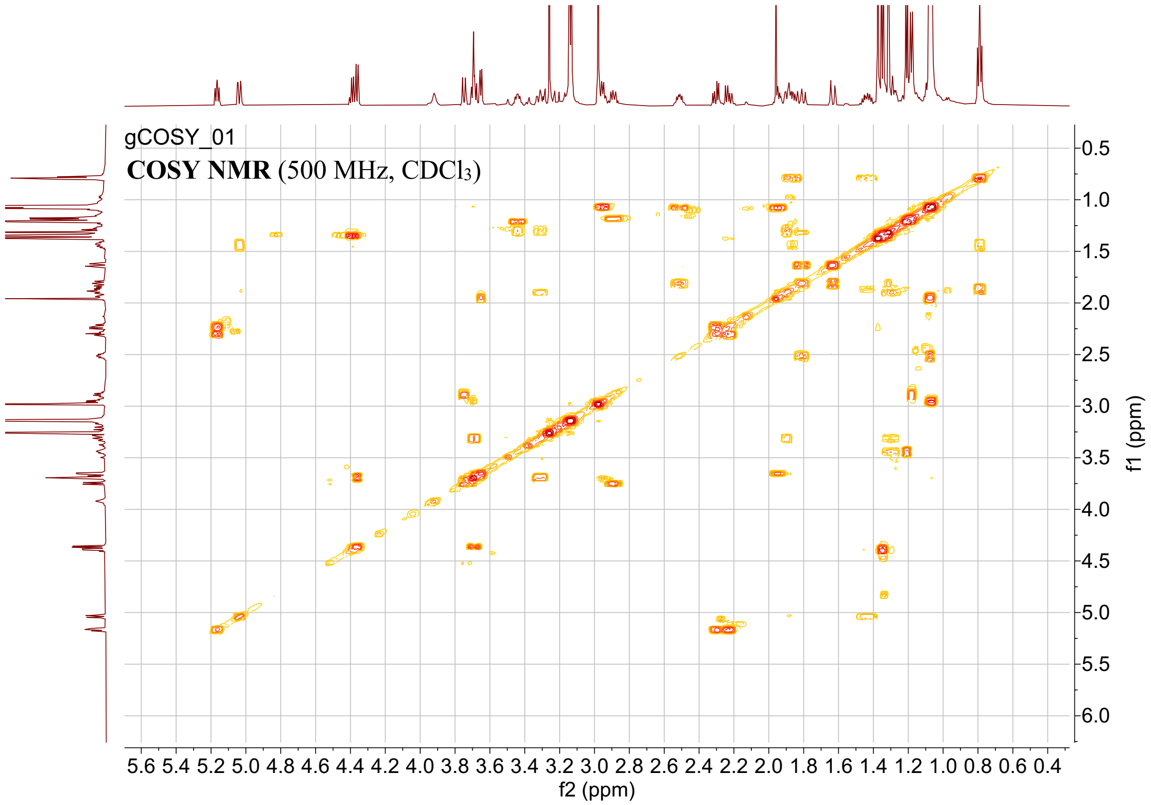
gCOSY_01
COSY NMR (500 MHz, $CDCl_3$)
f2 (ppm)
f1 (ppm)

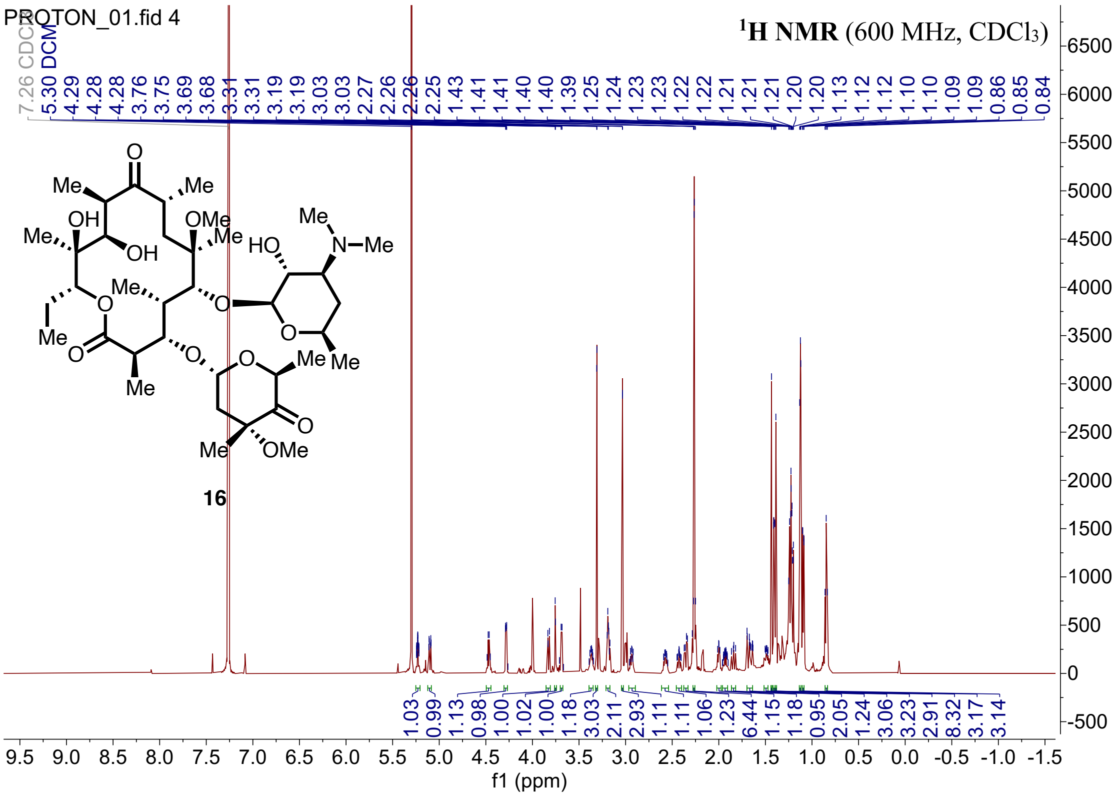
PROTON_01.fid 4
¹H NMR (600 MHz, CDCl₃)
7.26 CDCl3
5.30 DCM
4.29 4.28 4.28 3.76 3.75 3.69 3.68 3.31 3.31 3.19 3.19 3.03 3.03 2.27 2.26 2.26 2.25 1.43 1.41 1.41 1.40 1.40 1.39 1.25 1.24 1.23 1.23 1.22 1.22 1.21 1.21 1.21 1.20 1.20 1.13 1.12 1.12 1.10 1.10 1.09 1.09 0.86 0.85 0.84
1.03 0.99 1.13 0.98 1.00 1.02 1.00 1.18 3.03 2.11 2.93 1.11 1.11 1.06 1.23 6.44 1.15 1.18 0.95 2.05 1.24 3.06 3.23 2.91 8.32 3.17 3.14
16
f1 (ppm)

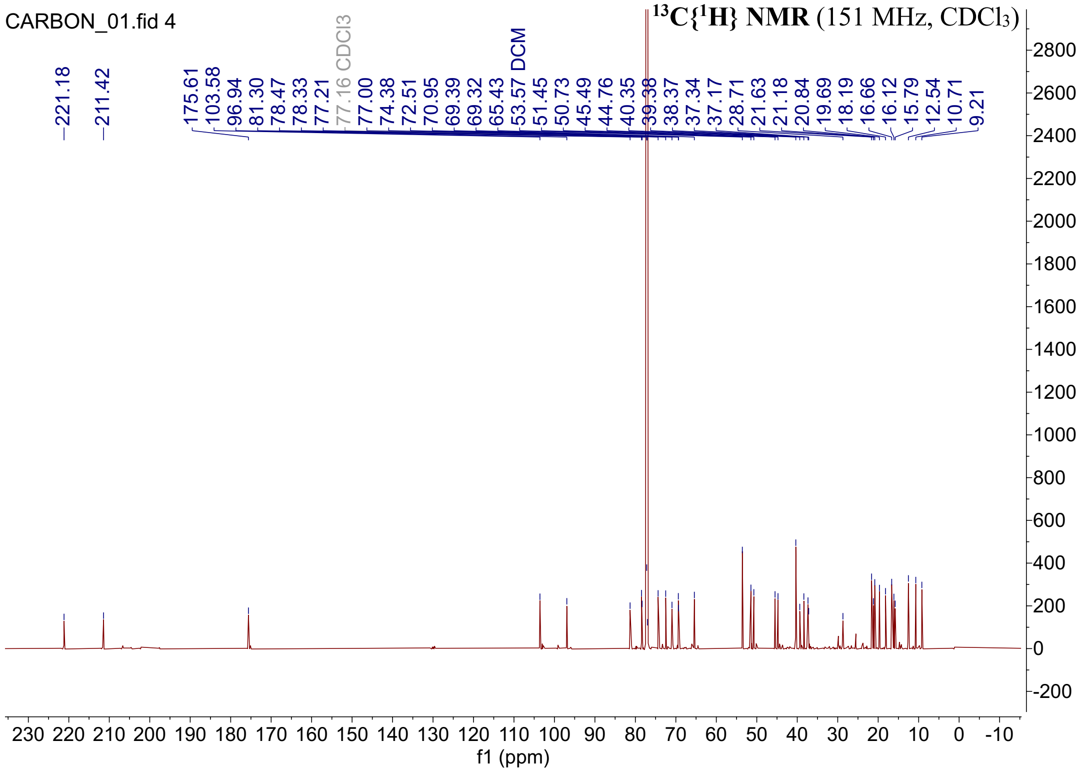
CARBON_01.fid 4
¹³C{¹H} NMR (151 MHz, CDCl₃)
221.18 211.42 175.61 103.58 96.94 81.30 78.47 78.33 77.21 77.16 CDCl3 77.00 74.38 72.51 70.95 69.39 69.32 65.43 53.57 DCM 51.45 50.73 45.49 44.76 40.35 39.38 38.37 37.34 37.17 28.71 21.63 21.18 20.84 19.69 18.19 16.66 16.12 15.79 12.54 10.71 9.21
f1 (ppm)

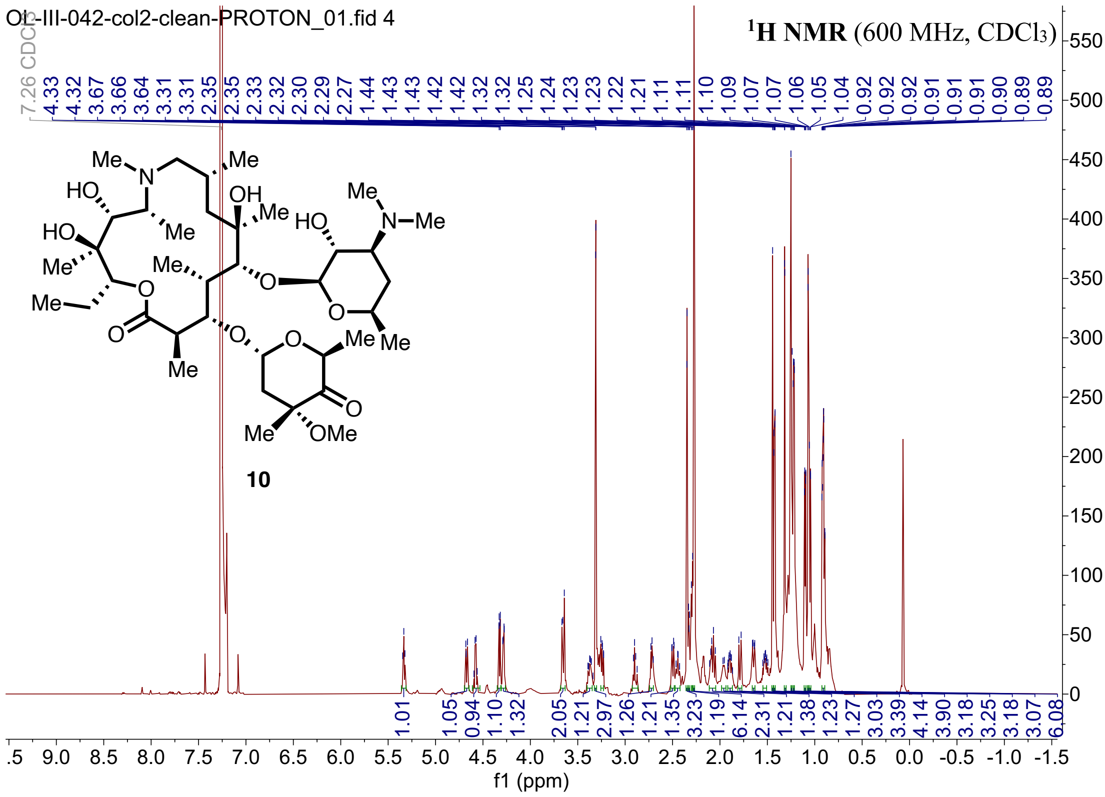
OL-III-042-col2-clean-PROTON_01.fid 4
^{1}H NMR (600 MHz, CDCl$_3$)
10
f1 (ppm)

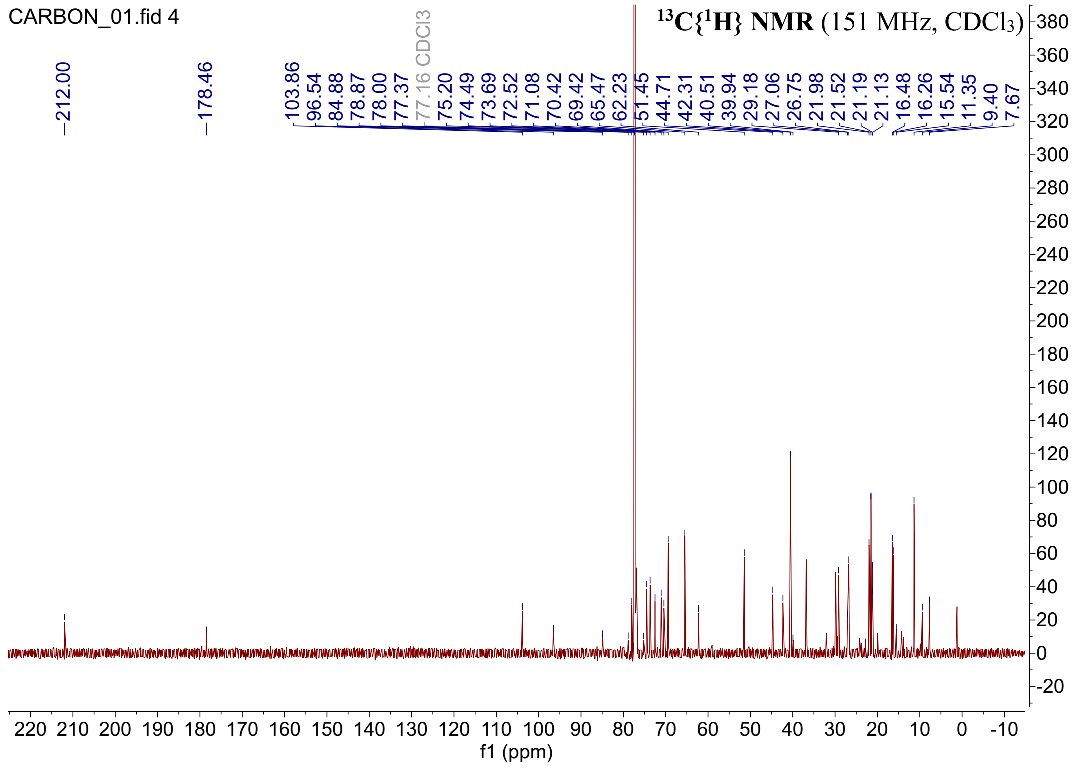
CARBON_01.fid 4
^{13}C{^{1}H} NMR (151 MHz, CDCl$_3$)
f1 (ppm)

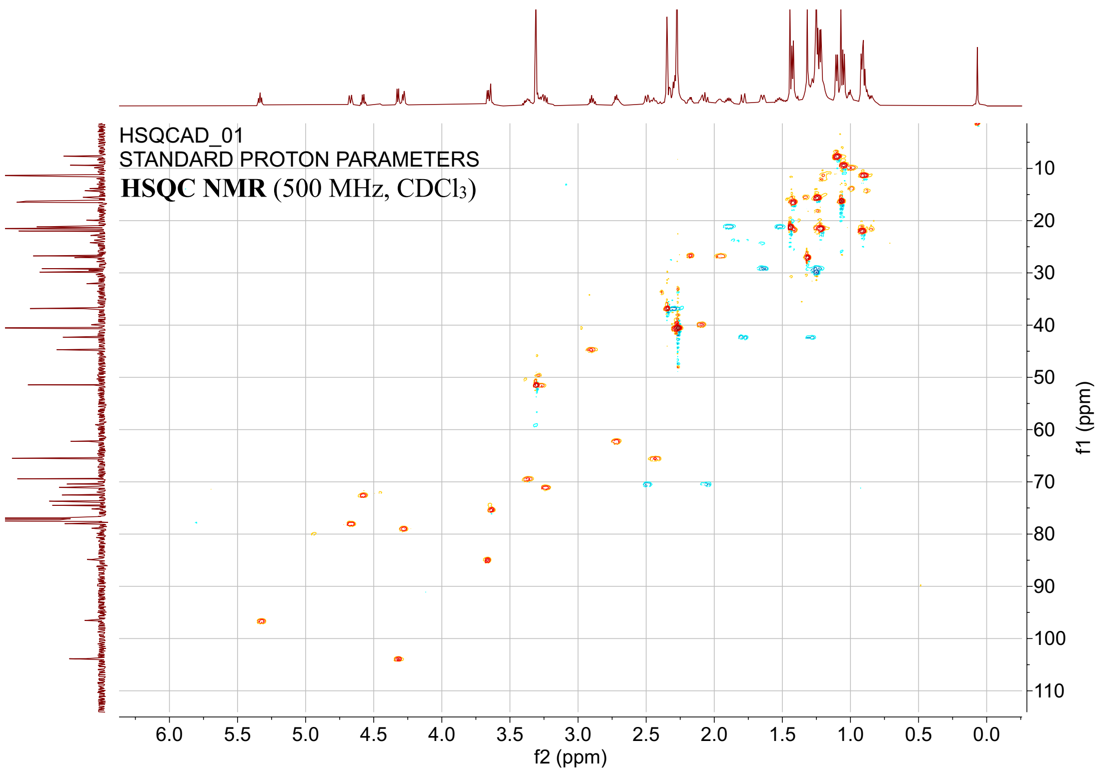
HSQCAD_01
STANDARD PROTON PARAMETERS
HSQC NMR (500 MHz, CDCl3)
f1 (ppm)
f2 (ppm)

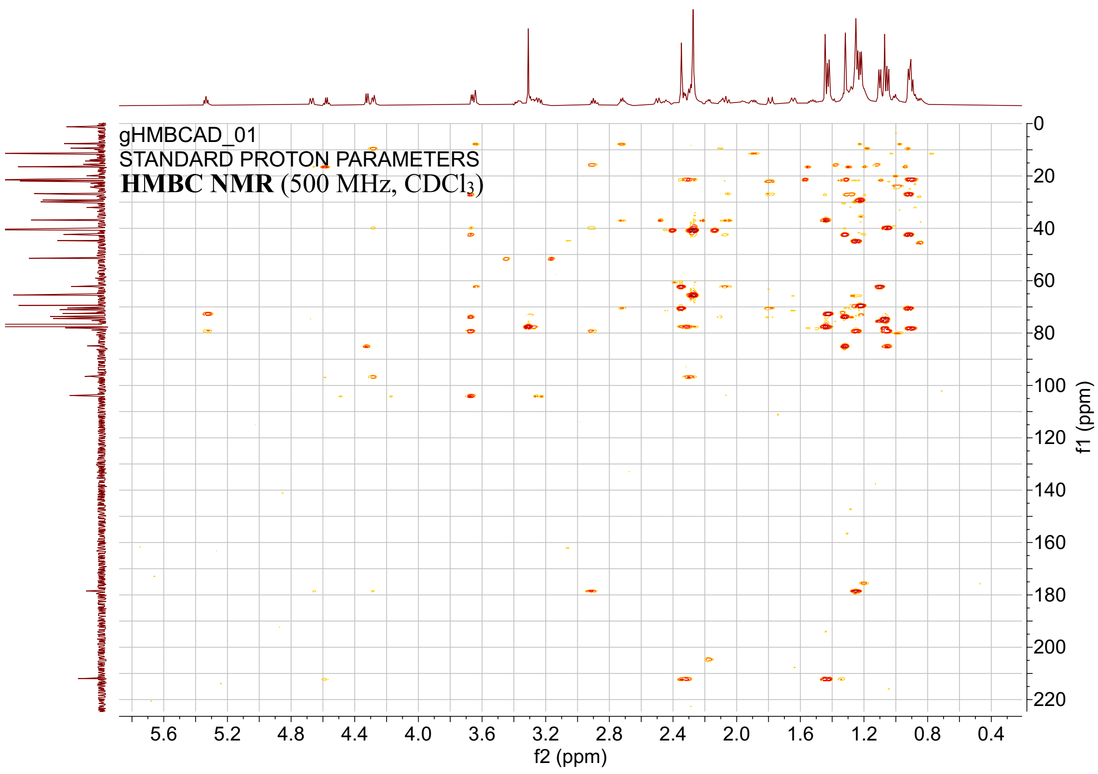
gHMBCAD_01
STANDARD PROTON PARAMETERS
HMBC NMR (500 MHz, CDCl3)
f1 (ppm)
f2 (ppm)

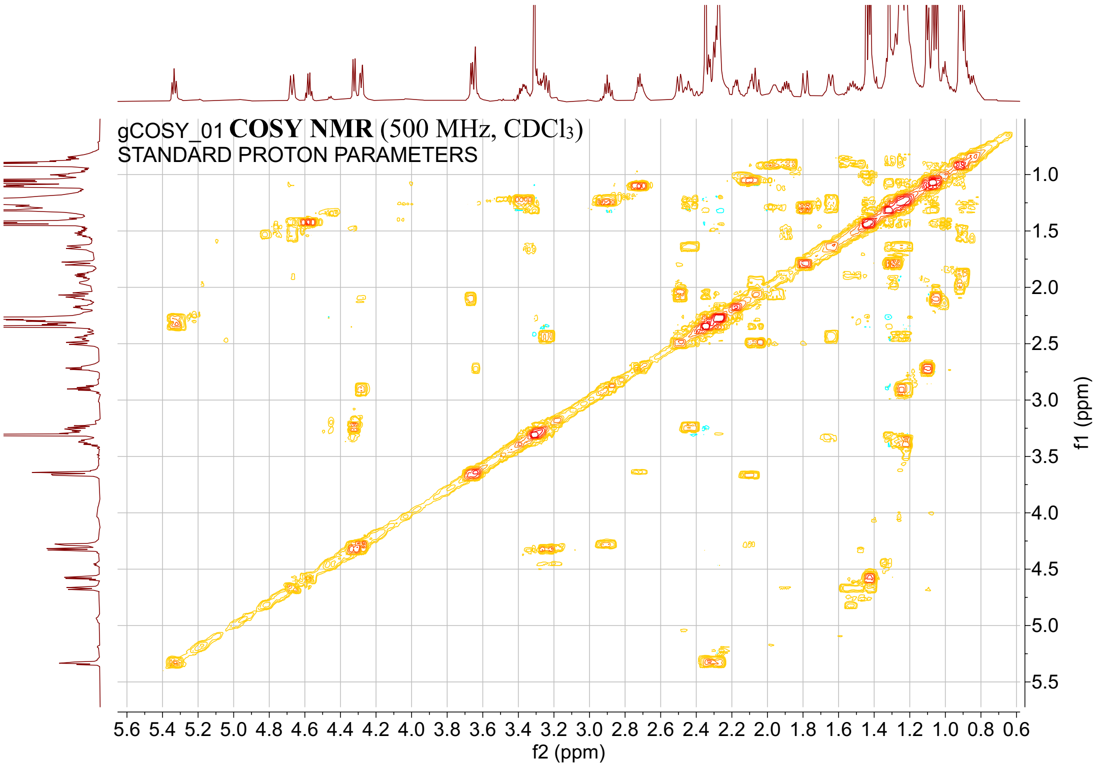

gCOSY_01 COSY NMR (500 MHz, CDCl3)
STANDARD PROTON PARAMETERS
f1 (ppm)
f2 (ppm)

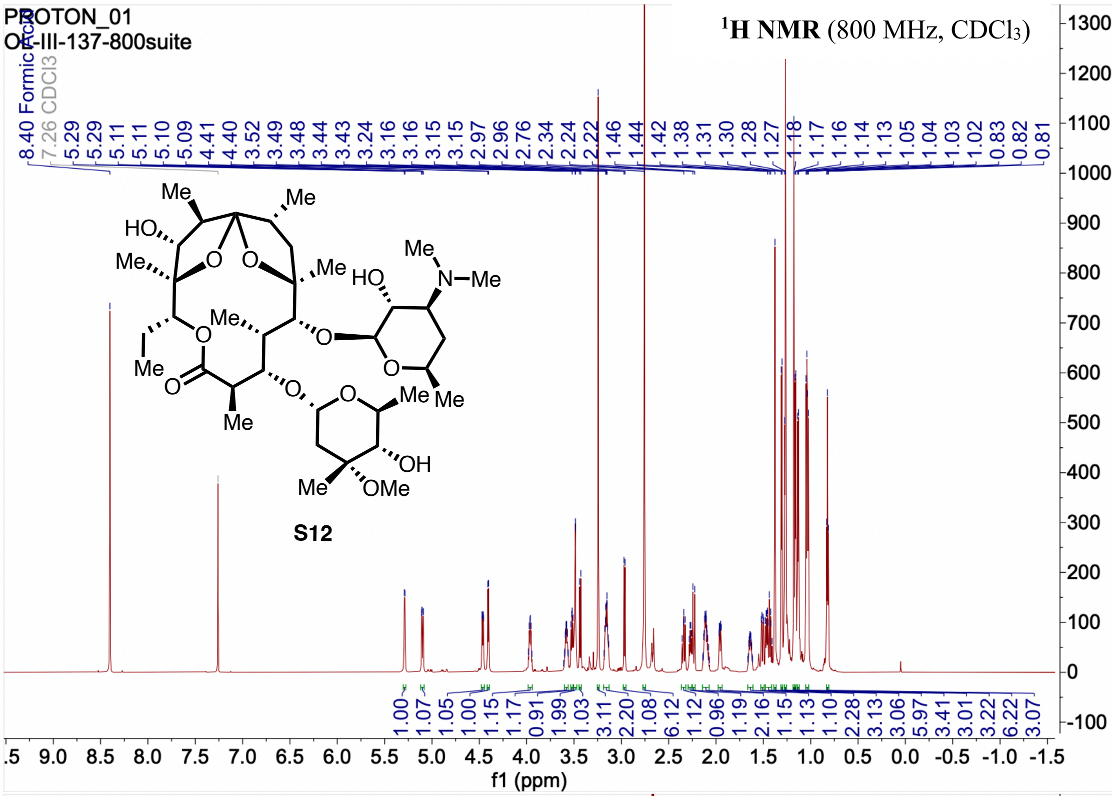
PROTON_01
OL-III-137-800suite
1H NMR (800 MHz, CDCl3)
8.40 Formic Acid
7.26 CDCl3
5.29 5.29 5.11 5.11 5.10 5.09 4.41 4.40 3.52 3.49 3.48 3.44 3.43 3.24 3.16 3.16 3.15 3.15 2.97 2.96 2.76 2.34 2.24 2.22 1.46 1.44 1.42 1.38 1.31 1.30 1.28 1.27 1.18 1.17 1.16 1.14 1.13 1.05 1.04 1.03 1.02 0.83 0.82 0.81
1.00 1.07 1.05 1.00 1.15 1.17 0.91 1.99 1.03 3.11 2.20 1.08 6.12 1.12 0.96 1.19 2.16 1.15 1.13 1.10 2.28 3.13 3.06 5.97 3.41 3.01 3.22 6.22 3.07
f1 (ppm)
S12

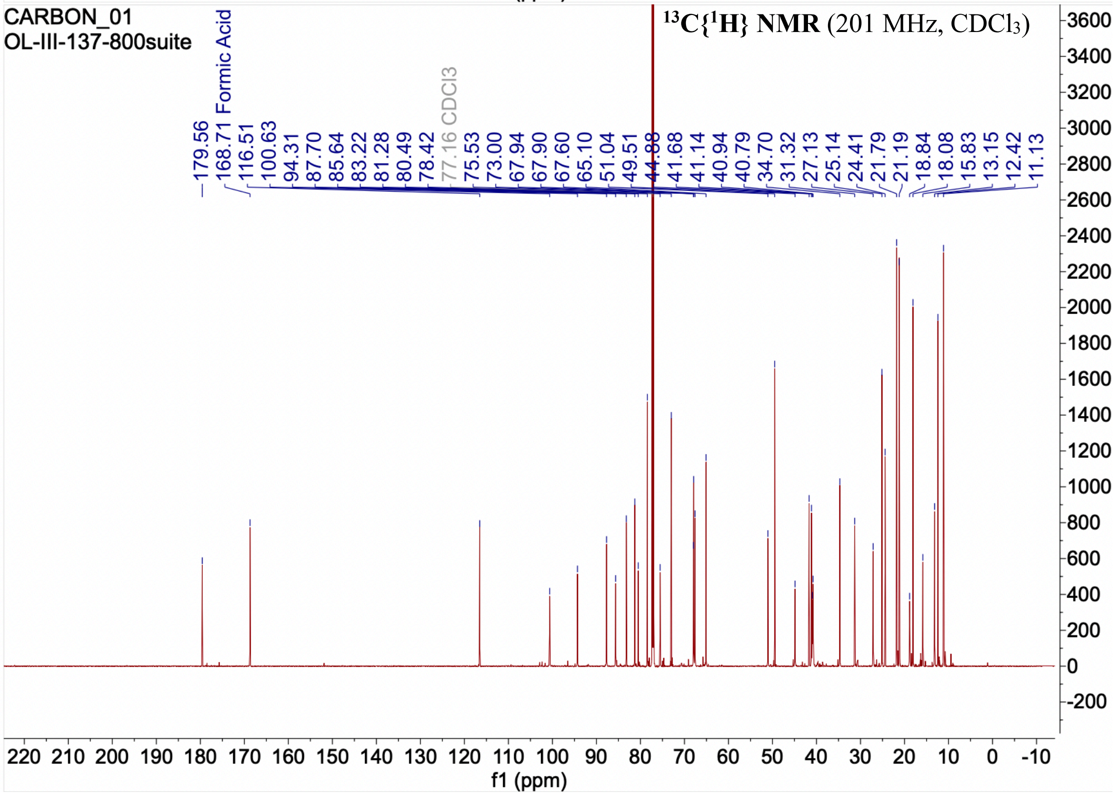
CARBON_01
OL-III-137-800suite
13C{1H} NMR (201 MHz, CDCl3)
168.71 Formic Acid
77.16 CDCl3
179.56 116.51 100.63 94.31 87.70 85.64 83.22 81.28 80.49 78.42 75.53 73.00 67.94 67.90 67.60 65.10 51.04 49.51 44.88 41.68 41.14 40.94 40.79 34.70 31.32 27.13 25.14 24.41 21.79 21.19 18.84 18.08 15.83 13.15 12.42 11.13
f1 (ppm)

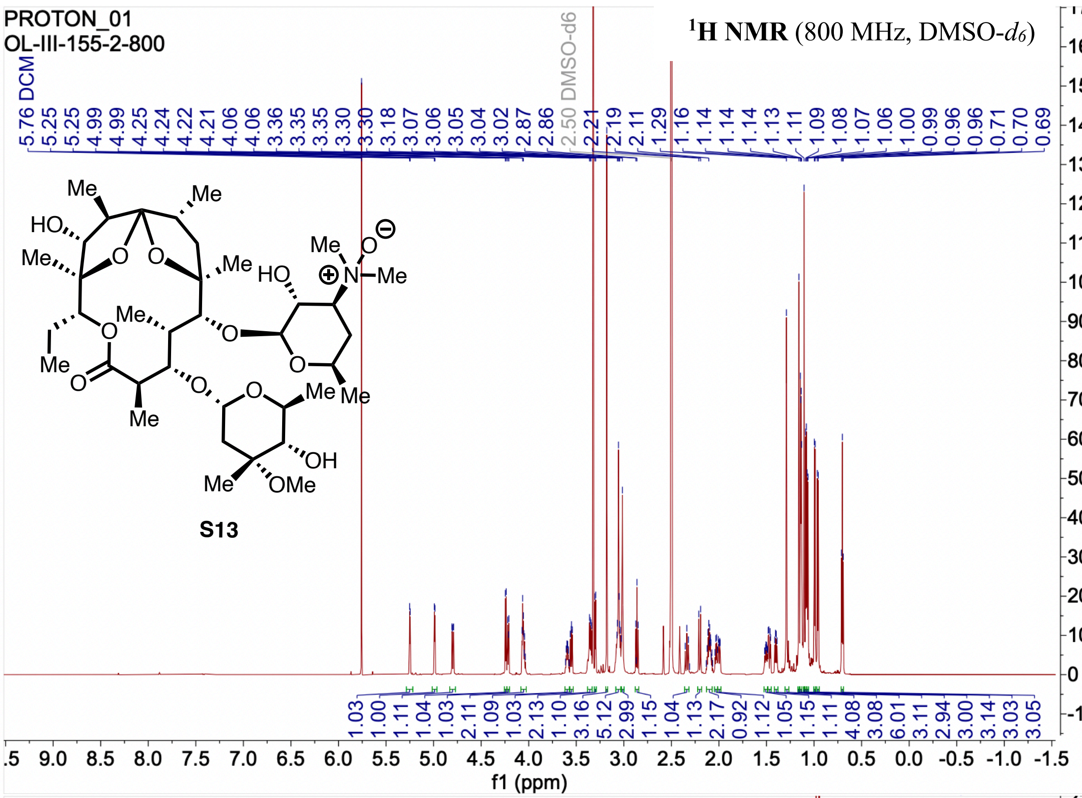

PROTON_01
OL-III-155-2-800
¹H NMR (800 MHz, DMSO-d6)
5.76 DCM
5.25
5.25
4.99
4.99
4.25
4.24
4.22
4.21
4.06
4.06
3.36
3.35
3.35
3.30
3.30
3.18
3.07
3.06
3.05
3.04
3.02
2.87
2.86
2.50 DMSO-d6
2.21
2.19
2.11
1.29
1.16
1.14
1.14
1.14
1.13
1.11
1.09
1.08
1.07
1.06
1.00
0.99
0.96
0.96
0.71
0.70
0.69
S13
1.03
1.00
1.11
1.04
1.03
2.11
1.09
1.03
2.13
1.10
3.16
5.12
2.99
1.15
1.04
1.13
2.17
0.92
1.12
1.05
1.15
1.11
4.08
3.08
6.01
3.11
2.94
3.00
3.14
3.03
3.05
f1 (ppm)

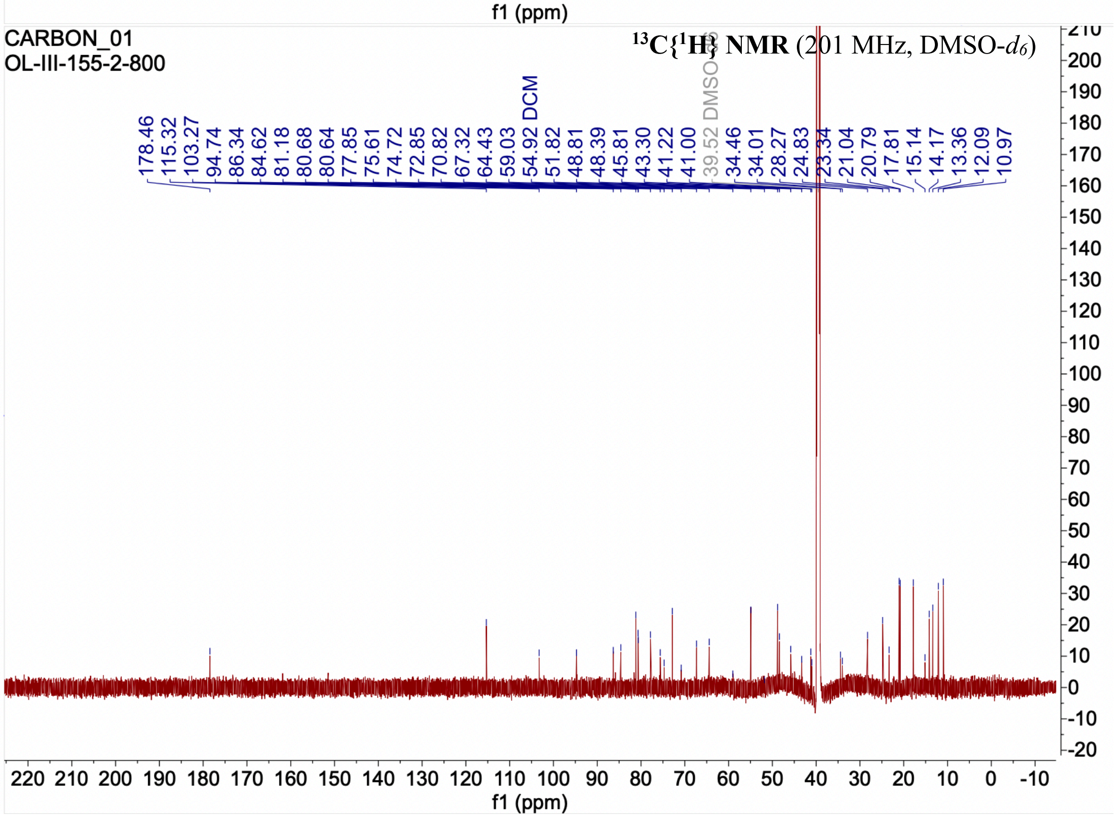

CARBON_01
OL-III-155-2-800
¹³C{¹H} NMR (201 MHz, DMSO-d6)
178.46
115.32
103.27
94.74
86.34
84.62
81.18
80.68
80.64
77.85
75.61
74.72
72.85
70.82
67.32
64.43
59.03
54.92 DCM
51.82
48.81
48.39
45.81
43.30
41.22
41.00
39.52 DMSO-d6
34.46
34.01
28.27
24.83
23.34
21.04
20.79
17.81
15.14
14.17
13.36
12.09
10.97
f1 (ppm)

HSQC NMR (800 MHz, DMSO-d_6)

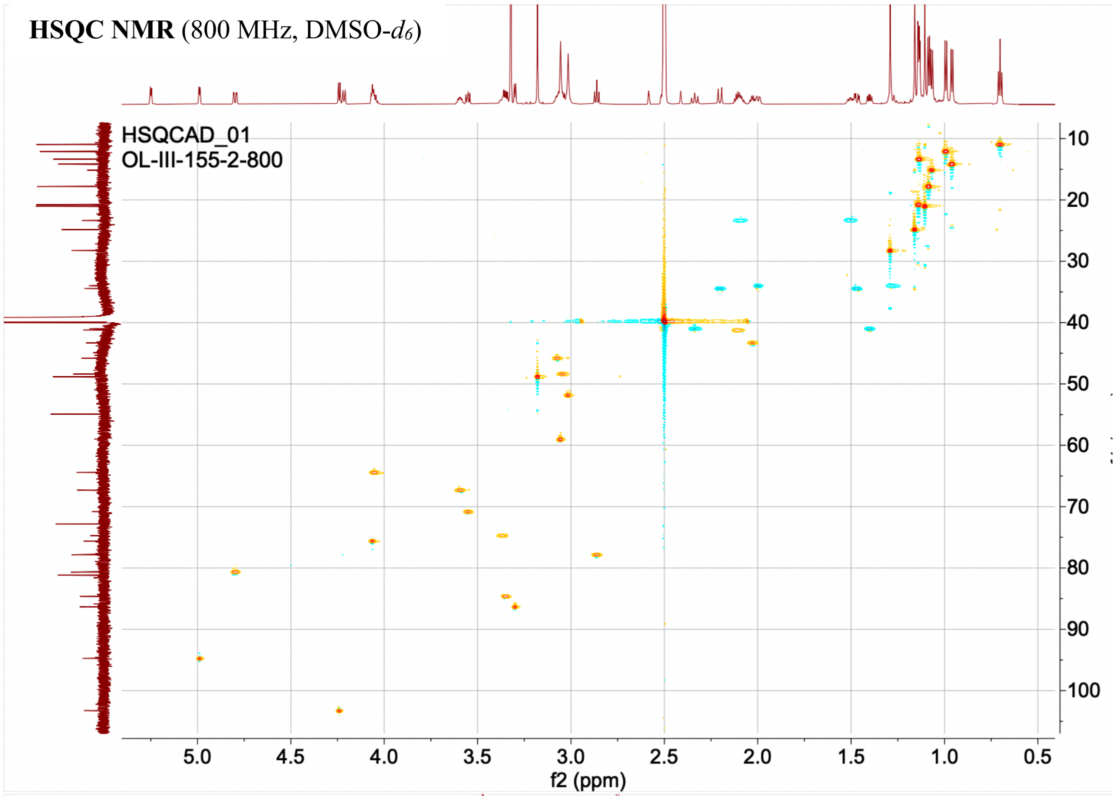

HMBC NMR (800 MHz, DMSO-d_6)

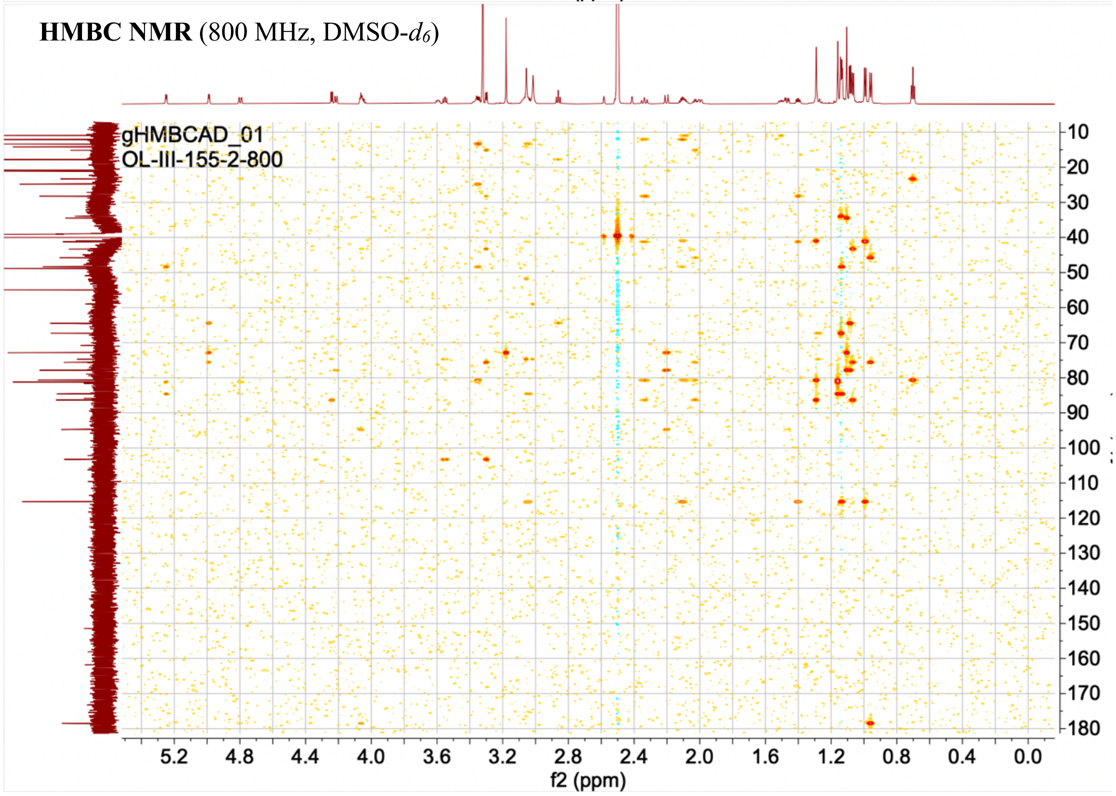

COSY NMR (800 MHz, DMSO-d_6)

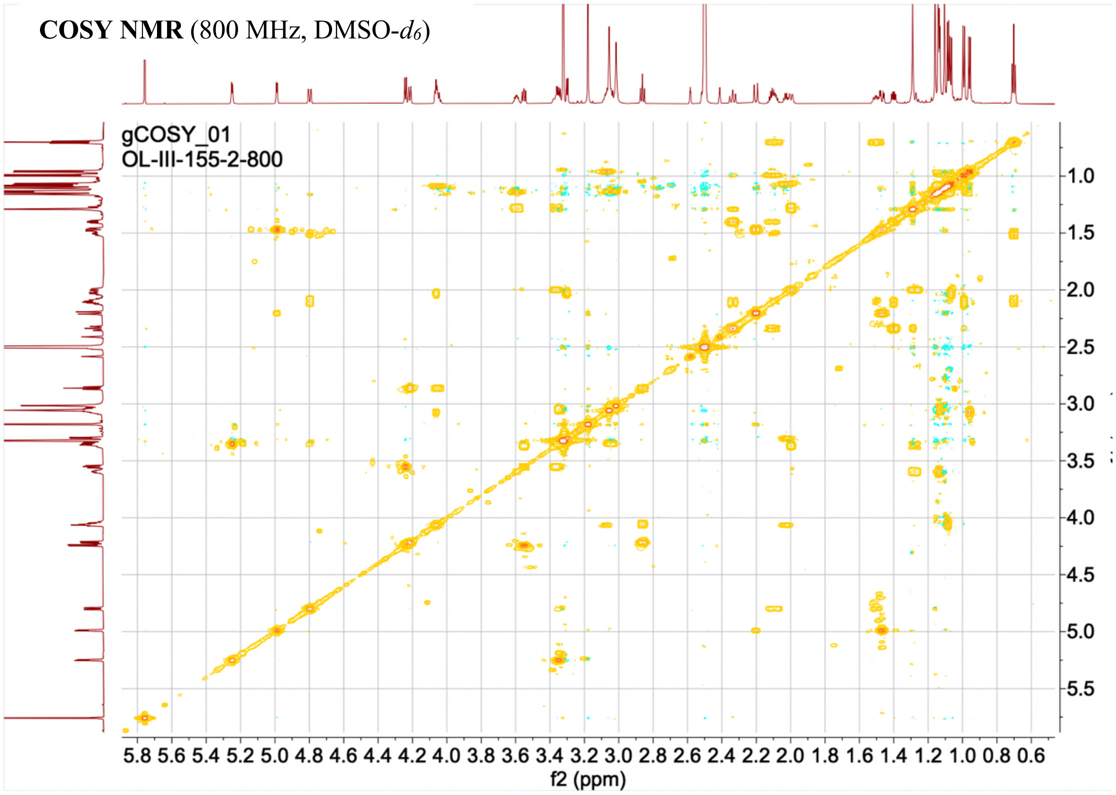

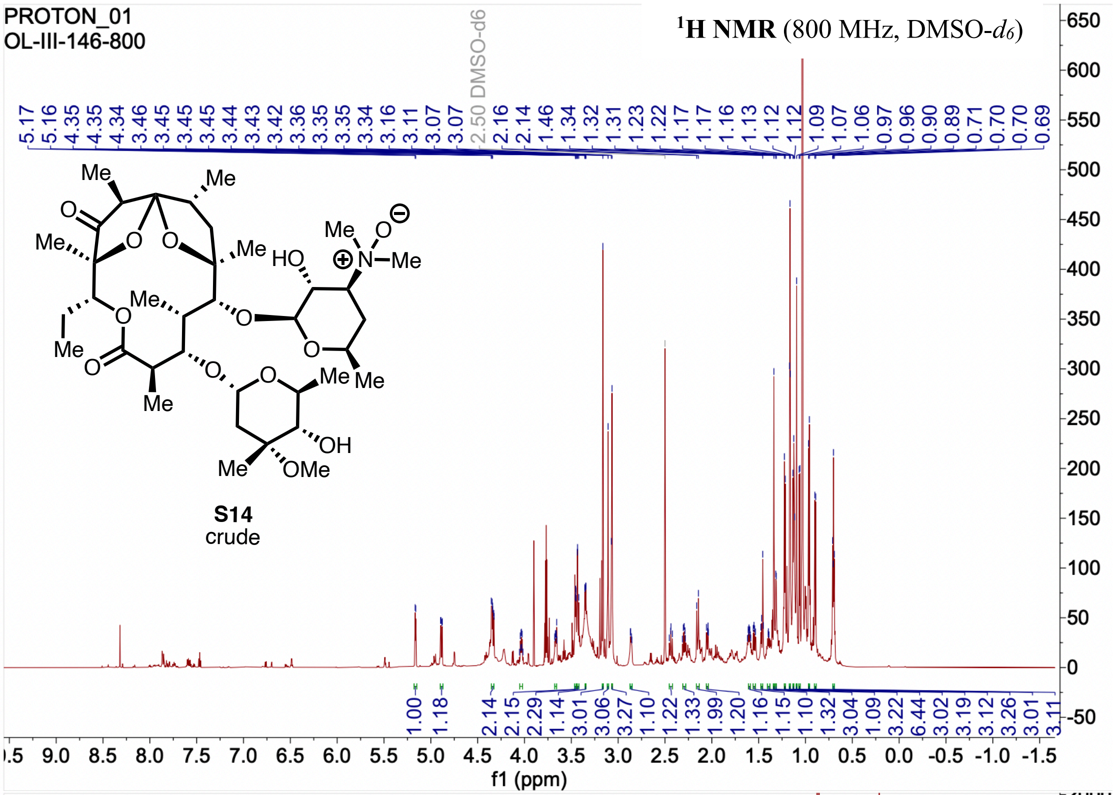
PROTON_01
OL-III-146-800
1H NMR (800 MHz, DMSO-d6)
2.50 DMSO-d6
5.17 5.16 4.35 4.35 4.34 3.46 3.45 3.45 3.45 3.44 3.43 3.42 3.36 3.35 3.35 3.34 3.16 3.11 3.07 3.07 2.50 2.16 2.14 1.46 1.34 1.32 1.31 1.23 1.22 1.17 1.17 1.16 1.13 1.12 1.12 1.09 1.07 1.06 0.97 0.96 0.90 0.89 0.71 0.70 0.70 0.69
Me
Me
O
Me
O
O
Me
HO
Me
O
N
Me
Me
O
O
Me
O
O
Me
Me
Me
Me
OH
Me
OMe
S14
crude
1.00 1.18 2.14 2.15 2.29 1.14 3.01 3.06 3.27 1.10 1.22 1.33 1.99 1.20 1.16 1.15 1.10 1.32 3.04 1.09 3.22 6.44 3.02 3.19 3.12 3.26 3.01 3.11
f1 (ppm)

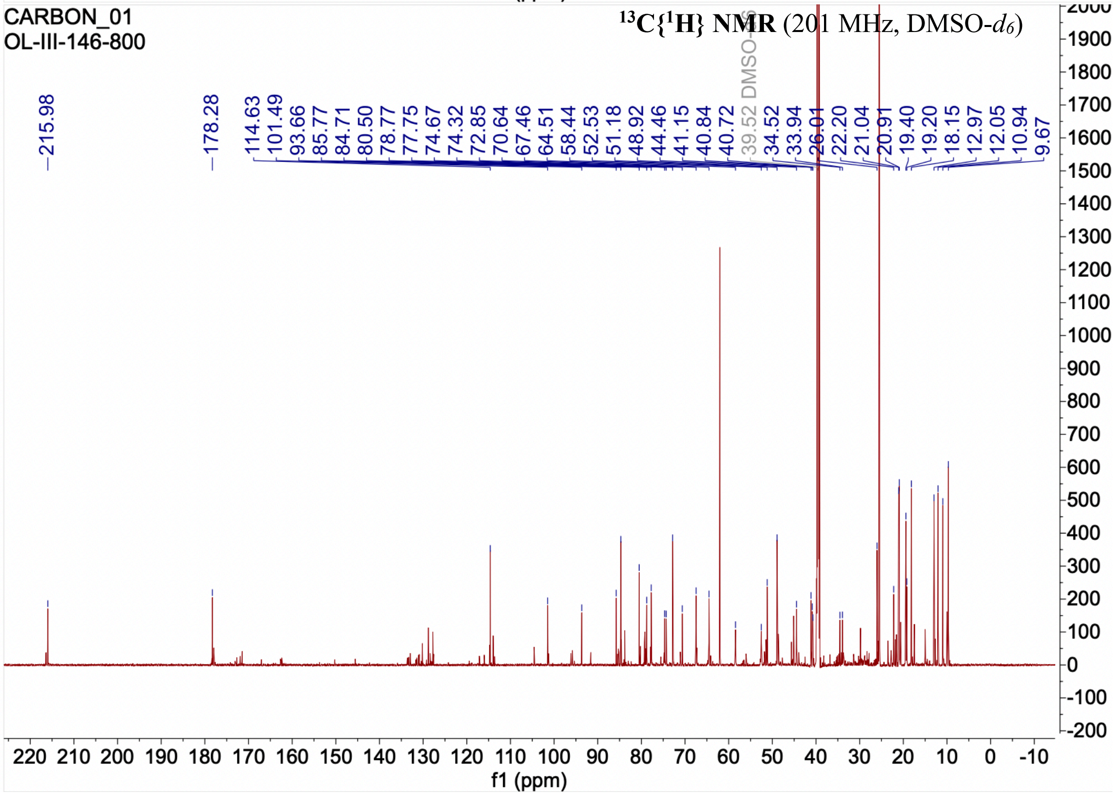
CARBON_01
OL-III-146-800
13C{1H} NMR (201 MHz, DMSO-d6)
39.52 DMSO-[illegible]
215.98 178.28 114.63 101.49 93.66 85.77 84.71 80.50 78.77 77.75 74.67 74.32 72.85 70.64 67.46 64.51 58.44 52.53 51.18 48.92 44.46 41.15 40.84 40.72 39.52 34.52 33.94 26.01 22.20 21.04 20.91 19.40 19.20 18.15 12.97 12.05 10.94 9.67
f1 (ppm)

HSQC NMR (800 MHz, DMSO-d_6)

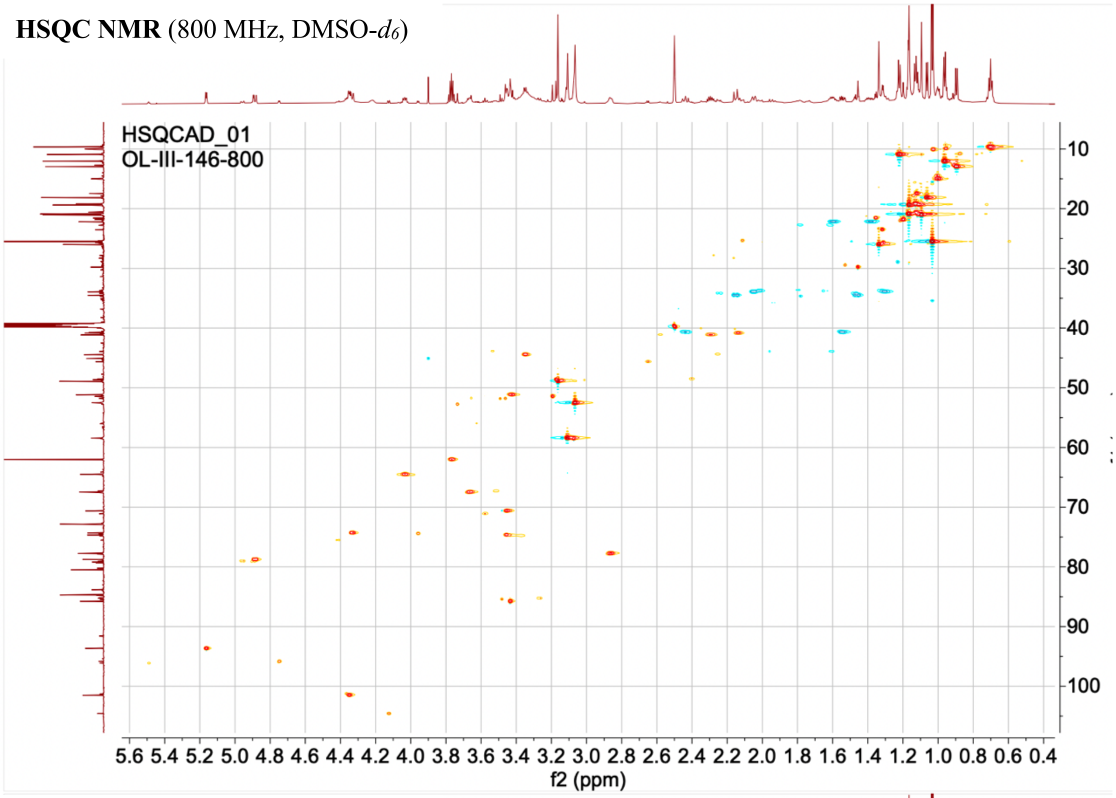

HMBC NMR (800 MHz, DMSO-d_6)

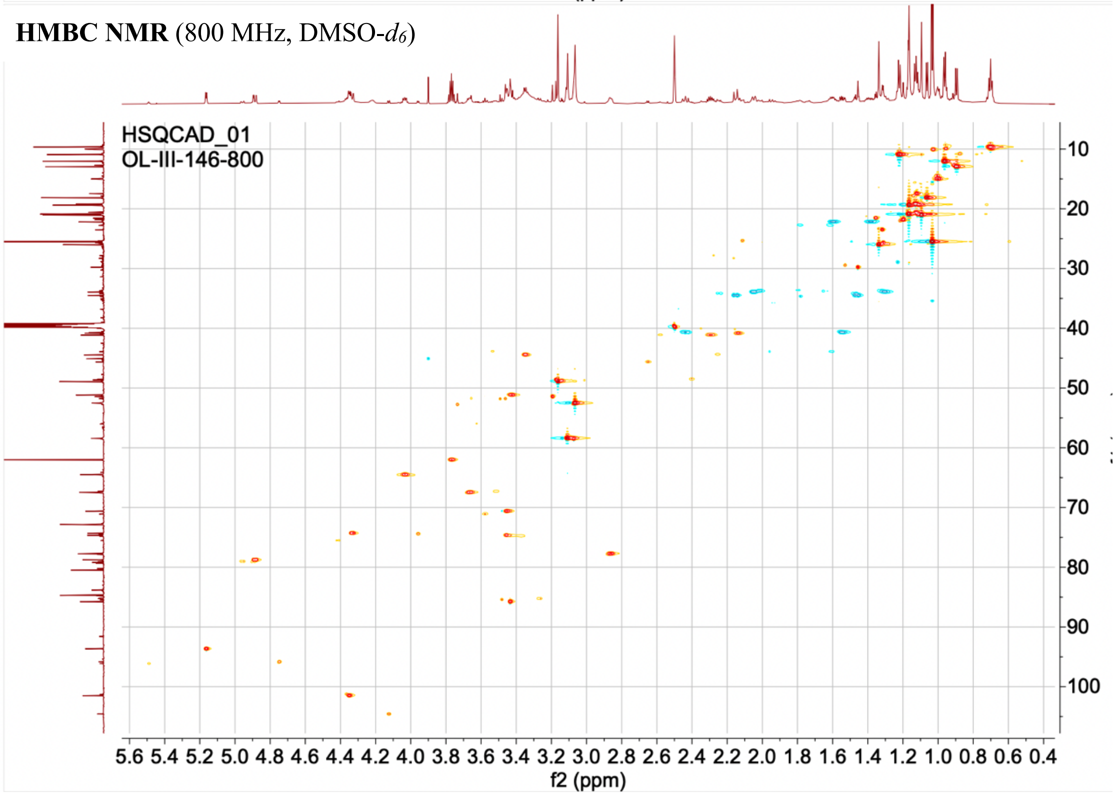

COSY NMR (800 MHz, DMSO-d_6)

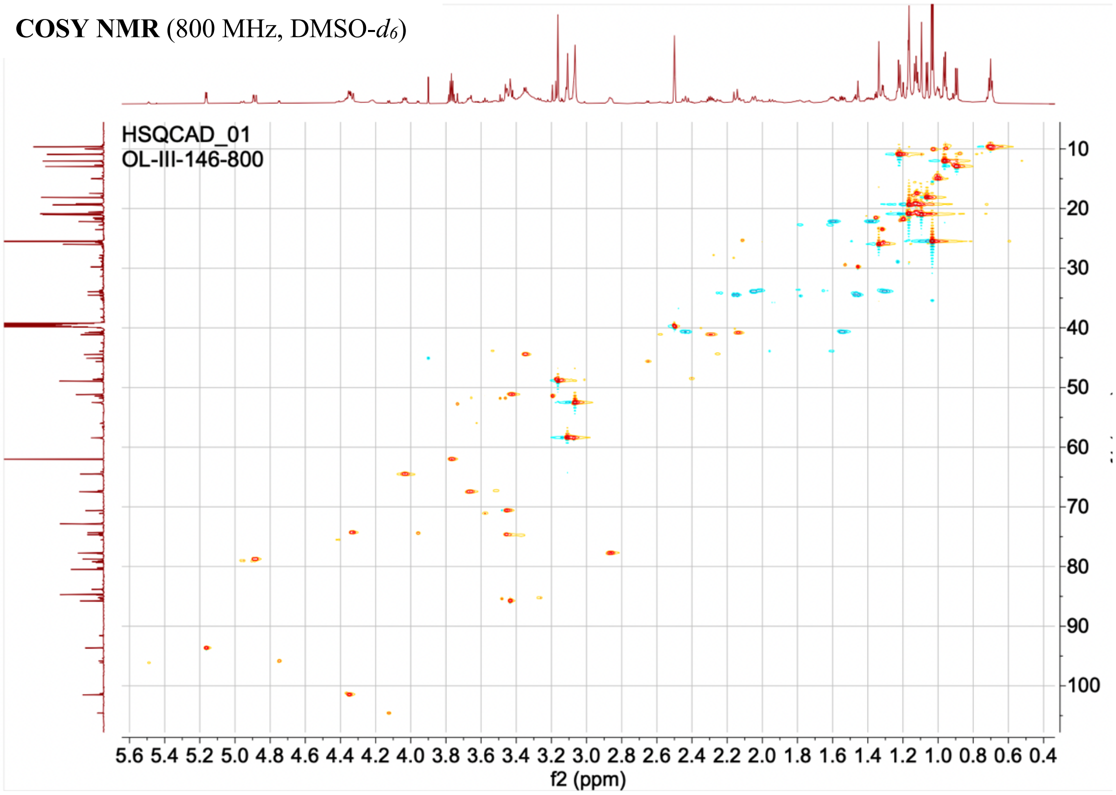

12. X-Ray Structures

5'

Experimental

The crystal was grown via slow evaporation of a mixture of MeCN, H_2O, and DCM.

Low-temperature diffraction data (ω-scans) were collected on a Rigaku MicroMax-007HF diffractometer coupled to a Saturn994+ CCD detector with Cu Kα (λ = 1.54178 Å) for the structure of 007b-24081. The diffraction images were processed and scaled using Rigaku Oxford Diffraction software.[28] The structure was solved with SHELXT and was refined against F^2 on all data by full-matrix least squares with SHELXL.[29] All non-hydrogen atoms were refined anisotropically. Hydrogen atoms were included in the model at geometrically calculated positions and refined using a riding model. The isotropic displacement parameters of all hydrogen atoms were fixed to 1.2 times the U value of the atoms to which they are linked (1.5 times for methyl groups). The third solvent pocket is likely a mix of solvents. The difference map suggests that dichloromethane is present at half occupancy. Beyond constraining the site occupancy factors to 0.5, no additional restraints or constraints were needed for a stable refinement. Two low-angle reflections were improperly recorded due to instrument artifacts. These were omitted from the least square refinement. The full numbering scheme of compound 007b-24081 can be found in the full details of the X-ray structure determination (CIF), which is included as Supporting Information. CCDC number 2493095 (007b-24081) contains the supplementary crystallographic data for this paper. These data can be obtained free of charge from The Cambridge Crystallographic Data Center via www.ccdc.cam.ac.uk/data_request/cif.

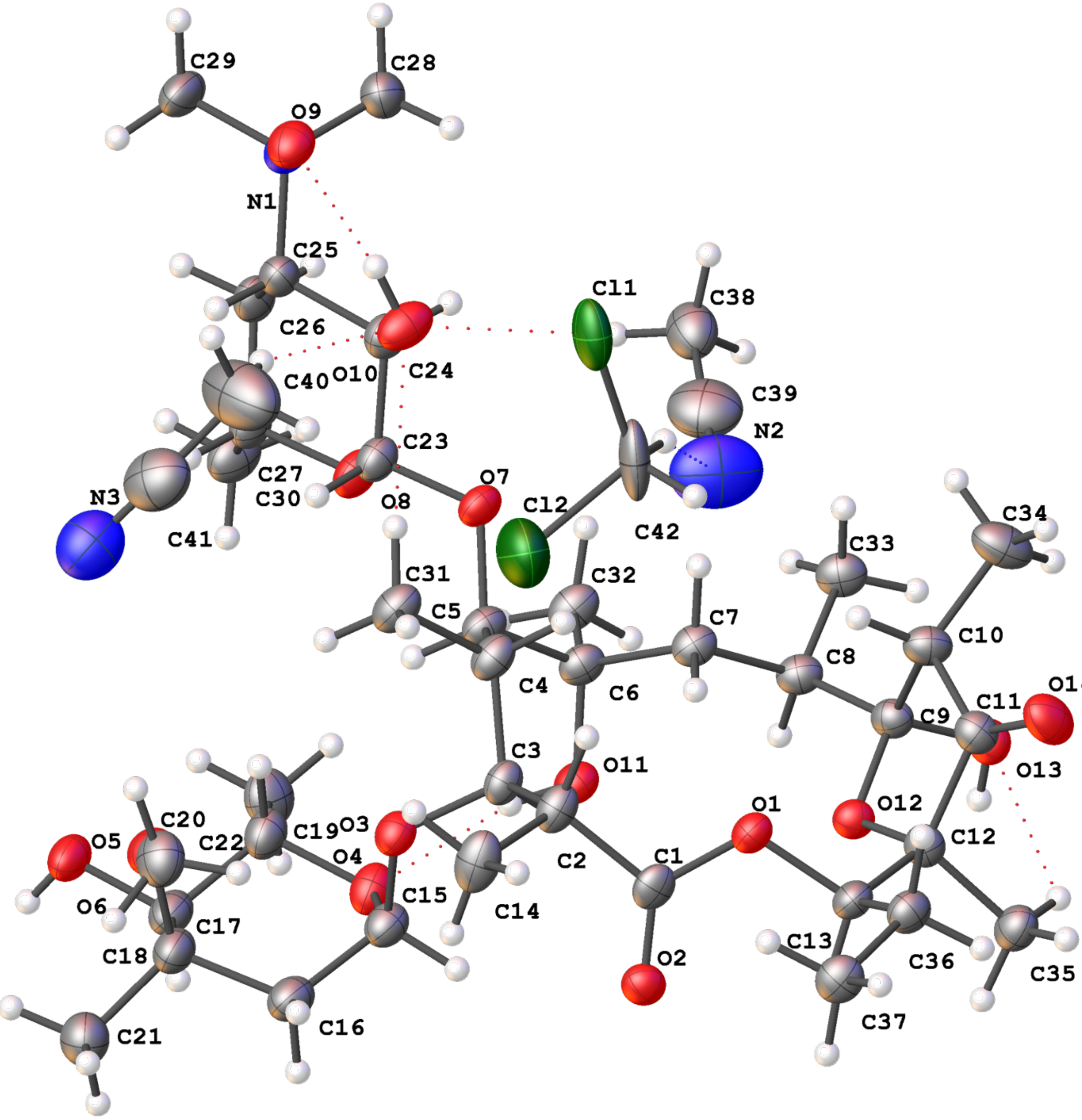

Figure S27. The complete numbering scheme of 007b-24081 with 50% thermal ellipsoid probability levels. The hydrogen atoms are shown as circles for clarity.

Table S15. Crystal data and structure refinement for 007b-24081.

Identification code	007b-24081	
Empirical formula	$C_{41.50}H_{72}ClN_3O_{14}$	
Formula weight	872.47	
Temperature	93(2) K	
Wavelength	1.54184 Å	
Crystal system	Orthorhombic	
Space group	$P2_12_12_1$	
Unit cell dimensions	a = 10.547(2) Å	a= 90°
	b = 11.633(3) Å	b= 90°
	c = 39.471(7) Å	g = 90°
Volume	4842.8(18) $Å^3$	
Z	4	
Density (calculated)	1.197 Mg/m^3	
Absorption coefficient	1.222 mm^{-1}	
F(000)	1884	
Crystal size	0.300 x 0.200 x 0.100 mm^3	
Crystal color and habit	colorless plate	
Diffractometer	Rigaku Saturn 944+ CCD	
θ range for data collection	4.412 to 66.883°.	
Index ranges	$-12 \leq h \leq 12$, $-12 \leq k \leq 13$, $-46 \leq l \leq 46$	
Reflections collected	31474	
Independent reflections	8392 [R(int) = 0.0787]	
Observed reflections (I > 2σ(I))	8177	
Completeness to θ = 66.883°	98.6 %	
Absorption correction	Semi-empirical from equivalents	
Max. and min. transmission	1.00000 and 0.70424	
Solution method	SHELXT-2014/5 (Sheldrick, 2014)	
Refinement method	SHELXL-2014/7 (Sheldrick, 2014)	
Data / restraints / parameters	8392 / 0 / 569	
Goodness-of-fit on F^2	1.065	
Final R indices [I>2σ(I)]	R1 = 0.0580, wR2 = 0.1722	
R indices (all data)	R1 = 0.0590, wR2 = 0.1736	
Absolute structure parameter	0.012(17)	
Largest diff. peak and hole	0.760 and -0.296 e.$Å^{-3}$	

7

Experimental

The crystal was grown via slow evaporation of $CHCl_3$.

Low-temperature diffraction data (ω-scans) were collected on a Rigaku Synergy-S diffractometer coupled to a HyPix-Arc 100 detector with Cu Kα (λ = 1.54178 Å) for the structure of syn-25016. The diffraction images were processed and scaled using Rigaku Oxford Diffraction software.[28] The structure was solved with SHELXT and was refined against F^2 on all data by full-matrix least squares with SHELXL.[29] All non-hydrogen atoms were refined anisotropically. Hydrogen atoms were included in the model at geometrically calculated positions and refined using a riding model. The isotropic displacement parameters of all hydrogen atoms were fixed to 1.2 times the U value of the atoms to which they are linked (1.5 times for methyl groups). The full numbering scheme of compound syn-25016 can be found in the full details of the X-ray structure determination (CIF), which is included as Supporting Information. CCDC number 2493094 (syn-25016) contains the supplementary crystallographic data for this paper. These data can be obtained free of charge from The Cambridge Crystallographic Data Center via www.ccdc.cam.ac.uk/data_request/cif.

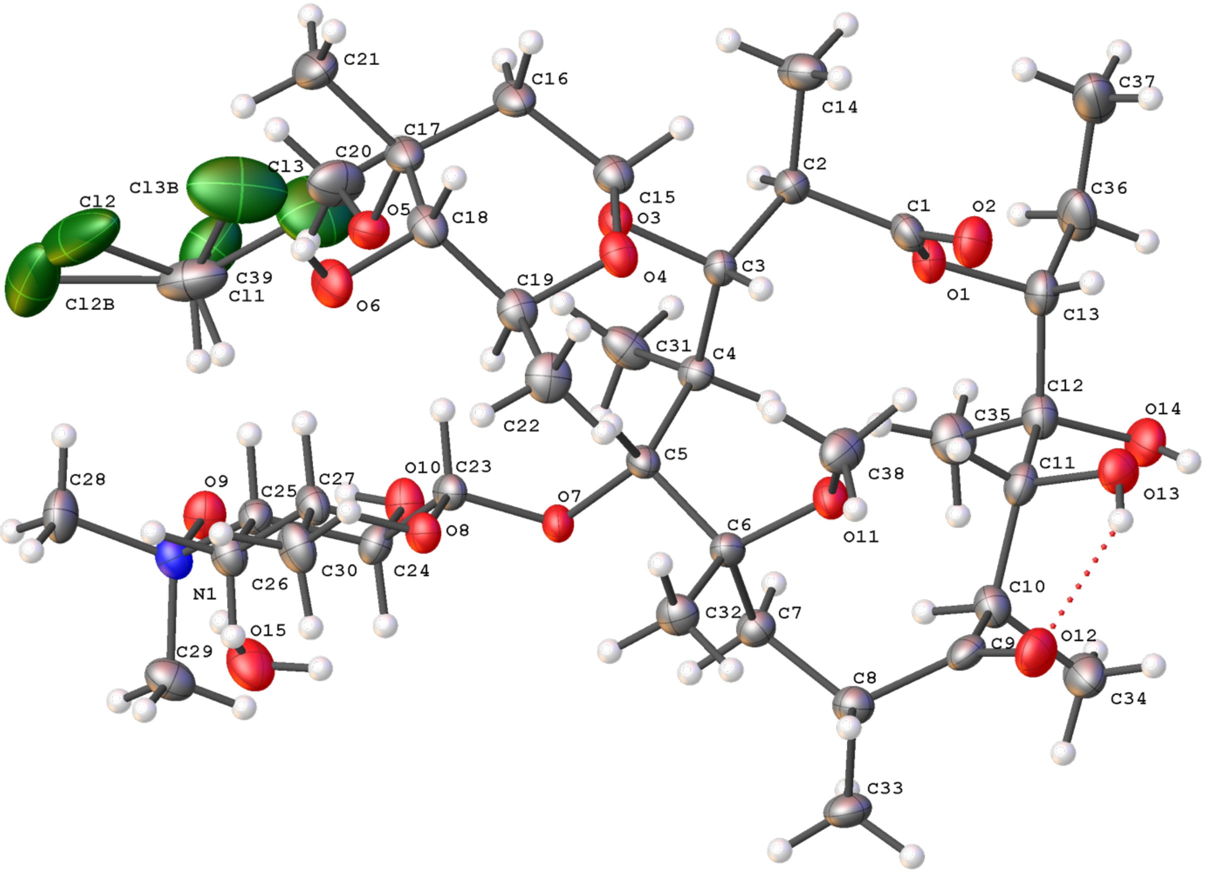

Figure S28. The complete numbering scheme of syn-25016 with 50% thermal ellipsoid probability levels. The hydrogen atoms are shown as circles for clarity.

Table S16. Crystal data and structure refinement for syn-25016.

Identification code	syn-25016	
Empirical formula	$C_{39}H_{72}Cl_3NO_{15}$	
Formula weight	901.32	
Temperature	100(2) K	
Wavelength	1.54184 Å	
Crystal system	Monoclinic	
Space group	$P2_1$	
Unit cell dimensions	a = 9.09480(10) Å	α= 90°
	b = 26.4164(4) Å	β= 92.9300(10)°
	c = 9.63000(10) Å	γ = 90°
Volume	2310.60(5) $Å^3$	
Z	2	
Density (calculated)	1.295 Mg/m^3	
Absorption coefficient	2.338 mm^{-1}	
F(000)	968	
Crystal size	0.200 x 0.200 x 0.050 mm^3	
Crystal color and habit	colorless plate	
Diffractometer	XtaLAB Synergy, Dualflex, HyPix-Arc 100	
θ range for data collection	3.346 to 66.982°.	
Index ranges	$-10 \le h \le 10$, $-30 \le k \le 31$, $-11 \le l \le 11$	
Reflections collected	32626	
Independent reflections	7884 [R(int) = 0.0969]	
Observed reflections (I > 2σ(I))	7463	
Completeness to θ = 66.982°	99.1 %	
Absorption correction	Semi-empirical from equivalents	
Max. and min. transmission	1.00000 and 0.85701	
Solution method	SHELXT-2014/5 (Sheldrick, 2014)	
Refinement method	SHELXL-2014/7 (Sheldrick, 2014)	
Data / restraints / parameters	7884 / 13 / 563	
Goodness-of-fit on F^2	1.107	
Final R indices [I>2σ(I)]	R1 = 0.0540, wR2 = 0.1502	
R indices (all data)	R1 = 0.0560, wR2 = 0.1527	
Absolute structure parameter	0.032(17)	
Largest diff. peak and hole	0.587 and -0.412 e.$Å^{-3}$	

15
Experimental

The crystal was grown via lyophilization of a mixture of MeCN, H_2O, and DMSO.

Low-temperature diffraction data (ω-scans) were collected on a Rigaku MicroMax-007HF diffractometer coupled to a Saturn994+ CCD detector with Cu Kα (λ = 1.54178 Å) for the structure of 007b-25056. The diffraction images were processed and scaled using Rigaku Oxford Diffraction software.[28] The structure was solved with SHELXT and was refined against F^2 on all data by full-matrix least squares with SHELXL.[29] All non-hydrogen atoms were refined anisotropically. Hydrogen atoms were included in the model at geometrically calculated positions and refined using a riding model. The isotropic displacement parameters of all hydrogen atoms were fixed to 1.2 times the U value of the atoms to which they are linked (1.5 times for methyl groups). The full numbering scheme of compound 007b-25056 can be found in the full details of the X-ray structure determination (CIF), which is included as Supporting Information. CCDC number 2493093 (007b-25056) contains the supplementary crystallographic data for this paper. These data can be obtained free of charge from The Cambridge Crystallographic Data Center via www.ccdc.cam.ac.uk/data_request/cif.

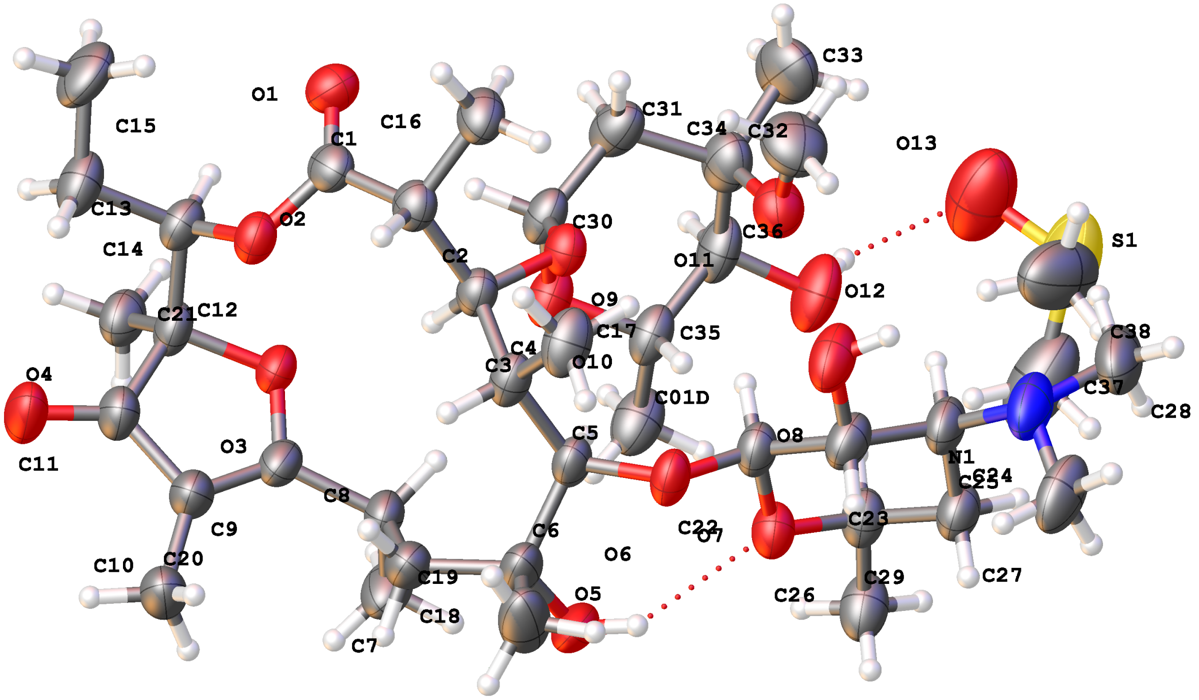

Figure S29. The complete numbering scheme of 007b-25056 with 50% thermal ellipsoid probability levels. The hydrogen atoms are shown as circles for clarity.

Table S17. Crystal data and structure refinement for 007b-25056.

Identification code	007b-25056	
Empirical formula	$C_{39}H_{69}NO_{13}S$	
Formula weight	792.01	
Temperature	100 K	
Wavelength	1.54184 Å	
Crystal system	Orthorhombic	
Space group	$P2_12_12_1$	
Unit cell dimensions	a = 10.08166(17) Å	α= 90°
	b = 13.7632(2) Å	β= 90°
	c = 30.2820(4) Å	γ = 90°
Volume	4201.81(12) $Å^3$	
Z	4	
Density (calculated)	1.252 Mg/m^3	
Absorption coefficient	1.204 mm^{-1}	
F(000)	1720	
Crystal size	0.23 x 0.1 x 0.08 mm^3	
Crystal color and habit	colourless prism	
Diffractometer	XtaLAB AFC11 (RCD3): quarter-chi single	
θ range for data collection	3.527 to 68.922°.	
Index ranges	$-12 \le h \le 12$, $-16 \le k \le 16$, $-36 \le l \le 36$	
Reflections collected	99976	
Independent reflections	7690 [R(int) = 0.1892]	
Observed reflections (I > 2σ(I))	7289	
Completeness to θ = 67.684°	99.6 %	
Absorption correction	Semi-empirical from equivalents	
Max. and min. transmission	1.00000 and 0.84689	
Solution method	SHELXT (Sheldrick, 2015)	
Refinement method	SHELXL 2019/3 (Sheldrick, 2015)	
Data / restraints / parameters	7690 / 64 / 505	
Goodness-of-fit on F^2	1.052	
Final R indices [I>2σ(I)]	R1 = 0.0705, wR2 = 0.1979	
R indices (all data)	R1 = 0.0762, wR2 = 0.2051	
Absolute structure parameter	0.03(2)	
Largest diff. peak and hole	0.516 and -0.754 e.$Å^{-3}$	

13. References

(1) Mintz, M. J.; Walling, C. *t*-Butyl Hypochlorite. *Org. Synth.* **1969**, *49*.
(2) Fulmer, G. R.; Miller, A. J. M.; Sherden, N. H.; Gottlieb, H. E.; Nudelman, A.; Stoltz, B. M.; Bercaw, J. E.; Goldberg, K. I. NMR Chemical Shifts of Trace Impurities: Common Laboratory Solvents, Organics, and Gases in Deuterated Solvents Relevant to the Organometallic Chemist. *Organometallics* **2010**, *29*, 2176-2179.
(3) Rein, J.; Rozema, S. D.; Langner, O. C.; Zacate, S. B.; Hardy, M. A.; Siu, J. C.; Mercado, B. Q.; Sigman, M. S.; Miller, S. J.; Lin, S. Generality-oriented optimization of enantioselective aminoxyl radical catalysis. *Science* **2023**, *380*, 706-712.
(4) Shibuya, M.; Tomizawa, M.; Suzuki, I.; Iwabuchi, Y. 2-Azaadamantane *N*-Oxyl (AZADO) and 1-Me-AZADO: Highly Efficient Organocatalysts for Oxidation of Alcohols. *J. Am. Chem. Soc.* **2006**, *128*, 8412-8413.
(5) Yamaoka, H.; Moriya, N.; Ikunaka, M. A Practical $RuCl_3$-Catalyzed Oxidation Using Trichloroisocyanuric Acid As a Stoichiometric Oxidant under Mild Nonacidic Conditions. *Org. Process Res. Dev.* **2004**, *8*, 931-938.
(6) Song, Z. J.; Zhou, G.; Cohen, R.; Tan, L. Preparation of ABNO on Scale and Analysis by Quantitative Paramagnetic NMR. *Org. Process Res. Dev.* **2018**, *22*, 1257-1261.
(7) Marvich, H. M.; Langner, O. C.; Rozema, S. D.; Miller, S. J. CCDC 2366598. *CSD Communication* **2026**.
(8) Chalmers, B. A.; Morris, J. C.; Fairfull-Smith, K. E.; Grainger, R. S.; Bottle, S. E. A novel protecting group methodology for syntheses using nitroxides. *Chem. Commun.* **2013**, *49*, 10382-10384.
(9) Toshima, K.; Mukaiyama, S.; Yoshida, T.; Tamai, T.; Tatsuta, K. Application of efficient glycosylation of 2,6-anhydro-2-thio sugar to the total synthesis of erythromycin A. *Tetrahedron Lett.* **1991**, *32*, 6155-6158.
(10) Sulejman, A. A., B.; Lewis, J. R.; Gorjana, L. NOVEL 14 AND 15 MEMBERED-RING COMPOUNDS. WO101590A1. 2005.
(11) Bannwarth, C.; Ehlert, S.; Grimme, S. GFN2-xTB—An Accurate and Broadly Parametrized Self-Consistent Tight-Binding Quantum Chemical Method with Multipole Electrostatics and Density-Dependent Dispersion Contributions. *J. Chem. Theory Comput.* **2019**, *15*, 1652-1671.
(12) Ehlert, S.; Stahn, M.; Spicher, S.; Grimme, S. Robust and Efficient Implicit Solvation Model for Fast Semiempirical Methods. *J. Chem. Theory Comput.* **2021**, *17*, 4250-4261.
(13) Spicher, S.; Grimme, S. Robust Atomistic Modeling of Materials, Organometallic, and Biochemical Systems. *Angew. Chem., Int. Ed.* **2020**, *59*, 15665-15673.
(14) Pracht, P.; Bohle, F.; Grimme, S. Automated exploration of the low-energy chemical space with fast quantum chemical methods. *Phys. Chem. Chem. Phys.* **2020**, *22*, 7169-7192.
(15) *TsCoDe - Transition State Conformational Docker*. 2020–2024. https://github.com/ntampellini/TSCoDe (accessed 2025 October 27).
(16) Grimme, S.; Hansen, A.; Ehlert, S.; Mewes, J. M. r(2)SCAN-3c: A "Swiss army knife" composite electronic-structure method. *J. Chem. Phys.* **2021**, *154*, 064103.
(17) Cossi, M.; Rega, N.; Scalmani, G.; Barone, V. Energies, structures, and electronic properties of molecules in solution with the C-PCM solvation model. *J. Comput. Chem.* **2003**, *24*, 669-681.
(18) Neese, F. Software update: The ORCA program system—Version 5.0. *WIREs Computational Molecular Science* **2022**, *12*, e1606.

(19) Neese, F.; Wennmohs, F.; Becker, U.; Riplinger, C. The ORCA quantum chemistry program package. *J. Chem. Phys.* **2020**, *152*, 224108.
(20) Mardirossian, N.; Head-Gordon, M. ωB97M-V: A combinatorially optimized, range-separated hybrid, meta-GGA density functional with VV10 nonlocal correlation. *J. Chem. Phys.* **2016**, *144*, 214110.
(21) Weigend, F.; Ahlrichs, R. Balanced basis sets of split valence, triple zeta valence and quadruple zeta valence quality for H to Rn: Design and assessment of accuracy. *Phys. Chem. Chem. Phys.* **2005**, *7*, 3297-3305.
(22) Perdew, J. P.; Ernzerhof, M.; Burke, K. Rationale for mixing exact exchange with density functional approximations. *J. Chem. Phys.* **1996**, *105*, 9982-9985.
(23) Lodewyk, M. W.; Siebert, M. R.; Tantillo, D. J. Computational prediction of ^{1}H and ^{13}C chemical shifts: a useful tool for natural product, mechanistic, and synthetic organic chemistry. *Chem Rev* **2012**, *112*, 1839-1862.
(24) *Avogadro*. https://avogadro.cc/ (accessed 2025 October 27).
(25) Ager, D. J.; Sood, C. K. The complete, unambiguous assignment of the ^{13}C NMR spectrum of erythromycin A*. *Magn. Reson. Chem.* **1987**, *25*, 948-954.
(26) *Methods for Dilution Antimicrobial Susceptibility Tests for Bacteria That Grow Aerobically, 11th ed.*; Clinical and Laboratory Standards Institute (CLSI). CLSI standard M07. CLSI, 950 West Valley Road, Suite 2500, Wayne, Pennsylvania 19087 USA, 2018.
(27) *Performance Standards for Antimicrobial Susceptibility Testing. 34th ed.* ; Clinical and Laboratory Standards Institute (CLSI). CLSI supplement M100. CLSI, 950 West Valley Road, Suite 2500, Wayne, Pennsylvania 19087 USA, 2022.
(28) Rigaku OD: The Woodlands, T. CrysAlisPro. 2015.
(29) Sheldrick, G. A short history of SHELX. *Acta Crystallographica Section A* **2008**, *64*, 112-122.
