# Supplementary material for: Site-Divergent Oxidations within Venerable Macrolide Antibiotic Scaffolds Unveil Compounds with Broad Spectrum and Anti-MRSA Activities
Source: ACS Cent Sci. 2026 Mar 17;12(3):375–82. doi: 10.1021/acscentsci.5c02343 (PMC13022725; doi:10.1021/acscentsci.5c02343)
Supplement: Supplementary file 2 [file oc5c02343_si_002.zip › Erythromycin Analog Characterization 2,5',11,12/11/IR/OL-II-283.pdf]

SHIMADZU

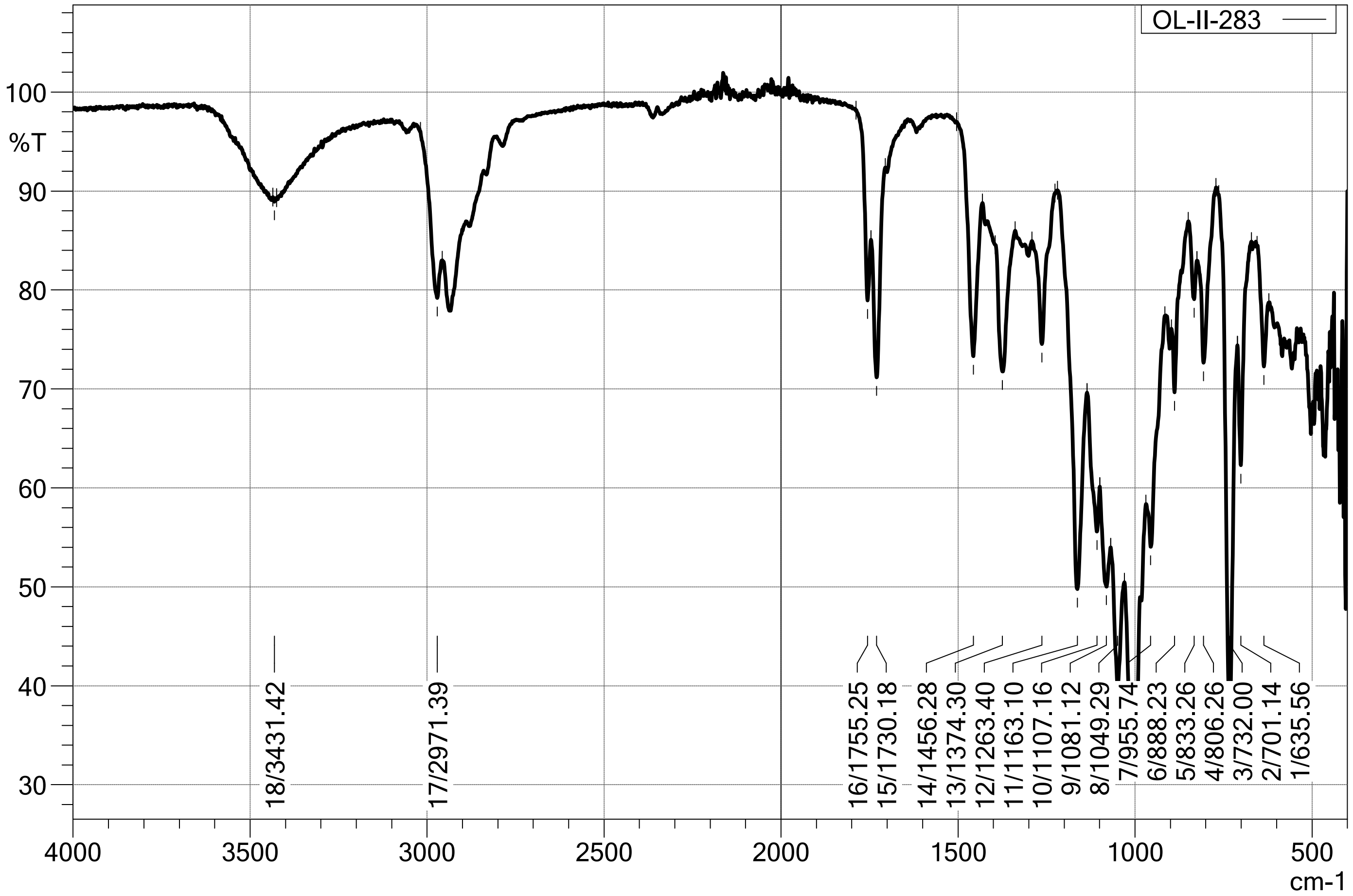

C:¥LabSolutions¥LabSolutionsIR¥Data¥Miller_OliviaL¥OL-II-283.ispd

	Item	Value
2	Sample name	
3	Sample ID	
4	Option	
5	Intensity Mode	%Transmittance
6	Apodization	Happ-Genzel
9	No. of Scans	32
10	Resolution	2 cm-1

	Peak	Intensity	Corr. Intensity	Base (H)	Base (L)	Area	Corr. Area	Comment
1	635.56	72.28	8.82	654.84	622.05	727.968	125.231	
2	701.14	62.29	14.69	710.78	671.24	960.608	156.226	
3	732.00	38.12	42.40	763.82	710.78	1878.290	925.463	
4	806.26	72.65	12.88	824.58	771.54	976.967	269.906	
5	833.26	79.07	5.27	849.66	824.58	436.307	58.961	
6	888.23	69.67	7.88	896.91	878.59	474.203	64.311	
7	955.74	54.04	9.09	969.25	915.24	1923.234	189.113	
8	1049.29	39.94	12.29	1068.58	1030.01	2063.674	220.927	
9	1081.12	50.02	6.46	1099.44	1068.58	1444.931	119.580	
10	1107.16	55.59	6.58	1135.13	1099.44	1398.700	145.283	
11	1163.10	49.77	26.68	1219.03	1135.13	2529.517	839.426	
12	1263.40	74.53	12.47	1291.36	1225.78	1127.961	299.054	
13	1374.30	71.76	13.33	1394.56	1338.62	1169.213	345.765	
14	1456.28	73.32	18.32	1503.54	1431.21	982.045	468.210	

15	1730.18	71.16	16.70	1745.61	1705.10	751.720	295.098	
16	1755.25	78.96	9.11	1788.04	1745.61	416.219	60.997	
17	2971.39	79.18	6.87	3018.65	2956.93	823.353	176.036	
18	3431.42	88.93	0.44	3435.28	3424.67	115.419	2.525	
