# Supplementary material for: Site-Divergent Oxidations within Venerable Macrolide Antibiotic Scaffolds Unveil Compounds with Broad Spectrum and Anti-MRSA Activities
Source: ACS Cent Sci. 2026 Mar 17;12(3):375–82. doi: 10.1021/acscentsci.5c02343 (PMC13022725; doi:10.1021/acscentsci.5c02343)
Supplement: Supplementary file 2 [file oc5c02343_si_002.zip › Erythromycin Analog Characterization 2,5',11,12/5'/IR/OL-II-275.pdf]

SHIMADZU

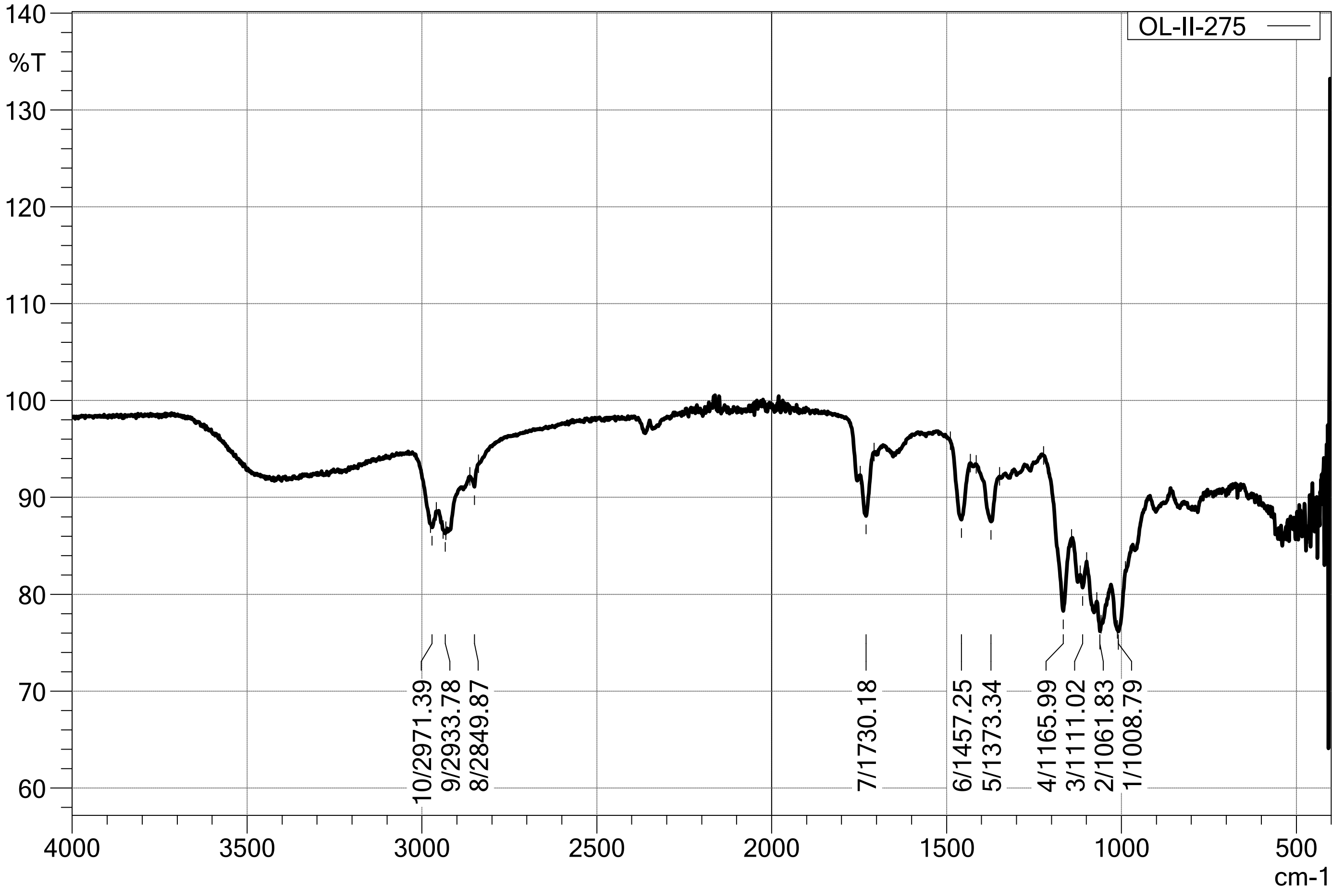


C:¥LabSolutions¥LabSolutionsIR¥Data¥Miller_OliviaL¥OL-II-275.ispd

| | Item | Value |
|---|---|---|
| 2 | Sample name | |
| 3 | Sample ID | |
| 4 | Option | |
| 5 | Intensity Mode | %Transmittance |
| 6 | Apodization | Happ-Genzel |
| 9 | No. of Scans | 32 |
| 10 | Resolution | 2 cm-1 |

| | Peak | Intensity | Corr. Intensity | Base (H) | Base (L) | Area | Corr. Area | Comment |
|---|---|---|---|---|---|---|---|---|
| 1 | 1008.79 | 76.15 | 0.99 | 1011.68 | 988.54 | 500.011 | 23.676 | |
| 2 | 1061.83 | 76.19 | 1.44 | 1070.51 | 1057.97 | 282.420 | 7.560 | |
| 3 | 1111.02 | 80.67 | 1.85 | 1117.77 | 1099.44 | 334.336 | 17.312 | |
| 4 | 1165.99 | 78.27 | 9.98 | 1222.89 | 1142.84 | 1076.726 | 280.784 | |
| 5 | 1373.34 | 87.51 | 5.13 | 1414.81 | 1348.27 | 623.626 | 144.004 | |
| 6 | 1457.25 | 87.69 | 6.86 | 1489.07 | 1432.17 | 495.152 | 193.264 | |
| 7 | 1730.18 | 88.07 | 5.23 | 1746.57 | 1707.03 | 353.589 | 96.603 | |
| 8 | 2849.87 | 91.09 | 1.80 | 2863.38 | 2838.30 | 197.221 | 17.481 | |
| 9 | 2933.78 | 86.26 | 0.32 | 2939.57 | 2931.85 | 104.326 | 1.096 | |
| 10 | 2971.39 | 86.89 | 0.69 | 2975.25 | 2958.85 | 204.762 | 6.749 | |
