# Supplementary material for: Site-Divergent Oxidations within Venerable Macrolide Antibiotic Scaffolds Unveil Compounds with Broad Spectrum and Anti-MRSA Activities
Source: ACS Cent Sci. 2026 Mar 17;12(3):375–82. doi: 10.1021/acscentsci.5c02343 (PMC13022725; doi:10.1021/acscentsci.5c02343)

==== Shimadzu LabSolutions Browser Report ====

PDA Chromatogram(OL-II-275.lcd)

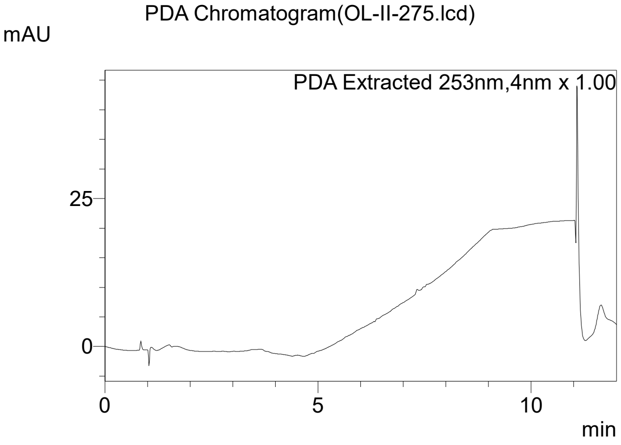

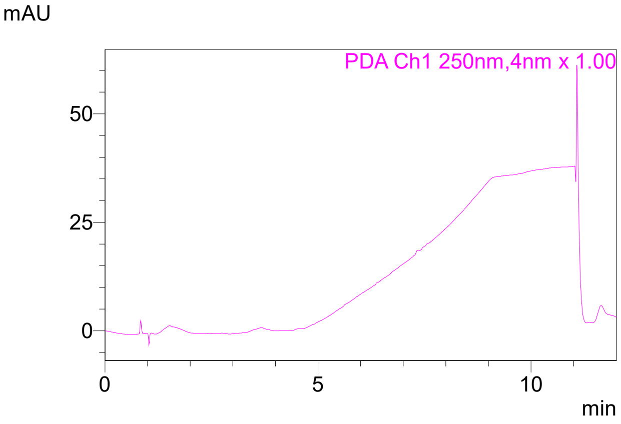

MS Spectrum(OL-II-275.lcd)

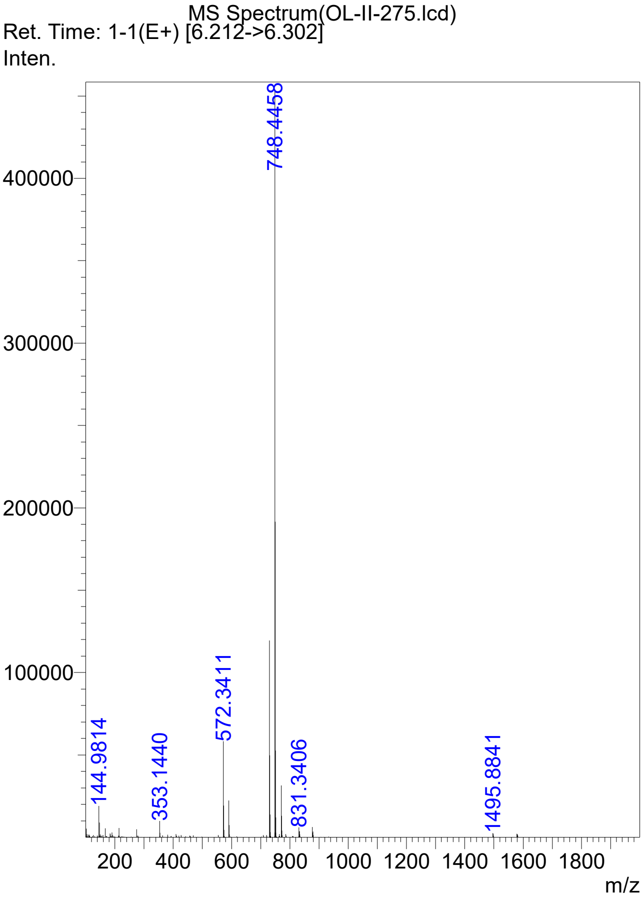

MS Chromatogram(OL-II-275.lcd)

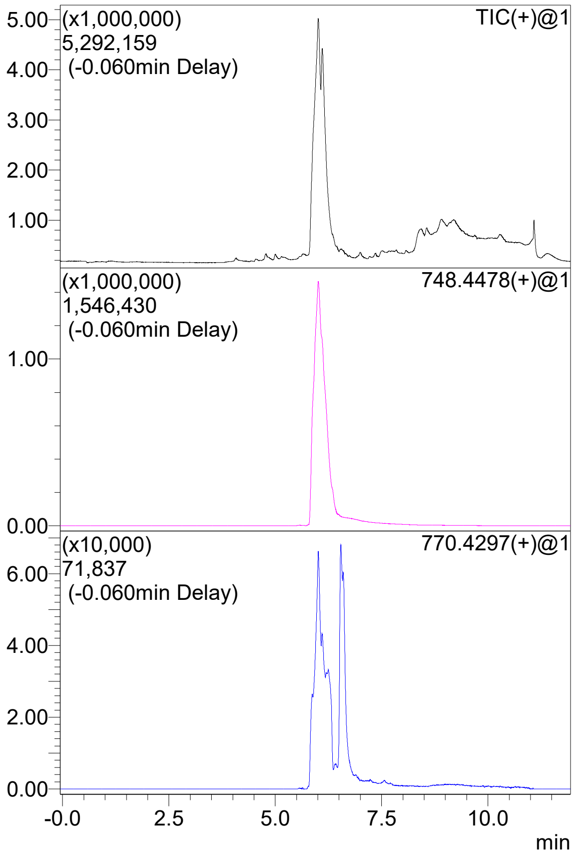

MS Spectrum(OL-II-275.lcd)

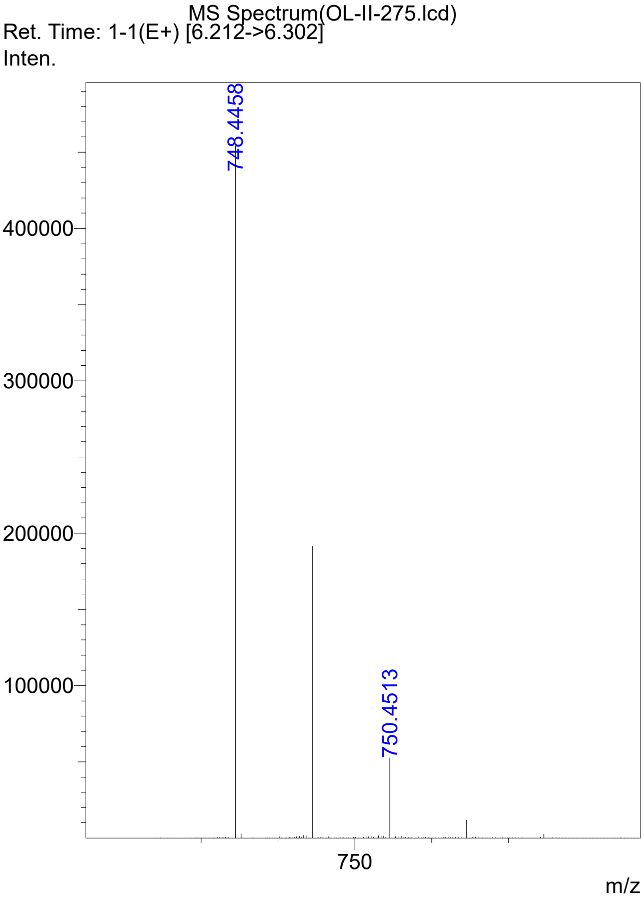

Supplement: Supplementary file 2 [file oc5c02343_si_002.zip › Erythromycin Analog Characterization 2,5',11,12/5'/HRMS/OL-II-275.pdf]
