# Supplementary material for: Site-Divergent Oxidations within Venerable Macrolide Antibiotic Scaffolds Unveil Compounds with Broad Spectrum and Anti-MRSA Activities
Source: ACS Cent Sci. 2026 Mar 17;12(3):375–82. doi: 10.1021/acscentsci.5c02343 (PMC13022725; doi:10.1021/acscentsci.5c02343)
Supplement: Supplementary file 2 [file oc5c02343_si_002.zip › Erythromycin Analog Characterization 2,5',11,12/12/IR/OL-III-041.pdf]

SHIMADZU

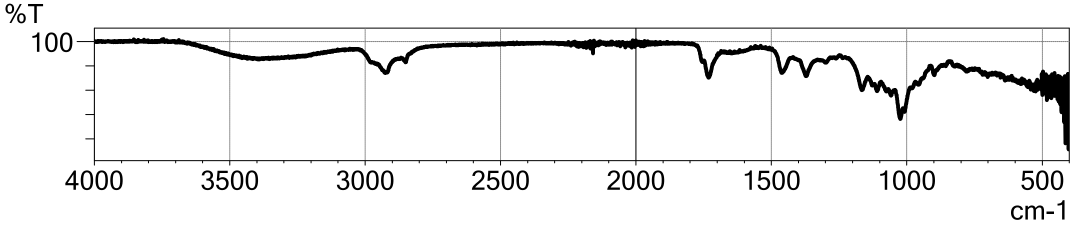

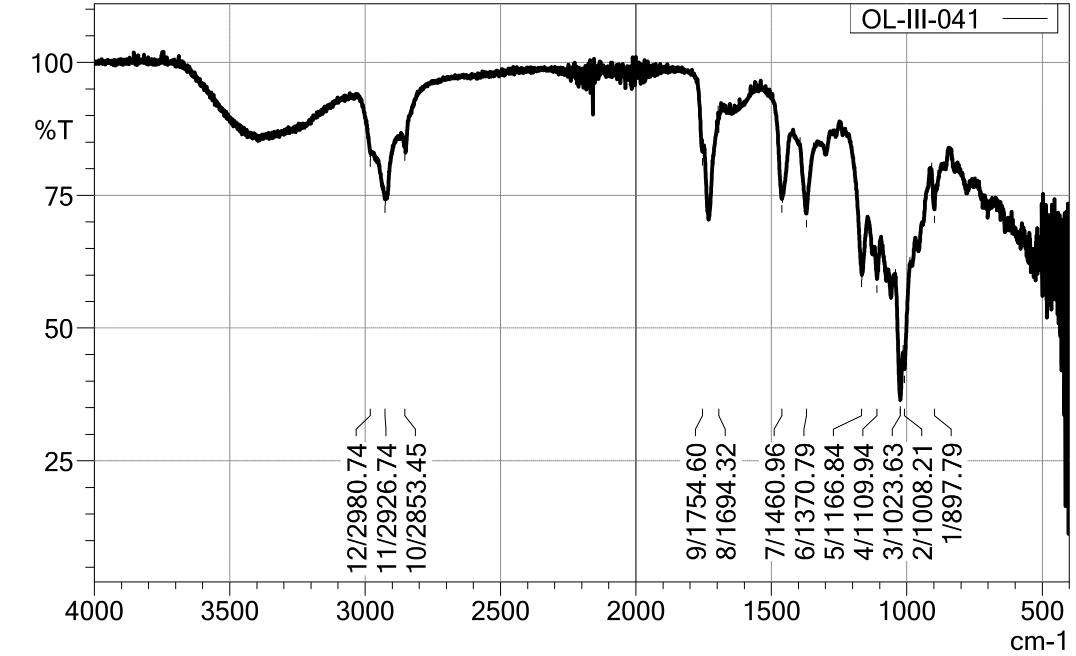

	Peak	Intensity	Corr. Intensity	Base (H)	Base (L)	Area	Corr. Area	Comment
1	897.79	72.36	4.50	908.40	893.93	362.589	41.472	
2	1008.21	42.26	6.69	1013.51	988.44	1222.313	63.117	
3	1023.63	36.44	12.17	1041.47	1016.40	1375.374	170.026	
4	1109.94	59.27	1.60	1112.83	1108.01	193.005	4.641	
5	1166.84	60.26	0.37	1169.25	1166.36	112.808	0.370	
6	1370.79	71.58	12.55	1394.42	1344.27	1101.868	304.332	
7	1460.96	74.31	2.18	1482.17	1458.55	447.517	35.397	
8	1694.32	89.35	1.17	1695.77	1689.50	63.361	3.859	
9	1754.60	83.21	3.04	1769.54	1751.22	222.770	33.443	
10	2853.45	84.09	0.31	2854.89	2852.97	30.383	0.374	
11	2926.74	74.29	0.37	2929.63	2925.77	97.830	1.130	
12	2980.74	82.93	0.21	2981.70	2980.26	24.378	0.137	

C:¥LabSolutions¥LabSolutionsIR¥Data
¥Miller_OliviaL¥OL-III-041.ispd

	Item	Value
2	Sample name	
3	Sample ID	
4	Option	
5	Intensity Mode	%Transmittance
6	Apodization	Happ-Genzel
9	No. of Scans	16
10	Resolution	1 cm-1
