# Supplementary material for: Site-Divergent Oxidations within Venerable Macrolide Antibiotic Scaffolds Unveil Compounds with Broad Spectrum and Anti-MRSA Activities
Source: ACS Cent Sci. 2026 Mar 17;12(3):375–82. doi: 10.1021/acscentsci.5c02343 (PMC13022725; doi:10.1021/acscentsci.5c02343)
Supplement: Supplementary file 2 [file oc5c02343_si_002.zip › Erythromycin Analog Characterization 2,5',11,12/2/IR/OL-III-023.pdf]

SHIMADZU

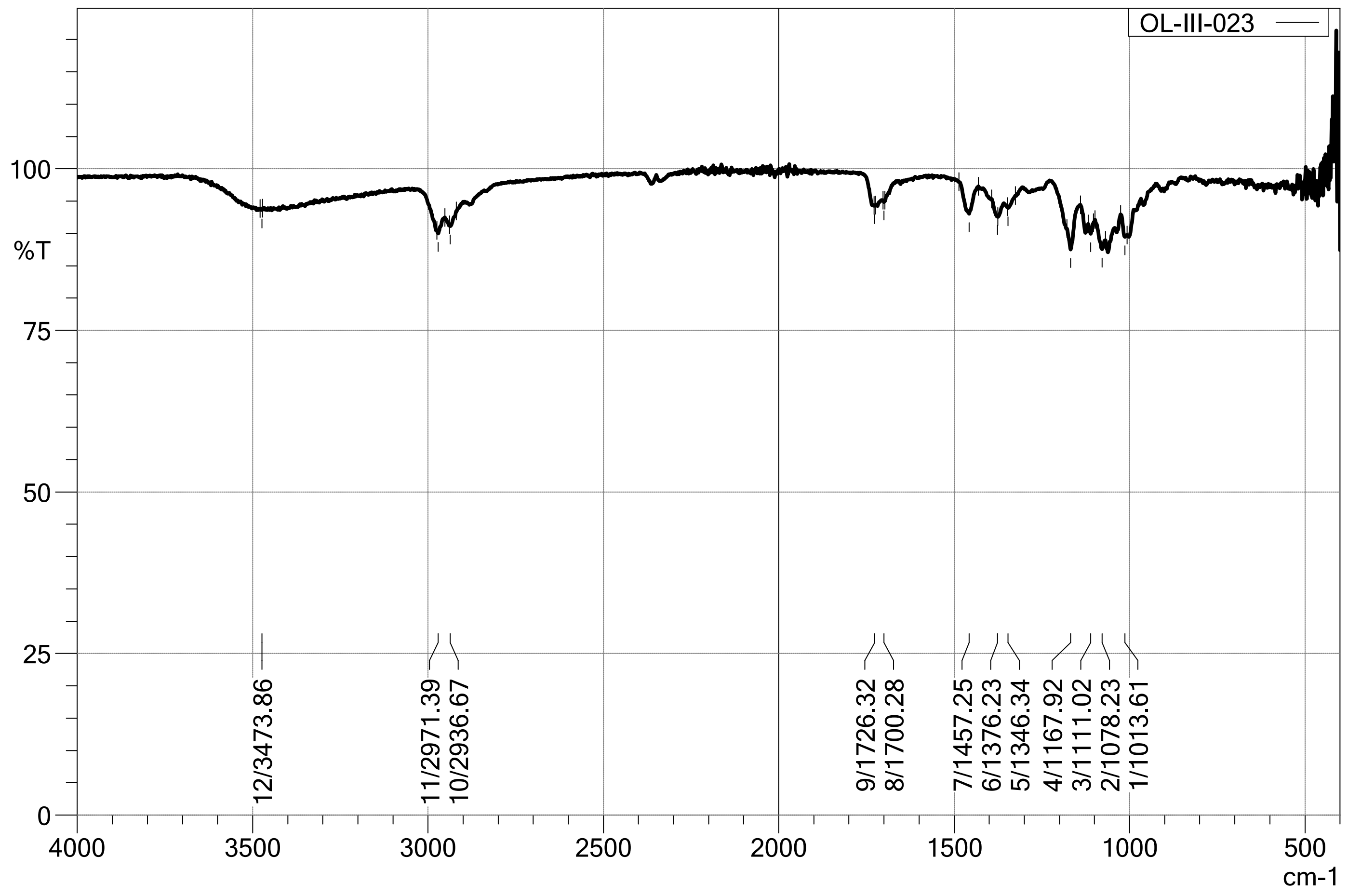

C:¥LabSolutions¥LabSolutionsIR¥Data¥Miller_OliviaL¥OL-III-023.ispd

	Item	Value
2	Sample name	
3	Sample ID	
4	Option	
5	Intensity Mode	%Transmittance
6	Apodization	Happ-Genzel
9	No. of Scans	32
10	Resolution	2 cm-1

	Peak	Intensity	Corr. Intensity	Base (H)	Base (L)	Area	Corr. Area	Comment
1	1013.61	89.46	1.47	1025.18	1006.86	170.773	12.234	
2	1078.23	87.58	2.39	1098.48	1068.58	317.832	34.836	
3	1111.02	89.92	1.64	1117.77	1103.30	134.528	12.478	
4	1167.92	87.51	4.27	1178.53	1139.95	336.821	51.838	
5	1346.34	93.95	0.32	1348.27	1325.12	119.953	4.012	
6	1376.23	92.60	0.15	1393.59	1375.27	115.317	4.839	
7	1457.25	93.07	4.56	1486.18	1431.21	251.216	122.040	
8	1700.28	94.87	0.28	1703.17	1697.39	28.738	0.698	
9	1726.32	94.30	0.06	1728.25	1724.39	21.880	0.115	
10	2936.67	91.14	0.41	2938.60	2919.31	152.486	5.773	
11	2971.39	90.00	0.73	2974.29	2952.10	193.060	4.037	
12	3473.86	93.61	0.27	3478.68	3471.93	42.433	1.040	
