# Supplementary figures and images for: Site-Divergent Oxidations within Venerable Macrolide Antibiotic Scaffolds Unveil Compounds with Broad Spectrum and Anti-MRSA Activities
Source: ACS Cent Sci. 2026 Mar 17;12(3):375–82. doi: 10.1021/acscentsci.5c02343 (PMC13022725; doi:10.1021/acscentsci.5c02343)

==== Shimadzu LabSolutions Browser Report ====

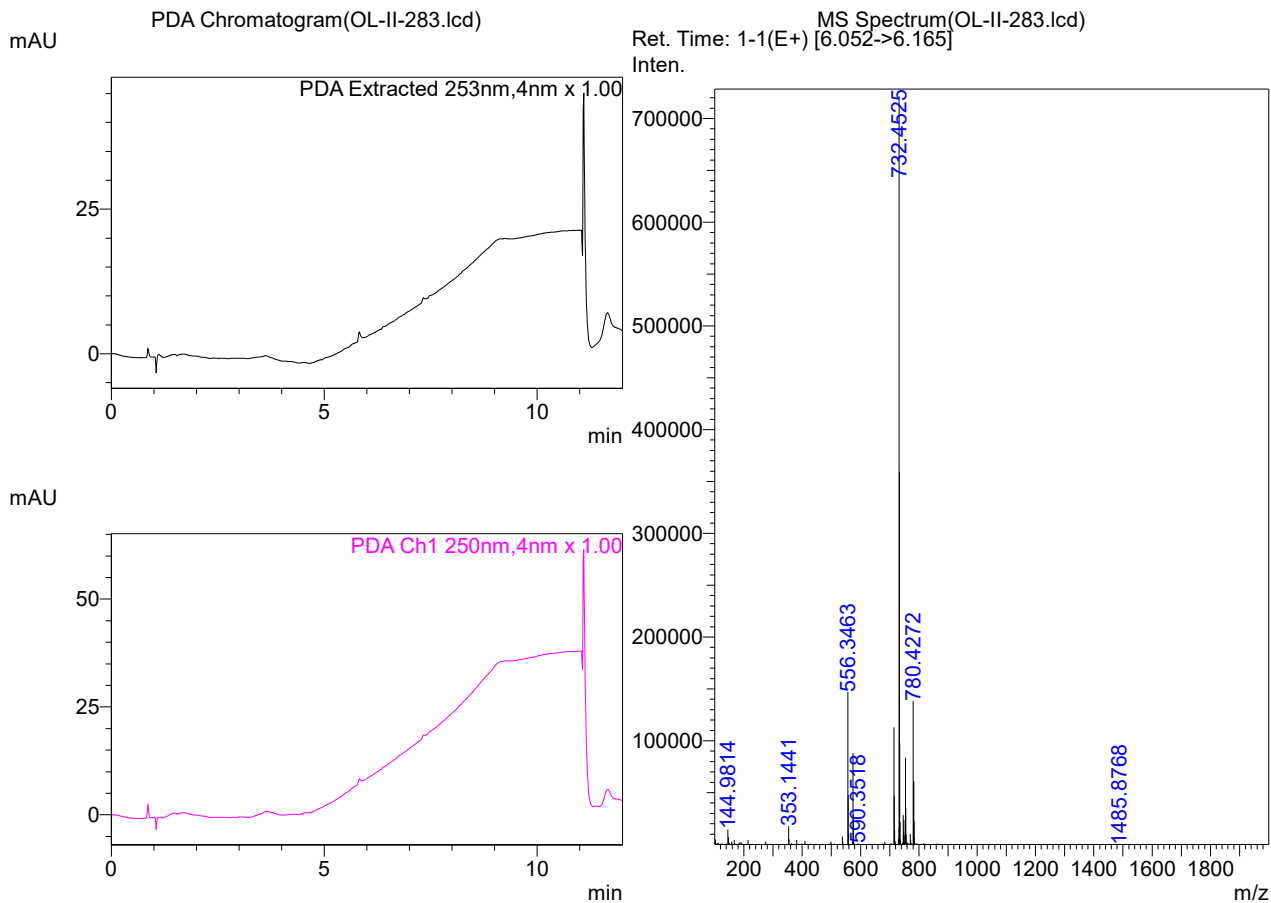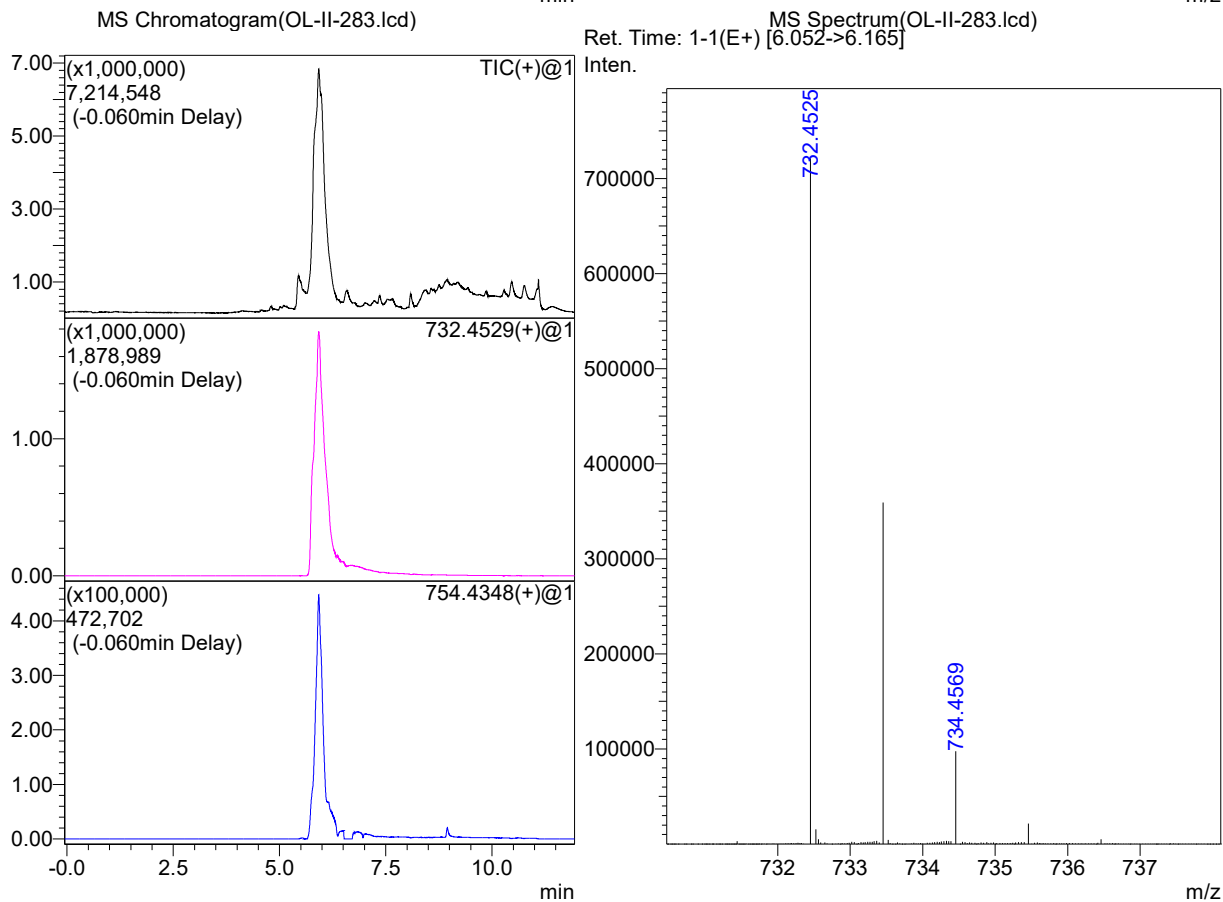

Supplement: Supplementary file 2 [file oc5c02343_si_002.zip › Erythromycin Analog Characterization 2,5',11,12/11/HRMS/OL-II-283.pdf]

# ==== Shimadzu LabSolutions Browser Report ====

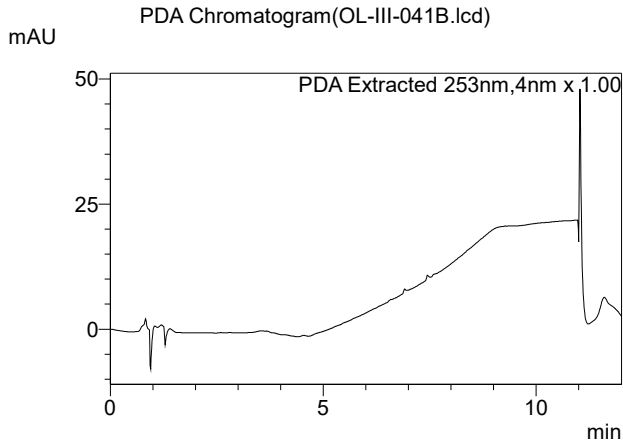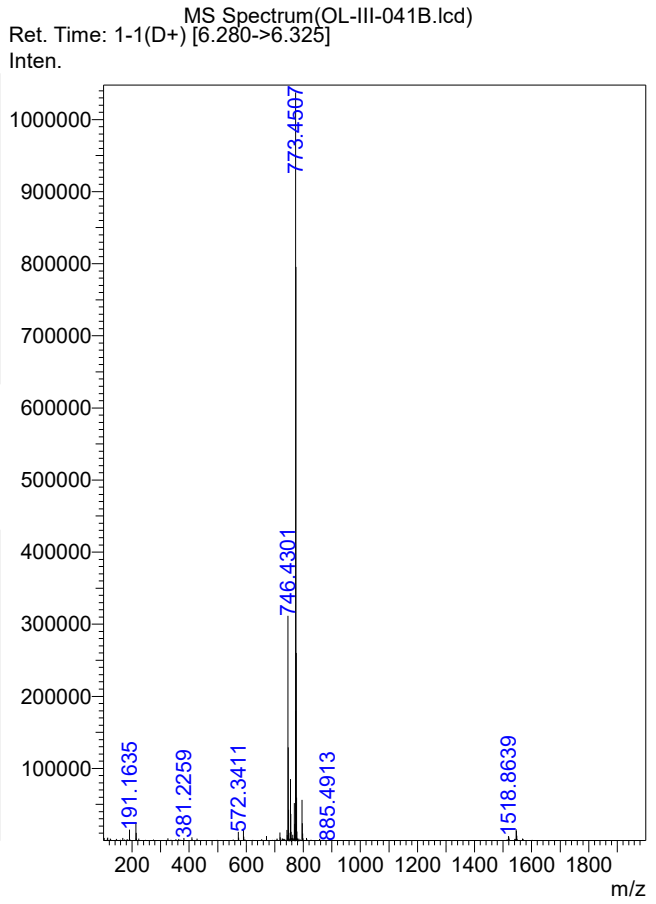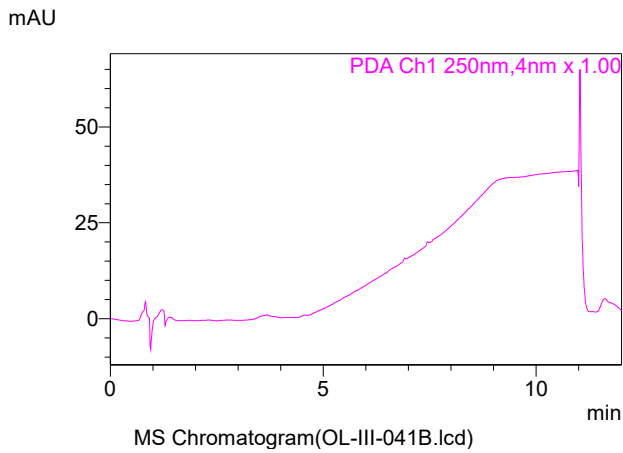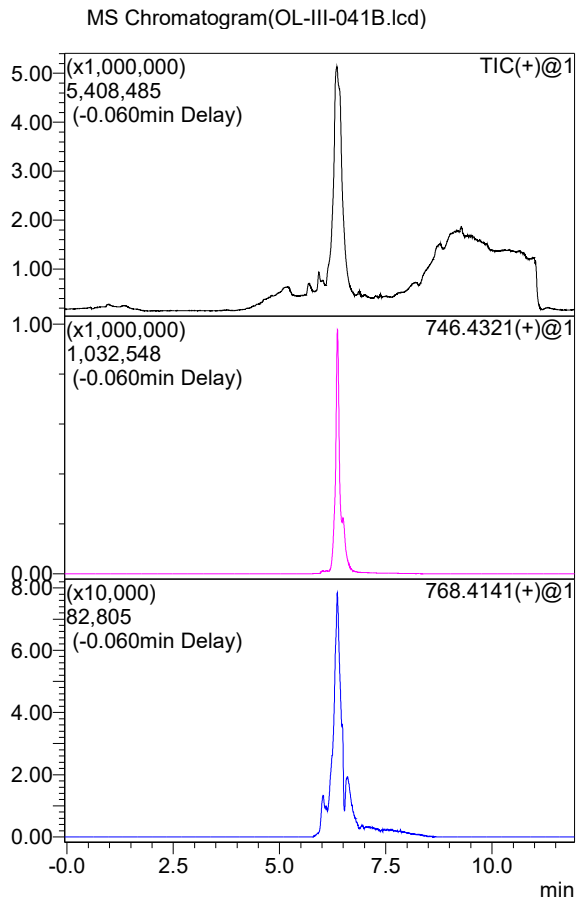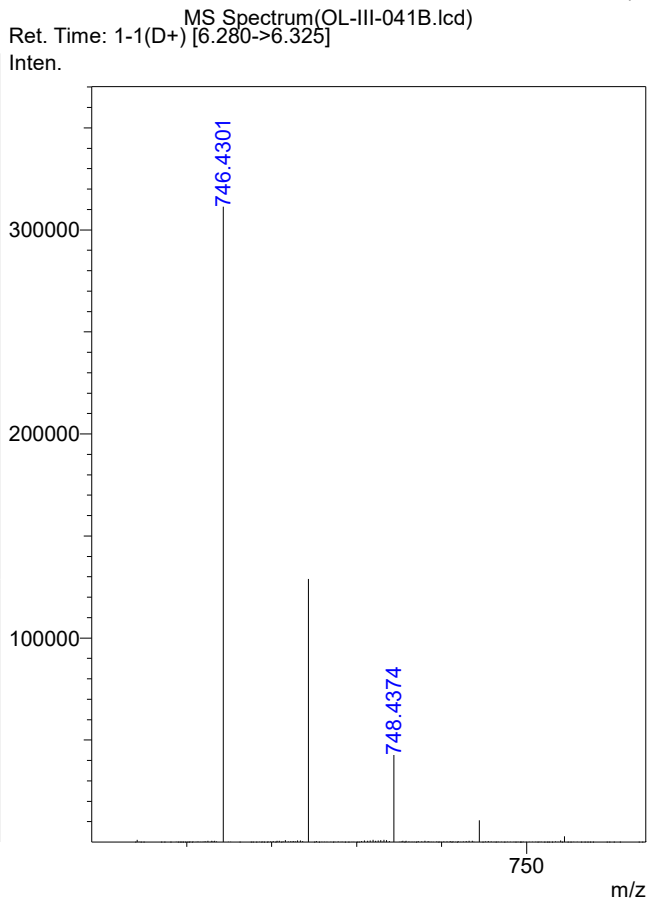

Supplement: Supplementary file 2 [file oc5c02343_si_002.zip › Erythromycin Analog Characterization 2,5',11,12/12/HRMS/OL-III-041B.pdf]

# ==== Shimadzu LabSolutions Browser Report ====

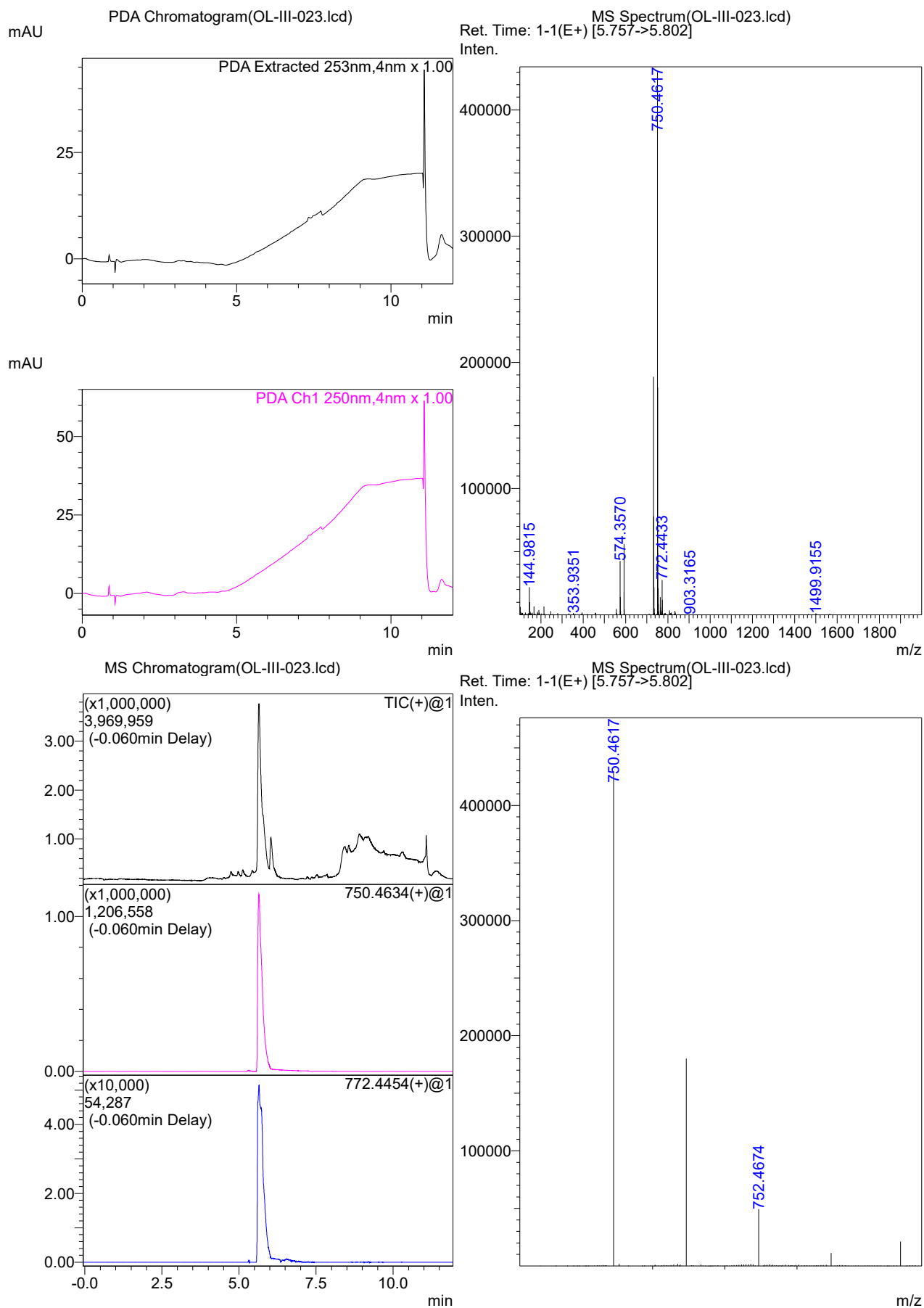

Supplement: Supplementary file 2 [file oc5c02343_si_002.zip › Erythromycin Analog Characterization 2,5',11,12/2/HRMS/OL-III-023.pdf]

# ==== Shimadzu LabSolutions Browser Report ====

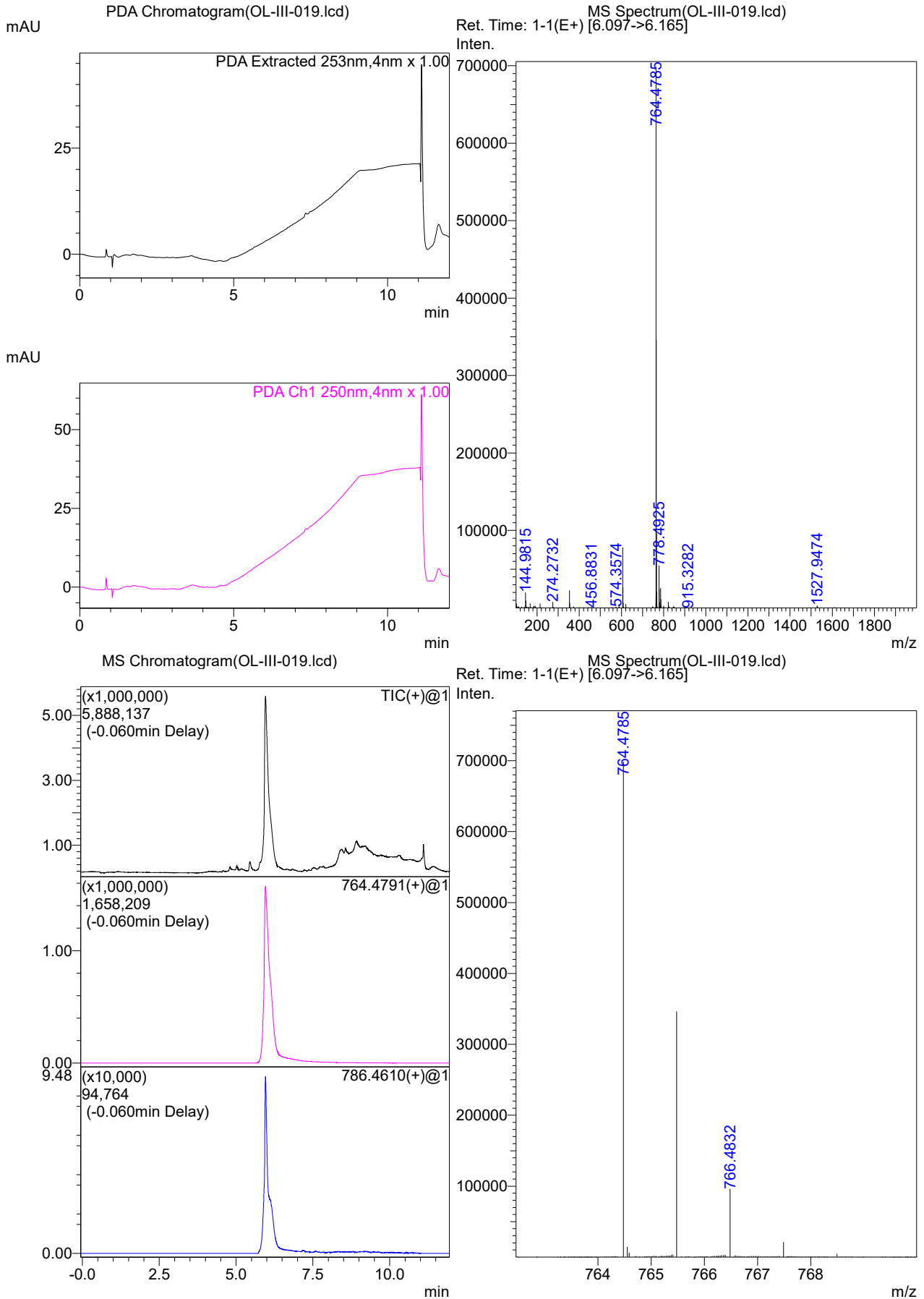

Supplement: Supplementary file 4 [file oc5c02343_si_004.zip › Clarithromycin and Azithromycin Analog Characterization/7/HRMS/OL-III-019.pdf]

# ==== Shimadzu LabSolutions Browser Report ====

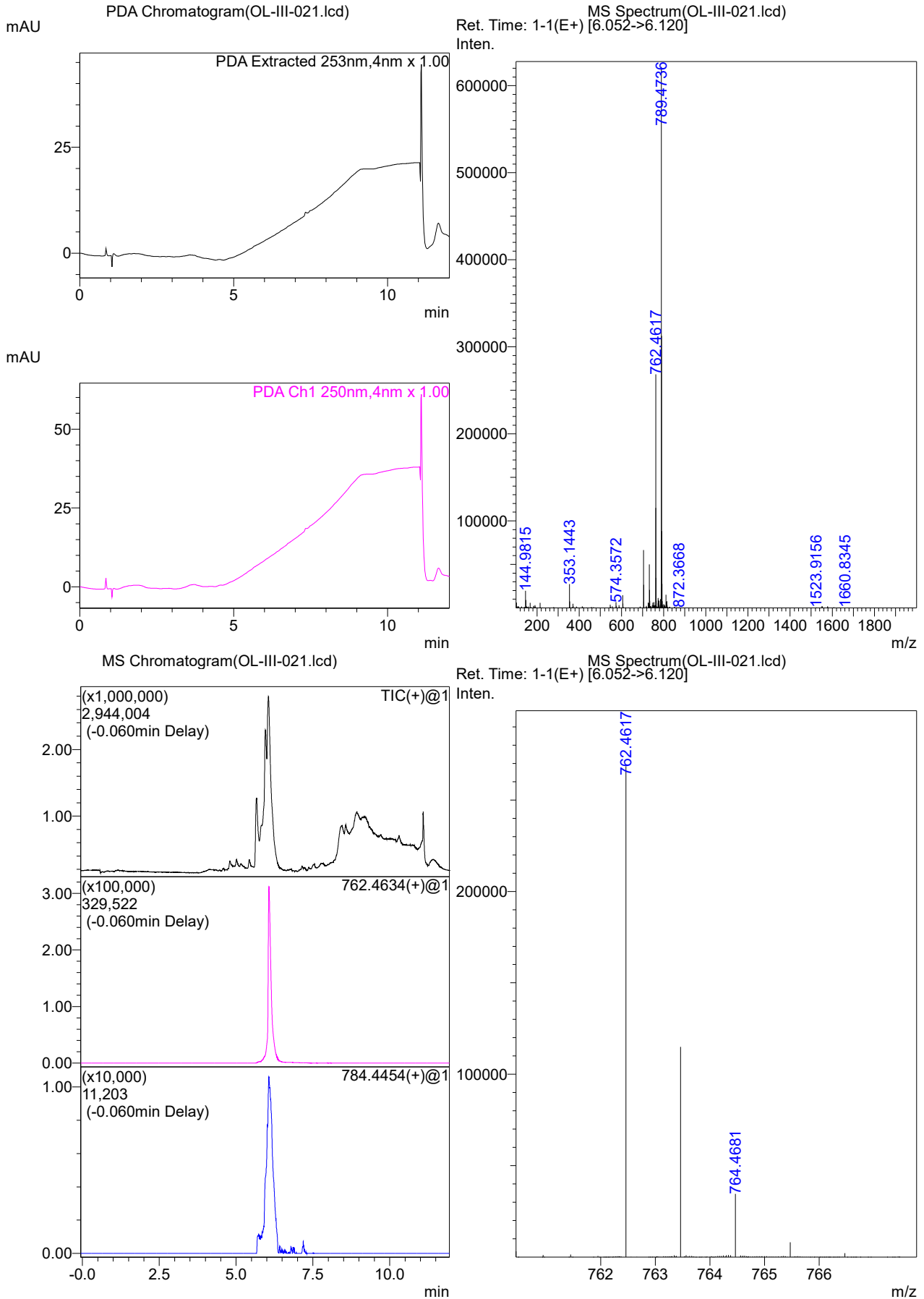

Supplement: Supplementary file 4 [file oc5c02343_si_004.zip › Clarithromycin and Azithromycin Analog Characterization/8/HRMS/OL-III-021.pdf]

# ==== Shimadzu LabSolutions Browser Report ====

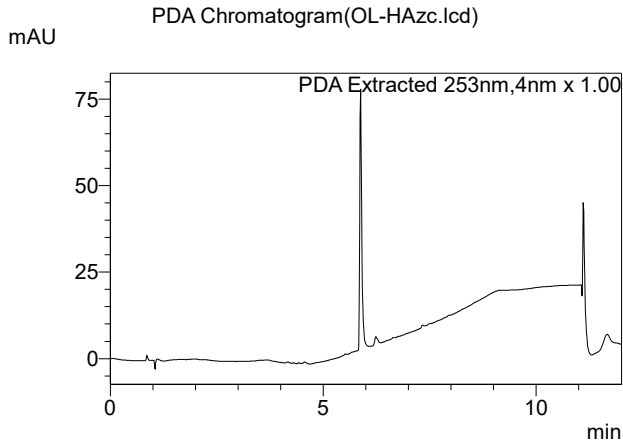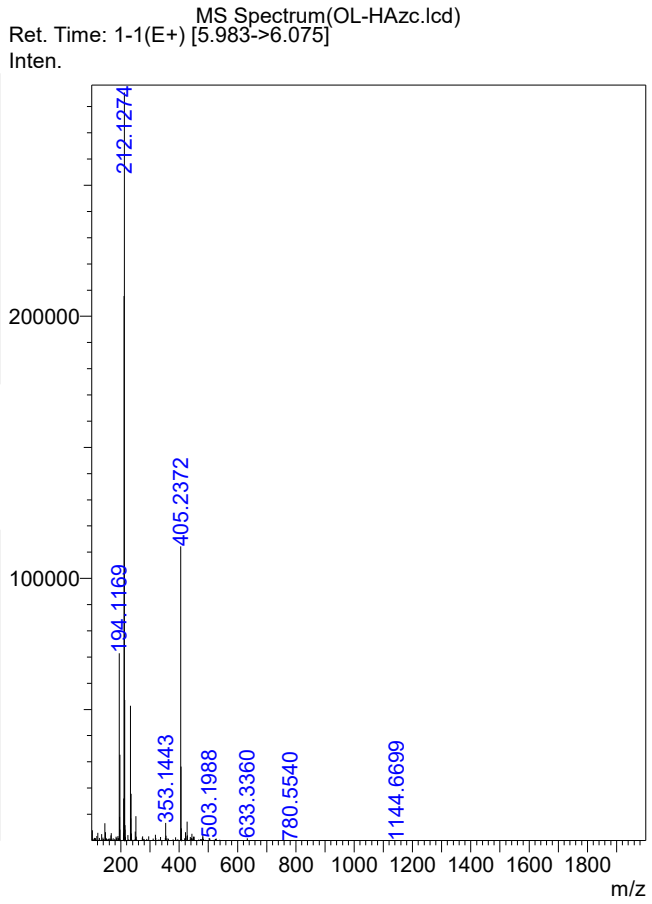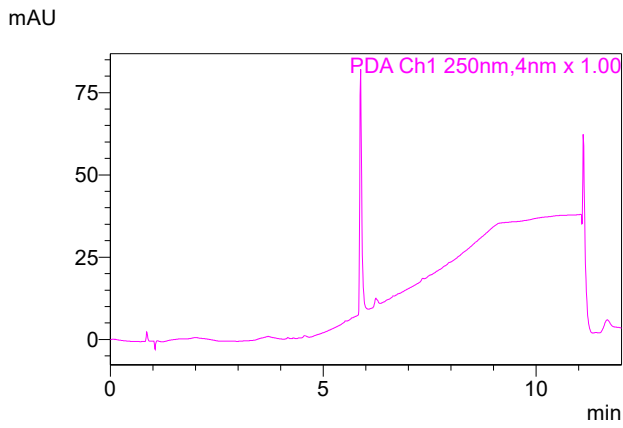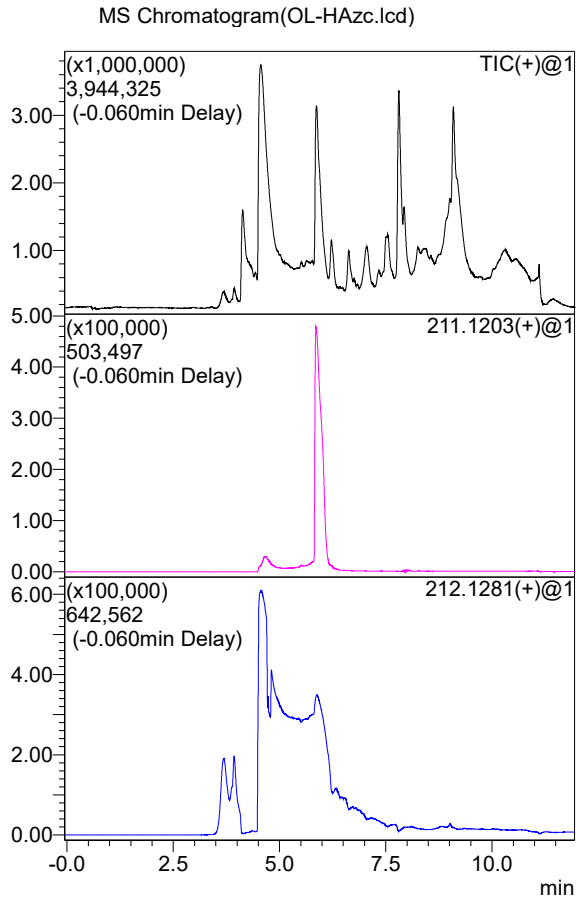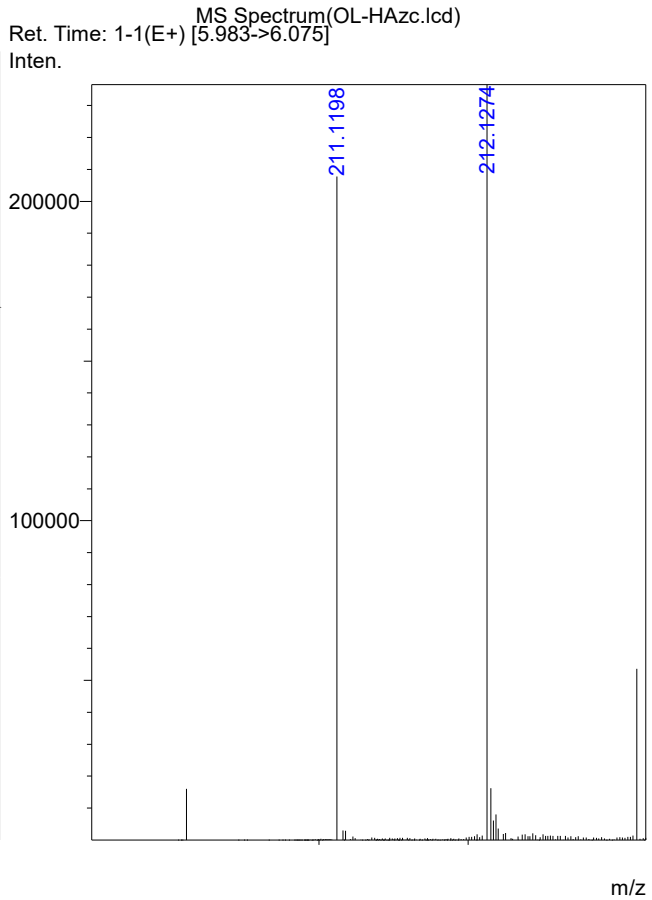

Supplement: Supplementary file 6 [file oc5c02343_si_006.zip › Catalyst and SI Compound Characterization/HAzc-OMe/HRMS/OL-HAzc_rad+H+.pdf]

# ==== Shimadzu LabSolutions Browser Report ====

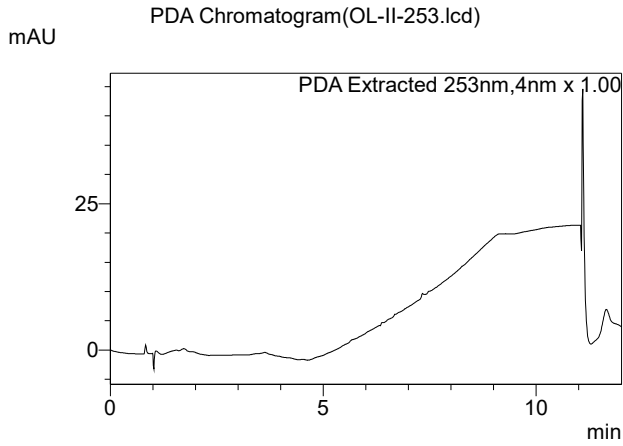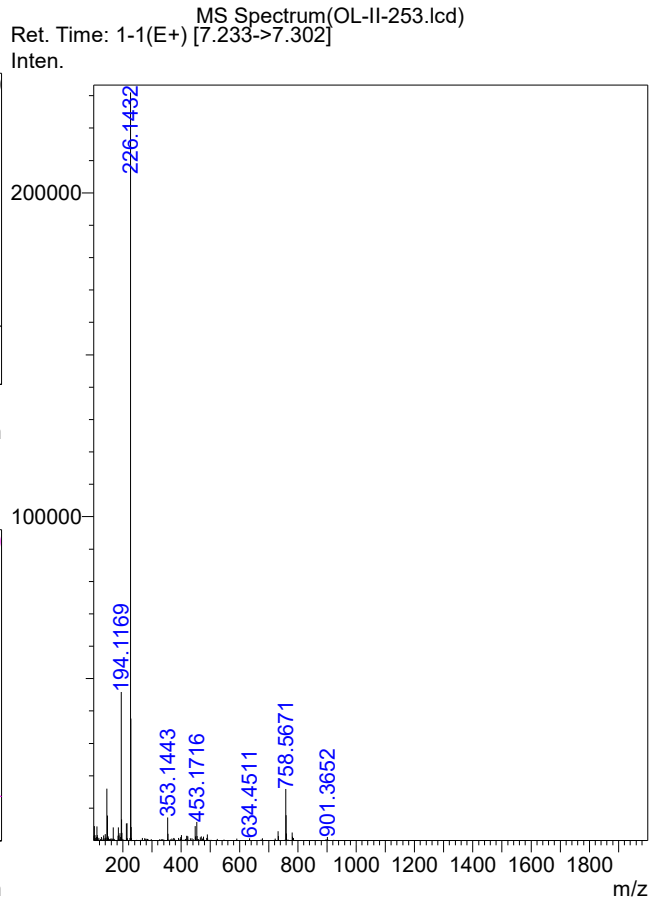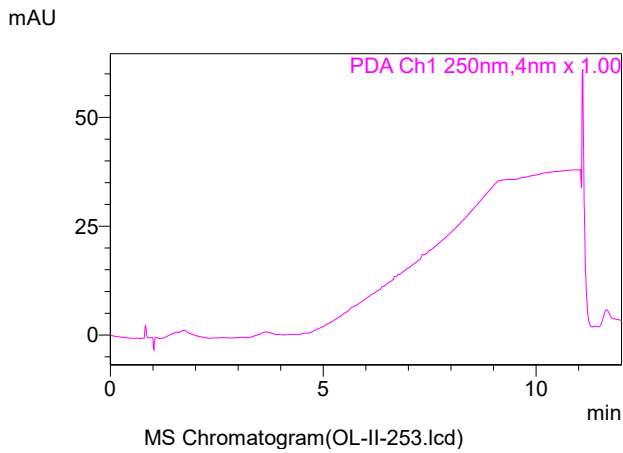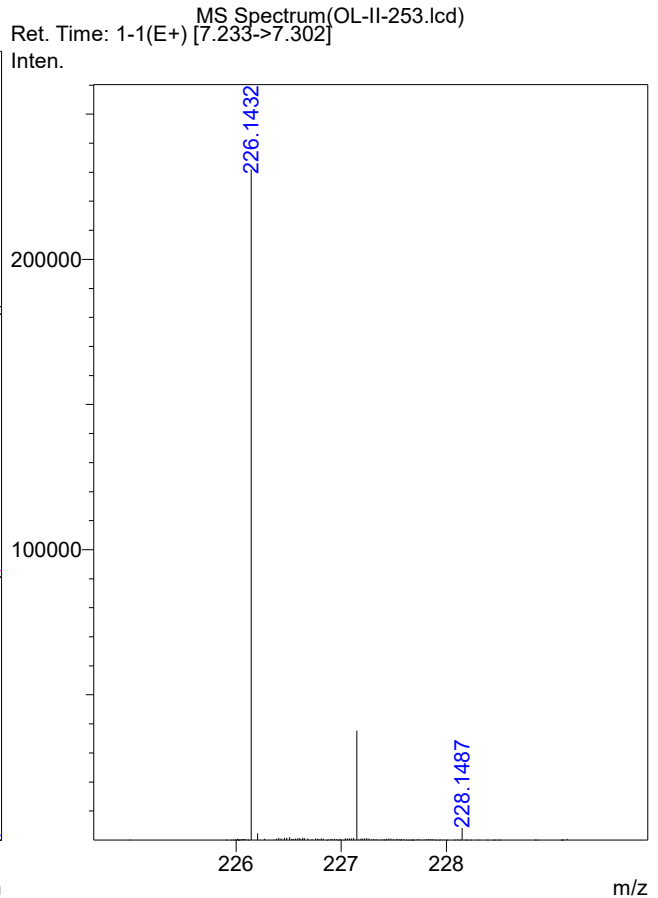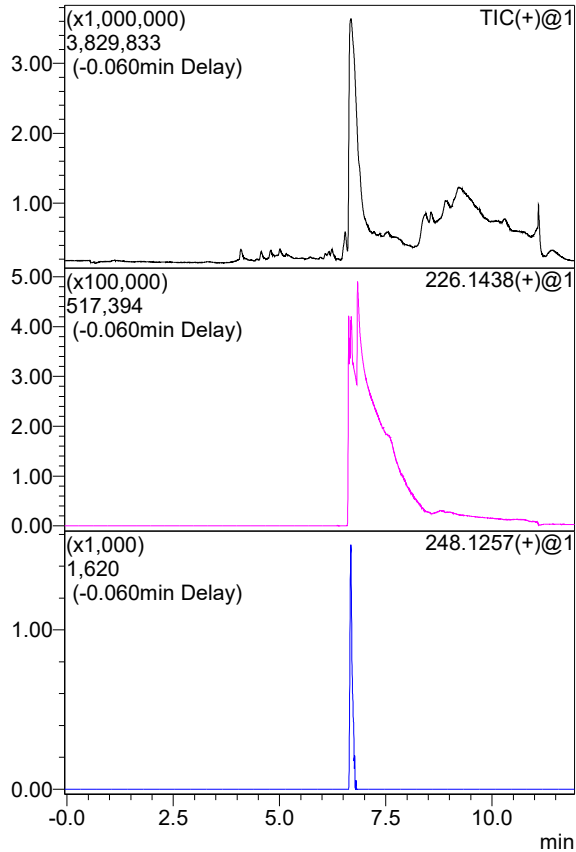

Supplement: Supplementary file 6 [file oc5c02343_si_006.zip › Catalyst and SI Compound Characterization/HAzc(OMe)-OMe/HRMS/OL-II-253.pdf]
