# Supplementary material for: Site-Divergent Oxidations within Venerable Macrolide Antibiotic Scaffolds Unveil Compounds with Broad Spectrum and Anti-MRSA Activities
Source: ACS Cent Sci. 2026 Mar 17;12(3):375–82. doi: 10.1021/acscentsci.5c02343 (PMC13022725; doi:10.1021/acscentsci.5c02343)
Supplement: Supplementary file 3 [file oc5c02343_si_003.zip › Erythromycin Analog Characterization 13,14,15/15/IR/OL-III-050.pdf]

SHIMADZU

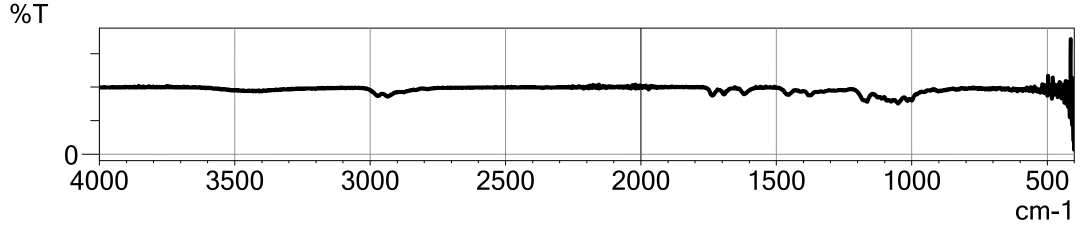

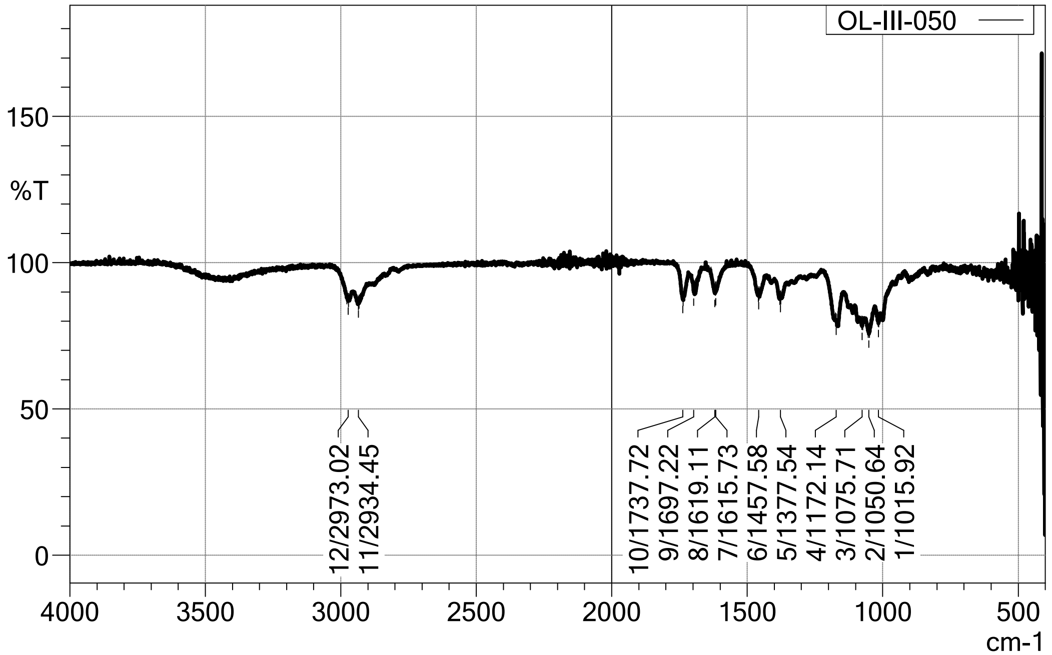

	Peak	Intensity	Corr. Intensity	Base (H)	Base (L)	Area	Corr. Area	Comment
1	1015.92	79.22	1.24	1016.88	1014.47	48.489	1.317	
2	1050.64	75.59	1.12	1053.53	1049.19	103.439	2.315	
3	1075.71	78.19	1.18	1077.15	1074.26	61.522	1.827	
4	1172.14	80.03	0.26	1174.07	1171.66	47.449	0.183	
5	1377.54	87.71	0.34	1379.47	1375.61	46.759	0.690	
6	1457.58	88.72	0.54	1458.55	1456.62	21.071	0.351	
7	1615.73	90.01	1.30	1616.70	1607.54	81.279	8.467	
8	1619.11	89.47	2.37	1633.57	1616.70	130.478	23.061	
9	1697.22	90.00	1.76	1700.59	1696.25	37.868	5.025	
10	1737.72	87.42	0.70	1751.70	1736.76	121.973	7.016	
11	2934.45	85.88	0.48	2935.42	2932.52	39.928	0.689	
12	2973.02	86.84	0.56	2977.36	2972.06	66.744	1.522	

C:¥LabSolutions¥LabSolutionsIR¥Data
¥Miller_OliviaL¥OL-III-050.ispd

	Item	Value
2	Sample name	
3	Sample ID	
4	Option	
5	Intensity Mode	%Transmittance
6	Apodization	Happ-Genzel
9	No. of Scans	16
10	Resolution	1 cm-1
