# Supplementary material for: Site-Divergent Oxidations within Venerable Macrolide Antibiotic Scaffolds Unveil Compounds with Broad Spectrum and Anti-MRSA Activities
Source: ACS Cent Sci. 2026 Mar 17;12(3):375–82. doi: 10.1021/acscentsci.5c02343 (PMC13022725; doi:10.1021/acscentsci.5c02343)

C:\Xcalibur\data\OL-111-116_pos_v1 09/25/2[illegible].38

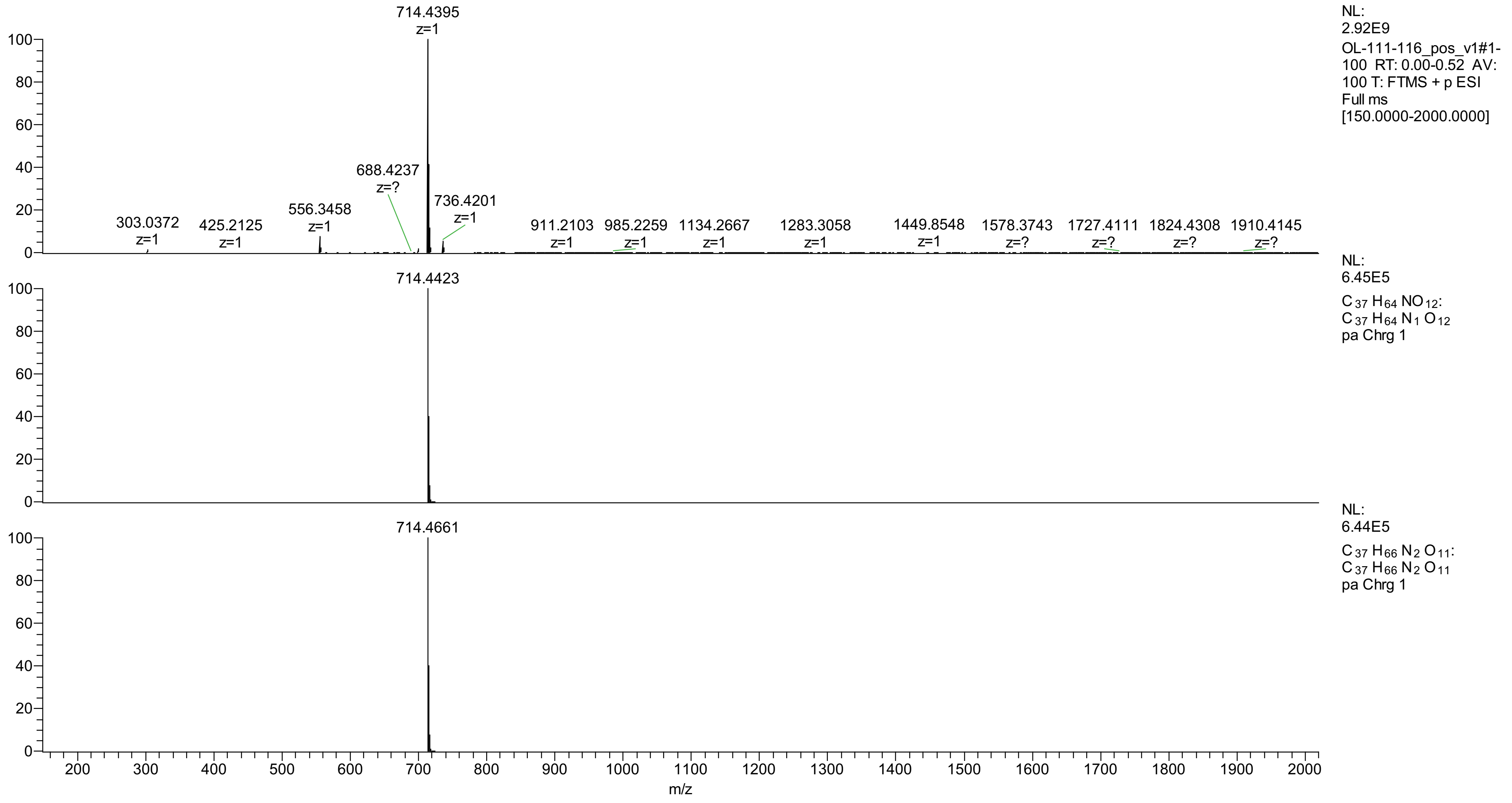

C:\Xcalibur\data\OL-111-116_pos_v1 09/25/2[illegible].38

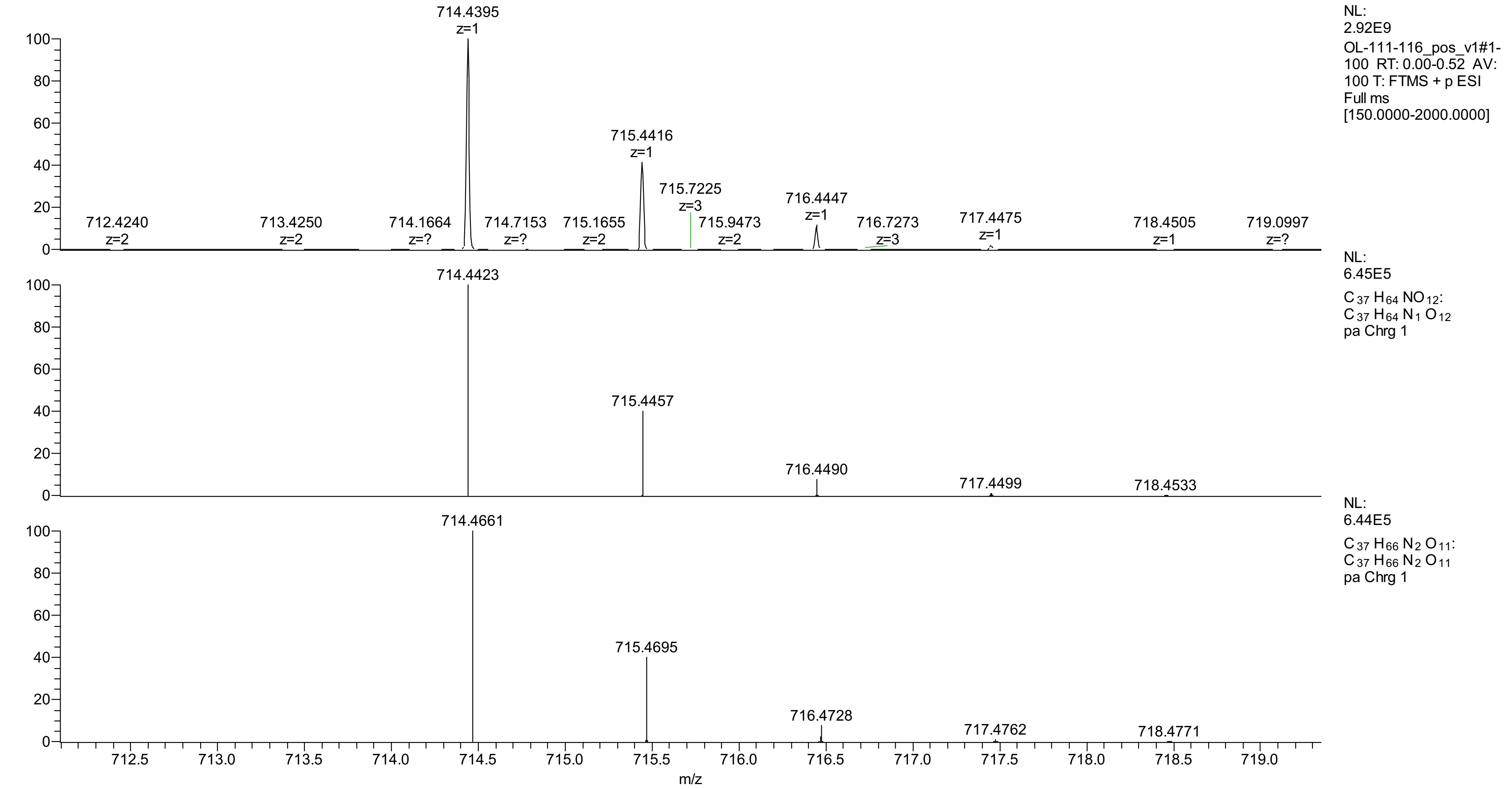

Supplement: Supplementary file 3 [file oc5c02343_si_003.zip › Erythromycin Analog Characterization 13,14,15/15/HRMS/OL-III-116-714.RAW.pdf]
