# Supplementary material for: Site-Divergent Oxidations within Venerable Macrolide Antibiotic Scaffolds Unveil Compounds with Broad Spectrum and Anti-MRSA Activities
Source: ACS Cent Sci. 2026 Mar 17;12(3):375–82. doi: 10.1021/acscentsci.5c02343 (PMC13022725; doi:10.1021/acscentsci.5c02343)
Supplement: Supplementary file 3 [file oc5c02343_si_003.zip › Erythromycin Analog Characterization 13,14,15/13/IR/OL-III-054.pdf]

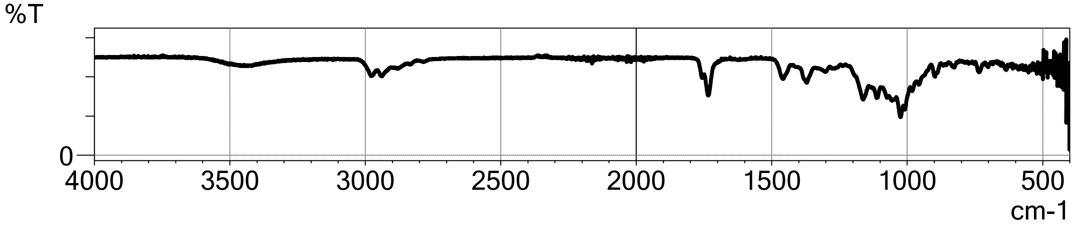

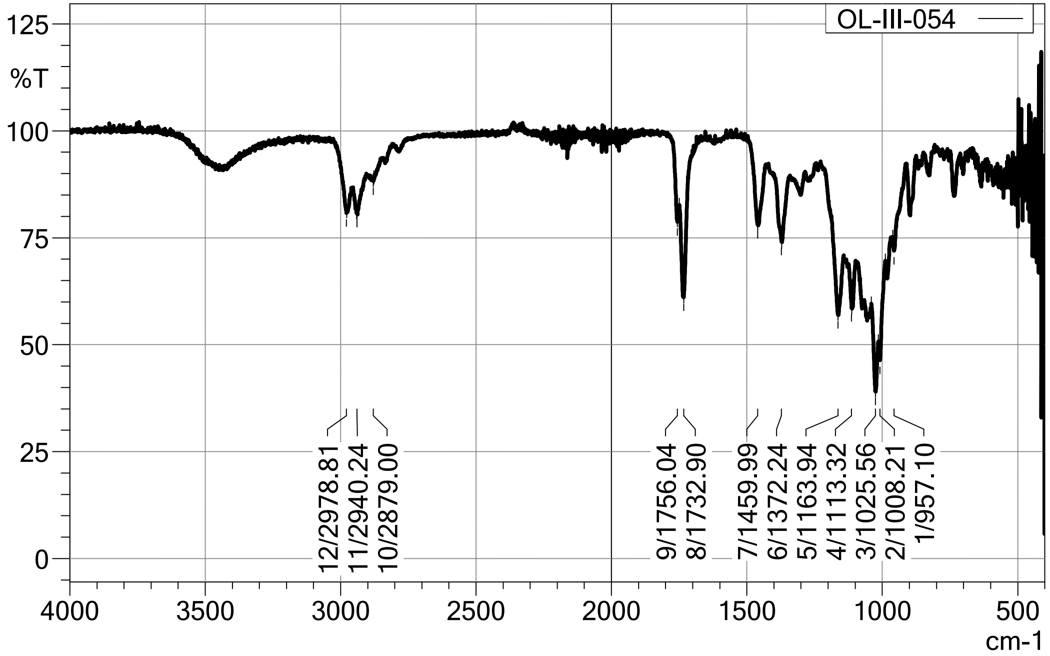

	Peak	Intensity	Corr. Intensity	Base (H)	Base (L)	Area	Corr. Area	Comment
1	957.10	71.99	4.18	961.92	949.38	328.935	34.035	
2	1008.21	46.35	9.29	1014.96	988.92	1126.596	89.917	
3	1025.56	39.07	0.88	1040.03	1025.08	752.945	-2.760	
4	1113.32	58.67	0.54	1119.10	1112.83	241.609	2.799	
5	1163.94	56.98	1.21	1187.57	1162.98	785.818	15.418	
6	1372.24	74.12	0.45	1379.47	1371.27	196.919	-0.901	
7	1459.99	78.04	0.42	1477.83	1459.51	283.604	13.793	
8	1732.90	61.13	1.07	1733.38	1717.47	414.324	21.057	
9	1756.04	78.70	0.89	1757.01	1749.29	149.437	2.269	
10	2879.00	88.24	0.42	2879.48	2877.07	27.581	0.634	
11	2940.24	80.66	0.25	2941.20	2939.27	36.960	0.145	
12	2978.81	80.82	0.90	2991.83	2977.85	222.587	8.349	

C:¥LabSolutions¥LabSolutionsIR¥Data
¥Miller_OliviaL¥OL-III-054.ispd

	Item	Value
2	Sample name	
3	Sample ID	
4	Option	
5	Intensity Mode	%Transmittance
6	Apodization	Happ-Genzel
9	No. of Scans	16
10	Resolution	1 cm-1
