# Supplementary material for: Site-Divergent Oxidations within Venerable Macrolide Antibiotic Scaffolds Unveil Compounds with Broad Spectrum and Anti-MRSA Activities
Source: ACS Cent Sci. 2026 Mar 17;12(3):375–82. doi: 10.1021/acscentsci.5c02343 (PMC13022725; doi:10.1021/acscentsci.5c02343)

==== Shimadzu LabSolutions Browser Report ====

PDA Chromatogram(OL-III-054.lcd)

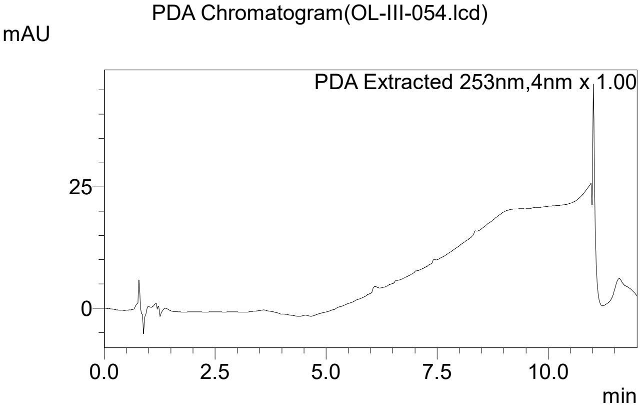

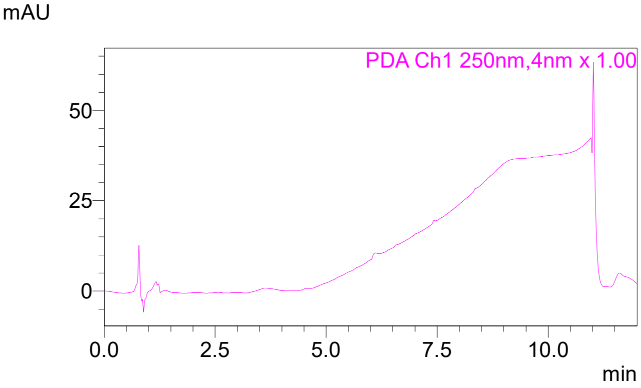

MS Chromatogram(OL-III-054.lcd)

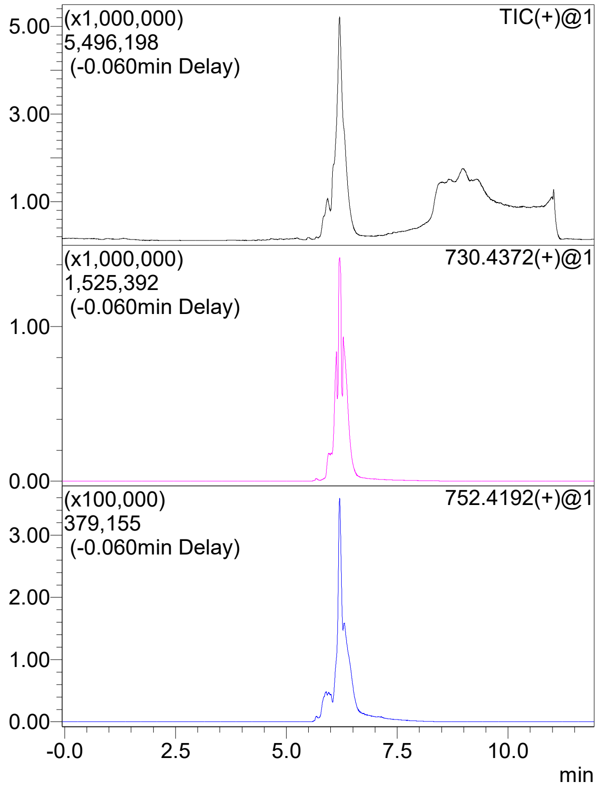

MS Spectrum(OL-III-054.lcd)

Ret. Time: 1-1(D+) [6.347->6.415]

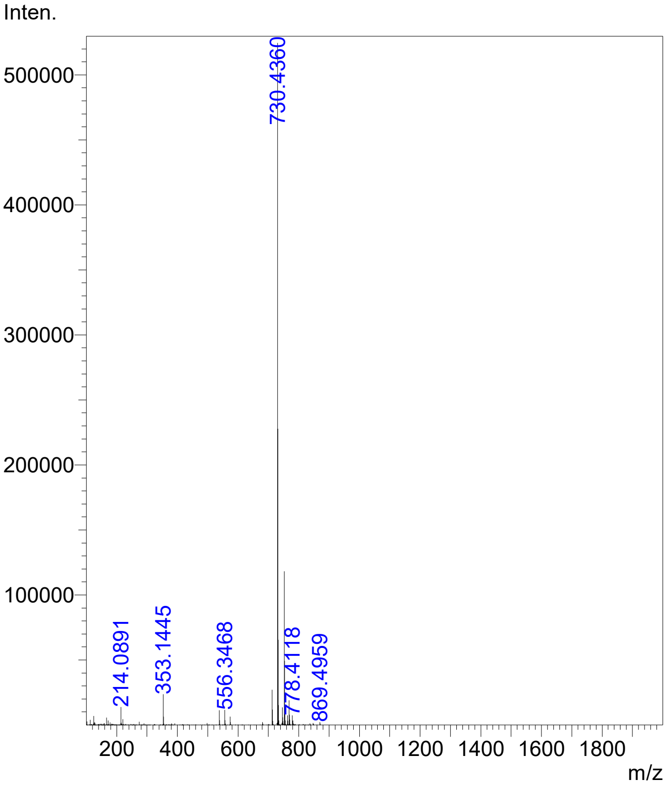

MS Spectrum(OL-III-054.lcd)

Ret. Time: 1-1(D+) [6.347->6.415]

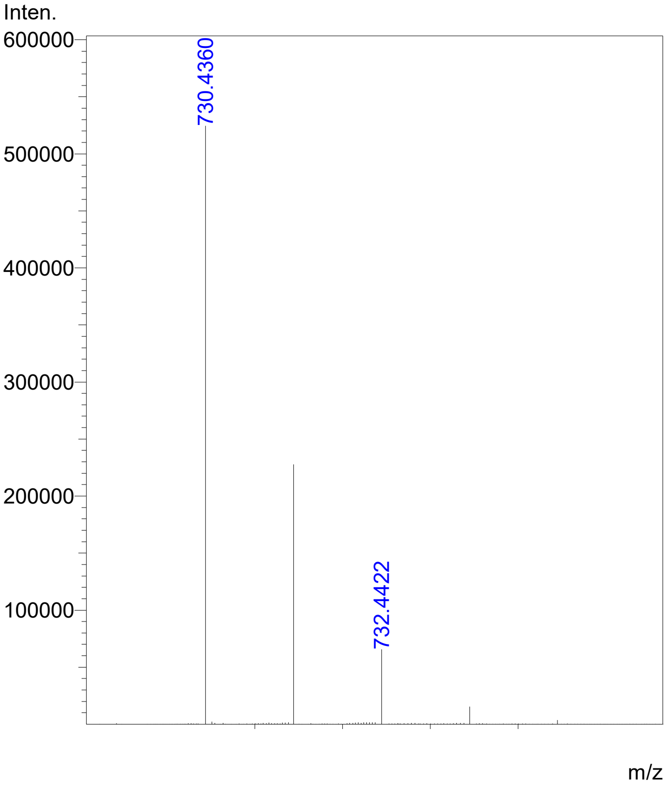

Supplement: Supplementary file 3 [file oc5c02343_si_003.zip › Erythromycin Analog Characterization 13,14,15/13/HRMS/OL-III-054.pdf]
