# Supplementary material for: Site-Divergent Oxidations within Venerable Macrolide Antibiotic Scaffolds Unveil Compounds with Broad Spectrum and Anti-MRSA Activities
Source: ACS Cent Sci. 2026 Mar 17;12(3):375–82. doi: 10.1021/acscentsci.5c02343 (PMC13022725; doi:10.1021/acscentsci.5c02343)
Supplement: Supplementary file 3 [file oc5c02343_si_003.zip › Erythromycin Analog Characterization 13,14,15/14/IR/OL-III-122.pdf]

SHIMADZU

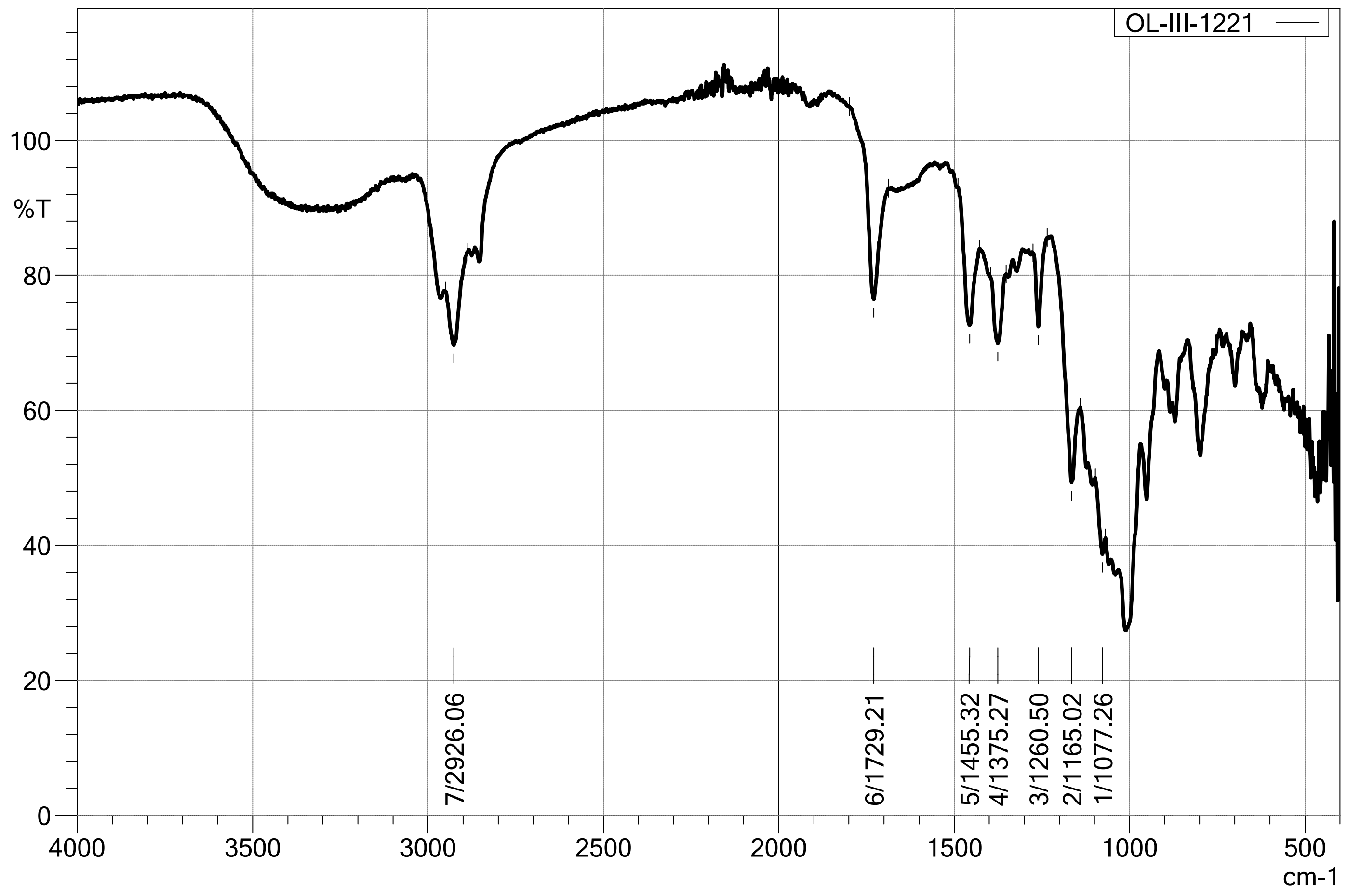

C:¥LabSolutions¥LabSolutionsIR¥Data¥Miller_OliviaL¥OL-III-1221.ispd

	Item	Value
2	Sample name	
3	Sample ID	
4	Option	
5	Intensity Mode	%Transmittance
6	Apodization	Happ-Genzel
9	No. of Scans	16
10	Resolution	2 cm-1

	Peak	Intensity	Corr. Intensity	Base (H)	Base (L)	Area	Corr. Area	Comment
1	1077.26	38.70	5.04	1097.52	1068.58	1654.377	78.170	
2	1165.02	49.30	19.13	1215.17	1139.95	2649.888	575.762	
3	1260.50	72.37	11.77	1274.97	1234.46	810.978	181.668	
4	1375.27	69.90	10.10	1396.49	1352.12	1118.154	231.313	
5	1455.32	72.59	15.39	1489.07	1428.31	1161.096	461.563	
6	1729.21	76.44	20.99	1798.65	1687.74	844.077	728.474	
7	2926.06	69.67	10.23	2950.17	2888.45	1503.261	301.555	
