# Supplementary material for: Site-Divergent Oxidations within Venerable Macrolide Antibiotic Scaffolds Unveil Compounds with Broad Spectrum and Anti-MRSA Activities
Source: ACS Cent Sci. 2026 Mar 17;12(3):375–82. doi: 10.1021/acscentsci.5c02343 (PMC13022725; doi:10.1021/acscentsci.5c02343)
Supplement: Supplementary file 3 [file oc5c02343_si_003.zip › Erythromycin Analog Characterization 13,14,15/14/HRMS/OL-III-122-HRMS.pdf]

C:\Xcalibur\data\OL-111-122_pos_v1 09/26/25 12:34:40

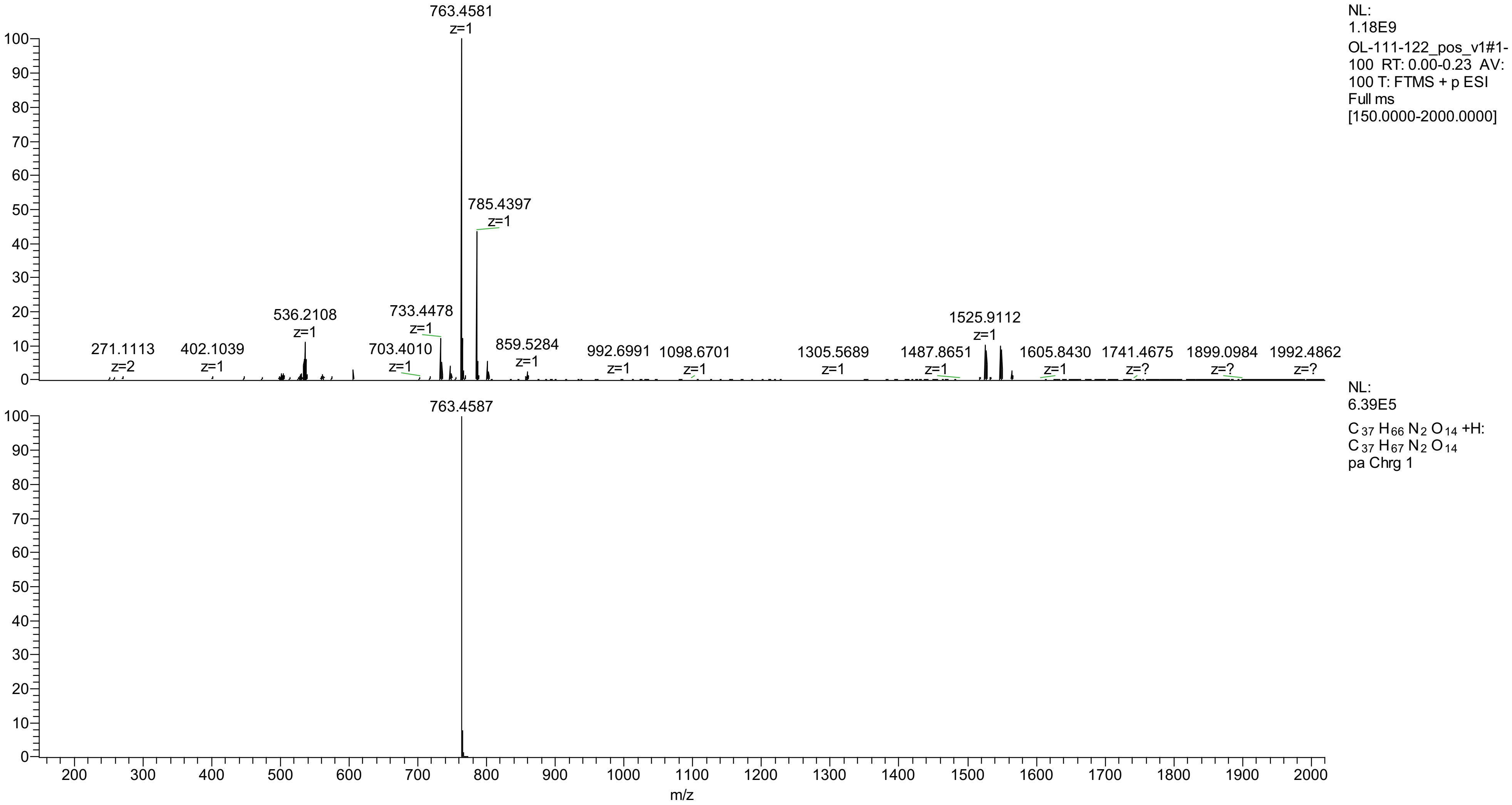

C:\Xcalibur\data\OL-111-122_pos_v1 09/26/25 12:34:40

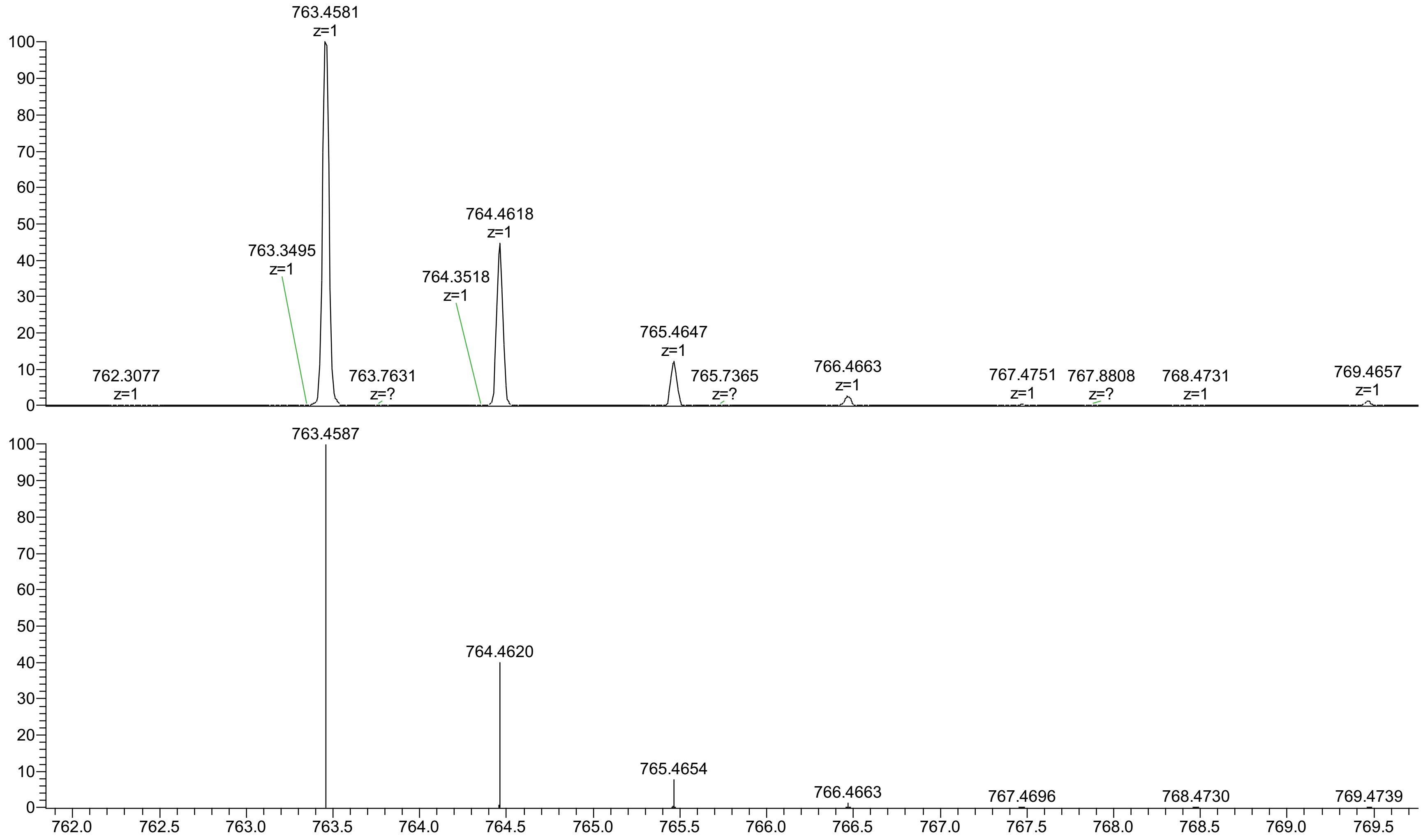

NL:
1.18E9
OL-111-122_pos_v1#1-100 RT: 0.00-0.23 AV: 100 T: FTMS + p ESI Full ms [150.0000-2000.0000]

NL:
6.39E5
$C_{37}H_{66}N_2O_{14}$ +H:
$C_{37}H_{67}N_2O_{14}$
pa Chrg 1
