# Supplementary material for: Site-Divergent Oxidations within Venerable Macrolide Antibiotic Scaffolds Unveil Compounds with Broad Spectrum and Anti-MRSA Activities
Source: ACS Cent Sci. 2026 Mar 17;12(3):375–82. doi: 10.1021/acscentsci.5c02343 (PMC13022725; doi:10.1021/acscentsci.5c02343)
Supplement: Supplementary file 4 [file oc5c02343_si_004.zip › Clarithromycin and Azithromycin Analog Characterization/7/IR/OL-III-019.pdf]

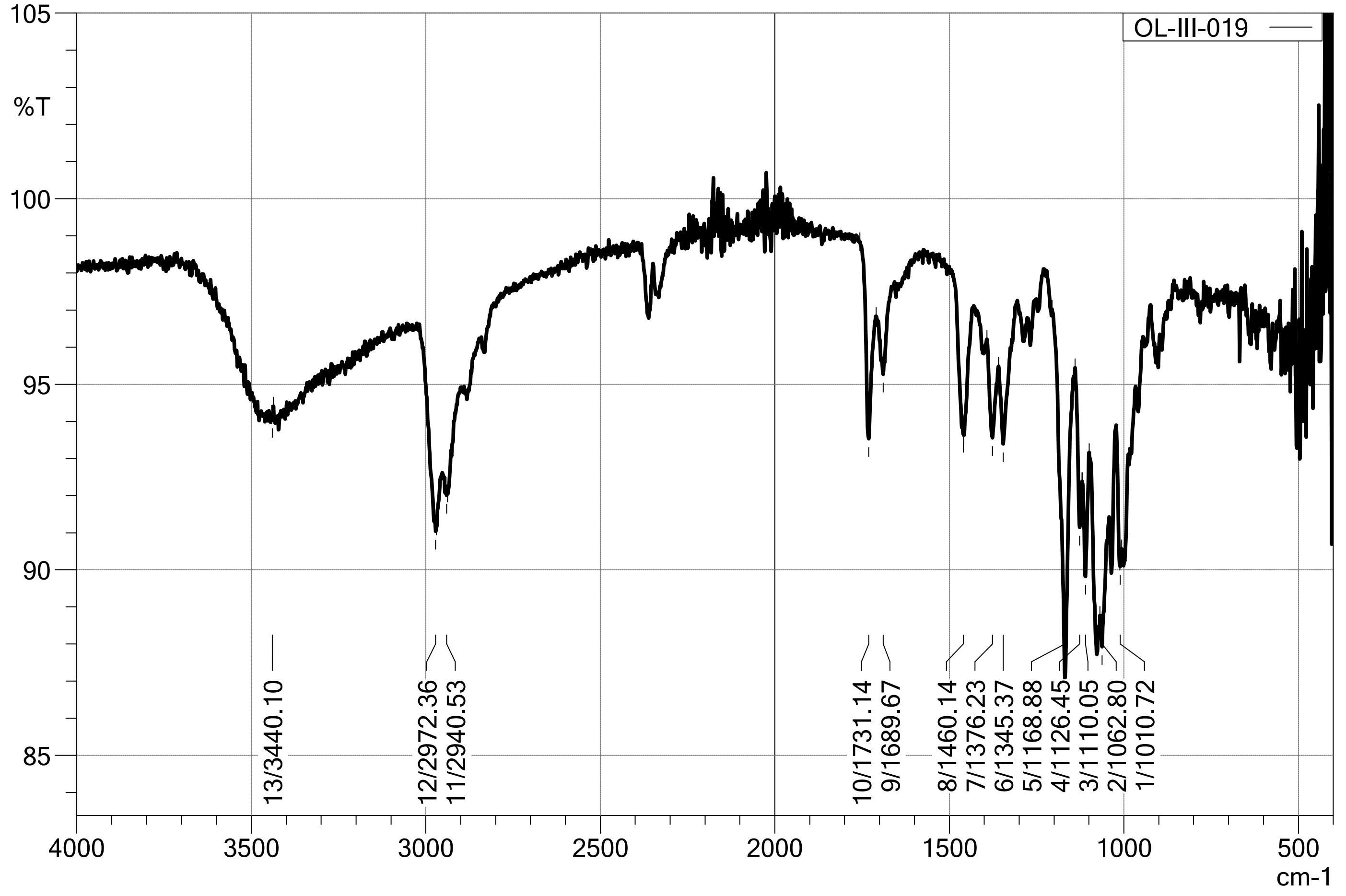

C:¥LabSolutions¥LabSolutionsIR¥Data¥Miller_OliviaL¥OL-III-019.ispd

	Item	Value
2	Sample name	
3	Sample ID	
4	Option	
5	Intensity Mode	%Transmittance
6	Apodization	Happ-Genzel
9	No. of Scans	32
10	Resolution	2 cm-1

	Peak	Intensity	Corr. Intensity	Base (H)	Base (L)	Area	Corr. Area	Comment
1	1010.72	90.09	0.20	1011.68	1005.89	56.041	0.504	
2	1062.80	87.93	1.43	1068.58	1048.33	220.564	13.736	
3	1110.05	89.82	2.93	1119.70	1099.44	174.548	28.209	
4	1126.45	91.14	2.26	1139.95	1119.70	143.660	20.352	
5	1168.88	87.09	8.89	1205.53	1142.84	453.759	210.865	
6	1345.37	93.39	2.29	1358.87	1325.12	182.493	38.485	
7	1376.23	93.56	2.30	1392.63	1358.87	179.561	39.507	
8	1460.14	93.65	0.05	1463.03	1459.17	24.228	0.107	
9	1689.67	95.27	0.42	1694.49	1686.78	35.033	1.810	
10	1731.14	93.54	4.22	1756.22	1709.92	170.277	70.297	
11	2940.53	92.01	0.17	2942.46	2937.64	38.213	0.404	
12	2972.36	91.03	0.24	2975.25	2969.46	51.051	0.555	
13	3440.10	94.05	0.21	3442.03	3436.24	33.674	0.667	
