# Supplementary material for: Site-Divergent Oxidations within Venerable Macrolide Antibiotic Scaffolds Unveil Compounds with Broad Spectrum and Anti-MRSA Activities
Source: ACS Cent Sci. 2026 Mar 17;12(3):375–82. doi: 10.1021/acscentsci.5c02343 (PMC13022725; doi:10.1021/acscentsci.5c02343)
Supplement: Supplementary file 4 [file oc5c02343_si_004.zip › Clarithromycin and Azithromycin Analog Characterization/16/IR/OL-III-049.pdf]

SHIMADZU

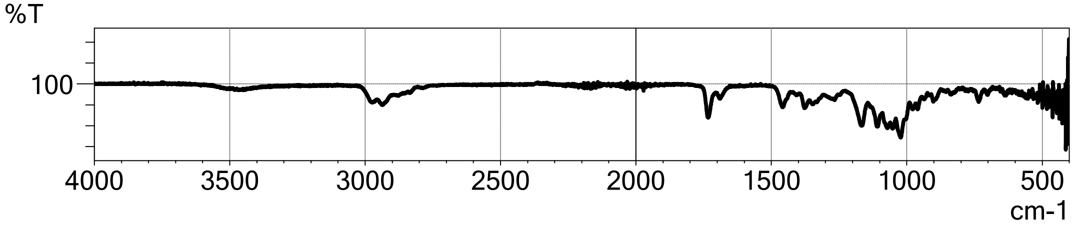

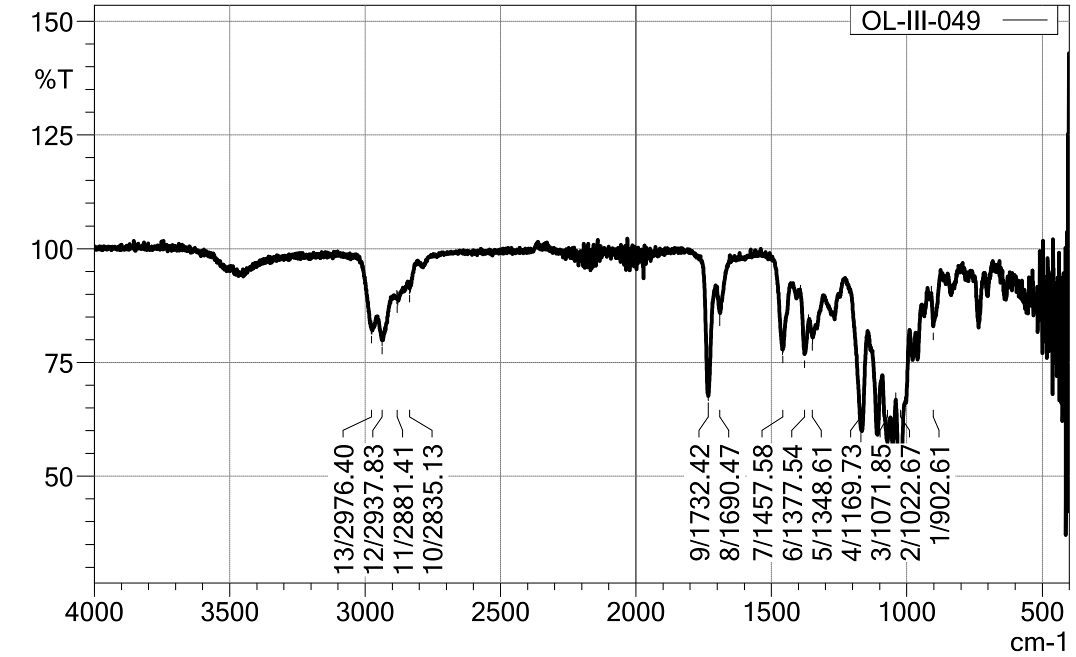

	Peak	Intensity	Corr. Intensity	Base (H)	Base (L)	Area	Corr. Area	Comment
1	902.61	82.94	3.78	909.84	898.27	165.886	20.424	
2	1022.67	48.56	17.77	1040.99	1008.69	1390.720	305.330	
3	1071.85	57.39	1.82	1074.26	1066.06	338.993	8.594	
4	1169.73	60.49	0.65	1185.64	1169.25	524.190	11.036	
5	1348.61	80.62	0.17	1351.99	1348.13	72.957	0.433	
6	1377.54	76.80	10.36	1392.97	1362.60	545.408	157.066	
7	1457.58	77.90	0.57	1458.55	1456.62	41.917	0.395	
8	1690.47	86.05	0.29	1696.25	1689.99	80.703	3.884	
9	1732.42	67.60	1.66	1733.38	1716.99	379.461	20.560	
10	2835.13	91.24	0.43	2836.09	2832.23	32.356	0.588	
11	2881.41	88.92	0.28	2882.86	2879.97	31.515	0.276	
12	2937.83	79.84	0.59	2944.09	2936.86	139.310	3.117	
13	2976.40	82.25	0.50	2977.85	2974.95	50.662	0.752	

C:¥LabSolutions¥LabSolutionsIR¥Data
¥Miller_OliviaL¥OL-III-049.ispd

	Item	Value
2	Sample name	
3	Sample ID	
4	Option	
5	Intensity Mode	%Transmittance
6	Apodization	Happ-Genzel
9	No. of Scans	16
10	Resolution	1 cm-1
