# Supplementary material for: Site-Divergent Oxidations within Venerable Macrolide Antibiotic Scaffolds Unveil Compounds with Broad Spectrum and Anti-MRSA Activities
Source: ACS Cent Sci. 2026 Mar 17;12(3):375–82. doi: 10.1021/acscentsci.5c02343 (PMC13022725; doi:10.1021/acscentsci.5c02343)

==== Shimadzu LabSolutions Browser Report ====

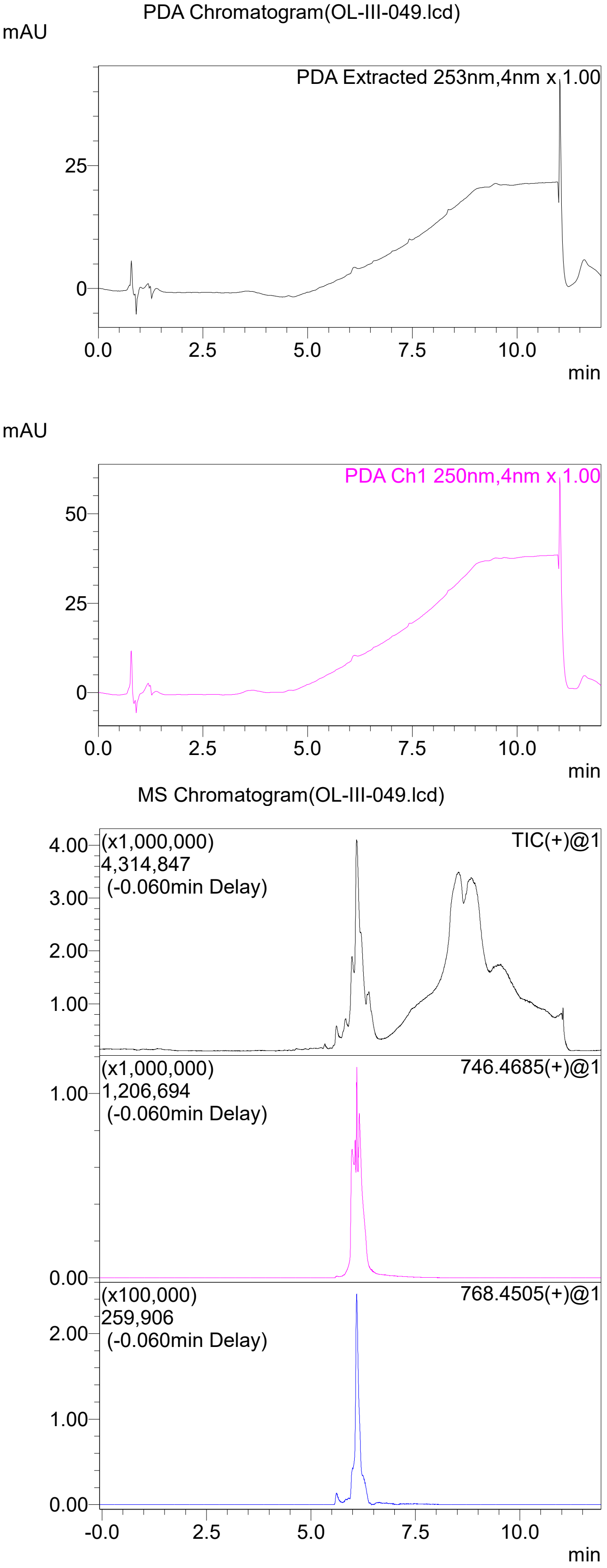

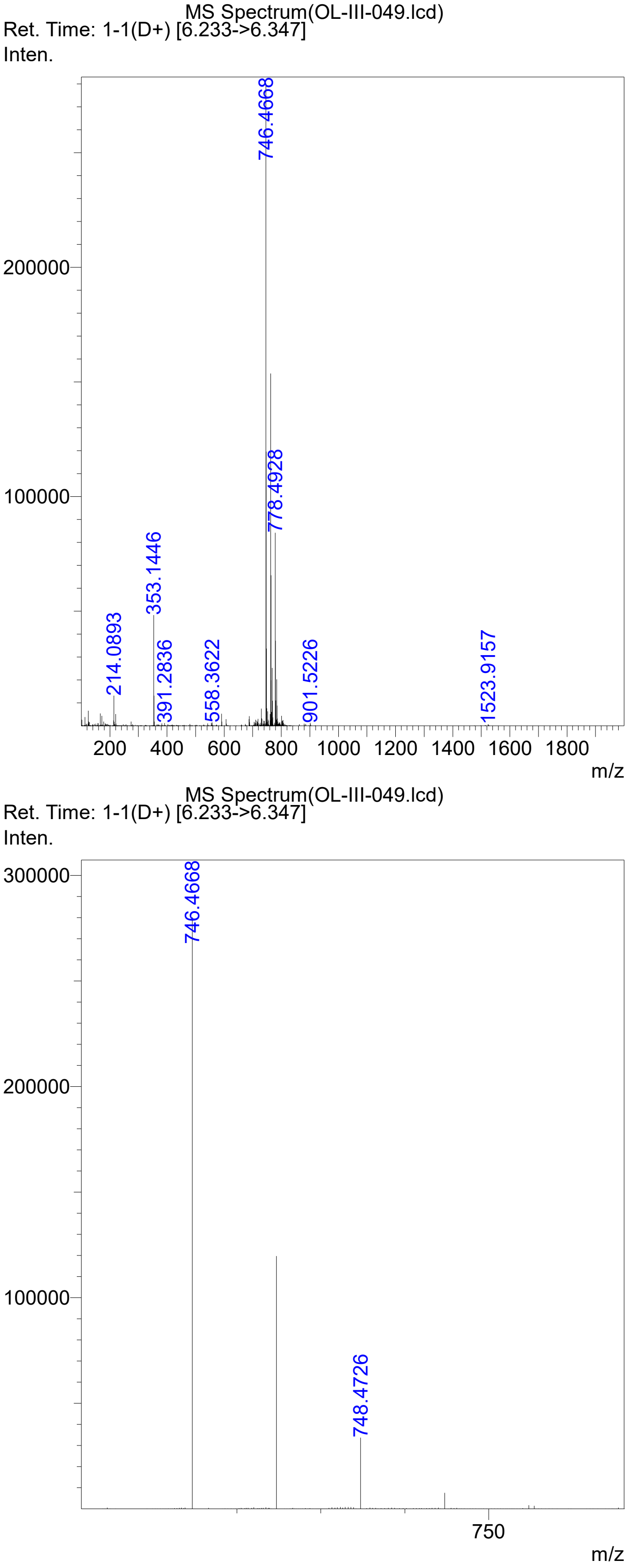

Supplement: Supplementary file 4 [file oc5c02343_si_004.zip › Clarithromycin and Azithromycin Analog Characterization/16/HRMS/OL-III-049.pdf]
