# Supplementary material for: Site-Divergent Oxidations within Venerable Macrolide Antibiotic Scaffolds Unveil Compounds with Broad Spectrum and Anti-MRSA Activities
Source: ACS Cent Sci. 2026 Mar 17;12(3):375–82. doi: 10.1021/acscentsci.5c02343 (PMC13022725; doi:10.1021/acscentsci.5c02343)
Supplement: Supplementary file 4 [file oc5c02343_si_004.zip › Clarithromycin and Azithromycin Analog Characterization/10/IR/OL-III-042.pdf]

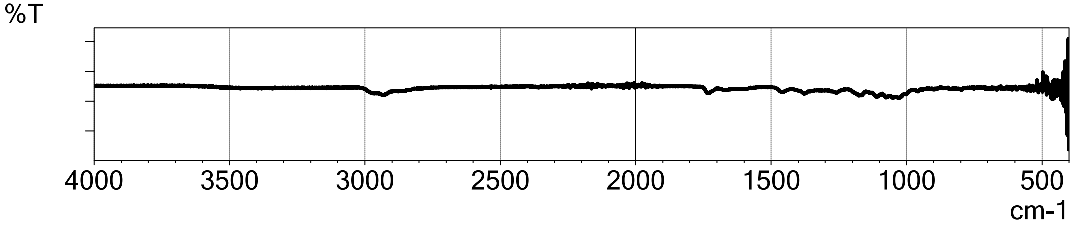

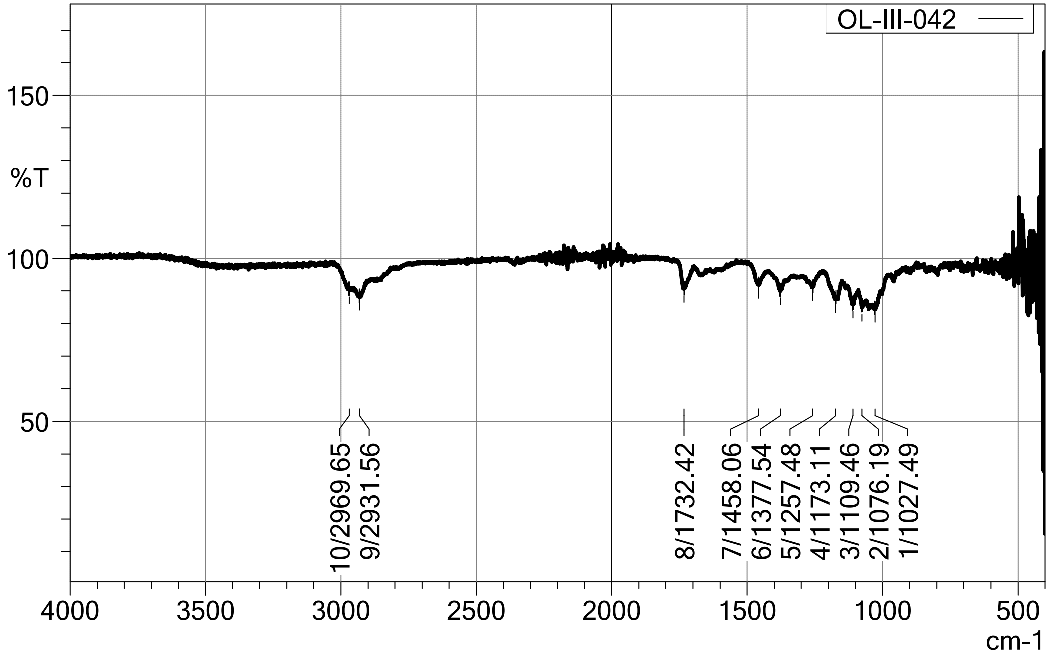

	Peak	Intensity	Corr. Intensity	Base (H)	Base (L)	Area	Corr. Area	Comment
1	1027.49	84.57	0.19	1028.46	1027.01	22.160	0.152	
2	1076.19	84.79	0.84	1077.15	1074.26	42.898	1.393	
3	1109.46	85.83	0.31	1109.94	1108.01	26.877	0.239	
4	1173.11	87.49	0.26	1173.59	1171.66	23.729	0.363	
5	1257.48	91.19	0.24	1258.45	1256.52	16.774	0.240	
6	1377.54	89.97	0.33	1380.44	1376.58	37.845	0.582	
7	1458.06	91.88	0.20	1459.03	1457.10	15.525	0.247	
8	1732.42	90.65	0.20	1733.38	1731.93	13.393	0.201	
9	2931.56	88.30	0.21	2932.52	2931.08	16.827	0.155	
10	2969.65	90.27	0.46	2971.10	2967.72	32.039	0.735	

C:¥LabSolutions¥LabSolutionsIR¥Data
¥Miller_OliviaL¥OL-III-042.ispd

	Item	Value
2	Sample name	
3	Sample ID	
4	Option	
5	Intensity Mode	%Transmittance
6	Apodization	Happ-Genzel
9	No. of Scans	16
10	Resolution	1 cm-1
