# Supplementary material for: Site-Divergent Oxidations within Venerable Macrolide Antibiotic Scaffolds Unveil Compounds with Broad Spectrum and Anti-MRSA Activities
Source: ACS Cent Sci. 2026 Mar 17;12(3):375–82. doi: 10.1021/acscentsci.5c02343 (PMC13022725; doi:10.1021/acscentsci.5c02343)
Supplement: Supplementary file 4 [file oc5c02343_si_004.zip › Clarithromycin and Azithromycin Analog Characterization/8/IR/OL-III-021.pdf]

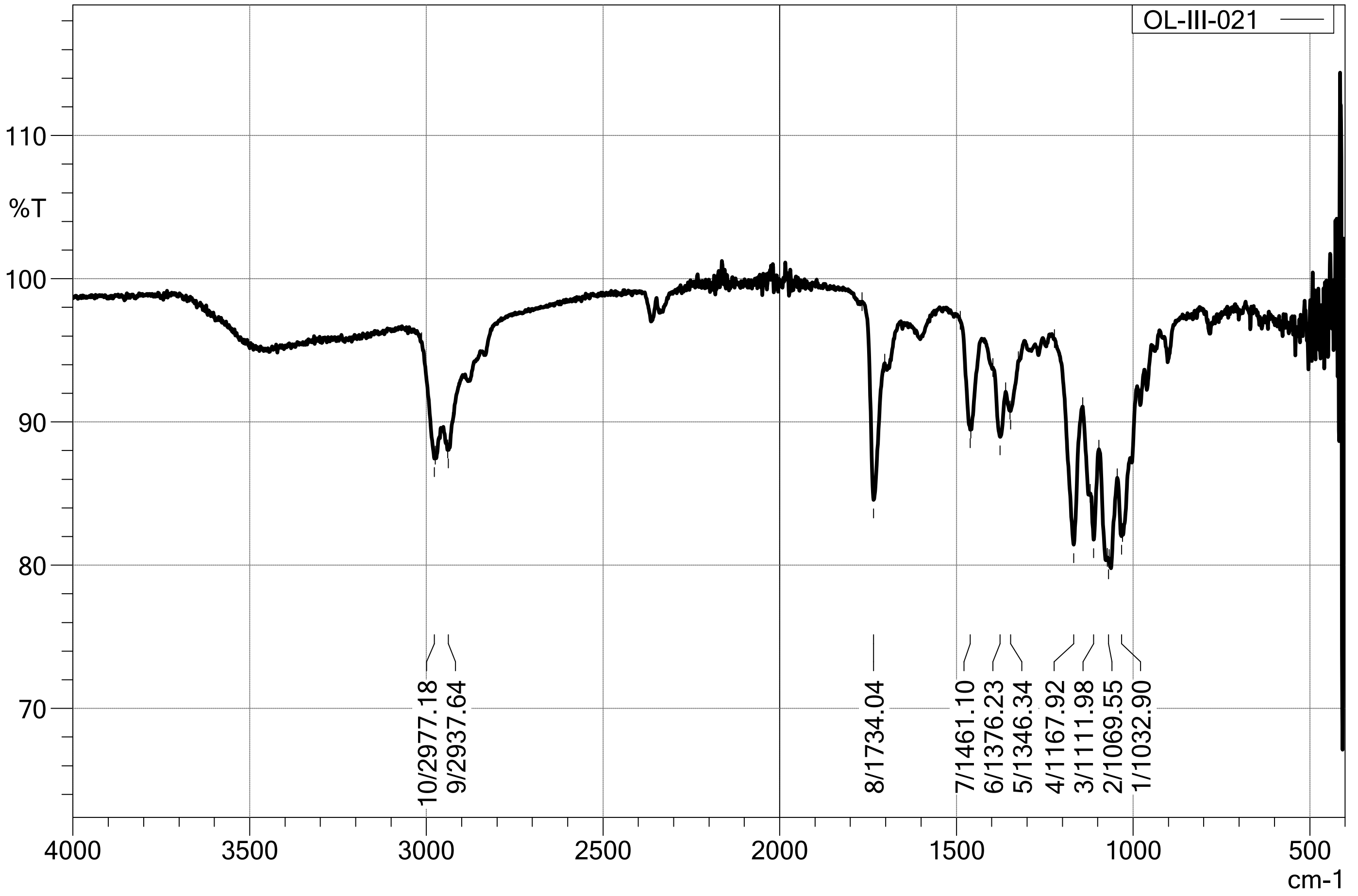

C:¥LabSolutions¥LabSolutionsIR¥Data¥Miller_OliviaL¥OL-III-021.ispd

	Item	Value
2	Sample name	
3	Sample ID	
4	Option	
5	Intensity Mode	%Transmittance
6	Apodization	Happ-Genzel
9	No. of Scans	32
10	Resolution	2 cm-1

	Peak	Intensity	Corr. Intensity	Base (H)	Base (L)	Area	Corr. Area	Comment
1	1032.90	82.02	1.02	1044.47	1030.01	235.877	7.107	
2	1069.55	80.32	0.17	1073.40	1067.62	113.303	0.460	
3	1111.98	81.79	4.40	1121.63	1096.55	386.575	49.419	
4	1167.92	81.43	11.13	1222.89	1142.84	846.667	321.688	
5	1346.34	90.76	0.35	1348.27	1324.15	187.023	7.443	
6	1376.23	88.96	3.88	1396.49	1360.80	323.125	71.710	
7	1461.10	89.47	0.54	1489.07	1459.17	197.295	-2.803	
8	1734.04	84.56	11.62	1766.83	1703.17	507.991	269.368	
9	2937.64	88.04	0.35	2939.57	2926.06	151.625	4.069	
10	2977.18	87.45	0.80	3013.83	2974.29	337.074	6.440	
