# Supplementary material for: Site-Divergent Oxidations within Venerable Macrolide Antibiotic Scaffolds Unveil Compounds with Broad Spectrum and Anti-MRSA Activities
Source: ACS Cent Sci. 2026 Mar 17;12(3):375–82. doi: 10.1021/acscentsci.5c02343 (PMC13022725; doi:10.1021/acscentsci.5c02343)
Supplement: Supplementary file 5 [file oc5c02343_si_005.zip › Biological, Computational, and X-ray Data/Biological/Final Report 11-14-2024- Yale 16.pdf]

FINAL REPORT

TITLE: The *In vitro* Activity of Thiostrepton Analogs from Yale University against a Collection of Gram-positive Pathogens

REPORT NUMBER: 11-14-2024-Yale 16

QUOTE NUMER: 09-04-2024-Yale 19

DATE: November 15, 2024

AUTHORS:

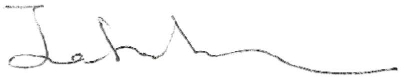

Seyedehameneh Jahanbakhsh, Ph.D.
Scientist

STUDY DIRECTOR :

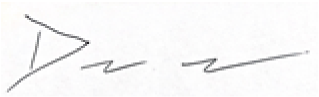

David Hufnagel, Ph.D.
Senior Scientist

SITE: Microbiologics
4717 Campus Drive
Kalamazoo, MI, USA 49008

INTRODUCTION

The Miller lab at Yale University is investigating novel thiostrepton-based compounds. This study evaluated in vitro activity of the compounds by broth microdilution following guidance of the Clinical and Laboratory Standards Institute (CLSI; 1, 2) against a collection of staphylococci, enterococci, and streptococci with different antibiotic resistance phenotypes.

MATERIALS AND METHODS

Test agents and comparators

The test agents, thiostrepton, and teicoplanin were provided by Yale and were stored at room temperature prior to testing. The comparator compounds vancomycin, linezolid and ceftazidime were provided by Microbiologics. Stock solutions of each compound were prepared on the day of testing using solvents recommended by CLSI and the client. Stock solutions of each compound were made at 101X the final testing concentration. DMSO (Sigma; St. Louis, MO; Lot No. MKCP0105) was used as the solvent and diluent for Yale compounds. Information regarding compound source, lot number, testing concentrations, and solvent/diluent information for the test agents and comparators are detailed below:

Test Agent	Supplier	Cat No./ Lot No.	Test Concentration Range (µg/mL)	Solvent/Diluent
POP-I-237	Yale		0.03-32/0.001-1[a]	DMSO/DMSO
POP-I-238	Yale		0.03-32/0.001-1	DMSO/DMSO
POP-I-252	Yale		0.03-32/0.001-1	DMSO/DMSO
POP-I-279	Yale		0.03-32/0.001-1	DMSO/DMSO
POP-I-277	Yale		0.03-32/0.001-1	DMSO/DMSO
POP-I-281	Yale		0.03-32/0.001-1	DMSO/DMSO
POP-I-167	Yale		0.03-32/0.001-1	DMSO/DMSO
POP-I-093	Yale		0.03-32/0.001-1	DMSO/DMSO
POP-I-118	Yale		0.03-32/0.001-1	DMSO/DMSO
POP-I-088	Yale		0.03-32/0.001-1	DMSO/DMSO
POP-I-280	Yale		0.03-32/0.001-1	DMSO/DMSO
POP-I-224A	Yale		0.03-32/0.001-1	DMSO/DMSO
POP-I-239A	Yale		0.03-32/0.001-1	DMSO/DMSO
POP-I-183	Yale		0.03-32/0.001-1	DMSO/DMSO
POP-II-001	Yale		0.03-32/0.001-1	DMSO/DMSO
POP-I-185	Yale		0.03-32/0.001-1	DMSO/DMSO
OL-CL	Yale		0.03-32/0.001-1	DMSO/DMSO
OL-CR	Yale		0.03-32/0.001-1	DMSO/DMSO
OL-II-281	Yale		0.03-32/0.001-1	DMSO/DMSO
OL-II-283	Yale		0.03-32/0.001-1	DMSO/DMSO
OL-II-280	Yale		0.03-32/0.001-1	DMSO/DMSO
OL-II-290	Yale		0.03-32/0.001-1	DMSO/DMSO
OL-II-275	Yale		0.03-32/0.001-1	DMSO/DMSO
Thiostrepton	Yale		0.03-32/0.001-1	DMSO/DMSO
Teicoplanin	Yale		0.03-32/0.001-1	DMSO/DMSO
Vancomycin	Sigma	V2002/080M1341V	0.016-16	diH_2O/diH_2O

Linezolid	Selleckchem	S1408/S140802	0.016-16	diH_2O/diH_2O
Ceftazidime	Sigma	C3809/117M4826V	0.016-16	diH_2O/diH_2O

[a]Yale compounds test ranges against streptococci were 0.001-1 µg/mL

Test organisms

The test organisms evaluated in this study consisted of reference strains from the American Type Culture Collection (ATCC; Manassas, VA) and clinical isolates from the Microbiologics repository (MMX; Kalamazoo, MI).

Upon receipt at Microbiologics, the isolates were streaked under suitable conditions onto agar media appropriate to each organism and were incubated for 18 to 24 hr at 35ºC. Colonies harvested from these growth plates were resuspended in the appropriate medium containing a cryoprotectant. Aliquots of each suspension were then frozen at -80ºC. Prior to testing, the isolates were streaked from frozen vials onto Trypticase soy agar with 5% sheep blood (Remel; Lenexa, KS; Lot No. 2146991) and incubated under optimal conditions for growth.

Test medium

The test medium used for the broth microdilution was cation adjusted Mueller Hinton broth (CAMHB; Becton Dickinson [BD]; Franklin Lakes, NJ; Lot No. 4102223). The medium was prepared according to guidelines from CLSI (1, 2). For *S. pneumoniae,* this medium was supplemented with 3% laked horse blood (LHB; Hemostat, Dixon, CA; Lot No. 788088-1-1).

Broth Microdilution MIC Assay

The MIC assay method followed the broth microdilution procedure described by CLSI (1, 2) and employed automated liquid handlers to conduct serial dilutions and liquid transfers. Automated liquid handlers included the Multidrop 384 (Labsystems, Helsinki, Finland), Biomek 3000, and Biomek FX (Beckman Coulter, Fullerton CA).

The wells in columns 2 through 12 in a standard 96-well microdilution plate (Costar 3795) were filled with 150 µl of the appropriate diluent. These plates would become the 'mother plates' from which 'daughter' or test plates would be prepared. The drugs (300 µL at 101X the desired top concentration in the test plates) were dispensed into the appropriate well in column 1 of the mother plates. The Biomek 3000 was used to make serial two-fold dilutions through column 11 in the "mother plates." The wells of column 12 contained no drug and served as the organism growth control wells.

Rows A through H of the daughter plates were loaded with 190 µL per well of the appropriate test medium using Multidrop 384. The daughter plates were prepared using the Biomek FX which transferred 2 µL of drug solution from each well of the mother plates to the corresponding well of the daughter plates in a single step.

A standardized inoculum of each organism was prepared by CLSI methods (1, 2). Colonies were picked from the primary plate and a suspension was prepared to equal a 0.5 McFarland turbidity standard. Suspensions were diluted 1:20 in the appropriate medium and transferred to

compartments of sterile reservoirs divided by length (Beckman Coulter). The Biomek 3000 was used to inoculate the plates. Daughter plates were placed on the Biomek 3000 in reverse orientation so that plates were inoculated from low to high drug concentration. The Biomek 3000 delivered 10 µL of standardized inoculum into each well of the appropriate daughter plate for an additional 1:20 dilution targeting a final inoculum concentration of approximately 5 x 10^5 CFU/mL.

The plates were stacked 3 to 4 high, covered with a sterile lid on the top plate, and incubated aerobically at 35ºC for 16 to 24 hr according to CLSI standards. Following incubation, the microplates were removed from the incubator and viewed from the bottom using a plate viewer. For each of the test media and drugs, an un-inoculated solubility control plate was observed for evidence of drug precipitation. The MIC was recorded as the lowest concentration of drug that completely inhibited visible growth of the organism.

RESULTS AND DISCUSSION

Results of susceptibility testing of the Yale test compounds are shown in **Table 1.** No precipitation was observed with test articles in this study. The comparators were within the established CLSI quality control ranges for *Staphylococcus aureus* ATCC 29213, *Enterococcus faecalis* ATCC 29212, *Streptococcus pneumoniae* ATCC 49619, and *Escherichia coli* ATCC 25922 (**Table 1**).

Staphylococci
Against the methicillin-susceptible *S. aureus* (MSSA) ATCC 29213, test articles OL-CL and OL-CR had the lowest MIC values of 0.25 and 0.5 µg/mL, respectively, while OL-II-283, POP-I-237, and POP-I-238 had MIC values of 2 or 4 µg/mL. Against the methicillin-resistant *S. aureus* (MRSA) NRS384, POP-I-237 and POP-I-238 had the lowest MIC values (4 µg/mL), with the rest of the compounds having MIC values ≥16 µg/mL. The MIC value for thiostrepton was observed to be 0.06 µg/mL against this organism and ≤0.03 µg/mL against *S. aureus* NRS384.

Enterococci
Against the enterococci, POP-I-237, POP-I-238 demonstrated the broadest activity with MIC values of 4 and 8 µg/mL against all 5 isolates isolates. Also, OL-CL, OL-CR, and OL-II-283 showed good activity against the vancomycin-susceptible enterococci (VSE; *E. faecalis* ATCC 29212 and *E. faecium* ATCC 19434) with MIC values ranging from 1 to 4 µg/mL; however, these 3 compounds showed no activity against the 3 vancomycin-resistant enterococci (VRE). The remaining compounds all had activity ≥16 µg/mL. The MIC value for thiostrepton was ≤ 0.03 or 0.06 µg/mL against the enterococci.

Streptococci
Against the streptococci, OL-CL and OL-CR showed the strongest activity with MIC values of 0.016 to 0.03 µg/mL. POP-I-237, POP-I-238, POP-I-281, OL-II-281, OL-II-283, OL-II-280, and OL-II-290 had MIC values ranging from 0.12 to 1 µg/mL; while the remaining compounds were inactive (MIC values of >1 µg/mL). The MIC value for thiostrepton was observed to be ≤0.001 and 0.002 µg/mL against *S. pneumoniae* and *S. pyogenes*, respectively.

Overall, POP-I-237 and POP-I-238 showed the broadest activity with MIC values generated against all Gram-positive test organisms. OL-CL and OL-CR showed the most potent activity against MSSA, VSE, and the streptococci; however, little to no activity was observed when testing against MRSA or VRE. OL-II-283 showed the same spectrum of activity to OL-CL and OL-CR with slightly higher MIC values against MSSA, VSE, and streptococci. OL-CL was the only test compound to show activity against *E. coli* ATCC 25922 (MIC value of 32 µg/mL). Eleven test compounds did not show activity in this study.

REFERENCES

1.) Clinical and Laboratory Standards Institute (CLSI). *Methods for Dilution Antimicrobial Susceptibility Tests for Bacteria That Grow Aerobically*. 11th ed. CLSI standard M07. CLSI, 950 West Valley Road, Suite 2500, Wayne, Pennsylvania 19087 USA, 2018.

2.) CLSI. *Performance Standards for Antimicrobial Susceptibility Testing.* 34th ed. CLSI supplement M100. CLSI, 950 West Valley Road, Suite 2500, Wayne, Pennsylvania 19087 USA, 2022.

Table 1. Activity of Yale test agents against Gram-positive pathogens, including resistant isolates

Species	Isolate info	MIC (µg/mL)															
		**POP-I-237**	**POP-I-238**	**POP-I-252**	**POP-I-279**	**POP-I-277**	**POP-I-281**	**POP-I-167**	**POP-I-093**	**POP-I-118**	**POP-I-088**	**POP-I-280**	**POP-I-224A**	**POP-I-239A**	**POP-I-183**	**POP-II-001**	**POP-I-185**
S. aureus	ATCC 29213 (MSSA; QC)	4	4	>32	32	32	16	>32	>32	>32	>32	>32	>32	>32	>32	>32	>32
	NRS384 (USA300; MRSA)	4	4	>32	32	32	16	>32	>32	>32	>32	>32	>32	>32	>32	>32	>32
E. faecalis	ATCC 29212 (VSE; QC)	4	4	>32	32	32	32	>32	>32	>32	>32	>32	>32	>32	>32	>32	>32
	MMX 486 (VanA; VRE)	8	4	>32	>32	>32	32	>32	>32	>32	>32	>32	>32	>32	>32	>32	>32
E. faecium	ATCC 19434 (VSE)	4	4	>32	32	>32	32	>32	>32	>32	>32	>32	>32	>32	>32	>32	>32
	MMX 485 (VanA; VRE)	4	4	>32	32	32	16	>32	>32	>32	>32	>32	>32	>32	>32	>32	>32
	MMX 487 (VanB; VRE)	4	4	32	32	32	32	>32	>32	>32	>32	>32	>32	>32	>32	>32	>32
S. pneumoniae	ATCC 49619 (PISP; QC)	0.25	0.25	>1	>1	>1	1	>1	>1	>1	>1	>1	>1	>1	>1	>1	>1
S. pyogenes	ATCC 19615	0.25	0.5	>1	>1	>1	1	>1	>1	>1	>1	>1	>1	>1	>1	>1	>1
E. coli	ATCC 25922 (QC)	>32	>32	>32	>32	>32	>32	>32	>32	>32	>32	>32	>32	>32	>32	>32	>32

Table 1 *continued…*

Species	Isolate info	MIC (µg/mL)											
		**OL-CL**	**OL-CR**	**OL-II-281**	**OL-II-283**	**OL-II-280**	**OL-II-290**	**OL-II-275**	**THI**	**TCO**	**VAN**	**LZD**	**CAZ**
S. aureus	ATCC 29213 (MSSA; QC)	0.25	0.5	>32	2	>32	>32	>32	0.06	0.5 (0.25-1)	1 (0.5-2)	4 (1-4)	16
	NRS384 (USA300; MRSA)	32	>32	>32	>32	>32	>32	>32	≤0.03	0.5	1	4	>16
E. faecalis	ATCC 29212 (VSE; QC)	1	2	>32	4	>32	>32	>32	≤0.03	0.25 (0.25-1)	2 (1-4)	2 (1-4)	>16
	MMX 486 (VanA; VRE)	>32	>32	>32	>32	>32	>32	>32	0.06	>32	>16	1	>16
E. faecium	ATCC 19434 (VSE)	2	2	>32	2	>32	>32	>32	0.06	0.5	0.5	4	>16
	MMX 485 (VanA; VRE)	>32	>32	>32	>32	>32	>32	>32	0.06	>32	>16	1	>16
	MMX 487 (VanB; VRE)	>32	>32	>32	>32	>32	>32	>32	≤0.03	0.5	>16	2	>16
S. pneumoniae	ATCC 49619 (PISP; QC)	0.03	0.03	1	0.12	1	1	>1	≤0.001	0.12	0.25 (0.12-05)	2 (0.25-2)	1
S. pyogenes	ATCC 19615	0.016	0.03	0.25	0.12	0.25	0.5	>1	0.002	0.12	0.25	1	0.12
E. coli	ATCC 25922 (QC)	32	>32	>32	>32	>32	>32	>32	>32	>32	>16	>16	0.25 (0.06-0.5)

THI, thiostrepton; TCO, teicoplanin; VAN, vancomycin; LNZ, linezolid; CAZ, Ceftazidime. MSSA, methicillin-susceptible Staphylococcus aureus; MRSA, methicillin-resistant *S. aureus*; VSE, vancomycin-susceptible Enterococci; VRE, vancomycin-resistant Enterococci; PISP, penicillin-intermediate resistant S. pneumoniae.
CLSI QC ranges shown in parenthesis.
