# Supplementary material for: Site-Divergent Oxidations within Venerable Macrolide Antibiotic Scaffolds Unveil Compounds with Broad Spectrum and Anti-MRSA Activities
Source: ACS Cent Sci. 2026 Mar 17;12(3):375–82. doi: 10.1021/acscentsci.5c02343 (PMC13022725; doi:10.1021/acscentsci.5c02343)
Supplement: Supplementary file 5 [file oc5c02343_si_005.zip › Biological, Computational, and X-ray Data/Biological/Final Report 04-30-2025-Yale 17.pdf]

FINAL REPORT

TITLE: Determination of the In Vitro Activity of Macrolide Analogs Against Gram-Positive and Fastidious Gram-Negative Bacteria

REPORT NUMBER: 04-30-2025-Yale 17

QUOTE NUMBER: 03-31-2025-Yale 20 (SOW O-019377)

DATE: June 20th, 2025

AUTHORS:

Chris Pillar, PhD
Director of Science and Operations

SITE: Microbiologics
4250 Commercial Ave
Portage, MI 49002

INTRODUCTION

Yale University is currently evaluating the activity of novel macrolide analogs. In this study, the activity of 12 analogs alongside comparators clarithromycin, azithromycin, erythromycin and levofloxacin was evaluated by broth microdilution against clinically relevant Gram-positive pathogens and fastidious Gram-negative respiratory pathogens including those with important macrolide-resistance mechanisms. Broth microdilution susceptibility testing was conducted in accordance with guidelines recommended by the Clinical and Laboratory Standards Institute (CLSI; 1-3).

MATERIALS AND METHODS

Test Articles and Comparators

The test articles were provided in powder form by Yale University. The compounds were stored at room temperature prior to testing. Comparator compounds were provided by Microbiologics. Stock solutions of all test compounds were prepared on the day of testing using DMSO for the Yale test agents and solvents recommended by the CLSI for comparators (3). Stock solutions of test agents were made at 101X the final testing concentration. Information regarding test agents and comparators is detailed below:

Test Articles	Supplier	Cat. No./ Lot No.	Test concentrations (µg/mL)	Solvent/Diluent
OL-II-283	Yale	-	0.06-64	DMSO/DMSO
OL-III-019	Yale	-	0.06-64	DMSO/DMSO
OL-III-021	Yale	-	0.06-64	DMSO/DMSO
OL-III-023	Yale	-	0.06-64	DMSO/DMSO
OL-III-040	Yale	-	0.06-64	DMSO/DMSO
OL-III-041B	Yale	-	0.06-64	DMSO/DMSO
OL-III-042	Yale	-	0.06-64	DMSO/DMSO
OL-III-046	Yale	-	0.06-64	DMSO/DMSO
OL-III-049	Yale	-	0.06-64	DMSO/DMSO
OL-III-050	Yale	-	0.06-64	DMSO/DMSO
OL-III-053	Yale	-	0.06-64	DMSO/DMSO
OL-III-054	Yale	-	0.06-64	DMSO/DMSO
clarithromycin	USP	R196J0	0.016-16	water (glacial acetic acid dropwise)/water

Test Articles	Supplier	Cat. No./ Lot No.	Test concentrations (µg/mL)	Solvent/Diluent
azithromycin	USP	R103C0	0.016-16	water (glacial acetic acid dropwise)/water
erythromycin	Sigma	011M1510V	0.016-16	water (glacial acetic acid dropwise)/water
levofloxacin	Sigma	038M4848V	0.008-8	water/water

Test Organisms

Test organisms consisted of isolates from the American Type Culture Collection (ATCC; Manassas, VA), Network of Antimicrobial Resistance in *S. aureus* (NRS; BEI Resources, Manassas, VA), and the Microbiologics Repository (MMX; Kalamazoo, MI). Upon initial receipt at Microbiologics, the organisms were sub-cultured onto an appropriate agar medium and incubated under atmospheric conditions required for growth. Following incubation for 18 to 24 hr at 35°C in the appropriate atmosphere, colonies were harvested from these plates and cell suspensions were prepared and frozen at -80ºC with a cryoprotectant.

Prior to testing, isolates were streaked from frozen vials onto the appropriate agar. Unless otherwise noted, Trypticase Soy Agar with 5% sheep blood (TSA; Remel; Lenexa, KS; Lot No. 227964) was used and inoculated plates were incubated at 35°C overnight in ambient atmosphere. *Haemophilus influenzae* isolates were streaked onto Chocolate agar (Remel; Lot No. 117772).

Test Media

Organisms were tested in the appropriate media according to CLSI guidelines (1–3). Cation-adjusted Mueller Hinton broth (CAMHB; BD/BBL Lot No. 4102223) was used for broth microdilution testing with the following exceptions: CAMHB was supplemented with 5% lysed horse blood (LHB; Hemostat; Dixon, CA; Lot No. 788088) for testing of *Streptococcus* spp. and for *H. influenzae* testing, *Haemophilus* Test medium (HTM) was made by supplementing MHB (Difco, Lot No. 3150802) with 15 µg/mL nicotinamide adenine dinucleotide (NAD; Sigma; Lot No. SLBX4629), 15 µg/mL hematin porcine (Sigma; Lot No. SLCL9885), and 5 g/L of yeast extract (BD; Lot No. 7179576).

Broth Microdilution MIC Assay

The MIC assay method followed the procedures described by CLSI and employed automated liquid handlers to conduct serial dilutions and liquid transfers. Automated liquid handlers included the Multidrop Combi (ThermoScientific), Biomek 3000, and Biomek FX (Beckman Coulter; Fullerton, CA).

The wells in columns 2 through 12 in standard 96-well microdilution plates (Costar 3795) were filled with 150 µl of the appropriate diluent. These would become the "mother plates" from

which "daughter," or test plates, would be prepared. The drugs (300 µL at 101X the desired top concentration in the test plates) were dispensed into the appropriate well in column 1 of the mother plates. The Biomek 3000 was used to make serial two-fold dilutions through column 11 in the "mother plate." The wells of column 12 contained no drug and served as the organism growth control wells.

Rows A through H of the daughter plates were loaded with 190 µL per well of the appropriate test medium using the Multidrop Combi. The daughter plates were prepared using the Biomek FX which transferred 2 µL of drug solution from each well of a mother plate to the corresponding well of the daughter plate in a single step. A standardized inoculum of each organism was prepared per CLSI (1-3). Colonies were picked from the streak plate and a suspension was prepared to equal a 0.5 McFarland turbidity standard in saline. Suspensions were then diluted 1:10 and transferred to compartments of sterile reservoirs. Daughter plates were placed on the Biomek 3000 in reverse orientation so that plates were inoculated from low to high drug concentration. The Biomek 3000 delivered 10 µL of standardized inoculum into each well of the appropriate daughter plate for an additional 1:20 dilution, targeting a final concentration of 5×10^5 CFU/mL.

Plates were stacked 3 to 4 high, covered with a sterile lid on the top plate, placed in plastic bags, and incubated at 35ºC and read at 20 hr. An un-inoculated control plate was observed for evidence of drug precipitation or contamination. MIC values were read where visible growth of the organism was inhibited according to CLSI guidelines (1-3).

RESULTS AND DISCUSSION

In this study, the in vitro activity of the Yale test articles were determined alongside comparators against Gram-positive pathogens with varied susceptibility to macrolides and varied resistance mechanisms along with fastidious respiratory Gram-negative pathogens along with *Escherichia coli*. The results from broth microdilution susceptibility testing are presented in **Table 1**.

MIC values were within CLSI-established test ranges for the comparator drugs against the QC organisms, except for levofloxacin which was one-dilution above the QC range for *Haemophilus influenzae* ATCC 49247 although this agent was in QC with the three other evaluated QC organisms.

Against *Staphylococcus aureus*, OL-III-049, -050, and -054 were active against the MSSA QC isolate, CA-MRSA, and mph(C) MSSA and MRSA isolates (MIC values of 0.5-16 µg/mL, 4-64 µg/mL, and 8-16 µg/mL respectively). These compounds along with the rest of the test compounds were not active against the erm(C) of cfr resistant isolates. OL-II-283 was active against the MSSA QC isolate and MSSA mph(C) isolates with MICs of 4 µg/mL and 32 µg/mL, respectively. OL-III-019, -021, and -042 were only active against the MSSA QC isolate with MICs of 64, 64 and 16 µg/mL, respectively. The remaining compounds had no activity against *S. aureus*. Among the evaluated isolates, the CA-MRSA, mph(C) MRSA, erm(C), and cfr isolates were resistant to azithromycin, clarithromycin, and erythromycin

Against the evaluated enterococci, none of the Yale compounds were active against the evaluated erm(A) and erm(B) isolate or the vancomycin-resistant *Enterococcus faecalis* or *Enterococcus faecium*. This was consistent with the comparator macrolides. OL-II-283, OL-III-042, OL-III-049, OL-III-050, and OL-III-054 all were active against the vancomycin-susceptible *E. faecalis* and *E. faecium* isolates with OL-III-049 having the most potent activity (MICs of 1-2 µg/mL consistent with clarithromycin) followed by OL-III-054 (2-4 µg/mL consistent with erythromycin), OL-III-050 (4-8 µg/mL), OL-II-283 (4-16 µg/mL consistent with azithromycin), and OL-III-042 (32-64 µg/mL). OL-III-019 had weak activity (MIC of 64 µg/mL) for vancomycin-susceptible *E. faecalis* only.

Against the two evaluated *Streptococcus pneumoniae*, potent activity was observed for the Yale test agents against the pencillin-intermediate QC isolate with MICs of ≤0.06-0.5 µg/mL for OL-II-283, OL-III-019, OL-III-049, OL-III-050, and OL-III-054 and all other Yale test agents had MICs of 1-4 µg/mL with the exception of OL-III-053 (MIC of 16 µg/mL). This potency was consistent with what was observed against the evaluated *Streptococcus pyogenes* isolate and was also consistent with the comparator macrolides. None of the Yale test agents or comparator macrolides were active against the erm(B) *S. pneumoniae* isolate.

Against the fastidious Gram-negative respiratory pathogens, little activity was apparent against *H. influenzae* with only OL-III-049 (MIC of 16 µg/mL), OL-III-042 (MIC of 32 µg/mL), and OL-III-040 (MIC of 64 µg/mL) and OL-III-050 (MIC of 64 µg/mL) inhibiting *H. influenzae*. Broader activity was observed against *Moraxella catarrhalis* with OL-III-049 (MIC of 0.12 µg/mL) and OL-III-042 (MIC of 0.25 µg/mL) having the most potent activity which was also consistent with the other macrolides.

The Yale test agents were inactive against *E. coli* with the exception of OL-III-042 and OL-III-049 where MIC values of 64 µg/mL were observed.

In summary, OL-III-049 and OL-III-050 demonstrated the most potent inhibitory activity against the evaluated isolates and were active against *S. aureus* mph(C) macrolide-resistant isolates. OL-III-054 was also active against the mph(C) *S. aureus* but with higher MICs. OL-II-283, OL-III-042, and OL-III-019 also had activity against macrolide susceptible staphylococci and enterococci. Several Yale test agents had potent activity against streptococci and *M. catarrhalis*, some had minimal activity against *H. influenzae*, and none were active against macrolide-resistant *S. pneumoniae*.

REFERENCES

1.) Clinical and Laboratory Standards Institute (CLSI). *Methods for Dilution Antimicrobial Susceptibility Tests for Bacteria That Grow Aerobically.* 12th *ed*. CLSI standard M07. CLSI, 950 West Valley Road, Suite 2500, Wayne, Pennsylvania 19087 USA, 2024.

2.) CLSI. *Performance Standards for Antimicrobial Susceptibility Testing.* 35th ed. CLSI supplement M100. CLSI, 950 West Valley Road, Suite 2500, Wayne, Pennsylvania 19087 USA, 2025.

3.) CLSI. *Methods for Antimicrobial Dilution and Disk Diffusion Susceptibility Testing of Infrequently Isolated or Fastidious Bacteria*. 3rd ed. CLSI guideline M45. CLSI, 950 West Valley Road, Suite 2500, Wayne, Pennsylvania 19087 USA, 2016.

Table 1. Broth microdilution results evaluating the Yale test articles and comparators

Organism	Isolate	Type	MIC (µg/mL)							
			**OL-II-283**	**OL-III-019**	**OL-III-021**	**OL-III-023**	**OL-III-040**	**OL-III-041B**	**OL-III-042**	**OL-III-046**
S. aureus	ATCC 29213	MSSA, QC	4	64	64	>64	>64	>64	16	>64
	NRS 384	USA300 CA-MRSA	>64	>64	>64	>64	>64	>64	>64	>64
	MMX 2170	mph(C) MRSA	>64	>64	>64	>64	>64	>64	>64	>64
	MMX 3247	mph(C) MSSA	32	>64	>64	>64	>64	>64	>64	>64
	MMX 3037	erm(C)	>64	>64	>64	>64	>64	>64	>64	>64
	MMX 3067	cfr	>64	>64	>64	>64	>64	>64	>64	>64
E. faecalis	ATCC 29212	VSE, QC	4	64	>64	>64	>64	>64	32	>64
	MMX 0486	VanA VRE	>64	>64	>64	>64	>64	>64	>64	>64
E. faecium	ATCC 19434	VSE	16	>64	>64	>64	>64	>64	64	>64
	MMX 0485	Van VRE	>64	>64	>64	>64	>64	>64	>64	>64
	MMX 0487	Van B VRE	>64	>64	>64	>64	>64	>64	>64	>64
	MMX 0854	erm(A), erm(B)	>64	>64	>64	>64	>64	>64	>64	>64
S. pneumoniae	ATCC 49619	PISP, QC	0.12	0.5	1	1	4	4	1	4
	MMX 3033	erm(B)	>64	>64	>64	>64	>64	>64	>64	>64
S. pyogenes	ATCC 19615	-	0.12	0.12	0.5	0.25	2	4	0.5	2
H. influenzae	ATCC 49247	QC	>64	>64	>64	>64	64	>64	32	>64
	ATCC 49766	QC	>64	>64	>64	>64	64	>64	32	>64
M. catarrhalis	ATCC 25238	-	1	16	32	32	32	>64	0.25	>64
	ATCC 8176	-	1	16	32	32	32	>64	0.25	>64
E. coli	ATCC 25922	QC	>64	>64	>64	>64	>64	>64	64	>64

Table 1. Broth microdilution results evaluating the Yale test articles and comparators - *continued*

Organism	Isolate	Type	MIC (µg/mL)							
			OL-III-049	OL-III-050	OL-III-053	OL-III-054	CLA	AZM	ERY	LVX
S. aureus	ATCC 29213	MSSA, QC	0.5	4	>64	8	0.25 (0.12-0.5)[1]	1 (0.5-2)	0.5 (0.25-1)	0.5 (0.06-0.5)
	NRS 384	USA300 CA-MRSA	16	64	>64	16	>16	>16	>16	1
	MMX 2170	mph(C) MRSA	8	64	>64	16	>16	>16	>16	8
	MMX 3247	mph(C) MSSA	4	16	>64	16	4	16	8	0.25
	MMX 3037	erm(C)	>64	>64	>64	>64	>16	>16	>16	0.12
	MMX 3067	cfr	>64	>64	>64	>64	>16	>16	>16	8
E. faecalis	ATCC 29212	VSE, QC	1	4	>64	2	1	4	1 (1-4)	0.5 (0.25-2)
	MMX 0486	VanA VRE	>64	>64	>64	>64	>16	>16	>16	>8
E. faecium	ATCC 19434	VSE	2	8	>64	4	2	16	4	>8
	MMX 0485	Van VRE	>64	>64	>64	>64	>16	>16	>16	2
	MMX 0487	Van B VRE	>64	>64	>64	>64	>16	>16	>16	2
	MMX 0854	erm(A), erm(B)	>64	>64	>64	>64	>16	>16	>16	>8
S. pneumoniae	ATCC 49619	PISP, QC	≤0.06	0.12	16	0.25	0.03 (0.03-0.12)	0.06 (0.06-0.25)	0.03 (0.03-0.12)	1 (0.5-2)
	MMX 3033	erm(B)	>64	>64	>64	>64	>16	>16	>16	1
S. pyogenes	ATCC 19615	-	≤0.06	0.12	16	0.25	≤0.016	0.03	0.03	0.5
H. influenzae	ATCC 49247	QC	16	64	>64	>64	8 (4-16)	2 (1-4)	8	0.06 (0.008-0.03)
	ATCC 49766	QC	32	>64	>64	>64	16	1	8	0.03
M. catarrhalis	ATCC 25238	-	0.12	2	64	2	0.06	≤0.016	0.12	0.03
	ATCC 8176	-	0.12	1	64	2	0.06	≤0.016	0.12	0.03
E. coli	ATCC 25922	QC	64	>64	>64	>64	>16	2	>16	0.016

MIC, minimal inhibitory concentration; QC, quality control; CLA, clarithromycin; AZM, azithromycin; ERY, erythromycin; MSSA, methicillin-susceptible *S. aureus*; MRSA, methicillin-resistant *S. aureus*; CA, community-acquired; PISP, penicillin-intermediate *S. pneumoniae*; VSE, vancomycin-susceptible *Enterococcus*; VRE, vancomycin-resistant *Enterococcus*

[1]CLSI QC ranges are indicated in parentheses where applicable (2)
