# Supplementary material for: Site-Divergent Oxidations within Venerable Macrolide Antibiotic Scaffolds Unveil Compounds with Broad Spectrum and Anti-MRSA Activities
Source: ACS Cent Sci. 2026 Mar 17;12(3):375–82. doi: 10.1021/acscentsci.5c02343 (PMC13022725; doi:10.1021/acscentsci.5c02343)
Supplement: Supplementary file 5 [file oc5c02343_si_005.zip › Biological, Computational, and X-ray Data/X-Ray/7/checkCIF_PLATON page 2.pdf]

checkCIF (basic structural check) running

Checking for embedded fcf data in CIF ...
Found embedded fcf data in CIF. Extracting fcf data from uploaded CIF, please wait . . . . .

checkCIF/PLATON (basic structural check)

Structure factors have been supplied for datablock(s) syn-25016

THIS REPORT IS FOR GUIDANCE ONLY. IF USED AS PART OF A REVIEW PROCEDURE FOR PUBLICATION, IT SHOULD NOT REPLACE THE EXPERTISE OF AN EXPERIENCED CRYSTALLOGRAPHIC REFEREE.

No syntax errors found. CIF dictionary
Please wait while processing .... Interpreting this report

Structure factor report

Datablock: syn-25016

Bond precision: C-C = 0.0060 A Wavelength=1.54184

Cell:	a=9.0948(1)	b=26.4164(4)	c=9.6300(1)
	alpha=90	beta=92.930(1)	gamma=90
Temperature:	100 K		

	Calculated	Reported
Volume	2310.60(5)	2310.60(5)
Space group	P 21	P 21
Hall group	P 2yb	P 2yb
Moiety formula	C38 H69 N O14, C H Cl3, H2 O	C38 H69 N O14, C H Cl3, H2 O
Sum formula	C39 H72 Cl3 N O15	C39 H72 Cl3 N O15
Mr	901.33	901.32
Dx,g cm-3	1.296	1.295
Z	2	2
Mu (mm-1)	2.338	2.338
F000	968.0	968.0
F000'	973.06	
h,k,lmax	10,31,11	10,31,11
Nref	8232[4213]	7884
Tmin,Tmax	0.658,0.890	0.857,1.000
Tmin'	0.597	

Correction method= # Reported T Limits: Tmin=0.857 Tmax=1.000 AbsCorr = MULTI-SCAN

Data completeness= 1.87/0.96 Theta(max)= 66.982

R(reflections)= 0.0540(7463) wR2(reflections)= 0.1527(7884)

S = 1.107 Npar= 563

The following ALERTS were generated. Each ALERT has the format
test-name_ALERT_alert-type_alert-level.
Click on the hyperlinks for more details of the test.

Alert level B

PLAT417_ALERT_2_B Short Inter D-H..H-D H6 ..H10 . 2.01 Ang.
x,y,-1+z = 1_554 Check

Alert level C

PLAT244_ALERT_4_C Low 'Solvent' Ueq as Compared to Neighbors of C39 Check

PLAT336_ALERT_2_C Long Bond Distance for ..... C39 -Cl3 1.876 Ang.
PLAT336_ALERT_2_C Long Bond Distance for ..... C39 -Cl2B 1.929 Ang.
PLAT340_ALERT_3_C Low Bond Precision on C-C Bonds .... 0.00603 Ang.
PLAT911_ALERT_3_C Missing FCF Refl Between Thmin & STh/L= 0.597 37 Report
7 24 0, -7 24 1, 6 26 1, 0 31 2, -8 20 3, -7 23 3,
3 29 3, -5 26 4, 3 28 4, 7 19 5, -9 3 6, -9 9 6,
5 22 6, 2 26 6, 7 0 7, 7 1 7, 8 4 7, 8 5 7,
8 6 7, -8 7 7, 7 13 7, -5 21 7, 3 23 7, 2 24 7,
7 0 8, 7 1 8, 7 2 8, 7 3 8, 7 6 8, 7 7 8,
-4 20 8, -6 11 9, -2 19 9, -2 8 11, 1 8 11, -1 9 11,
0 9 11,

Alert level G

PLAT003_ALERT_2_G Number of Uiso or U(i,j) Restrained non-H-Atoms 4 Report
PLAT007_ALERT_5_G Number of Unrefined Donor-H Atoms .... 6 Report
H6 H10 H13 H14 H15A H15B
PLAT178_ALERT_4_G The CIF-Embedded .res File Contains SIMU Records 1 Report
PLAT302_ALERT_4_G Anion/Solvent/Minor-Residue Disorder (Resd 2) 50% Note
PLAT790_ALERT_4_G Centre of Gravity not Within Unit Cell: Resd. # 2 Note
C H Cl3
PLAT791_ALERT_4_G Model has Chirality at C2 (Sohncke SpGr) R Verify
And 17 other PLAT791 Alerts
More ...
PLAT860_ALERT_3_G Number of Least-Squares Restraints .... 13 Note
PLAT909_ALERT_3_G Percentage of I>2sig(I) Data at Theta(Max) Still 85% Note
PLAT969_ALERT_5_G The 'Henn et al.' R-Factor-gap value .... 3.142 Note
Predicted wR2: Based on SigI**2 4.86 or SHELX Weight 13.79
PLAT978_ALERT_2_G Number C-C Bonds with Positive Residual Density. 2 Info

---

0 **ALERT level A** = Most likely a serious problem - resolve or explain
1 **ALERT level B** = A potentially serious problem, consider carefully
5 **ALERT level C** = Check. Ensure it is not caused by an omission or oversight
27 **ALERT level G** = General information/check it is not something unexpected

0 ALERT type 1 CIF construction/syntax error, inconsistent or missing data
5 ALERT type 2 Indicator that the structure model may be wrong or deficient
4 ALERT type 3 Indicator that the structure quality may be low
22 ALERT type 4 Improvement, methodology, query or suggestion
2 ALERT type 5 Informative message, check

It is advisable to attempt to resolve as many as possible of the alerts in all categories. Often the minor alerts point to easily fixed oversights, errors and omissions in your CIF or refinement strategy, so attention to these fine details can be worthwhile. In order to resolve some of the more serious problems it may be necessary to carry out additional measurements or structure refinements. However, the purpose of your study may justify the reported deviations and the more serious of these should normally be commented upon in the discussion or experimental section of a paper or in the "special_details" fields of the CIF. checkCIF was carefully designed to identify outliers and unusual parameters, but every test has its limitations and alerts that are not important in a particular case may appear. Conversely, the absence of alerts does not guarantee there are no aspects of the results needing attention. It is up to the individual to critically assess their own results and, if necessary, seek expert advice.

Publication of your CIF in IUCr journals

A basic structural check has been run on your CIF. These basic checks will be run on all CIFs submitted for publication in IUCr journals (*Acta Crystallographica*, *Journal of Applied Crystallography*, *Journal of Synchrotron Radiation*); however, if you intend to submit to *Acta Crystallographica Section C* or *E* or *IUCrData*, you should make sure that full publication checks are run on the final version of your CIF prior to submission.

Publication of your CIF in other journals

Please refer to the *Notes for Authors* of the relevant journal for any special instructions relating to CIF submission.

PLATON version of 02/02/2025; check.def file version of 02/02/2025

Datablock syn-25016 - ellipsoid plot

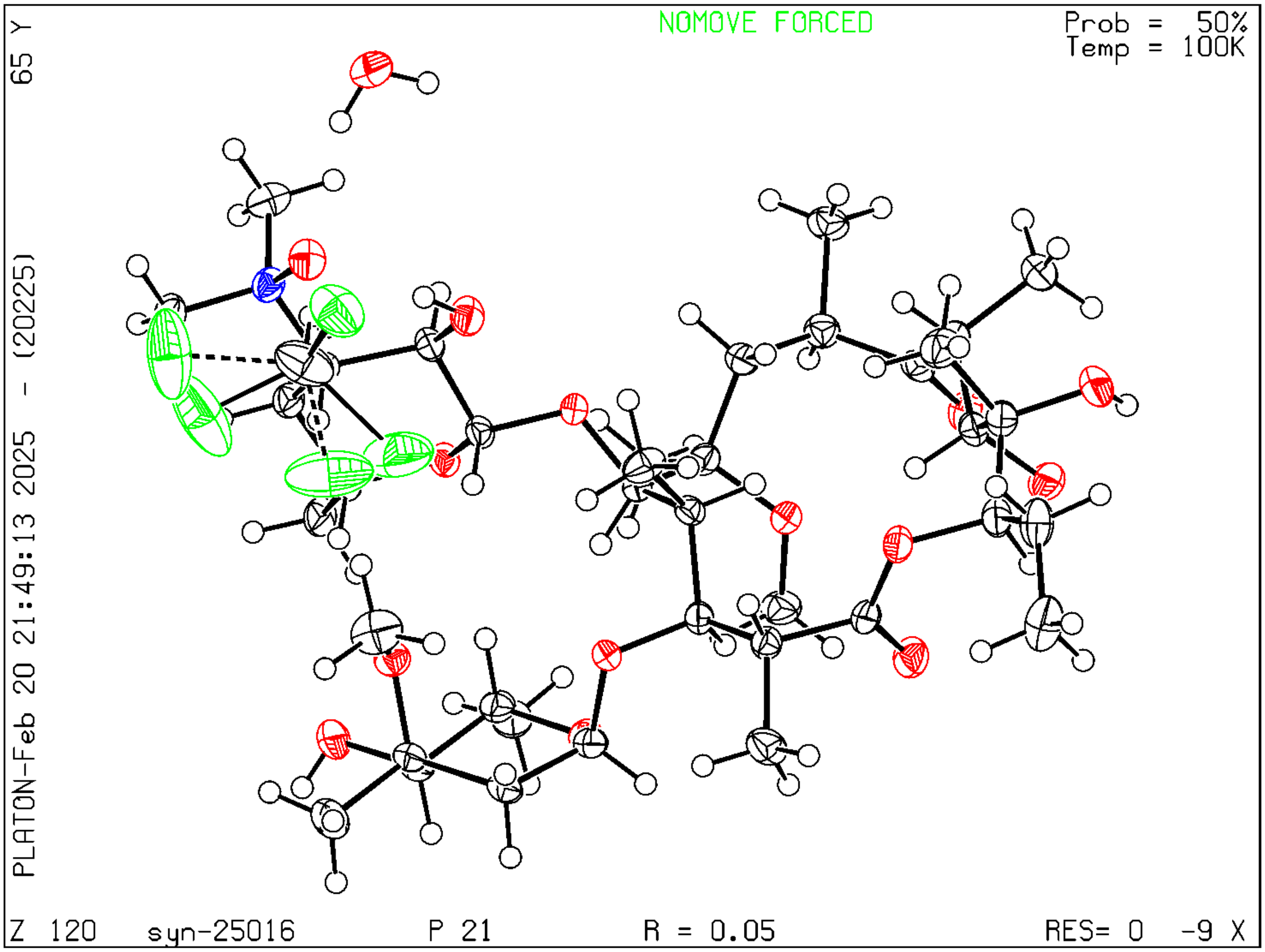

Download CIF editor (publCIF) from the IUCr
Download CIF editor (enCIFer) from the CCDC
Test a new CIF entry
