# Supplementary material for: Site-Divergent Oxidations within Venerable Macrolide Antibiotic Scaffolds Unveil Compounds with Broad Spectrum and Anti-MRSA Activities
Source: ACS Cent Sci. 2026 Mar 17;12(3):375–82. doi: 10.1021/acscentsci.5c02343 (PMC13022725; doi:10.1021/acscentsci.5c02343)
Supplement: Supplementary file 5 [file oc5c02343_si_005.zip › Biological, Computational, and X-ray Data/X-Ray/7/syn-25016.docx]

***Experimental***

Low-temperature diffraction data (ω-scans) were collected on a Rigaku Synergy-S diffractometer coupled to a HyPix-Arc 100 detector with Cu Kα (λ = 1.54178 Å) for the structure of syn-25016. The diffraction images were processed and scaled using Rigaku Oxford Diffraction software (CrysAlisPro; Rigaku OD: The Woodlands, TX, 2015). The structure was solved with SHELXT and was refined against F^2^ on all data by full-matrix least squares with SHELXL (Sheldrick, G. M. Acta Cryst. 2008, A64, 112–122). All non-hydrogen atoms were refined anisotropically. Hydrogen atoms were included in the model at geometrically calculated positions and refined using a riding model. The isotropic displacement parameters of all hydrogen atoms were fixed to 1.2 times the U value of the atoms to which they are linked (1.5 times for methyl groups). The full numbering scheme of compound syn-25016 can be found in the full details of the X-ray structure determination (CIF), which is included as Supporting Information. CCDC number XXXXXX (syn-25016) contains the supplementary crystallographic data for this paper. These data can be obtained free of charge from The Cambridge Crystallographic Data Center via www.ccdc.cam.ac.uk/data_request/cif.


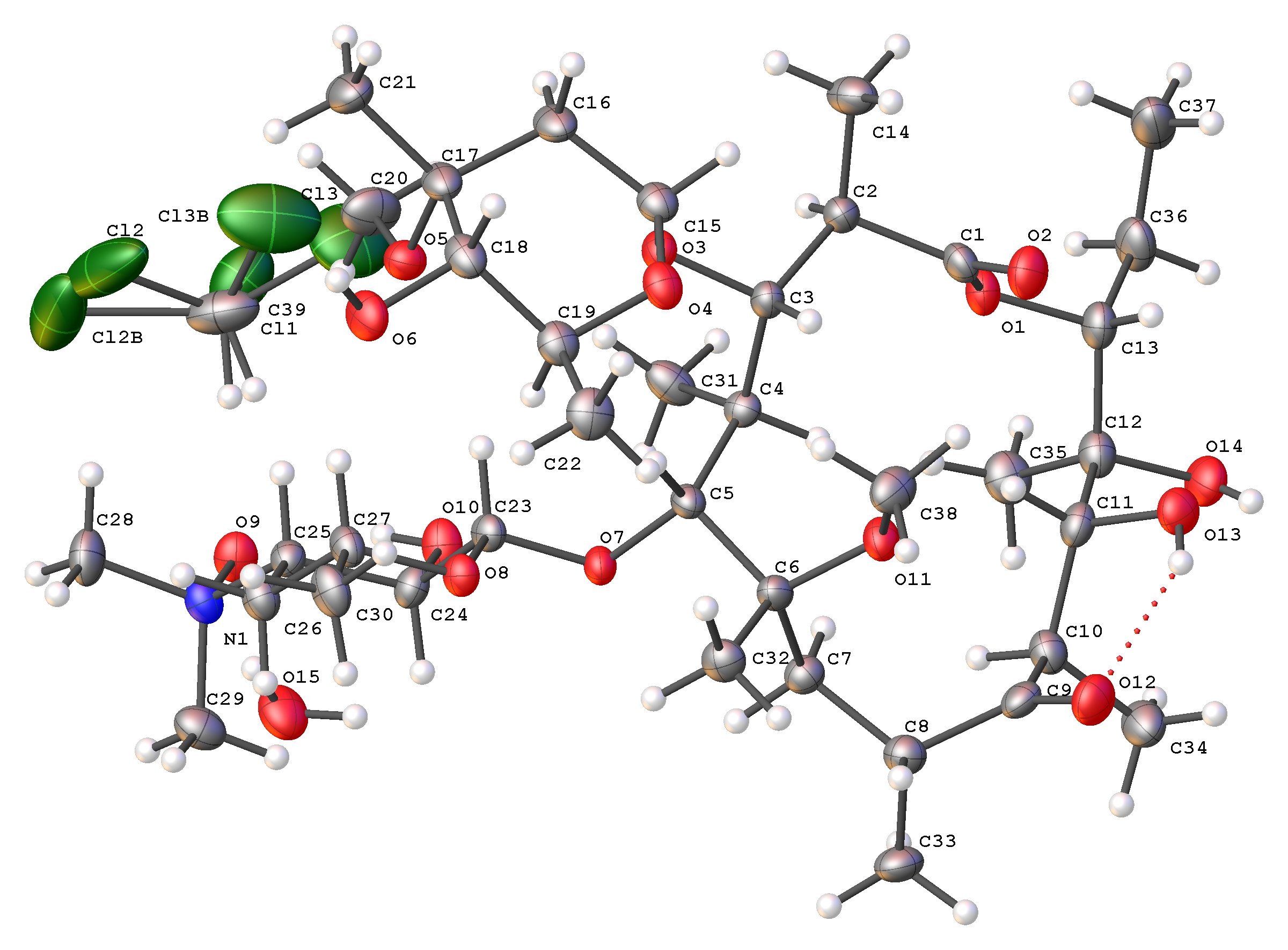


Figure 1. The complete numbering scheme of syn-25016 with 50% thermal ellipsoid probability levels. The hydrogen atoms are shown as circles for clarity.

Table 1. Crystal data and structure refinement for syn-25016.

Identification code syn-25016

Empirical formula C39 H72 Cl3 N O15

Formula weight 901.32

Temperature 100(2) K

Wavelength 1.54184 Å

Crystal system Monoclinic

Space group P2**_1_**

Unit cell dimensions a = 9.09480(10) Å α= 90°.

b = 26.4164(4) Å β= 92.9300(10)°.

c = 9.63000(10) Å γ = 90°.

Volume 2310.60(5) Å3

Z 2

Density (calculated) 1.295 Mg/m3

Absorption coefficient 2.338 mm-1

F(000) 968

Crystal size 0.200 x 0.200 x 0.050 mm3

Crystal color and habit colorless plate

Diffractometer XtaLAB Synergy, Dualflex, HyPix-Arc 100

Theta range for data collection 3.346 to 66.982°.

Index ranges -10<=h<=10, -30<=k<=31, -11<=l<=11

Reflections collected 32626

Independent reflections 7884 [R(int) = 0.0969]

Observed reflections (I > 2sigma(I)) 7463

Completeness to theta = 66.982° 99.1 %

Absorption correction Semi-empirical from equivalents

Max. and min. transmission 1.00000 and 0.85701

Solution method SHELXT-2014/5 (Sheldrick, 2014)

Refinement method SHELXL-2014/7 (Sheldrick, 2014)

Data / restraints / parameters 7884 / 13 / 563

Goodness-of-fit on F2 1.107

Final R indices [I>2sigma(I)] R1 = 0.0540, wR2 = 0.1502

R indices (all data) R1 = 0.0560, wR2 = 0.1527

Absolute structure parameter 0.032(17)

Extinction coefficient n/a

Largest diff. peak and hole 0.587 and -0.412 e.Å-3

Table 2. Atomic coordinates ( x 104) and equivalent isotropic displacement parameters (Å2x 103)

for syn-25016. U(eq) is defined as one third of the trace of the orthogonalized Uij tensor.

________________________________________________________________________________

x y z U(eq)

________________________________________________________________________________

Cl(1) 12119(2) 3428(1) 9002(1) 58(1)

Cl(2) 11375(5) 2915(4) 6355(9) 102(3)

Cl(2B) 11396(7) 2640(3) 7070(14) 87(3)

Cl(3) 10881(6) 3977(2) 6730(5) 82(2)

Cl(3B) 11235(8) 3672(5) 6186(8) 90(4)

C(39) 10929(6) 3330(3) 7535(6) 53(2)

O(1) 7523(3) 6598(1) 6975(3) 25(1)

O(2) 6436(4) 6850(1) 4934(3) 32(1)

O(3) 7660(3) 5334(1) 3668(3) 22(1)

O(4) 6039(4) 5338(1) 1680(3) 26(1)

O(5) 7970(3) 4262(1) 2470(3) 26(1)

O(6) 6074(4) 4069(1) 149(3) 32(1)

O(7) 4924(3) 4637(1) 5962(3) 21(1)

O(8) 4436(3) 4064(1) 4246(3) 22(1)

O(9) 7640(3) 3068(1) 7716(3) 29(1)

O(10) 6730(4) 3964(1) 7455(3) 30(1)

O(11) 3517(3) 5883(1) 5034(3) 25(1)

O(12) 1379(4) 6547(1) 6783(3) 34(1)

O(13) 3828(3) 7142(1) 7244(3) 30(1)

O(14) 5454(4) 7256(1) 9617(3) 33(1)

N(1) 6593(4) 2893(1) 6681(4) 24(1)

C(1) 7243(5) 6566(2) 5599(4) 23(1)

C(2) 8073(4) 6131(2) 4972(4) 23(1)

C(3) 6963(4) 5716(2) 4479(4) 21(1)

C(4) 6289(4) 5448(2) 5726(4) 20(1)

C(5) 4977(4) 5107(2) 5202(4) 20(1)

C(6) 3451(4) 5356(2) 5386(4) 21(1)

C(7) 3085(5) 5349(2) 6933(4) 23(1)

C(8) 1729(5) 5667(2) 7257(4) 26(1)

C(9) 2075(5) 6227(2) 7451(4) 25(1)

C(10) 3234(5) 6386(2) 8565(5) 26(1)

C(11) 4423(5) 6686(2) 7834(4) 25(1)

C(12) 5802(5) 6845(2) 8727(4) 28(1)

C(13) 6981(5) 7035(2) 7733(5) 27(1)

C(14) 8995(5) 6350(2) 3829(5) 33(1)

C(15) 7493(5) 5414(2) 2213(4) 25(1)

C(16) 8623(5) 5092(2) 1506(5) 30(1)

C(17) 8164(5) 4541(2) 1209(4) 27(1)

C(18) 6635(5) 4559(2) 484(4) 27(1)

C(19) 5550(5) 4824(2) 1389(4) 26(1)

C(20) 9260(5) 4164(2) 3314(6) 42(1)

C(21) 9257(6) 4279(2) 285(5) 38(1)

C(22) 4038(5) 4883(2) 662(5) 32(1)

C(23) 5488(4) 4217(2) 5301(4) 21(1)

C(24) 5701(5) 3801(2) 6386(4) 22(1)

C(25) 6232(5) 3325(2) 5660(4) 21(1)

C(26) 5142(5) 3178(2) 4490(4) 24(1)

C(27) 4908(5) 3630(2) 3490(4) 23(1)

C(28) 7302(6) 2467(2) 5953(5) 34(1)

C(29) 5285(5) 2702(2) 7381(5) 33(1)

C(30) 3738(5) 3522(2) 2374(4) 28(1)

C(31) 7459(5) 5160(2) 6606(5) 30(1)

C(32) 2268(5) 5066(2) 4516(4) 27(1)

C(33) 1018(5) 5478(2) 8580(5) 30(1)

C(34) 2490(6) 6677(2) 9726(5) 35(1)

C(35) 6400(6) 6418(2) 9653(5) 35(1)

C(36) 8326(6) 7282(2) 8444(5) 36(1)

C(37) 9320(6) 7532(2) 7423(7) 44(1)

C(38) 3320(5) 6021(2) 3598(5) 32(1)

O(15) 6950(5) 3018(1) 10609(4) 41(1)

________________________________________________________________________________ Table 3. Bond lengths [Å] and angles [°] for syn-25016.

_____________________________________________________

Cl(1)-C(39) 1.755(6)

Cl(2)-C(39) 1.644(8)

Cl(2B)-C(39) 1.930(10)

Cl(3)-C(39) 1.876(9)

Cl(3B)-C(39) 1.618(8)

C(39)-H(39) 1.0000

C(39)-H(39A) 1.0000

O(1)-C(1) 1.340(5)

O(1)-C(13) 1.466(5)

O(2)-C(1) 1.210(5)

O(3)-C(15) 1.418(5)

O(3)-C(3) 1.442(5)

O(4)-C(15) 1.409(5)

O(4)-C(19) 1.450(5)

O(5)-C(20) 1.416(6)

O(5)-C(17) 1.440(5)

O(6)-C(18) 1.423(5)

O(6)-H(6) 0.8400

O(7)-C(23) 1.391(5)

O(7)-C(5) 1.444(5)

O(8)-C(23) 1.418(5)

O(8)-C(27) 1.434(5)

O(9)-N(1) 1.420(5)

O(10)-C(24) 1.422(5)

O(10)-H(10) 0.8400

O(11)-C(38) 1.433(5)

O(11)-C(6) 1.435(5)

O(12)-C(9) 1.218(5)

O(13)-C(11) 1.425(5)

O(13)-H(13) 0.8400

O(14)-C(12) 1.429(5)

O(14)-H(14) 0.8400

N(1)-C(29) 1.486(6)

N(1)-C(28) 1.490(6)

N(1)-C(25) 1.530(5)

C(1)-C(2) 1.517(6)

C(2)-C(14) 1.530(6)

C(2)-C(3) 1.549(5)

C(2)-H(2) 1.0000

C(3)-C(4) 1.547(5)

C(3)-H(3) 1.0000

C(4)-C(31) 1.529(6)

C(4)-C(5) 1.558(5)

C(4)-H(4) 1.0000

C(5)-C(6) 1.554(5)

C(5)-H(5) 1.0000

C(6)-C(32) 1.534(6)

C(6)-C(7) 1.543(5)

C(7)-C(8) 1.538(6)

C(7)-H(7A) 0.9900

C(7)-H(7B) 0.9900

C(8)-C(9) 1.523(6)

C(8)-C(33) 1.541(6)

C(8)-H(8) 1.0000

C(9)-C(10) 1.524(6)

C(10)-C(11) 1.541(6)

C(10)-C(34) 1.542(6)

C(10)-H(10A) 1.0000

C(11)-C(12) 1.543(6)

C(11)-H(11) 1.0000

C(12)-C(35) 1.521(6)

C(12)-C(13) 1.557(6)

C(13)-C(36) 1.518(6)

C(13)-H(13A) 1.0000

C(14)-H(14A) 0.9800

C(14)-H(14B) 0.9800

C(14)-H(14C) 0.9800

C(15)-C(16) 1.520(6)

C(15)-H(15) 1.0000

C(16)-C(17) 1.538(6)

C(16)-H(16A) 0.9900

C(16)-H(16B) 0.9900

C(17)-C(18) 1.524(6)

C(17)-C(21) 1.534(6)

C(18)-C(19) 1.521(6)

C(18)-H(18) 1.0000

C(19)-C(22) 1.519(6)

C(19)-H(19) 1.0000

C(20)-H(20A) 0.9800

C(20)-H(20B) 0.9800

C(20)-H(20C) 0.9800

C(21)-H(21A) 0.9800

C(21)-H(21B) 0.9800

C(21)-H(21C) 0.9800

C(22)-H(22A) 0.9800

C(22)-H(22B) 0.9800

C(22)-H(22C) 0.9800

C(23)-C(24) 1.521(6)

C(23)-H(23) 1.0000

C(24)-C(25) 1.530(5)

C(24)-H(24) 1.0000

C(25)-C(26) 1.512(6)

C(25)-H(25) 1.0000

C(26)-C(27) 1.542(6)

C(26)-H(26A) 0.9900

C(26)-H(26B) 0.9900

C(27)-C(30) 1.501(6)

C(27)-H(27) 1.0000

C(28)-H(28A) 0.9800

C(28)-H(28B) 0.9800

C(28)-H(28C) 0.9800

C(29)-H(29A) 0.9800

C(29)-H(29B) 0.9800

C(29)-H(29C) 0.9800

C(30)-H(30A) 0.9800

C(30)-H(30B) 0.9800

C(30)-H(30C) 0.9800

C(31)-H(31A) 0.9800

C(31)-H(31B) 0.9800

C(31)-H(31C) 0.9800

C(32)-H(32A) 0.9800

C(32)-H(32B) 0.9800

C(32)-H(32C) 0.9800

C(33)-H(33A) 0.9800

C(33)-H(33B) 0.9800

C(33)-H(33C) 0.9800

C(34)-H(34A) 0.9800

C(34)-H(34B) 0.9800

C(34)-H(34C) 0.9800

C(35)-H(35A) 0.9800

C(35)-H(35B) 0.9800

C(35)-H(35C) 0.9800

C(36)-C(37) 1.520(8)

C(36)-H(36A) 0.9900

C(36)-H(36B) 0.9900

C(37)-H(37A) 0.9800

C(37)-H(37B) 0.9800

C(37)-H(37C) 0.9800

C(38)-H(38A) 0.9800

C(38)-H(38B) 0.9800

C(38)-H(38C) 0.9800

O(15)-H(15A) 0.8702

O(15)-H(15B) 0.8700

Cl(3B)-C(39)-Cl(1) 116.1(4)

Cl(2)-C(39)-Cl(1) 119.4(5)

Cl(2)-C(39)-Cl(3) 108.8(5)

Cl(1)-C(39)-Cl(3) 101.5(4)

Cl(3B)-C(39)-Cl(2B) 107.0(6)

Cl(1)-C(39)-Cl(2B) 101.1(4)

Cl(3B)-C(39)-H(39) 110.7

Cl(1)-C(39)-H(39) 110.7

Cl(2B)-C(39)-H(39) 110.7

Cl(2)-C(39)-H(39A) 108.9

Cl(1)-C(39)-H(39A) 108.9

Cl(3)-C(39)-H(39A) 108.9

C(1)-O(1)-C(13) 119.4(3)

C(15)-O(3)-C(3) 113.8(3)

C(15)-O(4)-C(19) 118.5(3)

C(20)-O(5)-C(17) 116.4(3)

C(18)-O(6)-H(6) 109.5

C(23)-O(7)-C(5) 115.5(3)

C(23)-O(8)-C(27) 112.5(3)

C(24)-O(10)-H(10) 109.5

C(38)-O(11)-C(6) 118.1(3)

C(11)-O(13)-H(13) 109.5

C(12)-O(14)-H(14) 109.5

O(9)-N(1)-C(29) 108.4(3)

O(9)-N(1)-C(28) 106.6(3)

C(29)-N(1)-C(28) 109.4(4)

O(9)-N(1)-C(25) 108.9(3)

C(29)-N(1)-C(25) 113.2(3)

C(28)-N(1)-C(25) 110.1(3)

O(2)-C(1)-O(1) 124.3(4)

O(2)-C(1)-C(2) 124.0(4)

O(1)-C(1)-C(2) 111.6(3)

C(1)-C(2)-C(14) 107.6(3)

C(1)-C(2)-C(3) 109.1(3)

C(14)-C(2)-C(3) 114.8(3)

C(1)-C(2)-H(2) 108.4

C(14)-C(2)-H(2) 108.4

C(3)-C(2)-H(2) 108.4

O(3)-C(3)-C(4) 107.6(3)

O(3)-C(3)-C(2) 111.3(3)

C(4)-C(3)-C(2) 111.4(3)

O(3)-C(3)-H(3) 108.8

C(4)-C(3)-H(3) 108.8

C(2)-C(3)-H(3) 108.8

C(31)-C(4)-C(3) 111.5(3)

C(31)-C(4)-C(5) 113.0(3)

C(3)-C(4)-C(5) 109.9(3)

C(31)-C(4)-H(4) 107.4

C(3)-C(4)-H(4) 107.4

C(5)-C(4)-H(4) 107.4

O(7)-C(5)-C(6) 104.6(3)

O(7)-C(5)-C(4) 112.2(3)

C(6)-C(5)-C(4) 113.1(3)

O(7)-C(5)-H(5) 108.9

C(6)-C(5)-H(5) 108.9

C(4)-C(5)-H(5) 108.9

O(11)-C(6)-C(32) 113.1(3)

O(11)-C(6)-C(7) 104.7(3)

C(32)-C(6)-C(7) 110.0(3)

O(11)-C(6)-C(5) 109.6(3)

C(32)-C(6)-C(5) 109.4(3)

C(7)-C(6)-C(5) 110.1(3)

C(8)-C(7)-C(6) 113.9(3)

C(8)-C(7)-H(7A) 108.8

C(6)-C(7)-H(7A) 108.8

C(8)-C(7)-H(7B) 108.8

C(6)-C(7)-H(7B) 108.8

H(7A)-C(7)-H(7B) 107.7

C(9)-C(8)-C(7) 113.2(3)

C(9)-C(8)-C(33) 107.8(4)

C(7)-C(8)-C(33) 111.4(4)

C(9)-C(8)-H(8) 108.1

C(7)-C(8)-H(8) 108.1

C(33)-C(8)-H(8) 108.1

O(12)-C(9)-C(8) 120.7(4)

O(12)-C(9)-C(10) 120.2(4)

C(8)-C(9)-C(10) 119.0(4)

C(9)-C(10)-C(11) 107.2(3)

C(9)-C(10)-C(34) 109.5(4)

C(11)-C(10)-C(34) 114.7(4)

C(9)-C(10)-H(10A) 108.4

C(11)-C(10)-H(10A) 108.4

C(34)-C(10)-H(10A) 108.4

O(13)-C(11)-C(10) 110.9(3)

O(13)-C(11)-C(12) 105.9(3)

C(10)-C(11)-C(12) 117.0(3)

O(13)-C(11)-H(11) 107.5

C(10)-C(11)-H(11) 107.5

C(12)-C(11)-H(11) 107.5

O(14)-C(12)-C(35) 107.2(3)

O(14)-C(12)-C(11) 110.0(4)

C(35)-C(12)-C(11) 112.5(4)

O(14)-C(12)-C(13) 107.7(3)

C(35)-C(12)-C(13) 111.3(4)

C(11)-C(12)-C(13) 108.1(3)

O(1)-C(13)-C(36) 106.2(4)

O(1)-C(13)-C(12) 108.1(3)

C(36)-C(13)-C(12) 115.2(4)

O(1)-C(13)-H(13A) 109.1

C(36)-C(13)-H(13A) 109.1

C(12)-C(13)-H(13A) 109.1

C(2)-C(14)-H(14A) 109.5

C(2)-C(14)-H(14B) 109.5

H(14A)-C(14)-H(14B) 109.5

C(2)-C(14)-H(14C) 109.5

H(14A)-C(14)-H(14C) 109.5

H(14B)-C(14)-H(14C) 109.5

O(4)-C(15)-O(3) 113.0(3)

O(4)-C(15)-C(16) 113.6(4)

O(3)-C(15)-C(16) 108.6(4)

O(4)-C(15)-H(15) 107.1

O(3)-C(15)-H(15) 107.1

C(16)-C(15)-H(15) 107.1

C(15)-C(16)-C(17) 115.4(4)

C(15)-C(16)-H(16A) 108.4

C(17)-C(16)-H(16A) 108.4

C(15)-C(16)-H(16B) 108.4

C(17)-C(16)-H(16B) 108.4

H(16A)-C(16)-H(16B) 107.5

O(5)-C(17)-C(18) 104.8(3)

O(5)-C(17)-C(21) 111.6(4)

C(18)-C(17)-C(21) 110.6(4)

O(5)-C(17)-C(16) 111.9(3)

C(18)-C(17)-C(16) 106.7(4)

C(21)-C(17)-C(16) 111.0(4)

O(6)-C(18)-C(19) 108.2(3)

O(6)-C(18)-C(17) 112.5(4)

C(19)-C(18)-C(17) 110.8(3)

O(6)-C(18)-H(18) 108.4

C(19)-C(18)-H(18) 108.4

C(17)-C(18)-H(18) 108.4

O(4)-C(19)-C(22) 104.8(3)

O(4)-C(19)-C(18) 109.9(3)

C(22)-C(19)-C(18) 112.4(4)

O(4)-C(19)-H(19) 109.9

C(22)-C(19)-H(19) 109.9

C(18)-C(19)-H(19) 109.9

O(5)-C(20)-H(20A) 109.5

O(5)-C(20)-H(20B) 109.5

H(20A)-C(20)-H(20B) 109.5

O(5)-C(20)-H(20C) 109.5

H(20A)-C(20)-H(20C) 109.5

H(20B)-C(20)-H(20C) 109.5

C(17)-C(21)-H(21A) 109.5

C(17)-C(21)-H(21B) 109.5

H(21A)-C(21)-H(21B) 109.5

C(17)-C(21)-H(21C) 109.5

H(21A)-C(21)-H(21C) 109.5

H(21B)-C(21)-H(21C) 109.5

C(19)-C(22)-H(22A) 109.5

C(19)-C(22)-H(22B) 109.5

H(22A)-C(22)-H(22B) 109.5

C(19)-C(22)-H(22C) 109.5

H(22A)-C(22)-H(22C) 109.5

H(22B)-C(22)-H(22C) 109.5

O(7)-C(23)-O(8) 107.7(3)

O(7)-C(23)-C(24) 107.3(3)

O(8)-C(23)-C(24) 110.1(3)

O(7)-C(23)-H(23) 110.5

O(8)-C(23)-H(23) 110.5

C(24)-C(23)-H(23) 110.5

O(10)-C(24)-C(23) 109.6(3)

O(10)-C(24)-C(25) 111.6(3)

C(23)-C(24)-C(25) 108.1(3)

O(10)-C(24)-H(24) 109.2

C(23)-C(24)-H(24) 109.2

C(25)-C(24)-H(24) 109.2

C(26)-C(25)-C(24) 110.0(3)

C(26)-C(25)-N(1) 113.5(3)

C(24)-C(25)-N(1) 112.4(3)

C(26)-C(25)-H(25) 106.8

C(24)-C(25)-H(25) 106.8

N(1)-C(25)-H(25) 106.8

C(25)-C(26)-C(27) 109.3(3)

C(25)-C(26)-H(26A) 109.8

C(27)-C(26)-H(26A) 109.8

C(25)-C(26)-H(26B) 109.8

C(27)-C(26)-H(26B) 109.8

H(26A)-C(26)-H(26B) 108.3

O(8)-C(27)-C(30) 107.2(3)

O(8)-C(27)-C(26) 109.7(3)

C(30)-C(27)-C(26) 111.7(3)

O(8)-C(27)-H(27) 109.4

C(30)-C(27)-H(27) 109.4

C(26)-C(27)-H(27) 109.4

N(1)-C(28)-H(28A) 109.5

N(1)-C(28)-H(28B) 109.5

H(28A)-C(28)-H(28B) 109.5

N(1)-C(28)-H(28C) 109.5

H(28A)-C(28)-H(28C) 109.5

H(28B)-C(28)-H(28C) 109.5

N(1)-C(29)-H(29A) 109.5

N(1)-C(29)-H(29B) 109.5

H(29A)-C(29)-H(29B) 109.5

N(1)-C(29)-H(29C) 109.5

H(29A)-C(29)-H(29C) 109.5

H(29B)-C(29)-H(29C) 109.5

C(27)-C(30)-H(30A) 109.5

C(27)-C(30)-H(30B) 109.5

H(30A)-C(30)-H(30B) 109.5

C(27)-C(30)-H(30C) 109.5

H(30A)-C(30)-H(30C) 109.5

H(30B)-C(30)-H(30C) 109.5

C(4)-C(31)-H(31A) 109.5

C(4)-C(31)-H(31B) 109.5

H(31A)-C(31)-H(31B) 109.5

C(4)-C(31)-H(31C) 109.5

H(31A)-C(31)-H(31C) 109.5

H(31B)-C(31)-H(31C) 109.5

C(6)-C(32)-H(32A) 109.5

C(6)-C(32)-H(32B) 109.5

H(32A)-C(32)-H(32B) 109.5

C(6)-C(32)-H(32C) 109.5

H(32A)-C(32)-H(32C) 109.5

H(32B)-C(32)-H(32C) 109.5

C(8)-C(33)-H(33A) 109.5

C(8)-C(33)-H(33B) 109.5

H(33A)-C(33)-H(33B) 109.5

C(8)-C(33)-H(33C) 109.5

H(33A)-C(33)-H(33C) 109.5

H(33B)-C(33)-H(33C) 109.5

C(10)-C(34)-H(34A) 109.5

C(10)-C(34)-H(34B) 109.5

H(34A)-C(34)-H(34B) 109.5

C(10)-C(34)-H(34C) 109.5

H(34A)-C(34)-H(34C) 109.5

H(34B)-C(34)-H(34C) 109.5

C(12)-C(35)-H(35A) 109.5

C(12)-C(35)-H(35B) 109.5

H(35A)-C(35)-H(35B) 109.5

C(12)-C(35)-H(35C) 109.5

H(35A)-C(35)-H(35C) 109.5

H(35B)-C(35)-H(35C) 109.5

C(13)-C(36)-C(37) 112.7(4)

C(13)-C(36)-H(36A) 109.0

C(37)-C(36)-H(36A) 109.0

C(13)-C(36)-H(36B) 109.0

C(37)-C(36)-H(36B) 109.0

H(36A)-C(36)-H(36B) 107.8

C(36)-C(37)-H(37A) 109.5

C(36)-C(37)-H(37B) 109.5

H(37A)-C(37)-H(37B) 109.5

C(36)-C(37)-H(37C) 109.5

H(37A)-C(37)-H(37C) 109.5

H(37B)-C(37)-H(37C) 109.5

O(11)-C(38)-H(38A) 109.5

O(11)-C(38)-H(38B) 109.5

H(38A)-C(38)-H(38B) 109.5

O(11)-C(38)-H(38C) 109.5

H(38A)-C(38)-H(38C) 109.5

H(38B)-C(38)-H(38C) 109.5

H(15A)-O(15)-H(15B) 104.5

_____________________________________________________________

Symmetry transformations used to generate equivalent atoms:

Table 4. Anisotropic displacement parameters (Å2x 103) for syn-25016. The anisotropic

displacement factor exponent takes the form: -2π2[ h2 a*2U11 + ... + 2 h k a* b* U12 ]

______________________________________________________________________________

U11 U22 U33 U23 U13 U12

______________________________________________________________________________

Cl(1) 58(1) 74(1) 41(1) -2(1) -4(1) 20(1)

Cl(2) 55(2) 125(6) 129(5) -85(4) 40(3) -30(3)

Cl(2B) 40(2) 66(4) 154(8) -58(5) -8(3) -1(2)

Cl(3) 102(3) 91(3) 54(2) 32(2) 7(2) 1(2)

Cl(3B) 74(3) 142(9) 52(4) 47(5) -16(3) -13(4)

C(39) 34(3) 81(4) 43(3) -8(3) 10(2) -12(3)

O(1) 31(2) 18(1) 25(1) -2(1) -2(1) 3(1)

O(2) 37(2) 27(2) 32(2) 2(1) -2(1) 9(1)

O(3) 28(1) 19(1) 19(1) -2(1) 4(1) 1(1)

O(4) 36(2) 22(1) 21(1) 2(1) -1(1) 0(1)

O(5) 25(1) 26(2) 27(2) 1(1) 2(1) -1(1)

O(6) 43(2) 27(2) 27(2) -9(1) 3(1) -4(1)

O(7) 26(1) 16(1) 21(1) -1(1) 4(1) 1(1)

O(8) 25(1) 21(1) 20(1) -3(1) -2(1) 0(1)

O(9) 30(2) 30(2) 27(2) -2(1) -7(1) 2(1)

O(10) 40(2) 26(2) 22(1) -3(1) -8(1) 4(1)

O(11) 34(2) 20(1) 23(1) 2(1) 4(1) 3(1)

O(12) 33(2) 31(2) 38(2) 4(1) 0(1) 10(1)

O(13) 31(2) 26(2) 31(2) 5(1) -1(1) 5(1)

O(14) 40(2) 30(2) 30(2) -9(1) 5(1) 1(1)

N(1) 28(2) 21(2) 23(2) 0(1) -3(1) -1(1)

C(1) 28(2) 17(2) 25(2) -2(2) 2(2) -4(2)

C(2) 22(2) 20(2) 26(2) -1(2) 4(2) -1(2)

C(3) 21(2) 18(2) 22(2) -2(2) 2(2) 0(2)

C(4) 22(2) 22(2) 16(2) -2(1) 2(2) -1(2)

C(5) 24(2) 18(2) 19(2) 0(2) 5(2) -2(2)

C(6) 19(2) 20(2) 24(2) 1(2) 2(2) 0(2)

C(7) 24(2) 25(2) 21(2) 0(2) 2(2) 1(2)

C(8) 26(2) 27(2) 26(2) 0(2) 3(2) -2(2)

C(9) 23(2) 28(2) 23(2) 0(2) 8(2) 5(2)

C(10) 33(2) 21(2) 25(2) 1(2) 2(2) 0(2)

C(11) 31(2) 22(2) 23(2) -2(2) 4(2) 7(2)

C(12) 36(2) 22(2) 25(2) -1(2) 1(2) 3(2)

C(13) 32(2) 21(2) 29(2) -5(2) 1(2) 3(2)

C(14) 31(2) 37(3) 32(2) -6(2) 9(2) -10(2)

C(15) 31(2) 24(2) 20(2) 1(2) 4(2) -3(2)

C(16) 37(2) 28(2) 25(2) -2(2) 15(2) -6(2)

C(17) 35(2) 24(2) 23(2) -1(2) 12(2) -3(2)

C(18) 40(2) 24(2) 18(2) -1(2) 5(2) -4(2)

C(19) 32(2) 25(2) 20(2) 2(2) -1(2) 0(2)

C(20) 26(2) 52(3) 47(3) 12(2) -3(2) 4(2)

C(21) 37(2) 36(3) 41(3) -8(2) 18(2) -3(2)

C(22) 37(2) 35(2) 23(2) 2(2) -6(2) 4(2)

C(23) 23(2) 19(2) 20(2) -5(2) 2(2) -2(2)

C(24) 25(2) 22(2) 20(2) -3(2) 1(2) 4(2)

C(25) 25(2) 19(2) 19(2) -1(2) 4(2) 1(2)

C(26) 30(2) 21(2) 22(2) -2(2) -2(2) -1(2)

C(27) 32(2) 18(2) 20(2) -2(2) 3(2) -2(2)

C(28) 47(3) 23(2) 32(2) 1(2) -1(2) 11(2)

C(29) 35(2) 34(2) 31(2) 8(2) 1(2) -6(2)

C(30) 40(2) 22(2) 23(2) -5(2) -6(2) -4(2)

C(31) 33(2) 30(2) 27(2) 7(2) -6(2) -9(2)

C(32) 26(2) 29(2) 26(2) -3(2) -1(2) -2(2)

C(33) 25(2) 37(2) 31(2) 3(2) 10(2) -1(2)

C(34) 46(3) 30(2) 31(2) -5(2) 13(2) -3(2)

C(35) 41(3) 33(2) 30(2) 4(2) -4(2) 2(2)

C(36) 36(2) 26(2) 45(3) -11(2) -5(2) -2(2)

C(37) 32(2) 30(2) 71(4) -5(2) 1(2) 0(2)

C(38) 34(2) 34(2) 27(2) 9(2) 3(2) 9(2)

O(15) 58(2) 32(2) 32(2) 7(1) 6(2) -2(2)

______________________________________________________________________________ Table 5. Hydrogen coordinates ( x 104) and isotropic displacement parameters (Å2x 10 3)

for syn-25016.

________________________________________________________________________________

x y z U(eq)

________________________________________________________________________________

H(39) 9878 3359 7783 63

H(39A) 9926 3246 7849 63

H(6) 6216 4004 -687 49

H(10) 7278 3721 7703 45

H(13) 2955 7092 6950 44

H(14) 4768 7428 9239 50

H(2) 8758 5984 5711 27

H(3) 6152 5877 3893 25

H(4) 5883 5717 6329 24

H(5) 5079 5030 4195 24

H(7A) 2913 4994 7216 28

H(7B) 3946 5477 7497 28

H(8) 987 5632 6460 31

H(10A) 3698 6073 8978 32

H(11) 4759 6473 7053 30

H(13A) 6510 7280 7053 33

H(14A) 9533 6076 3392 49

H(14B) 9696 6597 4236 49

H(14C) 8345 6517 3127 49

H(15) 7744 5776 2039 30

H(16A) 9545 5090 2099 35

H(16B) 8843 5256 615 35

H(18) 6698 4756 -398 33

H(19) 5466 4634 2280 31

H(20A) 9979 3987 2766 62

H(20B) 9012 3952 4104 62

H(20C) 9680 4485 3657 62

H(21A) 9183 4428 -648 56

H(21B) 9027 3917 223 56

H(21C) 10260 4323 688 56

H(22A) 3368 5043 1296 48

H(22B) 3656 4549 387 48

H(22C) 4114 5095 -167 48

H(23) 6446 4302 4891 25

H(24) 4737 3729 6800 27

H(25) 7170 3416 5223 25

H(26A) 4193 3081 4874 29

H(26B) 5520 2884 3981 29

H(27) 5855 3710 3052 28

H(28A) 8112 2598 5424 51

H(28B) 6573 2303 5318 51

H(28C) 7686 2221 6640 51

H(29A) 5590 2438 8050 49

H(29B) 4575 2561 6685 49

H(29C) 4825 2981 7870 49

H(30A) 3656 3810 1731 43

H(30B) 2793 3470 2798 43

H(30C) 4001 3217 1863 43

H(31A) 8040 4949 6001 45

H(31B) 6978 4944 7276 45

H(31C) 8109 5402 7107 45

H(32A) 1306 5224 4625 41

H(32B) 2240 4713 4832 41

H(32C) 2503 5074 3534 41

H(33A) 1745 5490 9367 45

H(33B) 677 5129 8439 45

H(33C) 179 5695 8776 45

H(34A) 3213 6743 10494 53

H(34B) 1680 6474 10065 53

H(34C) 2104 6999 9357 53

H(35A) 6611 6123 9079 52

H(35B) 5668 6326 10321 52

H(35C) 7307 6529 10155 52

H(36A) 8896 7022 8981 43

H(36B) 8001 7540 9108 43

H(37A) 9690 7275 6794 67

H(37B) 10151 7694 7935 67

H(37C) 8760 7787 6881 67

H(38A) 3870 5786 3032 47

H(38B) 3686 6366 3469 47

H(38C) 2272 6006 3311 47

H(15A) 6564 3319 10589 61

H(15B) 7198 2965 9761 61

________________________________________________________________________________ Table 6. Torsion angles [°] for syn-25016.

________________________________________________________________

C(13)-O(1)-C(1)-O(2) -7.8(6)

C(13)-O(1)-C(1)-C(2) 171.7(3)

O(2)-C(1)-C(2)-C(14) 55.7(5)

O(1)-C(1)-C(2)-C(14) -123.8(4)

O(2)-C(1)-C(2)-C(3) -69.5(5)

O(1)-C(1)-C(2)-C(3) 111.0(4)

C(15)-O(3)-C(3)-C(4) 141.9(3)

C(15)-O(3)-C(3)-C(2) -95.8(4)

C(1)-C(2)-C(3)-O(3) 170.3(3)

C(14)-C(2)-C(3)-O(3) 49.5(5)

C(1)-C(2)-C(3)-C(4) -69.6(4)

C(14)-C(2)-C(3)-C(4) 169.6(3)

O(3)-C(3)-C(4)-C(31) 57.9(4)

C(2)-C(3)-C(4)-C(31) -64.4(4)

O(3)-C(3)-C(4)-C(5) -68.2(4)

C(2)-C(3)-C(4)-C(5) 169.5(3)

C(23)-O(7)-C(5)-C(6) 136.5(3)

C(23)-O(7)-C(5)-C(4) -100.5(4)

C(31)-C(4)-C(5)-O(7) 16.5(5)

C(3)-C(4)-C(5)-O(7) 141.7(3)

C(31)-C(4)-C(5)-C(6) 134.6(4)

C(3)-C(4)-C(5)-C(6) -100.2(4)

C(38)-O(11)-C(6)-C(32) -38.7(5)

C(38)-O(11)-C(6)-C(7) -158.4(4)

C(38)-O(11)-C(6)-C(5) 83.7(4)

O(7)-C(5)-C(6)-O(11) 163.2(3)

C(4)-C(5)-C(6)-O(11) 40.7(4)

O(7)-C(5)-C(6)-C(32) -72.3(4)

C(4)-C(5)-C(6)-C(32) 165.3(3)

O(7)-C(5)-C(6)-C(7) 48.6(4)

C(4)-C(5)-C(6)-C(7) -73.8(4)

O(11)-C(6)-C(7)-C(8) 52.3(4)

C(32)-C(6)-C(7)-C(8) -69.5(5)

C(5)-C(6)-C(7)-C(8) 169.9(3)

C(6)-C(7)-C(8)-C(9) -82.8(4)

C(6)-C(7)-C(8)-C(33) 155.5(4)

C(7)-C(8)-C(9)-O(12) 126.6(4)

C(33)-C(8)-C(9)-O(12) -109.7(4)

C(7)-C(8)-C(9)-C(10) -57.6(5)

C(33)-C(8)-C(9)-C(10) 66.1(5)

O(12)-C(9)-C(10)-C(11) -61.8(5)

C(8)-C(9)-C(10)-C(11) 122.4(4)

O(12)-C(9)-C(10)-C(34) 63.2(5)

C(8)-C(9)-C(10)-C(34) -112.6(4)

C(9)-C(10)-C(11)-O(13) 64.1(4)

C(34)-C(10)-C(11)-O(13) -57.7(5)

C(9)-C(10)-C(11)-C(12) -174.2(3)

C(34)-C(10)-C(11)-C(12) 64.0(5)

O(13)-C(11)-C(12)-O(14) 49.4(4)

C(10)-C(11)-C(12)-O(14) -74.8(5)

O(13)-C(11)-C(12)-C(35) 168.8(4)

C(10)-C(11)-C(12)-C(35) 44.6(5)

O(13)-C(11)-C(12)-C(13) -67.9(4)

C(10)-C(11)-C(12)-C(13) 167.9(3)

C(1)-O(1)-C(13)-C(36) -122.1(4)

C(1)-O(1)-C(13)-C(12) 113.7(4)

O(14)-C(12)-C(13)-O(1) 170.8(3)

C(35)-C(12)-C(13)-O(1) 53.6(5)

C(11)-C(12)-C(13)-O(1) -70.4(4)

O(14)-C(12)-C(13)-C(36) 52.3(5)

C(35)-C(12)-C(13)-C(36) -65.0(5)

C(11)-C(12)-C(13)-C(36) 171.1(4)

C(19)-O(4)-C(15)-O(3) -81.6(4)

C(19)-O(4)-C(15)-C(16) 42.7(5)

C(3)-O(3)-C(15)-O(4) -69.8(4)

C(3)-O(3)-C(15)-C(16) 163.2(3)

O(4)-C(15)-C(16)-C(17) -42.0(5)

O(3)-C(15)-C(16)-C(17) 84.7(5)

C(20)-O(5)-C(17)-C(18) 176.6(4)

C(20)-O(5)-C(17)-C(21) 56.8(5)

C(20)-O(5)-C(17)-C(16) -68.2(5)

C(15)-C(16)-C(17)-O(5) -64.2(5)

C(15)-C(16)-C(17)-C(18) 49.9(5)

C(15)-C(16)-C(17)-C(21) 170.5(4)

O(5)-C(17)-C(18)-O(6) -61.2(4)

C(21)-C(17)-C(18)-O(6) 59.2(5)

C(16)-C(17)-C(18)-O(6) -179.9(3)

O(5)-C(17)-C(18)-C(19) 60.2(4)

C(21)-C(17)-C(18)-C(19) -179.4(4)

C(16)-C(17)-C(18)-C(19) -58.5(4)

C(15)-O(4)-C(19)-C(22) -172.7(3)

C(15)-O(4)-C(19)-C(18) -51.7(4)

O(6)-C(18)-C(19)-O(4) -176.4(3)

C(17)-C(18)-C(19)-O(4) 59.7(4)

O(6)-C(18)-C(19)-C(22) -60.1(5)

C(17)-C(18)-C(19)-C(22) 176.0(4)

C(5)-O(7)-C(23)-O(8) -75.8(4)

C(5)-O(7)-C(23)-C(24) 165.6(3)

C(27)-O(8)-C(23)-O(7) 180.0(3)

C(27)-O(8)-C(23)-C(24) -63.3(4)

O(7)-C(23)-C(24)-O(10) -61.6(4)

O(8)-C(23)-C(24)-O(10) -178.6(3)

O(7)-C(23)-C(24)-C(25) 176.5(3)

O(8)-C(23)-C(24)-C(25) 59.6(4)

O(10)-C(24)-C(25)-C(26) -177.4(3)

C(23)-C(24)-C(25)-C(26) -56.8(4)

O(10)-C(24)-C(25)-N(1) 55.1(4)

C(23)-C(24)-C(25)-N(1) 175.7(3)

O(9)-N(1)-C(25)-C(26) 179.0(3)

C(29)-N(1)-C(25)-C(26) -60.4(4)

C(28)-N(1)-C(25)-C(26) 62.4(5)

O(9)-N(1)-C(25)-C(24) -55.5(4)

C(29)-N(1)-C(25)-C(24) 65.2(4)

C(28)-N(1)-C(25)-C(24) -172.0(4)

C(24)-C(25)-C(26)-C(27) 55.6(4)

N(1)-C(25)-C(26)-C(27) -177.5(3)

C(23)-O(8)-C(27)-C(30) -177.5(3)

C(23)-O(8)-C(27)-C(26) 61.0(4)

C(25)-C(26)-C(27)-O(8) -56.4(4)

C(25)-C(26)-C(27)-C(30) -175.1(4)

O(1)-C(13)-C(36)-C(37) 69.2(5)

C(12)-C(13)-C(36)-C(37) -171.1(4)

________________________________________________________________

Symmetry transformations used to generate equivalent atoms:
