# Supplementary material for: Site-Divergent Oxidations within Venerable Macrolide Antibiotic Scaffolds Unveil Compounds with Broad Spectrum and Anti-MRSA Activities
Source: ACS Cent Sci. 2026 Mar 17;12(3):375–82. doi: 10.1021/acscentsci.5c02343 (PMC13022725; doi:10.1021/acscentsci.5c02343)
Supplement: Supplementary file 5 [file oc5c02343_si_005.zip › Biological, Computational, and X-ray Data/X-Ray/5'/checkCIF_PLATON page 2.pdf]

checkCIF (basic structural check) running

Checking for embedded fcf data in CIF ...
Found embedded fcf data in CIF. Extracting fcf data from uploaded CIF, please wait . . . .

checkCIF/PLATON (basic structural check)

Structure factors have been supplied for datablock(s) 007b-24081

THIS REPORT IS FOR GUIDANCE ONLY. IF USED AS PART OF A REVIEW PROCEDURE FOR PUBLICATION, IT SHOULD NOT REPLACE THE EXPERTISE OF AN EXPERIENCED CRYSTALLOGRAPHIC REFEREE.

No syntax errors found. CIF dictionary
Please wait while processing .... Interpreting this report

Structure factor report

Datablock: 007b-24081

Bond precision:	C-C = 0.0060 A		Wavelength=1.54184
Cell:	a=10.547(2)	b=11.633(3)	c=39.471(7)
	alpha=90	beta=90	gamma=90
Temperature:	93 K		

	Calculated	Reported
Volume	4842.8(18)	4842.8(18)
Space group	P 21 21 21	P 21 21 21
Hall group	P 2ac 2ab	P 2ac 2ab
Moiety formula	2(C37 H65 N O14), C H2 Cl2, 4(C2 H3 N)	C37 H65 N O14, 0.5(C H2 Cl2), 2(C2 H3 N)
Sum formula	C83 H144 Cl2 N6 O28	C41.50 H72 Cl N3 O14
Mr	1744.94	872.47
Dx,g cm-3	1.197	1.197
Z	2	4
Mu (mm-1)	1.222	1.222
F000	1884.0	1884.0
F000'	1891.36	
h,k,lmax	12,13,47	12,13,46
Nref	8614[4844]	8392
Tmin,Tmax	0.746,0.885	0.704,1.000
Tmin'	0.693	

Correction method= # Reported T Limits: Tmin=0.704 Tmax=1.000 AbsCorr = MULTI-SCAN

Data completeness= 1.73/0.97 Theta(max)= 66.883

R(reflections)= 0.0580(8177) wR2(reflections)= 0.1736(8392)

S = 1.065 Npar= 569

The following ALERTS were generated. Each ALERT has the format
test-name_ALERT_alert-type_alert-level.
Click on the hyperlinks for more details of the test.

Alert level C

PLAT041_ALERT_1_C Calc. and Reported SumFormula Strings Differ Please Check
Calc: C83 H144 Cl2 N6 O28
Rep.: C41.50 H72 Cl N3 O14
PLAT042_ALERT_1_C Calc. and Reported MoietyFormula Strings Differ Please Check

Calc: 2(C37 H65 N O14), C H2 Cl2, 4(C2 H3 N)
Rep.: C37 H65 N O14, 0.5(C H2 Cl2), 2(C2 H3 N)

PLAT094_ALERT_2_C Ratio of Maximum / Minimum Residual Density .... 2.57 Report
PLAT244_ALERT_4_C Low 'Solvent' Ueq as Compared to Neighbors of C41 Check
PLAT340_ALERT_3_C Low Bond Precision on C-C Bonds .... 0.00597 Ang.
PLAT911_ALERT_3_C Missing FCF Refl Between Thmin & STh/L= 0.596 68 Report

2 0 0, 2 1 0, 0 2 0, 1 2 0, 9 2 0, 2 0 1,
1 1 1, 2 1 1, 0 3 1, 1 3 1, 1 0 2, 1 1 2,
2 1 2, 0 1 3, 2 1 3, 0 2 3, 1 2 3, 0 0 4,
0 1 4, 1 1 4, 2 1 4, 0 2 4, 2 0 5, 3 0 5,
0 1 5, 0 2 5, 0 4 5, 0 0 6, 1 0 6, 3 0 6,
0 1 6, 1 1 6, 0 2 6, 1 2 6, 2 0 7, 0 2 7,
0 0 8, 0 1 8, 1 1 8, 0 3 8, 2 0 9, 1 1 9,
0 2 9, 0 0 10, 1 1 10, 11 6 10, 1 1 11, 0 3 11,
1 3 12, 3 13 12, 8 10 13, 0 3 14, 1 13 16, 0 3 18,
0 4 18, 10 6 20, 4 10 29, 0 9 34, 3 9 34, 2 9 35,
6 6 36, 0 2 38, 6 4 39, 3 7 39, 5 4 41, 5 1 43,
0 1 45, 0 4 45,

PLAT913_ALERT_3_C Missing # of Very Strong Reflections in FCF .... 14 Note

0 2 0, 1 0 1, 1 1 1, 0 3 1, 1 3 1, 1 0 2,
1 1 2, 1 2 3, 0 2 5, 1 0 6, 3 0 6, 0 2 6,
1 2 6, 2 0 7,

PLAT918_ALERT_3_C Reflection(s) with I(obs) much Smaller I(calc) . 6 Check
PLAT939_ALERT_3_C Large Value of Not (SHELXL) Weight Optimized S . 11.15 Check

---

Alert level G

PLAT007_ALERT_5_G Number of Unrefined Donor-H Atoms .... 4 Report
H6 H10 H11 H13
PLAT045_ALERT_1_G Calculated and Reported Z Differ by a Factor ... 0.500 Check
PLAT299_ALERT_4_G Atom Site Occupancy Constrained at .... 0.5 Check
Cl1 Cl2 C42 H42A H42B
PLAT302_ALERT_4_G Anion/Solvent/Minor-Residue Disorder (Resd 2) 100% Note
PLAT304_ALERT_4_G Non-Integer Number of Atoms in ..... (Resd 2) 2.50 Check
PLAT398_ALERT_2_G Deviating C-O-C Angle From 120 for O12 . 109.4 Degree
PLAT790_ALERT_4_G Centre of Gravity not Within Unit Cell: Resd. # 2 Note
C H2 Cl2
PLAT791_ALERT_4_G Model has Chirality at C2 (Sohncke SpGr) R Verify

And 17 other PLAT791 Alerts

More ...

PLAT909_ALERT_3_G Percentage of I>2sig(I) Data at Theta(Max) Still 93% Note
PLAT910_ALERT_3_G Missing # of FCF Reflection(s) Below Theta(Min). 3 Note
1 0 1, 0 1 1, 0 0 2,
PLAT933_ALERT_2_G Number of HKL-OMIT Records in Embedded .res File 2 Note
0 0 4, 2 0 1,
PLAT965_ALERT_2_G The SHELXL WEIGHT Optimisation has not Converged Please Check
PLAT969_ALERT_5_G The 'Henn et al.' R-Factor-gap value .... 6.555 Note
Predicted wR2: Based on SigI**2 2.65 or SHELX Weight 16.34
PLAT978_ALERT_2_G Number C-C Bonds with Positive Residual Density. 0 Info

---

0 **ALERT level A** = Most likely a serious problem - resolve or explain
0 **ALERT level B** = A potentially serious problem, consider carefully
9 **ALERT level C** = Check. Ensure it is not caused by an omission or oversight
31 **ALERT level G** = General information/check it is not something unexpected

3 ALERT type 1 CIF construction/syntax error, inconsistent or missing data
5 ALERT type 2 Indicator that the structure model may be wrong or deficient
7 ALERT type 3 Indicator that the structure quality may be low
23 ALERT type 4 Improvement, methodology, query or suggestion
2 ALERT type 5 Informative message, check

It is advisable to attempt to resolve as many as possible of the alerts in all categories. Often the minor alerts point to easily fixed oversights, errors and omissions in your CIF or refinement strategy, so attention to these fine details can be worthwhile. In order to resolve some of the more serious problems it may be necessary to carry out additional measurements or structure refinements. However, the purpose of your study may justify the reported deviations and the more serious of these should normally be commented upon in the discussion or experimental section of a paper or in the "special_details" fields of the CIF. checkCIF was carefully

designed to identify outliers and unusual parameters, but every test has its limitations and alerts that are not important in a particular case may appear. Conversely, the absence of alerts does not guarantee there are no aspects of the results needing attention. It is up to the individual to critically assess their own results and, if necessary, seek expert advice.

Publication of your CIF in IUCr journals

A basic structural check has been run on your CIF. These basic checks will be run on all CIFs submitted for publication in IUCr journals (*Acta Crystallographica*, *Journal of Applied Crystallography*, *Journal of Synchrotron Radiation*); however, if you intend to submit to *Acta Crystallographica Section C* or *E* or *IUCrData*, you should make sure that full publication checks are run on the final version of your CIF prior to submission.

Publication of your CIF in other journals

Please refer to the *Notes for Authors* of the relevant journal for any special instructions relating to CIF submission.

PLATON version of 19/12/2024; check.def file version of 19/12/2024

Datablock 007b-24081 - ellipsoid plot

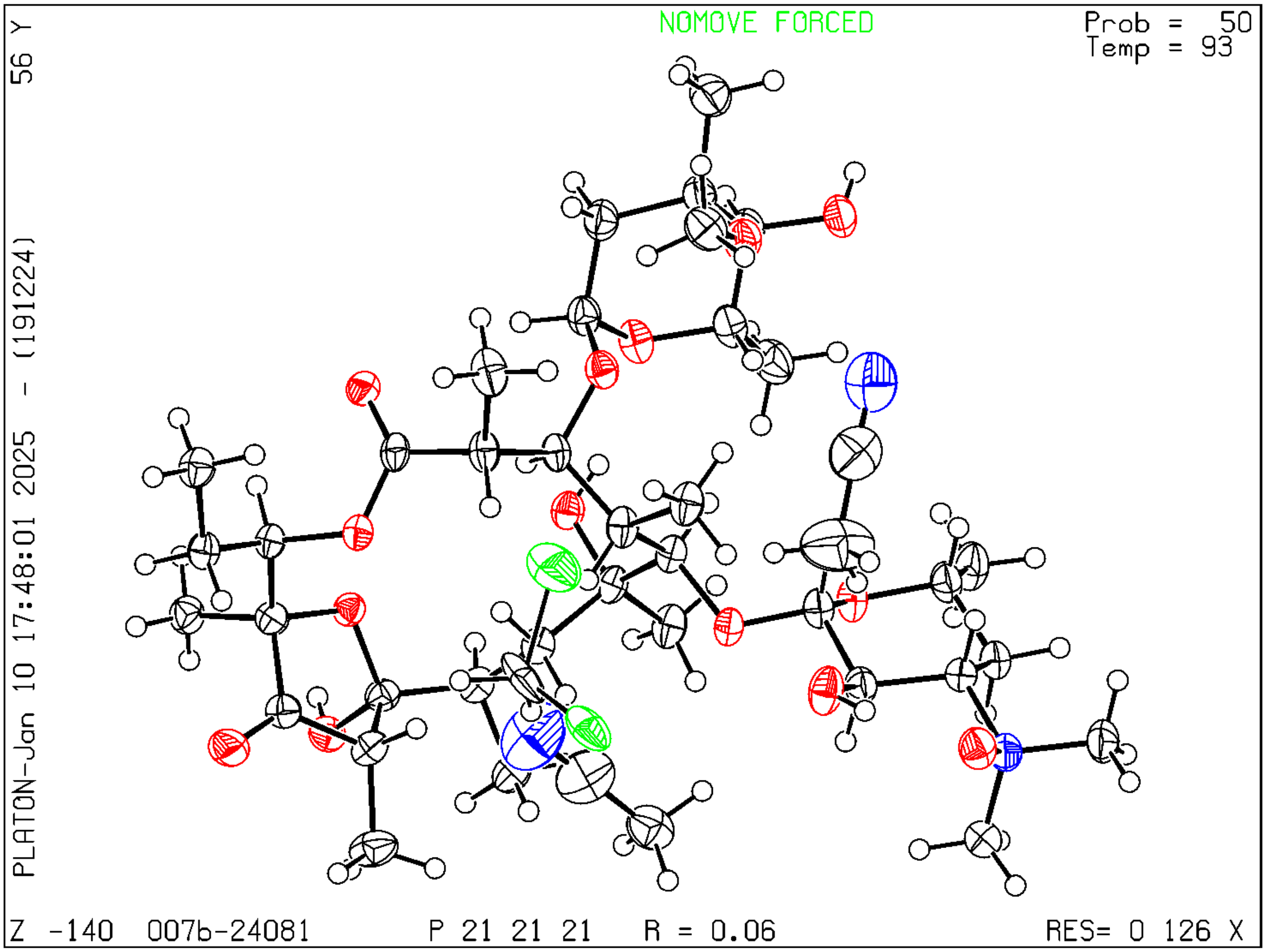

Download CIF editor (publCIF) from the IUCr
Download CIF editor (enCIFer) from the CCDC
Test a new CIF entry
