# Supplementary material for: Site-Divergent Oxidations within Venerable Macrolide Antibiotic Scaffolds Unveil Compounds with Broad Spectrum and Anti-MRSA Activities
Source: ACS Cent Sci. 2026 Mar 17;12(3):375–82. doi: 10.1021/acscentsci.5c02343 (PMC13022725; doi:10.1021/acscentsci.5c02343)
Supplement: Supplementary file 5 [file oc5c02343_si_005.zip › Biological, Computational, and X-ray Data/X-Ray/5'/007b-24081.docx]

***Experimental***

Low-temperature diffraction data (ω-scans) were collected on a Rigaku MicroMax-007HF diffractometer coupled to a Saturn994+ CCD detector with Cu Kα (λ = 1.54178 Å) for the structure of 007b-24081. The diffraction images were processed and scaled using Rigaku Oxford Diffraction software (CrysAlisPro; Rigaku OD: The Woodlands, TX, 2015). The structure was solved with SHELXT and was refined against F^2^ on all data by full-matrix least squares with SHELXL (Sheldrick, G. M. Acta Cryst. 2008, A64, 112–122). All non-hydrogen atoms were refined anisotropically. Hydrogen atoms were included in the model at geometrically calculated positions and refined using a riding model. The isotropic displacement parameters of all hydrogen atoms were fixed to 1.2 times the U value of the atoms to which they are linked (1.5 times for methyl groups). The third solvent pocket is likely a mix of solvents. The difference map suggests that dichloromethane is present at half occupancy. Beyond constraining the site occupancy factors to 0.5, no additional restraints or constraints were needed for a stable refinement. Two low-angle reflections were improperly recorded due to instrument artifacts. These were omitted from the least square refinement. The full numbering scheme of compound 007b-24081 can be found in the full details of the X-ray structure determination (CIF), which is included as Supporting Information. CCDC number XXXXXX (007b-24081) contains the supplementary crystallographic data for this paper. These data can be obtained free of charge from The Cambridge Crystallographic Data Center via www.ccdc.cam.ac.uk/data_request/cif.


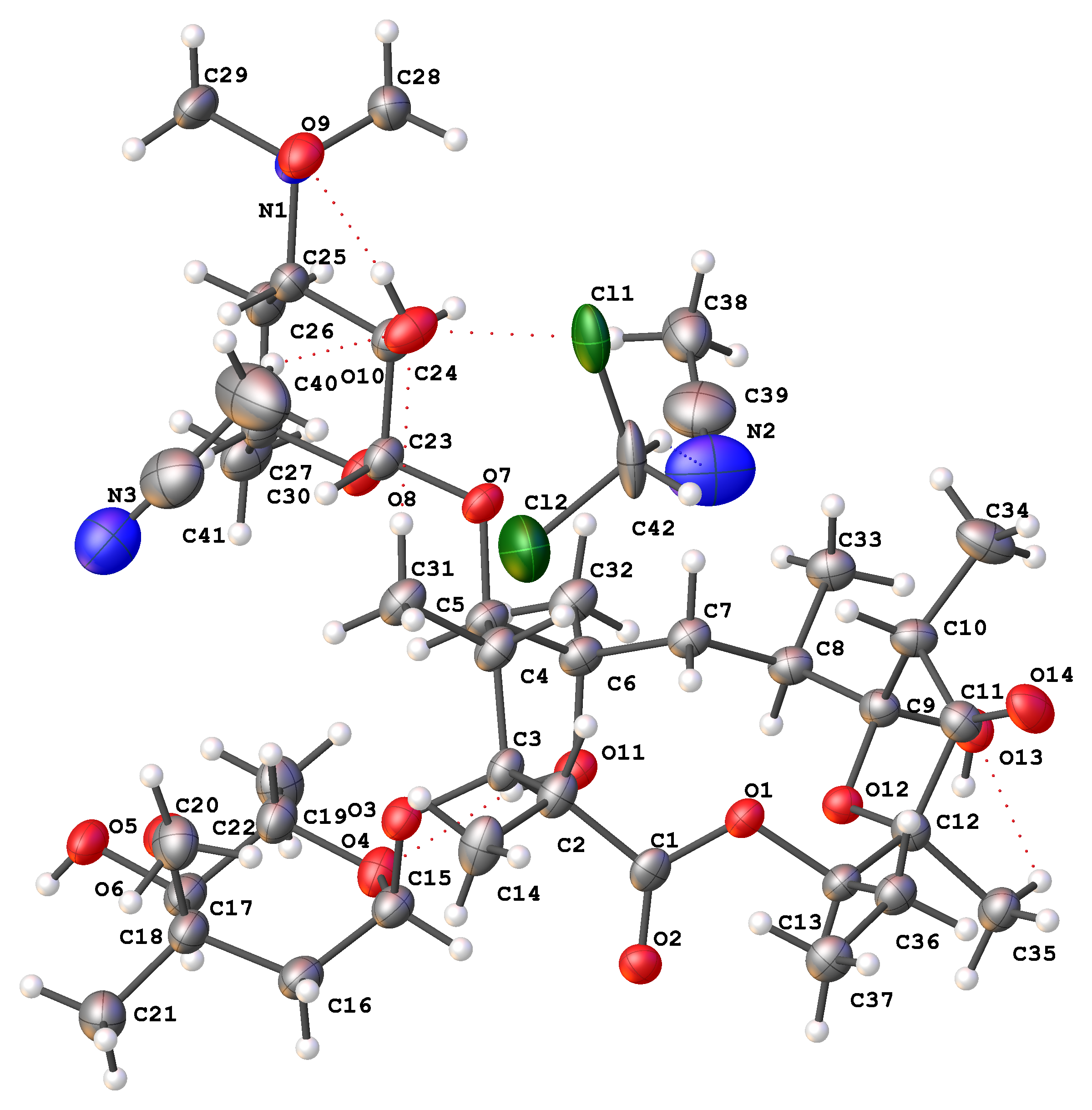


Figure 1. The complete numbering scheme of 007b-24081 with 50% thermal ellipsoid probability levels. The hydrogen atoms are shown as circles for clarity.

Table 1. Crystal data and structure refinement for 007b-24081.

Identification code 007b-24081

Empirical formula C41.50 H72 Cl N3 O14

Formula weight 872.47

Temperature 93(2) K

Wavelength 1.54184 Å

Crystal system Orthorhombic

Space group P2**_1_**2**_1_**2**_1_**

Unit cell dimensions a = 10.547(2) Å α= 90°.

b = 11.633(3) Å β= 90°.

c = 39.471(7) Å γ = 90°.

Volume 4842.8(18) Å3

Z 4

Density (calculated) 1.197 Mg/m3

Absorption coefficient 1.222 mm-1

F(000) 1884

Crystal size 0.300 x 0.200 x 0.100 mm3

Crystal color and habit colorless plate

Diffractometer Rigaku Saturn 944+ CCD

Theta range for data collection 4.412 to 66.883°.

Index ranges -12<=h<=12, -12<=k<=13, -46<=l<=46

Reflections collected 31474

Independent reflections 8392 [R(int) = 0.0787]

Observed reflections (I > 2sigma(I)) 8177

Completeness to theta = 66.883° 98.6 %

Absorption correction Semi-empirical from equivalents

Max. and min. transmission 1.00000 and 0.70424

Solution method SHELXT-2014/5 (Sheldrick, 2014)

Refinement method SHELXL-2014/7 (Sheldrick, 2014)

Data / restraints / parameters 8392 / 0 / 569

Goodness-of-fit on F2 1.065

Final R indices [I>2sigma(I)] R1 = 0.0580, wR2 = 0.1722

R indices (all data) R1 = 0.0590, wR2 = 0.1736

Absolute structure parameter 0.012(17)

Extinction coefficient n/a

Largest diff. peak and hole 0.760 and -0.296 e.Å-3

Table 2. Atomic coordinates ( x 104) and equivalent isotropic displacement parameters (Å2x 103)

for 007b-24081. U(eq) is defined as one third of the trace of the orthogonalized Uij tensor.

________________________________________________________________________________

x y z U(eq)

________________________________________________________________________________

O(1) 8976(2) 5788(2) 4743(1) 26(1)

O(2) 9249(3) 7659(2) 4609(1) 29(1)

O(3) 7908(3) 7472(2) 3764(1) 30(1)

O(4) 6083(3) 8616(3) 3805(1) 34(1)

O(5) 8080(3) 8557(3) 3095(1) 37(1)

O(6) 5842(3) 9547(2) 2923(1) 38(1)

O(7) 5256(3) 4573(2) 3679(1) 28(1)

O(8) 3666(3) 5164(3) 3329(1) 33(1)

O(9) 5341(3) 1657(3) 2721(1) 37(1)

O(10) 5975(3) 2863(3) 3240(1) 39(1)

O(11) 5193(3) 6995(2) 4278(1) 29(1)

O(12) 6716(2) 5867(2) 5066(1) 25(1)

O(13) 5172(3) 4980(2) 5394(1) 30(1)

O(14) 8397(3) 3649(3) 5458(1) 41(1)

N(1) 4091(3) 2090(3) 2743(1) 32(1)

C(1) 9143(3) 6664(3) 4526(1) 26(1)

C(2) 9134(3) 6259(4) 4161(1) 29(1)

C(3) 7801(3) 6571(3) 4011(1) 25(1)

C(4) 7185(4) 5518(4) 3849(1) 29(1)

C(5) 5726(4) 5637(3) 3822(1) 28(1)

C(6) 5005(3) 5838(3) 4159(1) 27(1)

C(7) 5528(3) 5066(3) 4441(1) 27(1)

C(8) 4859(4) 5129(3) 4787(1) 28(1)

C(9) 5802(3) 4954(3) 5078(1) 26(1)

C(10) 6613(4) 3866(3) 5073(1) 30(1)

C(11) 7718(4) 4246(3) 5287(1) 28(1)

C(12) 7796(4) 5565(3) 5272(1) 27(1)

C(13) 8999(4) 6063(3) 5105(1) 26(1)

C(14) 10256(4) 6796(5) 3975(1) 40(1)

C(15) 7429(4) 8554(3) 3858(1) 31(1)

C(16) 8106(4) 9485(4) 3659(1) 36(1)

C(17) 7726(4) 9562(4) 3283(1) 35(1)

C(18) 6275(4) 9585(4) 3263(1) 36(1)

C(19) 5701(4) 8580(4) 3452(1) 36(1)

C(20) 9388(4) 8242(4) 3098(1) 42(1)

C(21) 8283(5) 10645(4) 3122(1) 44(1)

C(22) 4264(5) 8592(4) 3451(1) 45(1)

C(23) 4852(4) 4614(3) 3344(1) 30(1)

C(24) 4763(4) 3371(4) 3226(1) 31(1)

C(25) 4196(4) 3338(3) 2869(1) 30(1)

C(26) 2951(4) 4001(4) 2855(1) 33(1)

C(27) 3175(4) 5218(4) 2987(1) 33(1)

C(28) 3326(5) 1350(4) 2968(1) 38(1)

C(29) 3547(5) 2061(4) 2396(1) 42(1)

C(30) 1973(5) 5924(4) 3006(1) 43(1)

C(31) 7800(4) 5237(4) 3506(1) 35(1)

C(32) 3580(4) 5688(4) 4103(1) 33(1)

C(33) 3767(4) 4260(4) 4814(1) 32(1)

C(34) 6022(4) 2726(4) 5179(1) 43(1)

C(35) 7694(4) 6062(4) 5629(1) 33(1)

C(36) 10246(4) 5624(3) 5248(1) 29(1)

C(37) 11387(4) 6259(4) 5105(1) 33(1)

N(2) 8087(6) 2540(6) 4238(2) 90(2)

C(38) 5794(5) 1844(4) 4058(1) 48(1)

C(39) 7084(6) 2224(5) 4156(2) 66(2)

N(3) 9131(6) 5433(5) 2537(1) 69(1)

C(40) 8934(9) 3395(6) 2791(2) 90(3)

C(41) 9055(6) 4539(6) 2650(2) 62(2)

Cl(1) 11413(2) 747(2) 3650(1) 47(1)

Cl(2) 11257(4) 3251(3) 3715(1) 76(1)

C(42) 11062(9) 1936(11) 3925(2) 53(3)

________________________________________________________________________________ Table 3. Bond lengths [Å] and angles [°] for 007b-24081.

_____________________________________________________

O(1)-C(1) 1.342(5)

O(1)-C(13) 1.464(4)

O(2)-C(1) 1.208(5)

O(3)-C(15) 1.405(5)

O(3)-C(3) 1.435(5)

O(4)-C(15) 1.436(5)

O(4)-C(19) 1.452(5)

O(5)-C(20) 1.428(6)

O(5)-C(17) 1.435(5)

O(6)-C(18) 1.418(5)

O(6)-H(6) 0.8400

O(7)-C(23) 1.390(4)

O(7)-C(5) 1.448(4)

O(8)-C(23) 1.407(5)

O(8)-C(27) 1.448(5)

O(9)-N(1) 1.414(5)

O(10)-C(24) 1.409(5)

O(10)-H(10) 0.8400

O(11)-C(6) 1.440(4)

O(11)-H(11) 0.8400

O(12)-C(9) 1.435(4)

O(12)-C(12) 1.443(4)

O(13)-C(9) 1.413(5)

O(13)-H(13) 0.8400

O(14)-C(11) 1.205(5)

N(1)-C(28) 1.476(6)

N(1)-C(29) 1.485(5)

N(1)-C(25) 1.539(5)

C(1)-C(2) 1.514(5)

C(2)-C(14) 1.527(6)

C(2)-C(3) 1.569(5)

C(2)-H(2) 1.0000

C(3)-C(4) 1.527(5)

C(3)-H(3) 1.0000

C(4)-C(31) 1.538(5)

C(4)-C(5) 1.549(5)

C(4)-H(4) 1.0000

C(5)-C(6) 1.551(5)

C(5)-H(5) 1.0000

C(6)-C(32) 1.529(5)

C(6)-C(7) 1.532(5)

C(7)-C(8) 1.539(5)

C(7)-H(7A) 0.9900

C(7)-H(7B) 0.9900

C(8)-C(9) 1.534(5)

C(8)-C(33) 1.536(5)

C(8)-H(8) 1.0000

C(9)-C(10) 1.527(5)

C(10)-C(11) 1.504(5)

C(10)-C(34) 1.523(6)

C(10)-H(10A) 1.0000

C(11)-C(12) 1.538(5)

C(12)-C(35) 1.527(5)

C(12)-C(13) 1.544(5)

C(13)-C(36) 1.519(5)

C(13)-H(13A) 1.0000

C(14)-H(14A) 0.9800

C(14)-H(14B) 0.9800

C(14)-H(14C) 0.9800

C(15)-C(16) 1.516(6)

C(15)-H(15) 1.0000

C(16)-C(17) 1.541(5)

C(16)-H(16A) 0.9900

C(16)-H(16B) 0.9900

C(17)-C(21) 1.530(6)

C(17)-C(18) 1.533(6)

C(18)-C(19) 1.512(6)

C(18)-H(18) 1.0000

C(19)-C(22) 1.516(6)

C(19)-H(19) 1.0000

C(20)-H(20A) 0.9800

C(20)-H(20B) 0.9800

C(20)-H(20C) 0.9800

C(21)-H(21A) 0.9800

C(21)-H(21B) 0.9800

C(21)-H(21C) 0.9800

C(22)-H(22A) 0.9800

C(22)-H(22B) 0.9800

C(22)-H(22C) 0.9800

C(23)-C(24) 1.522(6)

C(23)-H(23) 1.0000

C(24)-C(25) 1.529(5)

C(24)-H(24) 1.0000

C(25)-C(26) 1.523(6)

C(25)-H(25) 1.0000

C(26)-C(27) 1.526(6)

C(26)-H(26A) 0.9900

C(26)-H(26B) 0.9900

C(27)-C(30) 1.512(6)

C(27)-H(27) 1.0000

C(28)-H(28A) 0.9800

C(28)-H(28B) 0.9800

C(28)-H(28C) 0.9800

C(29)-H(29A) 0.9800

C(29)-H(29B) 0.9800

C(29)-H(29C) 0.9800

C(30)-H(30A) 0.9800

C(30)-H(30B) 0.9800

C(30)-H(30C) 0.9800

C(31)-H(31A) 0.9800

C(31)-H(31B) 0.9800

C(31)-H(31C) 0.9800

C(32)-H(32A) 0.9800

C(32)-H(32B) 0.9800

C(32)-H(32C) 0.9800

C(33)-H(33A) 0.9800

C(33)-H(33B) 0.9800

C(33)-H(33C) 0.9800

C(34)-H(34A) 0.9800

C(34)-H(34B) 0.9800

C(34)-H(34C) 0.9800

C(35)-H(35A) 0.9800

C(35)-H(35B) 0.9800

C(35)-H(35C) 0.9800

C(36)-C(37) 1.520(6)

C(36)-H(36A) 0.9900

C(36)-H(36B) 0.9900

C(37)-H(37A) 0.9800

C(37)-H(37B) 0.9800

C(37)-H(37C) 0.9800

N(2)-C(39) 1.165(9)

C(38)-C(39) 1.482(9)

C(38)-H(38A) 0.9800

C(38)-H(38B) 0.9800

C(38)-H(38C) 0.9800

N(3)-C(41) 1.134(8)

C(40)-C(41) 1.447(10)

C(40)-H(40A) 0.9800

C(40)-H(40B) 0.9800

C(40)-H(40C) 0.9800

Cl(1)-C(42) 1.797(13)

Cl(2)-C(42) 1.752(11)

C(42)-H(42A) 0.9900

C(42)-H(42B) 0.9900

C(1)-O(1)-C(13) 117.0(3)

C(15)-O(3)-C(3) 116.6(3)

C(15)-O(4)-C(19) 114.4(3)

C(20)-O(5)-C(17) 117.1(3)

C(18)-O(6)-H(6) 109.5

C(23)-O(7)-C(5) 116.5(3)

C(23)-O(8)-C(27) 112.1(3)

C(24)-O(10)-H(10) 109.5

C(6)-O(11)-H(11) 109.5

C(9)-O(12)-C(12) 109.4(3)

C(9)-O(13)-H(13) 109.5

O(9)-N(1)-C(28) 109.8(3)

O(9)-N(1)-C(29) 107.2(3)

C(28)-N(1)-C(29) 109.2(3)

O(9)-N(1)-C(25) 106.8(3)

C(28)-N(1)-C(25) 113.3(3)

C(29)-N(1)-C(25) 110.3(3)

O(2)-C(1)-O(1) 124.5(3)

O(2)-C(1)-C(2) 123.8(4)

O(1)-C(1)-C(2) 111.6(3)

C(1)-C(2)-C(14) 109.1(3)

C(1)-C(2)-C(3) 107.0(3)

C(14)-C(2)-C(3) 114.6(3)

C(1)-C(2)-H(2) 108.7

C(14)-C(2)-H(2) 108.7

C(3)-C(2)-H(2) 108.7

O(3)-C(3)-C(4) 109.6(3)

O(3)-C(3)-C(2) 110.9(3)

C(4)-C(3)-C(2) 110.7(3)

O(3)-C(3)-H(3) 108.5

C(4)-C(3)-H(3) 108.5

C(2)-C(3)-H(3) 108.5

C(3)-C(4)-C(31) 111.1(3)

C(3)-C(4)-C(5) 112.3(3)

C(31)-C(4)-C(5) 112.1(3)

C(3)-C(4)-H(4) 107.0

C(31)-C(4)-H(4) 107.0

C(5)-C(4)-H(4) 107.0

O(7)-C(5)-C(4) 106.9(3)

O(7)-C(5)-C(6) 107.2(3)

C(4)-C(5)-C(6) 116.2(3)

O(7)-C(5)-H(5) 108.8

C(4)-C(5)-H(5) 108.8

C(6)-C(5)-H(5) 108.8

O(11)-C(6)-C(32) 106.8(3)

O(11)-C(6)-C(7) 105.2(3)

C(32)-C(6)-C(7) 113.1(3)

O(11)-C(6)-C(5) 110.6(3)

C(32)-C(6)-C(5) 109.8(3)

C(7)-C(6)-C(5) 111.0(3)

C(6)-C(7)-C(8) 116.8(3)

C(6)-C(7)-H(7A) 108.1

C(8)-C(7)-H(7A) 108.1

C(6)-C(7)-H(7B) 108.1

C(8)-C(7)-H(7B) 108.1

H(7A)-C(7)-H(7B) 107.3

C(9)-C(8)-C(33) 110.3(3)

C(9)-C(8)-C(7) 111.2(3)

C(33)-C(8)-C(7) 111.9(3)

C(9)-C(8)-H(8) 107.8

C(33)-C(8)-H(8) 107.8

C(7)-C(8)-H(8) 107.8

O(13)-C(9)-O(12) 109.2(3)

O(13)-C(9)-C(10) 107.0(3)

O(12)-C(9)-C(10) 103.7(3)

O(13)-C(9)-C(8) 110.7(3)

O(12)-C(9)-C(8) 108.2(3)

C(10)-C(9)-C(8) 117.7(3)

C(11)-C(10)-C(34) 114.8(4)

C(11)-C(10)-C(9) 100.6(3)

C(34)-C(10)-C(9) 119.2(3)

C(11)-C(10)-H(10A) 107.2

C(34)-C(10)-H(10A) 107.2

C(9)-C(10)-H(10A) 107.2

O(14)-C(11)-C(10) 127.3(4)

O(14)-C(11)-C(12) 124.3(4)

C(10)-C(11)-C(12) 108.3(3)

O(12)-C(12)-C(35) 111.8(3)

O(12)-C(12)-C(11) 102.8(3)

C(35)-C(12)-C(11) 109.8(3)

O(12)-C(12)-C(13) 108.5(3)

C(35)-C(12)-C(13) 108.1(3)

C(11)-C(12)-C(13) 115.8(3)

O(1)-C(13)-C(36) 107.7(3)

O(1)-C(13)-C(12) 108.8(3)

C(36)-C(13)-C(12) 115.2(3)

O(1)-C(13)-H(13A) 108.3

C(36)-C(13)-H(13A) 108.3

C(12)-C(13)-H(13A) 108.3

C(2)-C(14)-H(14A) 109.5

C(2)-C(14)-H(14B) 109.5

H(14A)-C(14)-H(14B) 109.5

C(2)-C(14)-H(14C) 109.5

H(14A)-C(14)-H(14C) 109.5

H(14B)-C(14)-H(14C) 109.5

O(3)-C(15)-O(4) 111.2(3)

O(3)-C(15)-C(16) 109.6(3)

O(4)-C(15)-C(16) 110.8(3)

O(3)-C(15)-H(15) 108.4

O(4)-C(15)-H(15) 108.4

C(16)-C(15)-H(15) 108.4

C(15)-C(16)-C(17) 114.7(3)

C(15)-C(16)-H(16A) 108.6

C(17)-C(16)-H(16A) 108.6

C(15)-C(16)-H(16B) 108.6

C(17)-C(16)-H(16B) 108.6

H(16A)-C(16)-H(16B) 107.6

O(5)-C(17)-C(21) 110.9(3)

O(5)-C(17)-C(18) 104.3(3)

C(21)-C(17)-C(18) 110.3(4)

O(5)-C(17)-C(16) 112.6(3)

C(21)-C(17)-C(16) 110.5(4)

C(18)-C(17)-C(16) 108.1(4)

O(6)-C(18)-C(19) 108.2(3)

O(6)-C(18)-C(17) 111.7(4)

C(19)-C(18)-C(17) 111.1(4)

O(6)-C(18)-H(18) 108.6

C(19)-C(18)-H(18) 108.6

C(17)-C(18)-H(18) 108.6

O(4)-C(19)-C(18) 109.8(3)

O(4)-C(19)-C(22) 106.1(4)

C(18)-C(19)-C(22) 113.1(4)

O(4)-C(19)-H(19) 109.2

C(18)-C(19)-H(19) 109.2

C(22)-C(19)-H(19) 109.2

O(5)-C(20)-H(20A) 109.5

O(5)-C(20)-H(20B) 109.5

H(20A)-C(20)-H(20B) 109.5

O(5)-C(20)-H(20C) 109.5

H(20A)-C(20)-H(20C) 109.5

H(20B)-C(20)-H(20C) 109.5

C(17)-C(21)-H(21A) 109.5

C(17)-C(21)-H(21B) 109.5

H(21A)-C(21)-H(21B) 109.5

C(17)-C(21)-H(21C) 109.5

H(21A)-C(21)-H(21C) 109.5

H(21B)-C(21)-H(21C) 109.5

C(19)-C(22)-H(22A) 109.5

C(19)-C(22)-H(22B) 109.5

H(22A)-C(22)-H(22B) 109.5

C(19)-C(22)-H(22C) 109.5

H(22A)-C(22)-H(22C) 109.5

H(22B)-C(22)-H(22C) 109.5

O(7)-C(23)-O(8) 109.2(3)

O(7)-C(23)-C(24) 106.2(3)

O(8)-C(23)-C(24) 111.4(3)

O(7)-C(23)-H(23) 110.0

O(8)-C(23)-H(23) 110.0

C(24)-C(23)-H(23) 110.0

O(10)-C(24)-C(23) 109.3(3)

O(10)-C(24)-C(25) 112.5(3)

C(23)-C(24)-C(25) 109.3(3)

O(10)-C(24)-H(24) 108.6

C(23)-C(24)-H(24) 108.6

C(25)-C(24)-H(24) 108.6

C(26)-C(25)-C(24) 111.0(3)

C(26)-C(25)-N(1) 113.8(3)

C(24)-C(25)-N(1) 110.4(3)

C(26)-C(25)-H(25) 107.1

C(24)-C(25)-H(25) 107.1

N(1)-C(25)-H(25) 107.1

C(25)-C(26)-C(27) 108.9(3)

C(25)-C(26)-H(26A) 109.9

C(27)-C(26)-H(26A) 109.9

C(25)-C(26)-H(26B) 109.9

C(27)-C(26)-H(26B) 109.9

H(26A)-C(26)-H(26B) 108.3

O(8)-C(27)-C(30) 106.0(3)

O(8)-C(27)-C(26) 109.5(3)

C(30)-C(27)-C(26) 113.0(4)

O(8)-C(27)-H(27) 109.4

C(30)-C(27)-H(27) 109.4

C(26)-C(27)-H(27) 109.4

N(1)-C(28)-H(28A) 109.5

N(1)-C(28)-H(28B) 109.5

H(28A)-C(28)-H(28B) 109.5

N(1)-C(28)-H(28C) 109.5

H(28A)-C(28)-H(28C) 109.5

H(28B)-C(28)-H(28C) 109.5

N(1)-C(29)-H(29A) 109.5

N(1)-C(29)-H(29B) 109.5

H(29A)-C(29)-H(29B) 109.5

N(1)-C(29)-H(29C) 109.5

H(29A)-C(29)-H(29C) 109.5

H(29B)-C(29)-H(29C) 109.5

C(27)-C(30)-H(30A) 109.5

C(27)-C(30)-H(30B) 109.5

H(30A)-C(30)-H(30B) 109.5

C(27)-C(30)-H(30C) 109.5

H(30A)-C(30)-H(30C) 109.5

H(30B)-C(30)-H(30C) 109.5

C(4)-C(31)-H(31A) 109.5

C(4)-C(31)-H(31B) 109.5

H(31A)-C(31)-H(31B) 109.5

C(4)-C(31)-H(31C) 109.5

H(31A)-C(31)-H(31C) 109.5

H(31B)-C(31)-H(31C) 109.5

C(6)-C(32)-H(32A) 109.5

C(6)-C(32)-H(32B) 109.5

H(32A)-C(32)-H(32B) 109.5

C(6)-C(32)-H(32C) 109.5

H(32A)-C(32)-H(32C) 109.5

H(32B)-C(32)-H(32C) 109.5

C(8)-C(33)-H(33A) 109.5

C(8)-C(33)-H(33B) 109.5

H(33A)-C(33)-H(33B) 109.5

C(8)-C(33)-H(33C) 109.5

H(33A)-C(33)-H(33C) 109.5

H(33B)-C(33)-H(33C) 109.5

C(10)-C(34)-H(34A) 109.5

C(10)-C(34)-H(34B) 109.5

H(34A)-C(34)-H(34B) 109.5

C(10)-C(34)-H(34C) 109.5

H(34A)-C(34)-H(34C) 109.5

H(34B)-C(34)-H(34C) 109.5

C(12)-C(35)-H(35A) 109.5

C(12)-C(35)-H(35B) 109.5

H(35A)-C(35)-H(35B) 109.5

C(12)-C(35)-H(35C) 109.5

H(35A)-C(35)-H(35C) 109.5

H(35B)-C(35)-H(35C) 109.5

C(13)-C(36)-C(37) 112.6(3)

C(13)-C(36)-H(36A) 109.1

C(37)-C(36)-H(36A) 109.1

C(13)-C(36)-H(36B) 109.1

C(37)-C(36)-H(36B) 109.1

H(36A)-C(36)-H(36B) 107.8

C(36)-C(37)-H(37A) 109.5

C(36)-C(37)-H(37B) 109.5

H(37A)-C(37)-H(37B) 109.5

C(36)-C(37)-H(37C) 109.5

H(37A)-C(37)-H(37C) 109.5

H(37B)-C(37)-H(37C) 109.5

C(39)-C(38)-H(38A) 109.5

C(39)-C(38)-H(38B) 109.5

H(38A)-C(38)-H(38B) 109.5

C(39)-C(38)-H(38C) 109.5

H(38A)-C(38)-H(38C) 109.5

H(38B)-C(38)-H(38C) 109.5

N(2)-C(39)-C(38) 178.6(7)

C(41)-C(40)-H(40A) 109.5

C(41)-C(40)-H(40B) 109.5

H(40A)-C(40)-H(40B) 109.5

C(41)-C(40)-H(40C) 109.5

H(40A)-C(40)-H(40C) 109.5

H(40B)-C(40)-H(40C) 109.5

N(3)-C(41)-C(40) 178.8(9)

Cl(2)-C(42)-Cl(1) 111.3(5)

Cl(2)-C(42)-H(42A) 109.4

Cl(1)-C(42)-H(42A) 109.4

Cl(2)-C(42)-H(42B) 109.4

Cl(1)-C(42)-H(42B) 109.4

H(42A)-C(42)-H(42B) 108.0

_____________________________________________________________

Symmetry transformations used to generate equivalent atoms:

Table 4. Anisotropic displacement parameters (Å2x 103) for 007b-24081. The anisotropic

displacement factor exponent takes the form: -2π2[ h2 a*2U11 + ... + 2 h k a* b* U12 ]

______________________________________________________________________________

U11 U22 U33 U23 U13 U12

______________________________________________________________________________

O(1) 26(1) 30(1) 23(1) -3(1) -2(1) 0(1)

O(2) 28(1) 29(1) 31(1) 0(1) -4(1) -2(1)

O(3) 32(1) 32(1) 24(1) -2(1) 0(1) 4(1)

O(4) 35(1) 40(2) 27(1) -1(1) -1(1) 10(1)

O(5) 40(2) 37(2) 33(1) -4(1) -1(1) 3(1)

O(6) 55(2) 33(1) 27(1) -1(1) -10(1) 7(1)

O(7) 29(1) 32(1) 24(1) -4(1) -6(1) 1(1)

O(8) 31(1) 42(2) 26(1) -4(1) -7(1) 5(1)

O(9) 44(2) 34(1) 32(1) -4(1) -2(1) 7(1)

O(10) 37(2) 42(2) 38(2) -12(1) -10(1) 11(1)

O(11) 29(1) 31(1) 28(1) -4(1) -2(1) 3(1)

O(12) 24(1) 26(1) 27(1) -1(1) -3(1) 0(1)

O(13) 30(1) 32(1) 28(1) 1(1) 4(1) 0(1)

O(14) 42(2) 34(2) 48(2) 11(1) -12(1) -1(1)

N(1) 38(2) 32(2) 28(2) -1(1) -6(1) 0(2)

C(1) 16(2) 37(2) 25(2) -2(2) -5(1) 1(2)

C(2) 22(2) 41(2) 24(2) -3(2) -1(1) 5(2)

C(3) 22(2) 33(2) 21(2) -1(2) -2(1) 4(2)

C(4) 24(2) 38(2) 25(2) -6(2) 0(2) 5(2)

C(5) 26(2) 29(2) 27(2) -5(2) -5(2) 2(2)

C(6) 21(2) 32(2) 29(2) -5(2) -3(1) 1(2)

C(7) 22(2) 30(2) 29(2) -4(2) 0(2) 4(1)

C(8) 24(2) 28(2) 31(2) -5(2) 1(2) 1(2)

C(9) 25(2) 26(2) 28(2) -1(2) 2(2) -2(2)

C(10) 27(2) 27(2) 37(2) -4(2) -2(2) -1(2)

C(11) 26(2) 27(2) 31(2) 1(2) 0(2) 2(2)

C(12) 24(2) 28(2) 28(2) 2(2) 0(2) 0(2)

C(13) 28(2) 27(2) 22(2) -4(1) -1(1) -1(2)

C(14) 22(2) 66(3) 31(2) 2(2) 2(2) 2(2)

C(15) 35(2) 32(2) 27(2) -4(2) -3(2) 6(2)

C(16) 43(2) 35(2) 31(2) -4(2) -6(2) 2(2)

C(17) 45(2) 33(2) 26(2) -1(2) -4(2) 3(2)

C(18) 46(2) 33(2) 28(2) -4(2) -5(2) 5(2)

C(19) 42(2) 39(2) 27(2) 0(2) -4(2) 7(2)

C(20) 43(2) 47(2) 36(2) 1(2) 7(2) 5(2)

C(21) 51(3) 44(2) 38(2) 2(2) 0(2) 0(2)

C(22) 42(2) 48(3) 44(2) 9(2) -8(2) 7(2)

C(23) 30(2) 34(2) 24(2) -2(2) -4(2) 5(2)

C(24) 32(2) 34(2) 28(2) 1(2) -5(2) 1(2)

C(25) 35(2) 30(2) 26(2) 1(2) -3(2) -5(2)

C(26) 35(2) 37(2) 27(2) 2(2) -8(2) -2(2)

C(27) 37(2) 36(2) 27(2) 0(2) -8(2) 5(2)

C(28) 47(2) 35(2) 33(2) 3(2) -4(2) -4(2)

C(29) 59(3) 38(2) 29(2) -4(2) -13(2) 0(2)

C(30) 42(2) 47(2) 40(2) -5(2) -13(2) 8(2)

C(31) 29(2) 45(2) 30(2) -8(2) -1(2) 2(2)

C(32) 24(2) 43(2) 33(2) -8(2) -2(2) 4(2)

C(33) 24(2) 35(2) 39(2) -4(2) 1(2) -1(2)

C(34) 37(2) 28(2) 66(3) 4(2) -5(2) -1(2)

C(35) 33(2) 38(2) 27(2) -2(2) 2(2) -3(2)

C(36) 27(2) 32(2) 28(2) -2(2) -4(2) 2(2)

C(37) 24(2) 40(2) 35(2) -2(2) -1(2) 0(2)

N(2) 53(3) 89(4) 128(6) -16(4) -18(3) 15(3)

C(38) 57(3) 45(3) 42(2) 1(2) 10(2) 6(2)

C(39) 64(4) 52(3) 82(4) -15(3) 9(3) 11(3)

N(3) 64(3) 76(4) 67(3) -6(3) -4(3) 12(3)

C(40) 105(6) 69(4) 96(6) -8(4) 50(5) -7(4)

C(41) 57(3) 71(4) 59(3) -13(3) 14(3) -2(3)

Cl(1) 32(1) 71(1) 38(1) 25(1) 7(1) -1(1)

Cl(2) 102(2) 73(2) 52(2) 14(1) 9(2) -14(2)

C(42) 35(4) 91(8) 32(4) 36(5) 4(4) 2(5)

______________________________________________________________________________ Table 5. Hydrogen coordinates ( x 104) and isotropic displacement parameters (Å2x 10 3)

for 007b-24081.

________________________________________________________________________________

x y z U(eq)

________________________________________________________________________________

H(6) 5619 10209 2862 57

H(10) 6041 2367 3086 59

H(11) 5326 7431 4112 43

H(13) 4947 5657 5438 45

H(2) 9234 5404 4159 35

H(3) 7245 6845 4200 30

H(4) 7357 4851 4002 35

H(5) 5524 6281 3663 33

H(7A) 6432 5263 4475 32

H(7B) 5493 4260 4361 32

H(8) 4489 5917 4811 33

H(10A) 6933 3768 4837 36

H(13A) 8981 6918 5130 31

H(14A) 11049 6543 4080 60

H(14B) 10195 7635 3987 60

H(14C) 10242 6553 3737 60

H(15) 7603 8675 4104 38

H(16A) 9031 9346 3673 43

H(16B) 7933 10235 3768 43

H(18) 5966 10313 3369 43

H(19) 6006 7846 3347 43

H(20A) 9525 7598 2942 63

H(20B) 9633 8012 3328 63

H(20C) 9904 8899 3026 63

H(21A) 9210 10621 3137 66

H(21B) 7968 11325 3242 66

H(21C) 8028 10685 2883 66

H(22A) 3948 7913 3572 68

H(22B) 3955 8581 3217 68

H(22C) 3962 9289 3565 68

H(23) 5483 5041 3203 36

H(24) 4186 2945 3382 37

H(25) 4805 3742 2716 36

H(26A) 2305 3611 2995 40

H(26B) 2638 4031 2618 40

H(27) 3802 5617 2838 40

H(28A) 3733 1302 3191 57

H(28B) 3261 579 2869 57

H(28C) 2475 1679 2993 57

H(29A) 2636 2220 2407 63

H(29B) 3683 1298 2297 63

H(29C) 3963 2643 2256 63

H(30A) 1567 5944 2783 65

H(30B) 2180 6708 3077 65

H(30C) 1392 5576 3170 65

H(31A) 7648 5871 3347 52

H(31B) 7428 4529 3415 52

H(31C) 8715 5132 3536 52

H(32A) 3305 6183 3916 50

H(32B) 3123 5901 4310 50

H(32C) 3399 4884 4047 50

H(33A) 3055 4518 4674 49

H(33B) 3492 4201 5050 49

H(33C) 4057 3506 4735 49

H(34A) 5384 2864 5355 65

H(34B) 6683 2217 5268 65

H(34C) 5619 2365 4983 65

H(35A) 8405 5786 5767 49

H(35B) 6893 5816 5732 49

H(35C) 7718 6904 5617 49

H(36A) 10329 4795 5195 35

H(36B) 10237 5709 5497 35

H(37A) 11417 6158 4859 50

H(37B) 12164 5950 5207 50

H(37C) 11316 7079 5159 50

H(38A) 5200 2017 4242 72

H(38B) 5799 1014 4016 72

H(38C) 5530 2250 3853 72

H(40A) 9291 2834 2632 135

H(40B) 8036 3222 2829 135

H(40C) 9392 3354 3006 135

H(42A) 10178 1870 4007 63

H(42B) 11631 1910 4124 63

________________________________________________________________________________ Table 6. Torsion angles [°] for 007b-24081.

________________________________________________________________

C(13)-O(1)-C(1)-O(2) -3.7(5)

C(13)-O(1)-C(1)-C(2) 178.4(3)

O(2)-C(1)-C(2)-C(14) 49.1(5)

O(1)-C(1)-C(2)-C(14) -133.0(3)

O(2)-C(1)-C(2)-C(3) -75.4(4)

O(1)-C(1)-C(2)-C(3) 102.5(3)

C(15)-O(3)-C(3)-C(4) 128.6(3)

C(15)-O(3)-C(3)-C(2) -108.9(3)

C(1)-C(2)-C(3)-O(3) 110.4(3)

C(14)-C(2)-C(3)-O(3) -10.6(4)

C(1)-C(2)-C(3)-C(4) -127.7(3)

C(14)-C(2)-C(3)-C(4) 111.3(4)

O(3)-C(3)-C(4)-C(31) 47.2(4)

C(2)-C(3)-C(4)-C(31) -75.4(4)

O(3)-C(3)-C(4)-C(5) -79.3(4)

C(2)-C(3)-C(4)-C(5) 158.1(3)

C(23)-O(7)-C(5)-C(4) -106.5(3)

C(23)-O(7)-C(5)-C(6) 128.3(3)

C(3)-C(4)-C(5)-O(7) -177.7(3)

C(31)-C(4)-C(5)-O(7) 56.4(4)

C(3)-C(4)-C(5)-C(6) -58.1(4)

C(31)-C(4)-C(5)-C(6) 175.9(3)

O(7)-C(5)-C(6)-O(11) -166.6(3)

C(4)-C(5)-C(6)-O(11) 74.1(4)

O(7)-C(5)-C(6)-C(32) -48.9(4)

C(4)-C(5)-C(6)-C(32) -168.3(3)

O(7)-C(5)-C(6)-C(7) 77.0(4)

C(4)-C(5)-C(6)-C(7) -42.4(4)

O(11)-C(6)-C(7)-C(8) 63.2(4)

C(32)-C(6)-C(7)-C(8) -53.0(4)

C(5)-C(6)-C(7)-C(8) -177.1(3)

C(6)-C(7)-C(8)-C(9) -146.4(3)

C(6)-C(7)-C(8)-C(33) 89.8(4)

C(12)-O(12)-C(9)-O(13) 75.1(3)

C(12)-O(12)-C(9)-C(10) -38.7(3)

C(12)-O(12)-C(9)-C(8) -164.4(3)

C(33)-C(8)-C(9)-O(13) -53.6(4)

C(7)-C(8)-C(9)-O(13) -178.3(3)

C(33)-C(8)-C(9)-O(12) -173.2(3)

C(7)-C(8)-C(9)-O(12) 62.1(4)

C(33)-C(8)-C(9)-C(10) 69.9(4)

C(7)-C(8)-C(9)-C(10) -54.9(4)

O(13)-C(9)-C(10)-C(11) -78.3(3)

O(12)-C(9)-C(10)-C(11) 37.0(4)

C(8)-C(9)-C(10)-C(11) 156.4(3)

O(13)-C(9)-C(10)-C(34) 48.0(5)

O(12)-C(9)-C(10)-C(34) 163.4(4)

C(8)-C(9)-C(10)-C(34) -77.3(5)

C(34)-C(10)-C(11)-O(14) 22.8(6)

C(9)-C(10)-C(11)-O(14) 152.1(4)

C(34)-C(10)-C(11)-C(12) -153.2(3)

C(9)-C(10)-C(11)-C(12) -23.9(4)

C(9)-O(12)-C(12)-C(35) -95.2(3)

C(9)-O(12)-C(12)-C(11) 22.6(4)

C(9)-O(12)-C(12)-C(13) 145.7(3)

O(14)-C(11)-C(12)-O(12) -173.8(4)

C(10)-C(11)-C(12)-O(12) 2.3(4)

O(14)-C(11)-C(12)-C(35) -54.6(5)

C(10)-C(11)-C(12)-C(35) 121.5(3)

O(14)-C(11)-C(12)-C(13) 68.1(5)

C(10)-C(11)-C(12)-C(13) -115.7(4)

C(1)-O(1)-C(13)-C(36) -106.5(3)

C(1)-O(1)-C(13)-C(12) 128.0(3)

O(12)-C(12)-C(13)-O(1) -47.1(4)

C(35)-C(12)-C(13)-O(1) -168.5(3)

C(11)-C(12)-C(13)-O(1) 67.8(4)

O(12)-C(12)-C(13)-C(36) -168.0(3)

C(35)-C(12)-C(13)-C(36) 70.5(4)

C(11)-C(12)-C(13)-C(36) -53.1(4)

C(3)-O(3)-C(15)-O(4) -82.9(4)

C(3)-O(3)-C(15)-C(16) 154.4(3)

C(19)-O(4)-C(15)-O(3) -67.3(4)

C(19)-O(4)-C(15)-C(16) 54.7(4)

O(3)-C(15)-C(16)-C(17) 73.2(4)

O(4)-C(15)-C(16)-C(17) -49.8(4)

C(20)-O(5)-C(17)-C(21) 67.5(5)

C(20)-O(5)-C(17)-C(18) -173.8(3)

C(20)-O(5)-C(17)-C(16) -56.8(5)

C(15)-C(16)-C(17)-O(5) -65.1(5)

C(15)-C(16)-C(17)-C(21) 170.3(4)

C(15)-C(16)-C(17)-C(18) 49.5(5)

O(5)-C(17)-C(18)-O(6) -54.5(4)

C(21)-C(17)-C(18)-O(6) 64.6(4)

C(16)-C(17)-C(18)-O(6) -174.5(3)

O(5)-C(17)-C(18)-C(19) 66.5(4)

C(21)-C(17)-C(18)-C(19) -174.4(3)

C(16)-C(17)-C(18)-C(19) -53.6(4)

C(15)-O(4)-C(19)-C(18) -60.3(4)

C(15)-O(4)-C(19)-C(22) 177.1(4)

O(6)-C(18)-C(19)-O(4) -177.7(3)

C(17)-C(18)-C(19)-O(4) 59.3(4)

O(6)-C(18)-C(19)-C(22) -59.4(5)

C(17)-C(18)-C(19)-C(22) 177.6(4)

C(5)-O(7)-C(23)-O(8) -75.3(4)

C(5)-O(7)-C(23)-C(24) 164.6(3)

C(27)-O(8)-C(23)-O(7) -178.9(3)

C(27)-O(8)-C(23)-C(24) -62.0(4)

O(7)-C(23)-C(24)-O(10) -62.2(4)

O(8)-C(23)-C(24)-O(10) 179.1(3)

O(7)-C(23)-C(24)-C(25) 174.4(3)

O(8)-C(23)-C(24)-C(25) 55.7(4)

O(10)-C(24)-C(25)-C(26) -174.2(3)

C(23)-C(24)-C(25)-C(26) -52.6(4)

O(10)-C(24)-C(25)-N(1) 58.6(4)

C(23)-C(24)-C(25)-N(1) -179.8(3)

O(9)-N(1)-C(25)-C(26) 170.9(3)

C(28)-N(1)-C(25)-C(26) -68.1(4)

C(29)-N(1)-C(25)-C(26) 54.7(5)

O(9)-N(1)-C(25)-C(24) -63.5(4)

C(28)-N(1)-C(25)-C(24) 57.5(4)

C(29)-N(1)-C(25)-C(24) -179.7(4)

C(24)-C(25)-C(26)-C(27) 54.5(4)

N(1)-C(25)-C(26)-C(27) 179.8(3)

C(23)-O(8)-C(27)-C(30) -174.7(3)

C(23)-O(8)-C(27)-C(26) 63.0(4)

C(25)-C(26)-C(27)-O(8) -58.0(4)

C(25)-C(26)-C(27)-C(30) -176.0(4)

O(1)-C(13)-C(36)-C(37) 66.0(4)

C(12)-C(13)-C(36)-C(37) -172.4(3)

________________________________________________________________

Symmetry transformations used to generate equivalent atoms:

Table 7. Hydrogen bonds for 007b-24081 [Å and °].

____________________________________________________________________________

D-H...A d(D-H) d(H...A) d(D...A) <(DHA)

____________________________________________________________________________

O(6)-H(6)...O(9)#1 0.84 1.80 2.633(4) 173.0

O(10)-H(10)...O(9) 0.84 1.82 2.571(4) 148.3

O(10)-H(10)...N(1) 0.84 2.48 2.933(4) 114.5

O(11)-H(11)...O(4) 0.84 2.00 2.814(4) 162.6

O(13)-H(13)...O(2)#2 0.84 2.10 2.914(4) 162.8

____________________________________________________________________________

Symmetry transformations used to generate equivalent atoms:

#1 x,y+1,z #2 x-1/2,-y+3/2,-z+1
