# Supplementary material for: Site-Divergent Oxidations within Venerable Macrolide Antibiotic Scaffolds Unveil Compounds with Broad Spectrum and Anti-MRSA Activities
Source: ACS Cent Sci. 2026 Mar 17;12(3):375–82. doi: 10.1021/acscentsci.5c02343 (PMC13022725; doi:10.1021/acscentsci.5c02343)
Supplement: Supplementary file 5 [file oc5c02343_si_005.zip › Biological, Computational, and X-ray Data/X-Ray/15/007b-25056.docx]

***Experimental***

Low-temperature diffraction data (ω-scans) were collected on a Rigaku MicroMax-007HF diffractometer coupled to a Saturn994+ CCD detector with Cu Kα (λ = 1.54178 Å) for the structure of 007b-25056. The diffraction images were processed and scaled using Rigaku Oxford Diffraction software (CrysAlisPro; Rigaku OD: The Woodlands, TX, 2015). The structure was solved with SHELXT and was refined against F^2^ on all data by full-matrix least squares with SHELXL (Sheldrick, G. M. Acta Cryst. 2008, A64, 112–122). All non-hydrogen atoms were refined anisotropically. Hydrogen atoms were included in the model at geometrically calculated positions and refined using a riding model. The isotropic displacement parameters of all hydrogen atoms were fixed to 1.2 times the U value of the atoms to which they are linked (1.5 times for methyl groups). The full numbering scheme of compound 007b-25056 can be found in the full details of the X-ray structure determination (CIF), which is included as Supporting Information. CCDC number XXXXXX (007b-25056) contains the supplementary crystallographic data for this paper. These data can be obtained free of charge from The Cambridge Crystallographic Data Center via www.ccdc.cam.ac.uk/data_request/cif.


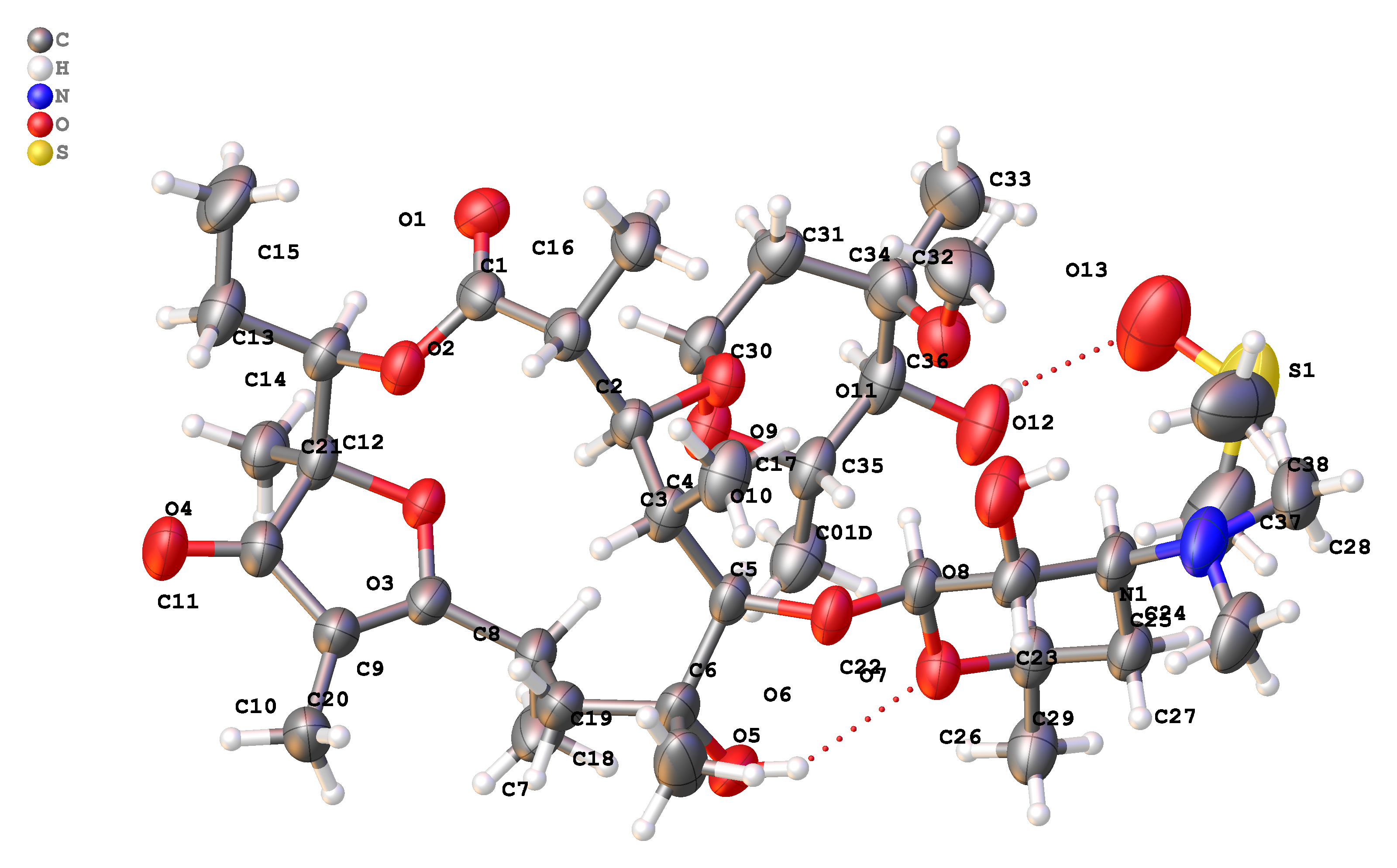


Figure 1. The complete numbering scheme of 007b-25056 with 50% thermal ellipsoid probability levels. The hydrogen atoms are shown as circles for clarity.

Table 1. Crystal data and structure refinement for 007b-25056.

Identification code 007b-25056

Empirical formula C39 H69 N O13 S

Formula weight 792.01

Temperature 100 K

Wavelength 1.54184 Å

Crystal system Orthorhombic

Space group P2**_1_**2**_1_**2**_1_**

Unit cell dimensions a = 10.08166(17) Å α= 90°.

b = 13.7632(2) Å β= 90°.

c = 30.2820(4) Å γ = 90°.

Volume 4201.81(12) Å3

Z 4

Density (calculated) 1.252 Mg/m3

Absorption coefficient 1.204 mm-1

F(000) 1720

Crystal size 0.23 x 0.1 x 0.08 mm3

Crystal color and habit colourless prism

Diffractometer XtaLAB AFC11 (RCD3): quarter-chi single

Theta range for data collection 3.527 to 68.922°.

Index ranges -12<=h<=12, -16<=k<=16, -36<=l<=36

Reflections collected 99976

Independent reflections 7690 [R(int) = 0.1892]

Observed reflections (I > 2sigma(I)) 7289

Completeness to theta = 67.684° 99.6 %

Absorption correction Semi-empirical from equivalents

Max. and min. transmission 1.00000 and 0.84689

Solution method SHELXT (Sheldrick, 2015)

Refinement method SHELXL 2019/3 (Sheldrick, 2015)

Data / restraints / parameters 7690 / 64 / 505

Goodness-of-fit on F2 1.052

Final R indices [I>2sigma(I)] R1 = 0.0705, wR2 = 0.1979

R indices (all data) R1 = 0.0762, wR2 = 0.2051

Absolute structure parameter 0.03(2)

Largest diff. peak and hole 0.516 and -0.754 e.Å-3

Table 2. Atomic coordinates ( x 104) and equivalent isotropic displacement parameters (Å2x 103)

for 007b-25056. U(eq) is defined as one third of the trace of the orthogonalized Uij tensor.

________________________________________________________________________________

x y z U(eq)

________________________________________________________________________________

S(1) 4049(4) 7124(2) 5206(1) 123(1)

O(13) 3594(9) 7542(5) 4774(2) 127(2)

C(37) 5741(10) 7283(7) 5207(3) 126(3)

C(38) 4045(9) 5906(7) 5153(3) 127(3)

O(1) 3569(3) 4660(2) 1911(1) 54(1)

O(2) 5573(3) 4063(2) 1729(1) 46(1)

O(3) 7442(3) 5302(2) 1962(1) 47(1)

O(4) 8533(4) 4333(3) 945(1) 72(1)

O(5) 9563(4) 3948(4) 3455(1) 78(1)

O(6) 7162(3) 2968(2) 3692(1) 49(1)

O(7) 7767(3) 4103(2) 4191(1) 51(1)

O(8) 5031(4) 2353(3) 4246(1) 68(1)

O(9) 4861(3) 4329(2) 3043(1) 42(1)

O(10) 5830(3) 5881(2) 3046(1) 51(1)

O(11) 3847(4) 5055(3) 3905(1) 59(1)

O(12) 5161(5) 6705(3) 4168(1) 75(1)

N(1) 5162(5) 2845(4) 5128(1) 73(1)

C(1) 4465(4) 4101(3) 1976(1) 46(1)

C(01D) 7311(6) 6634(4) 3547(2) 65(1)

C(2) 4549(4) 3388(3) 2356(1) 44(1)

C(3) 5548(4) 3788(3) 2708(1) 41(1)

C(4) 6393(5) 2984(3) 2924(1) 46(1)

C(5) 7351(4) 3435(3) 3270(1) 45(1)

C(6) 8839(5) 3335(5) 3160(2) 62(1)

C(7) 9205(5) 3665(5) 2689(2) 62(1)

C(8) 8845(4) 4714(4) 2552(1) 51(1)

C(9) 8502(4) 4725(4) 2066(1) 49(1)

C(10) 9068(5) 4271(4) 1719(2) 57(1)

C(11) 8309(5) 4543(4) 1336(2) 55(1)

C(12) 7213(5) 5217(3) 1489(1) 47(1)

C(13) 5790(5) 4865(3) 1419(1) 47(1)

C(14) 5504(6) 4528(4) 947(2) 63(1)

C(15) 4060(7) 4349(6) 858(2) 79(2)

C(16) 3169(5) 3181(4) 2541(2) 55(1)

C(17) 5581(6) 2142(4) 3114(2) 61(1)

C(18) 9266(7) 2265(6) 3212(2) 87(2)

C(19) 9944(6) 5447(6) 2648(2) 78(2)

C(20) 10276(6) 3641(6) 1690(2) 73(2)

C(21) 7433(5) 6219(4) 1280(2) 56(1)

C(22) 6669(5) 3542(3) 4034(1) 48(1)

C(23) 6156(5) 2885(4) 4401(2) 54(1)

C(24) 5796(5) 3488(4) 4800(1) 56(1)

C(25) 6985(5) 4099(4) 4948(1) 55(1)

C(26) 7470(5) 4716(4) 4563(1) 54(1)

C(27) 6049(8) 2173(5) 5338(2) 82(2)

C(28) 4429(7) 3420(7) 5460(2) 90(2)

C(29) 8720(6) 5262(4) 4664(2) 65(1)

C(30) 4698(5) 5332(3) 2939(1) 48(1)

C(31) 3454(6) 5714(4) 3156(2) 59(1)

C(32) 3594(6) 5926(4) 3655(2) 62(1)

C(33) 2363(7) 6451(6) 3821(2) 86(2)

C(34) 2841(6) 4329(5) 3896(3) 81(2)

C(35) 6044(5) 6045(3) 3512(1) 51(1)

C(36) 4858(6) 6535(4) 3715(2) 58(1)

________________________________________________________________________________ Table 3. Bond lengths [Å] and angles [°] for 007b-25056.

_____________________________________________________

S(1)-O(13) 1.501(6)

S(1)-C(37) 1.719(10)

S(1)-C(38) 1.684(9)

C(37)-H(37A) 0.9800

C(37)-H(37B) 0.9800

C(37)-H(37C) 0.9800

C(38)-H(38A) 0.9800

C(38)-H(38B) 0.9800

C(38)-H(38C) 0.9800

O(1)-C(1) 1.203(6)

O(2)-C(1) 1.346(5)

O(2)-C(13) 1.466(5)

O(3)-C(9) 1.367(6)

O(3)-C(12) 1.455(5)

O(4)-C(11) 1.241(6)

O(5)-H(5) 0.8400

O(5)-C(6) 1.429(7)

O(6)-C(5) 1.443(5)

O(6)-C(22) 1.394(5)

O(7)-C(22) 1.430(6)

O(7)-C(26) 1.441(6)

O(8)-H(8) 0.8400

O(8)-C(23) 1.429(6)

O(9)-C(3) 1.437(5)

O(9)-C(30) 1.426(5)

O(10)-C(30) 1.407(6)

O(10)-C(35) 1.445(5)

O(11)-C(32) 1.440(7)

O(11)-C(34) 1.424(7)

O(12)-H(12) 0.8400

O(12)-C(36) 1.424(5)

N(1)-C(24) 1.477(6)

N(1)-C(27) 1.435(9)

N(1)-C(28) 1.479(10)

C(1)-C(2) 1.514(6)

C(01D)-H(01A) 0.9800

C(01D)-H(01B) 0.9800

C(01D)-H(01C) 0.9800

C(01D)-C(35) 1.517(7)

C(2)-H(2) 1.0000

C(2)-C(3) 1.568(6)

C(2)-C(16) 1.526(6)

C(3)-H(3) 1.0000

C(3)-C(4) 1.542(6)

C(4)-H(4) 1.0000

C(4)-C(5) 1.553(6)

C(4)-C(17) 1.530(7)

C(5)-H(5A) 1.0000

C(5)-C(6) 1.542(7)

C(6)-C(7) 1.542(7)

C(6)-C(18) 1.544(9)

C(7)-H(7A) 0.9900

C(7)-H(7B) 0.9900

C(7)-C(8) 1.546(8)

C(8)-H(8A) 1.0000

C(8)-C(9) 1.511(6)

C(8)-C(19) 1.528(8)

C(9)-C(10) 1.349(7)

C(10)-C(11) 1.440(7)

C(10)-C(20) 1.497(7)

C(11)-C(12) 1.516(7)

C(12)-C(13) 1.529(7)

C(12)-C(21) 1.533(6)

C(13)-H(13) 1.0000

C(13)-C(14) 1.530(6)

C(14)-H(14A) 0.9900

C(14)-H(14B) 0.9900

C(14)-C(15) 1.501(9)

C(15)-H(15A) 0.9800

C(15)-H(15B) 0.9800

C(15)-H(15C) 0.9800

C(16)-H(16A) 0.9800

C(16)-H(16B) 0.9800

C(16)-H(16C) 0.9800

C(17)-H(17A) 0.9800

C(17)-H(17B) 0.9800

C(17)-H(17C) 0.9800

C(18)-H(18A) 0.9800

C(18)-H(18B) 0.9800

C(18)-H(18C) 0.9800

C(19)-H(19A) 0.9800

C(19)-H(19B) 0.9800

C(19)-H(19C) 0.9800

C(20)-H(20A) 0.9800

C(20)-H(20B) 0.9800

C(20)-H(20C) 0.9800

C(21)-H(21A) 0.9800

C(21)-H(21B) 0.9800

C(21)-H(21C) 0.9800

C(22)-H(22) 1.0000

C(22)-C(23) 1.525(6)

C(23)-H(23) 1.0000

C(23)-C(24) 1.510(7)

C(24)-H(24) 1.0000

C(24)-C(25) 1.531(7)

C(25)-H(25A) 0.9900

C(25)-H(25B) 0.9900

C(25)-C(26) 1.524(6)

C(26)-H(26) 1.0000

C(26)-C(29) 1.498(8)

C(27)-H(27A) 0.9800

C(27)-H(27B) 0.9800

C(27)-H(27C) 0.9800

C(28)-H(28A) 0.9800

C(28)-H(28B) 0.9800

C(28)-H(28C) 0.9800

C(29)-H(29A) 0.9800

C(29)-H(29B) 0.9800

C(29)-H(29C) 0.9800

C(30)-H(30) 1.0000

C(30)-C(31) 1.510(7)

C(31)-H(31A) 0.9900

C(31)-H(31B) 0.9900

C(31)-C(32) 1.544(7)

C(32)-C(33) 1.521(8)

C(32)-C(36) 1.536(8)

C(33)-H(33A) 0.9800

C(33)-H(33B) 0.9800

C(33)-H(33C) 0.9800

C(34)-H(34A) 0.9800

C(34)-H(34B) 0.9800

C(34)-H(34C) 0.9800

C(35)-H(35) 1.0000

C(35)-C(36) 1.506(8)

C(36)-H(36) 1.0000

O(13)-S(1)-C(37) 104.8(5)

O(13)-S(1)-C(38) 107.4(4)

C(38)-S(1)-C(37) 97.4(5)

S(1)-C(37)-H(37A) 109.5

S(1)-C(37)-H(37B) 109.5

S(1)-C(37)-H(37C) 109.5

H(37A)-C(37)-H(37B) 109.5

H(37A)-C(37)-H(37C) 109.5

H(37B)-C(37)-H(37C) 109.5

S(1)-C(38)-H(38A) 109.5

S(1)-C(38)-H(38B) 109.5

S(1)-C(38)-H(38C) 109.5

H(38A)-C(38)-H(38B) 109.5

H(38A)-C(38)-H(38C) 109.5

H(38B)-C(38)-H(38C) 109.5

C(1)-O(2)-C(13) 116.8(3)

C(9)-O(3)-C(12) 107.7(3)

C(6)-O(5)-H(5) 109.5

C(22)-O(6)-C(5) 116.9(3)

C(22)-O(7)-C(26) 114.6(4)

C(23)-O(8)-H(8) 109.5

C(30)-O(9)-C(3) 113.7(3)

C(30)-O(10)-C(35) 115.4(3)

C(34)-O(11)-C(32) 116.6(5)

C(36)-O(12)-H(12) 109.5

C(24)-N(1)-C(28) 110.7(5)

C(27)-N(1)-C(24) 114.6(5)

C(27)-N(1)-C(28) 110.8(5)

O(1)-C(1)-O(2) 123.8(4)

O(1)-C(1)-C(2) 125.5(4)

O(2)-C(1)-C(2) 110.6(4)

H(01A)-C(01D)-H(01B) 109.5

H(01A)-C(01D)-H(01C) 109.5

H(01B)-C(01D)-H(01C) 109.5

C(35)-C(01D)-H(01A) 109.5

C(35)-C(01D)-H(01B) 109.5

C(35)-C(01D)-H(01C) 109.5

C(1)-C(2)-H(2) 107.9

C(1)-C(2)-C(3) 108.9(3)

C(1)-C(2)-C(16) 110.5(4)

C(3)-C(2)-H(2) 107.9

C(16)-C(2)-H(2) 107.9

C(16)-C(2)-C(3) 113.6(3)

O(9)-C(3)-C(2) 110.7(3)

O(9)-C(3)-H(3) 107.7

O(9)-C(3)-C(4) 109.8(3)

C(2)-C(3)-H(3) 107.7

C(4)-C(3)-C(2) 113.1(3)

C(4)-C(3)-H(3) 107.7

C(3)-C(4)-H(4) 106.6

C(3)-C(4)-C(5) 110.0(3)

C(5)-C(4)-H(4) 106.6

C(17)-C(4)-C(3) 114.0(4)

C(17)-C(4)-H(4) 106.6

C(17)-C(4)-C(5) 112.5(4)

O(6)-C(5)-C(4) 109.7(4)

O(6)-C(5)-H(5A) 108.6

O(6)-C(5)-C(6) 106.2(3)

C(4)-C(5)-H(5A) 108.6

C(6)-C(5)-C(4) 115.1(4)

C(6)-C(5)-H(5A) 108.6

O(5)-C(6)-C(5) 108.1(4)

O(5)-C(6)-C(7) 106.4(5)

O(5)-C(6)-C(18) 110.9(5)

C(5)-C(6)-C(18) 109.5(5)

C(7)-C(6)-C(5) 113.9(4)

C(7)-C(6)-C(18) 107.9(5)

C(6)-C(7)-H(7A) 107.8

C(6)-C(7)-H(7B) 107.8

C(6)-C(7)-C(8) 117.9(5)

H(7A)-C(7)-H(7B) 107.2

C(8)-C(7)-H(7A) 107.8

C(8)-C(7)-H(7B) 107.8

C(7)-C(8)-H(8A) 108.1

C(9)-C(8)-C(7) 109.0(4)

C(9)-C(8)-H(8A) 108.1

C(9)-C(8)-C(19) 110.2(4)

C(19)-C(8)-C(7) 113.3(5)

C(19)-C(8)-H(8A) 108.1

O(3)-C(9)-C(8) 114.1(4)

C(10)-C(9)-O(3) 114.9(4)

C(10)-C(9)-C(8) 131.0(4)

C(9)-C(10)-C(11) 106.3(4)

C(9)-C(10)-C(20) 131.2(4)

C(11)-C(10)-C(20) 122.4(4)

O(4)-C(11)-C(10) 127.7(5)

O(4)-C(11)-C(12) 124.6(4)

C(10)-C(11)-C(12) 107.5(4)

O(3)-C(12)-C(11) 103.6(3)

O(3)-C(12)-C(13) 108.2(3)

O(3)-C(12)-C(21) 108.2(4)

C(11)-C(12)-C(13) 116.6(4)

C(11)-C(12)-C(21) 108.6(4)

C(13)-C(12)-C(21) 111.3(4)

O(2)-C(13)-C(12) 106.8(3)

O(2)-C(13)-H(13) 108.7

O(2)-C(13)-C(14) 109.9(4)

C(12)-C(13)-H(13) 108.7

C(12)-C(13)-C(14) 113.8(4)

C(14)-C(13)-H(13) 108.7

C(13)-C(14)-H(14A) 108.8

C(13)-C(14)-H(14B) 108.8

H(14A)-C(14)-H(14B) 107.7

C(15)-C(14)-C(13) 113.6(5)

C(15)-C(14)-H(14A) 108.8

C(15)-C(14)-H(14B) 108.8

C(14)-C(15)-H(15A) 109.5

C(14)-C(15)-H(15B) 109.5

C(14)-C(15)-H(15C) 109.5

H(15A)-C(15)-H(15B) 109.5

H(15A)-C(15)-H(15C) 109.5

H(15B)-C(15)-H(15C) 109.5

C(2)-C(16)-H(16A) 109.5

C(2)-C(16)-H(16B) 109.5

C(2)-C(16)-H(16C) 109.5

H(16A)-C(16)-H(16B) 109.5

H(16A)-C(16)-H(16C) 109.5

H(16B)-C(16)-H(16C) 109.5

C(4)-C(17)-H(17A) 109.5

C(4)-C(17)-H(17B) 109.5

C(4)-C(17)-H(17C) 109.5

H(17A)-C(17)-H(17B) 109.5

H(17A)-C(17)-H(17C) 109.5

H(17B)-C(17)-H(17C) 109.5

C(6)-C(18)-H(18A) 109.5

C(6)-C(18)-H(18B) 109.5

C(6)-C(18)-H(18C) 109.5

H(18A)-C(18)-H(18B) 109.5

H(18A)-C(18)-H(18C) 109.5

H(18B)-C(18)-H(18C) 109.5

C(8)-C(19)-H(19A) 109.5

C(8)-C(19)-H(19B) 109.5

C(8)-C(19)-H(19C) 109.5

H(19A)-C(19)-H(19B) 109.5

H(19A)-C(19)-H(19C) 109.5

H(19B)-C(19)-H(19C) 109.5

C(10)-C(20)-H(20A) 109.5

C(10)-C(20)-H(20B) 109.5

C(10)-C(20)-H(20C) 109.5

H(20A)-C(20)-H(20B) 109.5

H(20A)-C(20)-H(20C) 109.5

H(20B)-C(20)-H(20C) 109.5

C(12)-C(21)-H(21A) 109.5

C(12)-C(21)-H(21B) 109.5

C(12)-C(21)-H(21C) 109.5

H(21A)-C(21)-H(21B) 109.5

H(21A)-C(21)-H(21C) 109.5

H(21B)-C(21)-H(21C) 109.5

O(6)-C(22)-O(7) 106.0(4)

O(6)-C(22)-H(22) 110.6

O(6)-C(22)-C(23) 109.0(4)

O(7)-C(22)-H(22) 110.6

O(7)-C(22)-C(23) 109.9(3)

C(23)-C(22)-H(22) 110.6

O(8)-C(23)-C(22) 109.5(4)

O(8)-C(23)-H(23) 108.9

O(8)-C(23)-C(24) 110.7(4)

C(22)-C(23)-H(23) 108.9

C(24)-C(23)-C(22) 109.8(4)

C(24)-C(23)-H(23) 108.9

N(1)-C(24)-C(23) 108.2(5)

N(1)-C(24)-H(24) 106.5

N(1)-C(24)-C(25) 118.0(4)

C(23)-C(24)-H(24) 106.5

C(23)-C(24)-C(25) 110.4(4)

C(25)-C(24)-H(24) 106.5

C(24)-C(25)-H(25A) 109.8

C(24)-C(25)-H(25B) 109.8

H(25A)-C(25)-H(25B) 108.2

C(26)-C(25)-C(24) 109.4(4)

C(26)-C(25)-H(25A) 109.8

C(26)-C(25)-H(25B) 109.8

O(7)-C(26)-C(25) 109.8(4)

O(7)-C(26)-H(26) 109.2

O(7)-C(26)-C(29) 106.1(4)

C(25)-C(26)-H(26) 109.2

C(29)-C(26)-C(25) 113.2(4)

C(29)-C(26)-H(26) 109.2

N(1)-C(27)-H(27A) 109.5

N(1)-C(27)-H(27B) 109.5

N(1)-C(27)-H(27C) 109.5

H(27A)-C(27)-H(27B) 109.5

H(27A)-C(27)-H(27C) 109.5

H(27B)-C(27)-H(27C) 109.5

N(1)-C(28)-H(28A) 109.5

N(1)-C(28)-H(28B) 109.5

N(1)-C(28)-H(28C) 109.5

H(28A)-C(28)-H(28B) 109.5

H(28A)-C(28)-H(28C) 109.5

H(28B)-C(28)-H(28C) 109.5

C(26)-C(29)-H(29A) 109.5

C(26)-C(29)-H(29B) 109.5

C(26)-C(29)-H(29C) 109.5

H(29A)-C(29)-H(29B) 109.5

H(29A)-C(29)-H(29C) 109.5

H(29B)-C(29)-H(29C) 109.5

O(9)-C(30)-H(30) 107.4

O(9)-C(30)-C(31) 109.7(4)

O(10)-C(30)-O(9) 112.1(3)

O(10)-C(30)-H(30) 107.4

O(10)-C(30)-C(31) 112.7(4)

C(31)-C(30)-H(30) 107.4

C(30)-C(31)-H(31A) 108.6

C(30)-C(31)-H(31B) 108.6

C(30)-C(31)-C(32) 114.6(4)

H(31A)-C(31)-H(31B) 107.6

C(32)-C(31)-H(31A) 108.6

C(32)-C(31)-H(31B) 108.6

O(11)-C(32)-C(31) 111.9(4)

O(11)-C(32)-C(33) 111.4(5)

O(11)-C(32)-C(36) 104.1(4)

C(33)-C(32)-C(31) 109.8(5)

C(33)-C(32)-C(36) 112.2(5)

C(36)-C(32)-C(31) 107.2(4)

C(32)-C(33)-H(33A) 109.5

C(32)-C(33)-H(33B) 109.5

C(32)-C(33)-H(33C) 109.5

H(33A)-C(33)-H(33B) 109.5

H(33A)-C(33)-H(33C) 109.5

H(33B)-C(33)-H(33C) 109.5

O(11)-C(34)-H(34A) 109.5

O(11)-C(34)-H(34B) 109.5

O(11)-C(34)-H(34C) 109.5

H(34A)-C(34)-H(34B) 109.5

H(34A)-C(34)-H(34C) 109.5

H(34B)-C(34)-H(34C) 109.5

O(10)-C(35)-C(01D) 106.2(4)

O(10)-C(35)-H(35) 108.8

O(10)-C(35)-C(36) 110.5(4)

C(01D)-C(35)-H(35) 108.8

C(36)-C(35)-C(01D) 113.6(4)

C(36)-C(35)-H(35) 108.8

O(12)-C(36)-C(32) 112.5(5)

O(12)-C(36)-C(35) 107.3(5)

O(12)-C(36)-H(36) 108.5

C(32)-C(36)-H(36) 108.5

C(35)-C(36)-C(32) 111.5(4)

C(35)-C(36)-H(36) 108.5

_____________________________________________________________

Table 4. Anisotropic displacement parameters (Å2x 103) for 007b-25056. The anisotropic

displacement factor exponent takes the form: -2π2[ h2 a*2U11 + ... + 2 h k a* b* U12 ]

______________________________________________________________________________

U11 U22 U33 U23 U13 U12

______________________________________________________________________________

S(1) 189(3) 113(2) 66(1) -10(1) -6(1) 38(2)

O(13) 184(6) 112(4) 84(3) -1(3) -12(4) 52(4)

C(37) 201(9) 96(5) 82(5) 4(4) -42(6) -3(6)

C(38) 93(5) 170(8) 117(6) 77(6) -8(5) -7(5)

O(1) 50(2) 61(2) 50(2) -1(2) -7(1) 5(2)

O(2) 54(2) 51(2) 32(1) 1(1) -2(1) 4(1)

O(3) 46(1) 65(2) 29(1) 0(1) -4(1) 0(1)

O(4) 80(2) 103(3) 34(2) -1(2) 2(2) 28(2)

O(5) 50(2) 147(4) 37(2) 8(2) -10(2) -1(2)

O(6) 65(2) 54(2) 28(1) 5(1) 0(1) 7(1)

O(7) 60(2) 58(2) 34(1) -1(1) 2(1) -1(2)

O(8) 86(3) 80(2) 39(2) 9(2) -3(2) -25(2)

O(9) 53(2) 40(1) 32(1) -3(1) 0(1) 0(1)

O(10) 71(2) 48(2) 36(2) 1(1) -5(1) -7(1)

O(11) 63(2) 65(2) 49(2) -1(2) 5(2) -4(2)

O(12) 118(4) 69(2) 39(2) -9(2) -10(2) -8(2)

N(1) 80(3) 101(3) 38(2) 14(2) 0(2) -29(3)

C(1) 45(2) 55(2) 38(2) -10(2) -4(2) -2(2)

C(01D) 86(4) 61(3) 48(2) 4(2) -10(3) -21(3)

C(2) 50(2) 47(2) 36(2) -5(2) -1(2) 0(2)

C(3) 46(2) 45(2) 31(2) -2(2) -1(2) 4(2)

C(4) 61(3) 45(2) 32(2) 1(2) -4(2) 7(2)

C(5) 52(2) 55(2) 28(2) 6(2) -1(2) 8(2)

C(6) 59(3) 94(4) 33(2) 8(2) -3(2) 23(3)

C(7) 48(2) 99(4) 40(2) 6(2) -1(2) 14(2)

C(8) 44(2) 80(3) 29(2) 2(2) -1(2) -2(2)

C(9) 41(2) 73(3) 33(2) 5(2) -5(2) -7(2)

C(10) 48(2) 86(3) 37(2) 4(2) 0(2) 10(2)

C(11) 57(2) 76(3) 32(2) -2(2) 1(2) 7(2)

C(12) 60(2) 55(2) 26(2) 0(2) -3(2) 5(2)

C(13) 55(2) 54(2) 34(2) 5(2) -7(2) 1(2)

C(14) 83(3) 71(3) 35(2) 1(2) -8(2) -10(3)

C(15) 89(4) 101(4) 47(3) -7(3) -26(3) -11(4)

C(16) 57(3) 67(3) 42(2) -6(2) 0(2) -12(2)

C(17) 93(4) 46(2) 45(2) 3(2) -4(3) -8(2)

C(18) 93(4) 115(5) 53(3) 23(3) 12(3) 55(4)

C(19) 61(3) 126(5) 46(3) 4(3) -9(2) -27(3)

C(20) 58(3) 118(5) 43(2) 6(3) 5(2) 25(3)

C(21) 60(3) 69(3) 39(2) 10(2) -1(2) -7(2)

C(22) 59(2) 57(2) 29(2) 4(2) 1(2) 2(2)

C(23) 65(3) 64(3) 34(2) 9(2) -7(2) -7(2)

C(24) 62(3) 78(3) 29(2) 10(2) 0(2) -9(2)

C(25) 64(3) 68(3) 33(2) 5(2) -2(2) -3(2)

C(26) 68(3) 60(3) 35(2) 1(2) 3(2) 3(2)

C(27) 117(5) 84(4) 44(3) 22(3) -12(3) -24(4)

C(28) 79(4) 147(7) 45(3) 11(4) 12(3) -20(4)

C(29) 82(3) 69(3) 43(2) 1(2) 2(2) -18(3)

C(30) 65(3) 45(2) 32(2) -3(2) -9(2) 4(2)

C(31) 69(3) 57(3) 50(3) -10(2) -14(2) 14(2)

C(32) 74(3) 63(3) 48(3) -11(2) 2(2) 12(3)

C(33) 88(4) 95(4) 74(4) -26(3) 5(3) 30(4)

C(34) 69(3) 88(4) 86(4) 0(3) 7(3) -20(3)

C(35) 73(3) 48(2) 33(2) 2(2) -7(2) -5(2)

C(36) 90(4) 50(2) 34(2) -6(2) -5(2) 3(2)

______________________________________________________________________________ Table 5. Hydrogen coordinates ( x 104) and isotropic displacement parameters (Å2x 10 3)

for 007b-25056.

________________________________________________________________________________

x y z U(eq)

________________________________________________________________________________

H(37A) 6114 7027 4932 190

H(37B) 6127 6936 5458 190

H(37C) 5946 7977 5231 190

H(38A) 3132 5665 5170 190

H(38B) 4570 5617 5392 190

H(38C) 4432 5726 4868 190

H(5) 9324 3837 3716 117

H(8) 4545 2204 4461 102

H(12) 4740 7193 4258 113

H(01A) 8039 6280 3405 98

H(01B) 7526 6740 3859 98

H(01C) 7189 7262 3400 98

H(2) 4914 2764 2238 53

H(3) 6167 4244 2554 49

H(4) 6960 2703 2686 55

H(5A) 7136 4141 3302 54

H(7A) 8771 3215 2479 75

H(7B) 10175 3584 2653 75

H(8A) 8038 4914 2721 61

H(13) 5167 5406 1494 57

H(14A) 5832 5026 738 76

H(14B) 6003 3921 890 76

H(15A) 3961 4010 575 118

H(15B) 3589 4971 846 118

H(15C) 3686 3948 1095 118

H(16A) 2715 3797 2602 83

H(16B) 3250 2808 2816 83

H(16C) 2657 2806 2325 83

H(17A) 4978 2390 3342 92

H(17B) 6180 1661 3245 92

H(17C) 5065 1837 2878 92

H(18A) 8707 1852 3025 131

H(18B) 9167 2068 3522 131

H(18C) 10196 2194 3123 131

H(19A) 10728 5289 2471 116

H(19B) 10174 5422 2962 116

H(19C) 9636 6102 2573 116

H(20A) 11001 3941 1857 109

H(20B) 10539 3572 1380 109

H(20C) 10078 3000 1814 109

H(21A) 7319 6172 959 84

H(21B) 8332 6445 1347 84

H(21C) 6786 6680 1400 84

H(22) 5947 3977 3923 58

H(23) 6870 2416 4485 65

H(24) 5099 3957 4701 68

H(25A) 6722 4524 5197 66

H(25B) 7707 3667 5051 66

H(26) 6760 5188 4479 65

H(27A) 6527 1802 5113 123

H(27B) 5539 1727 5525 123

H(27C) 6685 2532 5521 123

H(28A) 5045 3853 5615 135

H(28B) 4010 2981 5674 135

H(28C) 3744 3808 5313 135

H(29A) 9418 4802 4747 97

H(29B) 8560 5714 4908 97

H(29C) 8999 5626 4401 97

H(30) 4567 5380 2613 57

H(31A) 2735 5233 3114 70

H(31B) 3184 6320 3005 70

H(33A) 1571 6075 3742 128

H(33B) 2312 7096 3685 128

H(33C) 2412 6519 4143 128

H(34A) 2684 4126 3590 122

H(34B) 2020 4594 4021 122

H(34C) 3127 3768 4071 122

H(35) 6183 5404 3660 61

H(36) 4725 7176 3567 70

________________________________________________________________________________ Table 6. Torsion angles [°] for 007b-25056.

________________________________________________________________

O(1)-C(1)-C(2)-C(3) -103.6(5)

O(1)-C(1)-C(2)-C(16) 21.9(6)

O(2)-C(1)-C(2)-C(3) 73.3(4)

O(2)-C(1)-C(2)-C(16) -161.3(3)

O(2)-C(13)-C(14)-C(15) 70.4(6)

O(3)-C(9)-C(10)-C(11) -1.2(6)

O(3)-C(9)-C(10)-C(20) 175.6(6)

O(3)-C(12)-C(13)-O(2) -45.6(4)

O(3)-C(12)-C(13)-C(14) -167.1(4)

O(4)-C(11)-C(12)-O(3) -174.6(5)

O(4)-C(11)-C(12)-C(13) 66.8(7)

O(4)-C(11)-C(12)-C(21) -59.8(7)

O(5)-C(6)-C(7)-C(8) -62.2(5)

O(6)-C(5)-C(6)-O(5) -70.2(5)

O(6)-C(5)-C(6)-C(7) 171.8(4)

O(6)-C(5)-C(6)-C(18) 50.8(5)

O(6)-C(22)-C(23)-O(8) -66.2(5)

O(6)-C(22)-C(23)-C(24) 172.1(4)

O(7)-C(22)-C(23)-O(8) 178.0(4)

O(7)-C(22)-C(23)-C(24) 56.2(5)

O(8)-C(23)-C(24)-N(1) 52.6(5)

O(8)-C(23)-C(24)-C(25) -176.9(4)

O(9)-C(3)-C(4)-C(5) -55.6(5)

O(9)-C(3)-C(4)-C(17) 71.8(5)

O(9)-C(30)-C(31)-C(32) 77.6(5)

O(10)-C(30)-C(31)-C(32) -48.0(6)

O(10)-C(35)-C(36)-O(12) -177.8(4)

O(10)-C(35)-C(36)-C(32) 58.6(5)

O(11)-C(32)-C(36)-O(12) -56.0(5)

O(11)-C(32)-C(36)-C(35) 64.6(5)

N(1)-C(24)-C(25)-C(26) -179.1(5)

C(1)-O(2)-C(13)-C(12) 130.8(4)

C(1)-O(2)-C(13)-C(14) -105.4(5)

C(1)-C(2)-C(3)-O(9) 93.8(4)

C(1)-C(2)-C(3)-C(4) -142.6(4)

C(01D)-C(35)-C(36)-O(12) -58.6(5)

C(01D)-C(35)-C(36)-C(32) 177.8(4)

C(2)-C(3)-C(4)-C(5) -179.8(3)

C(2)-C(3)-C(4)-C(17) -52.3(5)

C(3)-O(9)-C(30)-O(10) -83.2(4)

C(3)-O(9)-C(30)-C(31) 150.8(3)

C(3)-C(4)-C(5)-O(6) 125.0(4)

C(3)-C(4)-C(5)-C(6) -115.3(4)

C(4)-C(5)-C(6)-O(5) 168.3(4)

C(4)-C(5)-C(6)-C(7) 50.3(6)

C(4)-C(5)-C(6)-C(18) -70.8(5)

C(5)-O(6)-C(22)-O(7) -78.4(4)

C(5)-O(6)-C(22)-C(23) 163.4(4)

C(5)-C(6)-C(7)-C(8) 56.7(7)

C(6)-C(7)-C(8)-C(9) -146.5(4)

C(6)-C(7)-C(8)-C(19) 90.5(6)

C(7)-C(8)-C(9)-O(3) 139.3(4)

C(7)-C(8)-C(9)-C(10) -41.2(7)

C(8)-C(9)-C(10)-C(11) 179.3(5)

C(8)-C(9)-C(10)-C(20) -3.9(10)

C(9)-O(3)-C(12)-C(11) -1.8(5)

C(9)-O(3)-C(12)-C(13) 122.5(4)

C(9)-O(3)-C(12)-C(21) -116.9(4)

C(9)-C(10)-C(11)-O(4) 175.5(6)

C(9)-C(10)-C(11)-C(12) 0.0(6)

C(10)-C(11)-C(12)-O(3) 1.1(5)

C(10)-C(11)-C(12)-C(13) -117.5(5)

C(10)-C(11)-C(12)-C(21) 115.9(4)

C(11)-C(12)-C(13)-O(2) 70.5(4)

C(11)-C(12)-C(13)-C(14) -51.0(5)

C(12)-O(3)-C(9)-C(8) -178.5(4)

C(12)-O(3)-C(9)-C(10) 2.0(5)

C(12)-C(13)-C(14)-C(15) -169.9(5)

C(13)-O(2)-C(1)-O(1) 11.7(6)

C(13)-O(2)-C(1)-C(2) -165.2(3)

C(16)-C(2)-C(3)-O(9) -29.8(5)

C(16)-C(2)-C(3)-C(4) 93.8(5)

C(17)-C(4)-C(5)-O(6) -3.3(5)

C(17)-C(4)-C(5)-C(6) 116.4(5)

C(18)-C(6)-C(7)-C(8) 178.6(5)

C(19)-C(8)-C(9)-O(3) -95.8(5)

C(19)-C(8)-C(9)-C(10) 83.7(7)

C(20)-C(10)-C(11)-O(4) -1.6(10)

C(20)-C(10)-C(11)-C(12) -177.1(5)

C(21)-C(12)-C(13)-O(2) -164.3(3)

C(21)-C(12)-C(13)-C(14) 74.2(5)

C(22)-O(6)-C(5)-C(4) -114.8(4)

C(22)-O(6)-C(5)-C(6) 120.2(4)

C(22)-O(7)-C(26)-C(25) 59.4(5)

C(22)-O(7)-C(26)-C(29) -177.9(4)

C(22)-C(23)-C(24)-N(1) 173.6(4)

C(22)-C(23)-C(24)-C(25) -55.9(5)

C(23)-C(24)-C(25)-C(26) 55.7(5)

C(24)-C(25)-C(26)-O(7) -55.6(5)

C(24)-C(25)-C(26)-C(29) -174.0(5)

C(26)-O(7)-C(22)-O(6) -177.1(3)

C(26)-O(7)-C(22)-C(23) -59.4(5)

C(27)-N(1)-C(24)-C(23) 71.1(6)

C(27)-N(1)-C(24)-C(25) -55.1(7)

C(28)-N(1)-C(24)-C(23) -162.7(5)

C(28)-N(1)-C(24)-C(25) 71.0(7)

C(30)-O(9)-C(3)-C(2) -88.2(4)

C(30)-O(9)-C(3)-C(4) 146.3(4)

C(30)-O(10)-C(35)-C(01D) 179.4(4)

C(30)-O(10)-C(35)-C(36) -57.0(5)

C(30)-C(31)-C(32)-O(11) -64.5(6)

C(30)-C(31)-C(32)-C(33) 171.2(5)

C(30)-C(31)-C(32)-C(36) 49.0(6)

C(31)-C(32)-C(36)-O(12) -174.7(4)

C(31)-C(32)-C(36)-C(35) -54.1(5)

C(33)-C(32)-C(36)-O(12) 64.6(6)

C(33)-C(32)-C(36)-C(35) -174.8(5)

C(34)-O(11)-C(32)-C(31) -62.5(6)

C(34)-O(11)-C(32)-C(33) 60.8(6)

C(34)-O(11)-C(32)-C(36) -178.0(5)

C(35)-O(10)-C(30)-O(9) -73.1(5)

C(35)-O(10)-C(30)-C(31) 51.2(5)

________________________________________________________________

Table 7. Hydrogen bonds for 007b-25056 [Å and °].

____________________________________________________________________________

D-H...A d(D-H) d(H...A) d(D...A) <(DHA)

____________________________________________________________________________

O(5)-H(5)...O(7) 0.84 2.16 2.878(5) 143.4

O(12)-H(12)...O(13) 0.84 2.00 2.681(8) 137.4

____________________________________________________________________________
