# Supplementary material for: Site-Divergent Oxidations within Venerable Macrolide Antibiotic Scaffolds Unveil Compounds with Broad Spectrum and Anti-MRSA Activities
Source: ACS Cent Sci. 2026 Mar 17;12(3):375–82. doi: 10.1021/acscentsci.5c02343 (PMC13022725; doi:10.1021/acscentsci.5c02343)

# checkCIF/PLATON report

Structure factors have been supplied for datablock(s) 007b-25056

THIS REPORT IS FOR GUIDANCE ONLY. IF USED AS PART OF A REVIEW PROCEDURE FOR PUBLICATION, IT SHOULD NOT REPLACE THE EXPERTISE OF AN EXPERIENCED CRYSTALLOGRAPHIC REFEREE.

No syntax errors found. CIF dictionary Interpreting this report

## Datablock: 007b-25056

---

```
Bond precision:    C-C = 0.0073 A           Wavelength=1.54184

Cell:              a=10.08166(17)      b=13.7632(2)      c=30.2820(4)
                   alpha=90            beta=90           gamma=90
Temperature:       100 K
```

| | Calculated | Reported |
|---|---|---|
| Volume | 4201.81(11) | 4201.81(12) |
| Space group | P 21 21 21 | P 21 21 21 |
| Hall group | P 2ac 2ab | P 2ac 2ab |
| Moiety formula | C37 H63 N O12, C2 H6 O S | C37 H63 N O12, C2 H6 O S |
| Sum formula | C39 H69 N O13 S | C39 H69 N O13 S |
| Mr | 792.01 | 792.01 |
| Dx,g cm-3 | 1.252 | 1.252 |
| Z | 4 | 4 |
| Mu (mm-1) | 1.204 | 1.204 |
| F000 | 1720.0 | 1720.0 |
| F000' | 1726.66 | |
| h,k,lmax | 12,16,36 | 12,16,36 |
| Nref | 7808[ 4384] | 7690 |
| Tmin,Tmax | 0.865,0.908 | 0.847,1.000 |
| Tmin' | 0.758 | |

```
Correction method= # Reported T Limits: Tmin=0.847 Tmax=1.000
AbsCorr = MULTI-SCAN
```

Data completeness= 1.75/0.98        Theta(max)= 68.922

R(reflections)= 0.0705( 7289)        wR2(reflections)= 0.2051( 7690)

S = 1.052        Npar= 505

---

The following ALERTS were generated. Each ALERT has the format
**test-name_ALERT_alert-type_alert-level**.
Click on the hyperlinks for more details of the test.

---

**Alert level B**

```
RINTA01_ALERT_3_B  The value of Rint is greater than 0.18
            Rint given   0.189
```

**Author Response: There was signficant icing of the sample during data collection, which affected the consistency of the integrated intensities.**

```
PLAT020_ALERT_3_B The Value of Rint is Greater Than 0.12 .........      0.189 Report
```

**Author Response: There was signficant icing of the sample during data collection, which affected the consistency of the integrated intensities.**

---

**Alert level C**

```
PLAT260_ALERT_2_C Large Average Ueq of Residue Including        S1      0.126 Check
PLAT340_ALERT_3_C Low Bond Precision on  C-C Bonds ...............    0.00732 Ang.
PLAT911_ALERT_3_C Missing FCF Refl Between Thmin & STh/L=    0.600         17 Report
                2  0  0,   1  1  0,   1  0  1,   0  2  1,   2  1  2,   0  2  2,
                0  3  2,   0  3  3,   0  4  3,   2  0  4,   0  2  4,   1  2  4,
                0  2  5,   0  0  6,   1  0  6,   1  0  9,   0  2 13,
PLAT913_ALERT_3_C Missing # of Very Strong Reflections in FCF ....         12 Note
                2  0  0,   1  1  0,   1  0  1,   0  2  1,   0  2  2,   0  3  2,
                0  3  3,   1  2  4,   0  0  6,   1  0  6,   1  0  9,   0  2 13,
```

---

**Alert level G**

```
PLAT002_ALERT_2_G Number of Distance or Angle Restraints on AtSite          4 Note
PLAT003_ALERT_2_G Number of Uiso or U(i,j)  Restrained non-H-Atoms          4 Report
PLAT007_ALERT_5_G Number of Unrefined Donor-H Atoms ..............          3 Report
              H5    H8    H12
PLAT072_ALERT_2_G SHELXL First  Parameter in WGHT  Unusually Large       0.12 Report
PLAT172_ALERT_4_G The CIF-Embedded .res File Contains DFIX Records          2 Report
PLAT173_ALERT_4_G The CIF-Embedded .res File Contains DANG Records          1 Report
PLAT176_ALERT_4_G The CIF-Embedded .res File Contains SADI Records          2 Report
PLAT178_ALERT_4_G The CIF-Embedded .res File Contains SIMU Records          1 Report
PLAT186_ALERT_4_G The CIF-Embedded .res File Contains ISOR Records          1 Report
PLAT187_ALERT_4_G The CIF-Embedded .res File Contains RIGU Records          1 Report
PLAT191_ALERT_3_G A Non-default SADI Restraint Value has been used     0.0100 Report
PLAT191_ALERT_3_G A Non-default SADI Restraint Value has been used     0.0400 Report
```

```
PLAT398_ALERT_2_G Deviating  C-O-C   Angle From 120 for O3       .      107.6 Degree
PLAT432_ALERT_2_G Short Inter X...Y Contact  O13      ..C37      .       2.89 Ang.
                                          -1/2+x,3/2-y,1-z  =      4_466 Check
PLAT720_ALERT_4_G Number of Unusual/Non-Standard Labels .........          4 Note
              C01D    H01A    H01B    H01C
PLAT791_ALERT_4_G Model has Chirality at C2        (Sohncke SpGr)          R Verify
PLAT791_ALERT_4_G Model has Chirality at C3        (Sohncke SpGr)          S Verify
PLAT791_ALERT_4_G Model has Chirality at C4        (Sohncke SpGr)          R Verify
PLAT791_ALERT_4_G Model has Chirality at C5        (Sohncke SpGr)          R Verify
PLAT791_ALERT_4_G Model has Chirality at C6        (Sohncke SpGr)          R Verify
PLAT791_ALERT_4_G Model has Chirality at C8        (Sohncke SpGr)          R Verify
PLAT791_ALERT_4_G Model has Chirality at C12       (Sohncke SpGr)          R Verify
PLAT791_ALERT_4_G Model has Chirality at C13       (Sohncke SpGr)          R Verify
PLAT791_ALERT_4_G Model has Chirality at C22       (Sohncke SpGr)          S Verify
PLAT791_ALERT_4_G Model has Chirality at C23       (Sohncke SpGr)          R Verify
PLAT791_ALERT_4_G Model has Chirality at C24       (Sohncke SpGr)          S Verify
PLAT791_ALERT_4_G Model has Chirality at C26       (Sohncke SpGr)          R Verify
PLAT791_ALERT_4_G Model has Chirality at C30       (Sohncke SpGr)          R Verify
PLAT791_ALERT_4_G Model has Chirality at C32       (Sohncke SpGr)          R Verify
PLAT791_ALERT_4_G Model has Chirality at C35       (Sohncke SpGr)          S Verify
PLAT791_ALERT_4_G Model has Chirality at C36       (Sohncke SpGr)          S Verify
PLAT860_ALERT_3_G Number of Least-Squares Restraints ............         64 Note
PLAT910_ALERT_3_G Missing FCF Reflection(s) Below Theta(Min)[Deg]=       3.53 Note
                0  0  2,
PLAT912_ALERT_4_G Missing # of FCF Reflections Above STh/L=  0.600         41 Note
PLAT933_ALERT_2_G Number of HKL-OMIT Records in Embedded .res File          2 Note
               -1  2  4,   0  2  4,
PLAT969_ALERT_5_G The 'Henn et al.' R-Factor-gap value ..........      3.538 Note
              Predicted wR2: Based on SigI**2  5.80 or SHELX Weight 19.50
PLAT978_ALERT_2_G Number C-C Bonds with Positive Residual Density.          0 Info
PLAT992_ALERT_5_G Repd & Actual _reflns_number_gt Values Differ by          4 Check
```

---

0 **ALERT level A** = Most likely a serious problem - resolve or explain
2 **ALERT level B** = A potentially serious problem, consider carefully
4 **ALERT level C** = Check. Ensure it is not caused by an omission or oversight
38 **ALERT level G** = General information/check it is not something unexpected

0 ALERT type 1 CIF construction/syntax error, inconsistent or missing data
8 ALERT type 2 Indicator that the structure model may be wrong or deficient
9 ALERT type 3 Indicator that the structure quality may be low
24 ALERT type 4 Improvement, methodology, query or suggestion
3 ALERT type 5 Informative message, check

---

---

It is advisable to attempt to resolve as many as possible of the alerts in all categories. Often the minor alerts point to easily fixed oversights, errors and omissions in your CIF or refinement strategy, so attention to these fine details can be worthwhile. It is up to the individual to critically assess their own results and, if necessary, seek expert advice.

---

**PLATON version of 17/09/2025; check.def file version of 17/09/2025**

---

# duplicate check

## No duplication found

---

**Datablock 007b-25056** - ellipsoid plot

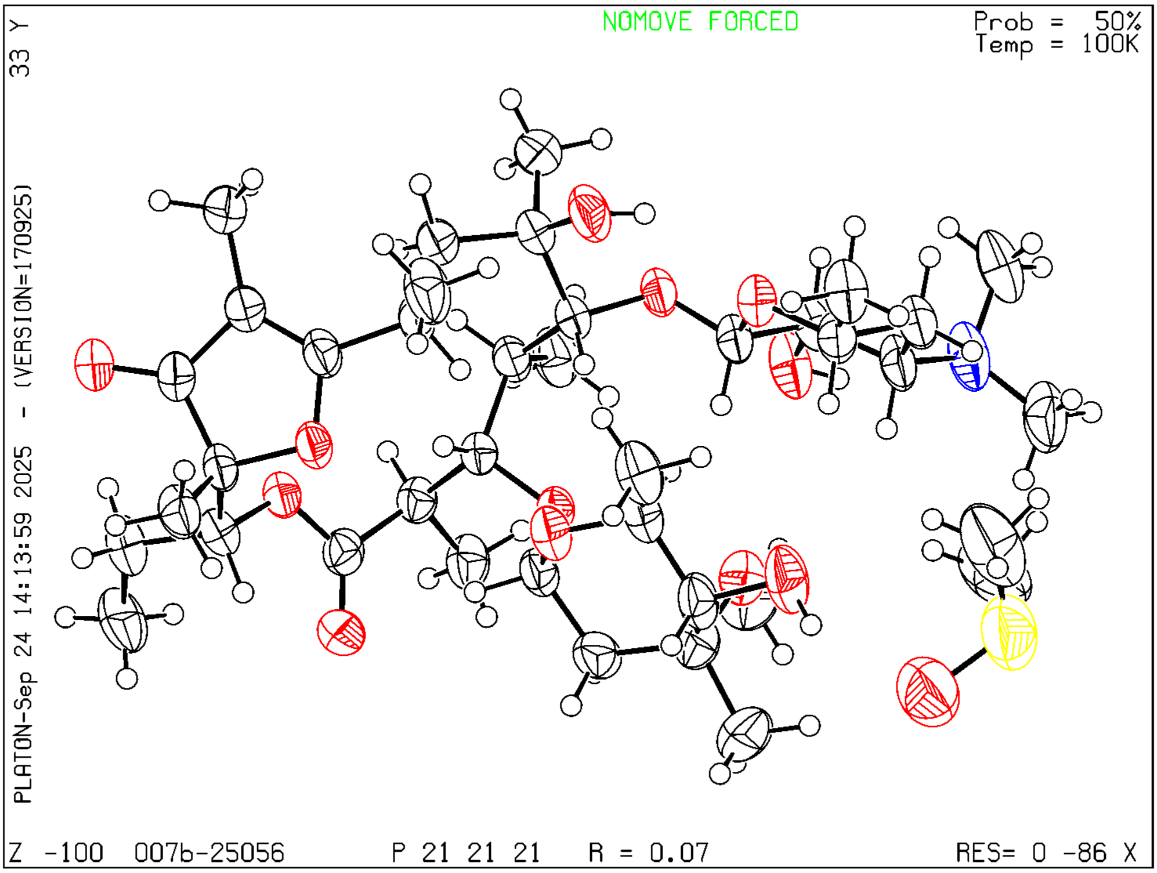

Supplement: Supplementary file 5 [file oc5c02343_si_005.zip › Biological, Computational, and X-ray Data/X-Ray/15/007b-25056 checkcif.pdf]
