# Supplementary material for: Site-Divergent Oxidations within Venerable Macrolide Antibiotic Scaffolds Unveil Compounds with Broad Spectrum and Anti-MRSA Activities
Source: ACS Cent Sci. 2026 Mar 17;12(3):375–82. doi: 10.1021/acscentsci.5c02343 (PMC13022725; doi:10.1021/acscentsci.5c02343)
Supplement: Supplementary file 6 [file oc5c02343_si_006.zip › Catalyst and SI Compound Characterization/C1 - HAzc(OMe)-Gly-OMe/IR/OL-III-026.pdf]

SHIMADZU

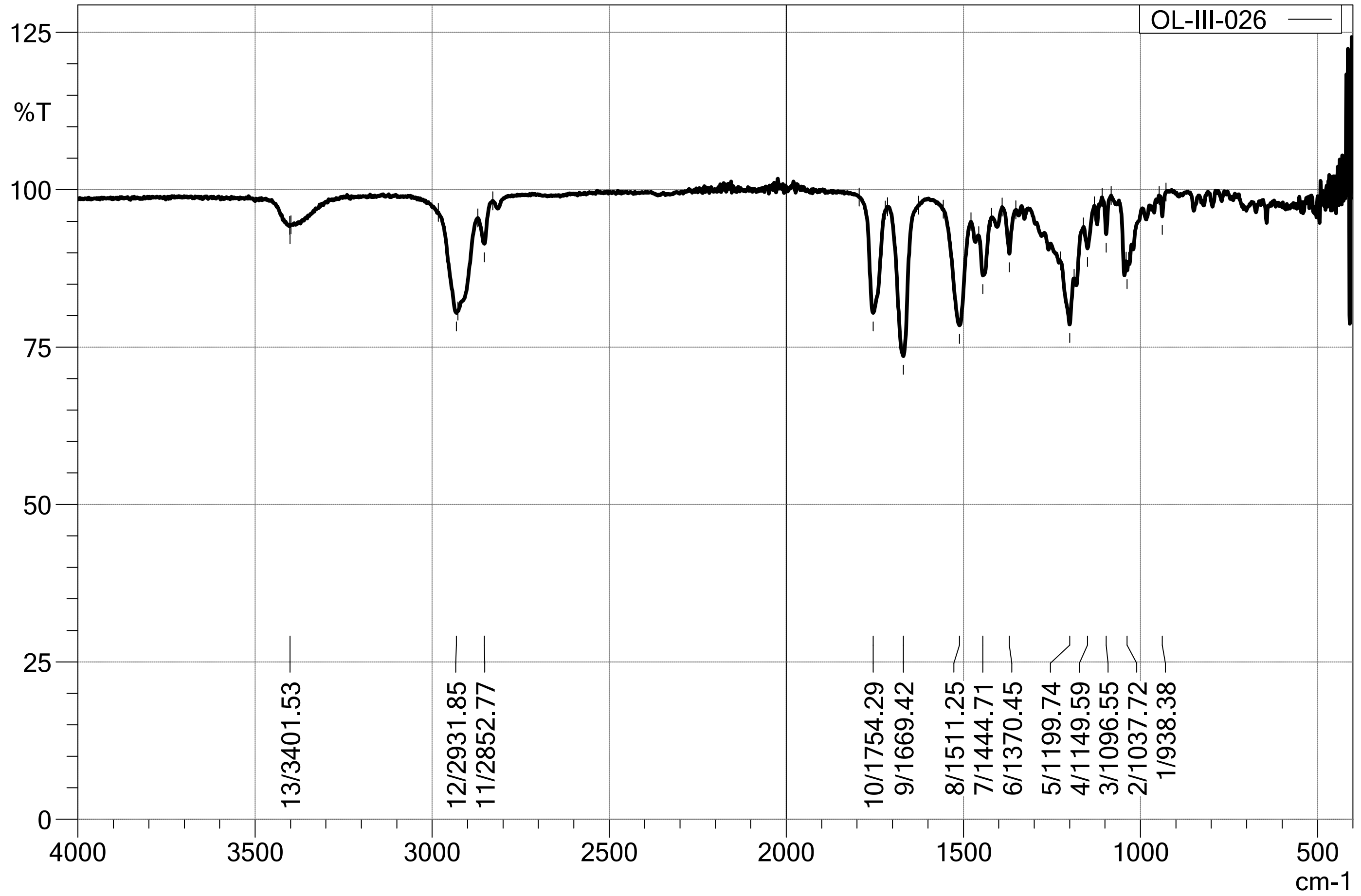

C:¥LabSolutions¥LabSolutionsIR¥Data¥Miller_OliviaL¥OL-III-026.ispd

	Item	Value
2	Sample name	
3	Sample ID	
4	Option	
5	Intensity Mode	%Transmittance
6	Apodization	Happ-Genzel
9	No. of Scans	32
10	Resolution	2 cm-1

	Peak	Intensity	Corr. Intensity	Base (H)	Base (L)	Area	Corr. Area	Comment
1	938.38	95.76	3.57	947.06	927.78	35.686	23.351	
2	1037.72	87.18	1.45	1040.61	1033.86	82.026	5.206	
3	1096.55	92.95	5.96	1108.12	1083.05	76.728	49.602	
4	1149.59	90.69	4.67	1161.17	1130.31	196.126	65.675	
5	1199.74	78.59	8.22	1225.78	1187.21	640.778	150.818	
6	1370.45	89.83	7.17	1390.70	1352.12	213.681	98.443	
7	1444.71	86.39	7.26	1456.28	1420.60	343.973	135.704	
8	1511.25	78.45	17.32	1556.58	1478.46	940.325	622.342	
9	1669.42	73.57	23.85	1713.78	1626.02	1008.202	781.629	
10	1754.29	80.45	17.28	1793.83	1720.53	700.971	540.660	
11	2852.77	91.41	5.28	2871.09	2828.66	217.203	84.794	
12	2931.85	80.45	1.65	2982.96	2927.03	636.348	-1.916	
13	3401.53	94.28	0.07	3402.49	3398.63	21.825	0.137	
