# Supplementary material for: Site-Divergent Oxidations within Venerable Macrolide Antibiotic Scaffolds Unveil Compounds with Broad Spectrum and Anti-MRSA Activities
Source: ACS Cent Sci. 2026 Mar 17;12(3):375–82. doi: 10.1021/acscentsci.5c02343 (PMC13022725; doi:10.1021/acscentsci.5c02343)
Supplement: Supplementary file 6 [file oc5c02343_si_006.zip › Catalyst and SI Compound Characterization/HAzc-OMe/IR/OL-HAzc-OMe.pdf]

SHIMADZU

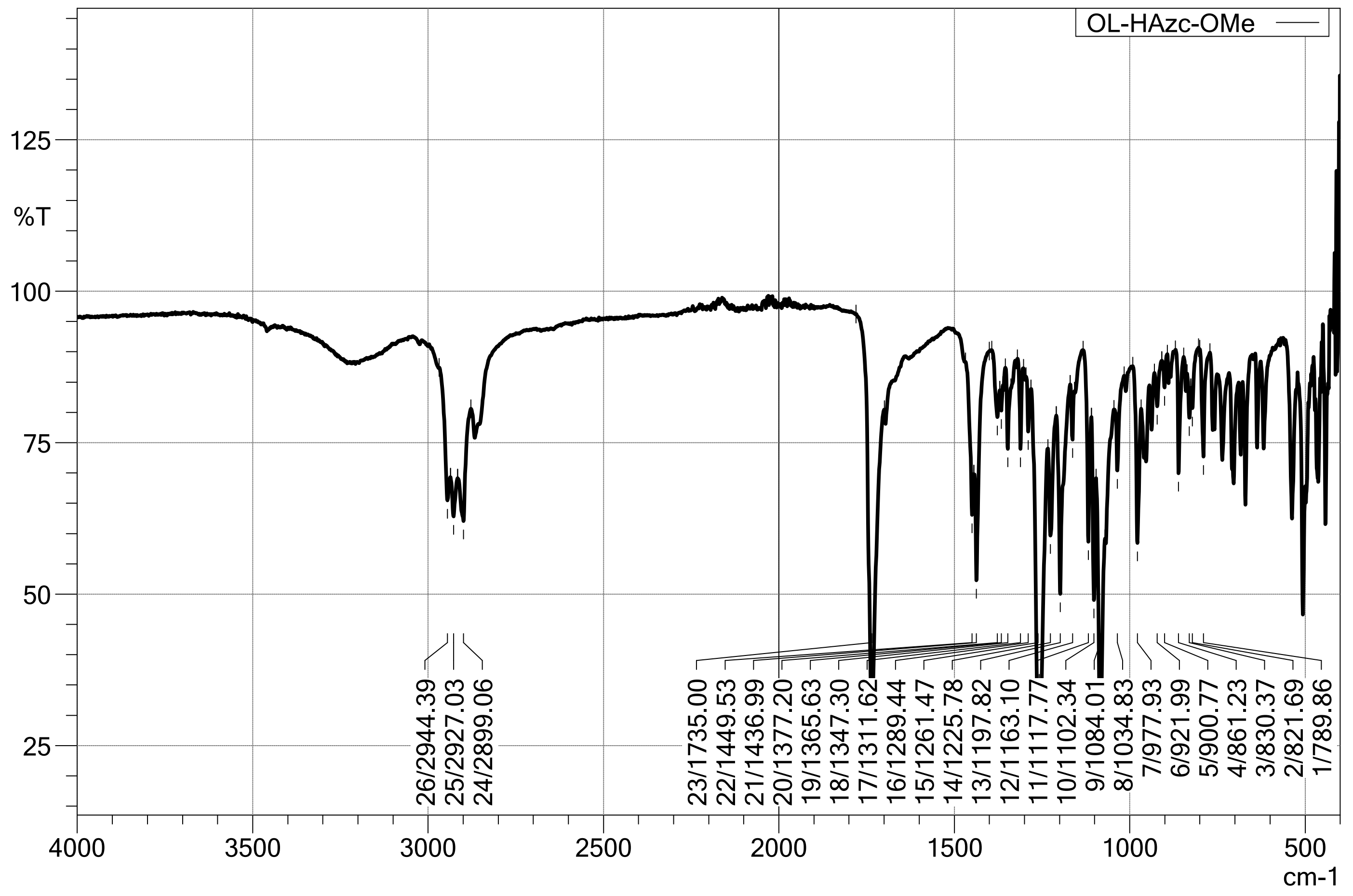

C:¥LabSolutions¥LabSolutionsIR¥Data¥Miller_OliviaL¥OL−HAzc−OMe.ispd

	Item	Value
2	Sample name	
3	Sample ID	
4	Option	
5	Intensity Mode	%Transmittance
6	Apodization	Happ-Genzel
9	No. of Scans	32
10	Resolution	2 cm-1

	Peak	Intensity	Corr. Intensity	Base (H)	Base (L)	Area	Corr. Area	Comment
1	789.86	72.71	17.53	800.47	771.54	437.220	152.705	
2	821.69	80.69	4.60	825.55	804.33	279.944	12.427	
3	830.37	79.14	5.99	836.16	825.55	189.092	32.455	
4	861.23	69.95	19.96	869.91	845.80	396.679	149.350	
5	900.77	84.09	4.99	908.49	893.06	196.778	28.269	
6	921.99	81.00	6.23	929.71	908.49	312.317	47.228	
7	977.93	58.43	25.28	991.43	967.32	608.334	225.811	
8	1034.83	70.43	11.92	1044.47	1015.54	565.417	81.679	
9	1084.01	24.63	39.90	1095.59	1070.51	1353.358	454.313	
10	1102.34	49.05	25.14	1109.09	1095.59	504.826	156.382	
11	1117.77	58.63	24.58	1133.20	1109.09	548.266	180.689	
12	1163.10	75.51	8.50	1169.85	1157.31	237.317	37.343	
13	1197.82	50.02	30.97	1209.39	1169.85	1126.692	417.008	
14	1225.78	59.69	16.09	1233.50	1209.39	718.158	157.817	

15	1261.47	30.48	12.57	1281.72	1257.61	1029.071	54.156	
16	1289.44	76.82	8.21	1295.22	1281.72	248.812	44.737	
17	1311.62	73.97	14.08	1320.30	1302.94	283.784	76.445	
18	1347.30	73.97	11.94	1354.05	1338.62	291.480	70.802	
19	1365.63	80.28	3.82	1370.45	1361.77	152.106	13.690	
20	1377.20	79.21	6.37	1393.59	1370.45	360.990	59.285	
21	1436.99	52.28	20.69	1443.74	1400.34	927.776	58.189	
22	1449.53	63.09	11.17	1467.85	1443.74	572.442	68.154	
23	1735.00	30.10	57.30	1780.33	1699.32	2311.546	1367.302	
24	2899.06	62.05	12.07	2915.46	2877.84	1104.142	158.493	
25	2927.03	62.81	6.41	2935.71	2915.46	681.812	58.053	
26	2944.39	65.47	8.62	2968.50	2935.71	781.250	71.596	
