# Supplementary material for: Site-Divergent Oxidations within Venerable Macrolide Antibiotic Scaffolds Unveil Compounds with Broad Spectrum and Anti-MRSA Activities
Source: ACS Cent Sci. 2026 Mar 17;12(3):375–82. doi: 10.1021/acscentsci.5c02343 (PMC13022725; doi:10.1021/acscentsci.5c02343)
Supplement: Supplementary file 6 [file oc5c02343_si_006.zip › Catalyst and SI Compound Characterization/C3 - HAzc(OMe)-Pro-OMe/IR/OL-II-099a.pdf]

SHIMADZU

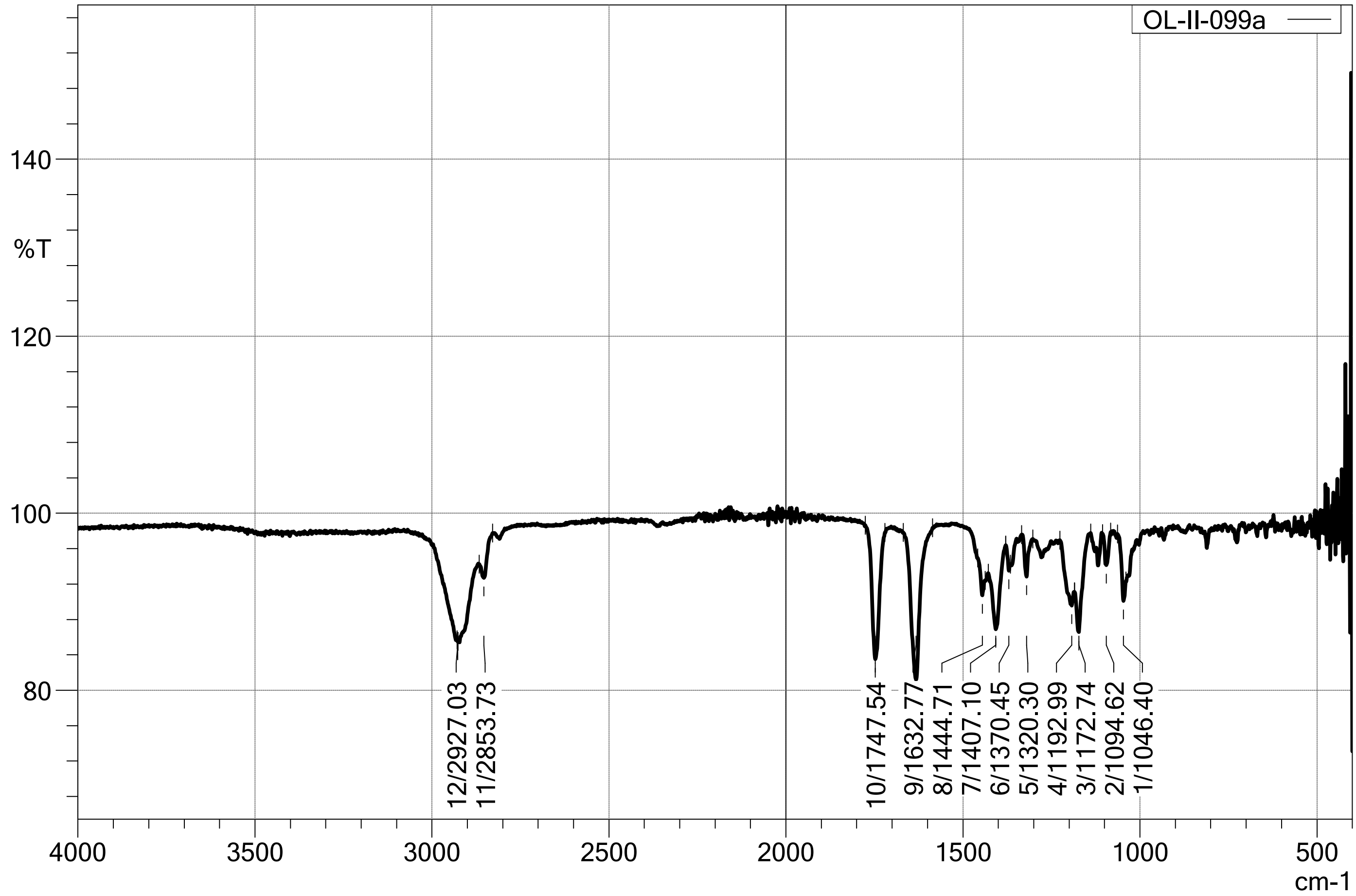

C:¥LabSolutions¥LabSolutionsIR¥Data¥Miller_OliviaL¥OL-II-099a.ispd

	Item	Value
2	Sample name	
3	Sample ID	
4	Option	
5	Intensity Mode	%Transmittance
6	Apodization	Happ-Genzel
9	No. of Scans	32
10	Resolution	2 cm-1

	Peak	Intensity	Corr. Intensity	Base (H)	Base (L)	Area	Corr. Area	Comment
1	1046.40	90.09	3.73	1063.76	1039.65	146.931	26.113	
2	1094.62	94.15	3.64	1105.23	1083.05	92.128	43.216	
3	1172.74	86.57	6.24	1184.31	1138.99	333.868	81.825	
4	1192.99	89.56	2.77	1225.78	1184.31	318.683	71.318	
5	1320.30	92.84	4.54	1333.80	1302.94	133.408	51.610	
6	1370.45	93.52	1.50	1379.13	1365.63	70.568	7.471	
7	1407.10	86.91	7.70	1428.31	1379.13	424.130	169.530	
8	1444.71	90.73	2.61	1458.21	1436.99	155.804	21.956	
9	1632.77	81.19	16.87	1668.45	1585.51	636.370	479.036	
10	1747.54	83.50	14.73	1775.51	1720.53	434.644	338.017	
11	2853.73	92.71	2.72	2866.27	2828.66	190.519	40.408	
12	2927.03	85.47	0.11	2928.96	2926.06	41.811	0.182	
