# Supplementary material for: Site-Divergent Oxidations within Venerable Macrolide Antibiotic Scaffolds Unveil Compounds with Broad Spectrum and Anti-MRSA Activities
Source: ACS Cent Sci. 2026 Mar 17;12(3):375–82. doi: 10.1021/acscentsci.5c02343 (PMC13022725; doi:10.1021/acscentsci.5c02343)

==== Shimadzu LabSolutions Browser Report ====

PDA Chromatogram(OL-II-099a.lcd)

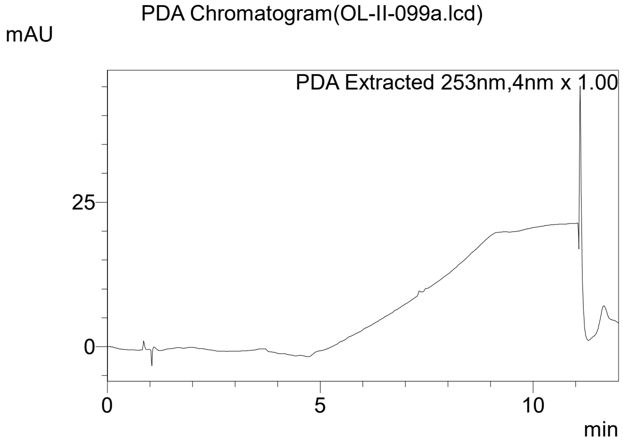

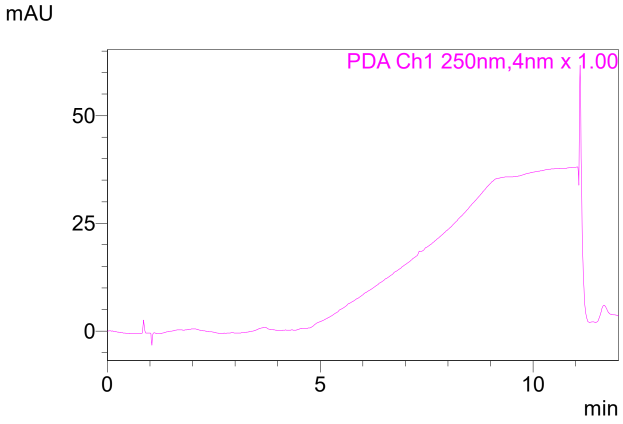

MS Chromatogram(OL-II-099a.lcd)

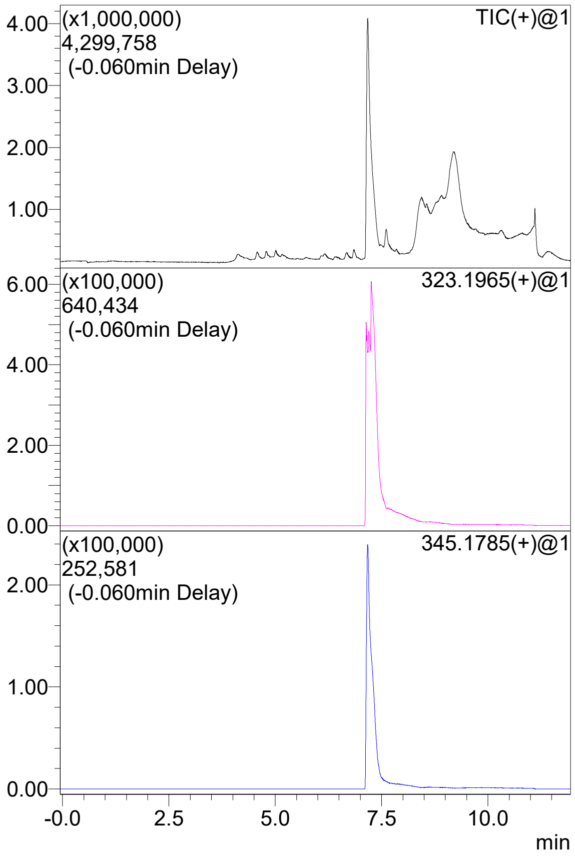

MS Spectrum(OL-II-099a.lcd)

Ret. Time: 1-1(E+) [7.370->7.438]

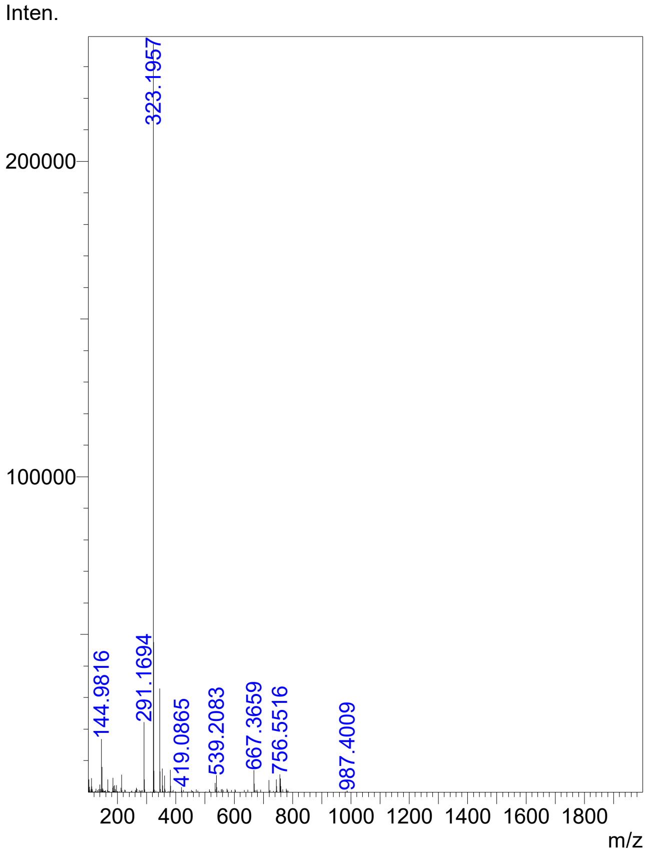

MS Spectrum(OL-II-099a.lcd)

Ret. Time: 1-1(E+) [7.370->7.438]

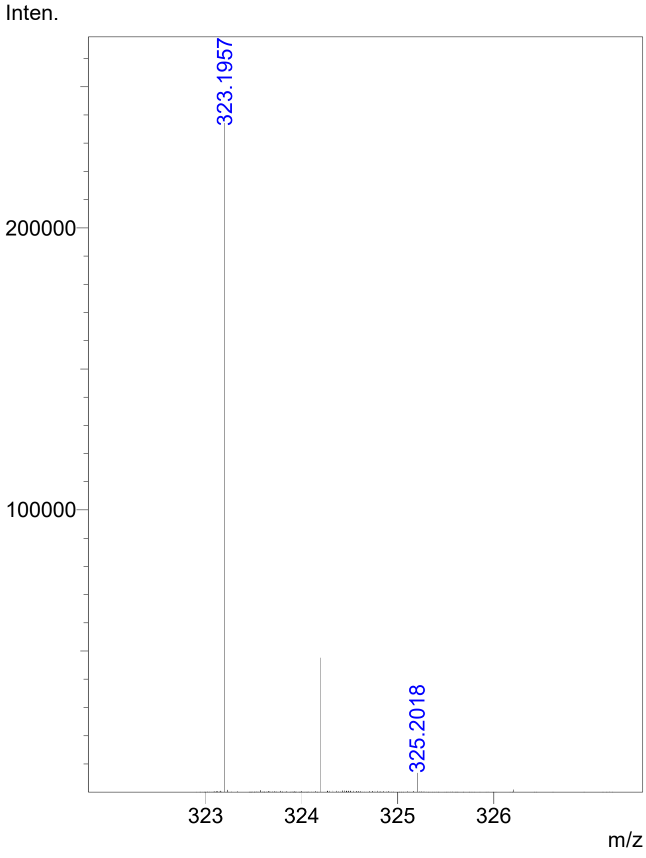

Supplement: Supplementary file 6 [file oc5c02343_si_006.zip › Catalyst and SI Compound Characterization/C3 - HAzc(OMe)-Pro-OMe/HRMS/OL-II-099a.pdf]
