# Supplementary material for: Site-Divergent Oxidations within Venerable Macrolide Antibiotic Scaffolds Unveil Compounds with Broad Spectrum and Anti-MRSA Activities
Source: ACS Cent Sci. 2026 Mar 17;12(3):375–82. doi: 10.1021/acscentsci.5c02343 (PMC13022725; doi:10.1021/acscentsci.5c02343)
Supplement: Supplementary file 6 [file oc5c02343_si_006.zip › Catalyst and SI Compound Characterization/S13/IR/OL-III-155.pdf]

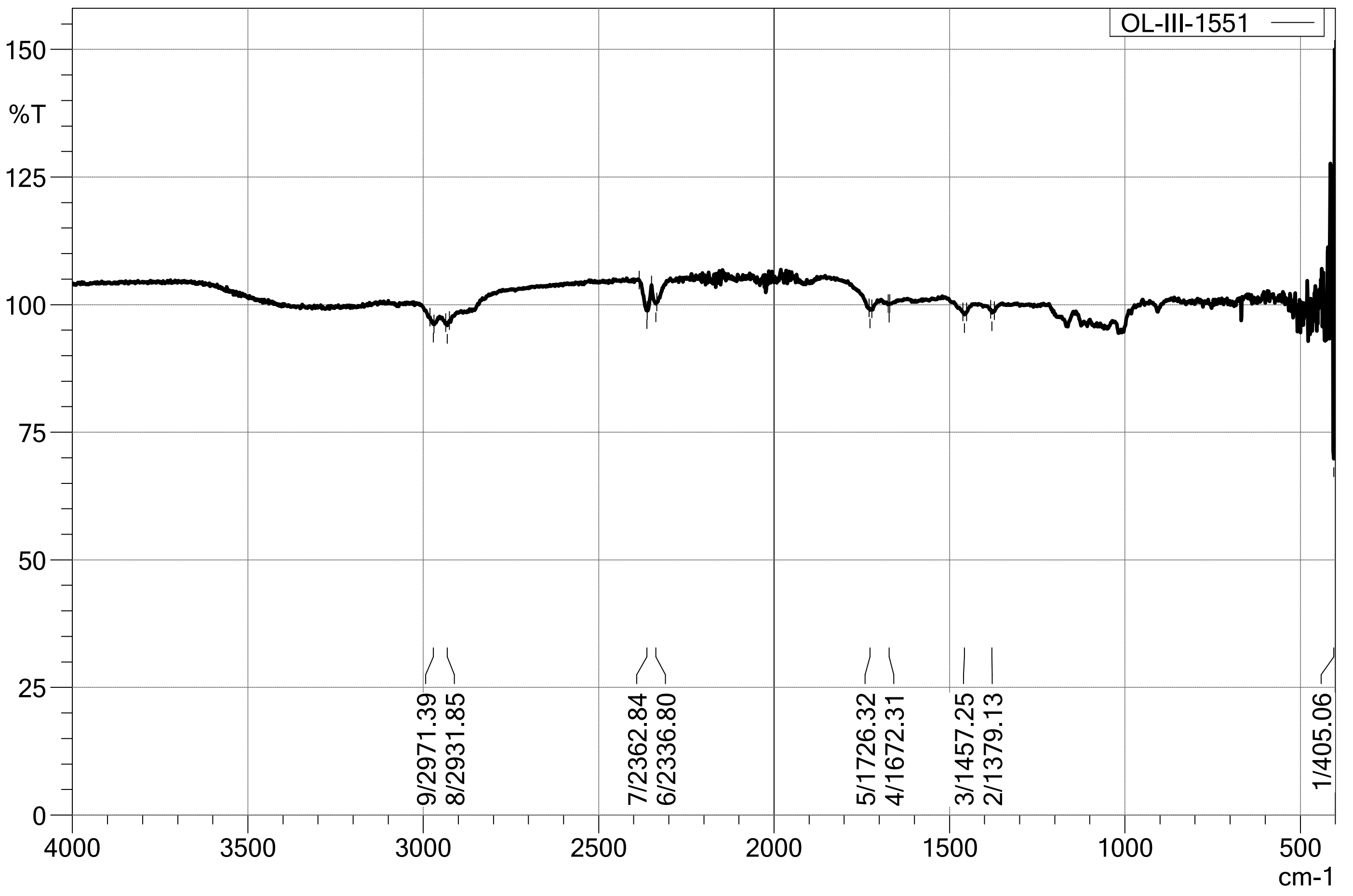

C:¥LabSolutions¥LabSolutionsIR¥Data¥Miller_OliviaL¥OL-III-1551.ispd

	Item	Value
2	Sample name	NY-636-2-p3
3	Sample ID	
4	Option	
5	Intensity Mode	%Transmittance
6	Apodization	Happ-Genzel
9	No. of Scans	20
10	Resolution	2 cm-1

	Peak	Intensity	Corr. Intensity	Base (H)	Base (L)	Area	Corr. Area	Comment
1	405.06	69.78	68.07	407.95	402.16	-12.738	206.303	
2	1379.13	98.39	0.58	1382.98	1372.38	14.224	3.037	
3	1457.25	98.01	0.58	1462.07	1451.46	18.079	3.156	
4	1672.31	100.07	0.14	1674.24	1670.38	-0.628	0.196	
5	1726.32	98.93	0.36	1729.21	1721.50	6.991	1.374	
6	2336.80	100.16	0.97	2349.34	2333.91	-18.446	15.303	
7	2362.84	98.82	0.32	2384.06	2361.88	-40.186	0.673	
8	2931.85	95.92	0.72	2935.71	2925.10	38.694	3.708	
9	2971.39	96.19	0.27	2981.04	2969.46	38.375	2.358	
