# Supplementary material for: Site-Divergent Oxidations within Venerable Macrolide Antibiotic Scaffolds Unveil Compounds with Broad Spectrum and Anti-MRSA Activities
Source: ACS Cent Sci. 2026 Mar 17;12(3):375–82. doi: 10.1021/acscentsci.5c02343 (PMC13022725; doi:10.1021/acscentsci.5c02343)

C:\Xcalibur\...\OL-III-155_pos_v1 10/24/25 16:32:44

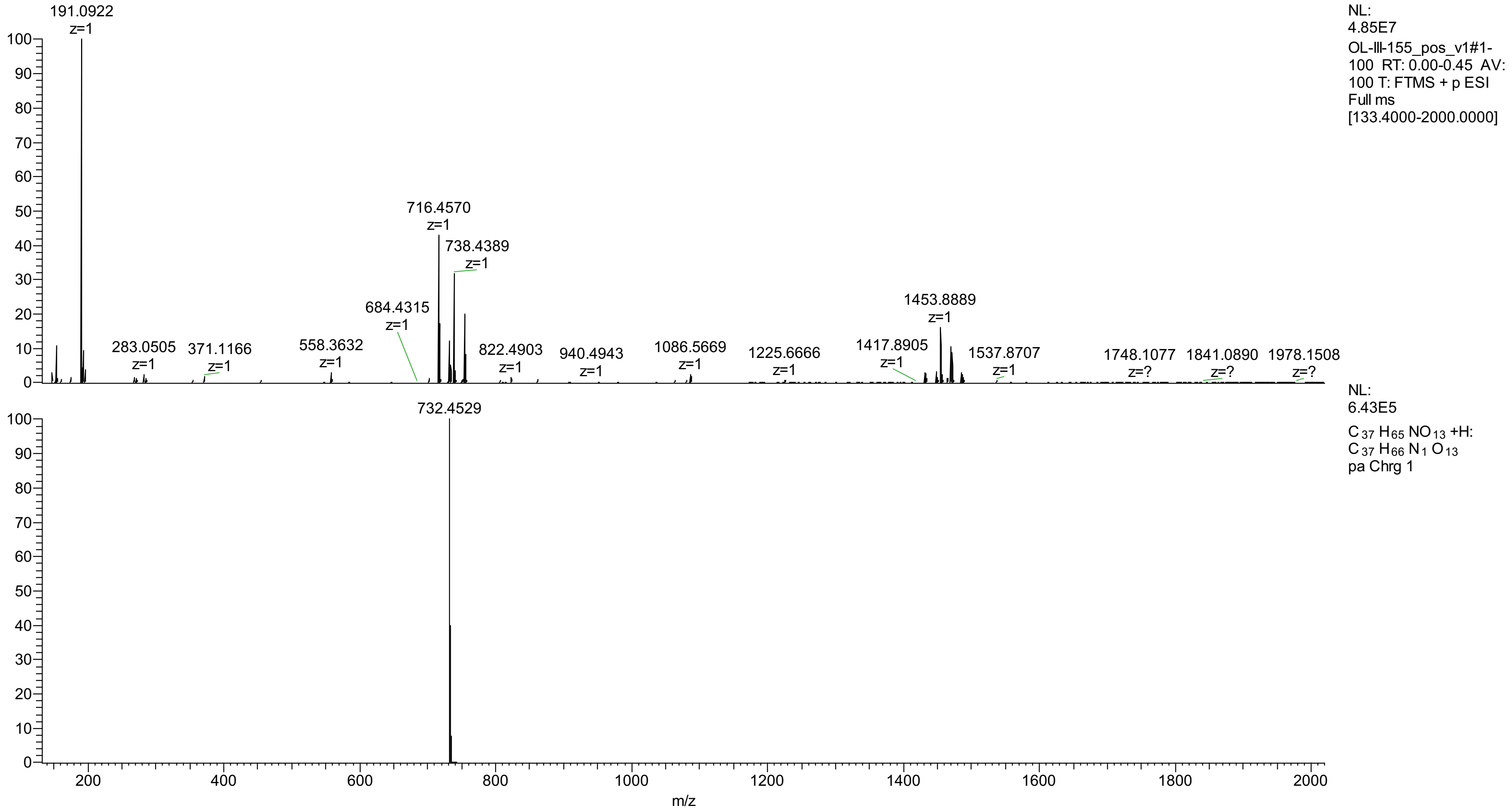

C:\Xcalibur\...\OL-III-155_pos_v1 10/24/25 16:32:44

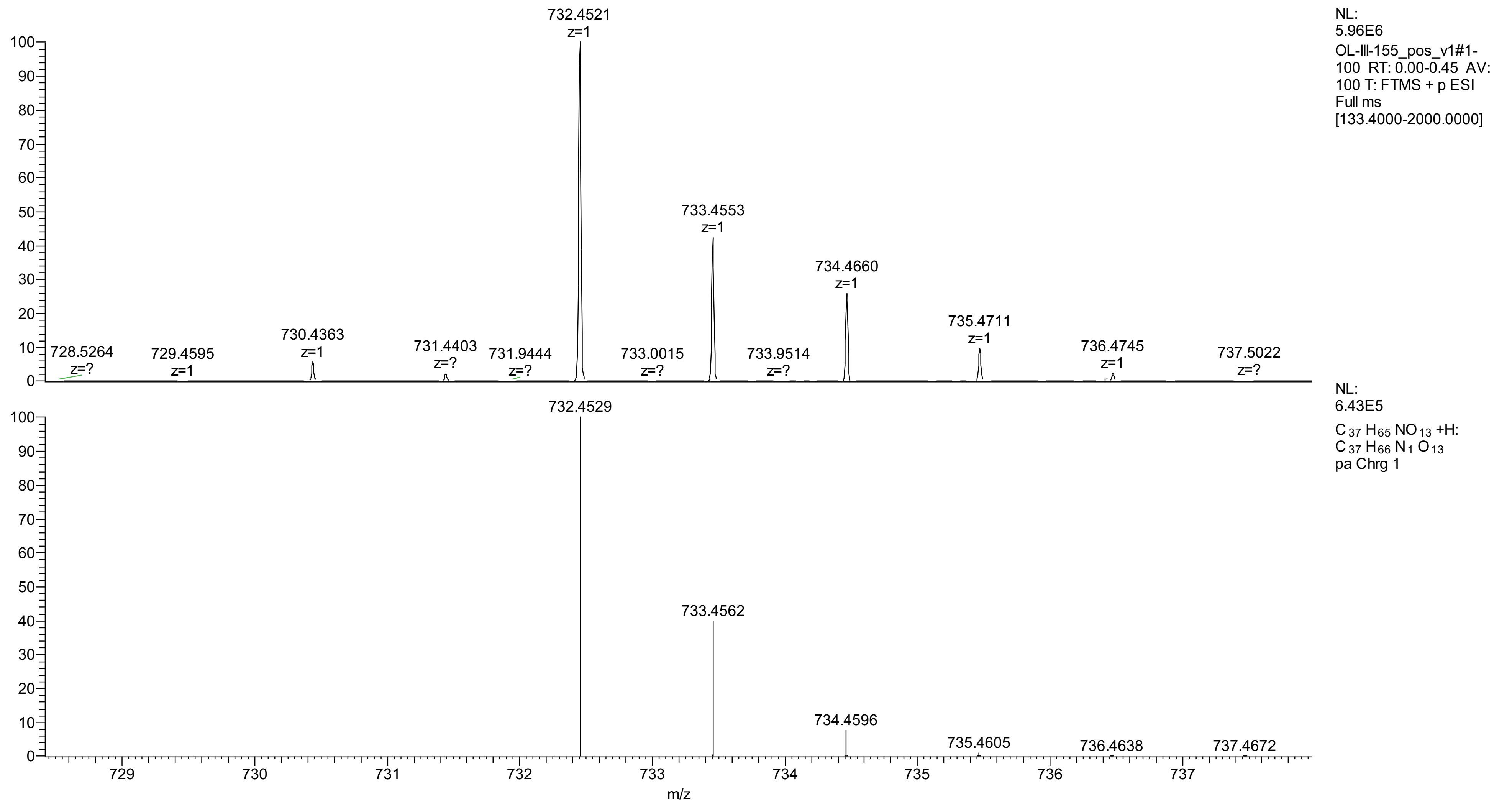

C:\Xcalibur\...\OL-III-155_pos_v2 10/24/25 16:34:30

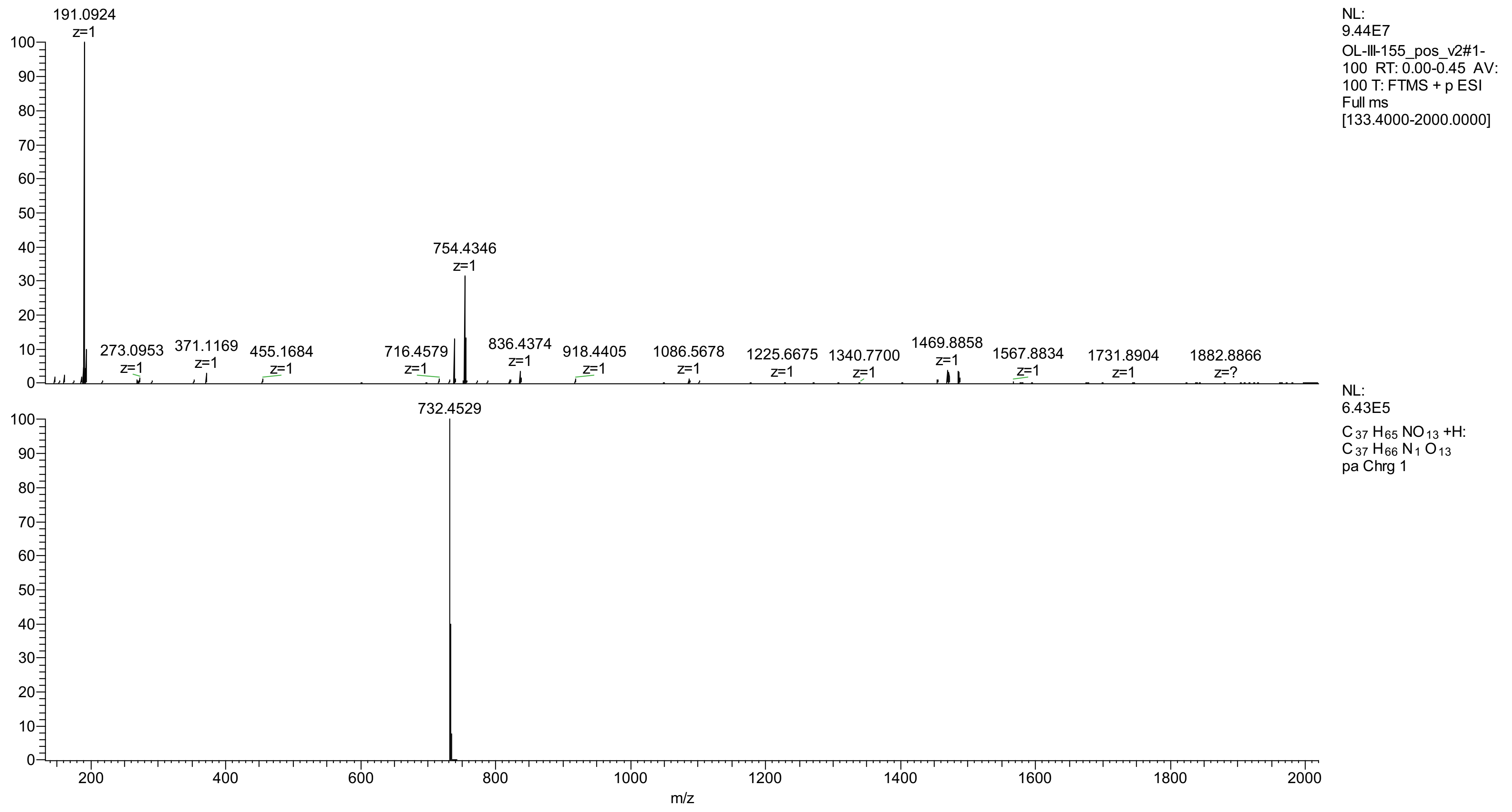

C:\Xcalibur\...\OL-III-155_pos_v2 10/24/25 16:34:30

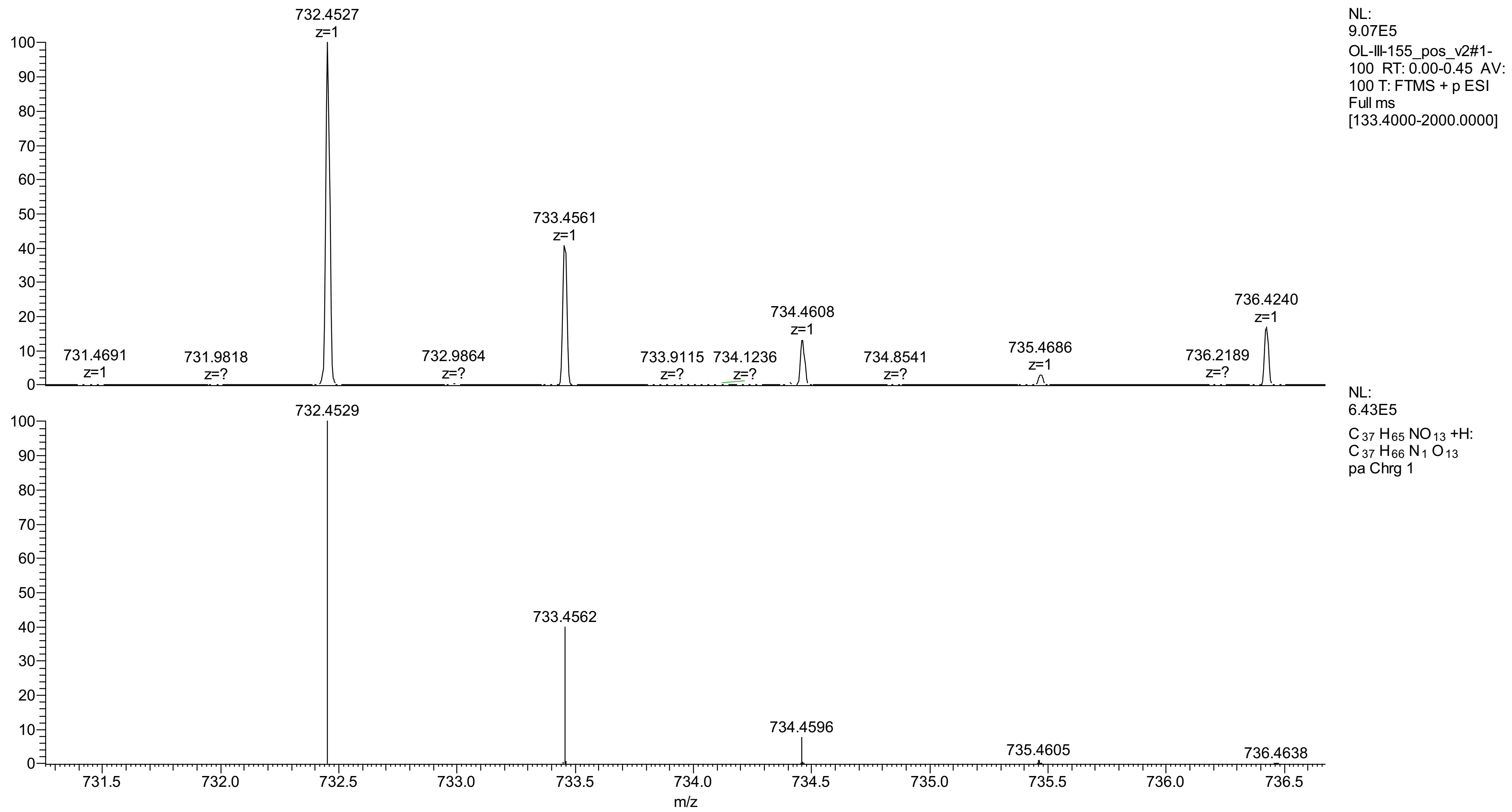

Supplement: Supplementary file 6 [file oc5c02343_si_006.zip › Catalyst and SI Compound Characterization/S13/HRMS/OL-III-155_pos_v1.RAW.pdf]
