# Supplementary material for: Site-Divergent Oxidations within Venerable Macrolide Antibiotic Scaffolds Unveil Compounds with Broad Spectrum and Anti-MRSA Activities
Source: ACS Cent Sci. 2026 Mar 17;12(3):375–82. doi: 10.1021/acscentsci.5c02343 (PMC13022725; doi:10.1021/acscentsci.5c02343)
Supplement: Supplementary file 6 [file oc5c02343_si_006.zip › Catalyst and SI Compound Characterization/S14/HRMS/OL-III-146-147-155_pos_v1.RAW.pdf]

C:\Xcalibur\...\OL-III-146_pos_v1 10/24/25 16:27:05

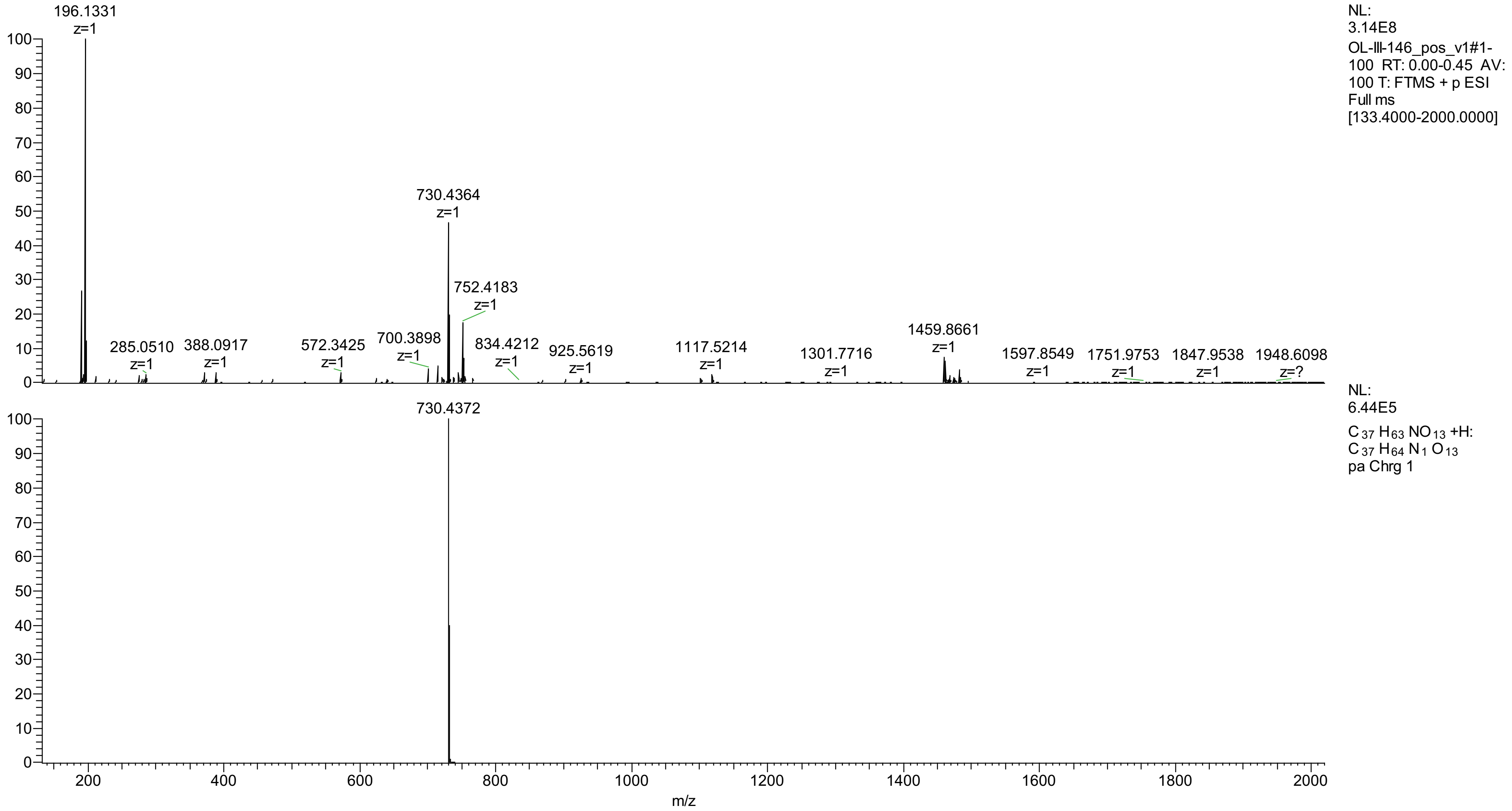

C:\Xcalibur\...\OL-III-146_pos_v1   10/24/25 16:27:05

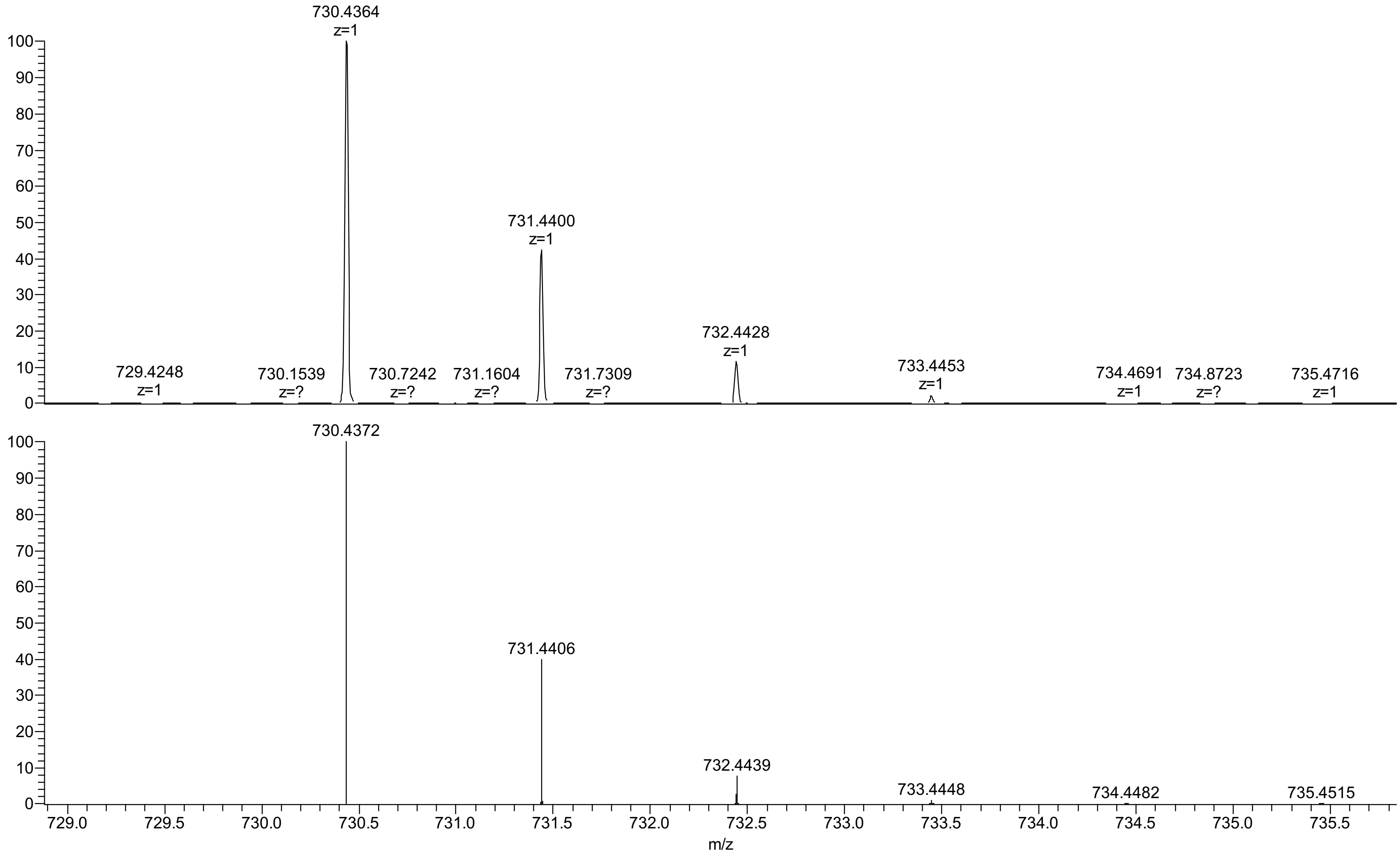

NL:
1.46E8
OL-III-146_pos_v1#1-100 RT: 0.00-0.45 AV: 100 T: FTMS + p ESI Full ms [133.4000-2000.0000]

NL:
6.44E5
$C_{37}H_{63}NO_{13}$ +H:
$C_{37}H_{64}N_1O_{13}$
pa Chrg 1
