# Supplementary material for: Site-Divergent Oxidations within Venerable Macrolide Antibiotic Scaffolds Unveil Compounds with Broad Spectrum and Anti-MRSA Activities
Source: ACS Cent Sci. 2026 Mar 17;12(3):375–82. doi: 10.1021/acscentsci.5c02343 (PMC13022725; doi:10.1021/acscentsci.5c02343)
Supplement: Supplementary file 6 [file oc5c02343_si_006.zip › Catalyst and SI Compound Characterization/S12/IR/OL-III-147.pdf]

SHIMADZU

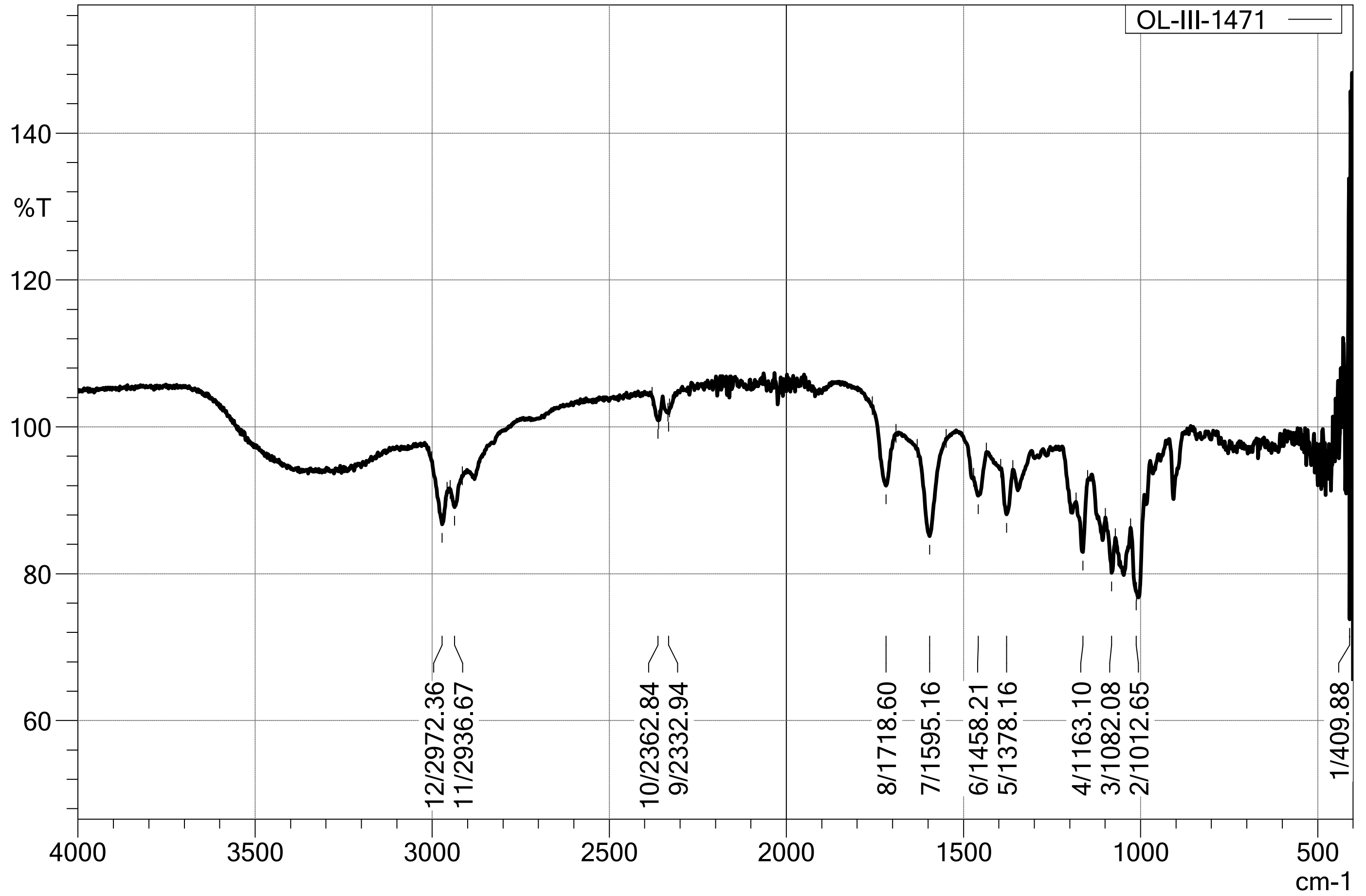

C:¥LabSolutions¥LabSolutionsIR¥Data¥Miller_OliviaL¥OL-III-1471.ispd

	Item	Value
2	Sample name	NY-636-2-p3
3	Sample ID	
4	Option	
5	Intensity Mode	%Transmittance
6	Apodization	Happ-Genzel
9	No. of Scans	20
10	Resolution	2 cm-1

	Peak	Intensity	Corr. Intensity	Base (H)	Base (L)	Area	Corr. Area	Comment
1	409.88	73.80	64.79	411.81	406.99	-26.818	164.986	
2	1012.65	77.54	0.54	1028.08	1011.68	309.133	12.761	
3	1082.08	80.13	5.82	1099.44	1071.48	457.709	74.172	
4	1163.10	82.98	8.60	1182.38	1149.59	417.563	132.654	
5	1378.16	88.10	6.18	1394.56	1360.80	308.036	114.836	
6	1458.21	90.64	3.76	1471.71	1435.06	270.680	82.258	
7	1595.16	85.13	6.94	1630.84	1548.87	625.900	-26.776	Div
8	1718.60	92.00	8.71	1757.18	1690.64	206.556	273.101	
9	2332.94	101.87	0.26	2333.91	2330.05	-8.159	0.654	
10	2362.84	100.90	0.39	2379.24	2360.91	-42.201	4.605	
11	2936.67	89.08	3.09	2949.21	2914.49	313.326	50.380	
12	2972.36	86.73	5.89	3001.29	2957.89	419.623	129.880	
