# Supplementary material for: Site-Divergent Oxidations within Venerable Macrolide Antibiotic Scaffolds Unveil Compounds with Broad Spectrum and Anti-MRSA Activities
Source: ACS Cent Sci. 2026 Mar 17;12(3):375–82. doi: 10.1021/acscentsci.5c02343 (PMC13022725; doi:10.1021/acscentsci.5c02343)
Supplement: Supplementary file 6 [file oc5c02343_si_006.zip › Catalyst and SI Compound Characterization/HAzc(OMe)-OMe/IR/OL-II-253.pdf]

SHIMADZU

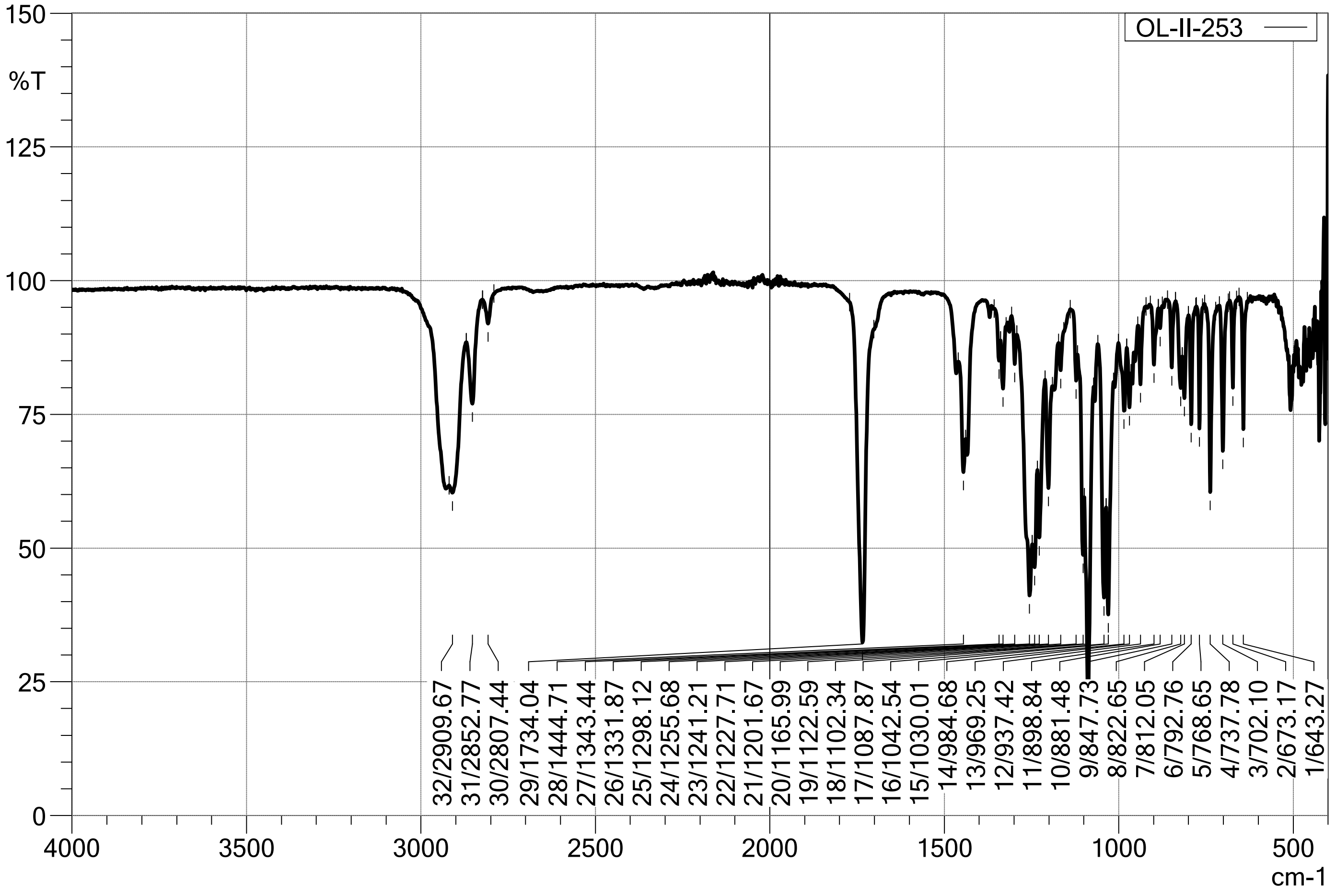

C:¥LabSolutions¥LabSolutionsIR¥Data¥Miller_OliviaL¥OL-II-253.ispd

	Item	Value
2	Sample name	
3	Sample ID	
4	Option	
5	Intensity Mode	%Transmittance
6	Apodization	Happ-Genzel
9	No. of Scans	32
10	Resolution	2 cm-1

	Peak	Intensity	Corr. Intensity	Base (H)	Base (L)	Area	Corr. Area	Comment
1	643.27	72.26	24.30	654.84	631.70	214.692	135.018	
2	673.17	80.00	16.34	682.81	662.56	155.909	81.792	
3	702.10	68.17	27.40	711.75	685.71	329.716	216.237	
4	737.78	60.46	34.51	754.18	722.35	434.929	275.524	
5	768.65	72.35	22.72	777.33	759.97	201.682	116.152	
6	792.76	73.18	20.92	800.47	779.25	239.514	118.629	
7	812.05	78.04	10.37	817.83	800.47	244.446	65.523	
8	822.65	79.91	8.51	837.12	817.83	227.248	53.566	
9	847.73	83.76	12.52	860.27	837.12	169.215	83.551	
10	881.48	91.12	3.91	886.31	874.73	76.055	18.975	
11	898.84	84.30	10.89	909.45	886.31	207.254	95.234	
12	937.42	80.60	12.17	945.14	921.99	231.602	78.298	
13	969.25	76.31	8.22	976.96	964.42	234.197	47.090	
14	984.68	75.67	12.06	1001.07	976.96	412.955	121.104	

15	1030.01	37.56	26.45	1035.79	1014.57	873.197	222.809	
16	1042.54	40.75	25.40	1059.90	1035.79	864.050	210.017	
17	1087.87	21.88	46.04	1098.48	1072.44	1334.229	548.544	
18	1102.34	48.73	16.12	1117.77	1098.48	589.353	65.706	
19	1122.59	81.28	6.83	1138.99	1117.77	210.804	7.625	
20	1165.99	83.27	6.03	1172.74	1157.31	199.817	36.773	
21	1201.67	61.24	19.70	1211.32	1190.10	584.490	178.864	
22	1227.71	51.96	17.03	1233.50	1211.32	756.285	158.090	
23	1241.21	46.40	10.79	1247.97	1233.50	697.878	85.334	
24	1255.68	41.14	16.42	1292.33	1247.97	1541.171	225.024	
25	1298.12	84.42	6.89	1306.80	1292.33	159.627	38.887	
26	1331.87	79.82	10.16	1338.62	1323.19	226.562	74.445	
27	1343.44	85.04	5.36	1356.95	1338.62	170.319	20.367	
28	1444.71	64.18	10.17	1460.14	1438.92	540.906	66.479	
29	1734.04	32.36	60.89	1771.65	1702.21	1973.377	1519.214	
30	2807.44	91.99	5.01	2823.83	2791.04	164.042	65.648	
31	2852.77	76.99	14.48	2870.13	2823.83	583.655	234.663	
32	2909.67	60.36	6.61	2919.31	2870.13	1402.473	178.191	
