# Supplementary material for: Site-Divergent Oxidations within Venerable Macrolide Antibiotic Scaffolds Unveil Compounds with Broad Spectrum and Anti-MRSA Activities
Source: ACS Cent Sci. 2026 Mar 17;12(3):375–82. doi: 10.1021/acscentsci.5c02343 (PMC13022725; doi:10.1021/acscentsci.5c02343)
Supplement: Supplementary file 6 [file oc5c02343_si_006.zip › Catalyst and SI Compound Characterization/C4 - HAzc(OMe)-Pro-Aib-Phe-OMe/IR/OL-II-033.pdf]

SHIMADZU

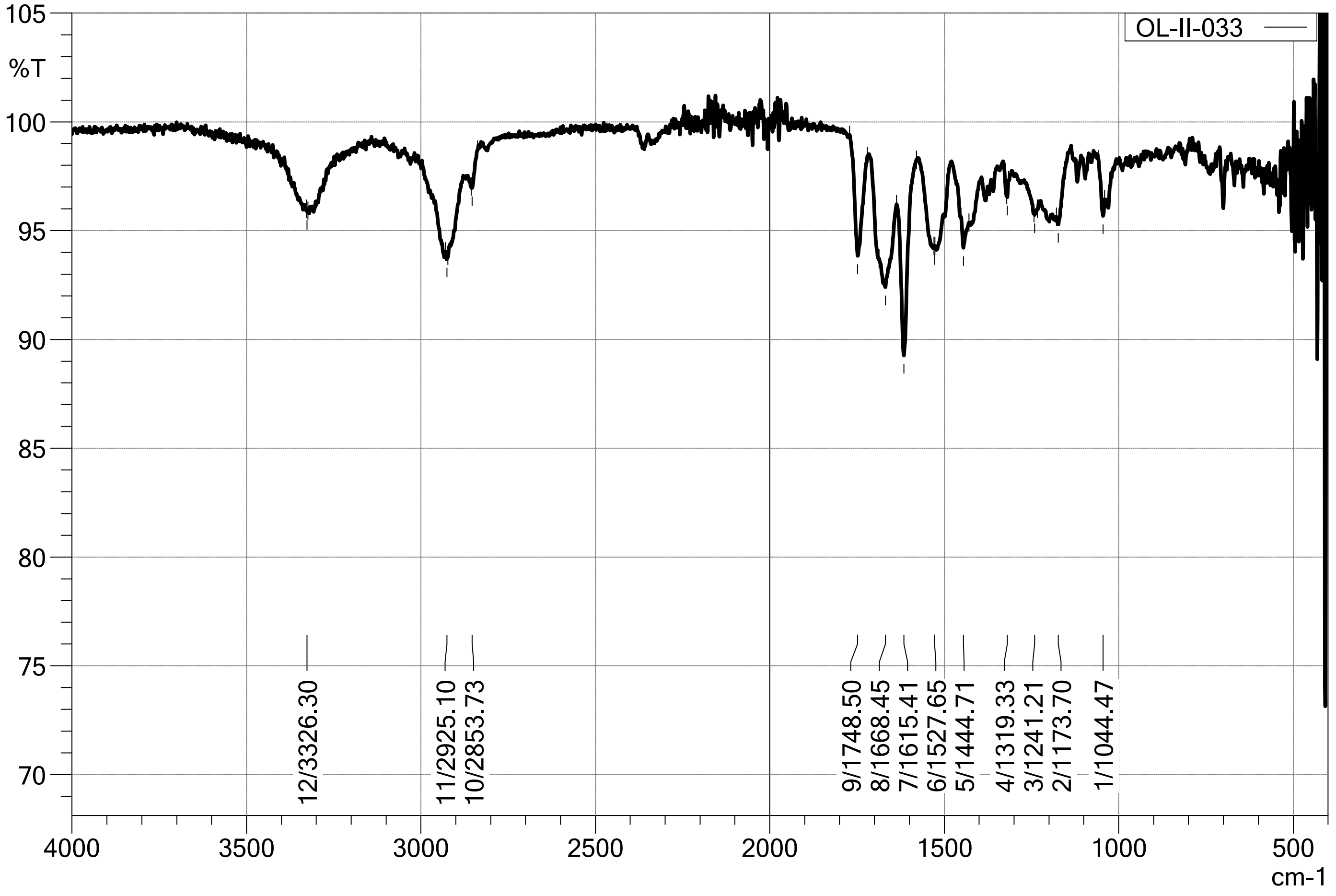

C:¥LabSolutions¥LabSolutionsIR¥Data¥Miller_OliviaL¥OL-II-033.ispd

	Item	Value
2	Sample name	
3	Sample ID	
4	Option	
5	Intensity Mode	%Transmittance
6	Apodization	Happ-Genzel
9	No. of Scans	32
10	Resolution	2 cm-1

	Peak	Intensity	Corr. Intensity	Base (H)	Base (L)	Area	Corr. Area	Comment
1	1044.47	95.68	1.17	1057.97	1040.61	57.293	11.455	
2	1173.70	95.28	0.78	1178.53	1157.31	83.183	10.376	
3	1241.21	95.73	0.12	1243.14	1233.50	40.112	0.614	
4	1319.33	96.54	0.33	1321.26	1315.47	18.273	0.843	
5	1444.71	94.21	1.34	1452.42	1429.28	117.635	13.449	
6	1527.65	94.27	0.02	1529.58	1526.68	16.537	0.024	
7	1615.41	89.27	7.71	1636.63	1579.73	312.872	155.467	
8	1668.45	92.40	1.01	1686.78	1660.74	183.952	14.800	
9	1748.50	93.85	5.12	1771.65	1720.53	176.306	120.874	
10	2853.73	96.96	0.25	2855.66	2837.34	40.546	1.689	
11	2925.10	93.69	0.21	2929.92	2923.17	41.634	0.775	
12	3326.30	95.88	0.08	3328.23	3324.37	15.733	0.160	
