# Supplementary material for: Site-Divergent Oxidations within Venerable Macrolide Antibiotic Scaffolds Unveil Compounds with Broad Spectrum and Anti-MRSA Activities
Source: ACS Cent Sci. 2026 Mar 17;12(3):375–82. doi: 10.1021/acscentsci.5c02343 (PMC13022725; doi:10.1021/acscentsci.5c02343)
Supplement: Supplementary file 6 [file OL-II-099b.pdf › Catalyst and SI Compound Characterization/C2 - HAzc(OMe)-Val-OMe/IR/OL-II-099b.pdf]

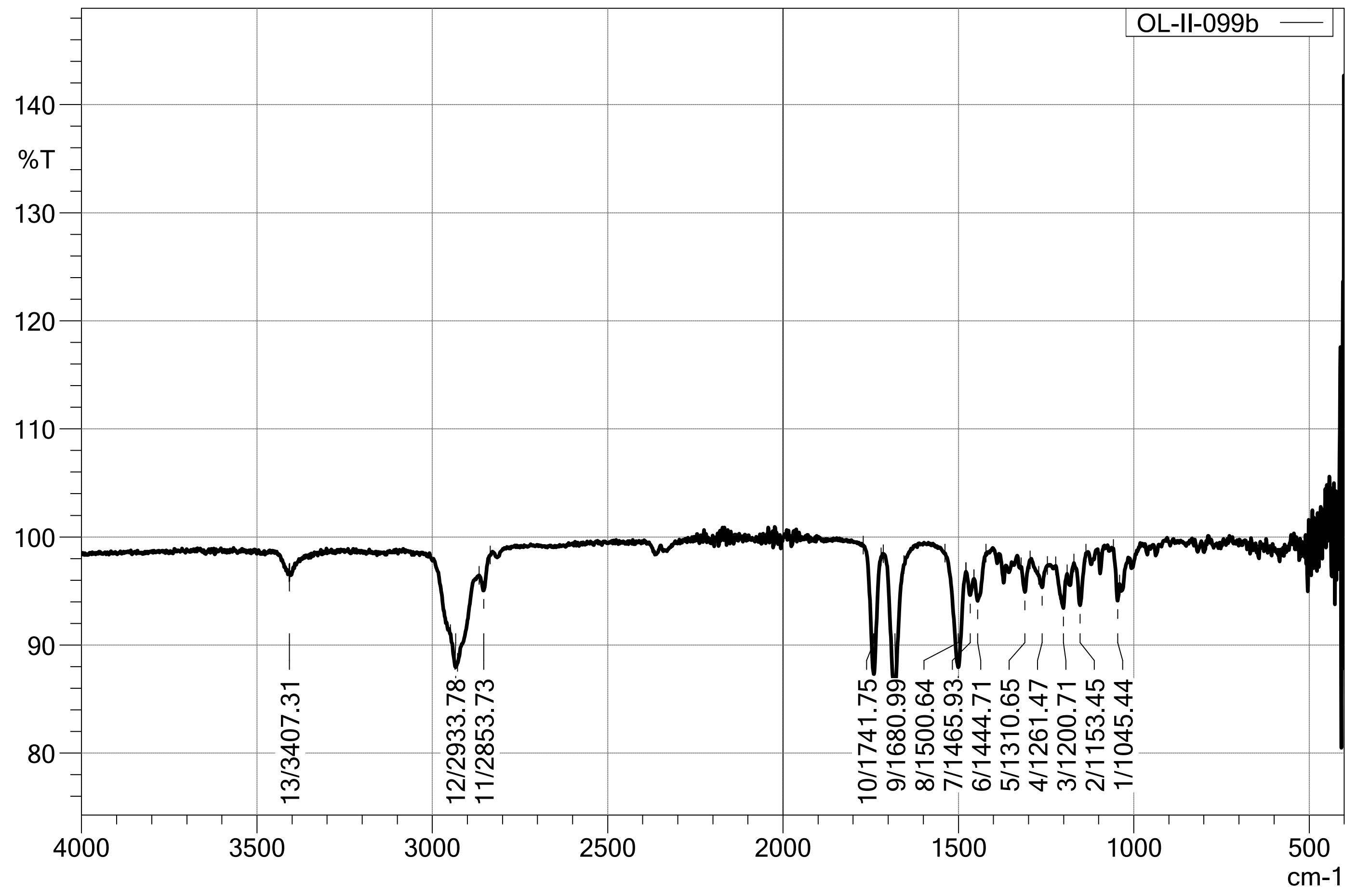

C:¥LabSolutions¥LabSolutionsIR¥Data¥Miller_OliviaL¥OL-II-099b.ispd

	Item	Value
2	Sample name	
3	Sample ID	
4	Option	
5	Intensity Mode	%Transmittance
6	Apodization	Happ-Genzel
9	No. of Scans	32
10	Resolution	2 cm-1

	Peak	Intensity	Corr. Intensity	Base (H)	Base (L)	Area	Corr. Area	Comment
1	1045.44	94.10	2.47	1057.97	1040.61	69.127	22.260	
2	1153.45	93.67	4.36	1170.81	1136.09	132.605	64.447	
3	1200.71	93.43	3.40	1222.89	1190.10	155.093	55.310	
4	1261.47	95.34	1.45	1271.11	1246.04	92.311	14.566	
5	1310.65	94.90	2.60	1321.26	1295.22	94.016	30.338	
6	1444.71	94.08	2.81	1455.32	1421.56	140.975	50.745	
7	1465.93	94.61	1.83	1478.46	1455.32	101.814	19.963	
8	1500.64	87.94	9.48	1538.26	1478.46	382.432	241.766	
9	1680.99	85.73	12.17	1713.78	1654.95	420.189	299.860	
10	1741.75	87.32	11.29	1771.65	1720.53	286.710	220.401	
11	2853.73	95.04	2.16	2866.27	2834.44	110.806	27.922	
12	2933.78	87.95	1.20	2948.25	2927.99	221.537	13.264	
13	3407.31	96.66	0.05	3408.28	3406.35	6.401	0.048	
